# 10th Trends in Medical Mycology Held on 8 to 11 October 2021, Aberdeen, Scotland, Organized by the European Confederation of Medical Mycology (ECMM)

**DOI:** 10.3390/jof7110916

**Published:** 2021-10-28

**Authors:** Oliver A. Cornely, Neil Gow, Martin Hoenigl, Adilia Warris

**Affiliations:** 1 Excellence Center for Medical Mycology (ECMM), Department I of Internal Medicine, Faculty of Medicine and University Hospital Cologne, University of Cologne, 50923 Cologne, Germany; oliver.cornely@uk-koeln.de; 2 Cologne Excellence Cluster on Cellular Stress Responses in Aging-Associated Diseases (CECAD), Chair Translational Research, Faculty of Medicine and University Hospital Cologne, University of Cologne, 50923 Cologne, Germany; 3 Clinical Trials Centre Cologne (ZKS Köln), Faculty of Medicine and University Hospital Cologne, University of Cologne, 50923 Cologne, Germany; 4 Partner Site Bonn-Cologne, German Centre for Infection Research (DZIF), 50923 Cologne, Germany; 5 Medical Research Council Centre for Medical Mycology, University of Exeter, Geoffrey Pope Building, Stocker Road, Exeter EX4 4QD, UK; n.gow@exeter.ac.uk; 6 Division of Infectious Diseases, Department of Internal Medicine, Medical University of Graz, 8036 Graz, Austria; martin.hoenigl@medunigraz.at; 7 Division of Infectious Diseases and Global Public Health, University of California San Diego, San Diego, CA 92092, USA

## Introduction to the Scientific Programme

**Plenary Sessions:**

No session will be held in parallel to these sessions.

 *Plenary sessions are indicated by the prefix: PS*

**Symposia:**

Each day, symposia will convene renowned speakers from several continents, who will cover a wide range of recent developments in their fields.

 *Symposia are indicated by the prefix: S*

A part of the symposia includes a selected submitted abstract.

**Quiz the Expert Sessions:**

The audience will actively participate in these small sessions.

 *Meet the expert sessions are indicated by the prefix: Q*

**Poster Sessions:**

All poster boards are situated on the exhibition floor of the congress centre and in the online platform. The poster exhibition is open to all participants during the entire congress. The numbers on the poster boards correspond with the abstract numbers in this abstract supplement.

All authors of odd poster numbers must be present at their poster on Saturday 9 October, from 11:00 to 12:00. All authors of even poster numbers must be present at their poster on Sunday 10 October, from 11:00 to 12:00.

 *All posters are indicated by the prefix: P*

## PS1.2 Highlights of Clinical Trials in Medical Mycology

**Emeritus George Petrikkos**

**Objectives:** Invasive fungal infections (IFIs) are associated with high all-cause mortality (ACM) despite the discovery of newer and less toxic antifungals, compared to the old conventional Amphotericin B (AmB), since the last 50 years. On the other hand limited clinical trials have been conducted for the treatment and prevention of the most common IFIs like invasive candidiasis and invasive aspergillosis and none for mucormycosis and other rare IFIs.

The objective of this presentation is to highlights the most important Randomized Clinical Trials (RCTs), regarding treatment of invasive candidiasis and aspergillosis, which are the most common IFIs clinical trials in medical mycology.

**Materials & Methods:** Rewew of the literature, of the last 20 years, in PubMed, for the most important RCTs, regarding treatment of invasive candidiasis and aspergillosis, which are the most common IFIs.

**Results:** Invasive candidiasis phase 3 trials with azoles and candins showed ACM 10–18% at day 30 and invasive aspergillosis phase 3 trials azoles showed ACM ~20% at 6-wks; ~30% at 12-wks.

IFI mortality improved with lipid-AmB, second generation mold active azoles and the candins.

Clinical trial patients are highly selected (e.g., patients who have complicated medical conditions are not eligible for trials) -real world mortality estimated to be higher at 30–95%.

Limited choice of antifungal drugs likely to be one of the drivers of high mortality. Only three main classes of antifungal drugs—much less than for antibiotics or antivirals, polyenes, azoles and candins have been discovered and the last new class of antifungal drug approved 20 years ago.

Antifungal clinical trials in mycology have always been difficult to recruit patients due to the relatively small number of patients with the specific invasive fungal infection (orphan population). Clinical trials in orphan populations require a global search for eligible patients, with a high number of sites (many will enroll zero patients) to offset low enrollment rates. Drugs to treat life-threatening rare diseases have been approved based on small data sets that support the substantial evidence of effectiveness required for approval of all drugs.

Clinical trials in IFIs are logistically complex, take a long time to conduct and require many resources (people and money)-enrollment costs are very high @ $100–200,000/patient.

Phase 3 RCTs in invasive candidiasis and invasive aspergillosis have historically required ~300–600 patients—however, the recruitment rates per site are very low invasive candidiasis 1.9 patients/site/year invasive aspergillosis 1.5 patients/site/year.

The most important RCTs, regarding treatment of invasive candidiasis and aspergillosis, which are the most common IFIs, are shown on the following tables.

Invasive Candidiasis—Randomized Clinical Trials.


**Study**

**Patients Enrolled**

**Years**

**Reference**
ISAVU vs. CASPO4502006–2015KullbergMICA vs. CASPO5952004–2006PappasANID vs. FLUCO2612003–2004ReboliMICA vs. AmB5312003–2004KuseVORI vs. AmB/FLUCO4221998–2003KullbergCASPO vs. AmB2391997–2001Mora-DuarteFLUCO vs. AmB/FLUCO2361995–1999Rex

More recently, clinical trials are being conducted in patients with limited or no treatment options-which increases the scarcity of patients who are eligible for the trials. These logistical challenges discourage sponsors and investors to develop new antifungal drugs.

**Conclusions:** Clinical trials in IFIs have historically been difficult to conduct-over the last few years it has become harder to enroll patients who are eligible/suitable for clinical trials; we anticipate this trend to continue.

Drugs to treat life-threatening rare diseases have been approved based on small data sets that support the substantial evidence of effectiveness required for approval of all drugs.

## PS2.1 Immune Recognition of Fungi: The Writing on the Wall

**Neil Gow**

Department of Biosciences, University of Exeter, Exeter EX4 4PY, UK

**Objectives:** The cell wall of a fungus is an exoskeleton composed of unique molecules that are unique to fungal cells and which are used by the immune system to induce antifungal responses. Immune surveillance and defence against potential fungal pathogens is based on the recognition of this suite of molecules in the fungal cell wall that are recognised by pattern recognition receptors of the innate immune system. Differences in the cell wall composition of different fungi and or the same fungus organisms growing in different morphologies and in differing environments generates a moving target for immune recognition.

**Materials & Methods:** We have used a variety of microscopic and imaging tools combined with, genetic and immunological methods to generate a new spatially accurate model of the cell wall and to explore how dynamic changes in the wall influence immune surveillance.

**Results:** We show that immune relevant epitopes can be diffuse or clustered, superficial or buried in the cell wall and they changed during batch culture and between yeast, hypha and other cellular morphologies. The immune reactivity of different fungal cell surfaces was not necessarily related to the phylogenetic relationship between organisms, or the relative virulence of different strains. These experiments demonstrate that the fungal cell surface is ordered, complex and dynamically changing, making immune recognition a challenging process requiring the concerted action of multiple receptors operating singly and in combination.

**Conclusions:** My presentation will focus on this work that demonstrates that describes recent advances that have generated a scaler model of the cell wall and show it behaves as an ordered and dynamically changing organelle that makes immune recognition a challenging process. Immune recognition requires the concerted action of multiple receptors operating singly and in combination.

**References**

1. Yadav, B.; Mora-Montes, H.M.; Wagener, J.; Cunningham, I.; West, L.; Haynes, K.; Brown, A.J.P.; Gow, N.R.R. Differences in fungal immune recognition by monocytes and macrophages: *N*-mannan can be a shield or activator of immune recognition. *Cell Surf.* **2020**, *6*, 100042, https://doi.org/10.1016/j.tcsw.2020.100042.
2. Lenardon, M.D.; Sood, P.; Dorfmueller, H.; Brown, A.P.J.; Gow, N.A.R. Scalar nanostructure of the *Candida albicans* cell wall; a molecular, cellular and ultrastructural analysis and interpretation. *Cell Surf.* **2020**, *6*, 100047, https://doi.org/10.1016/j.tcsw.2020.100047.

## PS2.2 *Aspergillus fumigatus* Cell Wall

**Isabel Valsecchi**

The fungal cell wall is a dynamic cellular compartment that must sense and adapt to different environmental stresses and opportunities.

In the case of *Aspergillus fumigatus*, this organelle consists mainly of polysaccharides, but also contains amylodogenic proteins, enzymes, pigments, as well as glycoproteins and GPI-anchored proteins, which together build the protection of fungal cells.

During the asexual life cycle of *A. fumigatus*, the different morphotypes maintain certain cell wall components, changing them in quantity, while others are specific to conidia or mycelium. In conidia, for example, we find that the hydrophobin RodA and the pigment DHN-melanin disappear in swollen conidia. On the other hand, the polysaccharide galactosaminogalactan is only present in hyphae and in the extracellular matrix, but is not present in conidia. The fibrillar core of polysaccharides consisting of branched β-(1,3) glucan cross-linked to chitin, galactomannan and β-(1,3)(1,4) glucan and embedded with α-(1,3) glucan polymers, is present in all morphotypes.

The composition and structures of some of these cell wall components will be described, as well as some techniques used for studying them.

## PS3.1 Influenza

**Paul Verweij**

Radboud University Medical Center

Severe influenza has emerged as risk to develop invasive pulmonary aspergillosis. The frequency of influenza associated pulmonary aspergillosis (IAPA) ranges, but may affect up to 1 in 5 patients that are admitted to the ICU with influenza. Predisposing host factors vary, but up to one third of patients have no underlying disease. In addition to alveolar disease, patients may present with invasive *Aspergillus* tracheobronchitis (IATB) where the infection is confined to the airways. Mortality rates are around 50%, but may be as high as 90% in IATB patients. Influenza was found to be an independent risk factor to develop IAPA and lytic effects of the virus on lung epithelium and subsequent immune dysregulation are believed to contribute to host susceptibility. A recent study indicated that IAPA was already present at ICU admission in 70% of patients (early IAPA), while the remainder of cases develop later on during ICU admission (late IAPA). A preventive strategy involving posaconazole prophylaxis failed due to early IAPA cases, but posaconazole may help to prevent late IAPA. Management of early IAPA is very challenging and might require empiric antifungal therapy in hospitals with high IAPA prevalence combined with a diagnostic work-up, with de-escalation in those patients in which negative *Aspergillus* tests.

## PS5.2 Tasting the Commensal *Candida albicans* in the Oral Mucosa

**Salomé LeibundGut-Landmann**

As part of the human microbiota, *Candida albicans* colonizes the mucosal surfaces of the human body. Commensalism results from the equilibrium between fungal growth and persistence on the host surface and the antifungal response preventing fungal overgrowth. The cytokine IL-17 is critical for controlling fungal load and preventing disease as illustrated by individuals with rare genetic defects in the IL-17 pathway that suffer from chronic mucocutaneous candidiasis. Stable maintenance of stable fungal commensalism requires long-lasting IL-17 production in vicinity of the epithelium. In my talk I will provide evidence that this is conferred by tissue resident memory (TRM) Th17 cells persisting in the *C. albicans*-colonized tissue and that TRM cells are sufficient for regulating the fungal load, without eliminating the fungus. While *C. albicans* can resist homeostatic levels of IL-17 to survive in barrier tissues, commensal growth of the fungus also depends upon fungus-intrinsic parameters. Natural isolates differ in their potential to stably colonize epithelial tissues.

In the second part of my talk, I will report on the identification of determinants of fungal commensalism. Our transcriptomic profiling of *C. albicans* in the oral mucosa of experimentally infected mice identified differentially expressed genes that are overexpressed in the commensal strain 101 in comparison to the highly virulent strain SC5314. Overexpression of one of the identified genes in strain SC5314 was sufficient to enhance persistence, and vice versa repression of this gene in strain 101 impaired fungal colonization in our experimental model. Together, my talk will highlight the multifaceted fungal and host mechanisms of regulation that allow *C. albicans* to establish commensalism at the epithelial host interface.

## S01.1 Coccidioidomycosis: Diagnostics

**Derek Bays and George R. Thompson**

**Abstract:** Coccidioidomycosis is the disease caused by the dimorphic fungus, *Coccidioides immitis* and *posadasii*. *Coccidioides* spp. is endemic to the Southwest of the United States, Mexico, and regions of South America, but the endemic region is expanding with climate change. Despite increasing incidence since the 1990s, diagnosis remains challenging with approximately 3-week delays in diagnosis even in endemic areas. The main stay of diagnosis remains serologic with immunodiffusion, complement fixation serology, and enzyme-linked immunosorbent assays (EIA) being the most used compared to newer lateral flow assay (LFA). Beyond the LFA, there are additional novel strategies to diagnose coccidioidomycosis including PCR, antibody profiles, and metabolite profiles. This review will focus on the current and future diagnostics for coccidioidomycosis while exploring their test performances.

## S01.2 Novel Non-Culture Diagnostics for Talaromycosis

**Thuy Le**

Talaromycosis caused by the dimorphic fungus *Talaromyces marneffei* is a leading cause of opportunistic infection and death in people with advanced HIV disease in Southeast Asia and southern China. The mortality on antifungal therapy in both HIV and non-HIV infected patients is as high as 30%. The current diagnosis relies on cultures of the pathogen which takes up to 14 days and is at best 70% sensitive. Late diagnosis increases mortality from 24% to 50%. This talk presents new data on the comparative performance of several novel non-culture-based assays for rapid diagnosis, including results of a ribosomal 5.8S real-time PCR assay, homebrew and commercial forms of the Mp1p enzyme immunoassays (EIAs), and a 4D1-monoclonal antibody (MAb)-based and Mp1p MAb-based point-of-care lateral flow assays (LFAs) current under development. The real-time PCR and antigen-detection assays show substantially higher sensitivity compared to blood cultures without sacrifices in specificity and represent highly-promising modalities for rapid diagnosis. The point-of-care LFAs in particular may be useful as a screening tool for pre-clinical asymptomatic infections.

## S01.3 Histoplasmosis

**Alessandro Pasqualotto**

*Histoplasma capsulatum* is a major killer in AIDS patients. Histoplasmosis is endemic in the Americas, and in many countries the disease leads to more deaths in AIDS than tuberculosis. Despite that, histoplasmosis is neglected in most parts of the world, in which patients have limited access to modern diagnostic and treatment modalities. This talk will review diagnostic strategies for histoplasmosis, with an emphasis on the detection of *Histoplasma* antigen in urine samples. In order to improve the prognosis of patients with histoplasmosis, there is an urgent need to improve access to diagnostics, particularly in the developing world.

## S01.4 Molecular Diagnosis of Sporotrichosis from Patients with Subcutaneous Lesions of Antioquia (Colombia) 1976–2021

**Erika Andrea Sánchez Cifuentes ^1^, Juan Guillermo McEwen Ochoa ^1,2^, Martha Eugenia Úran Jiménez ^1^, Laura Carolina Alvarez Acevedo ^1,3^ and Maria del Pilar Jiménez Álzate ^1^**

^1^ Medical Mycology Group, School of Medicine, University of Antioquia

^2^ Cellular & Molecular Biology Unit, Corporación para las investigaciones Biológicas

^3^ Dermatological Research Center CIDERM, School of Medicine, University of Antioquia

**Objectives:** Detect by chitinase PCR and Species-Specific PCR (SS PCR) the DNA of *Sporothrix* spp. in fresh tissue and Formalin Fixed Paraffin Embedded (FFPE) tissue from patients with sporotrichosis in Antioquia between 1976 and 2021.

**Materials & Methods:** Fresh tissue samples were taken by biopsy from 20 patients who arrived at the Medical Mycology Group, School of Medicine, University of Antioquia, with suspected sporotrichosis. Samples were cultured at room temperature and BHI supplemented, at 37 °C in 5% CO_2_. DNA extraction from fresh tissue was performed using the Mini Kit (Qiagen).

We selected 73 tissue samples from biopsy of patients with histopathological diagnosis between 1976 and 2021, distributed as follows: 52 samples of spotrichosis and 25 samples with other subcutaneous infectious diseases: histoplasmosis, chromoblastomycosis, leishmaniasis, paracoccidioidomycosis, cryptococcosis and necrotizing granulomatous tuberculosis, stored at the Dermatology Department at the Hospital San Vicente Fundación (HSVF). DNA extraction from FFPE tissue was performed using the DNA FFPE tissue kit (Qiagen).

Chitinase PCR was performed amplifying a 206 bp sequence of the chitinase gene. For species identification, species-specific primers were used, which amplify a 331 bp sequence for *S schenckii* s.str and 243 bp sequence for *S. globosa* of the calmodulin gen. For the SS PCR in tissue, a nested PCR was implemented using the primers CAL1 and CAL2 for the external sequence and the species-specific primers for the internal sequences.

**Results:** From the 20 samples of fresh tissue, 11 had positive culture for *Sporothrix* spp. and were positive for chitinase PCR. However, it was necessary to perform a second run using the product of the first PCR as sample, to increase the sensitivity of this PCR. It was not possible to isolate the fungus by culture from 3 samples due to bacterial contamination, however the chitinase nested PCR result for these three patients was positive. In 6 patients, both, the culture and the chitinase nested PCR were negative.

Nested SS PCR was applied to DNAs of positive chitinase nested PCR of fresh tissue samples. The results of these nested SS PCR were confirmed by the identification of species from the isolates recovered in culture. All were identified as *S. schenckii* s.str and there was 100% agreement with the results of nested SS PCR in tissue.

Of the 52 FFPE tissue samples with histopathological diagnosis of sporotrichosis, 25 were adequate for PCR and 30.7% (n: 16) of these were positive by chitinase nested PCR. All control tissues were negative for chitinase nested PCR.

**Conclusions:** Chitinase nested PCR has a good performance when applied to fresh and good quality paraffin tissue samples. However, PCR positivity decreased in samples stored in paraffin for more than 15 years. In fresh tissue samples, the chitinase nested PCR had 100% agreement with the culture results, as did the nested SS PCR. These two PCRs could be implemented as a diagnostic method that offers faster results in comparison to culture with the advantage of knowing directly from the sample the *Sporothrix* species that cause the disease.

## S01.5 Ex-Vivo and In-Vivo Production of Ros and Cytokine Profile of Laser-Irradiated Murine Neutrophiles in Infection by *Paracoccidioides brasiliensis*

**Bruno José Nascimento Gomes, Lauana Aparecida Santos, Nayara Andrade Dias, Vitor Roberto Souza and Eva Burger**

Federal University of Alfenas

**Introduction:** Paracoccidioidomycosis (PCM) is a severe granulomatous disease, which can affect any organ in the body. The etiologic agents of the disease are thermodimorphic fungi of the *Paracoccidioides brasiliensis* complex. Protective immunity in this disease is mainly excerted by the action of macrophages activated by cytokines produced by T lymphocytes, normally Th1 and Th17. PCM is characterized by the presence of an inflammatory infiltrate of polymorphonuclear/neutrophil leukocytes (PMNs), capable of combating fungi via the production of reactive oxygen specimens (EROS) and pro-inflammatory cytokines. PMNs from mice susceptible to infection produce less EROS, having less fungicidal capacity compared to resistant mouse cells. Stimulation of PMNs with low-level LASER therapy (LLLT) was able to increase the fungicidal capacity of these cells against *Candida albicans* and *P. brasiliensis*, making PMNs metabolically more active.

**Objectives:** Within this context, our objective was to evaluate the generation of EROS and cytokines by mature PMNs, obtained from subcutaneous air pouch exudate of *P. brasiliensis*-infected mice, treated or not with LLLT and cultured ex vivo and in vitro.

**Materials & Methods:** Female Swiss mice were inoculated in subcutaneous pouches with a suspension of 5 × 10^6^/mL yeast cells. The animals of the ex vivo experiments were irradiated with LLLT at alternate days, for seven days, and then the PMNs were collected for cultivation in RPMI medium. For the in vitro experiments, PMNs were collected from the air pouch, cultured in RPMI medium and irradiated with LLLT, in such a way that the LASER touched the bottom of the 12-well plate so that the radius struck perpendicularly to the its surface. In both experiments, the production of EROS was evaluated by luminol chemiluminescence assay, the mitochondrial activity by the MTT reduction method and the quantification of viable fungal cells through the counting of colony-forming units. The production of hydrogen peroxide and catalase activity were also measured. The concentration of IL-4, IL-6, IL-10, IL-12, IL-17 and Kc cytokines, was determined by capture ELISA tests, based on the use of specific monoclonal antibody pairs for each cytokine.

**Results:** The cells irradiated with LLLT, in both, ex vivo and in vitro experiments, showed higher mitochondrial activity and EROS production, including hydrogen peroxide, and lower viable fungal cell counts than those of the non-irradiated controls. In the in vitro experiments, there was an increase in the levels of IL-4 and KC in the treated groups, while there was no significant increase in the other cytokines. On the other hand, in the ex vivo model, LLLT promoted an increase in the production of IL-4, IL-6, IL-12 and IL-17 cytokines and a decrease in the concentrations of IL-10 and KC. The overall results indicate a possible immunomodulatory effect of LASER therapy, as well as an enhancement of the antifungal activity.

**Conclusions:** The overall results suggest that lasertherapy can increase the metabolism of PMN, rendering these cells more competent in their fungicidal activity, as LLLT was also able to modulate the expression of pro-inflammatory and anti-inflammatory cytokines.

## S02.1 Strategies to Change Public Perceptions of Fungal Disease

**Lorna Barnes**

**Objectives:** The MRC Centre for Medical Mycology has created a strategic plan to engage the public with the work of the Centre. An overarching aim of this strategy is to challenge and change public perceptions of fungal disease—from highlighting the scale and impact of fungal pathogens, to understanding the impacts on those who are most at risk from them.

**Materials & Methods:** The Centre will use a range of engagement methods, from the creation of digital resources for young people, to Patient and Public Involvement in research projects, to collaboration with other disciplines, including making interdisciplinary projects with artists. Activities will take place a local, regional, and national level.

**Results**/**Conclusions:** The MRC CMM is at the start of the public engagement delivery plan, and will evaluate its impact over the next five years.

## S02.3 Engaging Public and Politics in Low Middle-Income Countries

**Bright Ocansey**

Globally, little attention is given to medical mycology particularly in low middle-income countries (LMIC). The major underlying factor for this phenomenon is low awareness among relevant stakeholders including the healthcare workers, researchers, policy makers and the general public. Efforts to increase awareness broadly is critical to attract the required attention to improve diagnosis and treatment of serious fungal infections and also encourage research and training in medical mycology.

Although there have been some efforts to improve awareness about medical mycology in some LMIC, the LIFE and GAFFI burden-estimate project has in recent times has particularly stimulated a heightened enthusiasm on the African continent. One of the outcomes is the sprouting network of professional and academic groups or societies to undertake awareness creation including public engagement activities. There are quite a number of such existing societies and groups in LMIC and new ones coming out to champion this course. There are public engagement activities undertaken by these organizations/institutions in few LMIC. Examples; **FIKI Ghana/Ghana Medical Mycology Group:** Fungal Monday/Riddle, fungal infections talk, SMS, aspergillosis lecture, media briefings; **GAFFI:** advocacy works, provide financial support for sensitization programmes in LMICs especially Africa through its ambassadors. Provide free leaflets for distribution to create awareness; **Asian Fungal Working Group**/**ISHAM:** medical mycology training network conferences, educational programmes; **Pan-African Mycology Working Group:** replicate activities of AFWG and push for political will; **Medical Mycology Society of Nigeria:** sensitization programmes, national conference-media briefing, Fungal Disease Awareness Week (FDAW) walk; **Ugandan Association of Medical Mycology/Fungal Interest Group of Uganda:** collaborative research engagements; **Pulmonary Mycoses Centre:** clinical and laboratory mycology training.

Recent avenues for major public engagements are the mucormycosis and COVID-19 in India. This saw airing on major international and local news platforms.

Generally, there is an insufficient political will across most LMIC in improving the status quo regarding diagnosis and treatment of fungal infections as well as research and education in medical mycology. The major contributing factor is the scarcity of local or sub-regional epidemiological data to make a strong case for the buy in of state institutions or agencies that are mostly politically influenced. This makes it extremely difficult to sway stakeholders from a present practice that relatively snubs fungal infections to the integration of fungal infections into health systems. These obstacles can be categorized into “rare vs. common” and “expensive vs. cheap (cost-benefit)”. Some case studies or examples are; Pulmonary TB vs. Chronic fungal lung infections (chronic forms of aspergillosis, histoplasmosis, mucormycosis etc.); TB vs. Histoplasmosis in HIV/AIDS; Candida sepsis vs. bacterial sepsis, target therapy vs. empirical therapy, fungal culture vs. bacterial culture, special stains vs. routine stains.

In conclusion, conscious efforts including trials and pilot programmes with affirmative results are critical to improve the status quo.

## S02.4 Onsite Trainings to Improve Outcomes of People Living with HIV (PLHIV) in Sub-Saharan African Countries

**Aude Sturny Leclère ^1^, Dea Garcia-Hermoso ^1,2^, Alexandre Alanio ^1,4^, Stéphane Bretagne ^1,2,4^, Olivier Lortholary ^1,2,3^ and Angela Loyse ^5^**

^1^ Molecular Mycology Unit, Institut Pasteur
^2^ National Reference Center for Invasive Mycoses, Institut Pasteur
^3^ Necker Pasteur Center for Infectious Diseases and Tropical Medicine, Hôpital Necker Enfants Malades, AP-HP
^4^ Laboratoire de Parasitologie-Mycologie, Hôpital Saint-Louis, groupe Hospitalier Lariboisière, Saint-Louis
^5^ Institute of Infection and Immunity, St. George’s University of London

**Objectives:** Across the world, fungal pathogens are omnipresent in the environment. They affect over a billion people and kill more than 1.5 million people. This figure represents the same incidence than tuberculosis prevalence (1.4 million people died from TB in 2019) and three times more than the incidence for malaria (estimated number of deaths was 409,000 in 2019). Fungal opportunist pathogens occur as a consequence of many conditions, the most important being HIV infection high mortality rates. Globally, in 2019 there were 38 million people living with HIV (PLHIV) with 25.7 million of them located in Africa, highlighting the high numbers exposed to fungal pathogens in Africa. Few fungal diseases are recognized by WHO as neglected pathologies but the few listed represent a minority compared to the diversity of fungi responsible for disease worldwide. Specific issues limit studies in medical mycology in resource limited settings: lack of proper diagnostic procedures; absence of equipment in laboratories; limited clinician experience; poor access to antifungal medicines; lack of surveillance systems, and absence of mandatory reporting. Many of these factors impact the management of PLHIV with fungal disease, including time from presentation to care to diagnosis.

**Materials & Methods:** Our approach has been to organize onsite trainings for frontline healthcare workers (HCWs) alongside didactic mycology courses. Indeed, proper training is mandatory to improve the management of PLHIV with fungal disease and should allow accurate measurement of fungal burden. Only then can the clinical trials implemented in Sub-Saharan African countries be of sustained benefit for PLHIV and frontline HCWs in resource limited settings.

**Results:** We thus implemented two mycology courses in 2017 and 2018 in Senegal and South Africa. Through clinical cases discussions and practical sessions, we reviewed, in under2 weeks, sampling processes, direct exams, rapid diagnostic tests (RDTs), specific cultures and elements of molecular biology. The students were technicians, nurses, clinicians, scientists and students. We also organised 5 trainings focused on *Cryptococcal Meningitis* (and other common causes of HIV-related central nervous system infection) using the “train the trainer” model within the DREAMM (Driving Reduced AIDS Meningo-Encephalitis Mortality) project for frontline HCWs in Cameroon, Malawi and Tanzania which led to improved diagnosis procedures over time. We should soon be able to present data from the DREAMM project, including mortality data pre- and post- implementation of the DREAMM intervention which included these key on-site trainings.

**Conclusions:** Didactic trainings and onsite sessions including medical mycology should be made a priority to improve the diagnosis of fungal pathogens and above all for appropriate management of PLHIV in order to save lives.

## S02.5 Mucormycosis in Covid19 Pandemic: Another Foe Joining Hands with SARS-CoV-2 in India

**Jagdish Chander, Surinder Singhal, Nidhi Singla, Rajpal Punia and Varsha Gupta**

Government Medical College Hospital

**Objective:** Covid19 pandemic has been creating havoc worldwide with over 3 million deaths reported so far all over the world. The impaired lung function creates a state of immuno-compromization among the affected individuals, paving a way for opportunistic infections. Recently, India saw a major epidemic of mucormycosis among SARS-CoV-2 affected patients. India being the diabetic hub of the world with extensive use of steroids for treatment further complicated the situation. We also experienced an outbreak of mucormycosis among patients coming to our institute. Mucormycosis is one of the angioinvasive, rapidly fatal necrotizing fungal infection caused by various species of phylum Glomeromycota. The most common presenting species belong to genera *Rhizopus, Lichtheimia, Apophysomyces, Saksenaea* and *Mucor*. Hereby, we present the epidemiological and demographic analysis of the patients of mucormycosis from a tertiary care institute in North India, during the brief period of two and half months i.e., 1 April 2021 to 15 June 2021.

**Material and Methods:** The present study was conducted on various clinical samples namely biopsy tissue from nasal cavity in rhino-orbital infection, necrotic tissue from cutaneous infection and sputum/BAL in case of pulmonary involvement and were processed in Department of Microbiology as per the standard mycological protocol. Fungal etiology was established by direct KOH/CFW examination and fungal culture was put on Sabouraud’s dextrose agar. The morphological identification of fungal isolates was done by LCB preparation and whenever needed, slide culture was also put up.

**Results:** During the time period, a total of 67 cases were reported from the clinical cases. Maximum cases were of rhino-orbital mucormycosis (63) followed by pulmonary mucormycosis (4). Two patients had mixed infection (both aspergillosis and mucormycosis). Fifty patients gave history of positive COVID infection. Out of 67, 63 patients were diabetic. Male: female was 39:28. Maximum patients were seen in age group 46–60 year old (30) followed by 31–45 year age and more than 60 year age (both 17 each) and 16 to 30 year (3). Most of the patients were not vaccinated. Only 6 were vaccinated with only one having received two doses. Five of the patients had taken only first dose of vaccine. Most of the patients were from surrounding states (50) with 18 from Chandigarh. All patients were positive on direct KOH/CFW wet mount examination for aseptate fungal hyphae. Culture grew *R. arrhizus* (63) followed by *R. homothallicus* (2), *R. microsporus* (1) and *Lichtheimia corymbifera* (1). All patients were treated with liposomal amphotericin B, along with surgical debridement.

**Conclusions:** Co-infections, that too, like mucormycosis can have a great psychological impact on patient as well as the family members. As always, early diagnosis and quick treatment is again the key to treat this disease with severe morbidity and mortality. SARS-CoV-2 infection, treatment with steroids, diabetes mellitus is a triad posing greater risk for mucormycosis.

## S03.1 IFI in Patients Receiving Biologics and Targeted Therapies: Which Drugs and Clinical Situations Really Increases the Risk?

**Jose Aguado**

Currently invasive fungal infection (IFI) is not limited exclusively to patients with neutropenia or receiving corticosteroids or immunosuppressant drugs. Over recent years, the advent of biologic and targeted therapies has introduced new risk groups for IFI. In this presentation, we will overview of the current evidence stating which of these drugs is involved in the development of IFI and what are the preventive or management measures in these patients.

Biologics and targeted therapies associated with increased risk of invasive candidiasis.

Invasive candidiasis is a relatively infrequent complication of the use of biologics and targeted therapies. However, the use of biologics in combination with other immunosuppressants, including corticosteroids, can result in invasive candidiasis in settings where the risk associated with single-agent biologic or targeted therapy is low. An increased risk of mucocutaneous, but not invasive, candidiasis is seen in association with the use of anti-IL-17 agents. It has been previously noted that individuals with functional deficiencies in or antibodies against IL-17 are at risk of developing chronic mucocutaneous candidiasis, suggesting that this pathway plays an important role in the defense against candida infection. Infrequent cases of candidemia and esophageal candidiasis have been reported in association with the use of TNF-*α* inhibitors. The small increased risk of invasive candidiasis in patients taking all these drugs does not warrant anti-*Candida* prophylaxis.

Biologics and targeted therapies associated with increased risk of cryptococcosis.

Cryptococcosis has been reported with the use of biologics including TNF-*α* inhibitors, BTK inhibitors and many others. Patients receiving agents that impair either CD4+ T cells or macrophages are particularly in high risk. The precise risk attributable to each biologic therapy is unclear given that many patients are also receiving cytotoxic chemotherapy or steroids, many are immunosuppressed due to malignancy, and because cryptococcosis also occurs in immunocompetent hosts. No specific prophylaxis against *Cryptococcus* is indicated with any of these agents.

Biologics and targeted therapies associated with increased risk of invasive mould infection.

Invasive mould infection (IMI) is an uncommon complication of biologic and targeted therapies in most settings. Patients with hematological malignancies, particularly those who have received ibrutinib and multiple previous lines of treatment and have relapsed or refractory disease, appear to be at increased risk. This may be at least partially related to immune deficits associated with the underlying condition, as is the case with chronic lymphocytic leukaemia. The concomitant use of other immunosuppressive agents, such as corticosteroids or conventional chemotherapy, in addition to disease- or therapy-related neutropenia, also significantly increases the risk of IMI. Although the risk of IMI associated with ibrutinib therapy is moderate (<10% in most published studies), a high degree of suspicion should be maintained, particularly during the first six months of therapy when IMI risk is highest. Routine anti-mould prophylaxis is not recommended, but prophylaxis can be considered in patients with other risk factors for IMI, such as a history of relapsed or refractory underlying hematological malignancy.

Biologics and targeted therapies associated with increased risk of *Pneumocystis jirovecii* pneumonia.

It is difficult to precisely attribute risk of PJP to many biologics due to coexisting risk factors such as malignancy, transplants or steroid use, the impact of prophylaxis, and the novelty of many agents. This review will discuss the biologics with the strongest evidence of risk for PJP. Idelalisib is a phosphatidylinositol 3-kinase (PI3K) that significantly increases the risk of PJP and prophylaxis is universally recommended, although precise risk estimates are lacking.

In summary, although the overall incidence of IFI in patients receiving biologic or targeted therapies is low, awareness of these potentially serious complications of therapy is essential.

## S03.2 Is Antifungal Prophylaxis Necessary?

**Malgorzata Mikulska**

Antifungal prophylaxis is a well-known strategy to prevent fungal diseases (IFD) in populations which are at high risk for IFD. In order to establish the utility of antifungal prophylaxis, several issues need to be addressed.

First, the rate of IFD in a given population, established both as number of patients among those at risk and as a rate, i.e., a number of IFD for, typically, 1000-patient at-risk day. This is because in case of a very prolong at-risk period, the evaluation of cost-effectiveness changes significantly compared to a short period of at-risk time. Moreover, the increased risk of IFD usually does not depend exclusively on the administered treatment with biologics or targeted therapies, but is also influenced by the underlying disease, past or concomitant treatments and past or current immune deficits. Genetic predisposition plays also its role, although its evaluation in not feasible in most settings. Second, the clinical impact, in terms of morbidity and mortality, of the IFD or IFDs most frequently diagnosed in specific population. If the mortality and morbidity are not increased when IFD is promptly diagnosed and treated, and if such a prompt and reliable diagnosis and treatment are widely available, the early diagnostic strategy, either symptom-triggered or as screening and pre-emptive therapy, might be preferred. Third, the availability of active and feasible prophylactic options among the antifungals. The feasibility of prophylaxis will depend also on the length of at-risk period and the setting in which the patient is treated, since non-oral antifungals can usually be used only in some settings—either in hospital-admitted patients or in those with frequent clinical visits in whom intermittent administration can be proposed. Fourth, the toxicity of antifungal prophylaxis should be considered. The number of patients needed to harm (NNH, the number of patients experiencing side effects of prophylactic treatment), can provide a useful tool to evaluated the overall benefit when confronted with the number need to treat (NNT) to prevent one IFD. This evaluation will be also influenced by the length of at-risk period. In addition to direct toxicity, the impact of drug interactions, particularly with targeted therapies, should be carefully considered for the main class of antifungals used as prophylaxis, such as azoles. Finally, the clinical data form randomized and observational trials on the documented clinical benefit of prophylaxis are fundamental. They reflect the final benefit, which is influenced also by the real-life feasibility, tolerability and, in case of observational trials, also availability of the treatment.

So far, few biologics and certain targeted therapies, mainly administered in a well-known settings at high risk for IFDs such as acute myeloid leukemia or GvHD, have been associated with an increased risk of IFD. As azoles are currently the only oral option for antifungal prophylaxis, the problem of drug interactions with targeted agents is particularly challenging. Currently, there are not data from large randomized trials that support the use of antifungal prophylaxis specifically in patients treated with biologics or targeted agents. Therefore, careful clinical evaluation based on the aforementioned issues and expert advice remain the bases for difficult daily clinical decisions.

## S03.3 Biologic and Targeted Therapies for the Treatment of IFI

**Elham Khatamzas**

**Objectives:** Adjunctive modulation of host immune responses has recently become an increasingly attractive therapeutic strategy for the high number of challenging invasive fungal infections. Single case reports have provided promising first results where restoration of host immunity has led to control of infection. Animal and pre-clinical studies have provided insight into the complex fungi-host interaction and immunology.

Therapies under evaluation include cytokines, immune cell transfusions of innate and engineered T cells, monoclonal antibodies as well as checkpoint inhibitory agents. These need to be further studied in future clinical trials of defined patient populations with complex IFIs in order to expand our therapeutic armamentarium.

## S03.4 Immune Dysregulation in Psoriasis Patients with Skin or Mucosal Candidiasis during IL-17 or IL-(12)/23 Inhibitor Therapy

Mariolina Bruno ^1,^*, Linda Davidson^1,^*, Hans Koenen ^2^, Juul van den Reek ^3^, Bram van Cranenbroek ^2^, Elke de Jong ^3^, Frank van de Veerdonk ^1^, Bart-Jan Kullberg ^1^ and Mihai Netea ^1^

^1^ Radboud University Medical Center Center for Infectious Diseases (RCI), Department of Internal Medicine, Radboud University Medical Center, Nijmegen, the Netherlands
^2^ Laboratory for Medical Immunology, Radboud University Medical Center, The Netherlands
^3^ Department of Dermatology, Radboud University Medical Center, Nijmegen, The Netherlands

**Introduction:** Psoriasis is a common chronic inflammatory skin disease. Moderate-to-severe forms are often treated with targeted biologics. Biologics that block the T-helper (Th)17 pathway can be very effective, but the same pathway is known to be crucial for antifungal host defense. Clinical data suggest an increase in candidiasis incidence during interleukin (IL)-17 inhibitor therapy, but a comprehensive description of the effects of this biological therapy on anti-fungal host defense is missing.

**Objectives:** To investigate the innate and adaptive host immune response in psoriasis patients who developed candidiasis during treatment with biologics targeting the Th17 pathway: IL-17, IL-23 and IL-12/23 inhibitors.

**Methods & Materials:** Anti-fungal immune responses were studied in a case-series of 22 psoriasis patients with a history of skin and/or mucosal candidiasis during treatment with IL-17 or IL-(12)/23 inhibitors. Patients have been stratified according to biologic type in either IL-17 inhibitors (secukinumab, ixekizumab, brodalumab) or IL-(12)/23 inhibitors (ustekinumab, guselkumab). Healthy subjects served as a reference control group. Immunophenotype was characterized via whole blood flow cytometry. Immunological tests were performed in primary immune cells isolated from peripheral blood to characterize potential immune defects. The main parameters compared between patients and healthy controls were the cell counts of immune cell populations and cytokine production profiles. Regarding the latter, both innate and adaptive host immune response in terms of cytokine production upon different in vitro stimulations were investigated.

**Results:** Psoriasis patients with IL-17 as well as with IL-(12)/23 inhibitor therapy showed lower NK cell counts and higher B cell counts than controls, but a specific significantly lower count of CD4+ Th1 and Th1Th17 was only found in patients treated with IL-17 inhibitors as compared to controls. As compared to healthy controls, peripheral blood mononuclear cells (PBMC) from these patients stimulated with *Candida (C.) albicans* conidia showed significantly lower IL-6 and IL-1β production, while the release of TNF and reactive oxygen species was similar between patients and controls. The production of IFN-γ in response to *C. albicans* hyphae after 7 days was significantly decreased in patients receiving therapy, as well as IL-10 production in response to various stimuli. Finally, in response to stimulation with the polarizing cytokines IL-1β and IL-23, the Th17 cytokine response was significantly lower in patients, as compared to controls.

**Conclusions:** In this exploratory immunological study, psoriasis patients who developed skin and/or mucosal candidiasis during treatment with IL-17 or IL-(12)/23 inhibitors showed both innate and adaptive immune response defects. Compared to healthy controls, lymphocyte counts were altered and IL-6, IL-1β and IFN-γ production was decreased in response to *C. albicans*. These defects might contribute to susceptibility to candidiasis in psoriasis patients treated with biologics targeting the Th17 pathway.

## S04.2 Opelconazole (PC945): A Novel Inhaled Azole in Late-Stage Clinical Development for Invasive Pulmonary Aspergillosis

**Lance Berman**

The incidence of fungal infections and diseases has increased substantially over the past two decades and invasive forms are a leading cause of morbidity and mortality, especially among immune-suppressed patients. Aspergillosis results in a range of disorders either directly from the infection, or by triggering an allergic response, with the respiratory system most commonly affected.

Invasive aspergillosis (IA) occurs in 4% of patients undergoing remission induction chemotherapy for hematological malignancies, in 9% of allogeneic hematological stem cell transplant recipients [Van de Peppel 2018] and in 2–8% of lung transplant recipients [Samanta 2020, Baker 2020, Ullmann 2018], despite the use of antifungal prophylaxis in these patient groups. *Aspergillus* is a particularly important opportunistic infection in lung transplant recipients [Pasupneti 2017, Husain 2019] with invasive disease occurring in 2–8% of patients in the first-year post-transplant [Samanta 2020, Baker 2020, Ullmann 2018].

Currently available antifungal therapies have important limitations such as route of administration or dosing forms, treatment-limiting side effects and drug-drug interactions [Ullmann 2018, Husain 2016]. These pose a significant challenge in patients with an underlying malignancy or in recipients of a solid organ transplant. In lung transplant recipients the anastomotic site is particularly vulnerable to aspergillus infection due to disruption of blood supply and the presence of sutures. Even when treatment for IA includes the most recently approved systemic antifungals, success rates remain low (≤50%) and overall mortality remains high (up to 40%).

There are therefore limited therapeutic options for patients with invasive pulmonary aspergillus disease particularly for those who do not respond to initial therapy or in whom antifungals that are approved for the treatment of invasive aspergillosis are contraindicated.

A potent, effective inhaled anti-fungal agent with prolonged lung tissue residence and minimal systemic update would be a valuable adjunct to current therapeutic options. To date, treatment using inhaled antifungal agents has been limited to repurposing available systemic medicines. Opelconazole is a novel triazole antifungal agent which was specifically designed for inhaled use and which is being developed as an inhaled treatment for pulmonary fungal disease. The profile of opelconazole has been assessed in a range of in vitro and in vivo studies demonstrating that it has potent antifungal activity against A. fumigatus, C. albicans and a range of other fungi [Colley T et al., 2017; Colley et al., 2019]. Following inhaled delivery, local concentrations of opelconazole in the lung are high, while systemic bioavailability is minimal. This profile should allow opelconazole to provide effective antifungal activity in the respiratory tract while limiting the potential for systemic side effects.

Opelconazole has been supplied to patients with serious or life-threatening pulmonary aspergillosis who have not responded to available mold-active therapies under a Special Needs program regulated in the United Kingdom by the Medicines and Healthcare products Regulatory Agency. Successful outcomes in the first two patients who developed invasive pulmonary aspergillosis due to infections with *A. fumigatus* complex shortly after lung transplantation have been reported [Pagani 2020]. Available data from the program indicate that opelconazole was generally well tolerated with no drug-drug interactions reported in the 11 patients who received opelconazole as treatment and in the 12th patient who received it as prophylaxis. Favorable responses at 3 months were observed in 9 out of the 11 patients who received opelconazole as treatment.

## S04.4 NP339—A Novel Peptide Antifungal

**Deborah O’Neil**

NP339 is a broad-spectrum, membrane-acting, novel peptide antifungal. It is rapidly fungicidal, agnostic to target pathogen drug resistance status and furthermore, possesses a mechanism of action which mitigates the development of drug resistance in target pathogens. NP339’s exquisite specificity to fungal cell membranes means that it is not cytotoxicity against eukaryotic host cells. These, and a number of other differentiating characteristics of NP339 render it a promising, novel antifungal candidate. NP339 is currently in development by NovaBiotics as both an intravenous therapy for disseminated fungal disease and in inhaled form for respiratory fungal infection.

## S05.1 Global Epidemiology of Dermatophytosis: What Is Changing, What Is Remarkable?

**Rudramurthy Shivaprakash**

Dermatomycoses are among the most frequent forms of human infections, affecting more than 20–25% of the world’s population. *Trichophyton* species are the primary causative agents, with 70–90% prevalence for onychomycosis cases and 53.1–86% in tinea infection. *Trichophyton rubrum* is the key etiological agent, followed by *T. mentagrophytes, Microsporum canis, M. gypseum*. In western countries, *T. rubrum* and *M. canis* act as a significant cause of dermatophytosis. In contrast, southern and East European countries show the prevalence of *M. canis* and *T. mentagrophytes*. Previously, *T. rubrum* (anthropophilic) was the most common etiology agent responsible for dermatophytosis in India. But nowadays, the *T. mentagrophytes* complex is the primary etiology accountable for the ongoing epidemic. Presently, epidemiology has considerably changed due to increasing recalcitrant/relapse/recurrent and chronic patients due to widespread and atypical clinical presentation of lesions, treatment failure, and resistance to the antifungals (class-azoles, and allylamines), especially in India and neighbouring countries. Clinical strains of *T. rubrum* and *T. interdigitale* documented only 1% terbinafine resistance in Switzerland with point mutation at Leu393, Phe397, Phe415, and His440in squalene epoxidase gene. The present prevalence of *T. rubrum* resistance to terbinafine in Europe contrasts with that of resistant *T. mentagrophytes* isolates in India (30–70%). Phe397 is the most common mutation reported for terbinafine resistance in dermatophytes. The primary factor for contributing resistance in India is irrational or over-the-counter usage of antifungals agents without proper prescription. Although steroids initially improve the itching and redness caused due to dermatophytic infection, they did not wholly eradicate the fungi at the lesion site and are considered as a cause of atypical presentation. Several studies reported steroid-modified tinea cases due to frequent over-the-counter drugs (FDC cream) without prescription ranging from 42% to 81.3%. Increase in the incidence of terbinafine resistance in certain regions has compelled the performance of antifungal susceptibility testing for successful therapy. But the clinical breakpoints and epidemiologic cut-off values are not currently available for dermatophytes to help with the interpretation of these results. Clinical trials and pharmacokinetic and pharmacodynamics studies are urgently needed to address the issues of recurrence or clinical failures while treating dermatophytosis.

## S05.2 How to Determine MICs for My Dermatophyte Strain: Protocoles, Tips, and Trics

**Sevtap Arikan Akdagli**

Dermatophytes are pathogenic fungi that have high affinity for keratinized tissue and cause infections in hair, skin and nails. Dermatophytosis is a worldwide public health problem. Further, deep dermatophytosis may be observed in immunosuppressed individuals and in patients with primary immunodeficiencies. In addition to considerable clinical significance of dermatophytosis in terms of global incidence, antifungal resistance has recently drawn attention as an emerging problem in some dermatophyte strains. Resistance to terbinafine and/or azoles have so far been reported for isolates belonging to species of *Trichophyton* (*T. rubrum, T. interdigitale. T. mentagrophytes*, *T. indotineae* sp. nov. related to the *T. mentagrophytes/interdigitale* complex) and *Microsporum* (*M. canis*). The observation of emerging antifungal resistance has further augmented the necessity for the utility of in vitro antifungal susceptibility tests for clinical guidance in recalcitrant cases of dermatophytosis, in particular. Reference CLSI and EUCAST microdilution methodologies are available for testing antifungal drugs against dermatophytes. Following a multicenter project that was initiated in 2003, a modified CLSI M38-A reference method for testing dermatophytes was included in CLSI M38-A2 document that was published in 2008. This was recently followed by documentation of EUCAST reference method E.Def 11.0 in 2021 for microconidia forming dermatophytes. Moreover, EUCAST tentative epidemiological cut-off values were determined for itraconazole, terbinafine, and voriconazole against *T. interdigitale* and *T. rubrum*. While both CLSI and EUCAST reference methods follow microdilution test principles, differences in the test parameters do exist as summarized in the Table.

Given the fact that the ability of dermatophytes in forming biofilms may also contribute to antifungal resistance, in vitro and ex vivo (nail fragments, hair strands) models have also been studied for exploring the antibiofilm activities of antifungal drugs for dermatophytes. Ex vivo biofilm models provide a novel and exciting approach for assessment of activity of antifungal drugs against dermatophyte biofilms. Wide variability of experimental parameters used in in vitro and ex vivo biofilm models suggest the need for methodological standardization.

Utility of reference methods for antifungal susceptibility testing of dermatophytes will aid in further clarification of the extent of antifungal resistance and guidance of treatment particularly in refractory cases of dermatophytosis.


**Test Parameter **

**CLSI M38**

**EUCAST E.Def 11.0**
Glucose concentration in susceptibility testing medium (RPMI 1640 with L-glutamine and without sodium bicarbonate, pH = 7) 0.2%2%Cycloheximide and chloramphenicol supplementation of the inoculum #x2212;+Inoculum type and concentrationConidia 1−3 × 10^3^ cfu/mL
Microconidia 1−2.5 × 10^5^ cfu/mL
Microdilution plates96-well U-shaped96-well flat-bottomIncubation period and temperature for the microdilution plates4 days at 35 °C5 days at 25−28 °CMIC reading methodVisualSpectrophotometricMIC reading endpointMIC-1 (80% inhibition) (Terbinafine, azoles, ciclopirox olamine, and griseofulvin)MIC-2 (50% optic density reduction) (Terbinafine, azoles, and amorolfine)

## S05.4 Malassezia: Epidemiology and Host-Response Studies

**Dora Edith Corzo Leon**

**Abstract:** The genus *Malassezia*, is a group of lipophilic yeasts, and part of the normal human mycobiota. They colonise several regions of the body, mainly sebum-rich skin areas such as the scalp and thorax. This group of fungi can cause a variety of infections (pityriasis versicolour, folliculitis and fungaemia) and has been associated with non-infective inflammatory diseases such as seborrheic dermatitis and atopic eczema. A global description of clinical and epidemiological characteristics of infections caused by *Malassezia* species in the past 12 years will be done in this talk.

Most *Malassezia* species are unable to synthesise fatty acids and degrade carbohydrates. Hence the acquisition of lipids and carbohydrates is dependent upon the acquisition of exogenous fatty acids via a large repertoire of lipolytic enzymes. Lipases hydrolyse sebum triglycerides from the host skin to release fatty acids. Oleic acid (OA) and arachidonic acid are the fatty acids released by phospholipase and lipase activity and have an irritating/inflammatory effect on skin facilitating *Malassezia sympodialis* to directly interact with skin cells. The exogenously acquired fatty acids also contribute to the formation of a thick cell wall in *Malassezia* sp. characterised by a unique lipid-rich outer layer, which contributes to triggering the immune response against this group of fungi. Human antimicrobial peptides specifically β-defensins, RNase7, and S100A7 are key in the skin’s protective mechanisms. A description of host response to *Malassezia sympodialis* using two different skin environments (oily and non-oily) on an ex-vivo human skin model will be done and it will be compared to other models and clinical findings. Host:pathogen interactions analysed by SEM, histology, gene expression, immunoassays and proteomics will be showed.

Atopic eczema (AE) is a chronic inflammatory disease affecting up to 20% of children and 3% of adults. Multiple factors have been associated with the origin of AE such as impairment of skin barrier function, genetic, and environmental factors. The mechanisms linking *Malassezia* sp. to AE pathogenesis is the observation that allergens produced by *Malassezia* sp. induce specific IgE antibodies and autoreactive T-cells that can cross-react with skin cells. *M. sympodialis* encodes 13 allergens, some of these allergens are highly similar to human proteins and have been specifically linked to cross-reactive immune responses (Mala S 11 and Mala S 13). These allergens (specifically Mala S 1 and Mala S 7) are carried inside extracellular nanovesicles, which are produced by an endosomal mechanism and are thought to be released into the skin environment to elicit specific immune responses in AE individuals. New preliminary evidence on cross-reactivity of Mala S 1 allergen with skin will be showed.

## S05.5 Evaluation of the Multiplex Real-Time PCR DermaGenius® Assay for the Detection of Dermatophytes in Hair Samples from Senegal

**Mouhamadou Ndiaye ^1,2^, Rosalie Sacheli ^3,4^, Khadim Diongue ^1,2^, Caroline Adjetey ^3^, Rajae Darfouf ^3,4^, Mame Cheikh Seck ^1,2^, Aida Sadikh Badiane ^1,2^, Mamadou Alpha Diallo Diallo ^2^, Thérése Dieng ^1^, Marie-Pierre Hayette ^3,4^ and Daouda Ndiaye ^1,2^**

^1^ University Cheikh Anta Diop
^2^ Laboratory of Parasitology, University Hospital of Aristide Le Dantec
^3^ Department of Clinical Microbiology, Center for Interdisciplinary Research on Medicines (CIRM), University Hospital of Liege
^4^ National Reference Center for Mycosis, University Hospital of Liege

**Objectives:** For the successful treatment of dermatophytoses, there is a need for accurate and rapid diagnostic methods. A lot of recent literature has focused on the detection of dermatophytes directly on sample material such as nails, hair and skin scrapings. Molecular tools offer the ability to rapidly diagnose dermatophytosis within 48 h. This study aimed to compare the results of a commercial real-time PCR (real-time PCR) assay DermaGenius^®^(DG) complete version with those of conventional diagnostic methods (direct microscopy and culture).

**Materials & Methods:** A total of 129 hair samples were collected in Dakar (Senegal) from patients suspected of dermatophytosis. Hair samples were tested prospectively using microscopy, culture and PCR. Samples were prepared for microscopy using 20% potassium hydroxide and lactophenol cotton blue and cultured using Glucose peptone agar plate supplemented with chloramphenicol and Cycloheximide and Glucose peptone agar plate supplemented with chloramphenicol.The DG assay was performed according to manufacturer’s instructions. The DNA extracts were analysed using the Rotor-Gene® (QIAGEN®) real-time PCR cycler. Dermatophytes species and were differentiated by melting curve analysis. The agreement between different laboratory methods in measuring the same variable was estimated by Cohen’s kappa test (K). Sensitivity, specificity, positive predictive value (PPV), negative predictive value (NPV), accuracy of different laboratory methods was determined.

**Results:** Out of the 129 cases, 30 (23.26%) were male and 99 (76.74%) female. Patient’s age varied from 1 to 80 years along with a mean age at 23.64 ± 17.61 years. Of the 129 patients clinically suspected of TC, 46.41% (56/129) were positive and 56.59% were negative (73/129) in culture. Dermatophytes were detected by DG PCR in 52.71% (68/129). This study shows that DG PCR has 89.3% sensitivity, 75.3% specificity, 81.4% accuracy, 73.3% positive predictive value (PPV) and 90.2% negative predictive value (NPV). The DG PCR assay was more sensitive than culture for dermatophytes detection in patients (*p* < 0.05). The kappa coefficient in case of discrepancies between the two methods was good (k = 0.62). The dermatophytes isolated by culture were *T. soudanense* in 35 (27.13%) cases and 51 (39.53%) by DG PCR, followed by *M. audouinii* in 18 (13.95%) in DG PCR vs. 17(13.18%) cases in culture.Two mixed infections *T. soudanense/M. audouinii* (5/129 cases) and *T. soudanense/M. canis* (1/129 cases) were only detected in DG PCR.

**Conclusions:** DG PCR showed excellent performance characteristics for the detection of dermatophytes and is significantly faster than cultures techniques, which makes it very promising for routine diagnostics of dermatophytosis in Africa particularly in Senegal. It can help the clinician in initiating prompt and appropriate antifungal therapy. This technique is not only rapid but also simple and cheap in comparison to other molecular methodes for the detection of dermatophytes.

## S06.1 How do Antibiotics Promote Susceptibility to Healthcare-Associated Fungal Infections?

**Rebecca Drummond**

Antibiotics are recognized to modulate the immune system, potentially leading to abrogated antimicrobial immunity and/or altered host homeostasis. This occurs either through direct antibiotic effects on immune cell function, and/or through indirect systemic effects that are dysbiosis-driven. Antibiotic pre-exposure is a modifiable iatrogenic risk factor for the most common human nosocomial fungal infection, invasive candidiasis. Yet, the mechanisms underlying antibiotic-induced susceptibility to invasive fungal disease remain elusive. In this talk, I will present our data where we show that antibiotic pre-exposure, particularly with vancomycin, enhances susceptibility to invasive candidiasis in mice. The susceptibility maps to impaired lymphocyte-dependent IL-17A- and GM-CSF-mediated antifungal immunity within the gastrointestinal tract, which leads to non-inflammatory escape of bacteria and systemic bacterial co-infection and can be ameliorated by IL-17A or GM-CSF immunotherapy. Antibiotic pre-exposure was associated with increased risk of invasive candidiasis and subsequent death after invasive candidiasis in a large health record patient database. Our work highlights the importance of antibiotic stewardship in protecting vulnerable patients from life-threatening infections and provides mechanistic and potential therapeutic insights into a controllable iatrogenic risk factor for invasive candidiasis.

## S06.2 Human Genetic Determinism of Fungal Diseases

**Anne Puel**

It has been estimated that there are at least 1.5 million fungal species, mostly present in the environment, but only a few of these fungi cause human disease. Most fungal diseases are self-healing and benign, but some are chronic or life-threatening. Acquired and inherited defects of immunity, including breaches of mucocutaneous barriers and circulating leukocyte deficiencies, account for most severe modern-day mycoses. Other types of infection typically accompany these fungal infections. More rarely, severe fungal diseases can strike otherwise healthy individuals. Historical reports of fungi causing chronic peripheral infections (e.g., affecting the nails, skin, hair), and invasive diseases (e.g., brain, lungs, liver), in otherwise healthy patients, can be traced back to the mid-20th century. These fungi typically cause endemic, but not epidemic diseases, are more likely to underlie sporadic than familial cases, and only threaten a small proportion of infected individuals. The basis of this ‘idiosyncratic’ susceptibility has long remained unexplained, but it has recently become apparent that ‘idiopathic’ fungal diseases, in children, teenagers, and even adults, may be caused by single-gene inborn errors of immunity (IEIs). The study of these unusual IEIs has led to the identification of molecules and cells playing a crucial role in human host defenses against certain fungi at particular anatomic sites. A picture is emerging of inborn errors of IL-17 immunity selectively underlying chronic mucocutaneous candidiasis, with little inter-individual variability, and of inborn errors of CARD9 immunity underlying various life-threatening invasive fungal diseases, differing between patients.

## S06.4 Serum Proteins, Crucial Factors for Human Physiology that Are Exploited by Candida Species to Promote Pathogenicity

**Sophie Austermeier ^2^, Sophia Hitzler ^1^, Marina Pekmezovic ^2^, Ann-Kristin Kaune ^2^, Bernhard Hube ^2^ and Mark Gresnigt ^1^**

^1^ Junior Research Group Adaptive Pathogenicity Strategies, Leibniz Institute For Natural Product Research And Infection Biology—Hans Knöll Institute
^2^ Department of Microbial Pathogenicity Mechanisms, Leibniz Institute For Natural Product Research And Infection Biology—Hans Knöll Institute

**Objectives:** Serum proteins play vital roles in human physiology and coping with microbial infections. Nevertheless, opportunistic pathogens evolved elegant strategies to deal with environmental changes and threats imposed by the host, contributing to their success in causing infections. Yeasts of the genus *Candida* that colonize a variety of mucosal surfaces are no exception. Their constant interaction with the host in commensal niches may have driven co-evolution with the host and the capacity to exploit host proteins. We are studying the adaptive pathogenicity strategies of *Candida* species. Specifically our objective is to discover mechanisms by which these fungi exploit serum proteins.

**Materials & Methods:** To investigate how serum proteins play a role during the pathogenesis of *Candida* infections, we introduced these proteins in in vitro infection models to study *Candida* pathogenicity mechanisms during interactions with epithelium, organ tissue, and cells of the innate immune system.

**Results:** Albumin is the most abundant protein in human blood, but is also present in mucosal tissues. The high affinity of albumin to hydrophobic molecules, makes it a trap for hydrophobic microbial toxins, including the toxin candidalysin that is essential for full *C. albicans* virulence. Yet, we also observed that *Candida* species can exploit albumin, which contributes to their capacity to cause infection and escape neutrophil-mediated killing.

1-antitrypsin (AAT) is an acute-phase protein controlling inflammatory responses and preventing immunopathology. However, *C. albicans* increases filamentation in the presence of AAT. At physiological serum concentrations, this contributes to fungal escape from monocytes and macrophages.

**Conclusions:** While serum proteins are key players in human physiology, they are often disregarded in infection biology. Nevertheless, evidence is amassing that these proteins play vital roles in the pathophysiology of opportunistic fungal infections. Understanding these complex interactions provides key insights in the pathogenesis of candidiasis that could be exploited for novel diagnostic and therapeutic strategies.

## S06.5 Increased Efficacy of IL-18/Posaconazole Combination in a Neutropenic Animal Model of Pulmonary Invasive Aspergillosis by an Azole Resistant *A. fumigatus* Isolate

**Panagiota-Christina Georgiou ^1^, Maria-Ioanna Beredaki ^1^, Anastasia Tsiavou ^1^, Spyros Pournaras ^1^, Panagiotis Tsirigotis ^2^ and Joseph Meletiadis ^1,3^**

^1^ National and Kapodistrian University of Athens, Medical School
^2^ Hematology Unit of B-Educational Pathology Clinic, Attikon University Hospital
^3^ Department of Medical Microbiology and Infectious Diseases, Erasmus Medical Centre

**Objectives:** *Aspergillus fumigatus* is an ubiquitous airborne saprophytic fungus that can cause invasive pulmonary aspergillosis, a life-threatening infection for immunocompromised patients. Posaconazole, an extended-spectrum triazole, is extensively used for prophylaxis of aspergillus diseases in neutropenic patients with acute myeloid leukemia. Given the emergence of azole resistance in *A. fumigatus* new prophylactic regimens are required. Triggering host defense against *A. fumigatus* with cytokines is a new approach that has not been fully studied. We have previously shown that IL-18 increased survival of mice infected with azole-susceptible *A. fumigatus* isolate (Georgiou et al, ECCMID2021). In the present study, we investigated the efficacy of IL-18 alone and in combination with posaconazole in an animal neutropenic model of invasive pulmonary aspergillosis by an azole-resistant *A. fumigatus* isolate.

**Materials & Methods:** Six weeks old female CD-1 mice rendered neutropenic with cyclophoshamide (150 mg/kg i.p. −4, −1, 4 and 8 days of infection) were infected intranasally with 6 × 10^6^ CFU/mL of *A. fumigatus* (resistant to posaconazole strain v79-63 with EUCAST MIC >16 mg/L) conidia in PBS/Tween 20 under light anesthesia (isoflurane). Mouse cytokine IL-18 at the optimal concentration 10 ng/mouse based on previous studies (Georgiou et al, ECCMID2021) was given daily intranasally (50 μL/dose) to groups of 10 animals at −2 d till + 7 d, alone or in combination with posaconazole 16 mg/kg/day. Placebo group was treated with saline. Animals were also treated with ceftazidime 50 mg/kg/day as antibacterial prophylaxis. Survival was recorded until 15 d. Survival curves were compared using Log-rank (Mantel-Cox) test. The effect of IL-18 and posaconazole monotherapy and combination therapy on fungal burden in lungs was assessed in 3 animals treated for 3 d when animals were euthanized, lungs were excised and homogenized and CFUs were determined with quantitative cultures. Differences in CFU were assessed with one-way ANOVA followed by Dunnett’s test.

**Results:** The survival rates in each group were 20% in control group, 56% in IL-18 group, 56% in posaconazole group and 80% for combination group. Log-rank (Mantel-Cox) test showed statistically significant differences (*p* = 0.044) with a significantly higher survival rate found with the combination therapy but not with monotherapy regimens compared to placebo. The mean ± SEM log_10_CFU/lung were 4.23 ± 0.53 in combination group, 4.82 ± 0.44 in IL-18 group, 5.2 ± 0.48 in posaconazole group and 6.1 ± 0.08 in placebo which a significantly difference found between the combination and placebo group (*p* < 0.05).

**Conclusions:** A significant antifungal effect was found for the combination of IL-18 with posaconazole in prolonging survival and reducing fungal burden in lungs of neutropenic animal model. Further studies are required to explore the role of IL-18 against invasive pulmonary aspergillosis in neutropenic patients.

## S07.2 Antifungal Prophylaxis: Who, with What and How Long?

**Shmuel Shoham**

**Purpose of presentation:** This presentation reviews the role of antifungal prophylaxis in solid organ transplant recipients with focus on utilizing epidemiology and diagnostic assays to assist in selection of the right drug, for the right patient at the right time.

**Overview:** Invasive candidiasis and aspergillosis are the two most important fungal infections in the early post-transplant period. Prophylaxis to prevent those infections is routinely used in liver, pancreas, lung and small intestine transplant recipients. It is also used to prevent coccidioidomycosis in all recipients living in areas endemic for that infection, regardless of organ type. Prophylaxis efforts focus on balancing the risks of infections with the risks of antifungal therapy (e.g., drug toxicity, drug interactions, development of fungal resistance and cost). There are three broad prophylaxis strategies: (1) Universal prophylaxis, in which all recipients of an organ receive an antifungal, (2) Targeted antifungal prophylaxis, in which clinical and laboratory characteristics are used to identify patients for prophylaxis and (3) Preemptive prophylaxis/therapy, in which microbiologic assays are used to initiate antifungals at the earliest stages of infection.

Organ specific approaches:

**Liver transplant:** The two main approaches are universal prophylaxis with an anticandidal agent (usually fluconazole) and targeted prophylaxis based on risk factors for invasive candidiasis and/or aspergillosis. Risk factors for candidiasis include: renal failure requiring dialysis, *Candida species* colonization, MELD score ≥30, re-transplantation, living donor, transfusion of ≥40 units of cellular blood products during transplant, choledochojejunostomy, biliary leaks and need for re-operation. Such patients generally receive 14–28 days of anticandidal prophylaxis (e.g., fluconazole unless also at risk for aspergillosis or high prevalence of fluconazole resistance). Risk factors for invasive aspergillosis include fulminant hepatic failure, renal failure requiring dialysis, higher doses of steroids within a month of transplant, re-transplant, multi-visceral transplantation, biopsy-proven rejection requiring T cell depleting therapy and re-operation. Such patients generally receive 14–21 days of mold active antifungal therapy (e.g., echinocandin or lipid formulation amphotericin B). The role of prophylaxis with mold active azoles is evolving in liver transplant recipients.

**Pancreas transplant:** The two main approaches are universal prophylaxis with an anticandidal agent and targeted prophylaxis based on risk factors for invasive candidiasis. Risks for candidiasis include enteric drainage, vascular thrombosis, and post-perfusion pancreatitis. Patients typically receive 4 weeks of therapy (with fluconazole unless high prevalence of resistance).

**Lung transplant:** The predominant pathogens of concern are filamentous fungi, particularly *Aspergillus species*. Of note, the risk for invasive candidiasis can be significant in the first 30 days. The three main approaches are universal prophylaxis with a mold active antifungal agent (e.g., mold active azole or lipid formulation of amphotericin B), targeted prophylaxis based on risk factors for invasive aspergillosis and a preemptive approach based on protocolized bronchoalveolar lavage studies (culture and galactomannan) done at routine intervals. Risk factors taken into consideration for targeted antifungal prophylaxis include presence of *Aspergillus species* in airway cultures (especially in those with bronchiectasis), single-lung transplant, airway ischemia, CMV, use of a T cell depleting agent and acquired hypogammaglobulinemia. Prophylaxis is generally for 4–6 months. When a preemptive strategy is used prophylaxis/treatment is usually for 3–4 months.

**Other situations:** For recipients of small bowel transplant, anticandidal prophylaxis is used for 4 weeks or until the anastomosis has healed and rejection is not present. For any transplant recipient living in areas endemic for coccidioidomycosis an azole is typically used for the first 6–12 months after transplant, although some continue for life in lung transplant recipients.

## S07.4 Side-Effects and Completion Rates of Universal vs. Targeted Antifungal Prophylaxis among Lung Transplant Recipients 2010–2019

**Cornelia Geisler Crone ^1^, Signe Marie Wullf ^1^, Jannik Helweg-Larsen ^2^, Pia Bredahl ^4^, Maiken Cavling Arendrup ^5,6,7^, Michael Perch ^3,7^, Marie Helleberg ^1,2^**

^1^ Centre of Excellence for Health, Immunity and Infections (CHIP), Rigshospitalet
^2^ Department of Infectious Diseases, Rigshospitalet
^3^ Department of Cardiology, Section for lung transplantation
^4^ Department of Thoracic Aneasthesiology and Intensive Care, Rigshospitalet
^5^ Unit of Mycology, Statens Serum Institut
^6^ Department of Clinical Microbiology, Rigshospitalet
^7^ Department of Clinical Medicine, University of Copenhagen

**Objectives:** In lung transplant recipients, it is unclear if invasive fungal infections (IFI) are best prevented by universal or targeted antifungal prophylaxis. The Danish guideline for prevention of IFI in these patients was changed from universal to targeted antifungal prophylaxis in 2016. We examined side-effects and rates of completion of antifungal prophylaxis before and after this change.

**Materials & Methods:** Retrospective study of all adult lung transplant recipients at Copenhagen University Hospital during 2010 to 2019. Standard IFI prophylaxis 12 weeks after transplantation was voriconazole for all patients in 2010–2016. In 2017–2019 targeted prophylaxis with posaconazole and inhaled amphotericin B was used for high risk patients only. Failure to complete prophylaxis was defined as recieving <9 of the intented 12 weeks of prophylaxis. Liver and kidney toxicity was graded according to Common Terminology Criteria for Adverse Events (CTCAE) criteria (grades 0–4 according to degree of elevation). Grade ≥2 adverse events were classified as high. Lung biopsies were evaluated for acute rejection (grade ≥A2). Calcineurin-inhibitor (CNI) plasma-concentrations were evaluated for off-target episodes. High and low episodes were defined as ≥2 concecutive samples 20% above or below target range limits, respectively. Low CNI episodes were considered related to discontinuation of voriconazole if occurring 0–14 days from discontinuation. Acute rejections were classified as related to low CNI if they occurred 0–30 days from low CNI. Grade ≥2 kidney toxicity was considered related to high CNI if it occurred between two days prior to high CNI to 7 days after.

**Results:** We included 295 patients. Universal and targeted antifungal prophylaxis was used in 183 of 193 (94.8%) recipients during 2010–2016 and in 6 of 102 (5.6%) recipients during 2017–2019 (Table). The underlying diseases were comparable in the two periods, with emphysema as the main disease (46%). The rates of single lung transplantation were 13% in the universal vs. 5% in the targeted prophylaxis period (*p* = 0.048). Among the 183 patients receiving voriconazole prophylaxis, 114 (62%) failed to complete prophylaxis, 82 (72%) of these due to hepatic toxicity (Figure). From day 0–120 after transplantation in the universal prophylaxis and targeted periodes, respectively, 56% and 11% had ≥1 episode of grade ≥2 ALT increase, 5% and 13% had ≥1 episode of grade ≥2 increased creatinine related to high CNI plasma-concentration level, and 50% and 7% had acute rejection related to low CNI plasma-concentration (Table). Among patients from the universal prophylaxis period 45% had a low CNI plasma-concentration episode related to discontinuation of voriconazole.

**Conclusions:** Premature discontinuation rates of voriconazole prophylaxis were high, mainly due to hepatic side-effects. Episodes of low concentrations of immunosuppressives and acute rejections were more frequent during the period with universal- vs. targeted antifungal prophylaxis and a large proportion of low CNI episodes were related to discontinuation of voriconazole. This underlines the challenges related to the use of triazole prophylaxis in the lung transplant population and the importance of frequent dose adjustment of CNI.

## S07.5 Risk Factors for and Outcomes of Invasive Aspergillosis Following Kidney Transplantation in the United States

**Daniel Friedman ^1,2^, Bradley Johnson ^2^, Walter Kremers ^2^, Paschalis Vergidis ^1,2^**

^1^ Division of Infectious Diseases, Department of Medicine, Mayo Clinic
^2^ William J. Liebig Center for Transplantation and Clinical Regeneration, Mayo Clinic

**Objectives:** Kidney transplant (KTx) recipients are at increased risk of invasive aspergillosis (IA). The goal of this study was to determine risk factors for IA using a national database and assess its association with mortality and graft loss following KTx.

**Materials & Methods:** We performed a retrospective analysis using the United States Renal Data System (USRDS) database, which collects data from of Centers for Medicare & Medicaid Services (CMS) and the United Network for Organ Sharing (UNOS). We included patients who received KTx between 1986 and 2017 with follow-up until December 2018. Patients with IA were identified using ICD codes and matched 1:4 with controls by time and center of transplant. Characteristics of patients with IA were compared to those of patients without IA using chi-square and *t*-tests. Using logistic regression, we compared cases to matched controls to identify factors associated with increased odds of IA. We calculated the impact of IA on mortality and graft failure using Cox regression, Kaplan-Meier estimates and log-rank test.

**Results:** We matched 380 KTx recipients with IA to 1520 non-infected controls. The mean age at transplant was 53.7 years, and 59.5% of patients were male. The main indications for KTx were diabetic nephropathy (n = 560, 29.5%), glomerular disease (n = 394, 20.7%), polycystic kidney disease (n = 201, 10.6%) and hypertensive nephropathy (n = 111, 5.8%). One thousand fifty-four (55.4%) received a cadaveric allograft.

The median time to diagnosis of IA following KTx was 2.1 years (IQR, 0.5–7.9 years). Patients with IA were older (55.5 vs. 53.2 years, *p* < 0.01), had a longer time on dialysis pre-transplant (3.7 vs. 1.6 years, *p* < 0.01), and were more likely to have received a cadaveric allograft (74.2% vs. 50.8%, *p* < 0.01). Patients with IA were more likely to be Black (21.3% vs. 15.6%, *p* < 0.01) or American Indian (2.4% vs. 0.3%, *p* < 0.01) and were less likely to be White (69.5% vs. 78.2%, *p* < 0.01). The indication for KTx was more commonly diabetic nephropathy in those with IA (36.3% vs. 27.8%, *p* < 0.01) and less commonly polycystic kidney disease (5.8% vs. 11.8%, *p* < 0.01). We compared 31 comorbidities that are included in the Elixhauser comorbidity index (ECI). There were no significant differences between cases or controls for any comorbidities or for the mean composite ECI.

Within the study period, 607 patients (31.9%) died. One-year mortality was higher in patients with IA (aHR = 6.00, 95% CI [4.21–8.56], *p* < 0.01). Allograft failure occurred in 410 patients (21.6%). One-year graft failure was also higher in patients with IA (aHR = 4.76, 95% CI [2.61–8.67], *p* < 0.01).

**Conclusions:** Factors associated with increased odds of developing IA included older age, being American Indian, and longer time on dialysis pre-transplant. The latter highlights the importance of timely transplantation to mitigate the risk of IA post-transplant, particularly in patients with diabetic nephropathy or older age. The inequality of IA amongst the various races emphasizes the differing barriers to and social determinants of health faced. To decrease IA-related mortality and graft loss post-transplant, an individualized approach to antifungal prophylaxis is important, especially in centers with diverse transplant populations.

## S08.3 Prevalence of Chronic Pulmonary Aspergillosis in Patients with Tuberculosis

**Felix Bongomin**

**Abstract:** Pulmonary tuberculosis (PTB) is the most important risk factor and the differential diagnosis of chronic pulmonary aspergillosis (CPA), especially in tuberculosis endemic areas. However, there is a striking overlap in both the clinical and radiological presentation of these two diseases. After PTB treatment, residual cavities remain in 20 to 40% of the patient’s lungs. From published studies, in patients with CPA, treated PTB is the primary underlying respiratory condition in 17–93% of cases.

The importance of treated PTB in the pathogenesis of CPA was demonstrated in the mid to late 1960s by a large cohort study at over 50 clinics in the Great Britain. In this study, the prevalence of CPA increased from 14% at the end of TB therapy to 22% 3 years later. Several recent studies have been published describing the incidence of CPA in various situations, particularly as a co-infection with PTB, as a differential diagnosis of smear negative TB, as a consequence of TB, and in many cases years after TB. This talk will focus on these new insights.

**Keywords:** chronic pulmonary aspergillosis; pulmonary tuberculosis; incidence; prevalence

## S08.4 Prevalence of Chronic Pulmonary Aspergillosis among Active Pulmonary Tuberculosis Patients with Persisting Symptoms in Uganda

**Martha Namusobya ^1^, Felix Bongomin ^2^, John Mukisa ^3^, David Denning ^4^, Shailendra Prasad ^5^, Christine Sekaggya-Wiltshire ^1^**

^1^ Infectious Diseases Institute
^2^ Department of Medical Microbiology, Gulu University Medical School
^3^ College of Health Sciences, Makerere University
^4^ Manchester Fungal Infection Group, University of Manchester
^5^ University of Minnesota

**Objectives:** Treated pulmonary tuberculosis (PTB) is the most important risk factor for chronic pulmonary aspergillosis (CPA) with a significant impact on their health-related quality of life. However, recent evidence suggests CPA may occur before or during active PTB treatment. This study aimed at determining the prevalence and associated factors of CPA among PTB patients with persistent sysptoms despite treatment.

**Materials and Methods:** We conducted a prospective, observational cohort study on drug sensitive PTB patients with persisting pulmonary symptoms after 2 months of anti-TB treatment at the TB Treatment Unit, Mulago National Referral Hospital, Kampala, Uganda. Patients characteristics were documented using a semi-structured questionnaire. Sputum samples were collected for all patients and high volume culture performed. Serum *Aspergillus* IgG/IgM lateral flow assay (LFA) was performed and visually read. CPA was defined based on (1) Persisting pulmonary symptoms of cough or hemoptysis following PTB treatment with a total symptom duration >3 months; (2) Chest X-ray (CXR) showing cavities, infiltrates, fungal ball, peri-cavitary fibrosis, or pleural thickening; and (3) evidence of *Aspergillus* infection (positive sputum culture or an *Aspergillus* LFA).

**Results:** We enrolled a total of 83 patients, 51 (61.5%) were male. The median age was 31.5 years (range: 18–69). Twenty-seven (32.5%) participants were HIV positive. Twenty-three (27.7%) sputum sampes grew *Aspergillus niger* and 4 (4.8%) grew *A. fumigatus*. Only 10 (12%) participants tested positive on the *Aspergillus* IgG/IgM LFA. On CXR, 5 (6.0%) participants had a fungal ball, 29 (34.9%) had cavities and 34 (41%) had pleural thickening. Overall, The prevalence of CPA was 28.9% (n = 24). CPA was independently associated with prior PTB (adjusted odds ratio (aOR): 16.7 (95%CI: 2.6–106.5, *p* = 0.003), age >32 years (aOR: 0.1, 95% CI: 0.02–0.58, *p* = 0.01) and far advanced CXR changes (aOR: 10.9, 95%CI: 2.6–45.7, *p* < 0.001). The sensitivity, specificity, positive predictive value and negative predictive value of the *Aspergillus* LFA were 37.5%, 98.3%, 79.5%, and 90%, respectively.

**Conclusions**: CPA is highly prevalent among active PTB patients with persistent respiratory symptoms, especially those with history of prior PTB treatment and those with adavanced CXR changes. *A. niger* is a common species of *Aspergillus* in our setting. Aspergillus IgG/IgM LFA is highly specific but less sensitive for the diagnosis of CPA in Uganda. We recommend routine screening for CPA among these subset of patients.

## S08.5 CPAepi: A Study to Investigate Epidemiological Characteristics of *Aspergillus* spp. Isolated from CPA Patients

**Juan Carlos Soto Debran ^1^, Ioannis Tommos ^2^, Aleksandra Barac ^3,4^, Helmut J. F. Salzer ^5^, Eva Van Braeckel ^6^, Oxana Munteanu ^7^, Fabian Leo ^8^, Danila Seidel ^9^, Josep Meletiadis ^10^, Christophe Hennequin ^11^ and Ana Alastruey-Izquierdo ^1^**

^1^ Micology Reference labortory, Instituto De Salud Carlos Iii
^2^ 2nd Pulmonary Medicine Department, “Attikon” University General Hospital, Medical School, National and Kapodistrian University of Athens
^3^ Faculty of Medicine, University of Belgrade
^4^ Clinic for Infectious and Tropical Diseases, Clinical Centre of Serbia
^5^ Department of Pulmonology, Kepler University Hospital
^6^ Ghent University Hospital
^7^ Department of Pneumology & Allergology, State University of Medicine and Pharmacy “Nicolae Testemitanu”
^8^ Department of Pulmonary Medicine, ELK Thorax Center
^9^ Department I of Internal Medicine, University Hospital Cologne
^10^ Clinical Microbiology Laboratory, “Attikon” University General Hospital, Medical School, National and Kapodistrian University of Athens
^11^ Service de Parasitologie—Mycologie, Hôpital Saint-Antoine

**Objectives:** Chronic pulmonary aspergillosis (CPA) is a severe fungal pulmonary infection with a high morbidity and mortality. Information on the *Aspergillus* species and resistance pattern is of crucial importance in the management of CPA patients. However, a very limited number of studies reported 2–54% azole resistance rates. We present the epidemiology and antifungal susceptibility profile of *Aspergillus* species collected from CPA patients via CPAnet.

**Methonds & Materials:** Seventy six *Aspergillus* strains isolated from CPA patients were collected from seven European centers and sent to the Mycology Reference Laboratory of the National Centre for Microbiology in Spain. Isolates were sub-cultured and identified by means of ITS, BenA and/or calmodulin sequencing. Antifungal susceptibility profiles according to the EUCAST E.Def 9.3.2 method were determined for amphotericin B (AMB), itraconazole, voriconazole, posaconazole, isavuconazole, terbinafine, anidulafungin, caspofungin and micafungin. EUCAST clinical species-specific breakpoints were used to define resistance. For species with no breakpoints, ECOFFs were used to detect non-wild type phenotypes.

**Results:** *Aspergillus fumigatus* complex was present in 78.7% of all isolates (n = 59), followed by *A. niger* complex (n = 7; 9.3%), *A. flavus* complex (n = 6; 8%), *A. nidulans* complex (n = 2; 2.7%), and *A. terreus* complex (n = 1; 1.3%). Resistance in *A. fumigatus* complex to itraconazole, isavuconazole, posaconazole and AMB was 5% and 9% to voriconazole. In *A. flavus* complex, 17% of the isolates were resistant to itraconazole and isavuconazole while non-wild type were resistant to voriconazole and posaconazole. All isolates from *A. nidulans* complex were resistant to isavuconazole. Non-wild type isolates were found to be resistant for itraconazole (29%), voriconazole (29%) and posaconazole (43%) in *A. niger* complex. Echinocandins showed low MICs to all isolates tested but three strains that showed elevated MICs.

**Conclusions:** *Aspergillus fumigatus* was the most frequent species, but species from other complexes including cryptic species represent over 40% of the isolates. Resistance to azoles was present in *A. fumigatus, A. flavus* and *A. nidulans* complexes and to AMB in *A. fumigatus* complex. Treatment and outcome data are required to gain insight into potential mechanisms of resistance, and to evaluate the clinical impact of these findings.

## S09.1 Distribution and Antifungal Susceptibility of Yeasts Causing Invasive Fungal Diseases in China: An Update from the CHIF-NET Study

**Yao Wang**

Peking Union Medical CollegeHospital, China

The CHIF-NET study is a laboratory-based, nationwide multicenter study of invasive yeast infections in China, which was initiated in August 2009. A total of 87 hospitals had participated in this program for a period of tensurveillance years.All Candida, Cryptococcus, and otheryeast strains recovered from blood; other sterile body fluids, includingascitic fluid and peritoneal dialysate fluid; pus; and tissue from patientswith invasive yeast infections were included in this study. Yeast strains from bronchoalveolar lavage (BAL) fluid samples, centralvenous catheter (CVC) tips, and the gastrointestinal tracts (e.g., biliarytract fluid [aseptically collected]) of patients with invasive infections weretested; however, yeast strains from urine and the genital tract and othersconsidered colonizers were excluded. Isolates of the same species and ofthe same susceptible or resistant biotype profile from the same site of agiven patient that were recovered at a different time were considered duplicatesand also excluded. All isolates were forwarded to a central laboratory(Department of Clinical Laboratory, Peking Union Medical CollegeHospital) for study.

Isolates collected from 2010 and 2011 were identified by DNA sequencing of the fungal rDNA internal transcribed spacer region supplemented with D1/D2 domain of the 28S rRNA gene. From 2012, species identification was carried by matrix-assisted laser desorption ionization-time of flight mass spectrometry (MALDI-TOF MS). For any isolates with no identification or uncertain identification results to species level by MALDI-TOF MS, rDNA sequencing was performed as ‘gold standard’. Susceptibility to fluconazole and voriconazole was determined using the Clinical and Laboratory Standards Institute (CLSI) disk diffusion method for isolates collected from 2010 to 2014. From 2015, the in vitro susceptibility to nine antifungal agents, including fluconazole, voriconazole, itraconazole, posaconazole, caspofungin, micafungin, anidulafungin, amphotericin B and 5-flucytosine, was determined using Sensititre YeastOneTM YO10 methodology (Thermo Scientific, Cleveland, OH, USA) following manufacturer’s instructions.

In this presentation, we’ll report the distribution and antifungal susceptibility of yeasts causing invasive fungal diseases in China, anupdate from the CHIF-NET Study.

## S09.2 A Global Team Effort—FungiScope

**Danila Seidel**

FungiScope grew from a registry collecting clinical cases of emerging fungal infections initiated at the University Hospital of Cologne in 2003 by the European Confederation of Medical Mycology (ECMM) and The International Society for Human & Animal Mycology (ISHAM) to a global network with partners in currently 85 countries. Today, the registry includes also *Aspergillus*-related infections. Scientists and clinicians from around the world merge their expertise to improve clinical management and eventually outcome of patients with fungal infections. With their shared ambition, the network has a record of accomplishment of several publications on the epidemiology and treatment of fungal infections. Registry data were incorporated in the current global guideline on diagnosis and management of mucormycosis and infections caused by rare yeasts. During the pandemic, COVID-19 associated aspergillosis and mucormycosis came into focus. In this presentation, you will be updated on the latest findings and ongoing studies within the network. You will hear about the clinical impact of super rare fungal infections that clinicians might see once in their life and about the current struggle with COVID-19 associated fungal infections among others.

## S09.3 FUNGINOS—The Fungal Infection Network of Switzerland

**Nina Khanna**

The Fungal Infection Network of Switzerland (FUNGINOS) is a registered association with seat in Lausanne. This national hospital network was founded by infectious diseases specialists in 2005. FUNGINOS has developed a research network of infectious diseases specialists, clinical microbiologists and physicians of other specialties from all five universities and two tertiary care university-affiliated hospitals with expertise in conducting clinical, epidemiological, diagnostic, and microbiological studies on invasive fungal infections. Aim of the FUNGINOS association is to promote clinical and basic research on the epidemiology, diagnosis, treatment, and prevention of invasive fungal infections. The ultimate goal is to promote good clinical practice and medical education as well as to improve management and outcome of patients suffering from these life-threatening complications.

The present talk will give an overview of recent ongoing and published research work of FUNGINOS.

## S09.4 Update from ECMM and International Registries: FungiScope Candida/CandiReg

**Philipp Köhler**

**Objectives:** Invasive Candida infections (ICI) are among the most frequent bloodstream infections. They are associated with high morbidity and mortality. Due to medical progress, the number of critically ill patients at risk for ICI are continuously rising. The emergence of the multidrug-resistant species C. auris, has caused outbreaks worldwide and led to clinical alerts in United States and European healthcare facilities. The ECMM Candida Registry (FungiScope Candia/CandiReg) was founded in January 2018, triggered by the C. auris outbreaks in Spain and the United Kingdom. The main objective is to overcome the lack of knowledge on epidemiology, clinical course and molecular characteristics of invasive Candida infections and to function as a platform for international multicentre studies and surveillance of multinational C. auris outbreaks.

**Materials & Methods:** The platform is an international non-interventional multicentre registry project. The registry was founded in January 2018 and is ongoing without a defined endpoint. Treating physicians and medical microbiologists alike are invited to participate in the collection of clinical data and fungal isolates from cases of candidemia and tissue-invasive candidiasis. CandiReg uses an electronic case report form (eCRF) using the online electronic data capture platform www.clinicalsurveys.net. Case registration is on a voluntary basis. Target groups are patients with invasive candidiasis or candidemia.

**Results:** The ECMM Candida Registry (FungiScope Candia/CandiReg) serves as platform for international cooperation and studies on attributable mortality, Candida-reactive T cells, evaluations of guideline adherence, and the third multicentre ECMM study on incremental costs associated with nosocomial invasive candidiasis in Europe, CANDIDA III was initiated taking advantage of this platform. The CANDIDA III study focuses on evaluating the attributable mortality and costs as well as diagnostic and therapeutic approaches (including prolonged hospital stay for completion of parenteral antifungal treatment) of nosocomial invasive candidiasis in Europe. As a secondary objective, it will evaluate the antifungal resistance among Candida spp. causing invasive disease across Europe. The study will use a matched case-control design, which will allow the implementation of health economic analysis on the incremental costs associated with invasive Candida infections.

**Conclusions:** The platform can be used for future multinational surveillance studies on invasive candidemia in Europe. With this registry, we collect real-life data with long-term observations. The registry will provide controlled or uncontrolled level II evidence and is ready in case of an outbreak situation. In conclusion, the CandiReg platform promotes international collaboration, increases the quality of available evidence on invasive Candida infection and can contribute to improvement of patient management.

## S09.6 CPAnet: An International Chronic Pulmonary Aspergillosis Registry

**Eva Van Braeckel**

Chronic pulmonary aspergillosis (CPA) is a chronic fungal infection of the lung associated with high morbidity and mortality. The international CPA Research Network (CPAnet) was founded in 2017, followed by the creation of its multinational and multicenter CPAnet registry launched in 2018.

The CPAnet registry is a web-based registry, approved by the Institutional Review Board and Ethics Committee (EC) of the University Hospital Cologne, Germany, as well as by the local ECs of the other participating centers. As of February 2020, the CPAnet registry was awarded a Clinical Research Collaboration (CRC) by the European Respiratory Society (ERS).

The aim of this registry is to increase awareness in pulmonologists and infectious diseases specialists worldwide, to improve CPA knowledge and patient care, and to allow for future clinical and epidemiological research. An interim analysis of the data will be presented to lift a tip from the veil of the wealth of structured information embedded within the CPAnet registry. Additional clinical sites and reference centers are highly welcomed to join this clinical research collaboration (info@cpanet.eu).

In the near future, this registry will indefinitely allow for multicentric clinical and epidemiological research on CPA of crucial importance, aiming to fill the lack of previous evidence on disease management.

## S10.1 Diagnosis of Candida: Culture, β-Glucan and Mannan

**Elizabeth Johnson**

For the purposes of this presentation I use ‘*Candida*’ in the widest sense to include all those unrelated yeast species which taxonomically are now more accurately classified in different genera but which were previously in the taxonomic grouping known as ‘*Candida*’. Whilst the diagnosis of *Candida* infection is arguably one of the most straightforward in medical mycology, the need for more rapid diagnosis of candidaemia to improve the prognosis, especially in the face of sepsis, has driven biomarker development as an adjunct to, and ideally to pre-empt, culture results. Moreover biomarker detection is useful in conditions where blood culture is often not revealing such as endocarditis, chronic candidosis, CSF analysis in cerebral candidosis; and is also useful in prognosis.

Undoubtedly culture of blood, other sterile fluid or tissue remains the gold standard, with candidaemia a proven diagnosis. Blood culture techniques have improved over the years and have the advantage that they yield an organism. Identification methods for yeast have dramatically improved from the days of phenotypic and biochemical analysis, with rapid proteomic techniques using MALDI-ToF facilitating identification in as little as 20 min. Species identification is extremely helpful in predicting the likely susceptibility but isolation in culture also allows specific susceptibility testing.

Two biomarkers, β-D-glucan and mannan, have been employed on blood and CSF to detect the presence of *Candida*. The most sensitive is β-D-glucan detection, however this is a cell wall component of many, in fact the majority of clinically relevant fungal species, so it lacks specificity. A positive result is an indication of the presence of β-glucan which may be from a fungal source, including *Candida* infection, but may also be due to gut leakage of dietary β-glucans, contamination from cellulose membranes and dressings, gram negative bacteraemia, the administration of filtered blood products such as IVIg and even some antibiotic batches. A positive result is thus more useful in a patient where there is a high index of suspicion and no accompanying likely alternative source of β-glucan. Sensitivity and specificity reports therefore vary quite widely depending on the patient group.

Mannan is a cell wall component of many yeasts, but has a greater specificity than β-glucan. The issue with this biomarker is its propensity for positivity in patients merely colonised with yeast so it is an unreliable marker of infection. Nevertheless it has a place in confirming the CSF diagnosis of cerebral candidosis, chronic candidosis in the presence of hepato-splenic abscesses and *Candida* endocarditis in the presence of vegetations and a high *Candida* antibody titre, and can be a helpful prognostic marker in these conditions.

A major issue with biomarker testing has been turn-around-times as the benefit of a result designed to pre-empt culture is lost if the result takes several days to be returned to the clinician. In many laboratories, biomarkers are same-day tests and even during the massive pandemic-associated surveillance biomarker increase we maintained testing within 24 h. Many laboratory information management systems (LIMS) including ours, allow remote access so distant laboratories can access results on their patients as soon as they are authorised. A more recent UK development is the National Pathology Exchange (NPex) with the ambition to connect all UK labs through a single exchange hub, thus enabling test requests and pathology results to be sent digitally from and to any laboratory in a matter of seconds, whilst specimens are sent by an overnight tracked transport system. Setting this up takes a bit more effort and IT expertise on both sides but several of our major users are now connected.

## S10.2 The Role of PCR and T2 MR

**Cornelia Lass-Flörl**

**Objectives:** Mortality rates due to invasive candidiasis (IC) remain unacceptably high, in part because the poor sensitivity and slow turn-around time of cultures delay the initiation of antifungal treatment. Cultures are negative in ~50% of invasive candidiasis.

**Materials & Methods:** Based on a literature research, data are emerging for the performance of nonculture tests such as mannan/antimannan, *Candida albicans* germ tube antibody, 1,3-β-D-glucan, PCR, and the T2Candida panel in diagnosing both candidemia and deep-seated candidiasis. For tests to be useful, the pretest likelihood of invasive candidiasis and test performance for the most common disease manifestation in a given patient must be understand.

**Results:** Several PCR assays, including commercially available kits, have been developed for the detection of Candida spp. The high sensitivity makes these assays appealing tools for the early diagnosis of IC. Depending on the method used for DNA extraction, free DNA or intact pathogen cells are detected. This difference can be relevant for patients under antifungal therapy as this may specifically affect the outcome of molecular tests that detect only intact cells. On the other hand, the interpretation of positive results from assays detecting free DNA can be challenging. Thus, the position of Candida PCR assays in the diagnostic algorithm of IC is not easy to establish. As published data show, Candida PCR has a higher sensitivity than blood culture but shows the best efficacy when used in conjunction with blood cultures and/or additional tests.

The T2Candida system was recently licensed by the US FDA and was designated as superior to conventional blood culture systems for the detection of candidemia. In detecting the five most commonly recovered medically important Candida spp. (*Candida albicans, Candida tropicalis, Candida parapsilosis, Candida glabrata,* and *Candida krusei*), the T2Candida system utilizes a T2 magnetic resonance technology coupled with pathogen-derived nucleic acid amplification to identify the five target pathogens within 2 to 5 h from the time of initiation of the assay. Studies in adults have demonstrated a more rapid time to detection, in comparison with that of conventional blood cultures. Blood cultures can detect 1 CFU/mL of most Candida sp. and remain the gold standard, hence it is unknown what proportion of the T2Candida positive and blood culture negative results represent false-negative cultures, deep-seated occult infection with negative blood cultures, or false-positive T2Candida results.

**Conclusions:** The quick turn-around-time of T2Candida, ability to rapidly detect potential azole-resistant species, e.g., C. krusei and consequent earlier initiation of appropriate antifungal therapy would be expected to produce a noticeable survival benefit.

## S10.3 Mixed Yeast Infections—Common or not?

**Ana Alastruey-Izquierdo**

Mycology Reference Laboratory, National Centre for Microbiology, Madrid, Spain

**Objectives:** Invasive candidiasis remains one of the most prevalent systemic mycoses. Several studies have documented the presence of mixed yeast infections but data is very limited. We conducted a multicenter prospective study to describe the epidemiology and microbiological characteristics of mixed yeast infections causing invasive candidiasis.

**Materials & Methods:** Thirty-four centers from 14 countries participated. Samples were collected for a period of six months. All mixed yeast infections in sterile samples were isolated and sent to a reference laboratory to conduct molecular identification and antifungal susceptibility testing by EUCAST methodology.

**Results:** A total of 6895 yeast cultures were identified and mixed yeast infections occurred in 150 cases (2.2%). Most of the centers were from Europe, with an overall rate of 4.2% (118 out of 2840 yeast cultures). Of 122 cases, the most frequent combinations were *Candida albicans/C. glabrata* (42, 34.4%), *C. albicans/C. parapsilosis* (17, 14%), and *C. glabrata/C. tropicalis* (8, 6.5%). All *Candida* isolates were susceptible to amphotericin B, 6.4% were fluconazole-resistant, and two isolates (1.6%) were echinocandin-resistant.

**Conclusions:** The global rate of mixed yeast infections was 2.2%, 4.2% in Europe (the most represented continent). *C. albicans/C. glabrata* was the most common combination, but a high diversity of combinations and distributions was identified. Resistance to echinocandins was present but rare and fluconazole resistance rates were variable. As different susceptibility patterns can be identified in MY infections, it is important to accurately identify the species involved and to perform susceptibility testing to support the clinical management of these infections.

## S11.3 Mycobiome and Invasive Candidiasis

**Tobias Hohl**

The intestinal microbiota is a complex community of bacteria, archaea, viruses, protists and fungi. While the composition of bacterial constituents has been linked to immune homeostasis, infectious susceptibility, and clinical outcomes in bone marrow transplantation, the role of non-bacterial constituents and of cross-kingdom microbial interactions in these processes is poorly understood. Fungi represent a major cause of infectious morbidity and mortality in immune-compromised individuals. To examine the relationship of intestinal fungi (i.e., the mycobiota) with fungal bloodstream infections (BSI) we integrated an optimized bioinformatics pipeline with high-resolution mycobiota sequencing and comparative genomic analyses of fecal and blood specimens from recipients of allogeneic hematopoietic cell transplants (allo-HCT).

First, we conducted a small case-control study to compare the intestinal mycobiota of allo-HCT patients with (n = 8) and without (n = 7) *Candida* BSI. Patients with Candida BSI experienced a prior marked intestinal expansion of pathogenic *Candida* species; this expansion consisted of a complex dynamic between multiple species and subspecies with a stochastic translocation pattern into the bloodstream. The intestinal expansion of pathogenic *Candida* species was associated with a significant loss in bacterial burden and diversity, particularly in the anaerobes.

Second, we conducted a larger observational study in allo-HCT patients (n = 156) examine the overall density and biodiversity of intestinal fungi during allo-HCT. In this cohort, we found that global measures of fungal density and diversity were stable, but that the species composition changed drastically from day to day. We identified a subset of patients with fungal dysbiosis characterized by culture positivity, stable expansion of *Candida parapsilosis* complex species, and distinct trans-kingdom microbiota profiles. These patients had worse overall survival and higher transplant-related mortality, independent of candidemia. Thus, simultaneous analysis of intestinal fungi and bacteria identifies dysbiosis states across kingdoms that may promote facilitate invasive disease and poor allo-HCT outcomes. These findings support microbiota-driven approaches to identify patients at risk for fungal BSI and adverse allo-HCT outcomes for pre-emptive therapeutic intervention.

## S11.4 Mycobiome and Microbial Interactions

**Ilse Jacobsen**

**Objectives:** As both commensal and pathogen, *Candida* interacts not only with the host but also with members of the bacterial microbiota. While antagonistic interactions between *Candida* and bacteria have received considerable attention, synergistic interactions are less well understood. One of the most common *Candida*-associated bacteria identified during mixed infections are enterococci, especially *E. faecalis*. Our work and that by others demonstrated synergistic interactions between these species, and I will discuss some of the underlying mechanisms identified.

**Materials & Methods:** We used a mucus-producing in vitro enterocyte model with different MOIs and *C. albicans* and *E. faecalis* strains to determine factors contributing to synergistic damage. The role of physical interactions was analysed using transwell inserts and culture supernatants. The possible impact of metabolites was investigated by metabolome analysis and spiking experiments. As a proof-of-concept for the in vivo relevance we employed a murine oropharyngeal candidiasis model.

**Results:** Consequences of *E. faecalis*-*C. albicans* interactions for enterocyte damage in vitro depend on the bacterial strain and synergism requires expression of the *E. faecalis* cytolysin. Cytolysin-dependent synergism was confirmed in vivo in mice. Synergistic damage was independent of filamentation, candidalysin, and was also promoted by less pathogenic fungal species. While physical contact of fungi and bacteria contributed to synergistic damage, enhanced bacterial damage was also observed with *C. albicans*-conditioned media. We identified glucose depletion by *Candida* as one factor that promotes host cell damage by *E. faecalis*.

**Conclusions:** Our data and results from other groups with other bacteria indicate that certain bacterial-fungal combination have to potential to aggravate the course of infection in vitro and in animal models. This is likely also the case in human patients, with possible consequences for treatment and outcome. A deeper understanding on which bacteria and bacterial factors act synergistically with *Candida* is however necessary to determine which patients might be at increased risk and in which cases treatment should be adjusted to target specific coinfections.

## S11.5 Development of a Novel Mycobiome Diagnostic for Fungal Infection

**Danielle Weaver, Mike Bromley and Paul Bowyer**

University Of Manchester

**Objectives:** Pan-fungal diagnostics which simultaneously allow speciation would be a vast improvement to clinical mycology. Within the last decade, the human mycobiome has been increasingly characterised using NGS targeting the internal transcribed spacer (ITS) regions. Mycobiome analysis is a strong candidate diagnostic, with the potential to identify all species within a sample and possibly in a quantitative manner. ITS targets give broad coverage and high sensitivity, but their ability to provide an accurate, quantitative representation of a fungal community has been questioned. The variable copy number of ITS regions in fungal genomes and the presence of intra-species variation can introduce bias. To avoid these issues, this study aimed to develop a novel, alternative amplicon target for use as an NGS fungal diagnostic.

**Materials & Methods:** A novel DNA target was designed to enable mycobiome analysis using the latest Illumina sequencing platform, the iSeq100, whose relatively low cost, small size and ease-of-use makes it highly suitable for a diagnostic laboratory. To enable automated analysis and rapid results, an in-house bioinformatics workflow and sequence database were also developed. Pilot sequencing of mock fungal communities was performed to compare the new assay and established ITS1 method using the Illumina iSeq100 and MiSeq sequencing platforms.

**Results:** The novel assay performed similarly on both sequencing platforms and could successfully identify *Aspergillus, Penicillium, Candida, Cryptococcus, Rhizopus, Fusarium* and *Lomentospora* species. The new target was also capable of differentiating closely related species such as *Aspergillus fumigatus* and *Aspergillus fischeri*. In addition, it outperformed ITS1 at identifying *A. fumigatus, Rhizopus arrhizus, Fusarium oxysporum* and other filamentous pathogens in mixed fungal communities (in the presence or absence of background human DNA). Lastly, the assay could detect as few as 2 haploid genome equivalents of *A. fumigatus* from spiked sputum.

**Conclusions:** This study has developed and tested a novel metabarcoding target and found the assay to outperform the commonly used ITS1 region at identifying filamentous fungi. The assay is a promising diagnostic candidate that could provide affordable NGS analysis to a clinical mycology laboratory.

## S11.6 Effect of Exposition to Broad-Spectrum Antibiotics on the Gut Mycobiota of Healthy Volunteers

**Margot Delavy ^1^, Charles Burdet ^2,3^, Natacha Sertour ^1^, Stevenn Volant ^4^, Nathalie Grall ^2^, Amine Ghozlane ^4^, Xavier Duval ^2,5^, France Mentré ^2,3^, Christophe d’Enfert ^1^, Marie-Elisabeth Bougnoux ^1^ and for the CEREMI Group**

^1^ Institut Pasteur, INRA, Unité Biologie et Pathogénicité Fongiques
^2^ Université de Paris, INSERM, IAME, UMR 1137
^3^ AP-HP, Departement of Epidemiology, Biostatistic and Clinical Research, Hôpital Bichat
^4^ Institut Pasteur, Bioinformatics and Biostatistics Hub-C3BI-USR 3756 IP CNRS
^5^ INSERM Clinical Investigation Center 1425

**Objectives:** Antibiotic exposure impacts the intestinal bacterial microbiota, but little is known on its effect on the fungal intestinal microbiota (mycobiota) in humans. We studied the effects of 3rd generation cephalosporins (3GC), a class of β-lactam antibiotics, on the gut mycobiota of healthy volunteers and their impact on the proliferation of the opportunistic fungal pathogen *Candida albicans*.

**Materials & Methods:** Two groups of 11 healthy volunteers received either intravenous ceftriaxone (1 g/24 h) or cefotaxime (1 g/8 h) for 3 days. Fecal samples were collected before treatment at days (D)-15, -7, -1, during treatment at D1, D2 and D3, and after treatment at D4, D7, D10, D15, D30, D90, D180. We quantified fungal and *C. albicans* DNA by qPCR targeting 18S rRNA or *C. albicans* internal transcribed spacer (ITS) 2 rRNA region, respectively. We studied gut mycobiota by sequencing the ITS1 rRNA region (HiSeq platform, Illumina). Finally, β-lactamase activity was quantified in the fecal samples by measuring nitrocefin—a chromogenic cephalosporin substrate–hydrolysis.

**Results:** Before 3GC exposure, the volunteers’ mycobiota was characterized by a high within and between diversity (Bray-Curtis dissimilarity within/between median: 0.85/0.88) and a low alpha diversity (median of 25 Operational Taxonomic Units per sample, Shannon Index of 1.23). By qPCR, *C. albicans* DNA could be detected in 20/21 volunteers, leading to a colonization rate of 95.2%. In comparison, culture and sequencing methods underestimated this colonization rates (18.2% and 45.5% colonization rate, respectively).

Discriminative analysis identified disparately represented fungal taxa in the volunteers’ samples before and after antibiotic treatment. Two days after the start of 3GC treatment, we observed a long-term expansion of the mycobiota over the microbiota, remaining for 30 days. Penicillium sp. relative abundance (RA) was significantly reduced between D1 and D4 (Wilcoxon test; q-value of 0.013, 0.005, 0.003 and 0.0002, respectively). More interestingly, the RA of *C. albicans*, *Saccharomyces* sp. and *S. cerevisiae* were increased at D4 (Wilcoxon test; q-value of 0.042, 0.014 and 0.026, respectively). In addition of sequences analyses, *C. albicans* increase was also confirmed with qPCR data, with the maximum reached at D2 (Wilcoxon test; *p*-value of 0.026).

Finally, we observed a negative correlation between the changes in β-lactamase activity and the changes of *C. albicans* DNA concentration for the D0D10 period (Spearman correlation; R: −0.59, *p*-value: 0.009). Indeed, in volunteers displaying a high increase of the β-lactamase activity after 3GC exposure, we could not observe any *C. albicans* proliferation. However, we observed a significant increase of *C. albicans* DNA at D4 in volunteers displaying a lower increase or even a decrease of β-lactamase activity (Wilcoxon test; *p*-value of 0.840, 0.008).

**Conclusions:** The 3GC treatment affects the mycobiota both at short and long-term and is linked to an increase of the fungal load, and, especially, of *C. albicans*. We identified the changes of β-lactamase activity as a potential key factor for the proliferation of *C. albicans*.

By having a better understanding of the factors behind *C. albicans* proliferation in the gut, we could identify new ways to prevent potentially life-threatening secondary infections by this pathogen.

## S12.2 Definition, Diagnosis and Treatment of COVID-19 Associated Pulmonary Aspergillosis—ECMM/ISHAM Consensus Guidance

**Philipp Köhler**

**Objectives:** In December, 2019, COVID-19 emerged from Wuhan, China, rapidly evolved into a pandemic. Multiple publications and reports of COVID-19-associated pulmonary aspergillosis (CAPA), describing CAPA as an additional contributing factor to mortality have raised the awareness towards fungal superinfections in COVID-19 patients. Initial descriptions of patients with suspected CAPA illustrated the challenge of to diagnose CAPA. This is due to the fact that the use of established definitions including host factors and clinical factors (including radiology) may not be optimal in this patient population. Facing these challenges to diagnose and manage patients with CAPA, there was an urgent need to study the epidemiology and characteristics of CAPA.

**Materials & Methods:** To facilitate this, the European Confederation for Medical Mycology and the International Society for Human and Animal Mycology assigned a group of experts to propose consensus criteria for a case definition of CAPA and to provide up-to-date management recommendations for the diagnosis and treatment. The group gathered 22 experts from 14 countries in 6 continents. Literature review of particular topics were performed in small groups, that contributed subsections to draft consensus definitions, which was agreed on after several rounds of revision.

**Results:** CAPA is proposed to be defined as possible, probable, or proven. These categories depend from the sample validity and thus diagnostic certainty. Recommended first-line therapy is either voriconazole or isavuconazole. If azole resistance is of concern, then liposomal amphotericin B is the drug of choice.

**Conclusions:** The proposed consensus definition for CAPA facilitate homogeneous classification of CAPA patients in registries and interventional clinical trials. Moreover, the definitions can be used in daily practice for managing patients with CAPA.

## S12.3 COVID Associated Mucormycosis

**Arunaloke Chakrabarti**

With the specter of COVID-19 pandemic, invasive fungal infections (IFIs) loom large across the globe. Among IFIs, COVID-19 associated mucormycosis (CAM) epidemic in low-and middle-income countries especially India has drawn attention of world authorities, as the outbreak is unprecedented and number of CAM cases in India exceeded 50% of global cases. A multi-center Indian study during September to December 2020 reported CAM cases at 0.27% and 1.6% of COVID-19 patients treated in hospitals and intensive care units respectively. The rise of number was 2.1-fold during the study period compared to 2019 at those centers. The patients acquired the infection at median 18 days post-COVID diagnosis. Hypoxia and inappropriate steroid therapy were identified as significant risk factors for acquiring CAM. Subsequently, the situation turns grave in India with >50,000 CAM cases during the second wave of COVID-19 (March to July 2021). The case numbers may be more than this, as the Government of India CAM dashboard mentions ‘It is very likely that the actual figures are considerably higher than this’. For the first time, CAM is declared as ‘Notifiable Disease’ in India. During this period, management of CAM cases has gone out of hands due to limited number of skilled health workers to diagnose and treat mucormycosis, and non-availability of anti-Mucorales drugs. The question that raised in everyone’s mind is—‘Why is the surge of CAM cases in India?’. Myriad hypotheses including high Mucorales in the environment, contamination of oxygen supplies, respiratory equipment, humidifier water, reused face masks, and zinc supplementation are proposed as reasons for the epidemic. But except high spore count in both indoor and outdoor environment, none of those hypotheses is proven. Our recent studies on zinc supplementation and multi-centre study on environmental factors also negated those hypotheses. Then, what are the reasons? Four factors including high spore count in environment, uncontrolled diabetes, inappropriate steroid use, and COVID-19 virus itself have been identified as possible reasons for the outbreak. The most likely narrative is that due to very high number of COVID-19 patients in the hospital during the second wave, doctors failed to do glycemic control of hyperglycemic patients and inadvertently used inappropriate dose to steroid to maintain oxygen saturation of those patients due to oxygen crisis. COVID-19 virus is known to damage β-cells of pancreas and cause enthothelitis, but it is not clear whether it impairs Mucorales-specific immunity. To tide over the antifungal availability crisis, ECMM and ISHAM jointly recommended an emergency alternate algorithm. Since the month of August 2021, with decrease in number of COVID-19 patients and better management protocol in India, the outbreak is controlled to certain extent, but unknown issues regarding the CAM epidemic haunt the minds of medical microbiologists across the globe.

**References**

1. Chakrabarti A. The recent mucormycosis storm over Indian sky. *Indian J. Med. Microbiol.* **2021**, *39*, 269–270.
2. Hoenigl, M.; et al. The emergence of COVID-19 associated mucormycosis: Analysis of cases from 18 countries. *Bioxriv* **2021**, https://doi.org/10.2139/ssrn.3844587.
3. Patel, A.; et al. Multicenter Epidemiologic Study of Coronavirus Disease-Associated Mucormycosis, India. *Emerg. Infect. Dis.* **2021**, *27*, https://doi.org/10.3201/eid2709.210934.
4. Muthu, V.; et al. Is there an association between zinc and COVID-19-associated mucormycosis? Results of an experimental and clinical study. *Mycoses* **2021**, *22*, https://doi.org/10.1111/myc.13365. Epub ahead of print. PMID: 34420245.
5. Muthu, V.; et al. Epidemiology and pathophysiology of CIVID-19 associated mucormycosis: India vs. the rest of the world. *Mycopathologia* **2021**, *2021*, https://doi.org/10.1007/s11046-021-00584-8.
6. Rudramurthy, S.M.; et al. ECMM/ISHAM recommendations for clinical management of COVID -19 associated mucormycosis in low- and middle-income countries. *Mycoses* **2021**, *64*, 1028–1037. Available online: https://governmentstats.com/mucormycosis/index.html (accessed on 8 October 2021).

## S12.4 Cytomegalovirus Infection and Invasive Fungal Disease

**Monica Slavin**

Invasive fungal disease (IFD) and viral infections frequently occur in immune compromised hosts, with viral infection preceding IFD. The first reports of this interaction came from the observation that solid organ (SOT) and haematopoietic stem cell transplant (HCT) recipients who developed end organ cytomegalovirus (CMV) invasive disease were at a significantly increased risk of developing both early and late onset IFD, particularly invasive aspergillosis, invasive mould disease and Pneumocystis pneumonia. The association with candidiasis in less clear. Further studies showed that CMV viremia, antigenemia and recipient CMV serostatus also increased the risk. CMV viremia or antigenemia increases the risk for IFD in the order of three times. However, CMV kinetics also influence risk. In HCT recipients the area under the curve of CMV antigenemia (number of positive cells and duration of antigenemia) impacts risk and in the critically ill immune suppressed patient with CMV viremia, the peak viral load influences risk for IFD. The combination of CMV viremia and influenza infection also increased the risk for IFD 25 times in one study in immunocompromised ICU patients.

CMV infection may increase the risk of IFD due to a number of common and pathogen-specific, host, treatment and environmental risk factors. The immune modulatory effects of CMV infection and associated viruses such as HHV6 and the impact of ganciclovir treatment will be described. When a HCT or SOT recipient develops CMV reactivation, the risk for IFD should be considered along with whether prophylaxis for mould or Pneumocystis infection is indicated. In particular, risks for neutropenia with ganciclovir, likely duration of ganciclovir use, presence of other viral infections and level of immune-suppression should be taken into account.

Further research is warranted to determine the biological interaction between CMV and IFD in order to better identify future patients at high risk of developing IFDs and interventions to decrease the risk.

## S12.5 Pulmonary Aspergillosis in Critically Ill Coronavirus Disease 2019 Patients—A Multinational Study

**Juergen Prattes, Joost Wauters, Daniele Roberto Giaccobe, Jon Salmanton-García, Johan Maertens, Marc Bourgeois, Marijke Reynders, Lynn Rutsaert, Niels Van Regenmortel, Piet Lormans, Simon Feys, Alexander Reisinger, Oliver A. Cornely, Tobias Lahmer, Mericela Valerio, Laurence Delhaes, Kauser Jabeen, Joerg Steinmann, Mathilde Chamula, Matteo Bassetti, Stefan Hatzl, Riina Rautemaa-Richardson, Philipp Koehler, Katrien Lagrou, Martin Hoenigl**

Medical University Of Graz

**Objectives:** Coronavirus disease 2019 (COVID-19) associated pulmonary aspergillosis (CAPA) has emerged as a complication in critically ill COVID-19 patients. The main objectives of this study were to determine the prevalence of CAPA in patients with COVID-19 in intensive care units (ICU) and to investigate risk factors for CAPA as well as outcome.

**Methods:** The European Confederation of Medical Mycology (ECMM) conducted a multinational study including 20 centers from nine different countries to assess epidemiology, diagnosis, risk factors, treatment and outcome of CAPA. CAPA was defined according to ECMM/ISHAM consensus definitions.

**Results:** A total of 592 patients were included in this study, including 11 (1.9%) patients with histologically proven CAPA, 80 (13.5%) patients with probable CAPA, 18 (3%) with possible CAPA and 483 (81.6%) without CAPA. CAPA was diagnosed a median of 8 days (range 0–31) after ICU admission predominantly in older patients [adjusted hazard ratio (aHR) 1.04 per year] with any form of invasive respiratory support (HR 3.4) and receiving tocilizumab (HR 2.45). Median prevalence of CAPA per center was 10.7% (range 1.7–26.8%). CAPA was associated with significantly lower 90-day ICU survival rate (29% in patients with CAPA vs. 57% in patients without CAPA; Mantel-Byar **p* < 0.001*) and remained an independent negative prognostic variable after adjusting for other predictors of survival.

**Conclusions:** Prevalence of CAPA varied between centers. CAPA was more prevalent among older patients receiving invasive ventilation and tocilizumab, and an independent strong predictor of ICU mortality.

## S12.6 Immunological Profiles of Patients with Influenza-Associated Pulmonary Aspergillosis (IAPA)

**Intan M.W. Dewi ^1,3^, Nico A.F. Janssen ^1,3^, Edwin Ardiansyah ^1^, Mihai G. Netea ^1,3^, Paul E. Verweij ^1,3^, Reinout van Crevel ^1^ and Frank L. van de Veerdonk ^1,3^**

^1^ Department of Internal Medicine, Radboud University Medical Center
^2^ Department of Medical Microbiology, Radboud University Medical Center
^3^ Radboudumc—CWZ Center of Expertise for Mycology

**Objectives:** Influenza-associated pulmonary aspergillosis (IAPA) has recently been recognized as a serious complication of severe influenza and causes high mortality in patients. In this study, we aim to characterize the inflammatory and immune responses of patients with severe influenza and IAPA.

**Materials and methods:** We compared the levels of circulating inflammatory markers, ex vivo cytokine production in peripheral blood mononuclear cells (PBMCs) stimulated with various ligands, and reactive oxygen species (ROS) induction in PBMCs and neutrophils in response to *A. fumigatus* conidia, between patients with influenza-associated pulmonary aspergillosis (IAPA), severe influenza without IAPA, and healthy controls.

**Results:** Severe influenza patients (with or without IAPA) have higher levels of circulating inflammatory markers compared to healthy controls, which may indicate a state of hyperinflammation. IAPA patients show specific increase in several proteins, including IL-5, compared to severe influenza patients without IAPA. Ex vivo production of innate cytokines in PBMCs were comparable between patients and healthy controls. Strikingly, production of IFN-γ after cell stimulation with several stimuli were significantly lower in patient with influenza regardless of *Aspergillus* infection. Cytokine production was not significantly different between IAPA patients and severe influenza patients without IAPA. Furthermore, *Aspergillus*- induced ROS production was elevated in neutrophils of IAPA patients compared to patients with severe influenza without IAPA and healthy controls.

**Conclusions:** Hyperinflammation and adaptive immune dysregulation are present in severe influenza. Specific immune markers which are elevated in IAPA patients could be explored as potential biomarkers.

## S13.3 Tracing Patterns of Evolution and Acquisition of Drug Resistant *Aspergillus fumigatus* Infection from the Environment Using Population Genomics

**Matthew Fisher**

**Objectives:** Infections caused by opportunistic fungal pathogens are increasingly resistant to first-line azole antifungal drugs. However, despite its clinical importance, little is known about the extent to which susceptible patients acquire infection from drug resistant genotypes in the environment. Our objective was to undertake a population genomic analysis of the mould *Aspergillus fumigatus* from across the United Kingdom and Ireland in order to trace patterns of evolution and acquisition of drug resistant *A. fumigatus* infection from the environment.

**Materials & Methods:** A total of 153 clinical *A. fumigatus* isolates from five participating centres were included. Environmental isolates (*n* = 65) were collected from the following sources: soil (72%), plant bulbs (12%), air (3%), compost (2%), and unknown (11%). All isolates were genome sequenced and analysed within a phylogenetic framework.

**Results:** We present a population genomic analysis showing with high confidence occurrences where *A. fumigatus* has evolved resistance in the environment prior to infecting patients. We find that the fungus is structured into two clades (‘A′ and ‘B′) with little interclade recombination and the majority of environmental azole resistance genetically clustered inside Clade A. Genome-scans show the impact of selective sweeps across multiple regions of the genome. These signatures of positive selection are seen in regions containing canonical genes encoding fungicide resistance in the ergosterol biosynthetic pathway, whilst other regions under selection have no known function. Phenotyping identifies genes in these regions that act as modifiers of resistance.

**Conclusions:** Our study supports the hypothesis that the widespread use of azoles as fungicides in agriculture is coupled to the increasing isolation of azole-resistant *A. fumigatus* from environmental sources. That these isolates bear hallmark multi-locus genotypes that are indistinguishable to those recovered from patients supports the conclusion that adaptation to fungicides in the environment is leading to acquisition of *A. fumigatus* bearing azole-resistance genotypes. Here, we identify spatially widespread clones of *A. fumigatus* that are not only resistant to azoles but are also highly represented in both the environment and the clinic, suggesting that that there are few fitness costs associated with this phenotype. Our research underscores the need to more fully understand the risk posed by environmental reservoirs of pathogenic fungi that, through the use of agricultural fungicides have evolved resistance to first-line clinical azoles.

## S13.4 EHA Guideline on Antifungal Prophylaxis in Adult Acute Myeloid Leukemia Treated with Novel Agents

**Jannik Stemler ^1,2,3^, Nick de Jonge ^4^, Nicole Skoetz ^5^, Roger Bruggemann ^6,7^, János Sinkó ^8^, Alessandro Busca ^9^, Zdenek Racil ^10^, Ronen Ben Ami ^11^, Vanessa Piechotta ^5^, Russell Lewis ^12^ and Oliver A. Cornely ^1,2,3,13^**

^1^ Department I for Internal Medicine, University Hospital of Cologne
^2^ Chair Translational Research, Cologne Excellence Cluster on Cellular Stress Responses in Aging-Associated Diseases (CECAD), University of Cologne
^3^ German Centre for Infection Research (DZIF), Partner Site Bonn-Cologne
^4^ Department of Haematology, Amsterdam UMC, Amsterdam
^5^ Cochrane Haematology, Department I for Internal Medicine, University Hospital Cologne
^6^ Department of Clinical Pharmacy, Radboud University Medical Center
^7^ Nijmegen Institute for Health Sciences, Radboud University Medical Center
^8^ Department of Hematology and HSCT, South-Pest Central Hospital National Institute of Hematology and Infectious Diseases
^9^ Dipartimento di Oncologia ed Ematologia SSCVD Trapianto allogenico di Cellule Staminali, A.O. Citta’ della Salute e della Scienza di Torino
^10^ Ustav Hematologie A Krevni Transfuze
^11^ Infectious Diseases Division, Infectious Diseases unit, Tel Aviv Sourasky Medical Center
^12^ Dipartimento di Scienze Mediche e Chirurgichea, Alma Mater Studiorum Università di Bologna
^13^ Clinical Trials Centre Cologne (ZKS Köln), University of Cologne, Faculty of Medicine and University Hospital Cologne

**Objectives:** To define evidence- or consensus-based recommendations for antifungal prophylaxis for AML patients on treatment with novel agents.

Novel therapeutic agents for acute myeloid leukemia (AML) have become available recently. Antifungal prophylaxis is generally recommended during induction remission chemotherapy, however, treatment settings for AML patients have become diverse and the risk-benefit ratio for antifungal prophylaxis in those settings is not well assessed in clinical trials. Due to cytochrome p450 metabolization and its inhibition by antifungal drugs, there is potential for drug-drug interactions (DDI) between novel AML agents and antifungals.

**Materials & Methods:** Experts from the European Hematology Association (EHA) Working Group Infections in Hematology and Cochrane Hematology Group develop an evidence and consensus-based guideline according to the GRADE methodology. The following PICO endpoints for each of the novel agents were formulated: occurrence of fungal infection, prolongation of hospitalization, days on intensive-care unit and mortality due to fungal infection, quality-of-life, and potential drug-drug interactions. Systematic literature review was performed. In three consensus-meetings, recommendations for each novel AML drug and specific setting were formulated.

**Results:** The following novel agents for treatment of AML were identified with not all of them being yet licensed for treatment: Hypomethylating agents (HMA; Azacytidine and decitabine), Midostaurin, Venetoclax (+HMA), Lestaurtinib, Gilteritinib, Sorafenib, Quizartinib, Ivosidenib, Enasidenib, Crenolanib, Glasdegib, Sapacitabine, Custatuzumab, Iomab B, Idanasutlin.

Evidence from the literature was generally scarce since fungal infections and prophylaxis were generally not assessed in randomized controlled trials of the respective AML drug.

Evidence-based recommendations were formulated for HMA, Midostaurin, and Venetoclax/HMA, for all other agents, consensus-based recommendations were given including the patient-specific setting of application of the novel agents (relapsed/refractory AML, single therapy or in combination with chemotherapy, induction treatment or maintenance etc.) into the decision process.

Antifungal prophylaxis is not recommended or moderately recommended in most settings, and strongly recommended if the novel AML agent is administered with intensive chemotherapy during induction treatment. Dose adaptations of some of the AML agents (midostaurin, venetoclax, quizartinib, sorafenib, gilteritinib) are moderately recommended with limited evidence if antifungal prophylaxis is administered with a strong CYP3A4 inhibitor due to expected increased exposure.

**Conclusions:** The first guideline document to help supporting the decision if to use antifungal prophylaxis in AML patients under treatment with novel agents will soon become available.

## S13.5 *Candida auris* Clinical Isolates from Patients with COVID-19 in Saint Petersburg, Russia: Identification, Antifungal Susceptibility

**Natalia Vasilyeva ^1^, Ellina Oganesyan ^1^, Irina Vybornova ^1^, Svetlana Gordeeva ^2^, Sergey Kovyrshin ^1^, Irina Moshkevich ^1^, Anastasia Taraskina ^1^, Ivan Pchelin ^1^, Galina Chilina ^1^ and Tatyana Bogomolova ^1^**

^1^ North-western State Medical University named after I.I. Mechnikov
^2^ Clinical Infectious Hospital named after S.P. Botkin

**Objectives:** to determine the spectrum of resistance to antifungal drugs of *C. auris* clinical strains isolated in St. Petersburg 2020–2021 from patients with COVID-19.

**Materials and methods:** A total of 78 strains of *C. auris* from patients (urine—50, blood—24, sputum—2, bronchoalveolar lavage—1, sinus punctate—1) hospitalized in 5 clinics in St. Petersburg were studied.

Identification was conducted by MALDI-TOF MS (Bruker Daltonic, Germany) and sequencing the internal transcribed spacer region (ITS) rDNA.

Antifungal susceptibility testing was performed using colorimetric panels Sensititre YeastOneYO10 (Thermo Fisher Scientific, Renfrew, UK), including: amphotericin B, voriconozole, itraconazole, posaconazole, fluconazole, anidulafungin, caspofungin, micafungin, 5-flucytosine. *Candida auris* isolates were cultivated in Sabouraud dextrose agar at 35 °C for 24 h and an inoculum at 1–5 × 10^5^ cells/mL was prepared in distilled water. For the interpretation of the results, tentative breakpoints recommended by the CDC (Center for the Control and Prevention of Infectious Diseases, 2020) were used.

**Results**. MIC values (μg/mL) were as follows: amphotericin B—0.12–2, voriconazole—0.06–8, itraconazole—0.12–16, posaconazole—0.06–8, fluconazole—32–256, anidulafungin—0.015–8, caspofungin—0.06–1, micafungin—0.06–1, 5-flucytosine—≤0.06–0.12.

**Conclusions**. *C. auris* strains isolated from patients with COVID-19 in St. Petersburg are characterized by the development of resistance in 100% of fluconazole and 15% were resistant to amphotericin B and fluconazole.

## S14.1 Fungal Respiratory Infections in Cystic Fibrosis

**Michaela Lackner**

**Objectives:** An overview will be given on fungal agents causing infections in cystic fibrosis patients. Chronically ill patients with several therapeutic cycles have a higher risk to face drug-resistant infections and therapeutic failure. Particularly azole-resistant fungi, represent an emerging problem. The aim is to provide and overview on intrinically azole-resistant fungi causing infections in CF-patients and fungi that are prone to aquire azole-resistance. Fungi acquire azole-resistance not only during therapy, but also in the enviornment. Azole-contaminated nieches will be highlighted and common azole-resistance meachisms will be discussed.

**Conclusions:** The talk will be concluded with strategies to reduce the developement and favouring of azole-resistant fungi and to improve and generate new therapeutic options.

## S13.6 Hyperendemic Zoonotic Sporotrichosis: Implementation of a Public Health Assistance Service for Human Sporotrichosis in Southern Brazil

**Vanice Poester ^1^, David A Stevens ^2^, Rossana Basso ^1,3^, Livia Munhoz ^1^, Jéssica Benelli ^1,3^, Gabriel Klafke ^1^ and Melissa Orzechowski Xavier ^1,3^**

^1^ Universidade Federal Do Rio Grande
^2^ California Institute for Medical Research
^3^ HU-FURG/EBSERH

**Objectives:** In Brazil, zoonotic sporotrichosis is a public health problem, with thousands of cases in the last decade in the country. An important way to reduce the cat-human transmission of *Sporothrix brasiliensis* is to spread information through health education activities, and promote better access for laboratory diagnosis. We report the implementation of a public specialized reference service (SRS) for diagnosis and treatment of sporotrichosis in southern Brazil.

**Methods:** The SRS was implemented in Rio Grande city (RG) (Rio Grande do Sul (RS), Brazil) at the University Hospital (HU-FURG-EBSERH). The impetus to implement a SRS for human sporotrichosis emerged from epidemiological data provided by the Mycology Laboratory (FAMED/FURG) in the last ten years, showing more than 600 cases of feline sporotrichosis, and 20 human cases (between 2012 and 2017). To study the impact of implementing SRS, the annual average number of cases, and the period between the appearance of lesions until diagnosis were compared, considering prior data and that post-implementation.

**Results:** The SRS was officially implemented in 2017 after meetings with RG health professionals to discuss logistics, and achieve involvement from as many stakeholders as possible. A referral flow was proposed (Figure 1). It includes clinical screening for sporotrichosis suspicious cases by professionals in RG primary health care (UBS), then referral to the SRS infectious disease service (HU-FURG/EBSERH) as the reference center for diagnosis and treatment of the disease. Since the SRS began, February 2017 to February 2021, 53 patients with suspected sporotrichosis were referred to HU-FURG/EBSERH. The diagnosis was confirmed in 83% of these (n = 44). Comparing the average of sporotrichosis cases in the pre-service implementation period (2012–2017), 3.25 cases/year, with the average of 11 cases/year post-implementation (2017–2021), this represented an increase of 275% in diagnosed cases. The interval from the beginning of lesions until diagnosis in the pre-service implementation period was a mean of 206 days (range 15 to 1095). This interval was reduced to an average of 79.5 days (range 5 to 540) after service implementation, representing a decrease of 235%. This allows an early therapeutic attack on the disease, at a more responsive stage. Of 33 basic UBS, 14 units referred patients to the SRS, representing 42% of the municipal health units.

**Conclusions:** In view of the hyperendemic situation of sporotrichosis, our report highlights, at a level of one municipality, the impact that an integrated service can have in confronting this problem. Similar measures and actions need also to be improved and established with regard to animal health, directly addressing the feline population to decrease fungal dissemination. Finally, these actions should be developed not only at the municipality level, but encompass states, regions, and all the national territory, aiming to contain the geographic expansion of sporotrichosis.

## S14.1 Fungal Respiratory Infections in Cystic Fibrosis

**Michaela Lackner**

**Objectives:** An overview will be given on fungal agents causing infections in cystic fibrosis patients. Chronically ill patients with several therapeutic cycles have a higher risk to face drug-resistant infections and therapeutic failure. Particularly azole-resistant fungi, represent an emerging problem. The aim is to provide and overview on intrinically azole-resistant fungi causing infections in CF-patients and fungi that are prone to aquire azole-resistance. Fungi acquire azole-resistance not only during therapy, but also in the enviornment. Azole-contaminated nieches will be highlighted and common azole-resistance meachisms will be discussed.

**Conclusions:** The talk will be concluded with strategies to reduce the developement and favouring of azole-resistant fungi and to improve and generate new therapeutic options.

## S14.3 Immunotherapy

**Darius Armstrong-James**

**Objectives:** To outline the current and emerging approaches to immunotherapy for respiratory fungal infections in cystic fibrosis.

**Materials & Methods:** Literature review and presentation of current data from ongoing Cystic Fibrosis Strategic Research Centre “Targeting Immunotherapy for Fungal Infection in Cystic Fibrosis”

**Results:** Current options for fungal immunotherapy include cytokine and chemiine therapies although these have not been systematically evaluated in the context of cystic fibrosis. The advent of a number of monoclonal antibodies for the treatment of asthma has opened up new possibilities in terms of managing cystic fibrosis-associated allergic bronchopulmonary aspergillosis. A number of novel anti-inflammatories have also been developed for the treatment of airway inflammation in cystic fibrosis. Furthermore, there is emerging data on the impoact of novel CFTR channel modulators on inflammatory responses to airway infection in cystic fibrosis.

**Conclusions:** The opportiunities for immunotherapeutic approaches to fungal infection in cystic firbosis are wide-ranging and fast moving. Precision immunophenotyping and systematic clinical studies are required to better define the utility of novel immunotherapeutic agents in this setting.

## S14.4 Genotypic and Phenotypic Portrait of *Candida albicans* Clinical Isolates Colonizing the Airways of Patients with Cystic Fibrosis

**Mayssa GNAIEN ^1^, Corinne MAUFRAIS ^2^, Yasmine REBAI ^1^, Wael MAMI ^1^, Aicha KALLEL ^3^, Fatma KHALSI ^4^, Samia HAMOUDA ^4^, Hanen SMAOUI ^4^, Monia KHEMIRI ^4^, Sondes HADJ FREDJ ^4^, Taieb MESSAOUD ^4^, Khadija BOUSSETTA ^4^, Kalthoum KALLEL ^3^, Christophe d’ENFERT ^2^, Helmi MARDASSI, Marie Elisabeth BOUGNOUX ^2^ and Sadri ZNAIDI ^1,2^**

^1^ Institut Pasteur de Tunis, LR16IPT01
^2^ Institut Pasteur, INRA, Unité Biologie et Pathogénicité Fongiques
^3^ La Rabta, Laboratoire de Parasitologie et de Mycologie
^4^ Hôpital d’Enfants Béchir Hamza de Tunis

**Introduction:** *Candida albicans* is a colonizer of Cystic Fibrosis (CF)-patient airways, competing with CF-associated pathogens such as *Pseudomonas aeruginosa* and *Staphylococcus aureus* and contributing to the severity of disease progression.

**Objectives:** The main goal of our study is to identify specific molecular patterns of chronic colonization in *C.albicans* serial isolates recovered from CF-patients. These molecular patterns will serve as the next biological targets implemented for novel pharmacological approaches related to CF-respiratory infections.

**Methods:** We serially recovered ~140 *C. albicans* clinical isolates over a period of 30 months from the expectorated sputa of 23 pediatric and 2 adult CF patients at the Children’s Hospital in Tunis, and characterized the genotype and phenotype of a subset of strains using multilocus sequence typing (MLST) and growth assays on multiple stress-, filamentous growth- and biofilm-inducing media.

**Results:** Out of 15 patients sampled during at least 9 months, 8 and 4 were chronically and transiently colonized with *C. albicans*, respectively. MLST analyses of 57 strains originating from 15 patients indicate that each patient is colonized with a single strain, while 6 of them carry isolates from clade 4; a clade known to be enriched with strains from Middle-East/Africa. A subset of these isolates with the same sequence type and colonizing 3 unrelated patients displayed altered susceptibility to cell wall-perturbing agents, suggesting changes in cell wall structure/function during growth in the CF-patient lung. We also observed differential ability to filament and to form biofilms in a set of identical isolates from clade 10 sampled over a period of 1 year in a pediatric patient, suggesting alterations in phenotypes associated with virulence.

**Conclusions:** MLST allowed us to investigate the population structure and the epidemiology of *C. albicans* clinical isolates in the contexte of chronic airways colnization within CF-patients. Besides genotypes data, phenotypic assays findings enabled us to select 30 *C. albicans* clinical isolates for whole-genome sequencing analyses in order to identify polymorphisms that could explain the emergence of new traits during chronic outgrowth of *C. albicans* in the CF-patient lung.

## S14.5 Exophiala Dermatitidis Infection in Cystic Fibrosis Accelerates Lung Function Decline; A Retrospective Single-Centre Review of Historical Lung Function

**Jonathan Ayling-Smith ^1,2^, Gemma Ford ^3^, Lorraine Speight ^1^, Rishi Dhillon ^4^, Matthijs Backx ^4^, P. Lewis White ^4^, Kerry Hood ^2^ and Jamie Duckers ^1^**

^1^ Cardiff and Vale University Health Board
^2^ Cardiff University
^3^ Cwm Taf Morgannwg University Health Board
^4^ Public Health Wales

**Objectives:** The impact of *Exophiala dermatitidis* on lung function in CF is unknown. The objective of this study was to assess if the growth of this microorganism impacts the lung function of patients with cystic fibrosis.

**Materials & Methods:** All patients in a single CF centre who had grown *E. dermatitidis* were compared with a random negative control group. A historical limit of 2007 or 1 year before transitioning to the adult CF team was used. Patients with less than 24 months of data in the control group and 48 months of data in the *E. dermatitidis* group were excluded. The sample size was 31 and control group size was 62. Patients were included up to transplantation, CFTR modulator use, moving out of area or death. The rate of decline in lung function was calculated as FEV1% change/year using the mean FEV1 from the earliest 12-month period on record and the mean FEV1 from the most recent 12-month period. In the *E. dermatitidis* group rates were calculated prior to the first recorded isolation of *E. dermatitidis* and subsequent.

**Results:** The mean rate of decline of the control group of −0.824%/year (S.D. 1.36) was not statistically different (*P* = 0.2) to the mean rate of decline in the *E. dermatitidis* group, prior to positive culture (−0.337%/year, S.D. 2.38). However, there was a significant difference (*P* < 0.01) in rate of decline between the *E. dermatitidis* group pre and post positive culture (−0.337%/year compared to −1.824%/year S.D. 2.35). This is worse than national average. The control and *E. dermatitidis* groups were not significantly different in CF genotype, pancreatic sufficient proportion and amount of time studied.

**Conclusions:** The results suggest that *E. dermatitidis* growth has a temporal relationship with poorer lung function. It is not clear if this is cause or effect. The sample size of this retrospective analysis is small in a single centre but the significance of this result warrants further research into the microbiota of patients with *E. dermatitidis*.

## S15.1 Cryptococcus qPCR Assays: The Future for Routine Mycology Labs and Clinical Trials Dealing with Cryptococcosis—An AMBITION Sub-Study

**Alexandre Alanio**

**Background:** Routine mycology from clinical trials on cryptococcosis is mainly based on testing CrAg in blood and cerebrospinal fluid (CSF), CSF observation in India ink and fungal culture using quantitative cryptococcal culture (QCC), which is labor intensive and difficult to manage in some settings.

We aimed at evaluating quantitative (qPCR) and reverse transcriptase quantitative PCR (RT-qPCR) assays to quantify the fungal load in the CSF, in blood and to identify *Cryptococcus* species responsible for the infection. We also aimed at understanding the dynamics of fungal DNA and RNA detection overtime during treatment to better understand the pathophysiology of the disease and to find interesting diagnostic and prognostic markers.

**Methods:** We developed three identification quantification qPCR assays based on the amplification of the unique nuclear *QSP1* target gene specific of serotype A, D and B/C and a 28S multi-copy qPCR assay to improve sensitivity for detection in plasma and blood. We developed also a reverse transcriptase qPCR based on *QSP1* whole nucleic acids (WNA) amplification for viability testing.

We advantageously evaluated our assays on patients recruited in the AMBITION-cm study in Gaborone (Botswana) and Blantyre (Malawi), 2 of the 7 sites of this phase III randomized clinical trial evaluating the efficacy of a single dose of liposomal amphotericin B with flucytosine and fluconazole as compared to the current WHO recommended treatment regimen. Patients were sampled at day 0, 7 and 14 for CSF (pellet and supernatant) and at H24, day 3 and day 7 for plasma and whole blood.

**Results:** From a total of 209 patients recruited in the study (85 from Botswana; 124 from Malawi), we were able to obtain material from 205 patients. From *QSP1* qPCR tested in CSF at D0, 169 (81%) were identified including 138 (81.7%) as serotype A, 28 (16.6%) as serotype B/C, 3 (1.8%) with mixture of A and B/C, 36 with no qPCR amplification. No difference in terms of fungal loads at D0, D7 and D14 in CSF was observed between serotype A and B/C with QCC, and qPCR assays.

QCC was compared to qPCR quantification showing a good correlation with *QSP1* qPCR (slope = 0.797, R^2^ = 0.73) and with 28S qPCR (Slope = 0.771, R^2^ = 0.778) assays.

The number of patients positive with QCC at D7 and D14 was significantly lower than with qPCR *QSP1* and 28S assays (*p* < 0.0001).

The early fungicidal assay (EFA) was higher with QCC than with qPCR as the median slope was lower with QCC than with *QSP1* and 28S assays. Slope between D0 and D7 was significantly lower than that of D7-D14 in QCC and in qPCR assays. D0-D7 slopes calculated with *QSP1* quantification were significantly lower in the single arm regimen than in the control arm. No significant difference in slopes were observed in patients alive or dead at week 10 (W10) in QCC and qPCR assays.

D0 CSF fungal loads of patients with negative QCC or qPCR at D7 was lower than that with negative QCC or qPCR at D14 or than the patients with persistent signal at D14 (*p* < 0.0001). Interestingly, the patients with persistent signal at D14, EFA correlated with the initial fungal load in QCC (R^2^ = 0.499), QSP1 (R^2^ = 0.67) and 28S (R^2^ = 0.61) assays.

The fungal load at D0 was significantly higher in patient who died at week 2 (W2) and at W10 as compared to patients who survived at W10 (*p* < 0.01), with no significant difference of initial fungal load in both treatment regimens (*p* > 0.05).

Detection of *Cryptococcus* DNA (28S qPCR) in plasma or blood within the first 24 h of treatment was significantly associated with early mortality at W2 and mortality at W10 (*p* < 0.01).

*QSP1* RT-qPCR showed that detection of DNA was due to viable fungal cells as the quantification of *QSP1* WNA was systematically higher (×2 to 5) than that of DNA.

**Conclusions:** Quantification of the CSF and detection in plasma at D0 will be identifying patients at risk of death that would potentially benefit from a different therapeutic management.

## S15.2 AMBITION Trial and Beyond

**Joe Jarvis**

*Cryptococcal Meningitis* (CM) is a leading cause of HIV-related mortality. Based on phase-II study data showing that a single high-dose of liposomal amphotericin-B (AmBisome, Gilead Sciences Inc) was non-inferior to 14 days of standard dosing in clearing *Cryptococcus* from the central nervous system we performed a phase-III randomised controlled non-inferiority trial to examine the impact of a single high-dose of AmBisome in averting all-cause mortality from CM (the AMBITION trial). HIV-positive adults with a first episode of CM in Botswana, Malawi, South Africa, Uganda and Zimbabwe were randomised to induction therapy of either (i) single, high-dose AmBisome (10 mg/kg) given with 14 days of flucytosine 100 mg/kg/day and fluconazole 1200 mg/day (AmBisome) or (ii) 7 daily doses of amphotericin B deoxycholate (1 mg/kg) plus 7 days of flucytosine 100 mg/kg/day, followed by 7 days of fluconazole 1200 mg/day (control). All participants received consolidation therapy of fluconazole 800 mg/day for eight weeks. The primary endpoint was all-cause mortality at ten weeks with the trial powered to show non-inferiority with a 10% margin. Single high-dose AmBisome on a backbone of flucytosine and fluconazole was non-inferior to the current WHO recommended standard of care for HIV-associated cryptococcal meningitis. This talk will outline the background and scientific rationale for the AMBITION trial, present the headline findings, and discuss the implications for clinical practice worldwide.

## S15.4 Modifications of the Cell Wall during Cryptococcosis

**Liliane Mukaremera**

Globally, meningitis caused by *Cryptococcus* causes 15% of all HIV/AIDS related deaths (~181,000 individuals per year). Even with the best available treatment, mortality rates due to HIV-associated *Cryptococcal Meningitis* range from 40% in high income to 70% in low income countries. Therefore, new strategies to improve outcome of *Cryptococcus* infection are urgently needed, and this depends on a deeper understanding of *Cryptococcus* pathogenesis.

Our current research focuses on understanding cell wall modifications during *Cryptococcus* infection. Mammalian cells lack a cell wall, making it an ideal target for new anti-*Cryptococcus* therapies. Currently, no drugs targeting the cell wall are used to treat *Cryptococcus* infection, not even Echinocandins, widely used to treat other fungal infections. These drugs showed early promise against *C. neoformans* in vitro, but are ineffective in vivo. This indicates that the structure of *C. neoformans* cell wall in vivo is different from that observed in vitro, and different from that of other common fungal pathogens. These observations led us to investigate how the *Cryptococcus* cell wall is modified during infection.

Using a laboratory strain of *Cryptococcus neoformans* we found that the cell wall is different in *Cryptococcus* cells grown under laboratory conditions and those formed during infection. In addition, within the in vivo cell population, cells with different sizes and morphologies possess different cell wall composition. Next, we examined the cell wall of *Cryptococcus* clinical isolates obtained from HIV-infected patients suffering from *Cryptococcus* meningitis. Similar to the laboratory strain, all clinical isolates tested presented cell subpopulations with different cell wall composition. In particular, cell wall chitin and chito-oligomer contents varied between cell subpopulations, and cell proportions in each subpopulation also differed between isolates.

*Cryptococcus* cell wall chitin has been associated with a detrimental immune response and worsening of the disease in the mouse model of cryptococcosis. Therefore, our observations that *Cryptococcus* clinical isolates possess cell subpopulations with differing cell wall might explain differences in immune responses, as well as disease progression observed among patients suffering from *Cryptococcus* meningitis.

## S15.5 Molecular Analysis of *Cryptococcus* spp. Reveals Species Diversity and Multilocus Sequence Typing Heterogeneity among People Living with HIV in Kinshasa

**Bive Zono ^1,8^, Rosalie SACHELI ^2,8^, Hippolyte SITUAKIBANZA ^3^, Pius KABUTUTU ^1^, Marcel MBULA ^3^, Gaultier MUENDELE ^4^, Raphaël BOREUX ^8^, Nicole LANDU ^5^, Celestin MUDOGO ^1^, Pierre-Robert M’BUZE ^6^, Michel MOUTSCHEN ^7^, Georges MVUMBI ^1^, Marie-Pierre HAYETTE ^2,8^**

^1^ Molecular Biology Service, Department of Basic Sciences, Faculty of Medicine, University of Kinshasa
^2^ National Reference Center for Mycosis, University Hospital of Liege
^3^ Infectious Diseases Service, Department of Internal Medicine/Department of Tropical Medicine, Faculty of Medicine, University of Kinshasa
^4^ Advanced HIV-Infection Management Unit, Internal Medicine Department, Centre Hospitalier Mère et Enfant de NGABA
^5^ Advanced HIV-Infection Management Unit, Internal Medicine Department, Centre Médical et Evangélique Révérend LUYINDU
^6^ Advanced HIV-Infection Management Unit, Internal Medicine Department, Centre Hospitalier Roi Baudouin 1er
^7^ Department of Infectious Diseases and General Internal Medicine, University Hospital Center of Liege
^8^ Center for Interdisciplinary Research on Medicines, University of Liège

**Objectives:** In the Democratic Republic of Congo (DRC), the neuromeningeal cryptococcosis (NMC) hospital prevalence varies between 8.8 and 11% with a death rate of about 50%. However, the *Cryptococcus* isolates circulating are poorly and formerly described. We describe the molecular characterization of *Cryptococcus* spp. isolated from people living with HIV (PLWHIV), and the association between NMC severity factors and the *Cryptococcus* multilocus sequence typing (MLST) profiles.

**Materials & Methods:** Cerebral spinal fluid (CSF) was collected from PLWHIV with neuromeningeal syndrome hospitalized in three Kinshasa public hospitals from 1 February 2019 to 29 February 2020, and cultured on Sabouraud dextrose agar + chloramphenicol medium afterwards. Serotyping PCR, internal transcribed spacer (ITS) sequencing, and MLST from next-generation sequencing (NGS) data were performed to genotype the *Cryptococcus* isolates. Raw NGS data were processed and the loci alleles were manually extracted using Geneious software. NMC severity factors such as hypoglycorrhachia (<50 mg/dL), raised CSF opening pressure (>30 cm water), and the patient pejorative outcome were compared to the *Cryptococcus* sequence type (ST)-MLST profile which was identified, using Pearson chi-square test or Fisher exact test.

**Results:** Out of 29 isolates included in the present study, 23 (79.3%; 95% IC: 65.5–93.1) have been identified as serotype A using serotyping PCR, while 6 (20.7%; 95% IC: 6.9–34.5) had unidentifiable profile. Moreover, all the 29 isolates have been characterized by ITS sequencing as follow: *Cryptococcus neoformans var. grubii* (23 of 29 isolates, 79.3%)*, Cryptococcus curvatus* (5 of 29, 17.2%), and *Cryptococcus laurentii* (1 of 29, 3.5%). Apart from this highlighted species diversity, *C. neoformans var. grubii* isolates were all identified as molecular type VNI using the MLST ISHAM scheme, with seven different STs including ST93 (14 out of 23, 60.9%), ST53 (1 out of 23, 4.3%), ST31 (1 out of 23, 4.3%), ST5 (1 out of 23, 4.3%), ST4 (1 out of 23, 4.3%), ST69 (1 out 23, 4.3%), and one novel ST not yet reported worldwide (2 out of 23, 8.6%). Furthermore, two isolates (8.6%) showed a mixed molecular profile (under investigation). Phylogenetic analysis carried out by the unweighted pair group method with arithmetic mean algorithm (UPGMA) of concatenated gene sequences from the seven MLST loci indicated that the main ST isolated in this study is very close to the only isolate previously reported from the DRC. The others are distant from it, even more so for ST69. All the STs identified in the present study have been isolated in the neighbouring countries of the DRC except for ST53, which has only been reported in Southeast Asia. Among NMC severity factors, only the patient pejorative outcome was associated with the minority STs infections (87.5%, *p* = 0.02) (ST53, ST31, ST5, ST4, STA, ST69).

**Conclusions:** Molecular analysis of *Cryptococcus* spp. isolates showed wide species diversity, and ST-MLST heterogenicity within the only one molecular type identified. The existence of the STs isolated in this study in the DRC neighbouring countries suggests probable close-to-home spread. Furthermore, infections due to minority STs were associated with a pejorative outcome than those due to ST93.

## S16.3 Managing Antifungal Tolerant Biofilms: Our Current Understanding and Future Directions

**Gordon Ramage**

Managing antifungal tolerant biofilms: our current understanding and future directions.

Fungal biofilms are notoriously difficult to manage due to their inherent tolerance to antifungal agents. These structures are physiologically distinct, surrounded by extrapolymeric matrix and have primed resilience to antifungal agents. This presentation will summarise our current understanding of fungal biofilm tolerance and describe some of the key in vitro and in vivo studies that have examined the impact of polyenes and other antifungal agents against these hard-to-treat infections. It will also examine the relevant studies that have demonstrated positive clinical management of biofilm related infections and discuss how polyenes and newly developed antifungal agents are being used in medicine.

## S16.5 Ibrexafungerp Is Effective in Treating Murine Mucormycosis Caused by Rhizopus Delemar

**Teklegiorgis Gebremariam ^1^, Sondus Alkhazraji ^1^, Yiyou Gu ^1^, Laura Najvar ^2,3^, Katyna Borroto-Esoda ^4^, Nathan Wiederhold ^2^, Thomas Patterson ^2,3^, Scott Filler ^1,5^ and Professor Of Medicine Ashraf Ibrahim ^1,5^**

^1^ The Lundquist Institute at Harbor-UCLA Medical Center
^2^ University of Texas Health Science Center at San Antonio
^3^ South Texas Veterans Health Care System, San Antonio
^4^ Scynexis
^5^ David Geffen School of Medicine

**Objectives:** Despite antifungal therapy and surgical debridement, overall mortality of invasive mucormycosis is >40%. Currently, the world is witnessing an explosion in mucormycosis in India among COVID-19 patients with >47,000 cases reported since May 2021. Thus, novel therapeutic modalities are needed. Ibrexafungerp (IBREXA) is the first member of a new class of triterpenoid antifungal agents that inhibit glucan synthase enzyme involved in the synthesis of the fungal cell wall polymer β-(1,3)-D-glucan. We sought to compare the activity of IBREXA alone and in combination with clinically used antifungal drugs against murine mucormycosis.

**Methods & Materials:** In vitro susceptibility was conducted by the CLSI M38 method using a clinical isolate of *Rhizopus delemar* (a common cause of mucormycosis). ICR mice were immunosuppressed with cyclophosphamide and cortisone acetate on days −2, +3, and +8, relative to infection with intratracheally instilled *R. delemar*. Treatment with placebo (diluent control), IBREXA (30 mg/kg, po, BID), liposomal amphotericin B (LAMB,10 mg/kg, iv, OD), posaconazole (POSA, 30 mg/kg, po, QD), a combination of IBREXA + LAMB, or a combination of IBREXA + POSA began 24 h post infection and continued for 7 days for IBREXA and POSA and 4 days for LAMB. Mice survival through Day +21 served as the primary endpoint.

**Results:** The minimum effective concentration (MEC) value for IBREXA against *R. delemar* 99–880 was >8 µg/mL, while the MIC for AMB and POSA at 100% inhibition of growth were 0.06 and 0.5 µg/mL, respectively. While all placebo mice (n = 20/arm, from two experiments with similar results) died by day 16 post infection, all treatment groups resulted in significant enhancement in median survival time and overall percent survival (Table 1) (*p* < 0.002 for any treatment vs. placebo). Furthermore, IBREXA + LAMB treatment resulted in significantly improved overall survival when compared to any monotherapy treatment (*p* < 0.04 vs. IBREXA, LAMB, or POSA). Finally, there was no significant difference in the overall survival between IBREXA + LAMB vs. IBREXA + POSA (*p* = 0.173), although median survival was longest with IBREXA + LAMB.

**Conclusions:** Despite no in vitro activity, IBREXA monotherapy demonstrated in vivo efficacy in treating *R. delemar* infection in immunosuppressed mice. This IBREXA activity was equivalent to antifungal drugs that are currently used for treating mucormycosis. Importantly, IBREXA demonstrated synergy when combined with LAMB against murine mucormycosis. Continued investigation of IBREXA as a novel antifungal agent against mucormycosis is warranted.


**Table: Median Survival Time**

**Median Survival (Day)**

**% Survival**
Placebo80IBREXA1535LAMB1330POSA1325IBREXA + LAMB>2165IBREXA + POSA1550

## S16.6 Lanosterol 14-α-demethylase-F5 Homologue Mediates Short-Tailed Azole Resistance in Mucormycetes: Characterization of *Mucor circinelloides* LDM-Genes in The Heterologous Model *S. Cerevisiae*

**Katharina Rosam ^1^, Brian C. Monk ^2^, Mikhail Keniya ^2^, Paul Schuchter ^1^ and Michalea Lackner ^1^**

^1^ Institute of Hygiene and Medical Microbiology, Medical University of Innsbruck
^2^ Sir John Walsh Research Institute and Department of Oral Biology, Faculty of Dentistry, University of Otago

**Objectives:** *Lichtheimia corymbifera, Rhizopus arrhizus, and Mucor circinelloides* are the most prevalent causative species of mucormycosis in Europe. Mucormycosis is an often lethal fungal infection, due to its fast progression and the limited treatment options available. Recommended first-line treatment is amphotericin B (AmB). Isavuconazole (IVU) and posaconazole (PSC) are recommended as salvage therapies. Short-tailed azoles such as voriconazole (VRC) are ineffective due to intrinsic resistance, which is suspected to be mediated via the amino acid (AA) changes Y129F and V123A, exclusively occurring in the LDM-F5 version of lanosterol 14-α-demethylase (LDM). We aim to generate a deeper understanding of the impact of AA changes in the ligand-binding pocket of the LDM on drug resistance to generate more effective azole drugs.

**Materials & Methods:** LDM homologue genes (*Mc*LDM-F1 and *Mc*LDM-F5) of *M. circinelloides* were overexpressed with and without their cognate cytochrome-P450-reductase (CPR) at *PDR5* and *PDR15 loci* in *Saccharomyces cerevisiae,* respectively. *S. cerevisiae* parental strains AD2Δ and AD2Δgal were used, both strains have seven ABC-transporters deleted and a pdr1-3 gain of function mutation in the Pdr3 transcriptional regulator. AD2Δgal parental strain has in addition a galactose-inducible native *ERG11*. To study the effect on drug binding within the ligand-binding pocket, AA changes were introduced in the LDM-F1 and reverted in the LDM-F5 gene (F129Y, A293V, F129Y & A293V), respectively.

Resistance testing was performed according to EUCAST guidelines for: VRC, fluconazole (FLC), IVU, PSC, itraconazole (ITC), and AmB, the fungicide triadimenol (TDM), plus the novel drug candidates (MCC7995, MCC8186). Minimal inhibitory concentrations (MIC) 90 and fold-changes in reference to the parental strains were determined. Resistance- and growth-phenotypes and growth kinetics were characterized. All transformants were genetically confirmed and protein expressions were verified using SDS-page and Western blots. Enzyme functionality and drug binding was studied using a novel enzyme kinetic assays.

**Results:** All mean MIC values are given in mg/L for AD2Δgal. Resistance phenotypes were as follows: highest resistance against short-tailed azoles was found for *Mc*LDM-F5-CPR (VRC: 4 and FLC: 128), followed by *Mc*LDM-F5 (VRC: 0.58 and FLC: 42.67) and *Mc*LDM-F1-CPR (VRC: 0.29 and FLC: 14.67). Isavuconazole responded similar to VRC, as it showed high MIC values for *Mc*LDM-F5-CPR (4). The novel compounds MCC7995 and MCC8186, as well as TDM had the same resistance characteristics as FLC. MICs of long-tailed azoles (PSC, ITC) and AmB were not affected when compared to AD2Δ.

The calculated generation times for AD2Δgal strains ranged from 154 to 187 min (min) in the exponential phase, whereby *Mc*LDM-F5 was the fittest strain (154 min) followed by *Mc*LDM-F1 (173 min), *Mc*LDM-F1-CPR (175 min), and *Mc*LDM-F5-CPR (187 min). Morphologically, single and co-expressing strains showed similar growth patterns.

**Conclusions:** To summarize, we constructed viable *S. cerevisiae* strains carrying *Mc*LDM variants with and without the cognate CPR. Co-expression of cognate reductase significantly impacted on short-tailed azole resistance, even though generation time was not shorter than in single expressing strains. In conclusion, these data strengthen the hypothesis that the AA changes in the McLDM-F5 confer resistance to short-tailed azoles.

## S17.3 Impact of Environmental Approaches for Allergen Avoidance and Asthma Control

**Jean-Pierre Gangneux**

Patients with asthma are commonly exposed to multiple indoor allergens that may contribute to the increased asthma-related complications in this population. Preventing strategies are still questioned, among them pharmacological approaches but also air quality improvement leading to healthy home environments. The analysis of the literature shows contrasting results but methodologies greatly varies. Global allergen avoidance strategy with an individualized, home-based, comprehensive environmental intervention with an Indoor Environment Counsellor (IEC) is the most promising method. We will review the impacts of such method from the environmental side to clinical improvement.

## S17.4 Type 2-High Asthma Patients Reveal a Specific Indoor Mycobiome and Microbiome

**Louise-Eva Vandenborght ^2^, Raphaël Enaud ^1^, Charlotte Urien ^2^, Noémie Coron ^1^, Pierre-Olivier Girodet ^1^, Stéphani Ferreira ^2^, Patrick Berger and Laurence Delhaes ^1^**

^1^ CHU de Bordeaux, Inserm U1045, Université de Bordeaux
^2^ GenoScreen, Research and Development, Microbiota Team

**Background and Objectives:** Currently, the relation between microbial exposome and asthma is well-admitted, but combining NGS results from pulmonary and indoor microbiomes (or microbial exposome) of asthmatic patients with spirometry, clinical and endotypes parameters is a new research field. In this context, our objectives were to investigate the links between indoor microbial exposome and pulmonary microbial communities (exogenous, endogenous mycobiome and microbiome), and to document the exposome role onto inflammatory and clinical outcomes of patients with severe asthma (SA).

**Materials & Methods:** On the whole, 55 SA patients from the national COBRA cohort were submitted to an indoor microbial exposome analysis trough Electrostatic Dust Collectors (EDC). Among them, 22 patients were able to produce sputa during stable or pulmonary exacerbation periods. A set of 22 pairs of EDC and sputum were collected during the study period and analyzed using targeted metagenomic in order to compare microbial communities from all EDC and sputum samples of patients, according to type 2 (T2)-asthma endotypes.

**Results:** Compared to patients with T2-low SA, patients with T2-high SA exhibited an a decrease of fungal α-diversity of their indoor microbial exposome, significantly correlated to FeNO levels. In addition, the β-diversity of EDC mycobiome clustered significantly according to T2 endotypes. Moreover, the proportion of fungal taxa in common between sputum and EDC samples was significantly higher when patients exhibited an acute exacerbation compared to patients with stable pulmonary status.

**Conclusions:** These results illustrated for the first time a potential association between indoor mycobiome and clinical features of SA patients, renewing the interest in deciphering the interactions between indoor environment, fungi, and host in asthma.

## S17.5 Antifungal Therapeutic Outcomes in Allergic Broncho Pulmonary *Aspergillosis* (ABPA) Patients with Asthma

**Lisa Nwankwo ^1^, Desmond Gilmartin ^2^, Sheila Matharu ^2^, Anand Shah ^4^ and Darius Armstrong-James ^3,4,5^**

^1^ Pharmacy, Royal Brompton Hospital, Guy’s and St. Thomas’ NHS Foundation Trust
^2^ Clinical informatics, Royal Brompton Hospital, Guy’s and St. Thomas’ NHS Foundation Trust
^3^ MRC Centre for Molecular Bacteriology and Infection, Department of Infectious Diseases, Imperial College
^4^ Department of Respiratory Medicine, Royal Brompton Hospital, Guy’s and St. Thomas’ NHS Foundation Trust
^5^ MRC Centre of Global Infectious Disease Analysis, Department of Infectious Disease Epidemiology, School of Public Health

**Background:** The human host defence system determines the clinical presentation of infection due to *Aspergillus fumigatus*. In patients with underlying respiratory disease, it can cause pulmonary aspergillosis infections such as ABPA (allergic bronchopulmonary aspergillosis). Outcomes with antifungal treatment in patients with ABPA on a background of asthma have not been extensively studied in real world settings.

**Objectives:** The aim of this study was to characterize the clinical spectrum of therapeutic outcomes in patients with ABPA on a background of asthma, as a strategy for optimizing disease interventions. Description of real world treatment response in this patient group may help future focused research and for identification of patient subgroups that may benefit from intensive management and patient follow up to prevent disease progression.

**Materials & Methods:** This was a retrospective single-centre cohort study evaluating longitudinal outcomes in non-cystic fibrosis (CF) ABPA patients with underlying chronic lung disease (asthma), and description of treatment approaches. Statistical Analysis System (SAS) programming was used for data integration from multiple hospital electronic systems, for patient identification and data analysis. Criteria for inclusion were patients with a background of asthma, serum Total IgE >1000 IU/mL and a positive *Aspergillus* IgE, with or without a positive culture for *A. fumigatus* from a respiratory sample. Data collected included demographics, lung function severity, disease activity serological markers (*Aspergillus* IgE, *Aspergillus IgG*, Total IgE) and triazole levels. In this study the impact of fungal therapy on disease outcomes was assessed.

**Results:** SAS analysis identified 452 patients with asthma complicated by ABPA from Oct 2009 to March 2021 (11 years 6 months). Univariate and bivariate analysis were applied to understand the relationships between lung function, drug levels and *Aspergillus* serology over time. Trough triazole drug levels correlated inversely with *Aspergillus* IgE levels for posaconazole and voriconazole, (though *p* values were not statistically significant); as drug level increased into a therapeutic range, a corresponding decrease in *Aspergillus* IgE was observed. Forced expiratory volume (FEV) % predicted in 1 s improved in patients treated with antifungals. The highest improvement was seen with posaconazole (*r_s_* = 0.27, *p* = 0.1591) (Figure 1) but the impact of isavuconazole treatment on lung function could not be evaluated due to insufficient data. Though lung function improved on itraconazole therapy (Figure 1), and total IgE reduction occurred (Figure 2), it did not correlate with a reduction in *Aspergillus*-specific IgE, even when levels were in therapeutic range (*r_S_* = 0.11, *p* < 0.0001). For posaconazole, voriconazole and isavuconazole, both *Aspergillus* IgE and Total IgE reduction were observed (Figure 2). Of all the triazoles used in this cohort, a median sub-therapeutic level of 0.82 mg/L was experienced with voriconazole, with median levels for the other antifungals being therapeutic (Itraconazole—0.76 mg/L, posaconazole—1.39 mg/L, isavuconazole—3.29 mg/L, *p* < 0.0001, Kruskal-Wallis statistic).

**Conclusions:** Antifungal therapeutic outcomes and influencers of this in patients with ABPA on a background of asthma are complex and multi-factorial. Longitudinal monitoring in these patients and adjusting treatment accordingly may improve outcomes, but further analysis is needed, and consideration of the interaction of other non-fungal therapies should be explored.

## S18.2 Diagnosis of Invasive Aspergillosis: What Is New?

**Juergen Prattes**

Invasive aspergillosis remains a devastating disease in critically ill and immunocompromised patients. Early and reliable diagnosis represents a cornerstone for successful management of aspergillosis. In this presentation, new insights in already established diagnostic procedures as well as new diagnostic tools will be discussed. A special focus will be given to new point-of-care diagnostic tools, which enable rapid and easy to perform assessment of suspected aspergillosis.

## S18.3 PCR Diagnosis

**Lewis White**

Public Health Wales Mycology Reference laboratory, PHW Microbiology Cardiff, UHW, Cardiff, UK

PCR to aid in the diagnosis of aspergillosis has been used for over two decades, with use generally limited to specialist centres, restricted by limited standardization, the lack of commercial assays and external quality assurance schemes that prevented incorporation into international consensus definitions for classifying invasive fungal disease. However, many developments have arisen over the last decade making *Aspergillus* PCR comparable with other biomarker assays used for the diagnosis of aspergillosis.

The efforts of the Fungal PCR initiative have standardized molecular methods used to test a range of specimens, external quality assurance schemes for *Aspergillus* PCR are available from Quality Control for Molecular diagnostics and a range of commercial *Aspergillus* PCR assays are available. These developments have lead to the incorporation of *Aspergillus* PCR in to international definitions for classifying invasive aspergillosis and permits the incorporation of *Aspergillus* PCR methods in any centre with access to a general molecular diagnostics laboratory.

Molecular diagnostics remain the only non-culture assay with the capacity to identify potential resistance and data pertaining to a range of commercial *Aspergillus* PCR assays with the capacity to identify the most common mutations associated with azole resistance in *Aspergillus fumigatus*, in the absence of culture, is increasingly available. Given the expanding range of both clinically and environmentally driven mutations associated with azole resistance in *A. fumigatus*, methods such as pyrosequencing are being used to identify mutations direct from the sample.

A range of novel molecular tests are being developed with the capacity to enhance the molecular diagnosis of aspergillosis. Proximity ligation assays combine the specificity of antibody testing with the sensitivity of molecular testing, droplet digital PCR provides enhanced quantification and detection of limited DNA concentrations, while the use of meta-genomic sequencing can detect free DNA from a wide array of pathogens circulating in the plasma of patients.

A recent Cochrane review and meta-analysis of *Aspergillus* PCR testing of blood samples confirmed performance but opposite to GM-EIA testing showed that prior antifungal therapy impacted specificity but not sensitivity.

## S18.4 Urine Aspergillus Antigen Detection as an Aid to Diagnose Invasive Aspergillosis

**Kausik Datta ^1,2^, Robina Aerts ^3^, Johan Maertens ^3^, Nitipong Permpalung ^1^, Donald Sheppard ^4^ and Kieren Marr ^1,2^**

^1^ Johns Hopkins University School of Medicine
^2^ MycoMed Technologies LLC
^3^ Hematology Department, University Hospitals Leuven
^4^ McGill University

**Objective:** We previously reported the use of novel antibodies recognizing galactofuranose-containing glycans in a lateral flow assay as an aid to diagnose invasive aspergillosis (IA) using urine. Johns Hopkins and MycoMed Technologies have now optimized MycoMEIA™, a diagnostic enzyme immunoassay.

**Methods:** Validation studies were performed using samples obtained from JHU, AsTeC multicenter repository, and University Hospitals Leuven, Belgium. Diagnoses were adjudicated by reviewers blinded to test results. IA was graded as proven, probable, or possible IA, using consensus definitions. MycoMEIA assays were performed in batch, blinded to clinical diagnosis, and results were interpreted as sample index values relative to a threshold control, with index ≥1 considered positive.

**Results:** 710 specimens from 267 people were tested. Index values ranged from 0.236 to 24.366, corresponding to urinary antigen concentrations ranging from 8 ng/mL (analytical antigen estimation limit) to 180 ng/mL antigen equivalent. Numerical analysis of cohorts with confirmed or suspected IA excluded people with airway disease. In the validation subset that included urine specimens from people with proven or probable IA, collected with receipt of minimal (<3 days) antifungal therapy, MycoMEIA sensitivity was 95.2% (95% CI: 76.2–99.9) and specificity was 95.6% (95% CI: 91.2–98.2). Receiver operator characteristic area under the curve was 0.992 (95% CI: 0.98–1). In the larger cohort of subjects with minimal antifungal therapy, 4/4 (100%) people with proven IA and 27/31 (87%) people with probable IA had positive MycoMEIA assays, with a per case sensitivity of 88.6% (95% CI: 73.3–96.8) and specificity of 90% (95% CI: 80.5–95.9). In the overall cohort, specimens tested without consideration of prior antifungal receipt yielded positive assay results in 4/6 (67%) people with proven IA and 28/45 (62.2%) people with probable IA, lowering the sensitivity to 62.7% (95% CI: 48.1–75.9). MycoMEIA results were semi-quantitatively distributed according to certainty of invasive disease, with positive tests in 46/97 (47.4%) who had clinical signs and symptoms without confirmation using current diagnostics.

**Conclusions:** The MycoMEIA assay is a sensitive and specific diagnostic aide for IA. Since antigen expression may decrease with antifungal therapy, the test may be best employed with early signs of IA and/or for screening in people with high risks.

## S18.5 Proteomic Analysis of Humoral Immune Components in Bronchoalveolar Lavage of Patients Infected or Colonized by *Aspergillus fumigatus*

**Sarah Dellière ^1,2^, Magalie Duchateau ^3^, Sarah Wong ^2^, Quentin Giai Gianetto ^3^, Hélène Guegan ^4^, Mariette Matondo ^3^, Jean-Pierre Gangneux ^4^ and Vishukumar Aimanianda ^2^**

^1^ Laboratoire de Mycologie Médicale, Hôpital Saint-Louis
^2^ Molecular Mycology Unit, UMR2000, Institut Pasteur
^3^ Plateforme de Protéomique, Institut Pasteur
^4^ Inserm, EHESP, CHU de Rennes, Université de Rennes

**Objectives:** The role of humoral immunity against fungal pathogens is a disregarded research niche in medical mycology. While recent studies have indicated their specific as well as crucial roles against several fungal pathogens, a global view of the multitude/complex nature of humoral immune components is needed to bring new insight into host-fungal interaction. In this direction, we undertook a comparative proteomic analysis of the broncho-alveolar lavage (BAL) collected from individuals infected or colonized by *Aspergillus fumigatus* (an airborne fungal pathogen) vs. controls, to identify those humoral components affected upon this fungal infection.

**Methods:** Two BAL-pools, one each from patients infected or colonized by *A. fumigatus* (*Aspergillus* + BAL) and controls (n = 10 in each pool) were subjected separately to in-solution trypsin digestion and analyzed on a Q Exactive Plus mass-spectrometer coupled with an EASY-nLC1200 chromatography system. The raw data was searched with MaxQuant against Uniprot *Homo sapiens* reference proteome. Functional annotation was performed using DAVID and protein-protein interactions were studied using STRING database.

**Results:** A total of 1177 proteins were identified in the two BAL-pools, among which 181 proteins were less abundant and 337 proteins were completely absent in *Aspergillus*+ BAL compared to controls. Gene-Ontology analysis of these less abundant/absent proteins (518 in total) indicated that those highly overrepresented proteins were from innate immune functional categories, and mostly related to activation of the complement system (*p* = 7.6 × 10^−4^). C1q, mannan binding lectin serine peptidase 2 (MASP2), ficolin 2 (FCN2), surfactant protein D (SP-D), complement factor H related proteins 2 and 5, complement 8 and C-type lectin receptor CD206 were among completely absent proteins in the *Aspergillus* + BAL. In contrast, a total of 435 were more abundant in *Aspergillus*+ BAL, among which 111 proteins were only identified in *Aspergillus*+ BAL including ficolin-1 (FCN1) and interleukin-8 (IL-8). Pentraxin-3 was found significantly increased in *Aspergillus*+ BAL. Protein-protein interaction analysis further highlighted potential crosstalk between these humoral immune components.

**Conclusions:** While some of the humoral components found altered in *Aspergillus*+ BAL in our proteomic analysis, such as SP-D and FCN2, have been studied in host-*A. fumigatus* interaction, the roles of other components have yet to be studied. The absence or less abundance of humoral components in *Aspergillus* + BAL may be due to their consumption, degradation, or inhibition of their biosynthesis, while overproduction may be the reason for their increased abundance. These new leads and hypotheses require further investigation to understand the interplay between humoral immune system of host and *A. fumigatus*. Moreover, humoral components that were completely absent or only present in *A. fumigatus* infected or colonized BAL could be potential immunodiagnostic markers of *A. fumigatus* infection.

## S18.6 Aspergillus Lateral Flow Assay with Digital Reader for the Diagnosis of COVID-19 Associated Pulmonary Aspergillosis (CAPA): A Multicenter Study

**Brice Autier ^1^, Juergen Prattes ^2^, P Lewis White ^3^, Maricela Valerio ^4^, Marina Machado ^4^, Jessica Price ^3^, Matthias Egger ^2^, Jean-Pierre Gangneux ^1^ and Martin Hoenigl ^2,5,6^**

^1^ Inserm, EHESP, Irset (Institut de Recherche en Santé, Environnement et Travail), UMR_S 1085, CHU Rennes, Univ Rennes
^2^ Division of Infectious Diseases, Medical University of Graz
^3^ Public Health Wales Mycology Reference Laboratory, UHW
^4^ Hospital General Universitario Gregorio Marañón
^5^ Division of Infectious Diseases and Global Public Health, University of California San Diego
^6^ Clinical and Translational Fungal-Working Group, University of California San Diego

**Objectives:** The *Aspergillus* LFA is a rapid diagnostic test that has been recently introduced into the market to supplement some weaknesses of *Aspergillus* galactomannan immunoassay. This multicenter study evaluated the performance of the IMMY *Aspergillus* Galactomannan Lateral Flow Assay (GM-LFA) with automated cube reader for diagnosis of pulmonary aspergillosis in patients with COVID-19 (CAPA) associated acute respiratory failure requiring intensive care unit (ICU) admission.

**Materials & Methods:** A total of 198 respiratory samples and 149 serum samples from 243 patients were retrospectively tested with the IMMY sona *Aspergillus* Galactomannan LFA (IMMY, Norman, Oklahoma, USA). GM immunoassay (Platelia *Aspergillus* Ag ELISA; Bio-Rad Laboratories, Marnes-la-Coquette, France) was routinely and prospectively performed in the majority of samples. Participating centers were the University Hospital of Rennes (France), the University Hospital of Graz (Austria), the Public Health Wales Mycology Reference Laboratory (Cardiff, United-Kingdom), and the Hospital General Universitario Gregorio Marañón of Madrid (Spain). Patients were admitted to ICU between March 2020 and April 2021, and retrospectively classified for CAPA status according to the European Confederation of Medical Mycology (ECMM)/International Society for Human and Animal Mycoses (ISHAM) criteria, with the exclusion of *Aspergillus* LFA as mycological criterion (Koehler et al., Lancet Infect Dis 2020).

**Results:** At the 1.0 optical density (ODI) cutoff, sensitivity of GM-LFA was 68%, 83% and 42%, and specificity was 80%, 84% and 98% for tracheal aspiration (TA), non-directed bronchioalveolar lavage (NBL), and bronchioalveolar lavage (BAL), respectively. When combining all respiratory samples, area under the curve (AUC) for receiver operating characteristic (ROC) curve was 0.809 and 0.724, 0.887 and 0.723 for isolated TA, NBL and BAL, respectively. When testing serum using a 0.5 ODI cutoff, sensitivity and specificity of GM-LFA was 14% and 93%, respectively. Spearman correlation analysis showed a significant correlation between serum LFA-ODI and serum GM-ODI (rho 0.323, *p* = 0.001). For 8/85 patients without CAPA, serum GM-LFA result was between 0.5 and 0.67 and would have misclassify them as “probable CAPA” using the recommended 0.5 cutoff value.

**Conclusions:** Overall, the *Aspergillus* GM-LFA showed good performance for CAPA diagnosis, especially on respiratory samples at the 1.0 cutoff. As some false positive results can occur for serum samples, isolated results slightly above the recommended cutoff should lead to further mycological investigations to increase the specificity.

## S19.1 Antifungal Tolerance: Spanning the Genetic and Phenotypic Diversity of *Candida albicans*

**Judy Berman**

Mortality from invasive fungal diseases approaches 50%, despite the use of available antifungal drug. The rare appearance of antifungal drug resistance (e.g., <2% of *C. albicans* isolates are drug resistant) cannot explain these treatment failures. Antifungal tolerance is a poorly understood property that is expressed to different degrees in different susceptible (non-resistant) isolates, yet is not measured routinely in the clinic or in most research studies. We are studying how tolerance differs between isolates, what biological mechanisms drive it and how it affects only some cells within a single isolate. We also are interested in how to inhibit it so as to improve treatment outcomes. The cell biological properties of genetic and non-genetic contributions to *C. albicans* antifungal tolerance along with a large scale ‘OMICS approach that we have embarked upon to address the mechanistic issues at the species, isolate and single-cell levels.

## S19.3 BET Bromodomain Inhibition as a Potential New Antifungal Therapeutic Strategy

**Jérôme Govin**

**Objectives:** Opportunistic fungal infections are a major cause of morbidity and mortality in hospitalized immunocompromised individuals. The emergence of drug resistance in many strains and the toxicity and high cost of the limited repertoire of available drugs indicates that there is an urgent need for novel therapeutic agents.

Bromodomain and Extra-Terminal (BET) proteins are chromatin-associated factors that regulate gene transcription and chromatin organization. BET proteins recognize chromatin through their two bromodomains (BDs). BET BDs are readily druggable and efforts by both academic groups and biopharmaceutical companies have led to the discovery of several potent and selective human BET BD inhibitors, which are currently under clinical evaluation for the treatment of various cancers and other non-infectious diseases.

**Materials & Methods:** Our research program combines yeast genetics/epigenetics, biochemistry, structural biology, medicinal chemistry and medical mycology It involves a consortium of 4 laboratories: J. Govin, C. Petosa & M. Cornet (Grenoble, France); C. McKenna (USC, Los Angeles, CA, USA).

**Results:** Our data showed that the BET protein Bdf1 is essential in *Candida albicans* & C. glabrata and that mutations inactivating its two BDs result in a loss of viability in vitro and decreased virulence in mice. Furthermore, we identified small-molecule compounds that inhibit Bdf1 BDs with high selectivity over human BDs.

**Conclusions:** These findings establish BET inhibition as a promising antifungal therapeutic strategy and identify Bdf1 as an antifungal drug target.

## S19.4 Use of Animal Infection Models in Preclinical Antifungal Development

**David Andes**

Animal infection models in the pharmacokinetic/pharmacodynamic (PK/PD) evaluation of antimicrobial therapy serve an important role in preclinical assessments of new antifungals. The assays are utilized for dosing optimization in clinical trial design and for preliminary guidance in in setting susceptibility breakpoints. The goal of animal model studies is to both mimic the infectious disease process in humans to allow for robust PK/PD studies to find the optimal drug exposures that lead to therapeutic success. The PK/PD index and target drug exposures obtained in validated animal infection models are critical components in optimizing dosing regimen design in order to maximize efficacy while minimize the cost and duration of clinical trials. The output from use of models of invasive candidiasis and aspergillosis have correlated well with patient outcome. Emerging fungal pathogen models are similarly providing useful proof of principle to guide and support progression from preclinical to clinical investigation. This presentation will review the design and application of animal infection model use as a component of preclinical drug development.

## S19.5 Development of the Arabian Killifish (*Aphanius dispar*) as a Model Host to Study Human Fungal Pathogens

**Atayaf Hamied ^1,2^, Larissa John ^1^, Tina Bedekovic ^1^, Alex Brand ^1^, Tetsu Kudoh ^1^ and Mark Ramsdale ^1^**

^1^ University of Exeter
^2^ University of Baghdad

**Objectives:** Studies using zebrafish embryos have developed our knowledge of the immune response to human fungal pathogens and assisted in the development of antifungal drug screens. However, zebrafish embryos are not viable at human body temperatures (normal or pyrexic), which has a significant impact on these findings as a high temperature is crucial for the full virulence of many pathogenic fungi. In this study we have developed Arabian Killifish embryos, *Aphanius dispar* (originating from the warm waters of the Arabian and Persian Gulfs) as an alternative to zebrafish embryos, so that the pathogenesis of *Candida albicans* infections can be followed at physiologically relevant temperatures (30–40 °C).

**Materials & Methods:** Arabian killifish embryos were injected with *C. albicans* yeast cells (wild-type and mutant strains) and the progress of infection studied using CFU, direct imaging and embryo survival. Nanopore PromethION sequencing of the *A. dispar* genome allowed the monitoring of host immune function markers via Western blots and qPCR. Host immune functions were knocked down with targeted morpholinos. Infection progress has been monitored in the presence of fluconazole (0–32 µg/mL). A transgenic DARK fish line (*gch*-knockout) has been created using CRISPR-Cas9 technology to facilitate live-cell imaging of infection progression.

**Results:** Using this model in the context of *C. albicans* infection, we have shown dose dependent survival, tracked the spread of infection in live embryos, observed the interaction of *C. albicans* cells (yeast and hyphae) with immune cells, and shown that infections can be effectively treated with antifungal drugs. Genome sequencing revealed the presence of many genes of interest for further study related to immune function. Morpholino knockdown of *irf8* and *cxcr4* (required for macrophage differentiation and migration, respectively) strongly decreased embryo survival following challenge with *C. albicans*. The successful deletion of the *gch* gene using CRISPR-Cas9 to make DARK embryos demonstrates that transgenic killifish lines can be generated for the study of host responses to fungal infection.

**Conclusions:** Arabian killifish embryos can support *C. albicans* infections at 30–40 °C and the interaction with host immune cells can be monitored in real-time. In addition to the clear benefits of studying infections at physiologically relevant temperatures, the yolk of the Arabian killifish is clearer than in zebrafish helping to improve imaging, somite movement is delayed compared to zebrafish (reducing the requirement for tricaine which could interfere with host functions), and the duration of the embryonic stage is 12 d (*c.f* 9 d in zebrafish) enhancing the temporal resolution of host responses. Overall the Arabian Killifish model has many advantages over zebrafish and could make a significant contribution to our understanding of fungal pathogenesis.

## S20.1 From Trials to Routine Care—Strengthening Pathways to Effective Implementation—Lessons from Cryptococcal Meningitis and the UNITAID/CHAI Programme for Advanced HIV Disease

**Angela Loyse**

According to the latest UNAIDS 680,000 [480,000–1.0 million] people died from AIDS-related illnesses in 2020. Many of these deaths in resource limited settings (RLS) are preventable. Changes in routine care practices have lagged significantly behind advances in diagnostics and treatments demonstrated in clinical trials in the last decade. To reduce deaths and prevent morbidity there needs to be increased attention and resources allocated to effective and sustainable implementation interventions and strategies tailored to routine care RLS.

This talk will focus on the methodology and preliminary findings of the DREAMM implementation science project. The DREAMM project aimed to reduce mortality from HIV-related central nervous system (CNS) infections within routine care services and prospectively describe their epidemiology in Tanzania, Malawi and Cameroon. The project is divided into 3 key phases: (1) Observation, (2) Training, and (3) Implementation. The main DREAMM intervention is the implementation of an algorithm for the diagnosis and treatment of HIV-related CNS infection. Key features of the algorithm are bedside rapid diagnostic testing, alongside routine laboratory sample processing, and the use of WHO-recommended treatment strategies for cryptococcal meningitis, the leading cause of HIV-related meningo-encephalitis in Sub-Saharan Africa. The two main DREAMM implementation strategies are: (1) Empowerment of local leadership, and, (2) Improvements in the delivery of quality of hospital care in RLS. The delivery of quality care was improved using the following 3 tools: (1) Health system engineering, (2) A freely accessibly co-designed education program for laboratory technicians and frontline healthcare workers (HCWs), and (3) Joint laboratory and clinical communities of practise. Preliminary data suggest a substantial reduction in mortality following the implementation of the DREAMM intervention and implementation strategies.

Key advances and gaps in ending often preventable and unacceptably high deaths due to HIV-related CNS infection in RLS will be summarised. Programmatic advances in *Cryptococcal Meningitis* and advanced HIV disease (AHD) in African low-and middle-income countries (LMICs) more broadly will be outlined. Preliminary data from the flucytosine (5-FC) access program in South Africa will be presented. In addition, the Unitaid/CHAI program on AHD is rolling out commodities for AHD, including tests and medicines for cryptococcal meningitis, in 8 African LMICs and India. Significant implementation gaps remain however, including in access to CD4 testing, alongside gaps in demand creation, and R&D for *Cryptococcal Meningitis* and AHD.

## S20.3 Improving Outcomes for Patients with Sporotrichosis, Chromoblastomycosis and Mycetoma in South America

**Flavio Queiroz-Telles**

**Introduction:** The Implantation or subcutaneous mycoses (IM) include a heterogeneous group of fungal diseases that develop at the site of transcutaneous trauma. These diseases are a frequent health problem in Latin American countries and other tropical and subtropical areas of the world. Although these infections rarely cause disseminated or invasive disease, they have an important impact on public health, may be difficult to control, and often recur. According to their estimative burden, sporotrichosis, chromoblastomycosis (CBM) and mycetoma are the most relevant IM in South America (SA).

**Sporotrichosis** is the most prevalent and globally distributed of the IM. It is caused by several *Sporothrix species*, a genus of thermally dimorphic fungi that infect humans and animals. This disease usually develops after inoculation with contaminated organic material or via zoonotic transmission. Sapronotic transmission by plants, occurs around the globe, however, an emerging species, *S. brasiliensis*, is transmitted to humans and dogs by cats directly from the yeast form, and causing the largest sporotrichosis outbreak ever recorded. Cat transmitted sporotrichosis (CTS), is estimated to involve about 5000 humans, 8000 cats and 300 dogs and is expanding through into neighboring SA countries. The main issue of CTS is the early diagnosis suspicion. Microbiological confirmation may be time consuming (culture) and direct exam and/or histopathology have low sensitivity (<20%). Immunological or molecular tests are not commercially available in SA. The preliminary results of a rapid test (SLAF) using blended *Sporothrix* spp. antigens in 298 sera samples of patients with proved/probable CTS and 200 controls, showed 82.6% of sensitivity and 81.5% of with 95% CI of 73.69% to 89.56% and 75.41% to 86.63%, respectively When validated, SLAF may help early diagnosis and therapy of CTS, avoiding the increasing of morbity and reducing treatment duration.

**Chromoblastomycosis:** CBM is the second most prevalent implantation mycosis in the world, caused by several melanized fungi, almost all members of the Herpotrichiellaceae family, widely found in nature. CBM is mainly an occupational disease and prevalent in agriculturists. The estimative global burden of CBM is nearly 10,000 cases/year. In SA, in a 106 years period, 2619 cases were reported, mostly caused by *Fonsecaea pedrosoi*. Although the diagnosis of CBM does not rely on expensive and sophisticated laboratory tools, the disease remains neglected by all health systems, making the time of diagnosis too long (mean of 9 years). This aspect certainly impacts in morbidity, including disease progression, risk of superinfection and malignant transformation. The best therapeutic method for small initial lesions is surgical excision. Diagnosis is often delayed and systemic antifungal therapy is usually required. Long-term therapy with itraconazole is the best option for inoperable lesions. The response rate to itraconazole ranges from 15% to 80%, depending on the causative agent and severity of the disease. As this disease is caused by several types of transcutaneous trauma, the use of protective equipment such as gloves, shoes, and adequate clothes may reduce the risk of infection.

**Mycetoma:** Mycetoma is a chronic, progressive, implantation inflammatory disease characterised by severe and stigmatising deformities, disability, and high morbidity. The disease is caused by fungi (eumycetoma) and several Actinomycetales members (actinomycetoma). Its estimative global burden is 9000 cases. There are no robust epidemiological studies on mycetoma in SA, but it is tconsidered the third most prevalent endemic IM in SA, especially among low socioeconomic adult males living in rural areas from Argentina, Brazil, Colombia and Venezuela, where the principal agents are *Madurella* spp. and *S. apiospermum*. The diagnosis of eumycetoma relies on clinical suspicion, radiological histopathology and microbiology exams. Therapy for eumycetoma consists of prolonged antifungal therapy with Itraconazole or posaconazole. Cure rate is 25–35%.

## S20.4 Mycology Expereince from a Cancer Hospital in Pakistan

**Summiya Nizamuddin and Azra Parveen**

Shaukat Khanum Memorial Cancer Hospital and Research Center

**Objectives:** The incidence of invasive fungal infections (IFIs) in immunocompromised cancer patients has continued to increase over the last few decades and IFIs have become a leading cause of morbidity and mortality in this group of patients. The morbidity and the mortality of IFIs remain high while the diagnosis and treatment of IFIs are highly challenging, especially for a developing country. Being a large tertiary care cancer hospital in Pakistan, we encounter a variety of fungal infections in our patients. We report our experience of fungal infections in cancer patients from the last 2 years.

**Materials & Methods:** The retrospective study was conducted at Shaukat Khanum Memorial Cancer Hospital and Research Centre, Lahore, Pakistan, and comprised data related to all cancer patients with a reported positive fungal culture between January 2018 and April 2021.

**Results:** A total of 332 non-duplicate cases of invasive fungal infections were identified. 54% were caused by *Aspergillus* species, 41% by yeasts, 3% by mucorales and 2% by other moulds. Out of all the yeast infections, 70% were associated with candidemia and 30% by non-candidemia infections. 34% of all candidemias was caused by *C. albicans* where 66% were caused by non-*albicans Candida*. Those suffering from solid tumors developed candidemias more compared to those suffering from hematological maliagnancies. Males suffered more with candidemias compared to females. The age group of 40–60 years developed candidemias the most followed by the 1–10 years age group. 3 infections caused by the rare yeasts, *C. duobushaemulonii* and *C. auris* were also identified.

Out of all the IFIs caused by *Aspergillus* species, 78% were caused by *A. flavus* at 78%, *A. fumigatus* at 14% and *A. terreus* at 7%. Solid tumor patients and males suffered more in these infections as well. 2 infections caused by Madurella species were also identified in the infections caused by other moulds.

**Conclusions:** The a variety of pathogens, ranging from yeasts to to invasive molds were ecountered in this analysis. The bulk of IFIs in our cancer patients is caused by *Aspergillus* species followed by yeasts. Non-*albicans Candida* are more likely to be the cause of candidemia. With increasing reports of resistant fungal patheogens, continued and long term survillance of fungal disease epidemiology is required.

## S20.5 Laboratory Diagnosis of Invasive Fungal Infections in a Low-Resource Setting: A Five Year Retrospective Study

**Rebecca Peters, Adobi Dike and Rita Oladele**

Lagos University Teaching Hospital

**Objectives:** Serious fungal infections can be life threatening. Prompt laboratory diagnosis is critical in effective management of these infections. Nigeria with a population of >200 million people is estimated to have an 11.8% burden of fungal infections annually. This study examines laboratory data on invasive fungal infections (IFI’s) generated over a five-year period in the mycology unit of a tertiary health institution in Nigeria.

**Materials & Methods:** Data of clinical specimens/samples received for fungal studies between January 2016 and December 2020 were retrieved from the laboratory records of the Medical Microbiology and Parasitology laboratory, Lagos University Teaching Hospital (a 760 bed facility with a 10 bed ICU and HDU capacity). Samples were received from various clinics in the hospital including ICU/HDU, ENT, Neurosurgery, Maxillofacial and Pediatrics department. Suspected cases of superficial infections were excluded. Microscopic examination was carried out using 10% KOH, India ink and Giemsa staining. All samples were cultured on Sabouraud Dextrose Agar and 10% brain heart infusion agar supplemented with chloramphenicol and Gentamicin. Blood samples were cultured in Bactec (BD)/BactAlert blood culture systems. Yeast isolates were subjected to Germ tube test while encapsulated yeasts were tested for urease production. Mould isolates were identified using conventional fungal identification methods. Antifungal susceptibility testing was however not done.

**Results:** In the period under review, a total of two hundred and sixty five (265) specimens/samples were received from patients suspected to have invasive fungal infection. The age range of the patients was 2 months–84 years. Majority (226; 85.3%) was between ages 18–84 years. Male: female ratio was 1:1.7. Sputum 47 (17.7%) followed by nasal swabs 39 (14.7%), blood 26 (9.8%) and CSF 23 (8.8%) were the predominant samples; the least samples were urine 2 (0.8%), intracranial mass 5 (1.9%) and maxillofacial mass 5 (1.9%). A total of 80 (30.2%) fungal isolates were recovered from culture with species distribution as shown below. *Candida* spp. 50 (62.5%) accounted for majority of the isolates with non-*C. albicans* 35 (70%). This was followed by *Aspergillus* spp. 16 (20%) with *A. flavus* 10 (62.5%) predominant.

**Conclusions:** This study highlights the gaps in our laboratory diagnostic capacity. Low samples level probably due to lack of suspicion of fungal IFIs on part of clinicians in our setting. There is need to drive awareness amongst clinicians and inclusion of antifungal susceptibility testing and therapeutic drug level monitoring in our spectrum of services.

Pattern of types of samples sent to the laboratory for fungi studies 2016–2020.


**Specimen Type**

**Frequency (%)**
Vitreous fluid7(2.6)Corneal scrapping10(3.8)Ear swab24(9.1)Nasal swab39(14.7)Nasal polyps15(5.7)Sinonasal mass16(6.0)Intercranial mass5(1.9)Maxillofacial tissue5(1.9)Oral swab4(1.5)Urine2(0.8)CSF23(8.8)Blood26(9.8)Pleural14(5.3)Sputum47(17.7)Soft tissue biopsy21(7.9)

Distribution of isolated cultured and identified.

## S21.4 NEOGLUCAN Study: Serial (1–3) β D Glucan Levels in High Risk Neonates

**Laura Ferreras-Antolin ^1,2^, Nasreen Aziz ^3^ and Adilia Warris ^2,4^**

^1^ Paediatric Infectious Diseases Research Group, Infection and Immunity, St. George’s University of London
^2^ MRC Centre for Medical Mycology, University of Exeter
^3^ Neonatal Unit, St. George’s University Hospital NHS Foundation Trust
^4^ Paediatric Infectious Diseases, Great Ormond Street Hospital

**Objectives:** Neoglucan aimed to characterise the baseline values and the dynamics of serum (1,3)-β-D-glucan (BDG) in neonates at high risk of neonatal invasive candidiasis (NIC); as well as to determine the effect of various clinical variables on these levels.

**Methods:** Single centre prospective cohort study aiming to include 20 neonates. Neonates were included if they were prematurely born with a gestational age <29 weeks and/or if they presented with a birth weight ≤1000 gr. Blood samples for BDG assessment were obtained twice weekly for a period of 6 weeks. BDG concentration were measured using the Fungitell® assay.

**Results:** Nineteen neonates were enrolled. The median age was 10 days (IQR 6–15) with a median gestational age of 25 weeks (IQR 24–27) and a median birth weight of 730 gr (IQR 650–810). Eight neonates were girls (42.1%). There were no positive blood cultures for *Candida* spp. and none of the neonates was diagnosed with NIC. Only one neonate was found colonised with *C. parapsilosis*. A total of 190 serum samples were included in the study. The median BDG value was 59 pg/mL (IQR 30–148), whereas the mean was 119 pg/mL (SD ± 154). A total of 42.1% (80/190) samples showed values 80 pg/mL, with all the neonates presenting at least one test above this cut-off. Twenty-two percent of the samples showed values 160 pg/mL; 18/19 neonates had at least one value above this level. Neonatal age did not show linear association with BDG levels; regression coefficient was 0.45, *t*-test 0.6 (*p* = 0.547). The BDG levels were stratified by presence of factors which potentially can influence BDG test results. Only the exposure to steroids and the use of a heel prick as sampling method were associated with statistically significant differences in BDG levels. Neonates on steroids showed higher BDG levels compared to those not treated; median 100 pg/mL (IQR 53–225) vs. 53.5 pg/mL (28–143) (*p* < 0.05). Samples taken by heel prick presented a median BDG of 66.5 pg/mL (32–151), compared to those obtained by venepuncture or CVC, median was 22.5 pg/mL (13–49) (*p* < 0.01). Abdominal surgery or abdominal pathology, the administration of parenteral nutrition, blood product transfusion or exposure to antimicrobials did not show effect to BDG levels.

**Conclusions:** The BDG levels in serial neonatal blood samples showed high variability. A significant proportion of samples (42.1%) presented values above the threshold for positivity (e.g., ≥80 pg/mL) in the absence of NIC. The exposure to postnatal steroids and the heel prick as the method of blood sampling were associated with higher BDG levels. The current BDG threshold for positivity needs to be reconsidered to allow its use in neonates at high risk for NIC.

## S21.5 Optimization of Fluconazole Therapy for the Treatment of Invasive Candidiasis in Preterm Infants

**Aline G.J. Engbers ^1,2^, Robert B. Flint ^2,3^, Swantje Völler ^1,4^, Irwin K.M. Reiss ^2^, Jan-Willem Alffenaar ^6^, Dick Tibboel ^2^, Sinno H.P. Simons ^2^, Catherijne A.J. Knibbe ^1,7^ and Roger Brüggemann ^8,9^**

^1^ Department of Systems Biomedicine & Pharmacology, LACDR, Leiden University
^2^ Department of Paediatrics, Division of Neonatology, Erasmus UMC—Sophia Children’s Hospital
^3^ Department of Hospital Pharmacy, Erasmus University Medical Center
^4^ Division of BioTherapeutics, LACDR, Leiden University
^5^ Department of Neonatology, Radboud University Medical Center
^6^ Department of Clinical Pharmacy and Pharmacology, University Medical Center Groningen
^7^ Department of Cinical Pharmacy, St. Antonius Hospital
^8^ Department of Pharmacy and Radboudumc Institute of Health Sciences, Radboud University Medical Center
^9^ Radboudumc Center for Infectious Diseases and Center of Expertise in Mycology Radboudumc/CWZ, Radboud University Medical Center

**Objectives:** Fluconazole is the first-choice drug for treatment of invasive candidiasis in preterm infants, that affects 4–8% of intans with a birthweight below 1000 g [1]. Due to the poor outcome of invasive candidiasis timely and adequate treatment is essential, but currently there is no licensed dosing regimen for treatment with fluconazole in preterm infants. Preterm infants are therefore often treated as term infants, for which a maintenance dose of 6 to 12 mg/kg every 72 h if postnatal age is below 14 days, every 48 h if postnatal age is 15–28 days and daily once postnatal age is above 28 days. In this study we performed a population pharmacokinetic study on fluconazole in preterm infants to evaluate currently used dosing strategies and to develop a therapeutic regimen for this cohort.

**Materials & Methods:** Fluconazole concentrations obtained from preterm infants from two studies were pooled and analysed using NONMEM V.7.3. Patients were treated therapeutically or prophylactically. Dosing was at the discretion of the treating physician. Samples were obtained for therapeutic drug monitoring or opportunistically, aiming for 4 samples. A population pharmacokinetic model was developed based on objective function value and numerical and graphical performance. Postnatal age, gestational age, postmenstrual age, birthweight, actual bodyweight, gender, being small for gestational age and serum creatinine concentrations were examined as covariates and included in the model if they decreased the objective function value with 6.6 points minimally (*p* < 0.01) and decreased interindividual variability.The developed model was used to evaluate current dosing practice. A therapeutic dosing strategy aiming to reach a minimum target exposure of 400 and 200 mg·h/L per 24 h for fluconazole-susceptible *C. albicans* meningitis and other systemic infections, respectively, was developed.

**Results:** In 41 preterm neonates with median (range) gestational age 25.3 (24.0–35.1) weeks and median postnatal age at treatment initiation 1.4 (0.2–32.5) days, 146 plasma samples were collected. A one-compartment model described the data best, with an estimated clearance of 0.0147 L/h for a typical infant of 0.87 kg with a serum creatinine concentration of 60 µmol/L and volume of distribution of 0.844 L. Clearance of fluconazole was found to increase with 16% per 100 g increase in actual bodyweight, and to decrease with 12% per 10 µmol/L increase in creatinine concentration once postnatal age was above 1 week (Figure 1). To maintain target attainment throughout the treatment period we suggest to administer fluconazole daily, independent from postnatal age. Additionally, we suggest dose adjustments based on serum creatinine and postnatal age to achieve 400 or 200 mg·h/L in all preterm infants (Figure 2).

**Conclusions:** In preterm infants, fluconazole clearance is best predicted by actual bodyweight and serum creatinine concentrations. To account for these effects fluconazole dosing should not only be based on bodyweight but also on creatinine concentration to achieve optimal exposure in all infants.

**References**

1. Turner, K. et al. *Curr. Med. Chem.* **2012**, *19*, 4617–4620.

## Q02 Recurrent Vulvovaginal Candidiasis (RVVC)—A Diagnostic Challenge

**Riina Rautemaa-Richardson**

Recurrent vulvovaginal candidiasis (RVVC) is a debilitating, chronic condition which affects over 138 million (6%) women of reproductive age annually. It is a hormonally and immunologically driven condition triggered by Candida spp. However, Candida is also a common coloniser of the vulva and vagina. The symptoms of VVC are non-specific and shared with a number of infectious and non-infectious conditions. Therefore, the diagnosis of vulvovaginal candidiasis has to build on the combination of symptoms and clinical and laboratory findings, not culture results alone.

The following questions will be discussed in this session: How do you diagnose RVVC? What are the cornerstones of RVVC management? How do you manage patients with poor or partial response to treatment? What is the evidence for the benefit of alternative treatments? What alternative or additional diagnosis need to be considered?

## Q03 Clinical Mycology—How to Get Things Going in LMICs

**Rita Oladela ^1,2,3^**

^1^ Department of Medical Microbiology & Parasitology, College of Medicine University of Lagos, Idi araba, Lagos, Nigeria
^2^ Medical Mycology Society of Nigeria
^3^ Africa Mycology Working Group

**Background:** Currently, more than five times more people live in low- and middle-income countries (LMICs) than in high-income countries. The covid-19 pandemic and its fallout have laid bare deep-seated social and economic inequalities with marginalised groups being at greater risk of infection and being disproportionately affected by containment measures and their socioeconomic consequences. Most LMICs are burdened with marked by social and health inequalities, with poorly funded and overburdened health systems. GAFFI has estimated the burden of disease in a number of LMICs. An estimated 47 million Africans suffer from fungal diseases, of which an estimated 1.7 million suffer from a serious fungal infection annually. Additionally, a high prevalence of HIV in Sub-Saharan Africa contributes to a high burden of opportunistic fungal infections. Furthermore, approximately 50% of fungal related deaths in the setting of HIV infections occur in Africa.

Challenges: Fungal infection remains a neglected field, and diagnostic facilities and access to treatments are limited across most of these countries.

The diversity of clinical manifestations of invasive fungal diseases represents a diagnostic challenge in many scenarios.

Most LMICs lack a surveillance system for fungal infections despite a number of them being endemic for some life-threatening mycoses.

An important capacity limitation in clinical laboratories of LMICs is identification of antimicrobial resistant organisms as well as other pathogens to species level. This affects the surveillance of infections and antimicrobial resistance.

There is a pervasive picture of inadequate/poor diagnostic capacity for fungal infection (mostly due to lack of infrastructure and skilled personnel).

There is low level of awareness among health care workers and policy makers, with apathy on part of government.

Unavailability and non-accessibility of antifungal drugs is well documented.

The recent revolution in non-culture-based diagnostics is yet to penetrate most LMICs, with the exception of the cryptococcal antigen testing.

Way forward.

United efforts are mandatory to face the growing challenges in medical mycology—GAFFI, ISHAM, ECMM and CDC already started but more needs to be done.

Input from African CDC is crucial.

Individual country government ‘buy-in” with investments in clinical mycology and diagnostic resources should be advocated for.

Education and training of healthcare providers as well as educating the general public to change the attitudes of people is an another aspect that needs to be concentrate on. Intensive awareness programs for clinicians will drive clinical index of suspicion.

Capacity building including the human resources as well as the laboratory capacity in hospitals.

Medical school curriculum revision to capture mycoses adequately.

Some fungi cannot be routinely grown under lab conditions and culturing is time consuming and requires specialist training; also equipment need electricity, which at best is erratic in most LMICs. Thus, there is a need for diagnostics that can be widely applied by laboratory technicians lacking traditional fungal identification skills and facilities. This must be affordable.

Barriers to accessing antifungal drugs must be addressed, GAFFI contribution to this is noted but more needs to be done; prices and toxicities must be addressed alongside with availability of alternative medications/treatment options.

Surveillance of serious mycoses is essential to prevent and control infections, to detect outbreaks and also to see the effects of interventions.

Establishing Medical Mycology Societies have been demonstrated to drive awareness.

The role of telemedicine in areas that lack mycologists.

Funding research to address epidemiology and knowledge gaps.

## Q05 β-D-Glucan in Paediatrics

**Laura Ferreras-Antolin**

β-D-glucan (BDG) is a cell wall component of many pathogenic fungi. The detection of BDG as an assay is clinically broadly used and it has been included as mycological criterion in the revised definitions of IFD from the EORTC/MSG consensus group. However, the current data on BDG in paediatrics is limited prompting specific considerations when BDG is used in children. In this session we aim to discuss the current evidence surrounding BDG use in paediatrics.

## Q06 Teaching Medical Mycology

**Yang Dong-Hoon and June Kwon-chung**

National Institutes of Allergy and Infectious Diseases, NIH, Bethesda, Maryland, USA

*Cryptococcus gattii* species complex as an opportunistic pathogen: Underlying medical conditions associated with infection.

*Cryptococcus gattii* species complex has been considered a primary pathogen due to its high infection frequency among apparently immunocompetent patients. In order to scrutinize the pathogen status, we analyzed cryptococcal isolates and patient histories from 135 global *C. gattii* cases in which 86 were diagnosed as immunocompetent (though some had other medical issues) while 49 were diagnosed as immunocompromised with underlying conditions similar to those seen in *C. neoformans* infection. Plasma from 32 of the 86 seemingly immunocompetent patients were obtained to analyze for the presence of GM-CSF autoantibodies. 76% of the patients with no underlying health issues showed presence of GM-CSF autoantibodies, suggesting that the GM-CSF autoantibody is the major hidden risk for the infection. No relationship between the *C. gattii* lineages and underlying conditions was found, except for over representation of VGIV from HIV+ patients due to the prevalence of VGIV in Africa. Patients with *C. gattii* infections warrant detailed evaluation for unrecognized immunologic risk.

## Q06 Teaching Medical Mycology

**Esther Segal**

Medical Mycology is part of Medical Microbiology; the latter being considered a prerequisite of the curriculum for students in Medicine at the preclinical study stage. This in-turn will be the basis for Infectious Diseases, to be taught at the stage of clinical studies, including clerkships at rounds at the patient’s-bed.

Thus, different approaches may be taken for teaching Medical Mycology, such as detailed exhaustive studies during the initial preclinical study- stage or minimal studies at this stage and more in- depth and broader content during the clinical- study period.

In this Session the focus will be on the Teaching of Medical Mycology at the preclinical study stage.

Teaching Medical Mycology is focusing on presenting in form of lectures and in practical laboratory sessions the major:

- Morphological and physiological characteristics, the ecology, as well as the virulence-attributes of the fungi involved in human-diseases, and the major antifungal- groups in use.
- Lectures will also include:
  ✓ the epidemiological aspects of host-pathogen interactions
  ✓ the laboratory diagnosis, including laboratory test-demonstrations
  ✓ the principles of treatment
  ✓ The major groups of fungi associated with human-diseases are covered, including:
    ▪ The Dermatophytes and Dermatophytoses
    ▪ Mallassezia infections
    ▪ Candida and candidiasis
    ▪ Cryptococcus and cryptococcosis
    ▪ Aspergillus and aspergillosis
    ▪ Mucorales and mucormycosis
    ▪ The Dimorphic fungi—emphasis on Histoplasmosis and Coccididomycosis
    ▪ Pneumocystis.

Each group will be illustrated by a specific clinical-case description before the detailed characteristics are discussed.

We believe that this approach provides a sound basis of knowledge and understanding to the students for their later education in the clinical study-stage.

## Q09 Antifungal Therapeutic Drug Monitoring (TDM) for Optimal Dosing and Monitoring Strategies: The Recent Updates

**Chin Fen Neoh**

Invasive fungal diseases (IFD) are associated with high mortality and morbidity in particular among the immunocompromised and critically ill patients. Managing patients with IFD remains challenging. Inadequate antifungal drug exposure at the site(s) of infection may contribute to treatment failure while toxicity occurs if the drug concentration exceeds normal therapeutic range. There is an increasing emphasis on the utility of antifungal therapeutic drug monitoring (TDM) in optimising the management of invasive fungal diseases (IFD) over the last decade. Application of TDM is common for itraconazole, voriconazole, posaconazole and flucytosine that with considerable inter- and intra-individual variability in pharmacokinetics profiles or with greater tendency of drug-drug interactions. This presentation aims to provide the recent updates on the recommendations for the need for TDM of each antifungal agent, including the strategies for dose adjustment. The role of TDM for those antifungals without routine recommendations for TDM and the role of clinical pharmacogenetics implementation for CYP2C19 prior to initiation of voriconazole therapy will be briefly discussed as well.

## Q10 Radiology

**Joanne Cleverley**

In this question and answer session the imaging of fungal infection will be discussed. The session will emphasize the role of different imaging modalities, radiology terminology and the spectrum of imaging findings to suggest a diagnosis of fungal infection.

## Q12 Mycological Diagnosis on the Bedside: Where Are We in 2021 Cryptococcosis

**John Perfect**

This presentation is based around a “meet the expert” format. Initially, there will be a short discussion which examines the tools for diagnosis from cultures, antigens, PCR and histopathology. I will describe the tests and utilization with both their values and their deficits. I will touch on the simple, cheap lateral flow assays (LFA) and how they are being used in pre-emptive strategies in high-risk patients in highly endemic areas for cryptococcosis. I will also discuss some new research studies examining host blood cell transcriptome signatures to identify cryptococcosis. It is important to emphasize that our biggest deficiency in cryptococcosis is the prolonged time from symptoms to diagnosis which can lead to a poor prognosis. Furthermore, I will discuss how to follow treatment success with quantitative CSF yeast counts, repeated CSF antigens or PCR. One of the hardest clinical diagnostic needs in cryptococcal meningoencephalitis is to separate relapse/persistence infection from development of immune reconstitution inflammatory syndrome (IRIS). It is at this interface that one must use assessment of all tools including cultures, biomarkers, radiographs and timing of symptoms to make critical decisions in management strategies.

After a discussion of these issues, there will be several illustrative cases to describe principles of cryptococcal diagnosis. Finally, the discussion will be opened up to the attendees for questions directed to expert and if necessary, specific selected questions from the presenter will be asked and answered.

Cryptococcosis remains a worldwide infection as the most common fungal CNS infection in medicine. It is deadly and its risk groups are enlarging. New antifungals for cryptococcal disease are still several years away; therefore, to reduce the 15–30% mortality, it will be important that we utilize effectively our diagnostic platform.

## Q14 Diagnosis from Tissue: Histology and Accurate Identification—Is it Possible?

**Nathan Wiederhold**

The diagnosis and appropriate treatment of invasive fungal infections depends upon accurate identification of pathogens by pathologists and clinical microbiologists. Histopathologic and cytopathologic studies are useful in providing diagnostic insight in patients with suspected fungal infections. Such examinations can offer provisional identifications of fungal organisms, which can help guide initial therapy while laboratory results are pending. Common etiologic agents of invasive mycoses may be recognized based on morphologic characteristics observed in tissue and biologic fluids, such as those obtained from bronchoalveolar lavage and bronchial washings. However, care should be taken in the interpretation of these findings, as there may be a false sense of the ability to correctly categorize fungal organisms to the genus or species level by morphologic features alone. Studies have demonstrated discordant results between histopathology/cytology studies and laboratory results due to overlapping morphologic features, morphologic mimics, and sampling errors. Thus, histopathology/cytology findings should provide a differential of potential fungal pathogens and be combined with results from laboratory studies, including cultures, antigen tests, serology, and/or molecular assays, in order to improve accuracy in the identification of etiologic agents of fungal infections. Inaccurate identification of the infecting organism can lead to inappropriate antifungal therapy and possibly poor clinical outcomes.

## Q14 Diagnosis from Tissue: Histology and Identification

**Raquel Sabino ^1,2^**

^1^ Department of Infectious Diseases, Reference Unit for Parasitic and Fungal Infections, Instituto Nacional de Saúde Dr. Ricardo Jorge, Lisbon, Portugal
^2^ Instituto de Saúde Ambiental, Faculdade de Medicina, Universidade de Lisboa, Lisbon, Portugal

Histopathology continues to be a rapid and cost-effective means of providing a presumptive or definitive diagnosis of invasive fungal infections.

Tissue samples from patients with suspected invasive fungal disease (IFD) should be examined not only by mycological culture but also by microscopy and specific fungal stains should be included. For histopathological diagnosis of fungal infection is required deep knowledge about fungal morphology in tissue and also about the various reactions of the tissue in response to that infection. Highly experienced histopathologists are therefore essential to detect fungal structures and also to recognize tissue reactions associated with IFD, distinguishing them from staining artifacts.

Histopathology of specimens obtained from an affected site, showing a positive result by the presence of distinctive structures associated to specific endemic fungal species, is considered as criteria for proven IFD. On the other hand, observation of yeasts may sometimes point out for a specific IFD but without confidence. Examination of filamentous fungi in histopathological sections can provide important information like the presence of septa, the hyphal diameter or branching angle and melanisation and may thus yield valuable diagnostic clues on the nature of the causative agent. However, a reliable identification of the fungal species based solely on morphological criteria in histopathology is usually impossible. Species identification of fungi detected in histopathological sections should be attempted using immunohistochemical and/or molecular tools. The recently revised EORTC/MSG criteria for diagnosis of IFD from tissue specimens recommends the amplification of fungal DNA by PCR combined by DNA sequencing only when fungal elements are seen by histopathology.

During this presentation we will discuss with the audience, in an interactive mode, the advantages and pitfalls of tissue diagnosis, and which tests that can be performed to a more targeted diagnosis.

## Q16 Fungal Outbreaks

**Ilan Schwartz and Alida Fe Talento**

Fungi are ubiquitous in natural and built environments, and the ability to cause serious, opportunistic infections in persons with medical or surgical breaches to innate immune defences make hospital associated infections particularly challenging for infection prevention and control programs. Hospital outbreaks, defined as an increased number of cases linked by time and place, due to fungi can involves yeasts (unicellular fungi) or moulds (multicellular fungi). With regards to yeasts, outbreaks due to *Candida* species like *C. parapsilosis* and more recently *C. auris* have been increasing worldwide. Hospital outbreaks of cryptococcosis and pneumocystosis in immunocompromised patients are particularly troubling. Similarly, outbreaks due to moulds such as *Aspergillus* and *Mucorales* are well recognised. This session will focus on the challenges of fungal outbreaks in the healthcare setting. These challenges are fourfold. First is the detection of fungal outbreaks due to the difficulty of early and accurate diagnosis of fungal infections. Second is source identification given that fungi are widely dispersed in the environment. Third is the global emergence of antifungal resistance in a backdrop of limited effective therapeutic options. Fourth is the paucity of data on effective infection control measures to manage these outbreaks. Systems to prevent outbreaks in the healthcare setting is key and the role of new technologies such as whole genome sequencing in the management of fungal outbreaks will be discussed.

## Q17 Antifungal Stewardship

**Souha Kanj ^1^ and Samir Agrawal ^2^**

^1^ American University of Beirut Medical Center, Department of Internal Medicine, Division of Infectious Diseases
^2^ Senior Lecturer and Honorary Consultant, Division of Haemato-Oncology, St. Bartholomew’s Hospital, Barts Health NHS Trust; and Blizard Institute, Queen Mary University of London

Invasive fungal infections (IFI) remain a challenge to the treating physician because of difficulty in diagnosis and high rates of morbidity and mortality associated with these infections, which occur predominantly in the immunosuppressed and critically ill patients. The incidence of invasive fungal infections, especially candidiasis, aspergillosis, and mucormycosis, continues to rise. With the excessive use of azole antifungal agents, we have witnessed a global increase in more resistant fungal species and genera. In addition, the large-scale use of antifungal agents in agriculture has selected for resistant *Aspergillus* species. There is a wide variation in the epidemiology of fungal infections worldwide, even between neighbouring countries depending on antifungal use in humans and the environment. Consequently, it is no longer acceptable to manage IFI wholly empirically and identifying the species of the infecting organism and its susceptibility profile to the various antifungal agents is key.

Studies have shown that delay in initiation of antifungal therapy is associated with a significant increase in mortality. Therefore, empirical antifungals are often started, but every effort must be made to make a prompt diagnosis of IFI. While histopathology and fungal cultures remain the cornerstone of a definitive diagnosis of IFI, these they have poor sensitivity and take many hours to days for a result. The availability of rapid fungal biomarkers, such as the *Aspergillus* galactomannan, β-D-glucan, and polymerase chain reaction, can—potentially—provide diagnostic information in hours. These tools have been used for screening and guiding pre-emptive therapy in high-risk patients. Studies have shown that combining biomarkers increases the sensitivity and specificity with an impact on fungal infection-free survival in some patients and allows for early discontinuation. More recently, the development of new molecular tools such as Matrix-Assisted Laser Desorption/Ionization—Time of Flight, FilmArray, Light Cycler, T2Candida assay, and others, allows the rapid detection of fungal pathogens and resistance markers with a turnaround time of only a few hours. Investigational tools such as Proximity Ligation Assay, Breath Fungal Secondary Metabolite Signature, and Siderophore-based Molecular Infection Imaging are promising tools for the diagnosis of IFI. Point-of-care testing is helpful, especially in diagnosing *Cryptococcal Meningitis* in some developing countries where access to microbiology laboratory is limited, but also for diagnosing invasive aspergillosis using Lateral Flow Devices.

These diagnostic tools can have a tremendous impact on clinical management of IFI. Antifungal stewardship (AFS) team plays a key role in optimising individual patient care, by supporting the frontline clinicians with expert knowledge on the use and limitations of rapid fungal biomarkers, guiding choice of diagnostics and choice of antifungal drugs. The AFS team ideally should be made up of an ID physician, pharmacist and a doctor from the clinical service the patient is in. Studies have shown that AFS programmes ensure optimal antifungal drug use, support de-escalate them to narrower-spectrum agents, reduce overall drug usage and cost and lead to better patient care. These efforts will ultimately translate into decreasing antifungal resistance and reducing overall healthcare cost.

## P38 Evaluation of Three Methodologies that Allow In Vitro Induction of the Capsule of *Cryptococcus neoformans* var. *grubii*

**José Rodas ^1^, John Gómez ^1^, Clara Duque ^1^, Claudia Cuervo ^1^, Iván Mojica ^2^ and Juan Gómez ^2^**

^1^ University Institution Colegio Mayor of Antioquia
^2^ Synlab Laboratory S.A.S.

**Objective:** To evaluate the efficacy of three methods for in vitro capsule induction of *Cryptococcus neoformans* var. *grubii*.

**Materials & Methods:** 15 strains of *Cryptococcus neoformans* var. *grubii* were used for being preserved in 20% skim milk, which were reactivated, isolated and subjected to the technique of Thomaz et al., 2016, Zaragoza & Casadevall 2004 and a modification of the Zaragoza & Casadevall 2004 method for the induction and capsule formation in vitro, identified by MALDI-TOF EM. The incubation time corresponded to 24 h. Longer periods do not influence the increase in capsule size significantly. For method (A) a McFarland pattern was prepared between 0.89 and 1.29 in sterile water with each strain, for method (B) and (C) a pattern between 2.3 and 2.5 with PBS and sterile water respectively. After 24 h of incubation, the samples were centrifuged at 870× *g* for 5 min and the supernatant was discarded; subsequently it was suspended in 100 µL of sterile water. Once suspended the cells, 20 uL of suspension and 10 uL of diluted Chinese ink were taken 1:8 for its microscopic reading. Using an Olympus CX33 microscope equipped with a calibrated micrometric ruler, the measurement of the total yeast diameter and the diameter of the yeast with the capsular space was performed. Finally, the difference between the circumference of the capsule and the yeast circumference was found to define the capsular size. From each method, 20 blastoconidias were chosen per strain and thus estimate the percentage of cells with capsule production (capsule induction capacity).

**Results:** Method (A) and (C) induced capsule in 100% (15/15) of the strains studied, method (B) only in 86.67% (13/15) of the strains; similarly, for method A 99.33% (280/300) produced capsules in all cells of the population while for method B and C 53.33% (160/300) these presented between the (5–20%) and (60–100%) of capsulated cells respectively. The size of the capsule varied according to the method and sizes were found between 0.5 µm and 2 and 11.5 µm.

**Conclusions:** Method (A) presented more efficiency in terms of production and capsule size. Biological variability plays a decisive role in recognizing ideal conditions and inducing the production of capsules similar to that formed in the host.

## P001 Critical Assessment of Cell Wall Integrity Factors Contributing to In Vivo Echinocandin Tolerance and Resistance in *Candida glabrata*

**Rocio Garcia-Rubio ^1^, Rosa Y. Hernandez ^1^, Alissa Clear ^1^, Kelley R. Healey ^2^, Erika Shor ^1,3^ and David S Perlin ^1,3,4^**

^1^ Center for Discovery and Innovation, Hackensack Meridian Health
^2^ Department of Biology, William Paterson University
^3^ Department of Medical Sciences, Hackensack Meridian Health School of Medicine
^4^ Georgetown University

**Objectives:** Fungal infections cause significant mortality and morbidity worldwide. For several decades, azole drugs have been the primary therapy to treat infections caused by *Candida* species. However, the extensive use of azoles has led to an epidemiological shift toward *Candida* species with higher azole tolerance, such as *Candida glabrata*. Thus, another drug class, the echinocandins, is currently recommended as first line antifungal therapy. The echinocandins target 1,3-β-glucan synthase (GS), the enzyme responsible for producing a major component of the fungal cell wall. Although echinocandins are considered fungicidal, *C. glabrata* exhibits echinocandin tolerance both in vitro and in vivo, where a subset of the cells survives and facilitates the emergence of echinocandin-resistant mutants by modifications in GS-enconding *FKS* genes. Unfortunately, these mutations are often associated with therapeutic failure. The objective of this work was to identify *C. glabrata* genes that contribute to echinocandin tolerance in vitro and in vivo.

**Methods & Materials:** To identify genes that contribute to *C. glabrata* echinocandin tolerance, we examined a collection of *C. glabrata* deletion mutants chosen because they were sensitive to cell wall damaging agents and/or because these genes or their orthologs in *Saccharomyces cerevisiae* were known to function in cell wall maintenance. We used an in vitro tolerance assay to measure the survival of each knock-out strain after 24-h exposure to a wide range of caspofungin concentrations. The deletion mutants that resulted in significantly reduced tolerance to caspofungin were therefore selected for further studies in vivo using a mouse model of *C. glabrata* gastrointestinal (GI) colonization. In this model, the mouse GI tract was sterilized using antibiotics and then colonized via oral gavage. Stably colonized mice were exposed to high-dose daily caspofungin treatment after three days of colonization. Fresh fecal samples were collected every other day throughout the experiment to assess fungal burden in the GI tract. Echinocandin susceptibility testing as well as *FKS* sequencing was performed in strains isolated from the GI tract of mice with fungal rebound 15 days after colonization.

**Results:** We identified three *C. glabrata* genes involved in the maintenance of cell wall integrity—*YPS1. YPK2*, and *SLT2*—whose deletion mutants showed echinocandin hyper-susceptibility in vitro. We also assessed their contribution to echinocandin tolerance and emergence of resistance in vivo. We found that mice colonized with strains carrying deletions of these genes were more effectively sterilized by daily caspofungin treatment relative to mice colonized with the wild-type parental strain. Furthermore, consistent with a role of tolerant cells serving as a reservoir for generating resistant mutations, a reduction in tolerance was associated with a reduction in the emergence of resistant strains. Finally, reduced susceptibility in these strains was due both to the well described *FKS*-dependent mechanisms and as yet unknown, *FKS*-independent mechanisms.

**Conclusions:** The above-mentioned genes involved in the maintenance of cell wall integrity contribute to echinocandin tolerance both in vitro and in a mouse model of gastrointestinal colonization, shedding light on the importance of this pathway in echinocandin tolerance and emergence of resistance in vivo.

## P002 Antimicrobial Potentials of Lantana Camara Montevidensis Leaf Extract on Wounds Infected with *Candida* Isolates Using Animal Models

**Ofonime Ogba, Sunday Edim and Stanley Anyawu**

University Of Calabar

**Objectives:** Medicinal plants are important source of medication in traditional system of medicine. The aim of this study was to determine the antimicrobial activity of *Lantana camara* leaf extract against clinical isolates of *Candida* from infected wounds invitro and in vivo using animal models. The objectives of the study was to isolate and identify *Candida* isolates from wounds of patients attending University of Calabar Teaching Hospital (UCTH). Determine the susceptibility pattern of the *Candida* isolates and the antimicrobial potentials of *Lantana camara* leaf extract against the yeasts isolates.

**Materials & Methods:** Aqueous and methanol leaf extraction was done using Soxhlet apparatus. Ten *Candida* isolates associated with wound infections were re-identified and used for the study. Antifungal susceptibility testing was done using the E-Test strips. Fifteen healthy male Wistar rats were used for the study. The rats were anesthetized before making incision wounds on the neck region. The rats were treated with 100 mg/mL, 50 mg/mL and 25 mg/mL concentration of extract topically. The skin tissues of the sacrificed rats were obtained for histological examination using Heamatoxylin and eosin technique.

**Results:** The susceptibility rate of the *Candida* isolates ranged from (0.0–40.0%). Fluconazole was the most effective antifungal. Isolates were most susceptible (40.0%) to 100 mg/mL concentration of extract. Rats treated with 100 mg/mL methanol extract had significant mean wound contractions of 70.33 ± 0.58 with damaged tissue repair.

**Conclusions:** Methanol extract of *Lantana camara* leaf has antimicrobial activity on *Candida* isolates and topical healing effect on *Candida* wound infections and may be used as an alternative to antimicrobials for *Candida* wound management.

## P010 Multifactorial Role of Mitochondria in Echinocandin Tolerance Revealed by Transcriptome Analysis of Drug-Tolerant Cells

**Rocio Garcia-Rubio ^1^, Cristina Jimenez-Ortigosa ^1^, Lucius DeGregorio ^1^, Christopher Quinteros ^1^, Erika Shor ^1,2^ and David S Perlin ^1,2,3^**

^1^ Center for Discovery and Innovation, Hackensack Meridian Health
^2^ Department of Medical Sciences, Hackensack Meridian Health School of Medicine
^3^ Georgetown University

**Objectives:** Echinocandin drugs are a first line therapy to treat invasive candidiasis, which is a major source of morbidity and mortality worldwide. The opportunistic fungal pathogen *Candida glabrata* is notable for rapidly evolving echinocandin-resistant strains associated with clinical failure. Echinocandin resistance is thought to emerge within a small echinocandin-tolerant subset of *C. glabrata* cells that are not killed by drug exposure, but mechanisms underlying echinocandin tolerance are still unknown. The objective of this study was to gain new insights into echinocandin tolerance in *C. glabrata* by examining the transcriptome of echinocandin-tolerant cells.

**Methods & Materials:** Fluorescence-activated cell sorting was used to highly enrich for *C. glabrata* cells that have survived prolonged echinocandin exposure in vitro, followed by single-cell RNA sequencing to examine the transcriptional landscape of echinocandin-tolerant cells. Based on the functional categories of differentially expressed genes, we selected different types of chemical inhibitors to modulate the most significantly up- or downregulated pathways to ask whether this modulation altered *C. glabrata* echinocandin tolerance. Based on these results, we focused on the role of mitochondria in echinocandin tolerance. We examined ROS levels in caspofungin-treated cells by using ROS-sensitive dyes followed by flow cytometry analysis. We used qRT-PCR to examine individually the expression of several genes involved in oxidative stress responses during caspofungin or micafungin treatment. We also generated a number of mitochondria-deficient strains lacking various mitochondrial components using CRISPR, as well several petite mutants that emerged during ethidium bromide or echinocandin treatment. All mitochondria-deficient mutants were examined for echinocandin tolerance using the caspofungin killing assay. Finally, partially purified 1,3-β-D-glucan synthase (GS) was obtained to measure kinetic inhibition by echinocandins in vitro.

**Results:** This analysis identified a transcriptional signature of echinocandin tolerance distinct from the stereotypical yeast environmental stress response characterized by upregulation of pathways involved in chromosome structure and DNA topology and a downregulation of oxidative stress responses, the latter of which was observed despite increased levels of reactive oxygen species. Further analyses showed that inhibitors of mitochondrial complexes I and IV reduced echinocandin-mediated cell killing. However, an ROS scavenger did not alter cell killing dynamics, indicating that ROS per se do not significantly contribute to cell death upon echinocandin exposure. We also found that mutants lacking various mitochondrial components, including both respiration-proficient and respiration-deficient strains, all showed an echinocandin hyper-susceptible phenotype at below-MIC concentrations. Finally, GS purified from mitochondrial mutants exhibited normal in vitro inhibition kinetics, indicating that mitochondrial defects influence cell survival downstream of the drug-target interaction.

**Conclusions:** These results provide new insights into the *C. glabrata* response to echinocandins, revealing a unique transcriptional signature as well as a multifactorial role of mitochondria in echinocandin tolerance.

## P011 In Vitro Antifungal Effect of Plant-Based Compound CIN-102 on Filamentous Fungi and Their Biofilm

**Maurine D’agostino ^1^, Nicolas Tesse ^2^, Jean Pol Frippiat ^1^, Marie Machouart ^1,3^ and Anne Debourgogne ^1,3^**

^1^ EA Simpa
^2^ SEPTEOS
^3^ CHRU Brabois

**Objectives:** Today the increase of invasive fungal infections (IFIs) due to immunosuppressive therapies and the emergence of resistant strains can induce therapeutic failure. To face it, the SEPTEOS company has recently developed CIN-102, including cinnamaldehyde and active compounds from two essential oils of cinnamon. CIN-102 has already been shown to be active against the three main genera of filamentous fungi (*Aspergillus* sp., *Fusarium* sp. and *Scedosporium* sp.) with a unimodal distribution of MIC (minimal inhibitory concentration). The objective of this study is to describe the mode of action of this natural mixture on filamentous fungi (fungistatic or fungicidal effect) and to visualize its effectiveness on the biofilm, a factor of virulence and resistance to antifungals.

**Materials & Methods:** Eight strains are studied: Fusarium solani, Fusarium dimerum, Lomentospora prolificans, Scedosporium apiospermum, Aspergillus flavus, *Aspergillus fumigatus* and two azole resistant A. fumigatus. CIN-102 is compared with two antifungal drugs used in clinical practice: amphotericin B (AMB) and voriconazole (VRZ).

In order to determine if CIN-102 has an fungicidal or fungistatic effect, a time kill assay was performed. After differents incubation times with antifungals, the Colony Forming Units (FCU) are counted. A decrease in FCUs greater than 3 log 10 is considered as a fungicidal effect.

Biofilms were formed after a 90-min adhesion phase followed by the addition of RPMI medium and after a 48 h at 37 °C. Antifungals at different concentrations were added, either after the adhesion phase or after the 48 h of incubation to study their effect on the formation and on the preformed biofilm. After washing, a mixture of XTT-menadione was added to the wells. After 60 min at 37 °C, the OD (optical density) is measured at 450 and 630 nm. An optical microscope observation allows the visualization of elements of the biofilm.

**Results:** The fungicidal effect of CIN-102 was demonstrated at 2 or 4 MIC for all strains tested from 12 to 24 h for *Fusarium*. *Aspergillus* and Lomentospora prolificans and from 48 h for *Scedosporium apiospermum*.

We observed an antifungal effect of CIN-102 on biofilm, and particularly on its formation, with 100% inhibition achieved from 1 MIC. Finally, CIN-102 was able to inhibit 100% of biofilm formation by five other tested strains (*A. fumigatus, A. flavus, F. dimerum, L. prolificans* and *S. apiospermum*) against one for VRC (*A. fumigatus*) and two for AMB (*A. fumigatus* and *A. flavus*) and it was effective at low concentration for all of the strains (from 1/2MIC to MIC).

**Conclusions:** In conclusion, CIN-102 is active on filamentous fungi with fungicidal activities and actions on biofilm, it can be an interesting candidate as a new antifungal class. Further study including an in vivo study will be carried out in order to validate the potential of CIN-102 to offer new treatment possibilities against these pathologies.

## P012 Detection of Wild-Type *Candida* Vaginal Isolates with Reduced Susceptibility to Azoles Used for the Treatment of Vulvovaginal Canididiasis

**Joseph Meletiadis ^1,2^, Lamprini Kanioura ^2,3^, Maria Siopi ^1^, Drosos Karageorgopoulos ^4^, Flavia de Bernardis ^5^ and Stavroula Antonopoulou ^6^**

^1^ Clinical Microbiology Laboratory, Attikon University Hospital
^2^ Department Medical Microbiology and Infectious Diseases, Erasmus MC
^3^ MycoLab, Diagnostic Laboratory of Sexually Transmitted Diseases, Specific Infectious Diseases, Fungal, Microbiological and Cytologic Examinations
^4^ 4th Department of Medicine of the University of Athens, Attikon University Hospital
^5^ Department of Infectious Diseases, Istituto Superiore di Sanità
^6^ Department of Microbiology, General Hospital “G. Gennimatas”

**Objectives:** Azoles are the first line therapy of vulvovaginal canididiasis (VVC) with various efficacy rates against different *Candida* species. However, there no clinical breakpoints neither epidemiological cut-off values (ECOFF) for these drugs and Candida vaginal isolates hindering the detection of non-wild phenotypes and antifungal resistance. We therefore determined the ECOFFs of different azoles used in the treatment of VVC against *Candida* vaginal isolates and compared them with the MICs of fluconazole resistant isolates.

**Materials & Methods:** The in vitro activity of 216 yeast isolates (102 *C. albicans*, 93 *C. parapsilosis*, 21 *C. glabrata*) against fluconazole, ketoconazole, miconazole, econazole, itraconazole and clotrimazole) were tested with the EUCAST EDef 7.3.2. The correlation between fluconazole MICs and MICs of the other azoles were analyzed with Pearson correlation after log_2_ transformation. The MIC distributions were analysed with the ECOFFinder2.0 in order to define the wild-type population and determine local ECOFFs. The MICs of azoles for fluconazole susceptible (MIC ≤2 mg/L) and non-susceptible (MIC >2 mg/L) isolates were determined for each species.

**Results:** Statistically significant correlation was found between fluconazole MICs and MICs of all the other azoles for *C. albicans* (r = 0.73–0.95, *p* < 0.001) and *C. parapsilosis* (r = 0.23–0.75, *p* < 0.001) whereas for *C. glabrata* significant correlation was found only for itraconazole and clotrimazole (r = 0.83–0.90, *p* < 0.001). The median (range) MICs of fluconazole, ketoconazole, miconazole, econazole, itraconazole and clotrimazole for fluconazole susceptible and non-susceptible isolates together with ECOFFs encompassing >97.5%/99% of wild-type MICs for *C. albicans, C. parapsilosis* and *C. glabrata* are shown in Table 1. Although there was some overlapping between the MICs of fluconazole susceptible and non-susceptible isolates, the differences were statistically significant (*p* < 0.05) with the median MICs differ by >2 twofold dilutions in most cases. ECOFFs were 1–2 twofold dilutions higher than the median MIC of fluconazole susceptible isolates. However, the median MIC of fluconazole non-susceptible isolates were ≥2 twofold dilutions higher than the corresponding ECOFFs only for *C. albicans* and all azoles except and clotrimazole and for *C. parapsilosis* for ketoconazole and itraconazole.


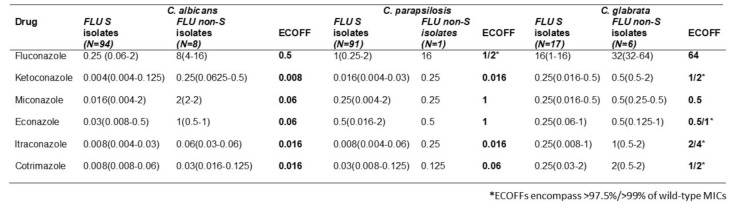


**Figure 1.** The median (range) MICs for each azole and Candida species for fluconazole susceptible (FLU S) and no-susceptible (FLU non-S) isolates together with estimated local epidemiological cut-off values (ECOFF).

**Conclusions:** Significant differences of ketoconazole, miconazole, econazole, itraconazole and clotrimazole MICs were found between fluconazole susceptible and non-susceptible isolates. Non-wild type phenotypes were associated with reduced azole susceptibility particularly for *C. albicans*. The estimated ECOFFs could be used to define wild-type population of *Candida* vaginal isolates and detect isolates with reduced susceptibility to azoles. Multicentre MIC distributions are required to verify wild-type populations and determine official ECOFFs for vaginal isolates.

## P013 Difficult-to-Treat Cryptococcal Meningitis in an Immunocompetent Patient: Antifungal Therapy Toxicity, Fluconazole Resistance and Severe Intracranial Hypertension

**Miguel Ángel Verdejo-Gómez ^1^, Lorena Salmerón-Godoy ^1^, Carmen Moreno de la Santa ^1^, Manuel Lizasoain-Hernández ^1^, Ana Pérez de Ayala ^2^, Francisco Jiménez-Morillas ^4^, María Pilar Hernández-Jiménez ^3^ and Carlos Lumbreras-Bermejo ^1^**

^1^ Internal Medicine Department, Hospital Universitario 12 De Octubre
^2^ Microbiology and Parasitology Department, Hospital Universitario 12 de Octubre
^3^ Internal Medicine Department, Hospital Universitario La Princesa
^4^ Emergency Department, Hospital Universitario 12 de Octubre

**Objectives:** *Cryptococcal Meningitis* is a rare condition in non-HIV, immunocompetent hosts. Clinical experiences must be shared in order to increase the knowledge about this infection.

**Materials & Methods:** We report the case of a 59-year-old woman who attended to the Emergency Deparment for 10-days progressive headache, nausea and vomiting. Se had a personal history of obesity, dyslipemia and classic migraine. She had not travelled abroad recently. On initial evaluation she was afebrile, with no abnormal findings in physical and neurological examination. Blood examination showed leukocytosis, mild hypokaliemia and mild lactate deshydrogenase increase. Ophthalmological fundus examination suggested intracranial hypertension (ICH) and a cranial computed tomography (CT) with intravenous contrast showed neither intraparenchymal injuries nor vascular complications. Eventually, lumbar puncture was performed obtaining a clear cerebrospinal fluid at an opening pressure of 44 cmH_2_O (normal <20 cmH_2_O), cyto-biochemical analysis with mononuclear-dominance pleocytosis (60 leukocytes/μL, 80% mononuclear), hypoglycorrhachia (45 mg/dL, with blood glucose: 120 mg/dL) and proteinorrachy (0.74 g/L). Urgent Gram’s staining showed inflammatory cells and the presence of yeasts, with Chinese ink staining that showed encapsulated blastoconidium cells compatible with *Cryptococcus* spp. (Figure 1). The latex agglutination for Cryptococcus antigen resulted positive at a >1/128 titer. After these findings, we started induction treatment for *Cryptococcal Meningitis* with liposomal Amphotericin B 4 mg/kg/day and Flucytosine 25 mg/kg/6 h.

**Results:** A comprehensive study of clinical predisposing conditions was performed with no findings. Thoracic-abdomen-pelvis CT did not show hidden neoplasia. After two weeks of treatment, we observed severe neutropenia secondary to flucytosine, which lead us to a substitution by high-dose fluconazole. Furthermore, she continued with moderate hypokalemia due to both persistent vomiting and amphotericin B toxicity, requiring prolonged parenteral supplementation.

Microbiological characterization was consistent with *Cryptococcus neoformans var grubii*, and susceptibility pattern revealed fluconazole Minimal Inhibitory Concentration (MIC) of >32 µg/mL by broth microdilution commercialized method, so we decided to change Fluconazole to Voriconazole for completing the induction scheme, which finally consisted in Voriconazole 300 mg bid plus liposomal amphotericin B at dose described previously for a total of 30 days.

Notwithstanding, the patient maintained persistent syntomatic ICH (range 29 to 50 cmH_2_O) despite daily lumbar puncture and an adequate microbiological response. Therefore, we decided to place a ventriculus-peritoneal derivation (VPD) that allowed us controlling intracranial pressure, with significant clinical improvement.

Once induction treatment was completed, we started consolidation treatment with oral Voriconazole 200 mg bid, buy an elevation of gamma-glutamyl transferase more than 10 times upper limit was developed. Voriconazole was stopped with subsequent normalization of liver enzymes. Instead, Isavuconazole 200 mg qd was used for up to 12 months.

Currently, the patient is not receiving antifungal therapy, she is in a good clinical condition with no evidence of microbiological relapse.

**Conclusions:** In conclusion, we present a 59-year-old immunocompetent women who developed acute meningitis due to *Cryptococcus neoformans*, requiring the use of new azoles for its treatment and with ICH poor control which forced us to place a VPD, resulting in a favorable outcome.


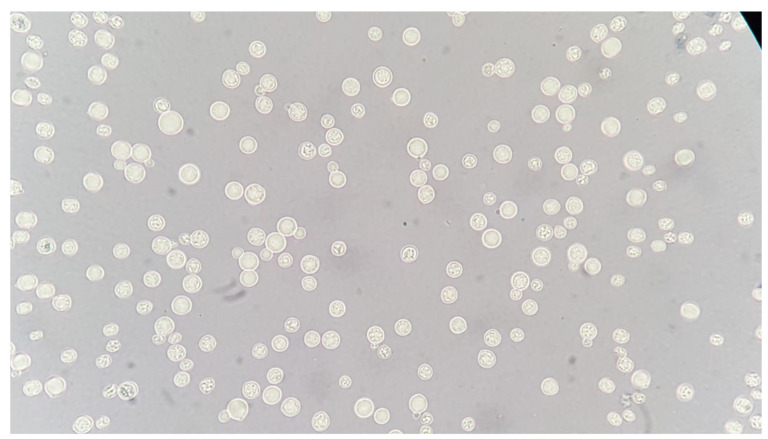


**Figure 1.** Chinese ink stain on CSF culture.

## P014 Elucidating Amphotericin B Tolerance, a Neglected Phenotype in Clinical Isolates of *Aspergillus terreus*

**Roya Vahedi-shahandashti and Cornelia Lass-Flörl**

Institute of Hygiene and Medical Microbiology, Medical University Innsbruck

**Objectives:** Infections due to *Aspergillus* species are an acute threat to human health. *Aspergillus* species within the section *Fumigati* are the most frequently occurring agents, but depending on the local epidemiology, representatives of section *Terrei* or section *Flavi* are 2nd or 3rd most important. *Aspergillus terreus* holds an exceptional position within the aspergilli due to its reduced sensitivity to amphotericin B (AmB). Usually, AMB minimum inhibitory concentrations (MIC) are high (>2 mg/L), but a broad range of MIC phenotypes have been reported in the absence of clinical breakpoints for AMB. The discordance between treatment outcome and infections due to the “AMB susceptible *A. terreus*” may be attributable to drug tolerance. This study aimed to examine the killing kinetic patterns of AMB against *A. terreus* isolates to define tolerant phenotypes.

**Materials & Methods:** Tolerance is the ability of a “susceptible isolate” to grow in the presence of AMB above the MIC; tolerant fungi are characterized by a longer minimum duration of killing time (MDK) than susceptible isolates. We investigated AMB-MDKs of *A. terreus* sensu stricto isolates (n = 4) representing susceptible (S < 1 mg/mL) and resistant (R > 4 mg/mL) phenotypes (EUCAST). Time-kill curve analysis (99% killing of the initial population) was performed in RPMI 1640 and AM3 media at different AMB concentrations, and in parallel, the germination rate was determined microscopically.

**Results:** Time-kill curves of AMB against *A. terreus* 164 (MIC = 0.5 μg/mL), *A. terreus* 81 and 31 (each AMB MIC = 1 μg/mL), and *A. terreus* 134 (MIC = 4 μg/mL) showed different killing patterns in AM3 and RPMI 1640, respectively. *A. terreus* 81 and *A. terreus* 31 showed tolerant phenotypes in AM3 at AMB concentrations of 20× MICs and 40× MICs. No tolerant phenotypes were detected in RPMI 1640 media. All isolates showed a significantly faster germination rate in AM3 medium when compared to RPMI 1640, evaluating different time points.

**Conclusions:** Our study revealed that *A. terreus* with AMB MICs = 1 mg/mL displays tolerant phenotypes, and that nutrition media may influence this activity. AM3 medium potentially supports the fungicidal activity of AMB against *A. terreus* by inducing rapid germination (vulnerable against AMB) in an early stage of incubation. The combination of the MDK and MIC provides a new framework to identify AMB-tolerance in *A. terreus*.

## P015 Polyunsaturated Fatty Acids Enhance the Activity of Fluconazole against *Candida krusei* In Vitro and in a *Caenorhabditis elegans* Model

**Abdullahi Temitope Jamiu, Jacobus Albertyn, Olihile M. Sebolai and Carolina H. Pohl**

Pathogenic Yeast Research Group, Department of Microbiology and Biochemistry, University of the Free State

**Objectives:** Although *Candida albicans* remains the major cause of invasive candidal infections, the incidence of infections caused by non-*albicans Candida* species, including *C. krusei* especially in immunodeficient population, is rapidly increasing and demands urgent public health attentions. *Candida krusei* intrinsically exhibits resistance to fluconazole while also rapidly displaying adaptive resistance to other antifungal drugs. Moreover, like other common *Candida* spp., this yeast has the propensity to form recalcitrant biofilm with increased resistance. Hence, the need to develop novel and effective therapeutic strategies to combat infections caused by this pathogen is undoubtable. One such approach is through combination therapy with natural compounds such as polyunsaturated fatty acids (PUFAs). This study was conceptualised to investigate the potentiating effect of PUFAs with fluconazole against *C. krusei* biofilm in vitro, as well as the conserved nature of this effect in a *Caenorhabditis elegans* infection model.

**Materials & Methods:** This study was carried out by exposing *C. krusei* biofilm to the sub-inhibitory concentration (SMIC_50_) of fluconazole alone or in combination with PUFAs; linoleic acid (LA) or gamma-linolenic acid (GLA), and evaluating the biofilm metabolic activity using XTT reduction assay. Subsequently, the influence of these treatments on biofilm morphology, membrane integrity, oxidative stress, and efflux pump activity was also evaluated with scanning electron microscopy, propidium iodide, antioxidative, and Rhodamine 6G efflux assays, respectively. In addition, the ability of the PUFAs to prolong the survival and reduce the fungal burden of infected *C. elegans* was assessed.

**Results:** Our results indicated that both PUFAs individually enhance the activity of fluconazole towards *C. krusei* biofilm in vitro via cell membrane damage, elicitation of oxidative stress, disruption of efflux pump activity, and ultimate induction of cell rupture. Additionally, the PUFAs potentiate the activity of fluconazole towards *C. krusei* in vivo, evident of the enhanced survival and significant reduction in fungal burden of infected *C. elegans*.

**Conclusions:** Taken together, PUFAs show potential as antifungal-potentiating agents against intrinsically fluconazole-resistant *C. krusei*, both in vitro and in vivo. This finding may pave the way for future studies into novel therapeutic options for overcoming increasing antifungal resistance.

## P016 Antifungal Susceptibility of Clinical *Cryptococcus gattii* Isolates from Colombia Varies among Molecular Types

**Carolina Firacative ^1^ and Patricia Escandón ^2^**

^1^ Universidad Del Rosario
^2^ Instituto Nacional de Salud

**Objectives:** To determine if clinical Colombian *Cryptococcus gattii* isolates of the molecular types VGI, VGII and VGIII differ in the susceptibility to commonly used antifungal drugs, as cryptococcosis by *C. gattii* is endemic in Colombia, affecting mostly immunocompetent hosts, and considering that antifungal susceptibility differences between molecular types of cryptococcal isolates have been reported in various other countries.

**Materials & Methods:** *C. gattii* isolates from 42 patients recovered in 15 Colombian states were included. Most patients (88.1%) did not have any risk factor for cryptococcosis. Susceptibility testing was carried out using Sensititre YeastOne plates (Thermo Scientific, Waltham, MA, USA), to amphotericin B, 5-flucytosine, fluconazole, itraconazole, voriconazole and posaconazole. Minimum inhibitory concentrations (MICs) of each antifungal drug were determined per molecular type and for all isolates. Mode and geometric mean MICs were calculated. MICs per drug and molecular type were compared with epidemiologic cut-off values (ECV) >95%, to determine if the isolates belong to the wild-type distribution, as established with worldwide isolates.

**Results:** Most (85.7%) *C. gattii* isolates from Colombia distribute among the wild-type populations of each molecular type, per antifungal drug. However, six (14.3%) non-wild-type isolates were identified. Among the molecular type VGII, two isolates were simultaneously fluconazole and voriconazole non-wild type. In addition, in VGI and VGIII, one 5- flucytosine and three fluconazole non-wild type isolates were identified, respectively. In general, the susceptibility of the studied isolates to amphotericin B did not differ among molecular types. The molecular type VGI was found to be less susceptible to 5-flucytosine than VGII. In addition, VGII was found to be less susceptible to fluconazole, itraconazole and voriconazole than VGI and VGIII. VGII was also less susceptible to posaconazole than VGIII.

**Conclusions:** This study emphasizes the importance of identifying reduced drug susceptibility of cryptococcal isolates when treating cryptococcosis and of establishing the molecular types of the isolates. MIC determination is essential, with special attention to the in vitro activity of fluconazole, as this is a drug with long-term usage that is prescribed in the three-part strategy of induction, consolidation, and maintenance, as such, the emergence of high-level of fluconazole resistance is probable. Reduced susceptibility to other azoles must be surveyed as well since these drugs are salvage consolidation therapies when fluconazole is not available. In general, broaden investigations of the genetic basis of reduced antifungal susceptibilities phenotypes in *C. gattii* are warranted, as it is still unknown what causes the differences in the in vitro antifungal susceptibilities of the molecular types and the occurrence of resistance when there has not been previous exposure to the drugs.

## P025 Improving Laryngectomy Clinical Practice, Patient Care, and Quality of Life by Tackling *Candida* Biofilm Formation on Voice Prostheses

**Campbell W. Gourlay, Daniel R. Pentland, Fritz A. Muhlschlegel and Jack Davis**

University of Kent

**Objectives:** To test whether a precise anti-fungal treatment regime can extend voice prosthesis lifespan thus offering a new evidence-based management approach that can be adopted within the clinic.

**Materials & Methods:** We formed a multi-disciplinary team consisting of ENT practitioners and University based researchers. Lab based experiments and clinical microbiology analyses were directed towards our research questions. This approach led to the construction and NHS ratification of a set of treatment guidelines. The guidelines were then applied to a patient cohort and analysed for significance in a study spanning seven years.

**Results:** Here we report that colonisation of VP by the yeast is the major problem underlying device failure and that *C. allbicans* is the most predominant coloniser. We present evidence that the high CO_2_ environment generated by exhaled breath is a key driver of *C. albicans* biofilm development on VP. Elevated CO_2_ activates and accelerates the biofilm program and enhances key support attributes including adhesion, iron acquisition, glucose uptake and hyphal transition. We present evidence from a patient study that antifungal treatment extends VP lifespan by an average of 270%, a the most significant enhancement of prosthesis performance reported to date. We also present evidence that antifungal treatment can reduce the ability of bacteria such as *S. aureus,* to colonise VP as a result of the inhibition of *C. albicans*-bacterial interactions.

**Conclusions:** As a result of our work we have introduced a robust anti-fungal treatment plan that significantly extends VP lifespan in patient studies. Our clinical care pathway offers a real improvement to patient care and is now being used in a number of NHS trusts across the UK.

## P026 Antibiofilm Activity of Olorofim (F901318) against Azole-Resistant *Aspergillus fumigatus*

**Lisa Kirchhoff ^1^, Silke Dittmer ^1^, Dan-Tiberiu Furnica ^1^, Jan Buer ^1^ and Joerg Steinmann ^1,2^**

^1^ Institute of Medical Microbiology, University Hospital Essen
^2^ Institute of Clinical Hygiene, Medical Microbiology and Infectiology, Klinikum Nuernberg

**Objectives:** In recent decades, the number of reports on invasive infections with azole-resistant *Aspergillus fumigatus* (ARAF) increased. Especially biofilm forming fungi can cause refractory infection-manifestations (e.g., in cystic fibrosis or immuno suppression) which are usually linked to increased resistances to anti-infective agents. The need of novel anti-infective therapeutics with new underlying mechanisms against several fungal infections is emerging. Here, the novel antifungal compound olorofim (F901318), which belongs to the new class of the orotomides, targeting the dihydroorotate dehydrogenase within the de novo pyrimidine biosynthesis pathway, has been tested for its activity against different stages of ARAF and non-ARAF biofilms.

**Materials & Methods:** Olorofim activity has been analyzed at different stages (prior adhesion, 4 h, 12 h, 24 h and 48 h after inoculation) of biofilm formation of clinical ARAF strains (n = 16) as well as azole susceptible *A. fumigatus* isolates (n = 4) by using an XTT assay. Olorofim was applied in concentrations of a total, a half, a quarter and an eighth of the previously determined minimum inhibitory concentrations (MICs) by broth microdilution method after EUCAST.

**Results:** Olorofim showed MICs between ≤0.008 and 0.03 mg/L against the here included *A. fumigatus* isolates. Antimicrobial and antibiofilm activity of olorofim has been detected to be strain specific. Best antibiofilm activity has been observed when olorofim was added prior to adhesion (t0) and right after adhesion (t4) with biofilm reduction up to 96% compared to the non-treated control. In contrast, olorofim did not show an effect on mature (t24 & t48) *A. fumigatus* biofilm. Additionally, the germinated biofilm of *A. fumigatus* has been detected to be resistant towards olorofim.

**Conclusions:** In conclusion, the novel drug olorofim showed promising effects against initial phases of *A. fumigatus* biofilm formation, regardless their susceptibility towards azoles. However, no effect on mature biofilms was found.

## P027 Combined Activity of Liposomal Amphotericin B and Voriconazole against *Fusarium solani* and *Scedosporium apiospermum* Biofilms

**Aikaterini Vikelouda ^1^, Maria Simitsopoulou ^1^, Charalampos Antachopoulos ^1^, Lemonia Skoura ^2^ and Emmanuel Roilides ^1^**

^1^ 3rd Department of Pediatrics, Aristotle University, School of Medicine
^2^ Department of Microbiology, Aristotle University, School of Medicine

**Objectives:** *Fusarium* species and *Scedosporium apiospermum* are distributed worldwide and considered important emerging pathogens, causing locally invasive or disseminated fungal diseases in humans especially among immunocompromised hosts. *Fusarium* spp. are related to vision-threatening keratitis among other infections, while *S. apiospermum* frequently colonizes airways of cystic fibrosis patients chronically impairing respiratory function. The ability of both fungi to form biofilms (BF) on solid and tissue culture surfaces has been suggested as an important factor that may contribute to the pathogenicity profile of these organisms. Little is known about drug combinations against *F. solani* or *S. apiospermum* biofilm-derived infections. We assessed the in vitro damaging activity of liposomal amphotericin B (L-AMB) and voriconazole (VRC) against *F. solani* (FS) and *S. apiospermum* (SA) mature BF alone or in combination.

**Materials & Methods:** *F. solani* (n = 3) and *S. apiospermum* (n = 3) clinical strains were incubated at 10^5^ cfu/mL in 96-well microtiter plates at 37 °C for 48 h. BF formation was assessed by 1% safranin staining and quantitated spectrophotometrically at 490 nm. For MIC determination, two-fold dilutions of L-AMB and VRC (0.007–256 mg/L) were incubated with BF for 24 h (n = 6–9). The combinational activity of L-AMB (0.5–32 mg/L) with VRC (0.125–64 mg/L) against BF at 37 °C for 24 h was determined using a checkerboard microdilution method (n = 10). BF damage compared to controls was assessed by XTT metabolic reduction assay. MIC50 was determined as ≥50% BF damage. Drug interactions were analyzed using Bliss independence model. The combination effect was defined as synergistic, antagonistic or indifferent when the observed BF damage was significantly higher, lower or equal to the expected damage, respectively.

**Results:** All strains of both organisms formed strong BF (OD range: 0.195–0.238). LAMB and VRC BF MIC50’s for *FS* were 2 and >256 mg/L, whereas MIC50’s for *SA* were 2 and 32 mg/L, respectively. LAMB was significantly more effective against BF of *FS* and *SA* compared to VRC (2 mg/L vs. >256 and 32 mg/L, respectively; *p* < 0.05), exhibiting comparable activities against either organism. Combinational treatment of *SA* biofilms resulted in synergistic effect at 2–4 mg/L of L-AMB combined with 4–16 mg/L of VRC. Mean ΔE value of significant interactions, 17% [range, 14% to 20%]; mean SE, 3.7% [range, 3.0% to 4.3%]. By comparison, antagonistic effects at concentrations of 0.5–4 mg/L of L-AMB combined with 0.125–16 mg/L of VRC was observed against *FS* biofilms, demonstrating mean ΔE value of significant interactions −28% (range: −11% to −55%) with mean SE 4.6% (range: 2.5–7.9). In contrast.

**Conclusions:** While a comparable antifungal effect is achieved by LAMB alone against BF of *F. solani* and *S. apiospermum*, voriconazole activity is poor against BF of either organism. However, combination of LAMB with VRC at near BF MIC concentrations exhibits synergistic activity against *S. apiospermum* BF, whereas the combined activity of LAMB with VRC is antagonistic against *F. solani* BF. These findings may have important implications in the treatment of biofilm-related infections caused by these fungi.

## P028 *Candida auris* Biofilm Heterogeneity Influences the Host Response in an In Vitro Wound Model

**Jason L. Brown ^1,3^, Christopher Delaney ^1,3^, Bryn Short ^1,^^3^, Mark C. Butcher ^1,3^, Emily McKloud ^1,3^, Craig Williams ^1,3^, Ryan Kean ^2,3^ and Gordon Ramage ^1,3^**

^1^ University of Glasgow
^2^ Glasgow Caledonian University
^3^ Glasgow Biofilms Research Network

**Objectives:** In the years since its sudden emergence in 2009, *Candida auris* quickly established itself as a prolific nosocomial pathogen and a global health risk. This enigmatic yeast has exhibited two distinct growth phenotypes that have been termed aggregating and non-aggregating cells. The aggregating phenotype stems from the inability of daughter cells to detach from the parent cell during dell division. Limited data has previously shown that these cell phenotypes influence traits such as biofilm formation and virulence. Herein we aimed to investigate the role of differing cell phenotypes in biofilm formation and virulence using in vitro skin epithelium infection models.

**Methods:** In this study, we screened a panel of *C. auris* isolates for biofilm formation, using an electron impedance method and biofilms were subsequently viewed either using scanning electron microscopy or confocal imaging via labelling with novel fluorescent smart-probes. Next, the transcriptional profile of aggregating and non-aggregating isolates during biofilm formation was compared using RNA-sequencing.

**Results:** These analyses showed a high level of biofilm heterogeneity between cell phenotypes and this was reflected in their respective transcriptomes. Analysis revealed an upregulation of genes relating to the fungal cell wall, adhesion and host cell invasion. Building upon these findings, we then investigated, for the first time, the fungal recognition and inflammatory response of a three-dimensional skin epithelial model to *C. auris*. In addition, a wound was inflicted on this epithelial model to mimic a portal of entry for the fungal cells. Although both cell phenotypes elicited a minimal response without a wound, when a passage of entry was created, both aggregating and non-aggregating cells induced a greater inflammatory response with the aggregating phenotype being more proinflammatory.

**Conclusions:** The ability of *C. auris* to generate such immune responses in wounded skin shows why this yeast has become such a high risk within nosocomial environments where susceptible patients may have multiple indwelling lines.

## P029 Omic Modelling of *Candida albicans* Biofilms

**Christopher Delaney Delaney ^1^, Jon Pratten ^2^, Dave Bradshaw ^2^ and Gordon Ramage ^1^**

^1^ University Of Glasgow
^2^ Oral Health R&D, GlaxoSmithKline

**Objectives:** *Candida* biofilms are a substantial clinical and human health burden which are still underappreciated. Benefits afforded by morphogenic switching from planktonic to biofilm communities include resistance to antimicrobials in the host’s immune system, and resilience towards mechanical disruption, all of which complicate the treatment and management of infections. Biofilm formation in *Candida* spp. is influenced by numerous factors, including response to the host, pH, bacteria, and many other environmental factors. We aimed to profile and to decipher the mechanisms that underpin the observed heterogeneity and phenotypic differences that we observe in clinical strains. Additionally, we aimed to discern molecular and metabolic signatures of biofilm formation in response to environmental and bacterial stimulus.

**Methods & Materials:** *C. albicans* isolates derived from BSI have been profiled and identified to be phenotypically distinct in their biofilm formation. Transcriptomics by RNA-Seq, proteomics by MS and metabolomics by MS was performed on phenotypically distinct *C. albicans* strains, biofilm competent and biofilm deficient, clinical isolates +- foetal calf serum (FCS). Data sets were integrated by computational, pathway-based and network methods to elucidate adaptive biofilm mechanisms. *C. albicans* has also been profiled in the presence of numerous bacterial species using established RNA-Seq pipelines to identify *C. albicans* transcriptional response to bacterial adhesion.

**Results:** Differences were observed in the biofilm competent (HBF) compared to the biofilm deficient (LBF) isolates with both susceptibilities and drug resistance correlated with the biofilm forming ability. From our high and low *Candida* isolates we observed phenotypic switching of LBF in the presence of serum. We also found functional differences related to phenotype and this observed switching. The LBF response to serum included enrichment in fatty acid and acl-coA metabolic pathways. Metabolomic analysis revealed changes in arachidonic acid metabolism in serum grown isolates and changes in the amino acid metabolism between LBF and HBF isolates. Integrating these data conceptually we were able to observe overlaps in the metabolic reprogramming of *C. albicans* isolates in serum with joint pathway analysis confirming changes in the fatty acid metabolic response in both transcriptomic and metabolomic data.

**Conclusions:** Through the application of transcriptomics and metabolomics we have demonstrated that these holistic methodologies are invaluable to biofilm research. We identified molecular processes and metabolomic reprogramming of *C. albicans* in response to the biofilm inducing stimulus of serum. We also highlight the current and potential benefits that integration of multiple omics data sets provides. Integration is not without its challenges, however, and we identify some key methodologies that could improve interpretability of omics datasets derived from microbial communities.

## P030 Differences in Yeast Biofilm Production According to Species and Clinical Origins

**Seydou Nakanabo Diallo ^2^, Ahalieyah Anantharajah ^1^, Maria Isabel Montesinos Hernandez ^3^, Marie Robins ^1^, Françoise Van Bambeke ^4^, Hector Rodriguez-Villalobos ^1^**

^1^ Cliniques Universitaires Saint Luc-UCL
^2^ Intitut national de Santé Publique
^3^ Laboratoire Hospitaliere Universitaire de Bruxelles LHUB
^4^ Louvain Drug Reseach Institute

**Objectives:** Biofilm production (BP) on implanted biomaterials and/or host surfaces is recognised as one of the most important virulence factors of *Candida* spp. and is associated with increased resistance to antifungal agents. There is however, a lack of data concerning the *Candida* spp. BP on different clinical sources. We analysed the BP by clinical isolates of yeast isolated from different infection sites.

**Methods & Materials:** A total of 122 yeast (106 clinical isolates) including 18 spp. were analysed for BP. Clinical isolates included 74 *C. albicans* and 32 non-*albicans yeast* (*C. glabrata, C. tropicalis, C. lusitaniae, C. kefyr, C. melibiosica, C. dubliniensis, C. parapsilosis, C. intermedia, C. norvengensis, C. krusei, C. fabiani, Exophiala dermatidis, Cryptococcus neoformans* and *Saccharomyces cerevisiae*). Strains were isolated from candidemias (n = 21), vaginosis (n = 21), urinary tract infections (n = 9), gastric infections (n = 2), respiratory tract including Cystic fibrosis (CF) patients (n = 50) and other sites (n = 3). Biofilms were grown in 96-well cell culture microplates in Sabouraud dextrose broth for 48 h and the biofilm biomass was evaluated by crystal violet staining. Results were normalized according to the CFU/mL and compared to *C. albicans* ATCC 24433, used as reference strain.

**Results:** All species analysed were biofilms producers. *C.tropicalis, C. melibiosica, C. fabiani, C. krusei, C. parapsilosis* showed 2–3 times higher bivomass level compared to reference strain. Among clinical isolates, 65% showed BP (58% of *C. albicans* and 75% of *Candida* non-*albicans*). Considering the clinical origin, 57% of *Candida* spp. isolated from candidemia, 45% of *Candida* spp. from urinary tract infections, 61% of *Candida* spp. from vaginosis, 62% *Candida* spp. from respiratory tract and 100% *Exophiala dermatidis* from CF patients were able to produce biofilm. Non-Significant differences on BP were noted among *C. albicans* isolates based on the infection site.

**Conclusions:** Our data showed that all tested species of yeast were biofilm producers including *C. melibiosica, C. palmioleophila, C. valida, Metschnikowua pulcherrima, Kluveyromyces lactis, C. sorbosa* and *C. pararugosa* (non-previously described). The majority of clinical isolates are biofilm producers. However significant differences in biofilm biomass were observed according to the species. Our data suggest an adaptation of some fungal species (*Exophiala* spp.) to clinical environment particularly in respiratory tract to cystic fibrosis patients, providing a sanctuary to the biofilm growth.

## P031 Antifungal Tolerance on Mono and Dual-Species Biofilm

**Ouassila Bekkal Brikci-Benhabib ^1^, Zahia Boucherit-Otmani ^2^ and Kebir Boucherit ^2^**

^1^ University Of Ain Temouchent
^2^ Laboratory Antibiotics Antifungals: Physico-chimical, Synthesis and Biological Activities, University of Tlemcen, Algeria

**Introduction:** Infections associated with vascular catheters have seen their incidence increase over the last decade because of their insertion which has become a systematic gesture at least on an interim basis. These vascular catheters provide a support for the adhesion of several microorganisms, in particular the yeasts Candida. The latter are capable of forming mono or multi-species biofilms, which originate from a high tolerance to clinically used antifungal agents.

It is in this context that our study aims to investigate the rate of mono or multi-species fungal alterations of peripheral venous catheters and to test their sensitivity to amphotericin B and Caspofungin in the planktonic state and also in biofilm mode.

**Materiel and methods:** The isolated strains were identified by phenotypic and genotypic methods and were analyzed to determine their minimal concentrations inhibiting their growth in planktonic and biofilms forms using amphotericin B and caspofungin.

**Results:** The results obtained showed that 12.10% of all catheters collected were by *Candida* sp. and 2.73% of catheters were contamined by *Saccharomyces cerevisiae, Cryptococcus neoformans, Trichosporon asahii*.

The mono-species alteration of the catheters is greater than that of the dual-species with a frequency of 79 and 21% respectively. Mixed combination isolated isolated on catheters is *Candida albicans/Candida glabrata* and *Candida parapsilosis/Trichosporon*.

All isolated strains in planctonic form were susceptible to amphotericin B with Minimum Inhibitory Concentrations (MIC) ranging from 0.03 to 1 mg/mL. MIC obtained with caspofungin vary from 0.06 to 1 μg/mL. In contrast, sessile cells of yeast mono and dual-species biofilms formed ranging from 0.25 to 8 μg/mL for amphotericin B and caspofungin ranges from 0.5 to 16 μg/mL.

**Conclusions:** Sessile Minimum Inhibitory Concentrations (SMIC) of *Candida* sp. and other yeast isolates in their mono and dual-species biofilms are up to 32 times greater than MICs for amphotericin B and 64 times for caspofungin.

## P032 Quantitative Analysis of In Vitro Biofilm Formation by Clinical Isolates of Dermatophyte and Anti-Biofilm Activity of Common Antifungal Drugs

**Marjan Motamedi and Forozan Sasanipoor**

Department of Medical Parasitology and Mycology, School of Medicine, Shiraz University of Medical Sciences

**Objectives:** Over the past decades, many pathogenic fungi, such as bacteria, have been shown to have the ability to form biofilms, and biofilm formation is an important virulence factor for fungi. The aim of this study was to investigate the ability of biofilm formation by clinical dermatophyte isolates from different parts of the body, inhibition of biofilm formation by common anti-dermatophyte drugs, and finally accurate identification of isolates by sequencing.

**Materials and Methods:** The study population consisted of 50 dermatophyte isolates that were collected from 2019 to 2020 from patients referred to the two mycology laboratory of Shiraz, Iran. The isolates were identified by ITS gene region sequencing. The suspension (equivalent to 0.5 McFarland) of the isolates was used to evaluate the ability of the isolates to form biofilms and also the effect of itraconazole, terbinafine and griseofulvin at concentrations of 2–32 μg/mL on inhibiting biofilm formation. Two optical microscope and a scanning electron microscope were used to study the microscopic structure of biofilms formed on the surface of 96-well plate wells.

**Result:** The ability of biofilm formation by the isolates was 74% (37 cases). There is a statistically significant relationship between the ability of biofilm formation and the affected organ (*p* value = 0.029). In species isolated from tinea cruis and tinea unguim, we see 86.6% and 80% of biofilm formation ability, respectively. There was no statistically significant relationship between different levels of biofilm formation and identified species (*p* value = 0.32). In terms of the ability biofilm formation, can be said that out of 74% of isolates that had this ability, 22% belong to *Trichophyton mentagrophytes*, 20% belong to *Trichophyton rubrum* and the rest (32%), includes other species. Itraconazole and terbinafine in 70% of samples and griseofluvin in 66% have IC50 in concentration <2. The mean concentrations of IC50 in the three drugs itraconazole, terbinafine and griseofluvin are 3.78, 2.42 and 3.18 μg/mL, respectively. Spearman correlation coefficient between griseofulvin and biofilm formation ability is closer to one number. This relationship indicates that isolates with higher biofilm formation ability at higher concentrations of the drug have IC 50. Observation of the biofilm structure by optical microscope showed extensive mycelial growth with lateral hyphae surrounded by a polysaccharide-rich extracellular matrix in some parts of the plate surface, which is characteristic of biofilm formation. A highly focused scanning electron microscope provided three-dimensional images of biofilms at the plate surface, allowing better evaluation of fungal structures (Figures 1 and 2).

**Conclusions:** Almost all common dermatophyt species studied in this study were able to produce biofilms in vitro, which can play an important role in the pathogenesis of dermatophyte and failure to treat dermatophytosis infections. Terbinafine with favourable antibiofilm activity can be considered as the first option for the treatment of dermatophytosis. Further research to identify the biofilm ability of other dermatophyte species and to better understand the biofilm inhibitory activity of other antifungal drugs could provide new insights into the successful treatment of dermatophytosis infection.

## P033 Quantification of Biofilm Formation by *Candida auris* Isolates in a Tertiary Care Centre in South India

**Mary Kiran Danni ^1^, J Jayalakshmi ^2^, M K Renuka ^1^ and Anupma Jyoti Kindo ^1^**

^1^ Sri Ramachandra Institute Of Higher Education And Research
^2^ KMCH Institute of Health Sciences and Research

**Objectives:** The aim of this study is to quantify biofilm formation by *Candida auris* strains isolated from a tertiary care centre in South India.

**Materials & Methods:** 30 isolates of *Candida auris* were obtained from different patients from various samples like blood, urine and pus. The isolates were identified as *Candida auris* by MALDI TOF MS and further confirmed by colony PCR.

The strains will be grown on Sabourauds Dextrose Agar at 37 degree Celcius, over 24 h. The growth will be transferred in 0.85% saline matching the density of 0.5% MacFarland nephelometer standard tube no. 3, to get a concentration of 10^7^ yeast cells/mL. Then this solution will be diluted in Sabourauds Dextrose broth at a ratio of 1:20. 100 µL of this suspension will be incubated at 37 °C overnight in a sterile, polystyrene 96 well microtiter flat bottom plate, for production of biofilm.

After attachment phase, the non-adhered cells will be washed using Phosphate Buffered Saline. 200 µL of freshly prepared Yeast Nitrogen Broth will be added to each well, to facilitate biofilm formation. After 24 h incubation at 37 °C, XTT Reduction Assay will be performed. Negative control will be a well containing test medium alone without yeast cells. Reference strains will be included.

The biofilms will be washed with 200 microlitre of PBS to remove the adherent cells. Freshly prepared XTT- Menadione solution, mixed at a volume of 20:1 (42 µL) will be transferred to the wells along with PBS (158 microliter). The plates will be covered and incubated in the dark at 37 °C for 3 h.

100 µL of this solution will transferred to new 96 well microtiter plate and the colorimetric changes will be measured using microtiter plate ELISA reader.

**Results:** Receipt of some of the materials needed for the study have been delayed due to the pandemic. Hence results awaited.

**Conclusions:** The XTT reduction assay will be useful in measuring the metabolic activity of biofilm produced by different isolates of *Candida auris*.

## P040 Evaluation of 11 DNA Automated Extraction Protocols for the Detection of the 5 Mains *Candida* species from Artificially Spiked Blood

**Estelle Menu ^1,2^, Jordi Landier ^3^, Elsa Prudent ^2^, Stéphane Ranque ^1,2^ and Coralie L’Ollivier ^1,2^**

^1^ Aix Marseille Univ, IRD, AP-HM, SSA, VITROME
^2^ Laboratoire Hospitalo-Universitaire de Parasitologie-Mycologie, IHU Méditerranée Infection
^3^ SESSTIM, IRD, INSERM, Aix Marseille Université

**Objectives:** Molecular detection of *Candida* plays an important role in the diagnosis of candidemia, a major cause of morbidity and mortality. Sensitivity of this diagnosis is partly related to the efficiency of yeast DNA extraction. Candidemia diagnosis is often incorporated in a syndromic approach of bloodstream infection diagnosis. Therefore, it is essential to evaluate automated DNA extraction methods likely to be shared to detect the main agents of blood infections.

**Materials & Methods:** In this monocentric study, we investigated the suitability of eleven recent automated procedures for the extraction of *Candida* DNA from artificially spiked blood. Eight concentrations of artificially spiked blood were tested: 0 CFU/mL, 10 CFU/mL, 50 CFU/mL, 102 CFU/mL, 103 CFU/mL, 104 CFU/mL, 106 CFU/mL and 108 CFU/mL. We also compared the performance of these extraction protocols to detect the five main species implicated in candidemia: *Candida albicans. Candida glabrata* complex, *Candida parapsilosis, Candida tropicalis* and *Candida krusei*.

**Results:** The efficacy of the DNA extraction procedures to detect up to 10 CFU/mL of *Candida* spp. in blood samples ranged from 31.4% to 80.6%. The NucliSENS^TM^ easyMAG^TM^ procedure was the most efficient, for each species and each inoculum. Notably, it significantly outperformed the other procedures at the lower *Candida* inocula mimicking the clinical setting. Moreover, this study highlighted a heterogeneity in DNA extraction efficacy between *Candida* species. Up to five automated procedures were adequate for the DNA extraction of *Candida krusei*, whereas only one method yielded an adequate detection of low amount of *Candida tropicalis*.

**Conclusions:** This evaluation of 11 commercially available automated DNA extraction methods for the PCR diagnosis of candidemia, put within reach of laboratories about the choice of an appropriate methods in routine diagnosis. The NucliSENS^TM^ easyMAG^TM^ procedure displayed the best performances for detecting, even low concentration, of the five main *Candida* species DNA in whole blood, which complies with the requirement of the current syndromic diagnosis of bloodstream infections.

## P041 Longitudinal Evaluation of Plasma Cytokine Levels in Patients with Invasive Candidiasis

**Stefanie Wunsch ^1,2^, Christoph Zurl ^1,2,3^, Heimo Strohmaier ^4^, Andreas Meinitzer ^5^, Jasmin Rabensteiner ^5^, Wilfried Posch ^6^, Cornelia Lass-Flörl ^6^, Oliver Cornely ^7,8,9^, Gudrun Pregartner ^10^, Elisabeth König ^1^, Gebhard Feierl ^11^, Martin Hoenigl ^1,12^, Juergen Prattes ^1,2^, Ines Zollner-Schwetz ^1^, Thomas Valentin ^1^ and Robert Krause ^1,2^**

^1^ Division Of Infectious Diseases And Tropical Medicine, Department of Internal Medicine, Medical University of Graz
^2^ BioTechMed-Graz
^3^ Department of Paediatrics and Adolescent Medicine, Division of General Paediatrics, Medical University of Graz
^4^ Center for Medical Research, Medical University of Graz
^5^ Clinical Institute of Medical and Chemical Laboratory Diagnostics, Medical University of Graz
^6^ Institute of Hygiene and Medical Microbiology, Medical University of Innsbruck
^7^ Excellence Center for Medical Mycology (ECMM), Department I of Internal Medicine, Faculty of Medicine and University Hospital Cologne, University of Cologne
^8^ Chair Translational Research, Cologne Excellence Cluster on Cellular Stress Responses in Aging-Associated Diseases (CECAD), Faculty of Medicine and University Hospital Cologne, University of Cologne
^9^ Clinical Trials Centre Cologne (ZKS Köln), Faculty of Medicine and University Hospital Cologne, University of Cologne
^10^ Institute for Medical Informatics, Statistics and Documentation, Medical University of Graz
^11^ Diagnostic & Research Institute of Hygiene, Microbiology and Environmental Medicine, Medical University of Graz
^12^ Division of Infectious Diseases and Global Public Health, Department of Medicine, University of California San Diego

**Objectives:** Interleukin (IL) 17A plays a decisive role in anti-*Candida* host defense. Previous data demonstrated significantly increased and time-dependent IL-17A values in candidemic patients compared to non-candidemic patients. The objective of the present study was the evaluation of IL-17A plasma levels and other cytokines involved in anti-*Candida* host defense as potential biomarkers for early anticipation of invasive *Candida* infection.

**Materials & Methods:** We evaluated levels and time courses of IL-17A, kynurenine, tryptophan, and other cytokines suggested to be involved in *Candida*-specific immunity (IL-6, IL-8, IL-10, IL-17F, IL-22, IL-23, interferon-γ, tumor necrosis factor-α, Pentraxin-related protein 3, transforming growth factor-β) in patients with invasive candidiasis (IC (other), IC (true)) compared to bacteremic patients (*Staphylococcus aureus. Escherichia coli*) and healthy controls (from previous 4 days up to day 14 relative to the index culture (−4; 14)). Statistical analyses were performed for the total study population (main analysis), as well as without immunocompromised patients or patients with hematologic malignancies (sensitivity analysis).

**Results:** IL-17A levels were significantly elevated in all patient groups compared to healthy controls (Figure 1). In patients with IC, the highest IL-17A values were measured around the date of index sampling (−1; 2) [median 8.8, IQR 5.4–17.3 pg/mL], compared to significantly lower levels prior and after sampling the index culture. Candidemic patients showed significantly higher IL-17A values compared to IC (other) at time interval (−1; 2) and (3; 7). No significant differences in IL-17A levels could be observed for IC patients compared to bacteremic patients. Interestingly, levels of TGF-β were shown to be significantly elevated in patients with IC (other) compared to bacteremic patients for time intervals (−1; 2), (3; 7) and (8; 14). In contrast, patients with true candidemia only presented significantly elevated TGF-β values when compared to patients with *E. coli* bacteremia [for time intervals (−4; −2), (−1; 2) and (3; 7)], but not *S. aureus* bacteremia. However, exclusion of immunocompromised patients and patients with hematologic malignancies (sensitivity analysis) resulted in significant differences of TGF-β levels between IC (true) and bacteremic patients (both *S. aureus* and *E. coli*) for time intervals (−4; −2), (−1; 2) and (3; 7), with significantly higher values in candidemic patients.

**Conclusions:** We did not observe a discriminative competence between fungal and bacterial infections for both IL-17A and kynurenine in this study. Following this, IL-17A may be valuable as a biomarker for either fungal or bacterial blood stream infection rather than solely for invasive *Candida* infection. Further, we detected significantly elevated TGF-β levels in patients with IC compared to bacteremic patients, proposing a potential significance of TGF-β for differentiation between bacterial and *Candida* infections. However, larger studies are warranted to investigate this association.


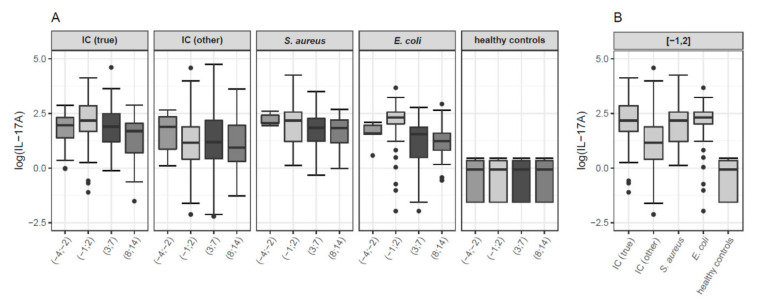


**Figure 1.** Boxplots of interleukin 17A (IL-17A) values of the investigated study groups for each time interval (**A**), and with focus on time interval (−1; 2) (**B**) for the total study population. IL-17A values (pg/mL) are depicted on the logarithmic scale. In the boxplots, median values are depicted as bold line and the box spans from first to third quartile; whiskers extend to a maximum of 1.5 times the interquartile range (IQR, third minus first quartile) out from the respective box end; all remaining values are indicated as dots. Time intervals represent study days. Time interval (−1; 2) includes blood samples collected one day prior until up to two days after collection of the index sample. IC = invasive candidiasis. IC (true) = patients with true candidemia. IC (other) = patients with candidemia of unclear significance (positive blood cultures from central veins only), proven IC other than candidemia, and patients with probable and possible IC. S. aureus = patients with S. aureus bacteremia. E. coli = patients with E. coli bacteremia.

## P042 Development and Application of an Intercalating Dye-Based PCR for the Diagnosis of Mucormycosis

**Juliette Guitard ^1^, Alexandre Godmer ^2,3^, Jeanne Bigot ^1^, Sandra Vellaissamy ^4^, Sophie Thorez ^4^ and Christophe Hennequin ^1^**

^1^ Sorbonne Université, Inserm, Centre de Recherche Saint-Antoine, CRSA, AP-HP, Hôpital Saint-Antoine, Service de Parasitologie-Mycologie
^2^ Sorbonne-Université, Hôpital Saint-Antoine, Département de Bactériologie
^3^ Sorbonne Université, INSERM, U1135, Centre d’Immunologie et des Maladies Infectieuses, Cimi-Paris
^4^ APHP, Hôpital St. Antoine, Laboratoire de Parasitologie Mycologie

**Objectives:** Early and specific diagnosis is crucial to manage the therapeutic strategy of mucormycosis. The sensitivity of conventional diagnosis is limited and no fungal biomarker is currently available. Thus, molecular DNA detection appears as the most promising approach for this diagnosis. This study aimed to assess an in-house pan-Mucorales real-time PCR to detect a wide variety of Mucorales species.

**Materials & Methods:** The method relies on the optimization of the primers sequence and the use of a fluorescent intercalating dye in a PCR targeting the rDNA operon. Analytical performances were assessed using 17 Mucorales strains (8 genera, 11 species) and 17 non-Mucorales strains (5 genera, 10 species). Technical challenges were realized with an external quality control program and 23 sera drawn from at-risk patients with a definitive diagnosis of proven mucormycosis. A clinical evaluation of the method was performed after its routine use for a 24-month period.

**Results:** Analytical assessment showed a 100% sensitivity and specificity. The limit of quantification ranged between 28 and 218 fg/µL depending on the species tested. Results obtained with the external quality control program and with the 23 sera drawn from at-risk patients with a definitive diagnosis of proven mucormycosis were 100% concordant.

364 clinical specimens from 166 at-risk patients were tested. According to EORTC classification (without taking into account the PCR results), 1 patient presented a proven mold infection of unknown origin, 6 and 1 patients presented a proven and a probable mucormycosis, respectively. 126 patients presented a possible mucormycosis, among them 119 presented an alternative diagnosis to explain clinical signs, and 7 had no alternative diagnosis.

A PCR test was positive for 22 specimens from 11 patients with 7 proven/probable mucormycosis, 1 proven mold invasive infection and 3 possible mold invasive infections. Considering only probable and proven mold infection, both clinical sensitivity and specificity were calculated at 1. Considering the 7 possible mucormycosis infection with no alternative diagnosis, sensitivity and specificity were calculated at 73.3% (IC95: 48.1% to 89.1%) and 100% (IC95: 97.5% to 100%), respectively.

Furthermore, among the 10 patients with proven/probable Mucormycosis and tested for Mucorales qPCR on serum, only 2 had a positive result. Both had a positive direct examination in biopsy specimens and died 11 and 19 days with an uncontrolled mucormycosis infection. Among the 8 patients with negative serum qPCR, 4 survived, and 3 died with a controlled mucormycosis infection.

**Conclusions:** This in-house real-time PCR represents a fast and reliable adjunctive tool for the diagnosis of mucormycosis.

## P043 Serum (1,3)-β-D-Glucan Cannot Reliably Exclude Pneumocystis Pneumonia in Patients with Hematological Malignancies

**Yohann Le Govic ^1,2^, Baptiste Demey ^1,2^, Cécile Pauc ^1^, Céline Damiani ^1,2^ and Anne Totet ^1,2^**

^1^ Laboratoire de Parasitologie-Mycologie, Centre de Biologie Humaine, CHU Amiens-Picardie
^2^ Agents Infectieux, Résistance et Chimiothérapie (AGIR), UR 4294, Université de Picardie Jules Verne

**Objectives:** *Pneumocystis* pneumonia (PCP) is a potentially life-threatening respiratory infection caused by the fungus *Pneumocystis jirovecii*. Accurately diagnosing PCP remains challenging, especially in HIV-negative patients who usually harbor lower pulmonary fungal loads than HIV-positive individuals. Given the invasiveness of the procedure employed for respiratory sampling and the expertise needed for interpreting *P. jirovecii* detection in the airways, there is a growing interest in using serum (1,3)-β-D-glucan (BG) assay as a screening test in the flowchart to diagnose PCP. Because of its high sensitivity, several studies have suggested that a negative result is sufficient for ruling out PCP. However, other reports indicated that BG testing has a limited sensitivity in HIV-negative PCP patients. The main objective of this study was to clarify the usefulness of serum BG as a tool to exclude PCP diagnosis in diverse HIV-negative patient populations. We also investigated the correlation between *P. jirovecii* burden in respiratory samples and serum BG levels.

**Materials & Methods:** We conducted a 10-year, monocentric retrospective study, of all HIV-negative adult patients with microscopically proven PCP and for whom a BG result was available. Serum BG levels (expressed in pg/mL) were measured using the commercialized Fungitell^®^ assay (Associates of Cape Cod, Inc.). Pulmonary fungal loads (expressed in Ct) were determined by an in-house real-time polymerase chain reaction.

**Results:** A total of 39 HIV-negative patients were enrolled in the study. Of them, 11 (28.2%) presented with hematological malignancies (HM group). Among the 28 (71.8%) remaining patients without HM (non-HM group), the most common underlying condition for the development of PCP was solid organ transplantation (n = 16, 41%), followed by systemic disorders (n = 9, 23.1%) and cancers (n = 3, 7.7%). Overall, 34 patients tested positive for BG [sensitivity: 0.87 (95% CI: 0.73–0.94)]. This result is in line with previous publications that have studied BG as a diagnostic marker for PCP in HIV-negative individuals. Nevertheless, performances of this test were significantly different between the two patient groups, with a positivity rate of 7/11 [sensitivity: 0.63 (95% CI: 0.35–0.84)] in HM vs. 27/28 [sensitivity: 0.96 (95% CI: 0.82–0.99)] in non-HM (*p* = 0.017, Fisher’s exact test). Moreover, BG levels were significantly different between these two groups with a median titer of 211 pg/mL [IQR 1489] in HM vs. 3425 pg/mL [IQR 1891] in non-HM (*p* < 10^−3^, Mann-Whitney test). The fungal load was also significantly lower in the HM group with a mean Ct of 27.1 [range 19–34] vs. 21.8 [range 15–30] in non-HM patients (*p* < 10^−3^, Student’s test). Besides, serum BG level and fungal burden correlated poorly in all 39 patients, regardless of the underlying disease (Spearman’s correlation coefficient *p* = −0.37, *p* = 0.018).

**Conclusions:** For HIV-negative patients, the sensitivity of BG assay varies substantially according to the underlying condition predisposing to PCP. Especially, a negative result alone is not sufficient to eliminate a PCP diagnosis in HIV-uninfected patients with HM. Altogether, BG results must be interpreted carefully and need to take into consideration the various PCP risk factors.

## P044 The Life and Death of Identification Methods in Mycology

**Sybren De Hoog, Sarah Ahmed, Abdullah Al-Hatmi and Roxana G. Vitale**

Radboud UMC

The high mortality and morbidity associated with fungal infections necessitate the use of accurate and fast diagnostic assays to facilitate appropriate antifungal treatment. The incidence and the spectrum of these infections have dramatically increased during the past decades, mainly due to growing populations at risk. Despite tremendous developments in available technologies, routine diagnostics has largely remained unchanged. Histology and direct microscopic observation of fungal elements are as still the basis for detection of prevalent species. Molecular identification tools such as isothermal amplification assays are applicable for limited species groups. Numerous techniques are available, but few have found wide application. Advances have been implemented with real-time PCR and Maldi-Tof, and some serological and lateral flow assays adapted for a limited number of diseases. For identification of less common opportunists, sequencing of ribosomal genes has received wide application, particularly identification of the plethora of filamentous fungi received great benefit from modern technology. A novel problem is multilocus phylogenetic taxonomy, which is driven close to the limit of detection while no diagnostic parameters are made available. Molecular methods require regular updating with curated databases. This also holds true for Maldi-Tof, which is already successfully applied in yeasts where barcoding gaps between species tend to be larger. An innovative general protein-based method, high-resolution accurate-mass spectrometry is promising but needs large-scale practical testing. The continuous increase in the number of clinically relevant fungi poses a great challenge for the development of a broad-spectrum identification: the list of 720 species treated in the 4th edition of the Atlas of Clinical Fungi grows with about 30–40 species each year.

## P045 Biomarkers of Inflammation in Patients with Asthma with *Aspergillus* spp. Sensitization

**Yana Kozlova, Ekaterina Frolova, Larisa Filippova, Aleksandra Uchevatkina, Oleg Aak, Ekaterina Burygina, Galina Solovjeva, Natalya Vasilyeva and Nikolay Klimko**

North-Western State Medical University named after I.I. Mechnikov

**Background:** Exposure to *Aspergillus* spp. is considered an important risk factor for the development of severe asthma. Study of new immunological serum markers of asthma patients with *Aspergillus* sensitization will expand diagnostic capabilities and improve disease control.

**Objectives:** To assess the levels of conventional and additional markers of inflammation and to determine the features of immune response regulation in asthma patients with *Aspergillus* sensitization.

**Methods and Materials:** The study included 36 asthma patients with *Aspergillus* sensitization (Me age—49 ± 14 years), 57 asthma patients without *Aspergillus* sensitization (Me age—50 ± 15 years), and 25 patients with allergic bronchopulmonary aspergillosis (ABPA) (Me age—45 ± 16 years). ABPA diagnosis was made according R. Agarwal et al., (2013) criteria. The control group consisted of 30 healthy people, (Me age 45 ± 6 years). Levels of thymic stromal lymphopoietin (TSLP), thymus and activation-regulated chemokine (TARC), IL-8, number of eosinophils, levels of total IgE and specific IgE to *A. fumigates* were determined in the serum by enzyme immunoassays.

**Results:** In asthma patients with *Aspergillus* sensitization median values of total IgE and *Aspergillus* sIgE were 700.0 [200.0; 792.0] and 0.9 [0.55; 1.27] IU/mL, which are significantly higher than in asthma patients without *Aspergillus* sensitization (*p* = 0.0008 and *p* = 0.0001). The absolute number of eosinophils in asthma patients with *Aspergillus* sensitization was higher than in the control group (*p* = 0.000), and asthma patients without *Aspergillus* sensitization (0.35 [0.16; 0.53] 10^9^/L vs. 0.29 [0.18; 0.48] 10^9^/L; *p* = 0.34). The highest rates compared to all other groups were ABPA patients (*p* < 0.05). The levels of total IgE and *Aspergillus* sIgE were 1950.0 [1150.0; 2950.0] IU/mL and 2.20 [1.15; 7.13] IU/mL respectively, the absolute number of eosinophils was 0.52 [0.40; 0.96] 10^9^/L.

The concentration of TSLP in the serum of the examined groups did not differ. Significant differences in the TARC levels were found in ABPA patients (718.0 [610.0; 907.0] pg/mg) in comparison with the asthma patients with *Aspergillus* sensitization (510.0 [333.0; 874.5] pg/mg, *p* = 0.03) and asthma patients without *Aspergillus* sensitization (202.5 [195.9; 256.0] pg/mg). The maximum concentration of periostin in the blood serum was recorded in patients with ABPA—31.89 [30.15; 40.87] ng/mL and was higher than in the asthma patients group (*p* = 0.0001) and the control group (*p* = 0.0000).

A positive correlation was found between the *Aspergillus* sIgE level with content of TARC, periostin and the number of eosinophils, and total IgE level (*p* < 0.001). A negative correlation was found between the levels of TARC and periostin and FEV1 index (*p* < 0.001).

**Conclusions:** The increased levels of TARC and periostin, along with conventional biomarkers of eosinophilic inflammation, indicates activation of the Th2 type of immune response in asthma patients with *Aspergillus* sensitization.

## P046 Development of a Real-Time PCR Assay to Identify *Cryptococcus neoformans* and *Cryptococcus gattii* Species Complexes

**Enoch Tay ^1,2^, Sharon Chen ^1^, Wendy Green ^1^, Ronald Lopez ^1^ and Catriona Halliday ^1^**

^1^ Centre For Infectious Diseases and Microbiology Laboratory Services, New South Wales Health Pathology
^2^ Research Education Network, Western Sydney Local Health District

**Objectives:** *Cryptococcus neoformans* and *Cryptococcus gattii* are the principle causative agents of cryptococcosis. Differences in epidemiological and treatment approaches emphasize the importance of distinguishing between the two species complexes. Whilst culture, histopathology and cryptococcal antigen (CRAG) detection are sensitive and simple, molecular methods are potentially more rapid; however, commercial PCR assays do not distinguish between the species complexes. Here we developed a real-time PCR assay to detect and identify *C. neoformans* and *C. gattii* directly from clinical specimens.

**Materials & Methods:** We targeted the mitochondrial Cytochome B (*CytB*) gene, as it is multicopy with sufficient homology to detect both *C. neoformans* and *C. gattii* using a single primer pair, yet has sufficient variation to differentiate between the two species complexes by melt curve analysis. The assay was first tested on the eight major cryptococcal molecular types (VNI-IV and VGI-IV). The assay performance was then evaluated using 197 specimens (fresh, and paraffin-embedded tissue (FFPE), cerebral spinal fluid (CSF), induced sputum (IS), bronchoalveolar lavage (BAL) fluid and fine needle aspirates (FNAs)) from patients with cryptococcosis (n = 48) or alternate respiratory tract infections. Assay performance was compared with culture and other methods including panfungal (ITS-directed) PCR.

**Results:** The *CytB*-directed assay detected all 8 *C.neoformans*/*C. gattii* genotypes (limit of detection ~10^1^ CFU/mL); there was no cross-reactivity with other respiratory pathogens. High resolution melt curve analysis demonstrated *C. neoformans* (VNI-VNIV) and *C. gattii* (VGI-VGIV) isolates had a melting point of 81 °C and 79 °C, respectively. Of 197 specimens, 46 tested positive for either *C. neoformans* (n = 31) or *C. gattii* (n = 15) (Table 1). PCR-negative specimens (n = 151) encompassed a range of specimens including IS and BAL submitted for *Pneumocystis jirovecii* infection. The assay sensitivity (95.8%) compares favorably with other diagnostic tests with only 2 specimens (one FFPE and CSF) from patients with cryptococcosis testing PCR-negative. Notably, the PCR (turn-around-time 4 h) enabled diagnosis and species identification in all 17 FFPE tissue samples vs. histology (13/17) and culture (0/17); panfungal PCR achieved diagnosis in 16/17 cases (turn-around-time-time ~72 h).

**Table 1.** Results of *Cryptococcus*-specific PCR compared with other diagnostic modalities.


**Specimen Type**

**Number**

**Culture Positive**

**Cryptococal Antigen**

**Histology**

**Panfungal PCR**

**Targeted PCR**

***C. neoformans***

**Targeted PCR *C. gattii***
FFPE Tissue17N/AN/A1316152CSF1223N/A1066BAL63N/AN/A324FNA62N/AN/A533IS30N/AN/A030Not Specified21N/AN/A220
**TOTAL**

**46**

**8**

**3**

**13**

**37**

**31**

**15**


**Conclusions:** The *Cryptococcus*-specific PCR assay is a rapid (4 h) sensitive method for detecting and differentiating *C. neoformans* and *C. gattii* species complexes in clincial samples. The assay is compatible with use on non-sterile specimens which often contain other fungal flora.

## P047 Diagnosis of Invasive Fusariosis by Detecting Circulating DNA: Retrospective Analysis of 10 Proven Cases

**Sarah Dellière ^1^, Marcela Sabou ^2^, Cécile Angebault ^3^, Majorie Cornu ^4^, Samia Hamane ^1^, Marie-Elisabeth Bougnoux ^5^, Juliette Guitard ^6^, Françoise Botterel ^3^, Stéphane Bretagne ^1^ and Alexandre Alanio ^1^**

^1^ Hôpital Saint-Louis
^2^ Centre Hospitalo-Universitaire de Strasbourg
^3^ Hôpital Henri-Mondor
^4^ Centre Hospitalo-Universitaire de Lille
^5^ Hôpital Necker
^6^ Hôpital Saint-Antoine

**Objectives:** *Fusarium* spp. are plant pathogens and opportunistic pathogen in severely immunocompromised (haematological malignancy, neutropenia, solid organ transplantation, …) and severely burned patients. Invasive fusariose often disseminates and mortality remains high partly due to delayed diagnosis in the absence of a positive culture. The aim of our study is to evaluate the detection of circulating *Fusarium* spp. DNA by quantitative PCR (qPCR) for early diagnosis of invasive infection.

**Materials & Methods:** This is a multicentric (6 centers) retrospective study that evaluates an in-house qPCR designed and optimised to identify all *Fusarium* specie complex in serum or plasma. A total of 10 patients diagnosed with proven fusariosis according to EORTC/MSGERC criteria between 2015 and 2021 were included. Patients with other invasive fungal diseases (aspergillosis, n = 4; mucormycosis, n = 4 and candidemia, n = 3) or without invasive fungal disease (IFD) (n = 10) were included as controls.

**Results:** No crossed-reactions were detected using DNA were detected using DNA extract from 81 opportunistic fungi. All *Fusarium* strains from five specie complexes (i.e., complex *oxysporum, solani, fujikuroi, dimerum, incarnatum*) known to be implicated in invasive fungal disease were detected. Limit of detection was evaluated to 3.2–32 femtogram/µL depending on the species. Circulating DNA was detected in 9 patients out of 10. Detection was possible up to 12 days (mediane = −5 [−12;7]) before the diagnosis was confirmed by positive blood culture or biopsy. Available biopsy (n = 2) of subcutaneous nodules from disseminated fusariosis allowed the amplification of *Fusarium* spp. with the qPCR. In two patients with follow up specimens, qPCR was still positive at day 3 and day 8 after introduction of voriconazole, respectively. Circulating DNA could not be detected in a patient with invasive fusariosis due to *Fusarium verticilloides* but no serum from the exact day of positive blood culture was available. However, extracted DNA from the strain isolated could be amplified and no mismatch was identified by sequencing the target. For specificity, qPCR was negative for all other IFD tested and IFD-free control patients.

**Conclusions:** We developed and validated a pan*Fusarium* qPCR assay in serum/plasma with a sensitivity and specificity of 90 and 100%, respectively. Considering the retrospective nature of this study using suboptimal quantity of stored serum/plasma leftovers, sensitivity is probably underestimated and will be further evaluated with a prospective study in the futur. Similarly to Mucorales qPCR, detection of circulating Fusarium DNA seems to be a non-invasive, sensitive and specific tool that could facilitates early diagnosis and treatment monitoring of invasive fusariosis.

## P048 Usefulness of BD BACTEC Mycosis IC/F Vials in the Diagnosis and Monitoring of Fungemia

**Laetitia Laroche ^1^, Victor Mercier ^1,2^ and Milène Sasso ^1,2^**

^1^ CHU Nîmes, Laboratoire Microbiologie
^2^ MIVEGEC, Univ Montpellier, CNRS, IRD

**Objectives:** The objective of this study was to determine the usefulness of the BD BACTEC Mycosis IC/F vial (Becton Dickinson) in the diagnosis and monitoring of fungemia.

**Materials & Methods:** A retrospective study of all fungemias detected by BD BACTEC Mycosis IC/F vials and/or BACTEC™ Plus Aerobic and BD BACTEC™ Lytic Anaerobic vials was conducted between 2013 and 2020.The type of vials used with the time of positivity, the fungal species involved and the presence of an antifungal treatment were collected.

**Results:** Between 2013 and 2020, 275 fungemias were diagnosed with BACTEC™ Plus Aerobic/BD BACTEC™ Lytic Anaerobic and/or BD BACTEC Mycosis IC/F blood culture bottles.In these cases, 95.6% were candidemias, with *Candida albicans* isolated in the majority (50.9%). For 20.7% of fungemic patients (57/275), one or more BD BACTEC Mycosis IC/F vials were collected, for a total of 78 vials over this period. Fungemia was diagnosed in 42 patients with the BD BACTEC Mycosis IC/F vial (alone or in combination) and in 15 patients only with the BACTEC™ Plus Aerobic and/or BD BACTEC™ Lytic Anaerobic vials.

From the 78 BD BACTEC Mycosis IC/F vials, 56 were collected simultaneously with the BACTEC™ Plus Aerobic and BD BACTEC™ Lytic Anaerobic vials.

In 44.6% of cases (25/56), all vials collected simultaneously were positive for yeast.In these cases, the time to positivity was favorable to the use of the BD BACTEC Mycosis IC/F vial for the initial diagnosis of fungemia (without antifungal treatment), with a mean time to positivity of 12.2 h less (32.0 h vs. 44.2 h).However, for fungemia monitoring or in case of antifungal treatment, the BACTEC™ Plus Aerobic and BD BACTEC™ Lytic Anaerobic vials showed better sensitivity, indeed they were found to be positive a mean of 55.7 h before the BD BACTEC Mycosis IC/F vials (33.7 h vs. 89.4 h).

In 30.4% of cases (17/56), the BD BACTEC Mycosis IC/F vial was negative with BACTEC™ Plus Aerobic and BD BACTEC™ Lytic Anaerobic vials positive for yeast.Fifteen of these 17 vials were collected under antifungal treatment.

In 25% of cases (14/39), only the BD BACTEC Mycosis IC/F vial was positive for yeast, with the BACTEC™ Plus Aerobic and BD BACTEC™ Lytic Anaerobic vials either negative (n = 5) or positive for bacteria (n = 9).

**Conclusions:** This study demonstrated the usefulness of using BD BACTEC Mycosis vials for the diagnosis of fungemia by providing earlier diagnosis compared to BACTEC™ Plus Aerobic and BD BACTEC™ Lytic Anaerobic vials. However, for patients on antifungal therapy, the BACTEC™ Plus Aerobic and BD BACTEC™ Lytic Anaerobic vials are most appropriate.In addition, some fungemia were detected either only on the BD BACTEC Mycosis IC/F vial or only on the BACTEC™ Plus Aerobic/BD BACTEC™ Lytic Anaerobic vials showing the interest of the combined use of these types of vials in optimizing fungemia diagnosis.

## P049 Prospective Evaluation of the GenMark Dx ePlex® Blood Culture Identification Fungal Pathogen Panel

**Jeremy Meeder ^1^, Derek Moates ^1^, Stefania Carmona ^2^, Rachael A. Lee ^3^, Todd McCarty ^3^ and Sixto M. Leal, Jr. ^1^**

^1^ UAB Fungal Reference Laboratory, Department of Pathology, Division of Laboratory Medicine, University of Alabama at Birmingham
^2^ Department of Medicine, University of Alabama at Birmingham
^3^ Department of Medicine, Division of Infectious Diseases, University of Alabama at Birmingham

**Objectives:** The ePlex BCID Fungal Pathogen Panel (FP) utilizes electrowetting technology to detect the most common causes of fungemia (15 targets) in positive blood culture bottles within 90 min. Rapid detection of intrinsic resistance to fluconazole (*Candida krusei*) or echinocandins (*Cryptococus neoformans, C. gattii, Rhodotorula*), detection of organisms of public health significance (*Candida auris*), and rapidly progressive pathogenic molds (*Fusarium)* enables early optimization of antifungals and infection prevention measures.

**Methods & Materials:** In this prospective study, we evaluated the performance of the BCID-FP Panel compared to traditional standard of care culture and identification with the BioMerieux Vitek MS Matrix Assisted Laser Desorption Ionization (MALDI) Time of Flight mass spectrometry system. Samples submitted for standard of care testing in BioMerieux BacT/Alert resin FA/FN blood culture bottles on the BacT/Alert VIRTUO automated blood culture system with fungal elements on direct exam (n = 60) were included. These results were also compared against T2Candida® when available within +/− 24 h.

**Results:** The majority of fungi 58/60 (96.6%) identified by MALDI were represented on the FP, excluding *C. pararugosa* and *C. nivariensis* and most tests 56/60 (93.3%; 2 *C. albicans, C. lusitaniae, C. parapsilosis*) yielded valid results. The FP exhibited a positive percent agreement (PPA) of 54/56 (96.4%) for all samples and 54/54 (100%) for samples with organisms represented on the panel. Traditional culture yielded a single false positive *C. lusitaniae*. A subset of samples (n = 22) analyzed by both FP and T2 showed a PPA of 22/22 (100%) for the FP and 11/22 (50.0%) for the T2. The T2 yielded two false positive *C. albicans/C. tropicalis* (A/T) results and 10 false negative results in which both culture and the FP identified 3 *C. albicans*, 2 *C. glabrata*, 2 *C. krusei*, 2 *C. parapsilosis*, and 1 *C. tropicalis*. Detection of species intrinsically resistant to echinocandins (2 *C. neoformans*; 1 without a concurrent + antigen test) or fluconazole (3 *C. krusei*) represented opportunities for early optimization of antimicrobial therapy in 5/60 (8.3%) of samples.

**Conclusions:** The BCID-FP Panel provides rapid accurate detection of a broad range of fungal pathogens enabling high quality data driven optimization of antimicrobial therapy.

## P050 Prospective Evaluation of Serum Mucorales Quantitative PCR for Diagnosis of Mucormycoses: The Modimucor Study

**Laurence Millon ^1^, Denis Caillot ^2^, Ana Berceanu ^1^, Stephane Bretagne ^3^, Fanny Lanternier ^4^, Florent Morio ^5^, Valérie Letscher-Bru ^6^, Frédéric Dalle ^2^, Blandine Denis ^3^, Alexandre Alanio ^3^, David Boutoille ^5^, Marie-Elisabeth Bougnoux ^4^, Françoise Botterel ^7^, Taieb Chouaki ^8^, Amandine Charbonnier ^8^, Florence Ader ^9^, Damien Dupont ^9^, Anne-Pauline Bellanger ^1^, Steffi Rocchi ^1^, Emeline Scherer ^1^, Houssein Gbaguidi-Haore ^1^ and Raoul Herbrecht ^6^**

^1^ University Hospital of Besançon
^2^ University Hospital of Dijon
^3^ Lariboisière Saint-Louis Fernand Widal Hospitals, APHP
^4^ Necker Enfants Malades Hospital, APHP
^5^ University Hospital of Nantes
^6^ Hôpitaux Universitaires de Strasbourg
^7^ CHU Henri Mondor, AP-HP
^8^ University Hospital of Amiens
^9^ Hospices Civils de Lyon

**Objectives:** Early diagnosis and prompt initiation of specific antifungal treatment is essential to improve the prognosis of Mucormycosis. Our aim was to assess the performance of serum Mucorales quantitative PCR (qPCR) for the early diagnosis of mucormycosis.

**Materials & Methods:** Patient with clinical suspicion of invasive mold infection (n = 233, including 28 finally classified with mucormycosis) and patients diagnosed with mucormycosis (n = 15) were prospectively enrolled in nine teaching hospitals. Usual radiological and mycological procedures were used to investigate invasive mold infection. Serum were collected twice-a-week for Mucorales qPCR tests targeting *Lichtheimia, Rhizomucor* and *Mucor/Rhizopus*. Patients were classified having possible, probable or proven invasive mold infection according to EORTC/MSGERC criteria.

**Results:** Sensitivity, specificity, positive and negative likelihood ratios were 82.1%, 89.8%, 8.0 and 0.20, respectively. The first Mucorales qPCR-positive serum was observed 4 days in median (IQR, 0–9) before the sampling date of the first mycological or histological positive specimen, and one day in median (IQR, (−2)−6) before the first imaging sign. Negativity of Mucorales qPCR within 7 days after liposomal-amphotericin B initiation was associated with an 85% lower 30-day mortality rate (Adjusted Hazard Ratio = 0.15; 95% CI, 0.03–0.73; *p* = 0.02).

**Conclusions:** Serum Mucorales qPCR is a non-invasive technique with great performances that can help to trigger early targeted antifungal treatment. Follow-up of Mucorales DNA load in serum could also be helpful for therapeutic management. Our study argues for the inclusion of qPCR detection of circulating Mucorales DNA in the diagnostic strategy of mucormycosis, and in consensual definitions from EORTC/MSGERC.

## P051 YeastOne and Micronaut-AM Antifungal Susceptibility Assays Comparison

**Nicolas Pellaton ^1^, Guy Prod’hom ^1^, Dominique Sanglard ^1^, Frederic Lamoth ^1,2^ and Alix-T. Coste ^1^**

^1^ Institute of Microbiologie, University Hospital
^2^ Service of Infectious Disease, University Hospital

**Objectives:** Fungi infections are an important cause of death worldwide. Antifungal susceptibility testing of yeast pathogens helps clinicians to better adapt patient treatments. One commonly used assay method in diagnosis is Broth microdilution susceptibility testing (BMST). BMST methods and criteria of interpretations are defined by two major consortia: CLSI and EUCAST. In this study, we compared three BMST methods: YeastOne (interpreted with CLSI criteria) read visually and MICRONAUT-AM (interpreted with EUCAST criteria), read visually and by spectrophotometer.

**Materials & Methods:** As BMST protocols differ following the consortia, Minimal inhibitory concentration (MIC) obtained by CLSI or EUCAST methods could not be compared. Thus, categories based on the number of MIC dilutions around breakpoint or ECOFF/ECV reference values were established to evaluate the accuracy between the 3 methods. Overall, 97 strains from 19 yeast species were measured for 8 antifungal drugs, for a total of 776 observations.

All the *Candida albicans, glabrata, lusitaniae* and *auris* strains, were previously analysed for antifungal resistance genes mutations, and were part of pairs or triplets of isogenic strains isolated from a single patient along treatment.

In addition, in order to evaluate repeatability and reproducibility of the MIC measurement, three strains: ATCC 22019 (*C. parapsilosis*), ATCC 6258 (*C. krusei*), and SC5314 (*C. albicans*) were measured 10 consecutive days and 5 times the same day with the three methods.

**Results:** In a first time, assigning MIC to categories, we realized that only 1/3 of the MIC measured could be assigned using breakpoint. Another 1/3 of the MIC could be assigned using ECOFF/ECV, and unfortunately, 1/3 of the MIC could not be assigned due to lack of criteria in EUCAST and/or CLSI. Overall, only 2/3 of the MIC measurement could be analysed. The accuracy of the categories, between the 3 BMST appeared to be relatively low, showing the difficulty to compare the BMST results.

In a second time, we analysed the results of the *C. albicans* and *C. glabrata* strains using the antifungal genes sequences as gold standard. We thus highlighted an already known candins susceptibility interpretation discrepancy between CLSI and EUCAST methods for *C. albicans* and *C. glabrata*. We also observed some differences of interpretation and categorization for azoles drug, which might be attributable to consortia differences, but also to reading difficulties due to tolerance phenomenon. However, globally, both methods detect diminution of antifungal susceptibilities, and acquisition of resistance for both candins and azoles, in the pair or triplet of characterized *Candida* strains.

In a third time, we observed a better reproducibility with Micronaut-AM (read visually or by spectrophotometer) as compared to YeastOne.

**Conclusions:** This study demonstrated, the valuable use of both type of BMST, essentially for *C. albicans* and *C. glabrata,* but highlighted the difficulty to conduct conclusive analyses without antifungal gene sequence data as gold standard.

## P052 Serum Lateral Flow Assay with Digital Reader for the Diagnosis of Invasive Pulmonary Aspergillosis: A Two Center Mixed Cohort Study

**Matthias Egger ^1^, Juergen Prattes ^1^, Jeffrey Jenks ^3,4,5^, Robert Krause ^1^, Eduard Schulz ^2^, Johannes Boyer ^1^ and Martin Hoenigl ^1,3,4^**

^1^ Division of Infectious Diseases, Medical University of Graz
^2^ Division of Hematology, Medical University of Graz
^3^ Division of Infectious Diseases and Global Public Health, University of California San Diego
^4^ Clinical and Translational Fungal-Working Group, University of California San Diego
^5^ Division of General Internal Medicine, University of California San Diego

**Objectives:** Among non-traditional risk groups, including critically ill patients in the intensive care units (ICU) and those with severe *coronovirus* disease (COVID-19) infection, *invasive Apergillosis* (IA) continues to be accompanied with high morbidity and mortality. Diagnosis is difficult due to unspecific clinical/radiological findings and low sensitivities of molecular tests and serum *galactomannan* (GM) testing, as well as varying turn around time. More rapid and accurate diagnostics are needed. We evaluated performance of the *Aspergillus Galactomannan* lateral flow assay (LFA), a novel point of care (POC) test for diagnosing IA, in serum specimens from a mixed cohort with and without invasive aspergillosis.

**Materials & Methods:** In a mixed bicentric cohort of 122 patients with clinical suspicion of IA (55% without hematological malignancies) and a GM ordered, 122 serum samples were retrospectively analyzed. Samples were collected between 2015 and 2021 at the University of California San Diego and the Medical University of Graz. The *Aspergillus Galactomannan* LFA was performed according to the manufacturer’s instructions. Digital readout was performed by an automated cube cube reader that was included with the test kits and displayed in optical density index (ODI). *Aspergillus* disease was classified according to the revised EORTC/MSG criteria, and for those with severe COVID-19 according to the ECMM/ISHAM criteria. LFA ODI cutoffs of 0.5 ODI and 1.0 ODI were compared by calculating sensitivity and specificity for proven/probable IA vs. no IA.

**Results:** The majority of patients were male (63% male vs. 37% female) and median age was 57 years (range 22–88). The LFA produced valid results in all samples. One patient fulfilled criteria of proven IA, 27 probable IA or SARS-Cov2- associated pulmonary *aspergillosis* (CAPA), and 87 did not fulfill criteria for IA or CAPA. Seven patients were non-classifiable. Underlying hematological malignancy was present in 45%. At a 0.5 ODI cutoff, sensitivity and specificity were 78.6% and 90.5%, respectively. When increasing the cutoff to 1.0 ODI, the LFA had a decreased sensitivity of 50%, but increased specificity of 96.6%. The serum LFA tended to be more discriminatory in patients with underlying hematological malignancies (AUC 0.853, 95%CI 0.750–0.977) vs. those without hematological malignancy (AUC 0.677, 95%CI 408–975). A total of 38% were receiving mold active antifungal prophylaxis or treatment at the time of serum sampling, but this did not impact discriminatory power of the LFA.

**Conclusions:** Our study is the first to evaluate LFA performance in serum specimens in a mixed cohrt of mostly non-hematologic malignancy patients and found 79% sensitivity and 91% specificity for IA by using a cut off of 0.5 ODI. Importantly, concomitant mold active prophylaxis or treatment did not impact diagnostic performance. The LFA from serum may serve a role as a rapid test for the diagnosis of IA. Prospective evaluation in a clinical setting would be valuable.


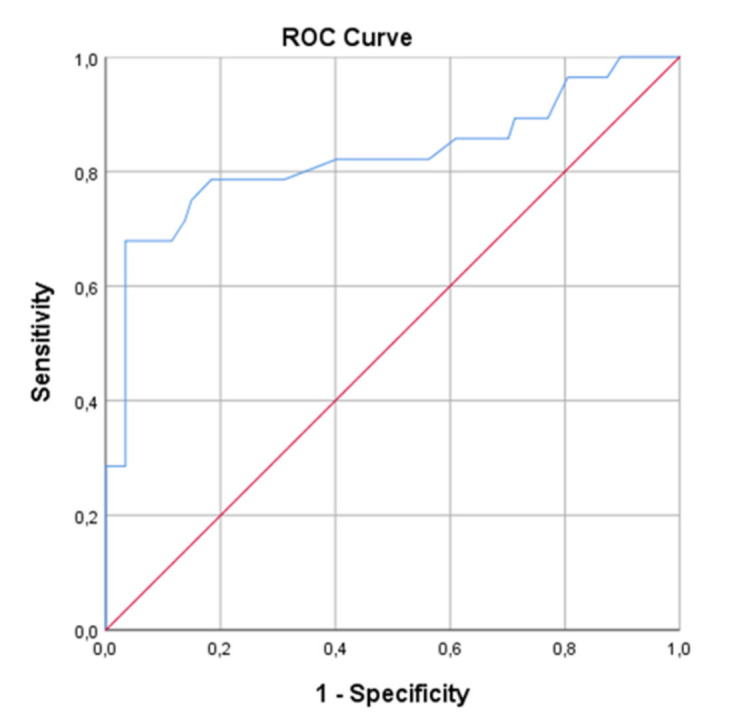


**Figure 1.** Receiver Operating Characteristics Analysis Curves for Serum Lateral Flow Assay for Diagnosing Proven/Probable Invasive aspergillosis or COVID-19 associated pulmonary aspergillosis vs. no aspergillosis in the overall Study Cohort.

## P053 Application of MALDI-TOF MS for the Identification of Clinical Yeast Isolates: Comparison of the Autof ms1000 and Bruker Biotyper Platforms

**Georgia Vrioni ^1^, Maria Drogari-Apiranthitou ^2^, Theodora Koliou ^1^ and Athanasios Tsakris ^1^**

^1^ Department of Microbiology, Medical School, National and Kapodistrian University of Athens
^2^ 4th Department of Internal Medicine, “Attikon” University General Hospital, National and Kapodistrian University of Athens

**Objectives:** Matrix-assisted laser desorption ionization-time of flight mass spectrometry (MALDI-TOF MS) has been established as a powerful tool for the fast and reliable identification of microorganisms, including yeasts. This study aimed to compare the performance of the recently developed Autof ms1000 (Autobio, Zhengzhou, China) with that of the Bruker Biotyper (Bruker Daltonics, Bremen, Germany) for identification of clinical yeast isolates.

**Materials & Methods:** A total of 87 yeasts recovered from various clinical specimens were used. The yeasts were identified at the species level with standard microbiological approaches including morphology and biochemical testing. *Candida auris* strains were identified by molecular method (*Candida auris* ITS2 gene, genesig® Standard Kit, Primerdesign^TM^ Ltd). The VITEK system (BioMerieux, version 8.02) was used for the identification of 21/87 strains. MALDI-TOF MS procedures were performed according to the manufacturers’ instructions. Library databases used were version 1.1.11 for Autof ms1000 and BDAL/8468 MSPs for Bruker Biotyper. Yeasts were transferred to the target plate by the formic acid method and by ethanol/formic acid extraction whenever results with both instruments were not reliable. Initial analysis of each strain was performed in duplicate, and the results with a higher range/score were used. Cutoff scores for reliable species/genus identification were 9.000–10.000/6.000–8.999 for Autof ms1000, and 2.00–3.00/1.70–1.99 for Bruker Biotyper.

**Results:** Both systems were able to identify all 87 yeast strains at the species level and the results obtained were identical (100% concordance). The only differences observed were in the score values. With the Autof ms1000 system 48/87 (55.2%) of the yeasts were identified at the species level with a high score (>9.000, reliable species identification), 37/87 were reliably identified only at the genus level, and 2 *C. auris* strains had a non reliable identification score. With the Bruker Biotyper 64/87 (73.6%) of the yeasts were reliably identified at the species level (score >2.00, reliable species identification), and 23/87 were reliably identified only at the genus level. When the results of the two MALDI-TOF MS platforms were compared with the VITEK system, identical results were obtained for 17/21 yeasts (80.9% concordance for both systems). VITEK failed to identify one *C. lusitaniae*, reliably identified by Bruker Biotyper. Three more strains, two identified as *Sacharomyces cerevisiae* and one as *C. albicans* by both MALDI-TOF MS systems, were identified as *C. famata, C. sphaerica* and *C. ciferrii* by VITEK, respectively. The Autof ms1000 system identified one of these strains reliably at the species level and three at the genus level. The Bruker Biotyper system reliably identified three of these isolates and one with reliability at the genus level.

**Conclusions:** Our study demonstrates that both, the Autof MS1000 and Bruker Biotyper systems can successfully identify most clinical yeast isolates, while performance is still highly dependent on the database and sample preparation protocols.

## P054 Performance of MALDI-TOF MS for Routine Identification of Fusarium Clinical Isolates: Comparison of the Autof ms1000 and Bruker Biotyper Platforms

**Georgia Vrioni ^1^, Theodora Koliou ^1^, Athanasios Tsakris ^1^ and Maria Drogari-Apiranthitou ^2^**

^1^ Department of Microbiology, Medical School, National and Kapodistrian University of Athens
^2^ 4th Department of Internal Medicine, “Attikon” University General Hospital, National and Kapodistrian University of Athens

**Objectives:** *Fusarium* species are rare but important human pathogens needing rapid and accurate identification. Yet their identification to the species level using phenotypic methods is difficult, requiring molecular sequencing. Matrix-assisted laser desorption ionization-time of flight mass spectrometry (MALDI-TOF MS) has been established as a powerful tool for the fast and reliable identification of microorganisms. This study aimed (i) to assess the accuracy of MALDI-TOF MS in the identification of *Fusarium* clinical isolates from Greece, and (ii) to compare the performance of the recently developed Autof ms1000 (Autobio Diagnostics, Zhengzhou, China) with that of the Bruker Biotyper (Bruker Daltonics, Bremen, Germany).

**Materials & Methods:** A total of 37 *Fusarium* strains recovered from various clinical specimens were used. Twenty-five (25/37) had been identified with molecular reference methods. MALDI-TOF MS procedures were performed according to the manufacturers’ instructions. Fresh colonies of the strains were transferred to the target plate by the formic acid method, or the ethanol/formic acid extraction whenever the initial results at species level were not relibable with both instruments. Cutoff scores for reliable species/genus identification were 9.000–10.000/6.000–8.999 for Autof ms1000 (Library database version 1.1.11), and 2.00–3.00/1.70–1.99 for Bruker Biotyper (BDAL Filamentous Fungi/577 MSPs). Analysis of each strain was performed in duplicate, and the results with a higher range/score were used.

**Results:** Identification results were obtained for 36 of the 37 *Fusarium* strains (97.3%), by both systems. With Autof ms1000 16/36 (44,4%) strains had a high identification score (>9.000) while with Bruker Biotyper 25/36 (69.4%) strains had a score of >2.00. The identification results with a non-reliable score were 5/36 (13.9%) for Autof ms1000 and 1/36 (2.8%) for Bruker Biotyper. However, 28/36 strains gave identical results by both systems, at least at the species complex (SC) level (77.8% concordance) and the differences were between the species *F. proliferatum, F. verticillioides* and *F. oxysporum*, known for their pattern similarities. Of the 25 molecularly identified strains, 16 (64%) were correctly identified by the Autof ms1000, 14 at species level and 2 at SC level. With Bruker Biotyper, 20/25 (80%) were correctly identified, 17 at species level and 3 at SC level. Three strains (1 *F. metavorans* and 2 *F. brachygibbosum*) could not be identified by either system, as they are new emerging species. Four *F. verticillioides* strains were misidentified, either as *F. proliferatum* (3 strains by Bruker Biotyper, 2 by Autof MS1000), both belonging to *F. fujikuroi* SC, or *F. oxysporum* (2 strains by Autof MS1000).

**Conclusions:** Both systems produced satisfactory results. The Autof MS1000 and Bruker Biotyper platforms could be equally utilised to identify clinical *Fusarium* isolates at SC level, although the Bruker Biotyper appeared to perform slightly better for the set of *Fusarium* strains used. Frequent updates of the mass spectrometry databases will enable more accurate identification of new emerging species, or species with similar patterns but potentially different antifungal susceptibility profiles.

## P055 Adaption of an Histoplasma RTqPCR Assay for Rapid Diagnosis of Disseminated Emergomycosis

**Aude Sturny Leclère ^1^, Tsidiso Maphanga ^2^, Dea Garcia Hermoso ^1^, Nelesh P. Govender ^2^ and Alexandre Alanio ^1^**

^1^ Institut Pasteur
^2^ NICD

**Objective:** A new reverse transcriptase quantitative PCR (RTqPCR) assay was recently designed and validated in a French cohort of patients with suspected histoplasmosis (907 patients; 1319 specimens analyzed). In laboratory testing, the assay had a very low limit of detection (20 copies) and a 100% analytical specificity against 114 different fungal species including *Coccidioides* spp., *Talaromyces marneffei, Paracoccidioides brasiliensis, Nanniziopsis obscura*, and *Emergomyces crescens*. The clinical validation demonstrated a 98% sensitivity (43/44 patients detected) in EORTC/MSG-proven cases and 99% specificity. Moreover, 92% of patients with a progressive disseminated histoplasmosis in immunocompromised patients were detected in blood specimens using this RTqPCR assay. This assay allowed diagnosis of cases of *Histoplasma capsulatum* var. *capsulatum* and *Histoplasma capsulatum* var. *duboisii*.

**Material and methods:** We first aimed to assess the analytical specificity of this *Histoplasma* RTqPCR assay on closely-related fungi responsible for similar diseases in South Africa including emergomycosis and blastomycosis.

**Results:** The *Histoplasma* RTqPCR assay allowed detection of a standardized concentration of *Emergomyces africanus, Emergomyces pasteurianus, Blastomyces percursus, Blastomyces emzantsi* DNA (0.1 ng/µL) with ∆Cq values between 4 to 11 compared to *Histoplasma* DNA (Table). This difference means that the assay is able to detect *Emergomyces* and *Blastomyces* DNA but with performance decreased by 20 to 1000 times as compared to *Histoplasma* DNA detection. We then designed a RTqPCR assay at the same locus (mtSSU) with primers and probes specific to *E. africanus* and observed an increased specificity for *E. africanus* compared to *Blastomyces* spp. and *Histoplasma* spp. with a ∆Cq value of 7 to 12 Cq value (decreased performance by 200 to 10,000 fold for *Blastomyces* and *Histoplasma* DNA detection compared to *Emergomyces* DNA).

**Conclusions:** Using both RTqPCR assays in patients with suspected disseminated histoplasmosis or emergomycosis among people living with HIV may help distinguish between these clinically-similar diseases by analysis of the Cq value.

## P056 Determining Cut-Off of *Aspergillus*-Specific Galactomannan Index in Tracheal Aspirate Samples in COVID-19 Associated Pulmonary Aspergillosis Patients from Pakistan

**Joveria Farooqi, Syed Ahsan Ali, Muhammad Irfan, Sadaf Zaka, Imtiaz Soomro, Faheem Naqvi and Kauser Jabeen**

Aga Khan Univeristy

**Objectives:** According to the consensus definition for COVID-19 associated pulmonary aspergillosis (CAPA), the microbiological criteria requires bronchoalveolar lavage fluid (BALF) for measurement of *Aspergillus*-specific Galactomannan Index (GMI), Aspergillus PCR or culture. In our setting, very few patients undergo bronchoscopy and BALF collection due to severe hypoxia in COVID-19 patients and biosafety concerns. Hence, we set out to determine the cut-off for GMI in easily accessed respiratory samples which could distinguish between CAPA and non-CAPA patients.

**Methods:** CAPA and non-CAPA patients were identified from hospital and laboratory database of Aga Khan University Hospital. Diagnostic criteria used were adapted from the 2020 ECMM/ISHAM consensus criteria by Koehler et al., modified to include isolation of *Aspergillus* species from TA and/or serum GMI >0.5, in the presence of clear radiological and clinical evidence and confirmed COVID-19 patients diagnosed on SARS CoV2 PCR to define possible and probable CAPA. GMI was measured using Platelia Aspergillus® (Bio-Rad Laboratories, Marnes-la-Coquette, France). ROC curve and Youden’s J statistic was used to determine the cut-off and its sensitivity and specificity. Frequencies and proportions computed and multiple linear regression model built to determine the increase in GMI with different covariates.

**Results:** There were 62 (62.6%) CAPA cases [26/62 (42%) probable CAPA and 36/62 (58%) possible CAPA] and 37 (37.4%) non-CAPA cases. Nineteen (31%) CAPA patients had both *Aspergillus* culture positive and serum GMI >0.5, while 36 (58%) CAPA patients had samples which were culture positive for *Aspergillus* species only and 7 (11%) CAPA patients had serum GMI >0.5 only. The cut-off for GMI was determined as 1.61 [Youden = 0.482, AUC = 0.755 (0.653–0.857), sensitivity 88.7% and specificity 59.5%] when both possible and probable CAPA were taken as cases. The GMI cut-off increased to 3.55 [Youden = 0.427, AUC = 0.736 (0.638–0.833), sensitivity 96.2%, specificity 46.3%] when only probable CAPA labelled as cases. At GMI of 4.5 in non-bronchoscopic lavage, as suggested by Koehler et al, sensitivity was 72.6%, specificity 62.2% and Youden 0.347 for all cases, while 88.5%, 50.7% and 0.391, respectively for probable cases only. The multiple linear regression model (adjusted R-square 0.288, *p* < 0.001) showed a GMI increase of 3.05 (95% CI: 1.30–4.79, *p* = 0.001) with probable CAPA case, 0.96 (95% CI: −0.72–2.63, *p* = 0.261) with possible case and 2.90 (95%CI: 1.29–4.51, *p* = 0.001) when *Aspergillus* species were isolated from the same sample.

**Conclusions:** The cut-off for GMI in tracheal aspirates was determined to be >1.6 to detect CAPA cases with a high sensitivity. The specificity remained low, which warrants further exploration with non-CAPA samples. This could be due to increased background rates of chronic pulmonary diseases and airway colonisation in our population.

## P057 Genetic Characterization and Antifungal Susceptibility Profiles of Trichosporon Species Isolated from Iranian Clinical Samples

**Fatemeh Ahangarkani ^1^, Kiana Abbasi ^2^, Jacques F Meis ^3^ and Hamid Badali^4^**

^1^ Antimicrobial Resistance Research Center, Communicable Diseases Institute, Mazandaran University of Medical Sciences
^2^ Department of Microbiology, Zanjan Branch, Islamic Azad University
^3^ ECMM Excellence Center for Medical Mycology, Centre of Expertise in Mycology Radboudumc/Canisius-Wilhelmina Hospital
^4^ Department of Medical Mycology, School of Medicine, Mazandaran University of Medical Sciences

**Objectives:** Trichosporonosis is an emerging fungal infection caused by *Trichosporon* species, a genus of yeast-like fungi, which are frequently encountered in human infections ranging from mild cutaneous lesions to fungemia in immunocompromised patients. The incidence of trichosporonosis has increased in recent years, owing to higher numbers of individuals at risk for this infection. Although amphotericin B, posaconazole and isavuconazole are generally effective against *Trichosporon* species, some isolates may have variable susceptibility to these antifungals. Herein, we evaluated the species distribution, genetic diversity and antifungal susceptibility profiles of *Trichosporon* isolates in Iran.

**Materials & Methods:** The yeasts were identified by matrix-assisted laser desorption/ionisation time-of-flight mass spectrometry (MALDI-TOF MS). Phylogenetic analysis was performed based on amplified fragment length polymorphism (AFLP). The in vitro susceptibilities of eight antifungal agents were analysed using the Clinical and Laboratory Standards Institute broth microdilution methods.

**Results:** The isolates belonged to the species *T. asahii* (*n* = 20), *T. japonicum* (*n* = 4) and *T. faecale* (*n* = 3). A dendrogram of the AFLP analysis demonstrated that *T. asahii* and non-*asahii Trichosporon* strains (*T. japonicum* and *T. faecale*) are phylogenetically distinct. While voriconazole was the most active agent (GM MIC = 0.075 μg/mL), high fluconazole MICs (8 μg/mL) were observed for a quarter of *Trichosporon* isolates. The GM MIC value of amphotericin B for *T. asahii* and non-*asahii Trichosporon* species was 0.9 μg/mL.

**Conclusions:** The distribution and antifungal susceptibility patterns of the identified *Trichosporon* species could inform therapeutic choices for treating these emerging life-threatening fungi. Additional studies are needed for the understanding of epidemiology and antifungal susceptibility patterns of *Trichosporon* i n I ran. The current study is t he first report on *Trichosporon* s pecies in Iran. The p resented data on t he distribution, genetic diversity and antifungal susceptibility patterns of these *Trichosporon* i solates can inform therapeutic choices for fungi from this emerging life-threating pathogen.

## P058 Non-Culture Based Approach to Detect Azole Resistance in Patients at Risk of Invasive Aspergillosis (ERESAA-BM Study): Preliminary Findings

**Rose-Anne Lavergne ^1,2^, Florent Morio ^1,2^ and Patrice Le Pape ^1,2^**

^1^ Parasitology and Mycology Laboratory, Nantes University Hospital
^2^ EA1155, Nantes University

**Objectives:** Azole resistance in *Aspergillus fumigatus* is a worldwide and worrisome problem. At our center, we previously evaluated the prevalence of azole resistance by in vitro susceptibility testing in cystic fibrosis patients, but no data are available in patients at risk of invasive aspergillosis (IA) linked to low sensitivity of conventionnal approach (culture) to detect *Aspergillus* from respiratory samples of these patients. The main objective of this study was to evaluate the benefit of a molecular approach to identify *Aspergillus fumigatus* and to detect molecular markers associated with environmental azole resistance in respiratory samples from patients at risk of IA.

**Materials & Methods:** From February 2020 to January 2021, BAL supernatants from patients at risk of IA were prospectively collected and stored at −20 °C. In this non-interventional study, patients at risk of IA were defined by the prescription of at least one *Aspergillus* galactomannan antigen (GM) dosage on serum or BAL, whatever the results. BAL were routinely processed according to conventional methods (direct examination followed by cultures +/− azole susceptibility testing +/− GM dosage). At the end of the collection step, all BAL supernatants were analysed by molecular approach: DNA extraction from 1 mL of sample using the Stabilized Saliva DNA® kit and Maxwell® instrument (Promega) followed by detection and identification of *Aspergillus* and TR34, L98H, Y121F and T289A mutations, using the AsperGenius® kit (PathoNostics). Results of routine cultures and molecular tests were then compared.

**Results:** During this one-year collection period, 143 BAL supernatants (n = 114 patients) were included. Underlying diseases included solid organ transplantation (n = 55), hematological malignancies (n = 33) or others situations at risk of IA (n = 26). Analysis of the first 36 samples (n = 31 patients) allowed to detect *Aspergillus* DNA in 6 samples from 6 patients. Of these 6 positive samples for *Aspergillus* DNA, 3 samples were positive for *Aspergillus fumigatus* (Ct from 34.6 to 39.6). By comparison, two samples were positive for *Aspergillus* in culture: 1 *Aspergillus nidulans* species complex, detected by the Aspergenius® assay and 1 *Aspergillus fumigatus* species complex not detected by the Aspergenius® assay (neither *Aspergillus* nor *A. fumigatus* PCR). None of the resistance markers (TR34, L98, Y121 and T289) could be amplified in the 3 *A. fumigatus*-positive samples. GM dosages were performed on 12 of the 36 BAL samples and all were negative (index < 0.5).

**Conclusions:** These preliminary results highlight that conventional cultures and molecular methods are complementary tools which, when combined are able to increase the detection rate of *Aspergillus* in patients at risk of IA, in line with the current guidelines. Molecular detection of resistance markers needs sufficient amount of *A. fumigatus* DNA. These preliminary results will be consolidated with the analysis of the 107 remaining samples (n = 83 patients).

## P059 Accelerate Antifungal Susceptiblity Testing of *Aspergillus fumigatus* Isolates Sent to Mycology Reference Laboratories by Direct Inoculation of Spore Suspensions

**Jochem Buil ^1,2^, Marlou Tehupeiory-Kooreman ^1,2^, Willem Melchers ^1,2^ and Paul Verweij ^1,2^**

^1^ Department of Medical Mycology, Radboud University Medical Center
^2^ Radboudumc—CWZ Center of Expertise for Mycology

**Background:** Antifungal susceptibility testing (AFST) of clinical relevant *A. fumigatus* isolates is important to guide antifungal therapy, especially in regions were azole resistance is prevalent. However, the reference methods for AFST are not widely available. Sending isolates to mycology reference centers causes delay in obtaining the susceptibility results, as isolates need to be subcultered before performing broth microdilution. In order to accelerate AFST, we studied whether spore suspensions, that are sent in can be directly inoculated, thereby eliminating the subculture-phase.

**Material/methods:** We used two methods for AFST: In the standard “EUCAST method”, AFST was performed using spores from a maturated colony. In the “direct inoculation method”, AFST was performed using the spore suspensions that were either stored or sent from other laboratories directly without a subculturing phase. Three groups of isolates were analyzed. Group I: experimental analysis: 13 *A. fumigatus* isolates were selected of which 6 were azole resistant. Spore suspensions were prepared and AFST was performed daily after storing the isolates for 4 days at room temperature, and after 4 days for spore suspensions stored at 2–4 °C and at 28 °C. All measurements were performed in triplicate. Group II, retrospective analysis: 50 isolates that were sent to our laboratory as spore suspensions were tested using the direct inoculation method and stored at −70 °C in glycerol broth. The stored isolates were revived and AFST was repeated using the EUCAST method. Group III: prospective analysis: 30 isolates that were sent to our laboratory as spore suspensions were tested using the direct inoculation method and simultaneously using the EUCAST methods without storage at −70 °C. MICs based on the EUCAST and direct inoculation methods were compared.

**Results:** The MICs of azoles and amphotericin B for isolates from Group 1 strains from stored spore suspensions did not differ more than one twofold dilution step comparison to the standard EUCAST method. The susceptibility classification based on clinical breakpoints showed 99.9% concordance for azoles and amphotericin B. One isolate resistant (MIC 2–4 mg/L) for voriconazole, tested susceptible after 4 days storage in 1 replicate. In Group II, 100% concordance was seen for amphotericin B and voriconazole. For itraconazole, 5 strains that had MICs of 2–4 mg/L in direct inoculation method while the MIC was 0.5–1 mg/L in EUCAST method. For 2 isolates the MIC of posaconazole were 1–2 dilutions lower in EUCAST methods resulting in a change in interpretation. For Group III isolates, 99.2% concordance was found. One isolates with a voriconazole MIC of 2 mg/L using EUCAST methods had a MIC of 1 mg/L using direct inoculation.

**Conclusions:** We evaluated a strategy to accelerate AFST of *A. fumigatus* isolates sent to mycology reference laboratories. For isolates with MICs close to the breakpoints, small variations in measurements did result in a change in interpretation for individual drugs. However, this is also the case for repeated measurements performed in accordance to EUCAST guidelines. Our results indicate that the subculturing phase can be eliminated for spore suspensions up to 4 days for azoles and amphotericin B.

## P060 Comparison of the Analytical Performances of Two Commercial Real-Time PCR Kits with an In-House Real-Time PCR Assay for Diagnosing Mucormycosis

**Victor El Jammal ^1^, Charline Miossec ^2^, Damien Dupont ^1^, Martine Wallon ^1^, Meja Rabodonirina ^1^, Florence Persat ^1^ and Jean Menotti ^1^**

^1^ Lyon University Hospitals/Claude Bernard University
  ^2^ Lyon University Hospitals

**Objectives:** The prognosis of patients with invasive mucormycosis depends on early diagnosis and treatment initiation. As conventional diagnosis relying on histopathological and mycological direct examination combined with mycological culture lacks sensitivity and/or yields delayed diagnosis, an in-house real-time PCR has been proposed as an additional diagnostic tool and has shown to yield earlier diagnosis (Millon et al., 2016). As two commercial real-time PCR kits recently became available for the diagnosis of mucormycosis, our aim was to assess their performances when compared to the home-made PCR assay to detect and quantify the main genera of Mucorales involved in human diseases.

**Materials & Methods:** The MycoGENIE® Aspergillus-Mucorales spp. real-time PCR kit and the Fungiplex® Mucorales PCR kit were compared to an in-house real-time PCR assay using hydrolysis probes targeting *Mucor/Rhizopus, Lichtheimia*, and *Rhizomucor*. A standard curve was generated for all assays with serial dilutions of *Rhizopus arrhizus, Lichtheimia ramosa, Rhizomucor pusillus, Mucor circinelloides, Cunninghamella bertholletiae*, and *Syncephalastrum racemosum* DNA.

**Results:** The lower limit of detection of all assays was assessed to be between 0.5 and 5 fg/µL of *Rhizopus arrhizus. Mucor circinelloides, Lichtheimia ramosa*, and *Rhizomucor pusillus* DNA, except for *L. ramosa* that was not detected by Fungiplex. The serial dilutions of *R. arrhizus* DNA yielded mean threshold cycles (Ct) 0.78 and 1.97 higher for Fungiplex and for MycoGENIE than for the in-house PCR assay, respectively. For *M. circinelloides*, mean Ct values were 0.12 and 3.39 higher than for the in-house PCR assay for Fungiplex and MycoGENIE, respectively. For *R. pusillus*, mean Ct values were 0.44 and 0.11 higher than for the in-house PCR assay for Fungiplex and MycoGENIE, respectively. For *L. ramosa*, mean Ct value was 0.73 higher for MycoGENIE than for the in-house PCR assay. *Lichtheimia corymbifera* was detected by all 3 assays. *Cunninghamella bertholletiae* was detected only by MycoGENIE. *Syncephalastrum racemosum* was not detected by the 3 assays.

**Conclusions:** Although the lower limit of detection of all three assays was globally quite close, some limits were observed, especially for *L. ramosa* which was not detected by Fungiplex. This could be due to an insertion/deletion or mismatches between *L. ramosa* sequence and the designed primers and/or probe. We also found quite higher mean Ct values than with the in-house PCR assay, especially for *M. circinelloides* and *R. arrhizus* with MycoGENIE. Although both kits were deemed to detect all Mucorales, *C. bertholletiae* was not detected by Fungiplex and *S. racemosum* was not detected by MycoGENIE and Fungiplex. The unavailability of the designed primers and probe sequences precludes the ability for mycologists to predict the species that could be detected by commercial kits. Mycologists should therefore verify the sensitivity of detection of the clinically relevant species so as to be aware of the limits of commercial PCR kits.

## P061 Respiratory Symptomatic Patient with Adult Chronic Disseminated Paracoccidioidomycosis in the COVID-19 Era

**Larry Luber Martínez Rosado**

Latin American Research Team in Infectology and Public Health ELISAP, La Maria Hospital

**Objetives:** To present a clinical case of endemic mycosis presented as a respiratory symptomatic case that made it necessary to rule out COVID-19 and tuberculosis.

**Methods:** Case report.

**Results:** A 40-year-old man, farmer, with a history of heavy smoking and use of tetrahydrocannabinol (THC). He came to the clinic due to a clinical picture of 30 days of evolution due to odynophagia, halitosis, nocturnal sweating, unquantified weight loss, occasional dry cough. On physical examination he was feverish, whitish plaques were documented on the tonsils, and bilateral diffuse rales. The ELISA for HIV, the PCR for COVID-19 and the glycosylated hemoglobin were negative, the chest X-ray revealed generalized alteration of the lung parenchyma, thickening of the interstitium and patchy opacity in ground glass and subsegmental multifocal pneumonia in the right upper lobe segment. It was labeled as respiratory symptomatic, ruling out pulmonary tuberculosis. A community pneumonia scheme was indicated with ampicillin sulbactam plus clarithromizine, a microbiology was requested in sputum induced for tuberculosis, the evaluation by otorhinolaryngology revealed ulceronecrotic pharyngotonsillitis justifying tonsillectomy. The KOH validated in the tonsil biopsy was positive and the pathological anatomy showed severe acute and chronic inflammatory infiltrate, with granulomatous areas and multinucleated giant cells of the Langhans type surrounding areas of necrosis, special stains were performed, including methenamine silver being positive for structures consistent with paracoccidioidomycosis (Figure 1). Complementary studies such as immunodiffusion and complement fixation serology for fungi were reactive with a band of precipitate and reactive paracoccidioidin with dilution >64 respectively. Studies of tuberculosis and other mycoses were negative (Table 1). Management was started with itraconazole, scheduled for 6 months, therapy with which the patient responded favorably.

**Conclusions:** Paracoccidioidomycosis is a systemic systemic fungal disease endemic to territories, which occurs in a chronic and progressive manner that mainly affects the oropharyngeal mucosa, lungs and the reticuloendothelial system; performs both hematogenous and lymphatic dissemination, which is why any organ can be affected. This mycosis is acquired directly by inhalation of conidia from humid soils and high rainfall (1). In Colombia, the first case was described in 1949, with multiple presentations since then (2). Currently the world is going through a state of emergency due to the pandemic potential of SARS CoV2 COVID-19, the first case in our country was reported on March 6 and to date 3,432,422 million cases have been confirmed with 89–297 deaths, however, lethal endemic pathologies in our territory should not go unnoticed.

## P062 Introduction and Evaluation of Fujifilm Wako β-D-Glucan Test in the Mater Misericordiae Hospital

**Breda Lynch and Assumpta Killarney**

Mater Misericordiae Hospital

Category: Diagnostics.

**Objectives:** Fungal infections carry a high morbidity and mortality, are difficult to diagnose and expensive to treat. β-D-glucan (BDG) is a serological test used in the diagnosis of fungal infections and was previously available to the Mater as an external laboratory reference test. The main objective of this project was to repatriate laboratory BDG testing to be performed in-house. The expected outcomes of this project were to improve diagnosis or out-ruling of fungal disease for patients, improve turn around time from current 109 days to less than 3 days and reduce unnecessary antifungal use.

**Materials & Methods:** The Wako BDG Test was validated for usage prior to equipment procurement. Once in place, staff training was completed and an education session of the use of BDG presented to the Clinical Microbiology (CM) and antimicrobial stewardship (AMS) team. In-house BDG testing service was established and data collected from patient testing in MMUH from February–September 2020 via retrospective analysis of clinical microbiology notes and digital records. Information gathered included test indication; ordering clinician; patient impact of result; AMS impact including financial savings, if any; rationalisation of therapy. Information was analysed and compared to international guidelines.

**Results:** Total number of samples 259:88 positive tests, 171 negative. 250 (96.5%) were under the guidance of the CM or Infectious Diseases team.

Expected and delivered outcomes of introduction of BDG in-house testing;

- Improve diagnosis or out-ruling of fungal disease for patients.
  Achieved: Analysis demonstrates sensitivity 92.9%, specificity 99.3%, with a positive and negative predictive value of 96.3% and 98.6% respectively.
- Improve turn-around time from current 109 days to less than 3 days.
  Achieved: TAT now average 1 day, range <1–7 days with only 21 of 259 results taking >2 days.
- Reduce unnecessary antifungal use.
  Achieved: 32 patients had anti-fungal therapy rationalised based on a negative BDG result leading to a direct cost saving of €81,064.

**Conclusions:** BDG contributes to confirming the diagnosis of fungal infection: 25 patients had a positive BDG result which contributed to early diagnosis and treatment of a fungal infection.

BDG contributes to out-ruling fungal infections and rationalising therapy therefore is a valuable AMS tool. A total of 87 results contributed to a patient not being prescribed an empiric antifungal. This protected these patients from any potential adverse events that may have occurred. While there would be a cost-saving attributable to this, it is difficult to quantify.

With charity funding for equipment, we were able to process the tests in-house for €16,669.24 cheaper than sending externally. TAT for results improved from an average of 109 days to 1 day. Repatriation of BDG long term is cheaper for the laboratory as well as providing an improved service.

In conclusion, the introduction of BDG testing into MMUH has been a successful initiative that both improves patient care as well as being a financially worthwhile endeavor.

## P063 Galactomannan Antigen Testing for Diagnosis of Invasive Aspegillosis in Pediatric Haematological Oncology Patients’

**Annapurna Parida ^1^, Malini R. Capoor ^1^ and Amitabh Singh ^2^**

^1^ Department of Microbiology, Safdarjung Hospital and Vardhaman Mahavir Medical College
^2^ Department of Pediatrics, Safdarjung Hospital and Vardhaman Mahavir Medical College

**Objective:** Undetected invasive aspergillosis (IA) is a life-threatening complication in immunocompromised and haematological malignancy paediatric patients. Biopsy culture for diagnosis of IA can do more harm than good to an already weakened patient. Non-invasive biomarker assays like galactomannan enzyme assay can expedite the diagnosis and treatment of IA. In this study the authors had tried to evaluate the role of Galactomannan immunoassay in diagnosis of IA as per revised EORTC/MSG 2019 criteria.

**Material method:** Serum of 96 consecutive cases of paediatric (age ranging 6 m to 14 years) haematological malignancy patients were followed prospectively after enrolment to record demographic, clinical, radiological and treatment characteristics. Clinical and mycological workup (potassium-hydroxide mount, fungal culture) of the patients was done to classify proven, probable and possible IA as per EORTC-MSG guidelines,2019. The serum GMI (galactomannan indices) were measured in terms of sensitivity, specificity, positive predictive value (PPV) and negative predictive value (NPV) and was validated with revised EORTC -MSG, 2019 of IPA. **Results:** Three of these revealed fungal hyphae on histopathology after biopsy. Forty-six patients were categorised into probable invasive aspergillosis when routine mycological findings were taken into consideration. On culture *Aspergillus flavus* in 71% (n = 33), followed by *Aspergillus fumigatus* 24% (n = 11), rest were *Aspergillus niger* 5% (n = 2). The maximal Youden index “J” was 60%, (95% confidence interval of 0.4167 to 0.6875) corresponding to a GM index of >1.008, demonstrating that a GM index of >1.008 (95% Confidence interval a >0.87 to >1.4315) was the best cut-off point to predict IA with sensitivity and specificity were calculated as 85.42% and 75.00% respectively.

**Conclusions:** GM EIA when done in addition to culture from nonsterile sites and in defined population in paediatric patients is a excellent point of care test for IA as seen in this study on haematological oncology paediatric patients. This study further highlights that revisiting the definition of probable category of EORTC is very important for better utility of GM EIA assay in overall management in paediatric oncology haematological patients.

## P064 Comparison of Bordier Elisa Assay vs. Ldbio Ictigg/Igm Lateral Flow Assay for Diagnosing Chronic Pulmonary Aspergillosis: A Nigerian Experience

**Tina Nwosu ^1,2^, Rita Oladele ^2,3^ and Jean-Pierre Gangneux ^4^**

^1^ Central Research Laboratory, College of Medicine, University of Lagos
^2^ Medical Mycology Society of Nigeria
^3^ Department of Medical Microbiology & Parasitology, College of Medicine, University of Lagos
^4^ CHU Rennes, EHESP, Institut de Recherche en Santé, Insert, Environnement et Travail (Irset), UMR_S 1085, F35000, Universite Rennes

**Objectives:** Chronic pulmonary aspergillosis (CPA) is a known complication of pulmonary tuberculosis (TB). The detection of anti-*Aspergillus* antibodies is key to diagnosing CPA. Available techniques for the detection of *Aspergillus* IgG are limited and pose a great challenge in resource-limited settings in terms of affordability, skilled personnel, equipment and regular power supply. A point of care test will address most of these challenges. The objective of this study was to evaluate the performance of the LDBio ICT IgG/IgM lateral flow assay vs. that of the Bordier Elisa product in a field study in Nigeria.

**Methods:** Stored sera of 41 confirmed cases of CPA (from 3 sites in Nigeria) and 406 sera of healthy blood donors (from 9 sites representing the 6 geopolitical zones in Nigeria) were evaluated using both the Bordier Elisa (using cut-off value of 0.8 AU/mL, Bordier Affinity Products, Crisser, Switzerland) and the *Aspergillus* ICT IgG/IgM lateral flow assay (LFA) device (LDBio, Diagnostics, Lyon, France). Results were read both visually and digitally. R Statistical Computing Software version 4.0.2 was used for data analysis.

**Results:** Of the 41 serum samples tested in the CPA patient group, 24 tested positive by LDBio with 58.54% sensitivity (95% CI, 42.4% to 73.7%). In the non-CPA group, 403 of the 406 sera tested negative by LDBio with 99.3% specificity (95% CI, 97.9% to 99.9%). The predictive positive and negative values of the LDBio test were 88.9% and 95.9%, respectively. The gold standard (Bordier) test was positive for 41 of the 41 CPA serum samples tested with 100% sensitivity (95% CI, 91.4% to 100%). A comparison of the two tests showed that the LDBio test demonstrated lower sensitivity than the Bordier in detecting *Aspergillus* antibody (McNemar’s *p*-value <0.0001). However, both LDBio and Bordier show no significant difference in specificity (McNemar’s *p*-value = 0.0832) (Table 1). Results were in agreement for 427 of the 447 samples tested (95.5%) with a Cohen’s kappa coefficient of 0.68 (95% CI, 0.55 to 0.81), indicating good agreement between the LDBio and Bordier results. Youden’s J statistic calculated for the LDBio test results indicated a good balance between sensitivity and specificity, and the test had a high DOR of 190 (95% CI, 52 to 692).

**Conclusions:** The LDBio LFA test is a simple and rapid point of care test that can be implemented in field studies where Elisa is not available. A very good specificity and negative predictive value were observed in this study while the sensitivity was only of 58.54% compared to the Elisa when using a cut-off value of 0.8 AU/mL. The lower sensitivity of the LDBio LFA might be due to *A. fumigatus* being the a specie involved in maximum 50–60% of cases in Nigeria. Nevertheless, when combined with clinical features, the LFA can be used as a screening tool for CPA in settings such as ours, with an estimated cut-off of positivity of 1.0 AU/mL.

## P065 Evaluation of the PneumoGenius® PCR Assay for the Detection of *P. jirovecii* DNA and DHPS Mutations in Respiratory Samples

**Maël Roojee ^2^, Hélène Guegan ^1^, Solène Le Gal ^3^, J-P Gangneux ^1^ and Florence Robert-Gangneux ^1^**

^1^ Univ Rennes, CHU, Inserm, Irset (Institut de Recherche en Santé, Environnement et Travail)—UMR_S 1085, Rennes, France
^2^ Laboratoire de Parasitologie-Mycologie, Centre Hospitalier Universitaire de Rennes, Rennes, France
^3^ Laboratoire de Parasitologie et Mycologie, Hôpital de La Cavale Blanche, CHU de Brest, Groupe d’Etude des Interactions Hôte-Pathogène (ER, GEIHP), Université d’Angers, Université de Brest, Brest, France

**Objectives:** *Pneumocystis* pneumonia (PCP) is the most frequent fungal opportunistic infection in HIV-infected patients, and is of growing importance in HIV-negative patients. In this latter category of patients, the diagnosis mainly relies on PCR detection of fungal DNA on respiratory samples. This study aimed at evaluating the clinical performance of the PneumoGenius® real-time PCR assay (Pathonostics) on respiratory specimens relative to that of our in-house PCR assay. The PneumoGenius® assay is a commercial multiplex real-time PCR method, which detects the *Pneumocystis jirovecii* mitochondrial ribosomal large subunit (mtLSU) and two dihydropteroate synthase (DHPS) point mutations (codons 55 and 57), thus could be of interest to detect therapeutic failure.

**Methods:** In total, 206 respiratory samples from severely immunosuppressed patients sent to our lab for PCP diagnosis were retrospectively included. The patients were graded according to modified EORTC-MSG criteria as proven PCP (n = 82), probable PCP (n = 80), colonized (n = 36), and excluded (n = 8). All DNA were stored at −20 °C and re-tested with the Pneumogenius® assay, following the manufacturer’s instructions. To evaluate the accuracy of the DHPS mutant’s identification, an in-house PCR and Sanger sequencing were performed on selected samples (n = 34).

**Results:** The in-house PCR was positive for 198 samples, the Pneumogenius® assay was positive for only 182 of them (92% relative sensitivity). Four diagnostics of proven/probable PCP were missed by the Pneumogenius® assay (Sensitivity 97.5% for this sub-group); the PCR could be considered as “inhibited” in one case (absence of amplification of the internal control), and the mean Cq obtained for the 3 remaining samples was 37.5. For proven cases, the mean Cq obtained with the in-house PCR and the PneumoGenius® assay was significantly different (27.77 vs. 28.92, respectively, *p* < 0.05). Twelve negative results were obtained with patients diagnosed as colonized using the in-house PCR (mean Cq 36.25 ± 1.34 with the in-house PCR). No false positive results were observed with the 8 negative samples.

DHPS genotyping was successful for 147/182 positive samples with PneumoGenius®. Of the 34 samples which benefited from genotyping with both methods, at least one mutation was detected in 8, with full concordance between both techniques. Four patients (5 samples) were infected with two distinct *Pneumocystis* strains. Overall, mutations in codons 55 and 57 were observed in 4 and 6 strains, respectively. Two strains harbored a mutation on both codons. The 35 samples for which PneumoGenius® genotyping failed had a mean Cq of mtLSU amplification of 35.63 ± 1.63.

**Conclusions:** Pneumogenius® showed a good performance for genotyping, but was unable to diagnose PCP in one proven case and 2 probable cases with low Cq. As the mean Cq of amplification was higher than with the in-house PCR, this could explain the lack of detection of samples with low fungal loads. This lower sensitivity is overweight by a higher specificity, since colonization is less frequently detected.

## P066 Comparison of Two Commercial Colorimetric Broth Microdilution Tests for *Candida* Susceptibility Testing: Sensititre YeastOne vs. MICRONAUT-AM

**Sophie Philips ^1,2^, Frederik Van Hoecke ^1^, Emmanuel De Laere ^1^, Steven Vervaeke^1^, Roos De Smedt ^1^, Jerina Boelens ^3^, Deborah De Geyter ^2^, Denis Piérard ^2^ and Katrien Lagrou ^4^**

^1^ Department of Laboratory Medicine, AZ Delta Hospital, Deltalaan 1, 8800 Roeselare, Belgium
^2^ Department of Microbiology and Infection Control, Vrije Universiteit Brussel (VUB), Universitair Ziekenhuis Brussel (UZ Brussel), Laarbeeklaan 101, 1090 Brussels, Belgium
^3^ Department of Laboratory Medicine, Ghent University Hospital, Corneel Heymanslaan 10, 9000 Ghent, Belgium
^4^ Department of Laboratory Medicine and National Reference Centre for Mycosis, University Hospitals Leuven, Herestraat 49, 3000 Leuven, Belgium

**Objectives:** *Candida* is the most common cause of invasive fungal infections. Antifungal susceptibility tests (AFST) are crucial to guide therapy. Two colorimetric broth microdilution AFST were compared, Sensititre YeastOne and MICRONAUT-AM for nine antifungal agents.

**Materials & Methods:** One hundred clinical *Candida* isolates were tested, representing a realistic population for susceptibility testing in daily practice. CLSI clinical breakpoints (CBPs) and epidemiological cut-off values (ECVs) were used for Sensititre MICs while for MICRONAUT the EUCAST CBPs and ECVs were used.

**Results:** The reproducibility characteristics were comparable. Voriconazole and itraconazole showed a low EA of respectively 64.6% and 72.7%. Posaconazole had a very low EA of 38.3%. For fluconazole, however, an EA of 98.0% was demonstrated. The EA of echinocandins was 84.8% for micafungin, 89.9% for anidulafungin and 94.9% for caspofungin. For 5-flucytosine and amphotericin B, an EA of respectively 94.9% and 100% was noted. Sensititre minimal inhibitory concentrations (MICs) were systematically higher than MICRONAUT MICs for all antifungals, except for itraconazole. Only fluconazole, micafungin, and amphotericin B had a categorical agreement of ≥90%. Other molecules ranged from 66.7% to 81.9%. For fluconazole, micafungin, and amphotericin B the susceptibility proportions were comparable. Susceptibility proportion of posaconazole and voriconazole was higher using the MICRONAUT system. For itraconazole and anidulafungin, the susceptibility proportion was higher using Sensititre.

**Conclusions:** It was not possible to determine the true MIC values or the correctness of a S/I/R result since both commercial systems were validated against a different reference method. These findings show that there is a significant variability in susceptibility pattern and consequently on use of antifungals in daily practice, depending on the choice of commercial system.

## P067 Urine *Aspergillus* Antigen Detection as a Screen for Invasive Aspergillosis in Hematology-Oncology Patients

**Robina Aerts ^1^, Kausik Datta ^2,3^, Johan Maertens ^1^ and Kieren A. Marr ^2,3^**

^1^ Department of hematology, University Hospitals Leuven
^2^ MycoMed Technologies
^3^ School of Medicine, Johns Hopkins University

**Objectives:** Available diagnostics for invasive aspergillosis (IA) require sampling for blood or bronchoalveolar lavage (BAL) fluid, limiting application for screening to detect early disease. We previously reported that novel antibodies recognizing small molecular weight glycans can be used in a lateral flow format as an aid to diagnose IA using urine. Johns Hopkins and MycoMed Technologies have now optimized an ultrasensitive diagnostic enzyme immunoassay, called MycoMEIA™, with validation data demonstrating sensitivity 95.2% (95% CI 76.2–99.9) and specificity 94.2% (95% CI 89.3–97.3) as an aid to diagnose IA in people with suspected and confirmed IA. Early positivity suggested potential utility as a screening tool in people with high risks.

**Methods:** To test performance as a screening assay, we analyzed sequential urine samples obtained from people with proven or probable IA (cases), and no IA (controls), obtained from people treated for hematologic malignancies and/or receipt of allogeneic blood or marrow transplant (BMT). Patients had undergone aggressive screening with serum GM EIA and BALs were performed with suspicion of IA. Urine samples had been collected twice weekly and stored frozen at −80 °C. Results were interpreted using a low index cut-off to define positivity in order to optimize sensitivity and negative predictive value (NPV) for screening application.

**Results:** 120 urine samples were analyzed from 11 cases (proven or probable IA) as well as controls. Using the screening index cut-off of 0.8, 10/11 cases and no controls had at least one positive assay, yielding 91% per-case sensitivity and 100% specificity. Urine was positive in 6/7 cases with positive serum GM EIA and 6/7 cases with positive BAL GM EIA. Positive MycoMEIA assay pre-dated diagnosis established with the aggressive conventional screening approach in 6/10 cases, at mean 24.2 (range 2–62) days. Positive MycoMEIA post-dated conventional diagnosis by 8 (range 4–12) days in 4 cases.

**Conclusions:** Urine screening with MycoMEIA performed well to identify early IA in high-risk hematology patients. Larger studies are being performed to evaluate analytical index cut-offs for further optimization of sensitivity and predictive values.

## P068 Early Phenotypic Detection of Fluconazole-Resistant and Anidulafungin-Resistant *Candida glabrata* Isolates

**Panagiota Christina Georgiou ^1^, Maiken Cavling Arendrup ^3,4,5^, Spyros Pournaras ^1^ and Joseph Meletiadis ^1,2^**

^1^ Medical School, National and Kapodistrian University of Athens
^2^ Department of Medical Microbiology and Infectious Diseases, Erasmus Medical Centre
^3^ Unit of Mycology, Statens Serum Institute
^4^ Department of Clinical Microbiology, Rigshospitalet
^5^ Department of Clinical Medicine, University of Copenhagen

**Objectives:** *Candida glabrata* is the *Candida* species that most rapidly develops acquired resistance to fluconazole and echinocandins thus challenging clinical management. Early detection of resistance is important for choosing optimal therapy. We therefore developed a phenotypic test based on EUCAST E.Def 7.3 protocol for rapid detection of fluconazole and anidulafungin resistant isolates utilizing the colorimetric dye XTT.

**Materials & Methods:** Thirty-one clinical *C. glabrata* isolates were included, 11 anidulafungin-resistant (EUCAST MICs ≥ 0.125 mg/L), 20 anidulafungin-susceptible (MICs = <0.06 mg/L), 16 fluconazole-resistant (MICs ≥ 32 mg/L) and 15 fluconazole-“I” (susceptible, Increased exposure) (MICs≤16 mg/L). For the rapid XTT assay, 0.5–2.5 × 10^5^ CFU/mL of each isolate was added to RPMI1640 + 2% glucose medium containing 100 mg/L XTT + 0.78 μΜ MEN and 0.06 mg/L anidulafungin or 16 mg/L of fluconazole in 96-well flat bottom microtitration plates in a total volume of 200μL. A drug-free control was also included. All wells were incubated at 37 °C. The metabolic activity in each well was kinetically assessed by measuring absorbance at 450 nm for 12 h in a microplate reader (Tecan Infinite F200). Τhe differences in XTT OD absorbance between the drug-free and the drug-treated wells between fluconazole-resistant and fluconazole-I isolates and between anidulafungin-susceptible and anidulafungin-resistant isolates were assessed with student *t*-test at different time points and ROC curves were used in order to identify the best time point and the best cut-off, according to the % of specificity and sensitivity.

**Results:** The earlier time point with the highest area under the ROC (1.00, *p* < 0.001 for fluconazole and 0.99, *p* < 0.001 for anidulafungin) was 7.5 for fluconazole and 5.5 h for anidulafungin. At 7.5 h, the mean±SD XTT-absorbance in drug-free and drug-treated wells containing 16 mg/L fluconazole were 0.73 ± 0.14 and 0.67 ± 0.15, respectively for the fluconazole-resistant isolates and 0.72 ± 0.09 and 0.51 ± 0.08, respectively for fluconazole-I isolates. The XTT-absorbance differences of the 16 mg/L-containing well from the drug-free well were 0.08 ± 0.05 for the fluconazole-resistant isolates significantly lower than the 0.25 ± 0.06 for the fluconazole-I isolates (*p* < 0.0001). ROC curve analysis identified an XTT-absorbance difference between the 16 mg/L-containing well and the drug-free well of 0.157 at 7.5 h with 100% sensitivity and specificity in detecting fluconazole resistant strains. For anidulafungin after 5.5 h, the mean ± SD XTT-absorbance in drug-free and drug-treated wells were 0.39 ± 0.05 and 0.25 ± 0.06 for the anidulafungin-resistant and 0.37 ± 0.04 and 0.11 ± 0.03 for anidulafungin-susceptible isolates. The difference between the XTT-absorbance of drug-free and drug-treated wells were 0.102 ± 0.07 for the resistant and 0.266 ± 0.04 for the susceptible isolates, a significant difference with *p* = 0.0001. ROC curve analysis identified an XTT-absorbance difference between the 0.06 mg/L-containing well and the drug-free well of 0.186 at 5.5 h with sensitivity 100% and specificity was 88.89% in detecting anidulafungin-resistant strains.

**Conclusions:** A simple, cheap and fast phenotypic test was developed using the XTT dye with high specificity and sensitivity in detecting fluconazole-resistant and anidulafungin-resistant *C. glabrata* isolates within 7.5 h and 5.5 h, respectively. This method can generate phenotypic resistance results within the time frame of molecular tests.

## P069 Diagnostic Value of Galactomannan Lateral Flow Device in Respiratory and Serum Samples for Patients with COVID-19 Associated Pulmonary Aspergillosis (CAPA)

**William Hurt ^1^, Tihana Bicinic ^1^, Lewis White ^3^, Jonathan Youngs ^1^, Duncan Wyncoll ^2^, Yusuff Hakeem ^4^, Phillip Hopkins ^5^, Susannah Leaver ^6^, Matt Wise ^7^, Silke Schelenz ^5^ and Anna Goodman ^2^**

^1^ St. Georges University
^2^ Guy’s and St. Thomas’ NHS Trust
^3^ Public Health Wales
^4^ University Hospitals of Leicester NHS Trust
^5^ King’s College Hospital NHS Trust
^6^ St. Georges University Hospitals NHS Trust
^7^ University Hospital of Wales NHS Trust

**Objectives:** To evaluate the performance characteristics of the IMMY aspergillus lateral flow device in serum and respiratory samples for the diagnosis of pulmonary aspergillosis in intubated and ventilated patients with COVID-19.

**Materials & Methods:** ASpiFlu (ISRCTN51287266; IRAS 271269) is a prospective, multicentre, observational cohort study at 5 UK hospitals including 2 extra corporal membrane oxygenation (ECMO) centres, aiming to estimate the incidence of CAPA in ventilated patients with COVID-19, and to validate novel point-of-care tests for diagnosing CAPA in the ICU.

A diagnosis of pulmonary aspergillosis relies on one of the following: a positive *Aspergillus* culture from a deep respiratory sample, or a positive PCR/galactomannan antigen from a deep respiratory or serum sample. The sensitivity of culture is poor [1], and PCR testing and galactomannan EIA is usually only performed at specialist centres as send away tests, resulting in long turn around times with the potential to delay diagnosis. Lateral flow galactomannan testing has been show to be comparable to galactomannan EIA testing for the diagnosis of pulmonary aspergillosis in BAL fluid from neutropenic and non-neutropenic patients [2] and is implementable as a point of care test. We therefore plan to analyse its performance in the setting of CAPA on the ICU, where its use has the potential to improve time to diagnosis.

From March 2020–March 2021, 258 COVID19 patients were prospectively enrolled. Study sampling included 2 sera taken one week apart as well as any leftover respiratory samples (tracheal aspirates, non-directed bronchoalveolar lavage and bronchoalveolar lavage). Fungal cultures were performed at study sites as per standard of care. Galactomannan EIA, *Aspergillus* PCR and IMMY lateral flow testing will be performed at the Welsh mycology reference centre on all sera and respiratory samples in June–July 2021. CAPA was defined as proven, probable and possible according to 2020 ECMM/ISHAM Consensus Criteria [3].

**Results:** Patient enrolment is complete and retrospective sample testing is currently ongoing but will be complete by August 2021. Data on incidence will be reported at ECCMID 2021, we expect this to be between 5–10%. The sensitivity, specificity, negative and positive predictive value of the IMMY galactomannan lateral flow testing will be calculated against proven/probable and possible CAPA. Modelling will also be performed to evaluate the potential cost-effectiveness of testing when compared to conventional testing using galactomannan EIA and culture. This will include potential time saved to diagnosis as well as the impact on antifungal use, extrapolated from the clinical information collected for patients enrolled into the Aspiflu study.

**Conclusions:** Aspiflu is the largest prospective study of CAPA incidence and includes patients from multiple UK ITUs, including 2 ECMO centres. The number of samples (~180 respiratory and ~500 sera) ensures that we are well placed to report on test performance. Aspergillus PCR, and galactomannan antigen EIA will performed in parallel on every sample enabling comparison against other recommended testing modalities.

**References**

1. Meersseman, W.; Lagrou, K.; Maertens, J. Galactomannan in bronchoalveolar lavage fluid: A tool for diagnosing aspergillosis in intensive care unit patients. *Am. J. Respir. Crit. Care Med.* **2008**, *177*, 27–34.
2. Jenks, J.D.; Mehta, S.R.; Taplitz, R. Point-of care Diagnosis of Invasive Aspergillosis in in Non-Neutropenic Patients: *Aspergillus* Galactomannan Lateral Flow Assay vs. *Aspergillus*-specific Lateral Flow device test in Bronchoalveolar Lavage. *Mycoses* **2019**, *62*, 230–236.
3. Koehler, P.; Bassetti, M.; Chakrabarti, A. Defining and managing COVID-19 associted pulmonary aspergillosis: 2020 ECMM/ISHAM consensus criteria for reseach and clinical guidance. *Lancet* **2020**, *21*, e149–e162.

## P070 Phenotypic, Genotypic and Proteomic Identification of Trichosporon Species: A Globally Emerging Yeast of Medical Importance

**Luciana da Silva Ruiz ^1^, Bruna Rossini Lara ^1,2^, Bruno Braidotti de Camargo ^2^, Claudete Rodrigues Paula ^3^, Regina Teixeira Barbieri Ramos ^3^, Diniz Pereira Leite Junior ^4^, Hans Garcia Garces ^2^, Mariana Volpe Arnoni ^5,6^, Mônica Silveira ^7^, Viviane Mazo Fávero Gimenes ^8^, Lumena Pereira Machado Siqueira ^8^, Juliana Possatto Fernandes Takahashi ^9^, Márcia de Souza Carvalho Melhem ^9^, Mário Mendes Bonci ^3^, Virgínia Bodelão Richini-Pereira ^1^ and Laís Anversa ^1^**

^1^ Adolfo Lutz Institute (IAL)
^2^ São Paulo State University (Unesp), Institute of Biosciences
^3^ University of São Paulo (USP), School of Dentistry
^4^ Faculty of Medicine, Federal University of Mato Grosso (UFMT)
^5^ Irmandade da Santa Casa de Misericórdia de São Paulo
^6^ Darcy Vargas Children’s Hospital
^7^ Bauru State Hospital (HEB)
^8^ University of São Paulo (USP), Institute of Tropical Medicine
^9^ Adolfo Lutz Central Institute (IAL)

**Objectives:** The present study aimed to compared the phenotypic, genotypic and mass-spectrometry (MALDI-TOF) identification of 59 yeasts of the genus *Trichosporon*. MALDI-TOF, in particular, will be compared to a molecular technique (gold standard).

**Materials & Methods:** This research included 59 yeast samples of the genus *Trichosporon*, 44 of which were of human origin, four of animal origin and 11 of environmental origin. The samples, previously identified as belonging to the genus *Trichosporon*, are part of the Collections at the following Brazilian laboratories: Laboratory of Mycology and Parasitology, Adolfo Lutz Institute (IAL) CLR II, Bauru-SP; Laboratory of Pathogenic Yeasts, School of Dentistry, University of São Paulo, São Paulo-SP; Laboratory of Mycology, Department of Microbiology and Immunology, São Paulo State University, Botucatu-SP; Laboratory of Research, Federal University of Mato Grosso, Cuiaba-MT; and Institute of Tropical Medicine, University of São Paulo, São Paulo-SP. For phenotypic identification, all samples were initially cultured on chromogenic medium, by the method of exhaustion. The yeasts also had their macroscopic, microscopic, reproductive and physiological characteristics studied according to the methods recommended. Tests included slide microculture on corn meal agar and Tween 80, urease test, tolerance to 0.1% cycloheximide, growth at 37 °C, auxanogram (carbohydrate and nitrogen assimilation) and zymogram (carbohydrate fermentation). Species of the genus *Trichosporon* were identified with the keys described in the literature. The MALDI-TOF were employed in the study for proteomic identification and the results were expressed as scores; values ≥2000 were adopted for species-level identification, values between 1700 and 1999 were adopted for genus-level identification, and values <1700 were not considered reliable for identification. The strains were molecularly identified by rDNA IGS1 region analysis. IGS1 region amplification was obtained with primers 26SF and 5SR. The amplified products were purified and sequenced. Then, amplicons were visualized in Chromas 2.3 Technelysium software, aligned by MEGA 7 program, applied to BLASTn and compared with the sequences available at GenBank. Maximal identity over 98% was accepted as molecular identification of the species.

**Results:** Concordance level between the traditional phenotypic method and the molecular technique (gold standard) in the identification of all 59 *Trichosporon* samples was 59.3%. Identification concordance between MALDI-TOF spectrometry and the molecular technique was 71.2%. No isolate of environmental origin was identifiable by MALDI-TOF mass spectrometry, and 100% of such environmental isolates were discordant for IGS region sequencing and phenotypic characterization. Both comparisons evidenced greatest concordance in the identification of *T. asahii*. The species *T. debeurmannianum, T. dermatis, T. venhuisii* and *T. insectorum* were not properly identified by both MALDI-TOF and the phenotypic technique.

**Conclusions:** MALDI-TOF, in particular, seems to be appropriate to investigate yeasts of the genus *Trichosporon*; however, database updates are still necessary, especially for species that are not common in the clinical routine.

## P071 Use of Serial Bronchoalveolar Lavage Fluid Aspergillus Galactomannan in Patients with Invasive Pulmonary Aspergillosis

**Daniel ZP Friedman ^1^, Elitza Theel ^2^, Randall Walker ^1^, Raymund Razonable ^1^ and Paschalis Vergidis ^1^**

^1^ Mayo Clinic, Department of Medicine, Division of Infectious Diseases
^2^ Mayo Clinic, Department of Laboratory Medicine and Pathology, Division of Clinical Microbiology

**Objectives:** Detection of *Aspergillus* galactomannan (GM) from serum and bronchoalveolar lavage (BAL) fluid can support the diagnosis of invasive pulmonary aspergillosis (IPA) in immunocompromised hosts. If positive, serum GM can be useful to monitor disease progression or improvement. No studies have investigated if changes in BAL GM can predict treatment response in a similar way to serum GM. Our aim was to describe the changes in BAL GM over time and determine if a serial change in BAL GM value correlated with clinical disease course while on antifungal therapy.

**Methods:** We performed a retrospective review of patients with a positive BAL GM collected January 2011 to February 2021. We identified patients from laboratory records and included those with a positive BAL GM and a follow-up GM collected within 7–100 days. Medical records were reviewed to confirm IPA diagnosis and to collect demographics, clinical characteristics, radiographic findings, therapy and outcomes.

We determined the patients’ response to therapy by comparing clinical symptoms and CT imaging findings from baseline to follow-up assessments. We used MSG/EORTC definitions to characterize the clinical response as a success or failure. GM change was considered concordant with clinical response: (i) if a decline of ≥0.500 in GM was observed in a patient with successful clinical outcome, or (ii) if an increase in GM was observed in a patient with clinical failure. Chi-square test, Student’s *t*-test or Mann-Whitney test were used to compare characteristics between patients with a decreasing vs. increasing GM and those with clinical success vs. failure.

**Results:** We found 218 patients who had serial BAL GM testing within the study period. Of those, 52 met criteria for IPA and had follow-up testing within 7–100 days. The mean age was 60.7 years (range = 11.4–80.5) and 67.3% (n = 35) were male. 98.1% (n = 51) of patients received immunosuppressive treatment in the preceding 60 days, 56.7% (n = 29) had a hematologic malignancy, 51.9% (n = 27) had severe neutropenia and 33.3% (n = 17) received a solid organ transplant. 46 patients (88%) had proven or probable IPA. The median baseline GM value was 3.483 (range = 0.606–3.750).

The median time to repeat GM measurement was 32 days (range = 7–89 days) and the meadian time to clinical reassessment was 49 days (range = 8–428 days).

Thirty patients (58%) had a decrease in BAL GM value by ≥0.500 and 13 patients (25%) had clinical success. Clinical success occured in 30.0% of those with decreasing GM vs. 18.2% in those increasing GM (*p* = 0.331). The median magnitude of GM decrease was 1.168 vs. 0.755 (*p* = 0.491) between those with clinical success and failure. Seventeen of 22 patients (77.3%) with an increase in GM had clinical failure.

**Conclusions:** In our cohort, a decrease in BAL GM was not predcitive of a successful outcome. However, stability or an increase in GM was more likely to occur in those with clinical deterioration. Discordant BAL GM response may reflect variability in bronchoscopist technique, sampling location or dilution effect. A standardized, prospective study would be most helpful to assess the use of serial BAL GM to predict clinical outcomes.

## P072 Antibodies against *Candida albicans* Germ Tubes Recognize Potential Biomarkers of Invasive Candidiasis

**Marta Bregón ^1,2^, Giulia Carrano ^1^, Ander Díez ^1,2^, Pilar Menéndez-Manjón ^1,2^, Iñigo Fernández de Larrinoa ^3^, Inés Arrieta ^1^ and Maria Dolores Moragues ^1^**

^1^ Department of Nursing, University of the Basque Country UPV/EHU
^2^ Department of Immunology, Microbiology and Parasitology, University of the Basque Country UPV/EHU
^3^ Department of Organic Chemistry, University of the Basque Country UPV/EHU

**Objectives:** Identification of proteins recognized by antibodies that react with superficial antigens of *Candida albicans* germ tubes (CAGTA), expressed in a cDNA phage library of *Candida albicans* growing as mycelia.

**Materials & Methods:** The mycelial *C. albicans* SC5314 cDNA library was harboured in Lambda Zap II phage, and was kindly provided by Dr P. Sundstrom and Dr W. Fonzi. The library screening was carried out with a CAGTA-enriched serum fraction of a White New Zealand rabbit infected with *C. albicans* SC5314. After two re-screening rounds, positive-clone inserts were amplified and sequenced.

Target proteins were identified with the BLAST tool and aligned with the BioEdit program. Information about the proteins was obtained through the databases UniProt and *Candida* Genome Database. Antigenic determinants on proteins were predicted with the Kolaskar-Tongaonkar algorithm (http://imed.med.ucm.es/Tools/antigenic.pl).

**Results:** The analysis of 1.5·10^6^ lysis plaques forming units returned 82 positive clones, of which 29 were not in the correct reading frame. More than half of the 53 phages with in-frame coded proteins matched the hyphally-regulated cell wall protein (Hyr1), while others corresponded to enolase 1 (Eno1), cystathionine γ-lyase (Cys3), aminopeptidase 2 (Ape2) and the coatomer subunit gamma (Sec21) of *C. albicans*.

According to the UniProt and *Candida* Genome Database information, except for the coatomer subunit gamma and enolase, the other proteins are mainly located in the cell wall of the fungus and/or have been related with virulence, biofilm formation or the yeast-to-hyphae transition of *C. albicans*. Even though enolase is a cytoplasmic enzyme, it has also been found in the cell wall, and several studies support its utility as a biomarker for Invasive Candidiasis. All the proteins identified by CAGTA in this study showed antigenic regions that could be suitable candidates for immunodiagnostic purposes.

**Conclusions:** The CAGTA enriched serum fraction of an animal model of Invasive Candidiasis recognizes proteins expressed in a cDNA library of the fungus *Candida albicans* growing as mycelia.

Most in-frame codified proteins recognized by the CAGTA-enriched serum fraction are related with the *C. albicans* yeast-to-hyphae or the biofilm formation processes and could be suitable candidates as biomarkers for the diagnosis of Invasive Candidiasis.

**Financial support:** M. Bregón was recipient of a grant from the UPV/EHU (PIF19/316) and P. Menéndez-Manjón was recipient of a grant from the Basque Government. GEIFI research team was supported by the project IT913-16 from the Basque Government.

## P073 Detection and Identification of Aspergillosis and Mucormycosis Etiologic Agents in Biological Samples of Patients Using Multiplex Real Time PCR

**Svetlana Ignatyeva ^1^, Tatiana Bogomolova ^1^, Yriy Avdeenko ^1^, Olda Shadrivova ^1^, Sofia Khostelidi ^1^, Ylia Borzova ^1^, Ekaterina Desyatik ^1^, Olga Kozlova ^1^, Alisa Volkova ^2^, Marina Popova ^2^, Ylia Chudinovskikh ^3^, Iliya Zyuzgin ^3^, Olga Uspenskaya ^4^, Nikolay Klimko ^1^ and Natalya Vasilyeva ^1^**

^1^ I. Metchnikov North-western State Medical University, Kashkin Research Institute Of Medical Mycology
^2^ I. Pavlov First Saint Petersburg State Medical University
^3^ Petrov Scientific and Research Oncology Institute of the Ministry of Health of Russia
^4^ Leningrad Regional Clinical Hospital

**Objectives:** The aim of this study was to evaluate a multiplex real time PCR with High Resolution Melt analysis (mHRM-RT-PCR) on clinical samples for simultaneous detection and identification of *Aspergillus* spp. and mucormycetes in biological samples of patients with mycosis.

**Methods:** We tested 47 BAL, 29 native tissue and formalin-fixed paraffin-embedded tissue samples from 59 patients with aspergillosis and 17 patients with mucormycosis in Saint-Petersburg between 2013 and 2020 yy. As controls, 34 tissue and 30 BAL samples were tested from patients without mycoses. Fungal DNA was extracted from clinical samples by a chloroform-isoamyl extraction method. DNA amplification was performed using *Aspergillus*- and mucormycetes-specific primers pairs separately and EvaGreen based mHRM-RT-PCR on Rotor-Gene 6000 cycler.

**Results:** In clinical samples from patients with aspergillosis and mucormycosis the mHRM-RT-PCR allowed to identify representatives of *Aspergillus* spp. to the genus and mucormycetes—to the species level: *Rhizopus arrhizus, Mucor racemosus, Rhizomucor pusillus* and *Lichtheimia corymbifera*. Direct microscopic examination of 38 BAL from patients with aspergillosis and 9 BAL from patients with mucormycosis was positive in 49% and 56% cases, respectively. *Aspergillus* spp. were isolated in 84% and mucormycetes in 89% cases. Sensitivity of PCR assay in BAL was 89%. In one patient mHRM-RT-PCR detected a mixed infection by *Aspergillus* and *Rhizopus arrhizus*. In patients with aspergillosis direct microscopy of 15 native tissue samples was positive in 80% cases. Only in 2 of 4 native tissue samples of patients with mucormycosis and positive direct microscopy culture was positive (*L. corymbifera* and *R. arrhizus* was isolated). mHRM-RT-PCR detected in 3 of 4 samples *L. corymbifera* and in 1 sample -*R. arrhizus*. PCR assay was positive in formalin-fixed paraffin-embedded tissue samples of 95% patients with aspergillosis and 100% patients with mucormycosis. mHRM-RT-PCR allowed to identify the representatives of mucormycetes: *Lichtheimia corymbifera* in 6 and *Rhizomucor pusillus* in 3, Rhizopus microsporus in 1 from 12 samples. In biological specimens of 2 patients the PCR assay detected a mixed infection by *Aspergillus* spp. + *Rhizopus microsporus* and *Aspergillus* spp. + *Rhizopus arrhizus*.The results of PCR assay in patients with mycosis and control patients correlated with results of microscopy and culture from BAL in 63 from 67cases (94%) and results of histological investigations from tissue samples—in 96% cases.

**Conclusions:** In patients with aspergillosis and mucormycosis the sensitivity of PCR assay depended according the type of biological specimens. The mHRM-RT-PCR may be a useful tool for detection of etiologic agents of mycoses, particularly in the case of a mixed infection by *Aspergillus* and mucormycetes.

## P075 Molecular Confirmation of the Phenotypic Identification of Clinical Strains of non-*albicans* Yeasts

**Theopisti Sarmourli, Argyri Togkousidou, Aikaterina Poulopoulou, Panagiotis Siasios and Timoleon-Achilleas Vyzantiadis**

Department of Microbiology, Medical School, Aristotle University of Thessaloniki

**Objectives:** Fungal infections are increasingly recognized as a serious concern for human health, especially in immunocompromised patients and those hospitalised with underlying conditions. Over the last decades an increasing number of cases of infections with non-*albicans* yeasts, have been identified by the use of morphologiacla/phenotypic and/or molecular methods. More recently, *Candida auris*, a multidrug resistant yeast of great public health concern, has been recognised as a cause of invasive infections and often misdiagnosed as many diagnostic platforms were not able (at least previously) to identify it. The purpose of this retrospective study was the sequence-based species delineation of several yeasts strains, from the fungal collection of our laboratory, in an effort to confirm the previous non-molecular identifications and also to check for possible misidentifications of *C. auris* during the last decade.

**Materials and methods:** A total of forty-eight isolates from deep or even superficial specimens referred for mycological diagnosis to our laboratory during the last decade were selected for analysis. All yeasts found in the collection that could be misidentified (according to the literature) instead of *Candida auris* were included. These isolates were previously identified by the use of microscopy, culture on several mycological media (including chromogenic media) and at different temperatures (30 °C and 35 °C), germ tube testing and biochemical testing by API ID 32C, as *Candida parapsilosis, C. lusitaniae, C. kefyr, C. guillermondii, C. famata* or *S. cerevisiae*. There was not any *Candida haemulonii*. From the large population of *C. parapsilosis*, there were selected only those that in API ID 32C, although they had a very good percentage of identification (%id), they presented a T index lower than 0.90, while its optimal value is 1.0. All isolates were recultivated on malt extract agar and the fungal DNA was extracted by heating at 95 °C under buffered conditions, followed by strong agitation and centrifugation. The extracts were molecularly analysed by the amplification and sequencing of the internal transcribed spacer 1 or 2 (ITS1 or ITS2) region of the fungal ribosomal DNA (rDNA) and compared according to the BLAST® tool and ISHAM ITS database. The final identifications were deposited in GenBank database.

**Results:** All isolates’ phenotypical identifications were confirmed by the present molecular procedure and no misidentifications were revealed. The only differences concerned the changes in several species’ names that occurred during the last years, connected mostly to the fungal teleomorph. Additionally, it was proved that among all these probably doubtful identifications, there was not any *Candida auris*, although most of the species tested have been previously described in the literature as possible sporadic misidentifications of *C. auris*.

**Conclusions:** The identification of yeasts based on phenotypical characteristics has a good accuracy, and is cost effective and clinically relevant. Sequencing, on the other hand, is a confirmative method, but requires more hands-on time and is not available in most clinical laboratories. *Candida auris* was not identified among the strains analysed. This, combined to the small number of reports, might indicate that the existence of the fungus in our geographical area is still low.

## P076 Performance of *Aspergillus galaktomannan* Lateral Flow Assay for Diagnosis of Invasive Aspergillosis in Adult Cancer Patients

**Ozlem Alhan Guncu ^1^, Zeynep Ture Yuce ^2^, Mehmet Mucahit Guncu ^3^, Huseyin Nadir Kahveci ^2^, Asu Fergun Yilmaz ^4^, Zekaver Odabasi ^1^**

^1^ Department of Infectious Diseases and Clinical Microbiology, Faculty of Medicine, Marmara University
^2^ Department of Infectious Diseases and Clinical Microbiology, Faculty of Medicine, Erciyes University
^3^ Department of Medical Microbiology, Institute of Health Sciences, Marmara University
^4^ Department of Hematology, Faculty of Medicine, Marmara University

**Objectives:** Invasive aspergillosis (IA) is associated with high mortality rates in cancer patients. The gold standard method for diagnosis of IA is a positive culture for *Aspergillus* in tissue biopsy, but biopsy is often not performed in patients with hematological malignancies. Galactomannan (GM) detection with enzyme-linked immunoassay (ELISA) is the most used non-culture method in screening and diagnosis for IA. *Aspergillus* galactomannan lateral flow assay (LFA) which is a cheaper and faster method than ELISA is promising for improving the diagnosis of IA. The performance of LFA in serum and bronchoalveolar lavage fluid (BALF) of cancer patients was evaluated in this multicentre study.

**Materials & Methods:** In this prospective multicentre study, we included 58 patients with hematological or oncological malignancies. All of the patients had nodule, cavity, air crescent sign, or lobar consolidation on their chest tomography. Only serum samples of 32 patients and serum and BALF samples of 26 patients were taken between 2019 and 2021 in two university hospitals in Turkey. GM ELISA (Platelia *Aspergillus* EIA, Bio-Rad^®^) and LFA (IMMY^®^) were studied from serum and BALF samples. All patients are grouped as ‘proven’, ‘probable’ and ‘possible’ IA according to the EORTC/MSG guideline published December 2019.

**Results:** The majority of patients had hematological malignancy (n = 48, 82%) and 13 patients of these group had bone marrow transplantation. According to EORTC/MSC criteria, cases were classified as proven (n = 2), probable (n = 20) and possible (n = 36). Twenty-one (36.2%) patients were receiving antifungal prophylaxis but only 4 patients had anti-mold prophylaxis (all were classified as possible IA). The study demonstrated a sensitivity of 81,8% and specificity of 91.6% at a 1.0 optimal density index (ODI) cut-off for LFA. When the cut-off point was 1.5 ODİ, the positive predictive value and specificity were found to be 100%, but the specificity of LFA dropped to 68% (Table 1). While 92.8% of patients with serum ELISA positive had LFA positive (13/14), 95.4% of patients with negative serum ELISA had LFA negative (42/44) at a 1.0 ODI cut-off for ELISA and LFA (Table 2).

**Conclusions:** *Aspergillus* galactomannan LFA seems as good as GM ELİSA for the diagnosis of IA in cancer patients. However, studies with larger samples are needed to determine the ideal cut-off point of LFA.

## P077 Evaluation of the New Micronaut-AM System to Determine the MICs of *Candida* spp.

**Christine Bonnal ^1^, Nikolett Szabo-Gyurtane ^1^, Sandrine Houzé ^1^ and Françoise Dromer ^2^**

^1^ Hôpital Bichat Claude Bernard
^2^ Institut Pasteur

**Objectives:** The determination of antifungal MIC is often challenging because the reference methods (CLSI or EUCAST) rely on measuring growth of a defined fungal inoculum in a specific growth broth in the presence of different concentrations of the antifungal drug. These methodologies are time-consuming. Therefore, Etest method is often prefered in routine but also has some limitations, mainly due to the difficulty to read the results and the absence of breakpoints for certain antifungal drugs.

Micronaut-AM system (Merlin Diagnostika, GmbH, Berlin, Germany) is a new method used to determine the antifungal MIC (fluconazole, voriconazole, posaconazole, caspofungin, anidulafungin, micafungin, amphotericin B and 5-flucytosine) based on EUCAST technic associated with a colorimetric reading limiting the trailing growth effect and facilitating the reading. It is only validated for *Candida* spp. and *Cryptoccus* spp. The aim of our study was to compare the results obtained with Micronaut-AM and the EUCAST technique for *Candida* spp.

**Materials & Methods:** Strains of *Candida* spp. for which the antifungal susceptibility have already been measured with the EUCAST technic performed by the National Reference Center of Invasive Mycosis (Institut Pasteur, France) were included (anidulafungin was not available). Antifungal MICs of these strains at a concentration of 0.5 McFarland were measured by using Micronaut-RPMI-1640 growth medium completed with the AST-Indicator. Each antifungal is available in up to 11 concentrations. Growth of the fungi is indicated by a color change from blue to pink. The MIC was considered the lowest concentration leading to colorimetric changes after 24 h to 48 h of incubation at 35 °C (read visually). Results were interpreted using the EUCAST 2018 susceptibility breakpoints.

The results obtained by the two technics (Micronaut-AM and EUCAST) were compared by using the number of dilutions between the MICs measured for each antifungal and each strain. Results were considered as concordant if the difference of MIC values was −2, −1, 0, 1 or 2. When the MICs were discordant, the interpretation of the sensitivity of the strain to the antifungal (Susceptible or Resistant) was also evaluated.

**Results:** Twenty-one strains were studied: 9 *C. albicans*, 3 *C. parapsilosis*, 3 *C. glabrata*, 2 *C. lusitaniae* and 1 of each, *C. krusei, C. tropicalis, C. kefyr*, and *C. metapsilosis*. The MICs obtained by the 2 technics were concordant for 19/21 strains for voriconazole, amphtericin B, fluconazole, micafungin, 18/21 strains for 5-fluorocytosin, 17/21 for posaconazole, but only 14/21 for caspofungine. Anyway, it never led to a modified interpretation of the sensitivity of the strains. The Micronaut-AM was easy to perform and to read whatever the *Candida* species.

**Conclusions:** This evaluation confirms the interest of Micronaut-AM for determining antifungal sensitivity of *Candida* spp. It is easier to perform than current methodes (Etest and EUCAST) because the reagents are ready to use and offers a greater number of breakpoints to interprete the results. Moreover a photometric reading (Micronaut Skan) is also available with the Micronaut Software facilitating the interpretation. Complementary evaluations are needed for *Cryptococcus* spp. and rare *Candida* spp.

## P078 Mycetoma in an Unusual Location, Nigeria

**Iriagbonse Osaigbovo ^1,2^, Ikponmwosa Obahiagbon ^1,2^ and Dele Imasogie ^1,2^**

^1^ University Of Benin
^2^ Teaching Hospital, University of Benin

**Objectives:** Mycetoma is a neglected tropical disease affecting skin, subcutaneous tissue and sometimes muscle, bone and contiguous organs. It may be of bacterial or fungal origin and typically affects the lower extremities. The objective of this report is to describe an unusual case of mycetoma in Southern Nigeria and the difficulties experienced in diagnosis.

**Materials & Methods:** An eleven year old school girl presented with a swelling on the nape of her neck of a year’s duration. The swelling consisted of multiple nodules, some with sinuses which on occasion drained purulent material. Her mother gave a history of onset about 2 weeks after she had a haircut in a barbing salon. There was no obvious trauma. The lesion began as a single ‘boil’ which gradually became multiple and recurrent. It was associated with itching. The swelling did not respond to antibiotics procured over the counter. She presented at the dermatology clinic and a diagnosis of carbuncle was made for which she was placed on Clindamycin for a week with no respite. She was then referred to clinical microbiology for review as a possible case of deep mycosis. Aspiration of one of the fluctuant nodules was done for microscopy and fungal culture and a skin biopsy was advised for both microbiology and histology.

**Results:** Aspiration yielded scanty seropurulent material. There were no grains seen or felt. Both Gram stained and Giemsa stained smears showed numerous polymorphonuclear cells, mostly neutrophils but with significant number of eosinophils and a few macrophages. No organisms were seen. Culture yielded no growth. Due to inavailability of funds, the patient did not do a biopsy but began a course of itraconazole 200 mg b.d prescribed by the dermatologist. On follow-up two weeks later, there was a marked improvement with regression of the lesion although some nodules remained. A biopsy was done at this time and sample sent for histology but not microbiology. Haematoxylin and eosin stained sections showed fibrosis in the dermis and granulomas made up of predominantly neutrophils. No fungi were seen on PAS. However, Ziehl Nielsen stained section showed an irregularly shaped grain with central clearing. A diagnosis of mycetoma was made possibly due to a *Nocardia* species.

**Conclusions:** This case of mycetoma is unusual because of the location at the nape of the neck. The aetiology is also unclear because microbiological analysis did not yield the causative agent. There was clinical response to itraconazole suggesting a fungal aetiology. However histopathological examination of a bipopsy taken from a healing lesion suggested an actinomycetoma, specifically one caused by *Nocardia*. Although results are not guaranteed from microbiological analysis, failure to send specimens for microbiology may hinder diagnosis. Improper tissue processing and inexperience may also hamper diagnosis especially as mycetomas are seen more in the central and northern states of Nigeria. Multidisciplinary approaches and application of newer techniques such as molecular methods may enhance the diagnosis of mycetoma in our setting.

## P079 Development of Dermatophytes Specific Medium

**Jihane Kabtani, Dounia Bergoug and Stéphane Ranque**

IHU—Mediterranee Infection

**Introduction:** Dermatophytes are microscopic filamentous fungi, mainly characterized by their affinity for keratin. The species of the *Trichophyton rubrum* complex constitute the most common agents of dermatophytosis, which gave *Trichophyton rubrum* the position of the most worldwide spread dermatophyte. Dermatophyte’s culture is very important in the process of the mycological diagnosis, and the use of the reference medium Sabouraud, supplemented with antibacterials (chloramphenicol ± gentamicin) and cycloheximide (Actidione®), does not always prevent contaminations by saprophytic fungi. A lot of studies have attempted to solve the problem, without success.

**Objectives:** The aim of our study is to develop a specific culture medium for these keratinophils, with an inhibitory effect on the growth of *Aspergillus fumigatus*, which represents the major contaminant.

**Methods and Materials:** To achieving this, we used two different approaches. We first screened the activity of 9 vital dyes (Janus green B, Bromocresol purple, Amaranth, Brilliant cresyl blue, Nigrosin, Ponceau BS, Red congo, Orange II and Rhodamine B) on type strains of *Trichophyton rubrum* and *Aspergillus fumigatus*.

Then, in order to phenotypically characterize the two fungi, we performed phenotypic tests with the Biolog’s advanced phenotypic technology using different type of MicroPlates (Filamentous Fungi MicroPlates (Gen III) for carbon sources assimilation, PM 1 to 10 for pH/Osmolytes/Nitrogen/Phosphorus/Sulfur sources and PM 21 to 25 for fungi chemical sensitivity tests). With the purpose to find one or several substrates assimilated by the *Aspergillus fumigatus* and not by *Trichophyton rubrum*, and using it to have an optimized medium.

**Results:** Three dyes showed growth inhibition of *Aspergillus fumigatus* and maintenance of dermatophyte’s one. The association between these three dyes was not as effective as their individual use. Concerning the second approach, all the substrates assimilated by *Trichophyton rubrum*, were also by *Aspergillus fumigatus*.

**Conclusions:** The dyes approach was more relevant. For proving its efficiency, the optimized media with dyes were tested on other clinically relevant dermatophytes contaminating fungi, such as; Aspergillus niger, Aspergillus oryzae and Aspergillus tubingensis.

**Keywords:** *Trichophyton rubrum*; dermatophytes; *Aspergillus fumigatus*; contamination; culture; dyes; biology

## P080 Clinical and Epidemiological Features of Patients with Co-Infection (*Aspergillosis*\*Tuberculosis*)

**Irina Burmistrova, Alexandra Gracheva, Anna Panova, Tatiana Tiulkova and Irina Vasilieva**

National Medical Research Centre for Phthisiopulmonology and Infectious Diseases

Aspergillosis is mainly diagnosed in chronic forms of tuberculosis with destruction of lung tissue and complicates its course.

**Objective:** to study the clinical and epidemiological features of co-infection (aspergillosis/tuberculosis) with lung damage.

**Methods:** We examined 101 patients with tuberculosis without HIV infection aged 21–83 years, who were treated in 2019–2020 in the clinic in our center. All patients underwent bronchoscopy with BAL(Bronchoalveolar fluid) sampling for microbiological and mycological studies. The groups were formed after the detection of aspergillosis (main, n = 53) and without it (control, n = 48). The average age in the main group was 46.3± in the control group—39.4 ± l (*p* > 0.05). In the main group, patients over 60 years of age were registered in 24.5%, and in the control group-in 12.5% of cases (*p* = 0.197). In both groups, men predominated by 58.8% and 72.9%, respectively (*p* = 0.083). The patients’ living conditions, social status, clinical forms of tuberculosis, the presence of destruction and damage to the bronchi, the registration group and concomitant pathology, the level of neutrophils and lymphocytes (%) were analyzed. For statistical processing, the exact Fisher test and the Student’s *t*-test with the Levin correction were used.

**Results:** Both groups were dominated by patients living in a city apartment (71.7% and 64.6%, respectively). People of retirement age were found in the main group in 20.8%, and in the control group-in 6.3% (*p* = 0.05). Representatives of other social groups met equally often. No differences were found in the structure of clinical forms of tuberculosis. Limited and widespread lung lesions did not differ in the study groups. The presence of destruction was found in 52.8% and 66.7% (*p* = 0.106), bronchial lesions in 33.3% and 36.0% (*p* = 0.871). We noted that co-infection (aspergillosis and tuberculosis) was detected in 47.2% of newly diagnosed patients, and tuberculosis lesions without aspergillosis occurred in half of the cases (*p* = 0.629), that is, we can assume a change in the pathomorphosis of the infection. Patients with chronic viral hepatitis (17.0% and 12.5%) and hypertension (7.5% and 12.5%) predominated among the comorbidities; diabetes mellitus, bronchiectatic disease and other diseases were registered in isolated cases in both groups. In the blood parameters, the level of neutrophils was 53.9 ± and 55.3 ± (*p* > 0.05), lymphocytes 29.3 ± and 28.0 ± (*p* > 0.05), respectively.

**Conclusions:** It was revealed that co-infection in 20.8% of cases was registered in persons of retirement age without clinical and laboratory signs of immunodeficiency. Registration of aspergillosis existed not only among patients with chronic tuberculosis, but also in newly diagnosed patients. This fact may indicate the pathomorphosis of the infection, the spread of aspergillosis in society. Other clinical and epidemiological features in patients with co-infection (aspergillosis/tuberculosis) were not revealed by us. Therefore, mycological examination should be carried out in all patients with tuberculosis of the respiratory system together with microbiological examination during bronchoscopy.

## P081 Polyol Is an Unusual Marker of *Malassezia* spp. Revealing under MALDI-TOF-Mass-Spectrometry

**Tatyana Bogdanova ^1^, Igor Riabinin ^1,2^, Andrey Alekseev ^1^ and Natalya Vasilyeva ^1,2^**

^1^ Department of Medical Microbiology, North-Western State Medical University n.a. I.I. Mechnikov
^2^ Kaschkin Research Institute of Medical Mycology, North-Western State Medical University n.a. I.I. Mechnikov

**Objectives:** The aim of the study was the revealing of peaks in identification MALDI-mass-spectra of Malassezia cells, which is most likely to have a non-polypeptide nature.

**Materials & Methods:** 113 *Malassezia* spp. strains were isolated from skin of volunteers and domestic animals (dogs, cats) and identified according to Ramadan S., 2012. Cultures were grown on modified Leeming-Notman medium at 32 °C for 5–7 days for mass-spectrometry of cell biomass. Cultures’ materials were prepared according to the manufacturer’s protocol with double treatment with formic acid under “sandwich” scheme. MALDI-TOF-mass-spectrometry was performed on Autoflex speed TOF/TOF (Bruker Daltonik GmbH, Germany) in the MBT-mode. The resulting mass-spectra were processed in flexAnalysis.

**Results:** About 4.4% of the studied cultures had peculiar combinations of peaks in the low molecular weight range distinguishable up to about 3.5 kDa along with typical protein and peptide peaks (see Figure 1). The combinations had the form of rhythmically repeating signals describing by apexes of a somewhat distorted parabolic contour. The structure of such complexes resembled a trace from the fragmentation of a polymer compound. To clarify the structure of this substance, the measurements of the molecular masses differences of ions (distance between the peaks) were made. ΔMr values were about 18–32 Da. Annotation made it possible to establish that such fragments belong to the =CH2 and =CH-OH groups located irregularly.

**Figure 1.** Low-mass segment of MALDI-mass-specter from *Malassezia sympodialis* culture. MS-visualization in flexAnalysis.

**Conclusions:** The genus Malassezia including 18 species which are identifying correctly by DNA-sequencing. MALDI-TOF-MS-based strategy for *malasseziae* identification is perspective, but there’re some difficulties, including the selection of optimal cultivation mode, low completeness of commercial main-spectral-profiles databases, as well as the peculiar chemical composition of *malasseziae* cells. Based on the obtained results we determined the compounds that form peculiar peak complexes as polyols of possibly linear structure. Most likely they are the products of the polyunsaturated fatty acids hydroxylation. Among representatives of >40 micromycetes genera similar findings were seen only in single strains of *Meyerozyma guilliermondii* and *Phialophora verrucosa*. We consider this metabolite as the species- or strain-specific biomarker for MALDI-identification of *Malassezia* spp. The most enigmatic fact remains the appearance at the time of MALDI-ionization in MBT-mode of chemical energy of such magnitude that is able to break carbon-carbon bonds.

## P082 Antifungal Zone Diameter and MIC Correlation and Categorical Agreement for *Candida auris* Isolates from Pakistan

**Joveria Farooqi, Sadaf Zaka, Faheem Naqvi and Kauser Jabeen**

Aga Khan University

**Objective:** We determined correlation of zone diameters (ZD) of fluconazole (FLU) and caspofungin (CAS) with their respective MICs and CAS ZD with anidulafungin (ANI) and micafungin (MCF) minimal inhibitory concentrations (MIC). We also determined categorical agreement between the above pairs.

**Methods:** Data on *Candida auris* isolates was retrieved from the Aga Khan University Laboratory database from January 2020–April 2021. First isolates of *Candida auris* per patient from blood culture or other clinically significant sites were selected for analysis. The identification of *C. auris* was based on a combination of colony morphology and biochemical identification on API 20C AUX and Vitek2 system. Antifungal susceptibility was performed by broth microdilution on Sensititre® YeastOne and interpreted according to CDC guidelines for FLU, CAS, MCF and ANI. Disc diffusion was performed using Oxoid® discs with 25 µg FLU and 5 µg CAS on Mueller Hinton Agar (Oxoid, UK) with 2% dextrose and methylene blue and results recorded on worksheets but not reported to the patient. Zone diameter interpretation of <12 mm was taken as resistant for both FLU and CAS from Nunnally et al., (JCM 2021).

**Results:** A total of 118 isolates were identified, with 76 unique isolates. There were 58 (76.3%) male patients, median age was 63.5 years (IQR: 42.5–75.5). Nine (11.8%) isolates were from the community, 74 (97.3%) originating from Karachi. Fourteen patients (18.4%) were admitted in the ICU, while 24 (31.6%) had central lines. The isolates were from urine [41 (53.9%)], blood [22 (28.9%)], central venous catheter tips [6 (7.9%)], pus and tissue [n = 5 (6.6%)], and ear swabs [n = 2 (2.6%)]. Susceptibilities on MIC were available for 74 isolates for all drugs, on ZD 68 for FLU and 36 for CAS. There were 63/68 (82.9%) categorised as FLU resistant on ZD and 57/74 (75%) on MIC, none as CAS resistant on ZD, 10/74 (13.2%) on MIC, 1/72 (1.4%) as MCF resistant, and none as ANI resistant.

The median (IQR) for FLU ZD was 0 mm (0), CAS ZD 23 mm (20–26.75), FLU MIC 48 µg/mL (32–256), CAS MIC 0.12 µg/mL (0.12–0.25), ANI MIC 0.25 µg/mL (0.12–0.25) and MCF MIC 0.12 µg/mL (0.06–0.12). Spearman correlation results were FLU ZD-MIC (−0.032, *p* = 0.798), CAS ZD-MIC (−0.225, *p* = 0.187), CAS ZD-ANI MIC (−0.207, *p* = 0.225), CAS ZD-MCF MIC (−0.245, *p* = 0.162). There were 16/17 (94.1%) major errors and 4/51 (7.8%) very major errors for FLU, agreement 70.5%, while it was 30/36 (83.3%) for CAS, all discrepancies being major errors. CAS ZD, however, showed 100% agreement with ANI and MCF as there were no ANI and MCF resistant isolates among the 36 tested on disc.

**Conclusions:** Unlike Nunnally et al, *C. auris* ZD of FLU and CAS do not correlate well with their MICs, nor CAS with MIC of ANI or MCF in isolates from our center. Categorical agreement is also low for FLU and CAS, but should be explored further between CAS ZD and ANI and MCF MIC for resistant isolates.

## P083 Direct Method for Rapid Identification of Candida Species from Fungus-Positive Bottles by Matrix-Assisted Laser Desorption Ionization Time-Of-Flight Mass Spectrometry

**Anna Malchikova and Galina Klyasova**

National Recearch Center For Hematology

**Objectives:** Reduction of time to the identification of *Candida* from fungus-positive bottles is a predictor of survival in patients with candidemia. In the study, we evaluated in-house method for rapid identification of *Candida* species from fungus-positive bottles and then compared it with routine conventional culture-based identification.

**Materials & Methods:** Prospective study was performed from 2016 to 2020 at the National Research Center for Hematology, Moscow. During the study period, all blood cultures (BC) bottles obtained from hematological patients were incubated in the BACTEC FX system (Becton Dickinson, USA) for microorganism growth monitoring. When BC bottle was detected as positive, the blood culture broth was deposited on a glass slide to be subjected to Gram staining. If yeast cells were detected by microscopy, the in-house method was used for the identification of yeasts. For that, BC media (5–6 mL) was transfer from fungus-positive bottles into MONOVETTE Serum Z (Sarstedt, Germany) with inert gel. In-house method included series section steps consisted from centrifugation, physically separates the blood cells and adding of 0.1% sodium dodecyl sulfate [1]. Routine conventional culture-based identification on Sabouraud dextrose agar (Oxoid, UK) was used simultaneously with the in-house method.

**Results:** During the study period, 14 fungus-positive bottles were obtained. The in-house method resulted in 78.6% (11/14) and 64.3% (9/14) identification rate at the genus and species level of *Candida*, respectively (Table 1). The identification of Candida to the species level was proofed in all cases by routine conventional culture-based method. Median time from the start of vials incubation in BACTEC FX system to identification of *Candida* by in-house method was shorter than by conventional culture-based identification and composed 38 h 05 min vs. 60 h 54 min (*p* = 0.039). The time spending to the identification of *Candida* by in-house method was 57 min, while by using of conventional culture-based identification was prolonged to 37 h.

**Conclusions:** Our in-house method was found to be in a good agreement with routine conventional culture-based identification. This technique is quick and consistent to identify *Candida* from fungus-positive bottles in less than 60 min, has a low cost in its implementation and provides helpful information for physicians when it comes to target therapy.

**Reference**

1. Klyasova, G.A.; Malchikova, A.O.; Dzhulakyan, U.L. RF Patent No. 2 739 758, 2020.

## P084 The Role of (1–3) -Β-D-Glucan in the Diagnosis of Invasive Aspergillosis in Patients with COVID-19 Disease

**Anna Katsiaflaka ^1^, Aikaterini Oikonomou ^2^, Ioanna Voulgaridi ^1^, Dimitrios Papadopoulos ^2^, Sotirios Papaoikonomou ^1^, Panagiotis Papamichalis ^2^, Apostolos Komnos ^2^, Maria Mavrouli ^3^, Georgia Vrioni ^3^ and Athanasios Tsakris ^3^**

^1^ Microbiology Laboratory, General Hospital Of Larissa
^2^ Intensive Care Unit, General Hospital of Larissa
^3^ Department of Microbiology, Medical School, National and Kapodistrian University of Athens

**Objective:** Invasive aspergillosis is a well-defined clinical entity in immunocompromised patients. There are certain criteria for classification of the diagnosis as “proven”. Microbiological criteria and biomarkers can classify a diagnosis as ”possible” when they are present in combination with clinical evidence and radiological findings. COVID-19 disease often leads to acute respiratory failure with a variety of complications, one of which is invasive aspergillosis, possibly in the context of immune—paralysis due to hypoxia and treatment with corticosteroids and immunosuppressants.

**Materials & Methods:** Thirteen patients with COVID-19 disease admitted to an intensive care unit (ICU) of a secondary hospital were studied over a period of two months. There was a deterioration both in clinical condition and laboratory parameters of patients, with acute respiratory failure and findings consistent with possible invasive aspergillosis on computed tomography.

The diagnosis of COVID-19 in patients was made by SARS CoV-2 rRT- PCR. During their hospitalization in the ICU, repeated cultures of bronchial secretions, urine and blood were performed, none of which led to growth of fungi. With deterioration of respiratory function, a CT scan was performed, which showed “ground glass” image or pulmonary cavities, while in two patients there was additionally hemoptysis. In the differential diagnosis, possible invasive aspergillosis was suspected. *Aspergillus* DNA was detected in bronchial secretions and in serum (Standard Real-Time PCR detection kit for *Aspergillus*, Primerdesign™Ltd, genesig®kit) and galactomannan antigen (Platelia *Aspergillus*, Bio-Rad, Hercules, CA, USA) and (1-3)-β-D-Glucan (Dynamiker Biotechnology Co., Ltd., Tianjin, China) were detected in patients’ sera by ELISA.

In the GM immunoenzymatic Platelia *Aspergillus* method, positive samples were considered those with a cut-off index ≥0.5. In the Dynamiker Fungus (1-3)-β-D-Glucan spectophotometry assay, positive serum samples were considered those with β-D-Glucan >95 pg/mL and inconclusive those with β-D-Glucan 70–95 pg/mL.

**Results:** Of all patients included, four showed no positive markers and the respiratory deterioration was attributed to another etiology. Of all the other patients, three were detected with positive (1-3)- β-D-Glucan antigen and two with equivocal result but positive molecular analysis in clinical samples, while it is noteworthy that none of them detected a positive galactomannan aspergillosis index. As the case may be there was a positive or negative Real Time PCR for *Aspergillus* DNA in bronchial secretions and serum.

**Conclusions:** Patients with COVID-19 disease develop immunosuppression due to viral infection and treatment, thus having an increased risk of developing aspergillus infection which greatly increases the mortality rate. The diagnosis of invasive aspergillosis is based on clinical, laboratory and imaging criteria. Unlike most invasive aspergillosis cases, in which the fungus is detected or isolated in cultures and galactomannan antigen is detected, in patients with COVID-19 it appears that the “ground glass” CT image and the detection of β-D-Glucan antigen may promptly lead to appropriate initiation of antifungal treatment. A larger number of patients with COVID-19 and invasive aspergillosis is required to confirm whether (1-3)-β-D-Glucan antigen detection in these patients may be a useful biomarker in the diagnosis of invasive aspergillosis in the absence of galactomannan.

## P085 Molecular Identification of *Candida* Species Isolated from Candiduria in Hospitalized Patients

**Mojtaba Nabili ^1^ and Maryam Moazeni ^2^**

^1^ Department of Medical Laboratory Sciences, Faculty of Medicine, Sari Branch, Islamic Azad University, Sari, Iran
^2^ Invasive Fungi Research Center, Communicable Diseases Institute, Mazandaran University of Medical Sciences, Sari, Iran

**Objectives:** The incidence of candiduria caused by *Candida* spp. has increased in recent years, particularly in hospitalized patients. Candiduria is most commonly caused by *C. albicans*, however during last decades an increase in non *albicans* species were also observed. The purpose of this study was determine the molecular identification of Candida species isolated from candiduria in hospitalized patients.

**Materials & Methods:** This cross-sectional study was conducted on 530 hospitalized patients at the selected Mazandaran Hospitals. Midstream urine sample was cultured on CHROMagar Candida culture medium. Molecular identification of common *Candida* species was carried out according to PCR-RFLP technique after *MspI* restriction enzyme digestion. *C. albicans* and *C. parapsilosis species* complexes were identified by amplification of the *HWP1* and intein-containing vacuolar ATPase precursor genes respectively.

**Results:** The frequency of candiduria was estimated at 14% among hospitalized patients of whom (n = 65; 87.8%) patients were female and (n = 9; 12.2%) were male. The most common underlying diseases were diabetes (n = 36; 48.6%). The most common isolates were *C. albicans* complex (n = 44; 59.4%) followed by *C. glabrata* (n = 16; 21.6%), *C. tropicalis* (n = 10; 13.5%), *C. Krusei* (n = 3; 4%) and *C. parapsilosis* (n = 1; 1.3%).

**Conclusions:** In current study, conventional and molecular methods were used to identify *Candida* species and almost similar results were obtained. However, accurate identification of *Candida* spp. requires the use of molecular techniques such as PCR-RFLP, HWP1 and intein-containing vacuolar ATPase precursor genes. But we can use chromogenic methods such as CHROMagar Candida for diagnosis of *Candida* spp. in laboratories with limited resources.

## P086 Diagnostic Performance of Unyvero Multiplex PCR in *Pneumocystis jiroveci*

**Khaled Alobaid ^1,2^ and Nadia Alenezi ^1,2^**

^1^ Mycology Reference Laboratory, 06010 Bristol, UK
^2^ Parasitology Unit, Jabriya 47060, Kuwait

**Objectives:** To evaluate the diagnostic performance of a commercial multiplex PCR (Unyvero, Curetis, Holzgerlingen, Germany) in the diagnosis of *Pneumoystis jiroveci* pneumonia (PCP) through comparison with direct immunofluorescent assay (Meriflour Pneumocystis, Meridian biosccience, Cincinnati, OH, USA)

**Materials & Methods:** 32 bronchoalveolar lavage (BAL) clincal samples submitted to Parasitology laboratory for PCP diagnosis were processed as follow: each BAL sample is divided into two portions. First portion is centrifuged at 2000 RPM per 10 min, then sediment is aspirated for further processing. In brief, two drops from the sediment are placed in a slide, prepared and treated with detection reagent containing monoclonal antibodies and then examined using fluorescent microscope. The second portion of BAL (180 µL uncentrifuged sample) is transferred to Unyvero sample tube, lysed, then placed in Unyvero respiratory cartilage which is inserted in the analyzer. Results are read after 4–5 h.


**Immunofluorescent Microscopy**

**Multiplex PCR**
Positive 106Negative 2226Total 3232

**Results:** Direct immunofluorescent assay has 100% sensitivity, while multiplex PCR has 60% sensitivity.

Multiplex PCR is 100% specific, while direct immunofluorescent assay 84% specific.

**Conclusions:** Multiplex PCR is more specific for the diagnosis of PCP, less subjective and provides fungal load which helps in accurate interpretation and facilitate prognostic assessment.

## P087 Analysis of Proteinuria Data to Investigate the Prevalence, Magnitude and Etiology of Proximal Renal Tubulopathy in Patients with Hematoproliferative Disorders

**Christoph Fux ^1^, Peter Lanz ^1^, Ramona Merki ^2^ and Mario Bargetzi ^2^**

^1^ Department of Infectious Diseases and Hospital Hygiene
^2^ Department of Hematooncology, Kantonsspital Aarau

**Objectives:** Clinical experience suggests that patients with hematoproliferative disorders undergoing chemotherapy frequently have proximal renal tubulopathies (PRT) which occasionally leads to severe dyselectrolytemia or Fanconi syndrome. We investigate the hypothesis that PRT is frequent, but overlooked in routine surveillance, as it initially does not result in alterations of serum creatinine levels, the standard parameter used for renal monitoring. The aim of this study is evaluating the prevalence and magnitude of PRT in patients with hematoproliferative disorders before and under chemotherapy.

**Methods and materials:** Laboratory data collected at the Kantonsspital Aarau between 2011 and 2019 were used. Prevalence and magnitude of PRT were determined by urine protein profiles. PRT dynamics were analyzed relative to chemotherapies and aplasia. Additional factors including blood and urine parameters, nephrotoxic drug exposure, comorbidities and type of neoplasia were considered in multivariable analyses to define risk factors. Descriptive statistics were performed using STATA 12.

**Results:** We analyzed 145 patients (median age 60 years, 63% male) suffering from MDS (n = 9), AML (n = 76), ALL (n = 16), aggressive Lymphoma (n = 39), MM (n = 6) among others. PRT was found in 18/96 patients (19%) before, in 43/70 (61%) after the first and 21/24 (88%) after the second chemotherapy. Recovery was observed after both chemotherapies. α1-microglobulin showed fewer and lesser increases as measured by multiples of cutoff (MOC) than retinol-binding protein. The extent of PRT inversely correlated with serum potassium, phosphate and uric acid as well as the requirement for potassium substitution. PRT occurred without eGFR impairment. AML and associated chemotherapies correlated with PRT with an OR of 5.22 (*p* = 0.05).

**Conclusions:** Extensive potassium substitution required for patients with hematoproliferative diseases in aplasia is not only related to intestinal losses, but a consequence of relevant PRT, which is higly prevalent in this population. Tubulotoxic drugs such as Amphotericin B, aminoglycosides or Tenofovir TDF therefore have to be administred with great caution. Amilorid may be a therapeutic option to overcome hypopotassemia in this setting.

## P088 First Trial Technique of Non-Invasive, In-Situ Fluorometric Detection of Onychomycosis

**Saeideh Amani Ghayyoum and Behnam Mohammadi Ghalehbin**

Department of Microbiology, Medical Parasitology & Mycology, School of Medicine, Ardabil University of Medical Sciences

**Objective:** *Onychomycosis* is the most common fungal infection of the nail that is caused by 3 different types of fungi including dermatophytes, yeasts and other molds. It causes near 30% of fungal infections of the skin and nails. Diagnosis of this infection is made by scraping the infected plate and laboratory tests and culture, which is the painful and long process for the patient and has bias in test results. And in cases where sampling is not done properly, it will lead to false negative results. The aim of this study is to compare common mycological diagnostic techniques with direct fluorescence induction in nail tissue without scrapping and to develop the new diagnostic method.

**Method & Materials:** At first we ask patients to wash and dry the nails and finger then put the infected nail in a clarifying composition for 5–10 min and in the suspension containing 1–2% calcofluoride resprctively. After that the finger placed inside the dark chamber of ONYCFLUO device. The process of detection revealed after short irradiation of ultraviolet light and in case of presence of fungi in the nail tissue fluorescent plate readers measure the light signals emmited by UV. The diagnostic results of fluorescence technique in nail tissue were compared with the results of direct light microscopy and analysed. For quality control cellulose and external chitin were removed.

**Result:** following the binding of calcofluor to the chitin of fungus cell wall exciting/emitting fluorescence at a wavelength of 370–475 nanometer will be generated which can be identified and reported by the sensitive spectrometer system of the device. Additional examination showed fluorescence emission spectrum of the calcofluor solution in the citrate-phosphate buffers (pH 7.30) was remarkably sharper and more detectable.

**Conclusions:** ONYCHFLUO as a fluorometric detection Device is applicable to use in physician offices and dermatology clinics, even veterinary centers for accelerating diagnostic approaches of *Onychomycosis*. No need for fluorescent microscopy and no need to scrapping of nail tissue through painful sampling, which was one of the difficulties in diagnosing this infection that consider as advantages of mentioned technique. Due to safety of ingredients the process is harmless on skin and it is not prevented by ethics.

## P089 Multicenter Evaluation of the VirClia Galactomannan Assay on Bronchoalveolar Lavage Fluid from Patients with Hematological Malignancies

**MD Jochem Buil ^1,2^, Sammy Huygens ^3^, Alexander Schauwvlieghe ^5^, Albert Dunbar ^3^, Marijke Reynders ^6^, Fatima Zohra Delma ^1^, Elizabeth de Kort ^1,5^, Willem Melchers ^1,2^, Bart Rijnders ^3^ and Paul Verweij ^1,2^**

^1^ Department of Medical Microbiology, Radboud University Medical Center

^2^ Radboudumc-CWZ Center of Expertise for Mycology

^3^ Department of Medical Microbiology and Infectious Diseases, Erasmus University Medical Center

^4^ Department of Hematology, Radboud University Medical Center

^5^ Department of Hematology, Ghent University Hospital

^6^ Department of Laboratory Medicine, Medical Microbiology, AZ St-Jan Brugge-Oostende Hospital

**Background:** Culture and microscopy lack sensitivity to diagnose invasive aspergillosis (IA), and may require up to 7 days before the culture becomes positive. Biomarkers, such as galactomannan (GM) and *Aspergillus* PCR, have increased the sensitivity and reduced turn-around time. However, the emergence of IA in patients with severe influenza or COVID-19, has increased the need for on demand tests.

Recently several point-of-care assays became available that can be used both on single patient samples without the need to batch, such as *Aspergillus* lateral flow device tests. These tests are rapid but give semi-quantitative results. Recently the VIRCLIA® Galactomannan AG assay was developed which can be used on individual samples and provides a quantitative result within 1.5 h. This new assay detects GM by an automated chemiluminescence immunoassay.

**Material/methods:** BALf samples from patients with hematological malignancies or stem cell transplants were collected between January 2017 and July 2021 in 2 academic centers in the Netherlands (Radboud UMC and Erasmus UMC), and 2 centers in Belgium (UZ Gent and AZ St. Jan Bruges). Samples were taken as part of routine clinical care. Informed consent was obtained for the use of residual material for research purposes. Samples were stored at −70 °C until tested.

In the main analysis, patients were classified according the 2020 European Organization for Research and Treatment of Cancer Invasive Fungal Infections Cooperative Group (EORTC)/Mycoses Study Group (MSG) consensus definitions as having proven, probable or probable IA. Proven and probable IA were defined as cases, while possible IA and unclassifiable patients were used as controls. In the secondary analysis, GM was excluded as criterion to classify patients. All results were analyzed using the cutoff of 1.0 OID for Platelia, and 0.2 OID for VirClia.

**Results:** A total of 79 patients were included (39 patients with probable IA, 36 with possible IA, and 4 patients were not classifiable). The Platelia was positive in 24 samples, while the VirClia was positive in 30 samples (Table 1). Of the 7 Platelia negative, but VirClia positive samples, 3 patients had probable IA based on a positive *Aspergillus* PCR. Of the 4 other patients, 3 patients had a negative *Aspergillus* PCR, while no PCR was performed in 1 patient. The agreement (POS/NEG) between the Platelia and VirClia was 89%. The results of Platelia an Virclia are shown in Figure 1. The VirClia detected 69% (27/39) and the Platelia detected 59% (23/39) of patients with probable IA.

29 of 39 patients had a probable IA if GM was excluded as a mycological criteria. The VirClia detected 69% (20/29) of probable IA cases and the Platelia 62% (18/29).

**Conclusions:** The Virclia GM showed a good correlation with the Platelia. The sensitivity of the VirClia to detect IA was comparable to the Platelia, also after GM was excluded as mycological criterium. The VirClia is a promising new assay, which decreases the time to result compared to the conventional Platelia assay.

## P090 Validation of Colibrí for Automated Preparation of MALDI-TOF Targets for Yeast Identification

**Robbe Heestermans, Pauline Herroelen, Kristof Emmerechts, Kristof Vandoorslaer, Ingrid Wybo, Denis Piérard and Astrid Muyldermans**

Department of Microbiology and Infection Control, Universitair Ziekenhuis Brussel, Vrije Universiteit Brussel (VUB)

**Objectives:** Invasive candidiasis in hospitalized patients is a growing challenge worldwide as it is associated with high morbidity and mortality. Given the increasing incidence of candidemia caused by non-*albicans* species, a rapid and reliable identification of the causative pathogen is of major importance to guide therapeutic choices. Recently, Copan (Italy) introduced the Colibrí^TM^ system for automatized colony picking and preparation of MALDI-TOF target plates. However, this system has not yet been validated for yeast identification.

**Materials & Methods:** Fifty *Candida* strains were selected to evaluate accuracy of Colibrí^TM^: *C. albicans* (n = 7), *C. glabrata* (n = 9), *C. parapsilosis* (n = 7), *C. tropicalis* (n = 8), *C. krusei* (n = 2), *C. guilliermondii* (n = 6), *C. dubliniensis* (n = 9) and *C. auris* (n = 2). For each strain, a Sheep Blood Agar plate supplemented with X and V factors (HEM plate) and a Sabouraud agar plate (SAB plate) were inoculated and incubated by WASPlab® (Copan, Italy). After 18 h and 36 h of incubation, isolates were spotted in parallel by Colibrí^TM^ and manually from each culture plate onto a MALDI target plate (Bruker, Germany) with addition of formic acid. Acceptance criterium for identification (ID) by MALDI Biotyper® (Bruker, Germany) was set on 1.8.

**Results:** Sufficient growth after 18 h incubation was observed for 36/50 HEM and 44/50 SAB plates. Overall, using Colibrí^TM^ for colony picking, 86% of strains cultured on HEM plates were identified with an acceptable ID score compared to 81% manually. SAB plates showed inferior results for both Colibrí^TM^ (66%) and manually (61%). Further detailed results are provided in Table 1. When evaluating the accuracy of Colibrí^TM^, there was an overall agreement of 86% for identification of strains on HEM plates between Colibrí^TM^ and the manual method after 18 h and 84% after 36 h. For SAB plates, an agreement of 77% after 18 h and 89% after 36 h was observed. With exception of *C. dubliniensis* and *C. tropicalis*, all included *Candida* species showed a 100% accuracy for Colibrí^TM^ on HEM plates. Detailed results for accuracy are shown in Table 2.

**Conclusions:** We observed a good agreement between Colibrí^TM^ and the manual reference method. These results demonstrate that Colibrí^TM^ is a reliable system for MALDI-TOF target preparation for yeast identification. The higher degree of standardization and lower hands-on time associated with its use means an important advantage for clinical microbiology laboratories in an era of increasing automatization.

## P091 PCR-Based Detection of Azole Resistance in *A. Fumigatus* to Improve Patient Outcome. A Prospective Multicenter Study

**Albert Dunbar ^1^, Sammy Huygens ^1^, Corné Klaassen ^3^, Willemien Zandijk ^3^, Alieke Vonk ^3^, Walter van der Velden ^4^, Paul Verweij ^5^, Jochem Buil ^5^, Nick de Jonge ^6^, Karin van Dijk ^7^, Bart Biemond ^8^, Aldert Bart ^9^, Anke Bruns ^10^, Pieter-Jan Haas ^11^, Astrid Demandt ^12^, Guy Oudhuis ^13^, Peter von dem Borne ^14^, Martha van der Beek ^15^, Saskia Klein ^16^, Peggy Godschalk ^17^, LFR Span ^18^, Martijn Bakker ^18^, Greetje Kampinga ^19^, Johan Maertens ^20,21^, Katrien Lagrou ^21,22^, Toine Mercier ^20,21^, Ine Moors ^2^, Jerina Boelens ^23^, Dominik Selleslag ^24^, Marijke Reynders ^25^, Jeanette Doorduijn ^26^, Jan Cornelissen ^26^, Alexander Schauwvlieghe ^2^ and Bart Rijnders ^1^**

^1^ Department of Internal Medicine, Section of Infectious Diseases and Department of Medical Microbiology and Infectious Diseases, Erasmus MC, University Medical Center
^2^ Department of Hematology, Ghent University Hospital
^3^ Department of Medical Microbiology & Infectious Diseases, Erasmus MC, University Medical Center
^4^ Department of Hematology, Radboud University Center
^5^ Department of Medical Microbiology, Radboud University Center
^6^ Department of Hematology, Amsterdam University Medical Center, location VUmc
^7^ Department of Medical Microbiology, Amsterdam University Medical Center, location VUmc
^8^ Department of Hematology, Amsterdam University Medical Centers, location AMC
^9^ Department of Medical Microbiology, Amsterdam University Medical Center, location AMC
^10^ Department of Medical Microbiology, University Medical Center Utrecht
^11^ Department of Medical Microbiology, University Medical Center Utrecht
^12^ Department of Hematology, Maastricht University Medical Center
^13^ Departmen t of Medical Microbiology, Maastricht University Medical Center
^14^ Department of Medical Microbiology, Leiden University Medical Center
^15^ Department of Hematology, Leiden University Medical Center
^16^ Department of Hematology, Meander Medical Center, Amersfoort
^17^ Department of Medical Microbiology, Meander Medical Center
^18^ Department of Hematology, University Medical Center Groningen
^19^ Department of Medical Microbiology, University of Groningen, University Medical Center Groningen
^20^ Department of Hematology, University Hospitals Leuven
^21^ Department of Microbiology, Immunology and Transplantation, KU Leuven
^22^ Department of Laboratory Medicine and National Reference Centre for Mycosis, University Hospitals Leuven
^23^ Department of Medical Microbiology, Ghent University Hospital
^24^ Department of Hematology, AZ St-Jan Brugge-Oostende Hospital
^25^ Department of Laboratory Medicine, Medical Microbiology, AZ St-Jan Brugge-Oostende Hospital
^26^ Department of Hematology, Erasmus University Medical Center

**Objectives:** Invasive aspergillosis (IA) is the most common mould infection in patients treated for a haematological malignancy. Azole resistant *Aspergillus fumigatus* is increasingly reported and associated with high mortality. Phenotypic susceptibility testing of fungi is time-consuming, not widely available and cultures often remain negative. AsperGenius® is a commercial multiplex real-time polymerase chain reaction (PCR) that allows for simultaneous detection of *A.* species, *A. fumigatus* and *A. terreus*. In *A. fumigatus* PCR positive samples, the 2 most common azole resistance associated mutation patterns (RAMs) in the *cyp51A* gen (TR34/L98H- TR46/T289A/Y121F) are also detected. The PCR can be performed directly on broncho-alveolar lavage fluid (BALf) and thus diagnose azole resistance more frequently (i.e., in culture negative IA as well) and faster (if done ad hoc) compared with phenotypic testing. In this study we evaluated the clinical value of this PCR in patients with a haematological malignancy with suspicion of IA undergoing BALf sampling.

**Materials & Methods:** 10 centers enrolled patients in the Azole Resistance Management Study (AzoRMan). Patients with pulmonary radiological abnormalities suspected for an invasive fungal infection underwent BALf sampling for fungal culture, galactomannan (GM) and PCR testing. Only patients without antifungal therapy or on triazole monotherapy for <120 h were included. When no mutations were detected in the *A. fumigatus cyp51A* gen, triazole monotherapy was initiated or continued while the detection of resistance by culture or PCR led to a switch to liposomal amphotericin-B. The main objectives of the study were (1) evaluate the impact of PCR-based resistance testing on the management and outcome of patients with IA and (2) systematically evaluate the incidence of triazole resistance in *A. fumigatus* caused by the 2 most frequent triazole RAMs in patients with culture positive, as well as culture negative IA.

**Results:** 323 patients were enrolled and BALf was available in 321 patients. BALf GM was positive (OD ≥ 1.0) in 72/321 (22%) and <0.5 in 217/321 (68%), Table 1. Sufficient BALf remained for PCR testing in 295. *Aspergillus* species DNA was detected in 114/295 (39%) while *A. fumigatus* DNA could be demonstrated in 86 (29%). In those with a GM ≥ 1.0, the species PCR was positive in 45/62 (73%) vs. 53/203 (26%) when GM was negative (i.e., <0.5). In 86 patients with a positive *A. fumigatus* PCR, the resistance PCR was successful for both RAMs in 67/86 (78%). In 7/67 (10.4%) RAMs were documented, 4 with a TR34/L98H, 2 with a TR46/T289A/Y121F and 1 with a mixed wildtype/TR34 infection. In 2 of these 7, GM was <0.5 (1 also culture negative) and in 3/7the fungal culture was negative and therefore resistance was detected by PCR only.

**Conclusions:** Even in countries with a rather high prevalence of azole resistance, the systematic use of a *cyp*51*A* resistance PCR on BALf resulted in relatively few (3 of 295) additional cases of azole resistant *A. fumigatus* detected compared with culture-based testing alone. A stepwise approach in which GM and *Aspergillus* PCR testing is done first followed by resistance PCR when *Aspergillus fumigatus* DNA is detected appears reasonable. The clinical relevance of *Aspergillus* DNA that was detected in 26% of GM negative BALf samples requires further study.

## P092 Mutation Analysis of the Squalene Epoxidase Gene of Dermatophytes in Dubai, Emirates, Using the DermaGenius® Resistance PCR Kit

**Silke Uhrlass ^1^, Srikumar Goturu ^2^, Stephanie Dessoi ^3^, Shyam B. Verma ^4^, Yvonne Graeser ^5^, Daniela Koch ^1^, Hanna Muetze ^1^, Constanze Krueger ^1^ and Pietro Nenoff ^1^**

^1^ Laboratory of Medical Microbiology
^2^ Dr. Joseph’s Polyclinic
^3^ Dermatological Office Dres. med. Gassenmaier
^4^ “Nirvan” and “In Skin” Clinics
^5^ Charite—Universitätsmedizin

**Objectives:** Recalcitrant dermatophytosis due to terbinafine resistant dermatophytes are on the rise in India. The causative agent is mainly *Trichophyton (T.) mentagrophytes* ITS genotype VIII, which is re-classified as *T. indotineae*. This emerging pathogen spreads to other countries worldwide. The way of infection goes from India probably via Arab countries and the Middle and Near East region to Europe. The focus of the study was on investigation the prevalence of *T. indotineae* in Dubai based on precise molecular biological diagnostics of the causative dermatophyte species and genotyping based on sequencing of the fungal DNA. Resistance testing of terbinafine by a breakpoint agar dilution method, and sequencing of the squalene epoxidase (SQLE) gene was compared with results of the new developed DermaGenius® Resistance RT-PCR-Kit (PathoNostic B.V., The Netherlands).

**Methods & Materials:** Patients from Dubai, Emirates, were examined for dermatomycoses caused by dermatophytes. In November 2020, skin scrapings were taken from 75 patients in Dubai with suspicion diagnosis of a dermatophytosis. Cultural diagnostics on Sabouraud’s Dextrose Agar and inhouse PCR for dermatophytes was done. For confirmation of the suspected dermatophyte species, Sanger sequencing of the ITS regions of rDNA genes (mainly the regions ITS 1, 5.8 S rRNA, ITS 2) using universal primers V9G (5′-TTACGTCCCTGCCCTTTGTA-3′) and LSU266 (5′-GCATTCCCAAACAACTCGACTC-3′) was performed for all isolates. All cultures were analysed for mutations in the SQLE gene associated with terbinafine resistance by sequencing, and additional by the commercially available DermaGenius® Resistance Kit. The DermaGenius® Resistance RT-PCR-Kit detects the mutations at position 393 and 397 of the SQLE gene.

**Results:** By culture, from 31 out of 75 skin scrapings, a dermatophyte grew. Sequencing revealed, that 28 samples of 31 cultivated dermatophytes were *T. indotineae*, and 3 samples, only, were T. rubrum. SQLE gene sequencing of *T. rubrum* revealed one terbinafine resistant strain with Phe397Leu amino acid substitution. The other 2 *T. rubrum* strains were wild types without mutations. In the *T. mentagrophytes* group, 3 strains, only, were wild types. The remaining 25 strains showed mutations. The mutations of these 18 terbinafine resistant strains were located at the following positions: Leu393Ser (n = 1), Phe397Leu (n = 15), Gln408Leu (n = 1), and Phe415Cys (n = 1). Three out of 10 sensible strains were wild types. The remaining 7 strains revealed the Ala448Thr mutation and amino acid substitution. By the DermaGenius® Resistance RT-PCR-Kit, all dermatophytes and by sequencing detected mutations at positions Leu393Ser and Phe397Leu could be confirmed. From additional 29 PCR positive skin scraping samples (without cultural growth of a dermatophyte), all 29 dermatophytes confirmed and in 25 for the mutation analysis a valid result could be achieved by DermaGenius® Resistance RT-PCR-Kit. Terbinafine resistance was detected in 16 skin scraping samples, 9 samples were terbinafine sensitive.

## P100 Fungal Contamination of the Water Distribution System in Lagos University Teaching Hospital

**Kolapo Olawale ^1^, Folasade Ogunsola ^1^, Folake Peters ^2^ and Rita Oladele ^1^**

^1^ Department of Medical Microbiology and Parasitology, College Of Medicine, University Of Lagos
^2^ Mycology Unit, Medical Microbiology Laboratory, Lagos University Teaching Hospital

**Objectives:** Fungal contamination of water and its attendant effects e.g., invasive fungal infections has been frequently reported. For infection prevention and control purposes, it is expedient to investigate for the presence of fungal contaminants in water utilized in the hospitals, since the ‘at risk’ populations are managed here. It is on this premise that the water distribution system of Lagos University Teaching Hospital was assessed for fungal contaminants.

**Materials & Methods:** 200 mLs of water each was taken from taps, showers, faucets and water storage tanks. Swab samples were also collected. The hospital sections categorized into low and high risk units. The membrane filtration method for water analysis was used. 100 mL of water sample was filtered through 0.45 µm pore size, 47 mm diameter membrane filter. The filter was placed on SDA plates supplemented with gentamycin and chloramphenicol; which were incubated at room temperature and at 37 °C. Pure colonies from subculture were identified using microscopic and morphological method of identification for fungi as described in existing literatures.

**Results:** One hundred and five (105) water and 49 swab samples were collected for analysis. There was 100% fungal contamination of the hospital water system. Low and high risk units had 25 and 18 different species of fungi isolated respectively. Labour ward and Modular theatre were the most contaminated of the units studied with 25.3% and 43.9% isolates count respectively. *Cladosporium* spp. was the most frequently isolated organism in the low risk units occurring in 7 of 9 units studied while *Aspergillus* was the most predominant genus with 5 species identified including *niger* (9.9%), *terreus* (4.4%), *flavus* (3.3%), *fumigatus* (8.8%) & *versicolor* (2.20%). *Paecilomyces* spp. had the highest percentage of occurrence (6/8) in the high risk units, while *Aspergillus* spp. followed closely with 3 species identified (*flavus*; 9.4%, *fumigatus*; 15.9%, *niger*; 10.3%), the Renal dialysis centre and theatre had the least contamination rates in the high risk units. Accidents and emergency theatre had the highest contamination rate of all the swab samples analysed. *Aspergillus niger, Cephalosporium curtipes, Penicilium chrysogenum* and *Penicilium glabrum were* each identified in 4 of 6 units from which swabs were taken from.

**Conclusions:** Our data points to water as a plausible source of infections in hospitals. A standard protocol for monitoring and regulation of fungi in water needs to be developed particularly for hospitals as a means of infection prevention and control.


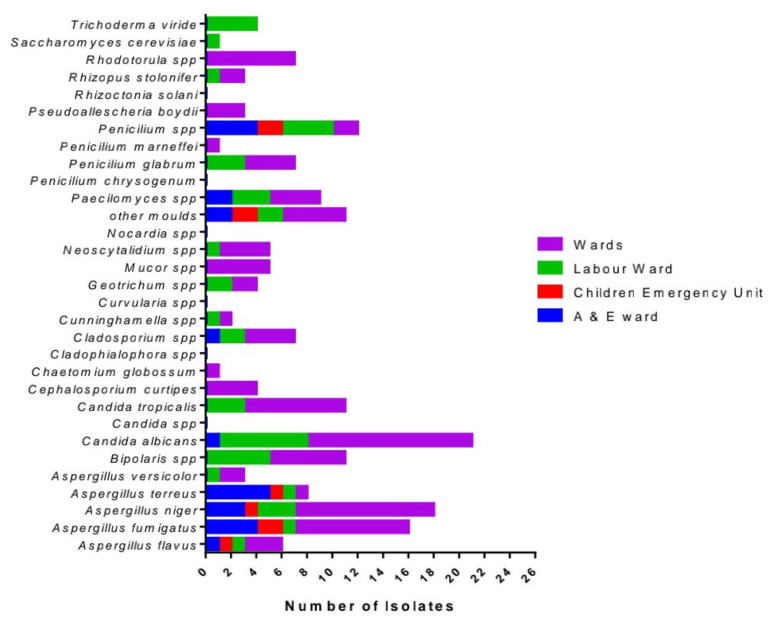


**Figure 1.** Distribution of isolates in water samples from low risk locations.


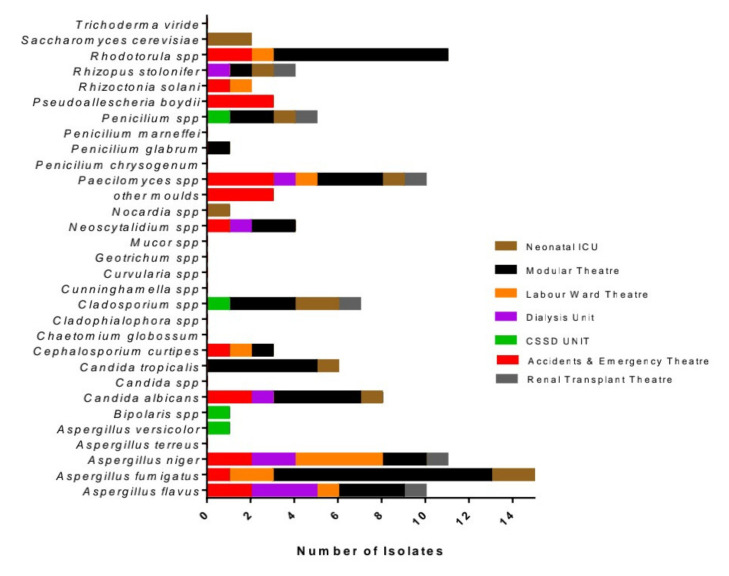


**Figure 2.** Distribution of isolates in water samples from high risk locations.

## P101 Environmental Yeasts, Pollution and Antifungal Resistance

**Maria Lúcia Scroferneker ^1,2^, Danielle Machado Pagani ^3^, Fabiana Vieira Tormente ^3^ and Patricia Valente ^2,3^**

^1^ Postgraduate Program in Medicine: Medical Sciences, Universidade Federal do Rio Grande do Sul
^2^ Department of Microbiology, Immunology and Parasitology, ICBS, Universidade Federal do Rio Grande do Sul
^3^ Postgraduate Program in Agricultural and Environmental Microbiology, Universidade do Rio Grande do Sul

**Objectives:** Verify the presence of antimicrobials and pesticides in a south lagoon in Brazil and isolate, identify and test antifungal susceptibility of yeasts from this envinronemnt.

**Materials & Methods:** The samples were collected in the rainy season and dry season for two years, totaling four collections. The samples were spread in a selective medium containing antifungal (fluconazole, terbinafine, amphotericin B, or caspofungin). The same samples were tested for the presence of agricultural fungicides. Seventy-one yeasts were obtained, and 31 species were identified, subsequently tested for Minimum Inhibitory Concentration (MIC) following the CLSI M27–A3 protocol, and classified as resistant-like when the MIC value was equal to or greater than the breakpoint established for *Candida* spp.

**Results:** Fifty-five isolates were classified as resistant-like for fluconazole (triazole) (MIC 64–2048 µg/mL), 39 for terbinafine (allylamine) (MIC 4–256 µg/mL), 42 for amphotericin B (polyene) (MIC 1–128 µg/mL) and 48 for caspofungin (echinocandin) (MIC between 1–128 µg/mL). All isolates were resistant-like to at least one antifungal. In total, 62 isolates were classified as multidrug-resistant (MIC ≥ at the breakpoint for two or more antifungals). In the water samples, agrodefensive agents were identified, such as carbendazim (benzimidazole) and tebuconazole (triazole).

**Conclusions:** Fungi can cause systemic diseases, either primary or secondary. Fungal diseases are neglected, especially in developing countries. From the observations made, it is possible to infer that resistant yeast selection is taking place in this environment, possibly making it a reservoir of resistance genes. Knowing the effects of human actions on the environment is an important step towards prevention and preparedness to deal with possible outbreaks and pandemics.

## P102 Isolation of *Exophiala phaeomuriformis* from an Aviation Kerosene Sample in Brazil

**Maria Lúcia Scroferneker ^1,2^, Mariane Rodrigues Lobato ^3^, Fátima Menezes Bento ^2,3^, Alessandra Koehler ^1^, Amanda Carvalho Ribeiro ^4^ and Danielle Machado Pagani ^3^**

^1^ Postgraduate Program in Medicine: Medical Sciences, Universidade Federal do Rio Grande do Sul
^2^ Department of Microbiology, Immunology and Parasitology, ICBS, Universidade Federal do Rio Grande do Sul
^3^ Postgraduate Program in Agricultural and Environmental Microbiology, Universidade do Rio Grande do Sul. Universidade Federal do Rio Grande do Sul
^4^ Graduation in Pharmacy, Universidade Federal do Rio Grande do Sul

**Objectives:** Identify the presence of black yeast species in aviation kerosene samples.

**Materials & Methods:** The aviation kerosone samples came from an aviation company in Porto Alegre, state of Rio Grande do Sul, Brazil. The fungi isolation was carried out using the modified ASTM D6974-09 Standard. In this procedure, 500 mL kerosene samples were filtered, in triplicate, under aseptic conditions, under vacuum, using a 0.22 μm membrane (Millipore). After filtering, the membranes were deposited in potato dextrose agar culture medium. The plates were incubated in an oven at 30 °C for seven days. After this period, the colonies were isolated in new culture media for purification of the isolates. Molecular identification was performed through the sequencing of ITS DNA regions. The profile of sensitivity to clinical antifungals was assessed following protocols M27-A3 of the Clinical and Laboratory Standards Institute (CLSI). The minimum inhibitory concentrations (MIC) of seven antifungal agents were determined: ketoconazole, itraconazole, posaconazole and voriconazole (16–0.03 μg/mL), terbinafine (0.5–0.001 μg/mL), caspofungin (8–0.015 μg/mL), and amphotericin (16–0.0313 μg/mL).

**Results:** We were able to isolate the black yeast *Exophiala phaeomuriformis* from the kerosene samples. The MIC values were the following: 125 µg/mL for ketoconazole, 0.25 µg/mL for itraconazole, 0.125 µg/mL for posaconazole, 0.25 µg/mL for voriconazole, 0.0156 µg/mL for terbinafine, >8 µg/mL for caspofungin and 2 µg/mL for amphotericin.

**Conclusions:** It is known that black yeasts are naturally associated with extreme environments rich in hydrocarbons, such as aviation kerosene. Pathogenic species such as *E. xenobiotica* and other species of the genus *Exophiala* has already been isolated from this type of samples. Our study was the first to isolate *E. phaeomuriformis* in kerosene samples. This black yeast is known to cause superficial mycosis in humans, but also deep infections mainly in the respiratory system. Our isolate was susceptible to five antifungal drugs (itraconazole, posaconazole, voriconazole, terbinafine and amphotericin), however highly resistant to ketoconazole and caspofungin. Considering the interest in using fungal isolates capable of degrading hydrocarbons to minimize environmental impacts, it is very important to select species that present this benefit without bringing risks to human health.

## P103 Inventory of Filamentous Fungi and Yeasts Found in the Water and Sand of the Beach of Pier in Arecibo P.R.

**Lourdes Echevarría**

Pontifical Catholic University of Puerto Rico

In recent years, an increase in filamentous fungal and yeast infections has been observed. The water and sand of the beach were studied, for three weeks beginning in February, and culminating in March 2021. **Objective:** To know the diversity of filamentous fungi and yeasts in the water and sand of the beach, to determine if they are pathogenic to man and to know the mycological quality of the sand. **Methodology:** Samples were taken at three points equidistant from the water and the sand in sterile bags. The culture media were used: SDA, RBA, HardyCHROM and CHROMagar. For water analysis, 100 mL were filtered in triplicate and placed on each plate with the different culture media. For sand analysis, 1 gram was weighed in triplicate and spread on the plates with each culture medium. They were sheltered for 7 to 14 days at 25 °C. Colonies were counted and then isolated. Macroscopic and microscopic analysis was performed. **Results:** The water analysis determined the presence of three genus: *Aspergillus, Rhizopus* and *Penicillium*. Average yeast ranged from 21 CFU on CHROMagar and 12 CFU on HardyCHROM. The yeast species identified were *C. albicans* and *C. tropicalis*. The average of filamentous fungi was 12 CFU in RBA and 15 CFU in SDA. The fungal species identified in the water were *A. ochraceus, A. flavus, A. parasitucus, A. niger, A. versicolor, P. citrinum, P. chrysogenum and R. oligosporus* and *R. stolonifer*. Two genus were identified in the sand: *Aspergillus* and *Penicillium*. The genus *Aspergillus* was the one with the highest identification. Yeast average was: 19 CFU on CHROMagar, 20 CFU on HardyCHROM. The two yeasts identified were: *C. albicans* and *C. tropicalis*. The average of filamentous fungi in each culture medium was 30 CFU in RBA and 38 CFU in SDA. The filamentous fungi species identified in the sand were *A. terreus, A. versicolor, A. niger, A. flavus, A. oryzae, A. fumigatus, P. monoverticillate* and *A. aculeatus*. **Conclusions:** The quality of the sand was classified as average. There is a connection between filamentous fungi and yeasts identified in water and sand with clinical samples. Most of the fungi identified are human pathogens. Causing infections in different parts of your body.

## P104 How Climate Changes Are Shaping Fundamental Niche of *Cryptococcus gattii* Vgi in Europe and Mediterranean Area

**Massimo Cogliati, Maria Carmela Esposto, Anna Prigitano and Luisa Romanò**

Università Degli Studi Di Milano

**Objectives:** In the present study, we analyzed how geographical distribution of the fungal pathogen *Cryptococcus gattii* VGI in Europe and Mediterranean area has evolved in the last four decades based on the climatic changes, and we tried to predict the scenario for the next decade.

**Methods:** Twenty-two occurrence points for *C. gattii* VGI isolates recovered from the environment were obtained by the Screen Project database, whereas temperature and precipitation datasets, from 1980 to 2019, were downloaded from WorldClim website. Species distribution analysis was performed by MaxEnt software.

**Results:** Niche modelling by Maxent analysis showed that recent climate changes have significantly affected the distribution of the fungus revealing a gradual expansion of the fundamental niche from 1980 to 2009 followed by an impressive increase in the last decade (2010–2019) during which the environmental surface suitable for the fungal survival was more than doubled. In the next decade, our model predicted an increase in the area of distribution of *C. gattii* VGI from the coasts of the Mediterranean basin towards the more internal sub-continental areas.

**Conclusions:** On the basis of these predictions, an increase of cases of cryptococcosis due to *C. gattii* VGI is expected in the next decade, therefore, a constant monitoring of the epidemiology of this fungal pathogen represents a crucial strategy to detect the onset of future outbreaks.

## P105 Analysis of the pH-Dependent Secretion of Lipases by *Candida albicans* Isolates

**Rocío Castro ^1^, Asier Ramos ^1^, Elena Sevillano ^1^, Guillermo Quindós ^1^, Elena Eraso ^1^ and Vladimir Kaberdin ^1,2^**

^1^ University Of The Basque Country Upv/ehu
^2^ IKERBASQUE, Basque Foundation for Science

**Objective:** *Candida albicans* is an opportunistic fungal pathogen that can cause superficial or invasive infections. *C. albicans* is able to adapt and thrive under adverse conditions such as suboptimal pH, nutrient scarcity and limited oxygen supply common in some host microenvironments. Its pathogenicity is often associated with the production of virulence factors, among which some hydrolytic enzymes can play a major role in disease development. The aim of this study was to determine the effect of external pH and strain origin on the efficiency of extracellular lipase production.

**Methods:** Twenty-three *Candida albicans* isolates from different sites of infection (oral cavity (6), urine (5), vagina (4), skin (4) and blood (4)) were analysed. To detect lipolytic activity, the strains were grown on malt agar plates supplemented with Tween 80 and calcium chloride. The assays were performed at different pH (5, 6.5 and 7.5) of the medium and the halos formed around the colonies were further examined to calculate the corresponding *E_z_* (enzymatic zone) indexes. Kruskal-Wallis and Dunn’s *post hoc* tests were performed. The data were considered to be statistically significant for *p* < 0.05.

**Results:** The lipolytic activity, initially assessed at pH 5, was observed for all *C. albicans* strains originated from oral cavity and skin lesions, while the occurrence of the lipase-secreting strains among the vaginal (75%), urine (25%) and blood (25%) isolates was considerably lower. At pH 6.5 and 7.5, only oral (66% and 50%, respectively) and skin (66.6% at both pH) isolates manifested lipolytic activities. In general, enzyme production at pH 5 was markedly higher regardless of the origin of the isolates. Furthermore, oral and skin isolates showed higher lipolytic activities at all pH compared to those that were obtained for *C. albicans* originated from other sites of infection. The most notable difference was observed between urine and skin isolates.

**Conclusions:** Production of lipase-like enzymes depends on pH and clinical origin of *C. albicans* isolates. Those from oral cavity and skin show significantly greater lipolytic activity than the rest of isolates and their activity was higher at low pH.

**Funding:** GIC15/78 IT-990-16 (Gobierno Vasco- Eusko Jaurlaritza)

## P106 Effect of pH on Secretion of Proteases by Clinical Isolates of *Candida albicans*

**Asier Ramos ^1^, Rocío Castro ^1^, Elena Eraso ^1^, Guillermo Quindós ^1^, Vladimir Kaberdin ^1,2^ and Elena Sevillano^1^**

^1^ University Of The Basque Country Upv/ehu
^2^ IKERBASQUE, Basque Foundation for Science

**Objectives:** *Candida albicans* is a well-known opportunistic pathogen frequently isolated from different infection sites of the human body (skin, vagina, oral cavity or gastrointestinal tract). The development of candidiasis is often linked to the production of different virulence factors including various hydrolytic enzymes. Since the pH conditions of the infection sites vary from acid (vagina) to the neutral (skin) pH, the aim of this study was to develop alternative agar plate techniques and analyse the effect of pH on the secretion pattern of proteases produced by *C. albicans* isolates at different pH.

**Materials & Methods:** A selection of 24 clinical *C. albicans* isolates were analysed. The strains were previously isolated from different sites of infection including blood, oral cavity, skin, urine, and vagina. In addition, 2 *Candida auris* blood isolates along with *Candida tropicalis* NCPF 3111 and *Candida albicans* NCPF 3153 isolates were tested. The production of proteases at different pHs was tested on solid media containing bacteriological agar, yeast extract, yeast nitrogen base and a protease substrate (i.e., skim milk or Bovin Serum Albumin (BSA)). The pH of the media was adjusted to 5.0, 6.4, and 7.5, thus mimicking the pH range in different sites of infection. The plates were inoculated with 10 µL suspension of *Candida* cells in saline solution McFarland value: 0.7–0.8) and incubated at 37 °C for 5 (milk agar plates) or 7 (BSA agar plates) days. The capacity of the tested isolates to secrete proteases was assessed by the appearance and size of the halos formed around each colony.

**Results:** Plates containing skim milk as a substrate made it possible to detect protease secretion by all 24 *C. albicans* clinical isolates, whereas detection with BSA plates was possible only for one third of the strains. Moreover, protease production was generally more efficient at pH 5.0 compared to pH 6.4 or pH 7.5. In addition, there seems to be a correlation between the pH of the infection sites and the pH at which the cognate *C. albicans* isolates show maximal protease secretion.

**Conclusions:** Milk agar plates offer the best option to study the pH-dependent secretion of proteases by *Candida* spp. Moreover, lower pH seems to promote protease secretion activity, and the strains originated from the infection sites with low pH appears to have a broader substrate specificity.

## P107 Lower Funneling Pathway in Scedosporium Species

**Kévin Ravenel**

University Of Angers

**Objectives:** Lignocellulolytic fungi that are able to degrade lignin have received a particular attention during the last decades. Recent studies evidence that similar enzymatic arsenal is required to degrade lignin, lignocellulose components and organic pollutants like aromatic hydrocarbons. A large number of fungi characterized as opportunistic pathogens are found in human-made environments and exhibit degrading abilities toward aliphatic and aromatic hydrocarbons. Moreover, for several pathogenic microorganisms, a link between capacity to degrade aromatic pollutants and virulence has been established.

Scedosporium are filamentous fungi usually soil saprophytes, that have been regularly reported as causing human infections, particularly in patients with cystic fibrosis. They are worldwide distributed and have been recovered in a wide variety of environments. All studies that have been conducted highlighted their common occurrence in polluted areas. Because of their limited susceptibility to current antifungals, a better understanding of their adaptative mechanisms to these environments is required.

Lignin degradation by microorganisms is initiated by extracellular oxidative steps and followed by intracellular metabolic degradation through the microbial funneling pathway. In fungi, four main aromatic intermediate compounds (protocatechuate, catechol, hydroxyquinol, gentisic acid) serve as substrates in the lower funneling pathway, where dioxygenases are key enzymes catabolizing the ring-opening step. This work was aimed to study these lower funneling pathway in Scedosporium species.

**Materials and Methods:** Orthologues dioxygenases from the literature were used to screen Scedosporium genomic data by tBLASTn (Basic Local Alignment Search Tool) searches. A comprehensive in silico analysis (i.e., alignments and phylogenetic analysis, genome organization) was performed to characterize these enzymes, and their genomic environment. Then, a focus on the gentisic acid pathway was done in order to validate the bioinformatic results. Growth studies and real-time PCR experiments were realized.

**Results:** Sixteen putative genes encoding ring-cleavage dioxygenase were identified on the reference strain S. apiospermum IHEM 14462. The bioinformatic analysis suggests that Scedosporium species are able to catabolize the main aromatic intermediates derived from lignin degradation (i.e., gentisic acid, hydroxyquinol, protocatechuate and catechol). Except for protocatechuate catabolism, the genes of the corresponding pathways are organized in cluster.

Then, the experimental part of the study focuses on the gentisic acid. Results confirm the ability of Scedosporium to grow on synthetic media containing lignin or the gentisic acid as the sole carbon source. Moreover, in these culture conditions, real-time PCR experiments demonstrated that the genes of the cluster were overexpressed.

**Conclusions:** Results obtained in this study confirm that Scedosporium species exhibit the enzymatic arsenal to degrade natural complex molecules and are capable to open aromatic rings. This may explain their presence in polluted environments. Considering the low susceptibility to current antifungal drugs of Scedosporium species, these metabolic pathway may constitute targets for the development of more potent antifungal.

## P120 Demonstration of the Yeasticidal Efficacy of Povidone-Iodine–Based Commercial Antiseptic Solutions against *Candida auris*

**Adélaïde Chesnay ^1^, Eric Bailly ^1^, Victor Evplanov ^2^, Filippo Favalli ^3^ and Guillaume Desoubeaux ^1^**

^1^ CHRU Bretonneau
^2^ Mylan
^3^ Meda Pharma

**Abstract:** Objectives: *Candida auris* is an emerging yeast pathogen with worldwide distribution and a great propensity for nosocomial spread. Recent reports have warned of the significant emergence of *C. auris* in several healthcare facilities. In order to stop its nosocomial transmission, use of antiseptics constitutes the first-line lever of action in the fighting against *C. auris* skin colonization. However, little is known about the efficacy of these products, and moreover no antiseptics are currently registered for use against *C. auris*. Material and methods: This study investigated the in vitro yeasticidal activity of povidone-iodine (Betadine^®^) against *C. auris,* and compared the findings to *C. albicans* and *C. glabrata*. Results: In all the samples, the fungal load was substantially reduced by ≥4.2 Log_10_ colony-forming units, according to the EN standard 1275:2005. Moreover even when largely diluted, povidone-iodine products still allowed sustainable decrease of the yeast viability below 0.1%. Conclusions: Overall, these results support the use of such commercial antiseptics in the context of colonization with this yeast.

**Keywords:** *Candida auris*; povidone-iodine; EN 1275

## P121 New Insights in the Pathogenic Trichosporon Species Intraspecific Diversity: A Comparative Analyses of IGS1 Sequencing and AFLP Fingerprinting

**Elaine Cristina Francisco ^1,2^, Chendo Dieleman ^2^, Eunice Then ^2^, Ferry Hagen ^2,3,4^ and Arnaldo Lopes Colombo ^1^**

^1^ Laboratório Especial de Micologia, Division of Infectious Diseases, Escola Paulista de Medicina, Universidade Federal de São Paulo
^2^ Department of Medical Mycology, Westerdijk Fungal Biodiversity Institute
^3^ Department of Medical Microbiology, University Medical Center Utrecht
^4^ Laboratory of Medical Mycology, Jining No.1 People’s Hospital, Jining

**Objectives:** *Trichosporon* species are opportunistic human fungal pathogens able to cause several clinical manifestations. Over the past few years, the taxonomy has been extensively revised, and high intraspecific diversity was been described among clinical *Trichosporon* isolates. Molecular typing of *Trichosporon* is currently based on solely the IGS1 ribosomal DNA locus, while a large diversity has been observed within *T. asahii* and *T. faecale*, making the validation of these genotypes by robust molecular typing tools crucial to understand the epidemiology. In this study, we used amplified fragment length polymorphisms (AFLP) fingerprinting to assess the degree of intraspecific variability among clinically relevant *Trichosporon* species, comparing results with the IGS1 typing tool.

**Materials & Methods:** A total of 114 *Trichosporon* spp. and three related *Trichosporonales* genera isolates were tested. Clinical isolates (n = 66) were selected from the yeast culture collection at Laboratório Especial de Micologia, São Paulo, Brazil, and reference strains (n = 48) from the CBS yeast collection WI-KNAW. The accurate species identification was performed by sequencing the IGS1 region from the rDNA, and the genotypic characterization of *T. asahii* and *T. faecale* were achieved using reference genotype sequences deposited in the GenBank. The phylogenetic tree was carried out by the Neighbor-Joining method based on the Kimura two-parameter model with 1,000 bootstrap replicates in MEGA7. AFLP fingerprinting was performed using HpyCH4IV and MseI. Selective-PCR was carried out using HpyCH4IV (5′-FLU-GTAGACTGCGTACCCGTC-3′) and MseI (5′-GATGAGTCCTGACTAATGAT-3′), primer combinations. Raw data were analyzed in BioNumerics v7.6, and a dendrogram was created using the UPGMA method.

**Results:** AFLP fingerprinting recognized relevant genetic differences among the 114 isolates tested. *Trichosporon asahii* (n = 24; 9 genotypes), *T. asteroides* (n = 22; 5 genotypes), *T. coremiiforme* (n = 10; 2 genotypes), *T. faecale* (n = 35; 5 genotypes), *T. inkin* (n = 4; 3 genotypes), *T. dohaense* (n = 1), *T. caseorum* (n = 1), *Trichosporon* sp. (n = 1), and the *Trichosporonales* related genera [*Apiotrichum* spp. (n = 4; 4 genotypes); *Cutaneotrichosporon* spp. (n = 10; 7 genotypes); and *Effuseotrichosporon* spp. (n = 2; 1 genotype)], these 39 subgenotypes were distributed over 7 main clusters, indicating high genetic heterogeneity in majority of the species tested than IGS1 sequencing typing.

**Conclusions:** Compared to IGS1 sequencing, AFLP fingerprint provides a higher resolution for *Trichosporon* species typing. Our results indicate a discrepancy between the established IGS-based genotyping vs. higher genotypic diversity when using AFLP fingerprinting. These results might have implications for the taxonomy of *Trichosporon*.

## P122 Codon Optimization and Promoter Selection Facilitates Use of *Mucor circinelloides* for Reporter Assays to Monitore Infection and Antifungal Efficacy

**Ulrike Binder ^1^, Maria Isabella Navarro-Mendoza ^2^, Francisco Esteban Nicolas ^2^, Carmen Kandelbauer ^1^, Rebecca Pföstl ^1^, Ingo Bauer ^3^, Cornelia Lass-Flörl ^1^ and Victoriano Garre**

^1^ Insitute of Hygiene and Medical Microbiology, Medical University Innsbruck
^2^ Fungal genomics and Molecular Biotechnology, University of Murcia
^3^ Insitute of Molecular Biology, Medical University Innsbruck

**Purpose:** Invasive infections caused by mucormycetes are increasingly seen in the clinics and are still associated with unacceptable high mortality rates. In the covid pandemic these infections, mainly the rhinocerebral form, are being reported at alarming frequency in India. Still, little is known about the biology of the pathogens, the establishment and progression of the infection, antifungal resistance mechanisms and successful therapy. Therefore, we aimed to generate tools for (1) alternative methods of drug testing in vitro, (2) non-invasive monitoring of the infection in *Galleria mellonella*, (3) visualization of antifungal efficacy and (4) reporter based gene transcription assays.

**Methods:** Firefly luciferase, both mammalian or codon-optimized without the peroxisomal target sequence was cloned in the pMAT1477 vector under the control of different promoters. Linear plasmid was used to transfect *M. circinelloides* protoplasts of auxotrophic strains. Positive transformants were checked for gene integration by PCR and light emission was measured under various conditions. Selected strains were used to determine antifungal susceptibility, virulence potential and in vivo monitoring of mucormycosis in *Galleria mellonella*.

**Results:** Firefly luciferase was successfully expressed in *M. circinelloides* with a single integration and light emission could be measured by luminometer and visualized. Codon optimization was critical to enhance light emission, making these strains usable for real-time, non-invasive infection monitoring in insect and- in the future-murine models, and the testing of antifungal efficacy by means other than survival. Phenotype, virulence potential in *G. mellonella* and antifungal susceptibility are indifferent to the wild-type strains. Importantly, gene expression was differentially regulated in different media and in vitro vs. in vivo.

**Conclusions:** The optimization of bioluminescent *Mucor* strains allows for the visualization of temporal and spatial progression of infection by a non-invasive method in insect and murine models, and the testing of antifungal efficacy by other means than survival only. Furthermore, this tool can be used to visualize and measure differential expression of genes. Together, our data will give valuable new insights in the pathogenesis of Mucormycete infections.

## P123 *Candida auris* Attributes Mediating Environmental and Host Survival

**Stefanie Allert ^1^, Daniela Schulz ^1^, Philipp Kämmer ^1^, Peter Großmann ^2^, Thomas Wolf ^2^, Sascha Schäuble ^2^, Gianni Panagiotou ^2^, Sascha Brunke ^1^ and Bernhard Hube ^1^**

^1^ MPM, Hans-Knoell-Institute Jena
^2^ SBI, Hans-Knoell-Institute Jena

*Candida* species are a major cause of invasive fungal infections. While *Candida albicans, C. glabrata, C. parapsilosis* and *C. tropicalis* are the clinically most dominant species causing life-threatening candidasis, *C. auris* recently emerged as a new species causing invasive infections with high rates of clinical treatment failures in various regions of the world.

To mimic the initial phase of systemic *Candida* infections with dissemination *via* the bloodstream, we used an ex vivo whole blood infection model. Similar to *C. albicans, C. glabrata, C. parapsilosis,* and *C. tropicalis, C. auris* is efficiently cleared from human blood, showing characteristic patterns of immune cell association, killing rates and induction of cytokines. A minor fraction of infecting *C. auris* is also able to survive human blood for several hours. Dual-species transcriptional profiling of *Candida*-infected blood revealed a rather uniform and conserved response against all five *Candida* spp. by human blood cells, while the fungal transcriptional profile was largely unique for each species, including *C. auris*. Its species-specific responses included adaptation and survival strategies, for example to counteract the defensive oxidative burst of blood immune cells, but also the expression of potential virulence factors, (drug resistance-associated) transporters and cell surface-associated genes.

In contrast to most other *Candida* species, nosocomial *C. auris* cells have been isolated from both patients and environmental niches. Therefore, we also analyzed this fungus under conditions mimicking the host or the hospital environment. We observed a higher stress resistance and long-term environmental survival rates of *C. auris* as compared to the other *Candida* spp. This likely increases the risk of contamination and distribution of *C. auris* in a nosocomial setting. Moreover, experimental in vitro infections of neutrophils with pre-starved *C. auris* cells suggest that environmental preconditioning of this fungus can have modulatory effects on the interaction with the human host.

## P124 Rare Erg6 Modification in an Amphotericin B Resistant *Candida auris* Clinical Isolate from South Africa

**Milena Kordalewska ^1^, Kevin D. Guerrero ^1^, Timothy D. Mikulski ^1^, Tony N. Elias ^1^, Rocio Garcia-Rubio ^1^, Nelesh P. Govender ^2,3^ and David S. Perlin ^1^**

^1^ Center for Discovery and Innovation, Hackensack Meridian Health
^2^ Centre for Healthcare-Associated Infections, Antimicrobial Resistance and Mycoses (CHARM), National Institute for Communicable Diseases, a Division of the National Health Laboratory Service
^3^ School of Pathology, Faculty of Health Sciences, University of the Witwatersrand

**Objectives:** Amphotericin B (AmB) is a polyene antifungal drug with broad spectrum activity against pathogenic fungi. In most *Candida* species, AmB resistance is rare in comparison to resistance to other antifungal drugs (azoles and echinocandins). Of concern, clinical isolates of *Candida auris*, a recently emerged nosocomial pathogen, were reported to have a higher prevalence of AmB resistance (up to 30%). However, underlying mechanisms of resistance were not identified.

The aim of this study was to analyze distribution of AmB minimal inhibitory concentration (MIC) values for *C. auris* isolates belonging to different geographic clades and decipher molecular resistance mechanism in isolates exhibiting elevated MIC values.

**Methods:** A total of 313 *C. auris* isolates representing five geographic clades (I—South Asian; II—East Asian; III—South African; IV—South American; V—Iranian) were investigated in this study. Antifungal susceptibility testing (AFST) with AmB was performed with Etest according to the manufacturer instructions. A tentative AmB MIC breakpoint of ≥2 mg/L, determined by the CDC, was used to categorize isolates as resistant to AmB. Ergosterol biosynthesis pathway genes *ERG2* (C-8 sterol isomerase)*, ERG3* (C-5 sterol desaturase)*, ERG6* (sterol 24-C-methyltransferase), and *ERG11* (lanosterol 14-α-demethylase) were amplified and sequenced. A wild-type *ERG6* gene was replaced in a susceptible strain with a nourseothricin-linked *ERG6* allele identified in an isolate with highly elevated AmB MIC (SA18) by using CRISPR/Cas9 system. AmB susceptibility was then determined for the transformed strain.

**Results:** The results of *C. auris* AFST and gene sequencing are presented in Table 1.

A total of 19 of 313 isolates (6.1%) exhibited AmB MIC values ≥2 mg/L and therefore were categorized as AmB-resistant. In 18 of these 19 isolates no mutations in *ERG2, ERG3, ERG6*, or *ERG11* were found that could explain elevated AmB values. Only one South African isolate (SA18), which had highly elevated AmB MIC (6 mg/L) presented unique non-wild-type *ERG6* genotype. *ERG6* of SA18 is missing base pairs 52–543, resulting in a shorter Erg6 (deletion of amino acids 18–181). Replacement of a wild-type *ERG6* with *ERG6* of SA18 in a susceptible strain (AmB MIC = 0.5 mg/L) induced amphotericin B resistance (AmB MIC >32 mg/L).

**Conclusions:** Mutations in key genes of ergosterol biosynthesis, which can be linked directly to AmB resistance, are extremely rare. Only 1 of 313 clinical isolates screened (0.3%) had an *ERG6* variant which induced AmB resistance in a wild-type strain. Mechanisms other than *ERG6* mutations may also contribute to reduced AmB susceptibility in *C. auris*, although this remains to be determined.

## P125 Terbinafine Resistance Testing of a Multitude of Dermatophyte Genera and Species Using a Simple Breakpoint Method

**Silke Uhrlass, Daniela Koch, Hanna Muetze, Constanze Krueger and Pietro Nenoff**

Laboratory of Medical Microbiology

**Objectives:** Terbinafine-resistant dermatophytes are spreading worldwide. For example, most of the strains of *Trichophyton* (*T.*) *mentagrophytes* ITS (Internal Transcribed Spacer) type VIII from India are in vitro and clinically terbinafine resistant. Today, this species is also found in Germany and Europe. What is new is that *T. rubrum*—isolated from local patients in Germany—may show terbinafine resistance in individual cases.

**Methods & Materials:** A total of 56 wild-type dermatophyte strains, isolated as part of the routine diagnostics of the Mölbis laboratory, were included in the study. The 28 investigated different dermatophyte species belonged to the genera *Trichophyton* (10), *Microsporum* (*M.*) (3), *Epidermophyton (E.)* (1), *Nannizzia (N.)* (7) und *Arthroderma* (*A*.) (7). Out of *Trichophyton* species, *T. rubrum* (2), *T. verrucosum* (1), T. *violaceum* (1), *T. soudanense* (1), *T. erinacei* (1), *T. equinum* (1), *T. benhamiae* (2), *T. quinckeanum* (2)*, T. interdigitale* (4), and *T. mentagrophytes* (20) were examined. The following *Microsporum* species were included in in vitro testing: *M. canis* (5), *M. ferrugineum* (2), and *M. audouinii* (1). The genus *Nannizzia* included *N. gypsea* (1), *N. fulva* (1), *N. persicolor* (1), *N. praecox* (1), *N. incurvata* (1), *N. nana* (1), and *N. perplicata* (1). Out of genus *Arthroderma*, 7 species were tested: *A. tuberculatum* (1), *A. quadrifidum* (1), *A. chiloniense* (1), *A. insingulare* (1), *A. eboreum* (1), *A. ciferrii* (1), and *A. crocatum* (1).

In addition, 9 ITS genotypes were examined within the *T. mentagrophytes*/*T. interdigitale* complex: *T. interdigitale* type II (3), II* (1) and *T. mentagrophytes* type III (3), III* (4), IV (4), VII (4), VIII = *T. indotineae* (2), IX (1), and XXV (2).

In vitro sensitivity testing against terbinafine was carried out using a modified breakpoint method according to Yamada et al., (2017) and Ebert et al., (2020). Minimum inhibitory concentrations (MIC) <0.2 µg/mL are associated with in vitro sensitivity of the isolate, at MIC values ≥0.2 µg/mL, the strain is resistant to terbinafine in vitro.

**Results:** In vitro resistance to terbinafine was found within the *T. mentagrophytes*/*T. interdigitale* complex only for *T. mentagrophytes* ITS type VIII (currently, *T. indotineae*). All other genotypes are in vitro terbinafine sensitive. All other dermatophytes of the 5 genera are predominantly in vitro sensitive to terbinafine. One single strain of *M. canis*, however, had to be classified as in vitro resistant. Few strains were conspicuous with MIC values of 0.1 µg/mL, but not resistant. *A. tuberculatum* was also conspicuous with slightly reduced terbinafine sensitivity.

**Conclusions:** Terbinafine resistance is a new phenomenon and especially a clinical problem in dermatomycology. Currently, almost exclusively the isolates of *T. mentagrophytes* VIII (*T. indotineae*) from India are affected. Other genera, species, and genotypes of dermatophytes—in particular, *T. rubrum* as the most common isolated species in this country—are currently still in vitro sensitive to terbinafine.

## P127 Use of Whole Genome Sequencing to Investigate Outbreaks and Study Population Structure of *Saprochaete clavata* among European Clinical/Environmental Isolates

**Marie Desnos-Ollivier ^1^, Alexis Criscuolo ^2^, Françoise Dromer ^1^ and Geotrichum Investigation Group ^1^**

^1^ Institut Pasteur, CNRS UMR 2000, Molecular Mycology Unit, National Reference Center for invasive Mycoses & Antifungals
^2^ Institut Pasteur, Bioinformatics and Biostatistics HUB—Mutualized Platform for Microbiology

**Objectives:** *Saprochaete clavata* (*Magnusiomyces clavatus)* is considered as a very rare pathogen worldwide. This species is intrinsically resistant to echinocandins and fluconazole. Since 2012 it has been identified as responsible for local and national outbreaks in France. Infections with *S. clavata* usually occur in patients with haematological malignancies and are associated with a very high global mortality rate. We recently showed that defective dishwasher should be considered as a source of contamination. The objective of the present study is to use whole genome data to study population structure of this emerging pathogen.

**Methods:** The whole genome of 189 clinical and environmental isolates recovered mainly in France but also in 9 European countries, between 2001 and 2021 was sequenced (P2M facility, Institut Pasteur), using a NextSeq 500 sequencer, Illumina.

**Results:** Determination of single nucleotide polymorphism (SNPs) positions among the genome of 12 Mb showed a low polymorphism suggesting a recent emergence of this species or a poor genome plasticity. The presence of clades previously identified as responsible for French outbreaks was confirmed, and new clades were also identified. The persistence of the same *S. clavata* clade over 8 years was also demonstrated in one hospital. Furthermore, environmental isolates, mainly recovered from dishwashers, and clinical isolates recovered during local outbreaks were genetically closely related confirming that dishwasher can be considered as the main source of contamination.

**Conclusions:** Our analysis highlights the fact that control measures such as control of the efficiency of dishwashers including correct temperature cycles, rigorous and thorough cleaning of dishes could be implemented in hospitals to limit the spread of *S. clavata*.

## P128 Peptidorhamnomannan of the Cell Wall of *S. brasiliensis* Induces a Strong IL-1-Dependent Th1-Th17 Response

**Brenda Kischkel ^1,2^, Jéssica C. dos Santos ^1^, Carlos P. Taborda ^1^, Leila M. Lopes-Bezerra ^1^, Leo A. B. Joosten ^2^ and Mihai G. Netea ^2^**

^1^ Department of Microbiology, University of São Paulo
^2^ Department of Internal Medicine, Radboud University Medical Center

**Objectives:** Peptidorhamnomannan (PRM) is a component of the cell wall of *Sporothrix* spp. that is involved in the recognition of the fungus by human macrophages. Previous studies have shown that there are structural differences between the PRMs of *S. brasiliensis* (*S.b* PRM) and *S. schenckii* (*S.s* PRM). We hypothesize that these structural differences may be associated with the different strengths of pathogenicity observed among these species. Therefore, in this study we evaluated the mechanisms involved in the induction of cytokines by *S.b* and *S.s* PRMs.

**Materials & Methods:** Human peripheral blood mononuclear cells (PBMCs) were stimulated with *S.b* PRM, *S.s* PRM as well as heat-killed yeasts of *S. brasiliensis, S. schenckii* and *Candida albicans* as control stimulus. The profile of pro- and anti-inflammatory cytokine production was evaluated by ELISA. In addition, the cytokine production was assessed in the presence of specific inhibitors of pattern recognition receptors (PRRs), cytokines and their associated downstream and metabolic pathways.

**Results:** Exposure of PBMCs to *S.b* PRM resulted in the production of pro-inflammatory cytokines associated with innate immunity TNF-α, IL-6 and IL-1β at levels comparable to *C. albicans*, as well as induction of T-helper cytokines IFN-γ, IL-17 and IL-22. Surprisingly, *S.s* PRM induced the production of higher concentrations of interleukin-1 receptor agonist (IL-1Ra) than *S.b* PRM which in turn resulted in low production of T-helper cytokines, mainly IL-17 and IL-22. Stimulation of PBMCs with the whole organisms showed the same pattern of associated innate immunity cytokines induced by their respective PRMs, but at higher concentrations. Interestingly, *S.b* PRM induced up to 10 times more T-helper cytokines IFN-γ, IL-17 and IL-22 than *S. brasiliensis*, while *S.s* PRM induced levels comparable to *S. schenckii*. The induction of TNF-α, IL-6 and IL-1β by *S.b* PRM was associated with TLR4 and CR3 receptors. For *S.s* PRM, dectin-1 and CR3 was associated with the production of IL-1β and TNF-α, respectively. The blockade of the IL-1 receptor with rhIL-1Ra (anakinra) considerably impaired the induction of pro-inflammatory TNF-α, IL-6 and IL-1β and T-helper IFN-γ, IL-17 and IL-22 cytokines by *S.b* PRM, whereas for *S.s* PRM only the production of IFN-γ was not decreased in the presence of rhIL-1Ra. The glutaminolysis pathway was involved in the production of TNF-α, IL-6 and IL-17 by *S.b* PRM and *S. brasiliensis*, and TNF-α, IL-1β, IL-17 and IL-22 by *S. schenckii*. The addition of a glycolysis inhibitor, impaired the production of all cytokines evaluated by *S.b* PRM, *S. brasiliensis* and *S. schenckii*. Regarding *S.s* PRM, the production of IL-1Ra, IFN-γ, IL-17 and IL-22 decreased significantly.

**Conclusions:** Our results demonstrate that immune response induced by the *S.b* or *S.s* PRM are distinct. *S.s* PRM is marked by high concentrations of IL-1Ra. In in vivo models of aspergillosis this cytokine can increase the host’s susceptibility to fungal infection. *S.b* PRM induces a potent Th17 inflammatory response in a IL-1 dependent manner. This findings opens novel treatment strategies that aim to control the local inflammation process induced by *S. brasiliensis* infection through IL-1 inhibition.

## P129 Emergence of Unusual and Multidrug-Resistant Candida Species in a Tertiary Care Set-Up

**Priyanka Jangra, Malini Capoor, D K Gupta, B K Tripathi and HC Sachdeva**

Vmmc and Safdarjung Hospital

**Objectives**: The aim of this study was to determine the species distribution and antifungal susceptibility pattern of candidemia cases in adult patients at a tertiary care hospital.

**Materials and methods:** *Candida* species identification was performed by phenotypic methods, Vitek (Biomerieux, France) and DNA sequencing. The antifungal susceptibility was performed by broth microdilution method as per CLSI M27-A4 guidelines 2017.

**Results:** Out of 1274 blood samples, 70 samples (5.5%) yielded the growth of *Candida* species. There was predominance of NAC spp. over *C. albicans* in candidemia patients. *C. tropicalis* (28.57%, 20/70) was the predominant *Candida* species followed by *C. parapsilosis* (22.85%, 16/70), *C. glabrata* (14.28%, 10/70), *C. auris* (12.85%, 9/70). Rare species among NAC spp. included *C. auris, C. mesorugosa, C. lusitaniae, P. kudriavzevii* and *C. haemulonii* were isolated. The most common predisposing factor for candidemia was urinary catheter (72.85%, 51/70) followed by increased period of hospitalization (42.85%, 30/70), diabetes mellitus (21.5%, 15/70), etc. The significantly associated risk factor associated with *C. auris* was diabetes mellitus (*p* = 0.02). The overall resistance was 22.57% to all antifungal drugs. The multidrug resistance (MDR) was noted in 5.71% of isolates.

**Conclusions:** Early identification of risk factors, *Candida* speciation and timely management are crucial for the outcome of candidemia cases. Non- *albicans* species was predominant over *C. albicans* depicting the change in the epidemiology and emergence of MDR *Candida* spp. like *C. auris, C. glabrata, C. mesorugosa, C. lusitaniae, Pichia kudriavzevii (C. kruseii)*. This warrants routine antifungal susceptibility testing (AFST) and close monitoring. The knowledge of local epidemiological profiles of *Candida* spp., accurate species identification and their antifungal susceptibility are crucial for overall patient management.

## P130 First Detection of TR46/Y121F/T289A Cyp51 Mutation in *Aspergillus fumigatus* Isolate in the Russian Federation

**Galina Klyasova, Anna Malchikova and Svetlana Khrulnova**

National Recearch Center For Hematology

**Objectives:** Invasive aspergillosis remains a major problem in immunocompromised individuals. Resistance of *Aspergillus fumigatus* to one or more triazoles has been reported for the last years. The aim of the study was to evaluate the antifungal susceptibility for clinical *A. fumigatus* isolates.

**Materials & Methods:** Retrospective evaluation of antifungal susceptibility for *A. fumigatus* isolates from bronchoalveolar lavage (BAL) fluid in pts with invasive pulmonary aspergillosis was performed. All isolates were obtained from hematological pts admitted to National Research Center for Hematology (Moscow) between 2016 and 2020. All *A. fumigatus* species were identified by morphology, matrix-assisted laser desorption ionization time of flight mass spectrometry. The azole-resistant strains were identified by ITS, β-tubulin, calmodulin and actine gene sequencing. Antifungal susceptibility for *A. fumigatus* to voriconazole, posaconazole, itraconazole, amphotericin B, caspofungin, anidulafungin was carried out by Sensititre YeastOne Y010 AST Plate (Thermo Scientific, Waltham, MA, USA). The MIC of antifungal agents were read according to the CLSI (2020). Cyp51A was sequenced to establish the most common resistance mechanisms embedded in azole-resistant *A. fumigatus*.

**Results:** A total of 35 *A. fumigatus* were tested, of them 2 (5.7%) isolates had elevated minimum inhibitory concentrations (MIC) to voriconazole >8 μg/mL. The azole-resistant strains were identified as *A. fumigatus sensu stricto* by ITS, β-tubulin, calmodulin and actine gene sequencing. The first voriconazole-resistant *A. fumigatus* was isolated in 2017, the second—in 2020. Both voriconazole-resistant *A. fumigatus* were susceptible to other anifungal agents. Analyzes of *Cyp51A* gene of the two confirmed voriconazole-resistant *A. fumigatus* species revealed TR46/Y121F/T289A mutation in one isolate. Second isolate was positive for I242V mutation.

Characteristics of pts with invasive pulmonary aspergillosis caused by voriconazole-resistant *A. fumigatus* are presented in Table 1. Both pts under went allogeneic hematopoietic stem cell transplantation. Previous voriconazole regarding was used in one patient.

**Conclusions:** This is first report from Russia regarding detection of voriconazole-resistant *A. fumigatus* caused by TR_46_/Y121F/T289A mutation which is predominated in the world. The other potential resistance mechanism was caused by I242V mutation of the Cyp51A gene.

## P131 First Case Report of Bloodstream Infection by *Candida blankii*, in a Neonate from Pakistan

**Summiya Nizamuddin**

Shaukat Khanum Memorial Cancer Hospital and Research Center

**Objective:** Invasive candidiasis is a cause of high morbidity and mortality in neonates. Recently, an outbreak of candidemia was reported due to the rare multidrug-resistant yeast *Candida blankii* in an Indian neonatal unit. We report the first case report of a blood stream infection caused by *Candida blankii* in a neonate, from Pakistan.

**Materials and methods:** Blood cultures grew *C. blankii* in a full term neonate, who was admitted in the Neonatal Intensive Care Unit of a small peripheral hospital in Punjab, Pakistan. Blood culture sample was received at the laboratory of the Shaukat Khanum Memorial Cancer Hospital and Research Centre. *C. blankii* was isolated on culture. Identification was confirmed on the Vitek MS. Antifungal susceptibility testing was performed by the Vitek 2 fungal susceptibility card.

**Results:** *C. blankii* candidemia was confirmed via the MALDI-TOF and was informed immediately. The child was admitted in the neonatal unit following sepsis. The *C. blankii* strain was found to be resistant to fluconazole. The child was hence, treated with amphotericin. The neonatal unit team was duly informed to implement all infection control measures and be vigilant for any other neonates developing candidemia. However, no other cases of candidemia were seen.

**Conclusions:** The case report highlights of sporadic isolation of a rare and uncommon yeast, *C. blankii*, with reduced susceptibility to antifungal agents in nosocomial fungaemia. Genomic analysis of this strain would be required to analyze relatedness to the Indian strains.

## P132 Preliminary Data on *Aspergillus flavus* Epidemiology in France

**Lise Bertin ^1^, Stéphane Bretagne ^2^, Karine Sitbon ^2^, Jean-Pierre Gangneux ^3^ and Fanny Lanternier ^1,2^**

^1^ Necker Hospital, Aphp
^2^ Institut Pasteur
^3^ University and Hospital Center of Rennes

**Objectives:** Invasive aspergillosis is mainly known through *Aspergillus fumigatus*, which essentially causes lung damage. Other aspergillus species are also a source of various infections in humans. Among those, *Aspergillus flavus* is the main pathogen of Ear-Nose-Throat (ENT) localizations (sinus, ophthalmologic, auricular). It can also be the cause of cutaneous or lung infections.

Due to its lower frequency, few clinical cases and meta-analyzes are reported and this pathogen remains poorly understood.

**Materials & Methods:** We studied risk factors, localizations and prognosis of *A. flavus* infections. For this purpose, we studied here 54 cases recorded since 2012 in its database by the French National Reference Center for Invasive Mycoses and Antifungals (NRCMA) as part of its surveillance assignments (RESeau de Surveillance des Infections Fongiques invasives en France, RESSIF).

**Results:** Our preliminary results found two major risk factors: blood malignancies (29/54, 53%) mainly acute leukemia under corticosteroid or immunosuppressive therapy and type 2 diabetes (14/54, 26%). We also observed 9 cases of various solid organ transplant (kidney, heart or lung) under immunosuppressive therapy (2/9 with type 2 diabetes associated), 2 patients suffering from major burns, 1 case with type 1 diabetes and 1 case of drugs induced neutropenia.

In agreement with previous studies, *A. flavus* predominantly affects the lungs (72% of cases) or/and ENT system (34% of cases) with frequent extension to the skull base.

The lung was the main localization in patients suffering from blood disorders (25/29, 86%), in contrast to patients with diabetes for whom it was mostly the ENT system (12/14, 85.7%).

The global mortality was high, 53% mortality at 3 months, higher than that recorded for *A. fumigatus* infections. The most severe forms include disseminated disease and lung localization.

**Conclusions:** These preliminary results uncover two main patterns for *A. flavus* infections in France, highly dependent of the underlying conditions.

## P133 Investigating *Candida auris* in West Africa

**Oluwademilade Agbalaya ^1^, Gabriel Adeleke ^1^, Abdul-Wahab Ettu ^2^ and Rita Oladele ^1^**

^1^ Department of Medical Microbiology and Parasitology, College Of Medicine, University Of Lagos
^2^ Marigold Hospital, Surulere

**Objectives:** *Candida auris* is an emerging pan resistant pathogen globally, and has been reported to cause outbreaks in a number of ICUs in Japan, India, USA, South Africa. It is of particular importance in resource limited settings such as ours where there is poor availability and accessibility of drugs needed to treat its infections. *Candida auris* was identified in 4 blood culture samples from 4 different hospitals in Nigeria using Vitek® 2 Compact. Three of the patients were in the ICU and one was a critically ill patient in a Gyneacological ward. The objective of this work was to investigate the potential reservoirs of the organism in the patients’ environment.

**Materials & Methods:** Two hospitals gave consent for environmental investigation which was carried out using the environmental surveillance toolkit adapted from CDC’s website. Swabs were collected from relevant environmental sources according to the toolkit’s guidance. The collected samples were then cultured on Saboraud Dextrose Agar slants at 37 °C for 24 to 72 h. Identification of yeast isolates was carried out using the Biomerieux Vitek® 2 Compact, and data analysis was done using Microsoft Excel.

**Results:** A total of 141 swab samples were collected from both sites, 60(42.5%) of the samples yielded growth. There were 9 yeast isolates: *Candida rugosa* 3(33.3%) from a bedside locker, mattress and a pulse monitor; *Cryptococcus laurentii* 2(22.2%) from an axilla and groin composite skin swab and a sink. *Candida albicans* 1(11.1%) was from a bed railing, *Candida lusitaniae* 1(11.1%) was from a bedside locker, *Candida parapsilosis* 1(11.1%) and *Candida tropicalis* 1(11.1%) was from drug carts. There were 20 mold isolates from bed railings (14;70%), drug carts (3;15%), bedside lockers (1;5%), matress (1;5%) and a sphygmomanometer (1;5%). There were 31(22%) bacteria isolates. No *Candida auris* was isolated in this study. Three patients died and one has been discharged wihout having had any antifungal therapy.

**Conclusions:** *Candida auris* was not isolated from the patients’ environment and this is not surprising, we lacked the resources to do this, like the environmental sponge sticks, circulating stomacher and the *Candida auris* Chromagar. Interstingly, quite a significant number of molds were isolated from bed rails, but not surprsing because we have a tropical climate and the ward windows are open down. It is imperative that in view of possible outbreaks of *Candida auris*, these resources be made available in our setting. Educating clinicians is also critical to curtail possible outbreaks.

## P134 Invasive Osteoarticular Infections from the French Scedosporiosis/Lomentosporosis Observational Study (SOS): No Mortality with Long Term Antifungal Treatment and Surgery

**Damien Blez ^1^, Didier Bronnimann ^2^, Dea Garcia-Hermoso ^3^, Fanny Lanternier ^1,3^ and French Mycoses Study Group**

^1^ Assistance Publique-Hôpitaux de Paris (APHP), Hôpital Necker-Enfants Malades, Université de Paris
^2^ Hôpital Saint André
^3^ Unité de Mycologie Moléculaire, CNRS, French National Reference Center for Invasive Mycoses & Antifungals, UMR 2000, Institut Pasteur

**Objectives:** Little is known about localized osteoarticular Scedosporiosis (LOS). Most of the data come from case reports and small case series. Here we present an ancillary study of the nationwide French Scedosporiosis Observational Study (SOS), describing 18 well documented consecutive cases of LOS between January 2005 and December 2015.

**Materials & Methods:** All adult patients diagnosed with LOS defined by osteoarticular involvement without distant foci reported in SOS were included. All cases were carefully reviewed to exclude eumycetomas and malignant otitis.

**Results:** 17 proven and 1 probable LOS were analyzed. All patients had trauma or an underlying disease as predisposing condition. Ten out of 18 patients had an underlying disease (2 had only diabetes while the others were immunocompromised). Fourteen out of 18 patients had prior related trauma that could account as potential inoculation. The anatomical sites involved were joints (44%), long bones (28%), spine (17%) and thoracic wall (11%). The most common clinical manifestation was pain (87%), followed by abscess (44%), swelling joint or limb (39%), cutaneous fistulization (39%) and fever (33%). Involved species were *S. apiospermum* (n = 10), *S. boydii* (n = 4), *L. prolificans* (n = 3) and *S. dehoogi* (n = 1). The species distribution was unremarkable except for the *S. boydii* bones and joints infections (BJI) that were associated with healthcare inoculations. The management was based on medical and surgical treatment for 72% of the patients. One patient did not receive antifungals and four patients did not undergo surgery. The median duration of antifungals was 7.6 months. Patients received voriconazole in 94% of cases. All patients were eventually cured or stabilized on antifungal therapy. Three patients (17%) required suppressive therapy, one patient underwent limb amputation. Compared to other invasive Scedosporiosis (n = 73) in the SOS database, localized osteoarticular disease was associated with less haematologic malignancies (0% vs. 46%), more traumatic inoculations (78% vs. 8%), required more often surgical management (78% vs. 41%) and had lower 3-months mortality (0% vs. 32%) (*p* < 0.05).

**Conclusions:** Despite an overall good clinical outcome compared to other invasive Scedosporiosis, LOS was associated with a heavy morbidity due to extended courses of antifungals, multiple surgeries and repeated hospitalizations. This underscores the importance of raising awareness of this disease and the need to improve the knowledge about its optimal management.

## P135 Kazachstania Slooffiae, an Emerging Pathogen to Watch out for?

**Ana Cristina Gallotti ^1^, Maria Soledad Cuétara ^1^, Maria del Mar Lombera Garcia-Corona ^2^, Ignacio Pinilla ^3^, Alberto Nieto ^4^, Jorge Ligero Lopez ^1^ and Oscar Zaragoza ^5^**

^1^ Microbiology department. Severo Ochoa University Hospital
^2^ Digestive unit. Severo Ochoa University Hospital
^3^ Department of Pathology. Severo Ochoa University Hospital
^4^ Microbiology department. Badajoz University Hospital
^5^ Mycology Reference Laboratory of National Centre for Microbiology

**Introduction:** Kazachstania slooffiae formerly known as Candida slooffiae is a member of the Kazachstania telluris species complex, which includes Kazachstania bovina, Kazachstania pintolopesii, Kazachstania slooffiae, Kazachstania heterogenica y Kazachstania telluris. It has been isolated from horse and porcine gut. Fatal cases in animals have been described but little is known about its role as pathogen in humans.

We report a case of an elderly man who is followed by the digestive unit due to esophageal dilation, a gastroscopy was performed obtaining samples for microbiology and anatomopathology, and *K. slooffiae* was isolated.

**Case report:** 80-year-old male patient, with a previous history of prostate cancer, consulted for progressive dysphagia and was initially evaluated with a barium gastroduodenal study that showed a significantly dilated oesophagus. An upper endoscopy was requested. The mucosa was covered with a whitish irregular layer, apparently thick. Biopsies of the oesophageal mucosa demonstrated a paved non-keratinizing mucosa with a polymorphonuclear inflammatory infiltrate and the PAS stain showed fungal structures.

The biopsy sample was seeded onto Sabouraud dextrose agar and incubated at 35 °C. Colonies were shiny, cream-colored with lobate margins. The identification was performed with the MALDI-TOF MS analysis of the MALDI Biotyper instrument equipped with MALDI Biotyper software version 4.1.14, obtaining *Kazachstania slooffiae* score of 1.71. Fungal identification was confirmed in the Mycology Reference Laboratory of the National Centre for Microbiology by sequencing the internal transcribed sequence from the ribosomal DNA. ITS region was amplified by PCR using ITS1 (5′TCCGTAGGTGAACCTGCGG3′) and ITS4 (5′TCCTCCGCTTATTGATATGC3′) oligonucleotides. Finally, the PCR products were sequenced using ITS1 and ITS4 oligonucleotides using the Sanger protocol. Correct identification was confirmed using the database of the ITS sequences of the Mycology Reference Laboratory of the National Centre for Microbiology using Infoquest software (version 4.5, BioRad).

Antifungal susceptibility testing was performed using Sensititre® YTAMYUCC panel (Thermofisher® Scientific). This showed MICs (πg/mL) to amphotericin B 0.25, fluconazole 0.25, posaconazole 0.015, voriconazole 0.008, itraconazole 0.015, isavuconazole 0.008, anidulafungin 0.06, micafungin 0.03 and caspofungin 0.125.

The patient underwent a 10-days fluconazole treatment, after which he described an important clinical improvement and no longer complaint for dysphagia.

An endoscopy was repeated after a month of the end of the treatment and biopsies were taken. No more fungal structures were seen and microbiological cultures were negative.

**Discussion:** *K. slooffiae* is an uncommon yeast. As far as we know, there has been only one previous case in humans. In our patient, being the only pathogen seen and cultured in the oesophageal biopsy, supported the empirical treatment with fluconazole. Based on the good clinical-histological-mycological response we believe that this yeast was responsible for the clinical picture of our patient.

**Conclusions:** This case reflects that there are no uniformly non-pathogenic fungi: any fungus can cause infection in a sufficiently immunocompromised host and should never be dismissed out of hand as a contaminant.

These new species might lead to issues with identification and antifungal susceptibility reason why clinicians should be aware of infections with uncommon yeast species and consider confirmation through DNA sequencing.

## P136 Screening of Agriculture Fungicides to Inhibit the Growth of Emerging Fungal-like *Pythium insidiosum*

**Hanna Yolanda ^1,4^, Tassanee Lohnoo ^2^, Thidarat Rujirawat ^2^, Wanta Yingyong ^2^ and Theerapong Krajaejun ^3^**

^1^ Section for Translational Medicine, Faculty of Medicine, Ramathibodi Hospital, Mahidol University
^2^ Research Center, Faculty of Medicine, Ramathibodi Hospital, Mahidol University
^3^ Department of Pathology, Faculty of Medicine, Ramathibodi Hospital, Mahidol University
^4^ Department of Parasitology, School of Medicine and Health Sciences, Atma Jaya Catholic University of Indonesia

**Objectives:** *Pythium insidiosum* is a fungal-like microorganism, belongs to the class Oomycete. This organism causes the life-threatening disease, called pythiosis, in humans and animals, usually manifested as arteritis, keratitis, and granulomatous cutaneous ulcers. The disease has been increasingly reported worldwide. *P. insidiosum* morphologically resembles filamentous fungi that are frequently misdiagnosed as fungal infection in a clinical setting. The antifungals drugs are less effective against *P. insidiosum* due to the lack of drug-target ergosterol biosynthesis enzymes. An effective anti-*P. insidiosum* drug is needed for the treatment of pythiosis. Currently, a handful of agriculture fungicides show inhibitory effects against a broad range of plant-pathogenic oomycete organisms. This study aims to screen such fungicides for a non-toxic chemical that can inhibit the growth of *P. insidiosum*.

**Materials & Methods:** Screening of 24 agriculture fungicides that exhibit inhibitory activity against plant-pathogenic oomycete through the PubChem database revealed 6 chemicals (i.e., mandipropamid, fluopicolide, cyazofamid, fenamidone, oxathiapiprolin, and dimethomorph) lack adverse characteristics, such as irritation, corrosion, acute toxicity, and health hazard. These agriculture fungicides were initially evaluated for inhibitory effect against 3 phylogenetical distinct strains of *P. insidiosum* (i.e., strains CBS101555, P38, and ATCC90586) isolated from humans and animal with pythiosis in Thailand, Brazil, and United States. A radial growth assay was employed for the in vitro susceptibility analysis. Each chemical was tested against *P. insidiosum* in 3 biological replicates, using the concentration range of 0.25–128 μg/mL. The percentage of the colony’s radial growth of the treated organism was calculated in relation to the untreated control. The microscopic changes of the microorganisms were recorded.

**Results:** Fluopicolide, cyazofamid, and fenamidone showed solid inhibitory activity that reduced the *P. insidiosum* growths by at least 70% compared to the control (Figure 1). Their MICs were 8, 16, and 64 μg/mL, respectively. The microscopic examination of *P. insidiosum* exposed to 0.5 μg/mL of fluopicolide or cyazofamid demonstrated ballooning hyphal tips and frequently branching spiral hyphae, respectively **(Figure 2)**. Mandipropamid, oxathiapiprolin, and dimethomorph showed no significant inhibition against *P. insidiosum*.

**Conclusions:** Fluopicolide, cyazofamid, and fenamidone exhibited good inhibitory activity against *P. insidiosum*. Further studies are needed to confirm the anti-*P. insidiosum* effect, investigate the safety, and explore their antimicrobial mechanism.

## P137 Respective Roles of the Transcription Factors Tac1 and Mrr1 in *Candida auris* Azole Resistance

**Jizhou Li ^1,2^, Alix T Coste ^1^, Daniel Bachmann ^1^, Dominique Sanglard ^1^ and Frederic Lamoth ^1,2^**

^1^ Microbiology Institute, University Hospital Lausanne
^2^ Infectious Diseases Service, Department of Medicine, Lausanne University Hospital and University of Lausanne

**Objectives:** *Candida auris* is an emerging yeast pathogen, which has attracted worldwide attention because of its ability to develop multidrug resistance and to cause nosocomial outbreaks. Remarkably, development of fluconazole resistance is a hallmark of *C. auris*. Several mutations in the azole target gene *ERG11* were found to play a role in *C. auris* azole resistance. Moreover, overexpression of the drug transporters Cdr1 and Mdr1, resulting from gain-of-function mutations in the transcription factors Tac1 (Tac1a and Tac1b) and Mrr1, may contribute to azole resistance. The objective of the study was to investigate the respective and combined roles of Tac1 and Mrr1 in *C. auris* azole resistance.

**Methods:** Single and double deletions of *TAC1* and *MRR1* were performed by CRISPR-Cas9 in one *C. auris* isolate from clade III (III.8). Antifungal susceptibility testing was performed for assessment of the minimal inhibitory concentrations (MIC) of fluconazole (FLC) and voriconazole (VOR) by standard microbroth dilution method. *CDR1* and *MDR1* expression was measured by quantitative reverse transcription PCR (RT-qPCR).

**Results:** Double deletion of *TAC1a* and *TAC1b* in the *C. auris* III.8 isolate (III.8 *tac1a*/*tac1b* strain) resulted in a 2-fold MIC decrease for FLC (512 to 256 g/mL) and VOR (2 to 1 g/mL). Deletion of *MRR1* (III.8 *mrr1* strain) led to the same effect with a 2-fold MIC decrease for both FLC and VOR. Simultaneous deletion of *TAC1a/b* and *MRR1* (III.8 *tac1a*/*tac1b*/*mrr1* strain) resulted in a 4-fold MIC for both FLC and VOR compared to the parental III.8 strain (FLC: 512 to 128 g/mL; VOR: 2 to 0.5 g/mL). RT-qPCR showed that *TAC1a/b* deletion resulted in a 1.36-fold increase of *CDR1* expression (*p* = 0.025) without significant change of *MDR1* expression, while *MRR1* deletion resulted in a 13.44-fold decrease of *MDR1* expression (*p* = 0.0002) without impact on *CDR1* expression (Figure 1). Both individual effects (modest *CDR1* overexpression and important *MDR1* repression) were observed in the III.8 *tac1a*/*tac1b*/*mrr1* strain.

**Conclusions:** This study shows the respective and combined roles of the transcription factors Tac1 and Mrr1 in *C. auris* azole resistance. Notably, double *TAC1* and *MRR1* deletion resulted in a significant and higher decrease of azole resistance compared to the single deletions.

## P139 COVID-19 Associated Disseminated Mucormycosis in a Young Obese Non-Diabetic Asian Male

**Vidya Krishna ^1^, Jaymin Morjaria ^2^, Rona Jalandari ^4^, Fatima Omar ^4^ and Sundeep Kaul ^2,3^**

^1^ Department of Infectious Diseases, Immunology and BMT, Great Ormond Street Hospital
^2^ Department of Respiratory Medicine, Royal Brompton and Harefield Hospital, Guy’s and St. Thomas Hospital NHS Foundation Trust
^3^ Department of Intensive Care, Royal Brompton and Harefield Hospital, Guy’s and St. Thomas Hospital NHS Foundation Trust
^4^ Department of Cardiology, Royal Brompton and Harefield Hospital, Guy’s and St. Thomas Hospital NHS Foundation Trust

**Objectives:** COVID associated mucormycosis(CAM) has gained attention after the recent outbreak in India. While rhino-orbital cerebral is the commonest presentation reported, especially in diabetics, we present a case of disseminated mucormycosis in a non-diabetic young obese Asian male. There was no ante-mortem clue to mucormycosis in spite of extensive workup. We wish to highlight the importance of this unique presentation of disseminated mucormycosis as a thrombo-embolic disease and systemic sepsis which can be difficult to differentiate from severe progressive COVID-19 disease. To the best of our knowledge, this is the first documented case of disseminated mucormycosis in COVID-19.

**Materials & Methods:** A 22-year-old obese (BMI-44 kg/m^2^) Asian descent male, with known hypothyroidism, was hospitalised in April 2020 with a right anterior cerebral artery (ACA) stroke and severe COVID-19 pneumonitis. He had no prior history of diabetes mellitus and his average blood glucose during his ICU stay was 8.4 mmol/L. He had multi-organ dysfunction involving lungs, kidneys, liver, brain and pancreas requiring mechanical ventilation and renal replacement therapy (CVVHDF). He subsequently developed pulmonary thrombosis, haemorrhagic transformation of his cerebral infarct, and pericarditis as identified by appropriate imaging modalities.

He had recurrent episodes of vasoplegic shock with persistent lymphopenia, raised inflammatory markers (CRP >300 mg/Land Pro-calcitonin >100 ng/mL), D-dimers and troponin levels. Infective workup including a broncho-alveolar lavage and fungal biomarkers [serum 1,3-β-D-glucan (BDG) and galactomannan (GM)] were negative.

He received hydroxychloroquine, azithromycin, meropenem, teicoplanin, tigecycline, caspofungin and steroids along with systemic anti-coagulation and anti-platelet therapy. Despite broad spectrum anti-microbial therapy, he succumbed to the disease 20 days after the illness onset. Autopsy findings revealed disseminated mucormycosis in his lungs, pericardium, hilar nodes and cerebral capillaries in the infarct zone showed fungal thrombi with vasculitis and cerebral invasion. Severe COVID 19 pneumonia, haemorrhagic pancreatitis and steato-hepatitis were also reported.

**Results:** Our patient’s clinical and laboratory parameters demonstrated persistent sepsis with multi-organ dysfunction. He had negative bacterial and fungal workup with no response to empirical broad-spectrum anti-microbial therapy. His clinical course was attributed to progressively severe COVID-19 disease until the autopsy revealed associated disseminated mucormycosis. He did not have any radiology or microbiology indicative of mucormycosis, and thrombo-embolic manifestations were thought to be consistent with severe SARS-CoV-2 infection.

We hypothesize that the primary site of mucormycosis infection in him was the lung, as has been often reported in the literature, with local migration to the mediastinum and haematogenous spread to the brain.

**Conclusions:**

1. In critically ill COVID-19 patients in septic shock unresponsive to conventional anti-microbial therapy with features of immune dysregulation and thrombosis, there should be a high index of suspicion for invasive mycoses including mucormycosis especially when BDG and GM are negative. Empirical amphotericin may be a therapeutic option to consider.
2. There is a need for novel and efficient biomarkers and diagnostic assays to help in early diagnosis of mucormycosis.
3. Conducting more post mortem studies in severe COVID patients will help identify the exact spectrum of CAM.

## P140 Delineation of the Direct Contribution of Mutations in *Candida auris* ERG11 and TAC1B to Triazole Resistance alone and in Combination

**Jeffrey M. Rybak, Katherine S. Barker and P. David Rogers**

St. Jude Children’s Research Hospital

**Objectives:** *Candida auris* is an emerging fungal pathogen of great clinical concern. Greater than 90% of all *C. auris* isolates exhibit high-level fluconazole resistance with MIC typically as high as or even exceeding 256 mg/L. While it has previously been shown that mutations in both *ERG11* and *TAC1B* are common among fluconazole-resistant clinical isolates of *C. auris*, the extent to which these mutations, alone or in combination, impact MIC to each of the triazole antifungals remains unknown. The objective of these studies is to determine the direct contribution of mutations in *C. auris ERG11* and *TAC1B* to triazole resistance alone and in combination.

**Materials & Methods:** Two clinical isolates of *C. auris* exhibiting multi-antifungal resistance and harboring mutations in both *ERG11* and *TAC1B*, one from Clade 1b (Isolate B) and one from Clade 1c (Isolate C), were used in these studies. Each of these isolates exhibit combinations of fluconazole-resistance associated genotypes previously reported among multiple clinical isolates from different countries of origin (Isolate B: *ERG11*^Y132F^ and *TAC1B*^A583S^, Isolate C: *ERG11*^K143R^ and *TAC1B*^A640V^). A Cas9-mediated genetic manipulation system was used to replace mutations of interest in *C. auris ERG11* and *TAC1B* with the wildtype sequence (matching the B8411 clade I reference). MIC for triazole antifungals were determined by broth microdilution.

**Results:** In Isolate B, introduction of the *ERG11*^WT^ genotype resulted in a 32-fold reduction in fluconazole MIC (128 vs. 4 mg/L), while introduction of the *TAC1B*^WT^ genotype decreased fluconazole MIC by 4-fold (128 vs. 32 mg/L). Changes in voriconazole MIC were similar to those of fluconazole, while isavuconazole, itraconazole, and posaconazole MIC remained largely unchanged. In Isolate C, introduction of the *ERG11*^WT^ sequence in place of the mutation encoding K143R lowered fluconazole MIC by 8-fold. MIC for voriconazole, isavuconazole, and posaconazole were reduced 2-fold, while itraconazole MIC remained unchanged. By contrast, introduction of the *TAC1B*^WT^ sequence in place of the mutation encoding A640V alone was sufficient to lower MIC for all 5 clinically available triazoles by 8 to 32-fold. Introduction of the wildtype sequence for both *ERG11* and *TAC1B* in Isolate C resulted in a 128-fold reduction in fluconazole MIC, while MIC of the other triazoles were similar to those observed upon introduction of the wildtype *TAC1B* sequence alone.

**Conclusions:** These studies demonstrate that mutations in *C. auris ERG11* and *TAC1B* both contribute to the high degree of fluconazole resistance observed among clinical *C. auris* isolates. Additionally, the contribution of specific mutations in either of these genes vary significantly, with the mutation encoding the A640V substitution in *TAC1B* alone most significantly increasing resistance to the entire triazole class. Further research is needed to determine if this difference in impact on triazole resistance extends to other *ERG11* and *TAC1B* mutations.

## P141 Chronic Disseminated Bipolaris Phaeohyphomycosis from Prolonged Hazardous Indoor Exposure to Documented Airborne Stachybotrys and Bipolaris

**Irene Grant and Ameet Kamat**

Integrative Medicine Group

**Objectives:** Case Report describing prolonged course of chronic Bipolaris infection, Methods and Materials: Prospective Medical Care and photographic timeline.

**Results:** Within a year of living with a collapsed bathroom ceiling, recurrent water/sewage intrusion and elevated airborne Stachybotyrs and Bipolaris spores, a 27 year-old female developed widespread ulcerative cutaneous/oral lesions, a rapidly growing 15 cm black nodular necrotic liver mass occupying 70% of the R. lobe, which at surgery was adherent to the vena cava and diaphragm and presumptively diagnosed as focal nodular hyperplasia without any fungal studies done.

Over the next 10 years she developed progressive relapsing multisystem symptoms with disfiguring subcutaneous face-scalp swelling and induration, diffuse ulcerations with pox- keloid-like scarring, chronic nasal bloody discharge with bloody tissue “chunks”, granulomatous rhinosinusitis, nasal osteochondritis, chronic scalp and neck lymphadenitis, diffuse scalp alopecia, 2–3” hairline recession, striking hair change [straight blond to curly jet black], dark olive green-blue-grey oral thrush, swollen red-purple soft-palate and throat, recurrent fluctuating neurological symptoms with neurocognitive impairment, twitching, foot drop, choreiform movement disorder, paresis, CSF lymphocytosis and a putamen basal ganglia lesion.

Cellular and adaptive immunodeficiencies were repeatedly documented: lymphopenia, Eosinophilopenia [undetectable], low CD3 (Mature T memory cells), CD8 Lymphocytes (T suppressor cells), CD16+CD56 Natural Killer Lymphocytes, Low CD19+ lymphocytes (B cells), and Low IGG subclass 3.

She received numerous empiric treatments over the next 10 yrs from multiple physicians for multiple unproven diagnoses (leukcocytoclastic vasculitis, Raynaud’s, Sjogren’s, Systemic Lupus Erythematosus, PANDAS, chronic EBV, “sero-negative” Lyme disease, seronegative tick borne disease, lepromatous leprosy, Sjogren’s, chronic EBV, leukcocytoclastic vasculitis, granulomatous sinusitis, encephalitis, and Leishmaniasis).

In Year #6 nasal fungal culture documented filamentous *Aspergillus*-like spp. “Not fumigatus, flavus or niger”. Partial improvement in skin and neurological occurred with courses of voriconazole, and later albendazole.

Every re-exposure to moldy indoor environments, steroids, and methotrexate acutely triggered further deterioration, flaring neurological symptoms. Symptoms temporarily abated with repeated sinus surgical resections and partially responded to Amphotericin irrigation, and voriconazole or albendazole.

In Year #10, nasal hawk-bill deformity developed with fronto-ethmoid mucoperiosteal thickening and a polypoid mass that grew despite broad-spectrum antibacterial therapy. At microscopy showed inflamed granulomatous tissue, foreign body giant cell reaction, nasal Septum Bone Cartilage with many white blood cells, yeasts, molds, and rare thin Branching Gram Positive rods on gram stain; septated hyphae on KOH, and Candida lipolytica and Bipolaris on culture. Strikingly obvious clinical improvement in all symptoms occurred within 3 weeks of intravenous liposomal Amphotericin. Clinical course complicated by renal toxicity. Recurrences occurred with interruption in antifungal treatment.

Photograph Time of Physical Findings to be provided.

**Conclusions:** Bipolaris infection requires definitive diagnosis and prolonged monitoring to be effectively treated.

## P142 Highly Drug Resistant Mucormycosis in a COVID Infected Patient

**Khaled Alobaid ^1^, Sara Mazloum ^2^ and Abdullah Al-Hatmi ^3^**

^1^ Mycology Reference Laboratory, Mubarak Al-Kabeer Hospital, Kuwait
^2^ Microbiology Unit, Medical Laboratory Department, Jaber Al Ahmad Hospital
^3^ Natural & Medical Sciences Research Center, University of Nizwa

**Introduction:** During it’s second year, SARS CoV2 have continued to infect a huge number of people causing considerable morbidity and mortality. The presence of secondary fungal infections such as candidemia and aspergillosis in critically COVID 19 infected patients pose diagnostic and therapeutic challenges and seem to worsen the patients outcome. Recently, mucormycosis related cases have been reported in different geographic regions. We report here for the first time a case of mucormycosis in a COVID 19 infected patient.

**Case report:** An-85-year old man, who is known to have diabetes, hypertension and ischemic heart disease, was admitted with pneumonia one week after being infected with SARS CoV2. His chest X ray showed diffuse bilateral infiltration. He was treated with ceftriaxone 1 gram once daily, methylprednisone 1 mg/kg once daily, and oxygen therapy 5 L. One week later, he developed a stroke and was shifted to ICU for ventilatory support. Methylprednisone was replaced by dexamethasone 6 mg once daily. An endotracheal aspirate was sent for culture which grew a mould next day. Few days later, he developed acute renal impairment and septic shock requiring inotropic support. The mould was identified as *Rhizopus arrhizus* and the patient was started on liposomal amphotericin B 50 mg once daily. However, the patient died few days later. Antifungal susceptibility testing performed by E test revealed very high MICs against amphotericin B (>32 µg/mL) and posaconazole (>32 µg/mL).

**Discussion:** Although the apparent coexistence of COVID 19 and mucormycosis is debatable and is currently subjected to a critical analysis, the reported cases so far, reveal a significant morbidity and mortality. Our patient was diabetic and had received steroids, and both are known risk factors for mucormycosis. In addition, his old age, occurrence of COVID 19, and new onset stroke have resulted in a poor outcome. What is more frightening, is the presence of multidrug resistance associated with the etiological agent: *Rhizopus arrhizus*. The case calls for comprehensive and critical analysis in mucormycosis associated COVID 19 cases to understand pathogenesis and epidemiology of such dreadful coinfection. In addition, antifungal susceptibility testing should be standard of care especially in invasive infections.

## P143 A Monocentric Outbreak of *Saprochaete clavata* Seen in COVID-19 Pandemic

**Irem Nida Akkoyun ^1^, Ozlem Alhan Guncu ^1^, Nurcan Duman ^2^, Arzu Ilki ^2^ and Zekaver Odabasi ^1^**

^1^ Department Of Infectious Diseases and Clinical Microbiology, Marmara University
^2^ Department Of Medical Microbiology, Marmara University

**Objectives:** *Saprochaete clavata* (formerly *Geotrichum clavatum*) is a yeast-like filamentous fungus that is usually first seen as fungal growth with pseudohyphae formation in blood cultures. *S.clavata* isolates are usually resistant to echinocandins and have high mortality rates.

**Materials & Methods:** We describe 7 cases of *S.clavata* infections between March–May 2021 in Marmara University Hospital. All yeast strains from blood, respiratory, and urine specimens were grown on blood agar at 37 °C for 48 h in aerobic conditions. These strains were subcultured onto Sabouraud dextrose agar (SDA) and incubated at 30 °C and 37 °C for 48 h in mycology laboratory. *S clavata* formed dry cottony colonies with frosted glass appearance on the agar, macroscopically (Figure 1). In microscopic examination true fragmented hyphae, pseudo-hyphae, and arthroconidia formations were determined (Figure 2). Isolates were identified by MALDI-TOF MS (Vitek MS, BioMerieux, Marcy-l’ÉtoileFrance).

**Results:** The clinical characteristics and therapeutic agents of patients are summarized in table-1. Five of seven patients were hospitalized in the intensive care unit and the other 2 patients were hospitalized in hematology ward. The source of fungal isolates was; 4 “blood”, 3 “urine” and 2 “deep tracheal aspirate”. Four patients had fungemia, two patients were diagnosed with upper urinary tract infection, and one possible fungal pneumonia. Six patients had the Covid-19 infection. Three patients had hematological malignancy and were under anti-mold prophylaxis included in their chemotherapy regimens. All patients were taking a high dose of steroids and broad-spectrum antibiotics. One patient died on the day of fungal blood growth without receiving any antifungal treatment, all other 6 patients were treated with the combination of liposomal amphotericin B and +/− voriconazole. The treatment of two patients with acute leukemia was completed 14 days after the first negative blood cultures.

**Conclusions:** *S.clavata* infection represents a life-threatening mycosis in immunosuppressive patients and first time in literature we showed an outbreak of *S.clavata* in Covid-19 patients. The use of high-dose steroids in the treatment of Covid-19 is probably the most important risk factor for the development of *S.clavata* infection.

## P144 COVID-19 Associated Mucormycosis: Mixed Mucorales Infection

**Harsimran Kaur ^1^, Rimjhim Kanaujia ^1^, Shivaprakash M Rudramurthy ^1^, Naresh K Panda ^2^ and Arunaloke Chakrabarti ^1^**

^1^ Department of Medical Microbiology, PGIMER
^2^ Department of Otolaryngology, PGIMER, India

**Objectives:** To describe five cases of COVID-19 associated mucormycosis (CAM) caused by two different *Mucorales*.

**Materials & Methods:** Endoscopic nasal scrapings/biospy from the patients suspected of mucormycosis before and after surgery were subjected to calcoflour white potassium hydroxide (KOH) microscopy and culture on Sabouraud dextrose agar (SDA) incubated at 25 °C and 37 °C and brain heart infusion (BHI) agar incubated at 25°C. Culture was identified phenotypically by lactophenol cotton blue preparation. Detailed history of the patients was obtained from their medical records.

**Results:** We identified five cases of CAM where more than one type of *Mucorales* were obtained in one or more samples. (Table 1) The mean age of the patients was 39 years and male: female ratio was 3:2. All these patients had history of diabetes (two recently diagnosed and rest ranging from 2–9 years). They presented with symptoms after 1–29 days of diagnosis of COVID-19. All patients presented with symptoms of rhino-orbital mucormycosis (ROM) namely facial/orbital pain, facial/orbital swelling, toothache and headache. The duration of symptoms ranged from 1–4 days. Direct microscopy of preoperative and post operative samples of all patients showed presence of aseptate, ribbon like hyphae (Image 1). All except one grew single species of *Mucorales* in preoperative samples [*Rhizopus arrhizus* (n = 2); *Apophysomyces variabiliis* (n = 1); *Lichtheimia corymbifera* (n = 1) and *Rhizopus homothallicus* and *R. arrhizus* (n = 1)] Concurrent growth of two different *Mucorales* was noted in postoperative samples of four patients [*R. arrrhizus* and *R. homothallicus* (n = 2); *A. variabiliis* and *L. corymbifera; R. arrhizus* and *Mucor* spp. (Image 2)] while one patient who grew *L. corymbifera* in preoperative sample showed growth of *R. homothallicus* in post-operative sample. All patients were managed surgically (maxillectomy or orbital exenteration) and with liposomal amphotericin B 3–5 mg/kg/day. One patient expired while others continue to improve.

**Conclusions:** Mixed infection by more than one *Mucorales* in CAM is unique. The emergence of *Mucorales* (*R. homothallicus*) other than the commonest *R. arrhizus* in India and the occurrence of dual infection warrants epidemiological investigation. Diabetes remains the most important risk factor for CAM in India. The interaction of *Mucorales* and SARS-CoV-2 might further provide some insight into emergence of CAM in India.

Image legends

Image 1: Calcoflour white potassium hydroxide mount showing flourescing aseptate, ribbon like hyphae of *Mucorales* (×400)
Image 2: Sabouraud dextrose agar showing mixed culture of *R. arrhizus* (greyish black fluffy growth on the left) and *Mucor* spp. (yellowish black fluffy growth in left and right tubes).

**Table 1.** Demographic, clinical and mycological details of cases of mixed *Mucorales* infection.




**Age/Sex**

**Residence**

**Diabetes Duration**

**Symptoms**

**Symptoms Post COVID-19 Diagnosis (Days)**

**Management**

**Outcome**

**Sample**

**Smear/Culture**
123/MUttarakhandRecentToothache, left orbital pain, swelling × 3 days29Maxillectomy and OE, L-AMBImprovingNasal scrapingAseptate *L. corymbifera*Post-opAseptate *R. homothallicus*235/FUttar Pradesh8 yearsHeadache, toothache, left orbital pain, swelling × 4 days1Maxillectomy and OE, L-AMBImprovingNasal scrapingAseptate *R. arrhizus*Post-opAseptate *Mucor* spp., *R. arrhizus*332/MHaryanarecentRight facial pain, orbital swelling × 3 days2Maxillectomy and OE, L-AMBExpiredNasal scrapingAseptate *A. variabilis*Post opAseptate *L. corymbifera, A. variabilis*462/MHaryana9 yearsRight facial pain, swelling × 1 day1Maxillectomy, L-AMBImprovingNasal scrapingAseptate *R. arrhizus*Post opAseptate *R. homothallicus, R. arrhizus*541/FHaryana2 yearsRight orbital pain, swelling × 4 days1Maxillectomy, L-AMBImprovingNasal scrapingAseptate *R. homothallicus, R. arrhizus*Post opAseptate *R. homothallicus, R. arrhizus*
Abbreviations: L-AMB: Liposomal Amphotericin B; OE: Orbital exenteration; Post-op: Post-operative sample.


## P145 Candidemia Clonal Outbreak Caused by Emerging Fluconazole-Resistant *Candida parapsilosis* Isolates during the SARS-CoV-2 Pandemic in a Northern Italy Hospital

**Valentina Lepera, Andrea Zappavigna, Chiara Gorrini, Roberta Schiavo, Irene Peroni, Lorena La Vergata, Claudia Cordini, Gabriella Tocci, Paolo Gigante, Domenico Caleca, Giulia Bandieramonte and Giuliana Lo Cascio**

Unit of Microbiology, Department of Clinical Pathology, “Guglielmo da Saliceto” Hospital

**Objectives:** *Candida parapsilosis* (CPA), the most common species of *Candida* non *albicans* isolated from bloodstream infections, although usually susceptible to azoles, had been recently reported as an emerging multi-resistant yeast. Azoles play an important role in the therapeutic management of invasive candidiasis. In recent years, *Candida* isolates with acquired resistance to azoles have been reported more and more frequently. Therefore, antifungal susceptibility testing and the detection of mutations in resistance genes are becoming increasingly important to detect antifungal resistance and determine the underlying resistance mechanisms. Fluconazole prevents fungal cell growth by inhibiting 14-α-demethylase, which is responsible for the production of an ergosterol precursor and is encoded by the gene ERG11. As the current SARS-CoV-2 pandemic has evidenced that COVID-19 patients are more susceptible to secondary fungal infections, the aims of this study were: (1) to evaluate the incidence of invasive infection due to fluconazole-resistant CPA (CPA-R) strains during the SARS-CoV-2 pandemic, (2) to detect mutations in resistance genes of azole-resistant strains by sequencing, focusing on mutations and/or alterations of the ERG11 gene.

**Materials & Methods:** This study was conducted at the “Guglielmo da Saliceto” Hospital, Piacenza, Northern Italy in order to investigate the antifungal susceptibility to fluconazole of CPA isolates in blood cultures in two different periods: period 1 (March 2019–January 2020; control period) and period 2 (March 2020–January 2021; SARS-CoV-2 pandemic). Yeast identification was performed by using MALDI-TOF MS (Vitek MS, bioMérieux, France) and antifungal susceptibility was evaluated by using the Vitek2 system (bioMériueux, Marcy L’Etoile, France) and confirmed with Sensititre Yeast One YO10 (Thermofisher Scientific, Waltham, MA, USA) microdilution broth. ERG11 gene of the isolates was analyzed by sequencing according to Sanger method.

**Results:** The comparative evaluation revealed a 26.9% increase of *C. parapsilosis* blood isolates and a 160% increase of CPA-R strains in the period 2. Among the patients with CPA-R infection, 98.8% (11 of the 13 cases) had a diagnosis of COVID-19. Moreover, two possible clusters were observed analysing the temporal distribution of CPA-R strains during SARS-CoV-2 pandemic period: the first from April to June 2020 with 7 CPA-R strains and the second from December 2020 to January 2021 with 4 CPA-R strains. These clusters appear to follow a pattern overlapping the epidemiological waves of SARS- CoV-2 pandemic in Piacenza. The preliminary evaluation of ERG11 gene sequences in the isolated strains allowed to highlight the presence of Y132F mutation.

**Conclusions:** The observed increase in both infection and resistance rates among CPA blood isolates during period 2 and the presence of two clusters could be linked to COVID-19 from different points of view: severe COVID-19 infections expose patients to invasive secondary fungal infections and the spread of multidrug resistant strains likely escaped from control due to extraordinary involvement of infection control teams in the containment efforts of SARS-CoV-2 pandemic in Piacenza, one of the most affected Italian cities. The presence of the same mutation in the whole of the strains suggests the existence of a spreading cluster. Further investigations will be performed by NGS sequencing in order to confirm our suspicion.

## P146 Molecular Epidemiology and In Vitro Susceptibility of 70 Respiratory Isolates of *Paecilomyces* spp.: A Multicenter Study

**Lorra Monpierre ^1^, Nawel Aït Ammar ^1^, Anne-Cécile Normand ^2^, Isabel Valsecchi ^3^, Juliette Guitard ^4^, Arnaud Riat ^5^, Antoine Huguenin ^6^, Christine Bonnal ^7^, Boualem Sendid ^8^, Lilia Hasseine ^9^, Hélène Raberin ^10^, Loïc Favennec ^11^, Stéphane Ranque ^12^, Renaud Piarroux ^2^, Eric Dannaoui ^13^ and Françoise Botterel ^1^**

^1^ Henri Mondor Hospital APHP
^2^ La Pitié-Salpêtrière Hospital
^3^ UPEC
^4^ Saint-Antoine Hospital
^5^ Geneva University Hospital
^6^ CHU Reims
^7^ Bichat Hospital
^8^ CHU Lille
^9^ CHU Nice
^10^ CHU Saint-Etienne
^11^ CHU de Rouen
^12^ CHU Marseille
^13^ HEGP

**Objectives:** *Paecilomyces* spp. is a rare emerging fungal pathogen where *P. lilacinus* and *P. variotii* are the mostly species reported. Over the past few years, taxonomic revision of this fungus shown that *P. variotii* represents a species complex while *P. lilacinus* corresponds to a different genus namely *Purpureocillium lilacinum*. The aims of this study were to investigate the molecular and proteomic identification of clinical *Paecilomyces* spp. isolates and to determine their antifungal susceptibility profiles.

**Materials & Methods:** Seventy *Paecilomyces* spp. isolated from respiratory patient’s samples were collected from eleven university hospitals in France and Switzerland. Species identification was performed by ITS, D1/D2 and β-tubulin sequencing gene and by Matrix Assisted Laser Desorption Ionisation-Time Of Flight (MALDI-TOF). Protein spectra were analyzed by Brucker^®^ Microflex-LT spectrometer (using both the MALDI Biotyper v3.0 and MSI v1 references databases), and by LT2-Andromas^®^ spectrometer. For antifungal susceptibility, minimal inhibitory concentrations (MIC) or minimal effective concentrations (MEC) were determined for 8 antifungal drugs (4 azole, 1 polyene and 3 echinocandins drugs).

**Results:** Among the 70 *Paecilomyces* spp., identified by ITS sequencing, 25 (36%) were *P. variotii stricto sensu*, 18 (26%) *P. formosus* and 27 (38%) *P. lilacinum*. No strain has been responsible for an invasive fungal infection. The identification concordance between ITS and Microflex using Biotyper and MSI references databases was 65.7% and 97.1%, respectively. Concordance with Andromas was 92.9%. These differences mainly concerned *P. formosus*, which was misidentified by MALDI-TOF. The D1/D2 and β-tubulin gene sequencing are in progress. All *P. lilacinum* had high amphotericin B MICs and echinocandins MECs unlike *P. variotii* and *P. formosus*. Azoles MICs were variable (Table 1).

**Conclusions:** In our study, MALDI–TOF MS proved to be a rapid, reliable, and logistically simplified alternative to sequence-based analysis for the routine identification of *Paecilomyces* spp. Antifungal susceptibility is variable depending on the species. Therefore, identification is essential for management of invasive infection.

**Table 1.** Susceptibility of *Paecilomyces* isolates to antifungals by EUCAST.


Species


VCZ

PCZ

IVZ

ICZ

AMP

CAS

MYC

ANI
*P. variotii* *Stricto sensu* (n = 25)Range (mg/L)0.03–80.015–80.06–80.06–80.06–80.03–80.015–0.50.015–1GM (mg/L)5.1790.1004.8880.5740.4181.7670.0470.068*P. formosus* (n = 18)Range (mg/L)1–80.015–24–80.03–80.125–80.03–80.015–80.015–8GM (mg/L)7.1270.0527.6980.7600.3810.9550.0970.077*P. lilacinum* (n = 27)Range (mg/L)0.12–80.015–80.5–80.5–88–88–88–88–8GM (mg/L)0.4490.6152.4756.8178888

## P147 Isavuconazole for the Treatment of Patients with Possible Coronavirus Disease-Associated Aspegillosis (CAPA): A Single-Center Matched Case-Control Study

**Vasso Georgakopoulou ^1^, Antonios Markogiannakis ^1^, Dimitris Basoulis ^1^, Angeliki Tsifi ^2^, Athanasios Kakasis ^2^, Nikolaos V. Sipsas ^2^ and Maria N. Gamaletsou ^2^**

^1^ Medical School of Athens, Laiko General Hospital
^2^ Pathophysiology Department, Medical School of Athens, Laiko General Hospital

**Objectives:** The aim of this analysis was to assess differences in baseline clinical characteristics, selected biochemical markers and 30-day mortality in a group of patients with possible coronovirus disease-associated aspergillosis (CAPA) who were treated with isavuconazole compared with COVID-19 patients without CAPA in a matched case-control study conducted in General Hospital of Athens “Laiko”, a 535-bed tertiary hospital.

**Materials & Methods:** Sixteen among 1200 (1.3%) consecutive patients admitted in six COVID-19 clinics (5 internal medicine and 1 ICU), during the second phase of the pandemic in Greece (January–May 2021) were diagnosed with possible (15 patients) or confirmed (1 patient) CAPA. All 16 patients were treated with isavuconazole (isavuconazole treatment group-ITG). All patients in the ITG were matched with two patients presenting with common characteristics (sex, age, hospitalized in the same clinic and in the same time-period). In the analysis we have included a number of baseline characteristics: age, severity of illness, risk factors for coronavirus disease (e.g., obesity, diabetes, treatment with immunosuppressive agents), specific biochemical markers: white blood cells (WBC), C-reactive protein (CRP), number of lymphocytes, ferritin and X-ray score and finally the 30-day mortality. Associations between isavuconazole use for CAPA and other clinical/laboratory factors were investigated using univariable and multivariable conditional logistic regression models. Probability of death within 30 days from hospital admission was investigated using logistic regression models. *P*-values less than 0.05 were considered statistical significant.

**Results:** Regarding the use of isavuconazole for possible or confirmed CAPA, univariable analyses revealed a significant difference in favor of isavuconazole use in more severely ill patients (*p* = 0.031; Table 1). Moreover, isavuconazole was administered more frequently in patients who had more risk factors for severe COVID-19 (*p* = 0.04). Finally there was a notable trend towards using isavuconazole in patients with higher ferritin levels (*p* = 0.05). There was no statistically significant difference in the use of isavuconazole with regards to other variables: WBC, CRP, number of lymphocytes and X-ray score (Table 1). The patients who were treated with isavuconazole had a higher 30-day mortality compared to the control group (*p* = 0.007; Table 2). Other factors associated with increased 30-day mortality were the severity of illness (*p* < 0.001), age (*p* = 0.036), lymphopenia (*p* = 0.008) and possibly elevated ferritin levels (*p* = 0.075; Table 2). The only variable found to be statistically significant in multivariable analysis was the level of lymphocytes (OR = 0.78 per 100/mL; *p* = 0.022) whereas a trend was observed regarding number of risk factors (OR = 2.53 per 1 additional factor; *p* = 0.057).

**Conclusions:** In this study, we noticed that the use of isavuconazole as a treatment for possible or confirmed CAPA was highly associated with increased disease severity, higher ferritin levels and higher number of risk factors for severe COVID-19. Therefore, the patients who were treated with isavuconazole had a higher 30-day mortality compared to the control group. An increased 30-day mortality was also related to the severity of illness, number of risk factors and degree of lymphopenia. These findings are in accordance with the data that have been published to date.

## P148 Exploring the Diversity of Yeast-like Prototheca Microalgae in Aquatic Environments: Comments on the Taxonomy and Description of Three New Species

**Tomasz Jagielski and Mateusz Iskra**

University of Warsaw

**Objectives:** The *Prototheca* genus (Trebouxiophyceae) comprises unicellular, nonphotosynthetic, yeast-like microalgae, associated with rare yet potentially very serious infections of animals and humans, collectively referred to as protothecosis. In animals, the disease most commonly affects dairy cattle, resulting in (sub-)clinical mastitis, while in humans the predominant manifestations are linked to cutaneous, articular, and systemic involvement. The taxonomy of the *Prototheca* genus has long been controversial and frequently revised. Recent studies based on the phylogenetic analysis of the apocytochrome B-coding sequence data have established a new taxonomic classification system of the *Prototheca* algae, installing within the genus a total of 15 species.

Although the *Prototheca* algae are believed to be ubiquitous in nature, since the mid-1980s, no studies have investigated in depth their environmental habitat. The purpose of this study was to explore the occurrence of Protothecae in a wide range of natural and artificial aquatic environments and demonstrate the species diversity of the algae by using a molecular taxonomic profiling approach.

**Materials & Methods:** In total, 362 samples were collected from freshwater and artificial water reservoirs across Poland over a 2-year period (2018–2020). Liquid and semisolid samples were spread on the *Prototheca* Isolation Medium (PIM) plates, either directly or after 48-h pre-incubation in liquid PIM. Plates were incubated at 30 °C for 2–5 days. Colonies suspected of being *Prototheca* spp., upon macro- and micromorphology observations, were subjected to species-level identification, by using PCR-sequencing of the *CYTB* gene. To better recognize the phylogenetic relatedness, ribosomal DNA sequencing was performed on selected *Prototheca* isolates. The assimilation profiles of new species were examined using API®20C AUX system (bioMérieux, France).

**Results:** Of the samples collected, 51 (14%) yielded *Prototheca* growth with *P. wickerhamii* being the most frequently isolated species (17 or 33.3%), followed by *P. pringsheimii* (12; 23.5%), *P. cerasi* (7; 13.7%), *P. bovis* (5; 9.8%), *P. ciferrii* (3; 5.8%), *P. cookei* (2; 3.9%), and *P zopfii* (1; 1.9%). Based on the *CYTB* gene and rDNA phylogenies, four isolates, designated PK1, PK2, PK6, and W3, were conspicuously different from all other *Prototheca* species described so far. Their *CYTB* gene sequences showed less than 88% similarity to each other and less than 96% to all other *Prototheca* species. Colonies of those strains were creamy-white, slightly raised, with a smooth surface and even margins. Each strain was of butyrous consistency except W3, whose colonies were clearly slimy. Upon auxanography, all strains utilized glucose, galactose, trehalose, whereas glycerol was assimilated only by W3 and PK2. Furthermore, PK1 grew much more slowly than the remaining isolates. The combined geno- and phenotyping concluded in the proposal of three new *Prototheca* species, namely *Prototheca lentecrescens, Prototheca fontanea*, and *Prototheca vistulensis*.

**Conclusions:** After four decades, this is the first study to explore thoroughly the occurrence of *Prototheca* spp. in water sources. Unexpectedly, *Prototheca* were isolated at a relatively low rate. This is in contrast to a common belief of environmental ubiquity of the algae. However, finding three novel species among so few strains recovered speaks of important species diversity of the *Prototheca* algae.

## P149 Fungal Empyema Thoracis an Emerging Entity, a Case Series from Pakistan

**Nousheen Iqbal ^1^, Aqusa zahid ^2^, Kausar Jabeen ^2^ and Muhammad Irfan ^2^**

^1^ Jinnah Medical and Dental College and Aga Khan Hospital
^2^ Aga Khan Hospital

**Objectives:** Fungal empyema is a rare entity which is associated with high mortality. It is mostly seen in immunocompromised host. However there is little data available on fungal empyema from our country on pathogenesis, risk factors, treatment and outcome.

**Materials & Methods:** A retrospective observational study was done on proven fungal empyema cases in admitted patients at Aga Khan hospital Karachi Pakistan during January 2018 to May 2021.

**Results:** Total 26 patients were diagnosed, 16 (61.5%) were male. Mean age was 43.58 ± 20 years, common underlying comorbid was diabetes 10 (38.4%), folowed by hypertension 9 (34.6%), Malignancy 6 (23.07%) and 4 (15.38%) liver disease. *Candida* spp. Isolated in 18 (69.23%) patients [commonest was *tropicalis* in 9 (50.0%) and *albicans* in 6 (33.33%) patients], follwed by *aspergillus*spp. 7 (26.9%) patients, *fusarium* and *mucor* in 1 patient respectively. Video assisted thoracoscopy done in 11 (42.3%), conservative managemnet (chest tube& antifungal) done in 12 (46.1%) patients. Overall mortality seen 9 (34.6%) patients, 10 (38.4%) devloped respiratory failure, 11 (42.3%) had clinical improvement and 6 (23.07%) patients were lost to followup.

**Conclusions:** Our data suggest fungal empyema is not uncommon and necessitate high index of suspicion to prevent delay in diagnosis, treatment and imrpove outcome.

## P150 Diabetes as Risk Factor for COVID-19 Associated Mucormycosis

**Dora Edith Corzo Leon ^1^, Thomas Pichl ^1^, Luis David Chora-Hernandez ^2^, Rosa Colamarino ^1^ and Carol Munro ^1^**

^1^ University Of Aberdeen
^2^ Hospital General Dr. Miguel Silva SSM

**Objectives:** The aims of this study is 1) to describe characteristics of COVID-19 associated mucormycosis (CAM) in diabetic individuals and 2) to describe phagocytosis of *Rhizopus oryzae* in an in vitro model simulating COVID-19 and high glucose environment.

**Methods:** Previous informed consent, clinical characteristics and outcomes from CAM cases have been identified during 2020 and 2021 in a newly established mycology reference centre in Western Mexico. At laboratory level, J774.1 macrophages were exposed to high glucose concentrations (4 mg/mL) and/or previously heat-inactivated (HI) supernatant containing viral proteins from wild type SARS-CoV-2 propagation in Vero E6 cells. After 24 h, these same macrophages were challenged with *R. oryzae* spores for 3 and 5 h. Engulfment and fungal killing was evaluated.

**Results:** Six CAM cases have been identified in diabetic individuals. Infections were diagnosed 20 days (IQR 13–25) after COVID-19 diagnosis. All patients received corticosteroids for more than 7 days, 5/6 had rhino-ocular damage and 3/6 had palate ulcer. Four cases were treated with amphotericin B deoxycholate and two were also treated with surgery. Mortality rate was 66% (4/6). In vitro, *R. oryzae* growth was faster when grown in both high glucose and HI viral supernatants compared to control culture medium. Neither high glucose nor viral supernatants affected uptake of *R. oryzae* spores by macrophages but intracellular hyphae production was higher in these two conditions after 3 and 5 h of co-incubation.

**Conclusions:** Diabetic individuals treated with corticosteroids due to severe COVID-19 are at high risk of mucormycosis. High glucose concentration and the COVID-19 environment impact on the immune response to Mucorales such as *R. oryzae*.

## P151 Autochthonous *Cryptococcus bacillisporus* (AFLP5/VGIII) Infection in a Grey Parrot (*Psitaccus erithacus*), from Portugal

**Carolina Silva ^1^, Carles Juan-Sallés ^2^, Joana Mendes ^1^, Ana Mendes ^1^, Mariana Ruivo ^1^, Juan Luis Abad ^3^, Ferry Hagen ^4^ and Francisca Colom ^3^**

^1^ VetExoticos, Veterinarian Clínic
^2^ Noah’s Path, Veterinary Pathology Laboratory
^3^ Medical Mycology, University Miguel Hernández, Institute for Healthcare and Biomedical Research of Alicante (ISABIAL)
^4^ Department of Medical Microbiology, Westerdijk Fungal Biodiversity Institute, University Medical Center

**Objectives:** To report an autochthonous case of cryptococcosis caused by *Cryptococcus bacillisporus* (AFLP5/VGIII) in a 12-year-old female grey parrot (*Psittacus erithacus*) born and bred in captivity in Portugal.

**Materials & Methods:** The bird developed a several slow-growing masses (about 8 months) in the maxillar portion of the beak (rhinoteca). Given the suspicion of neoplasia, a biopsy of the mass was taken and sent for histopathological study, which detected the presence of yeasts morfologically suggestive of *Cryptococcus*. After obtaining the biopsy results, a second sample was obtained and cultured onto Sabouraud dextrose Agar. The yeast obtained in culture were further analyzed by phenotypic tests including CGB agar, urease and phenoloxidase production, carbon auxonograme by Auxacolor system (Biorad®), presence of polysaccharide capsule and melanine production onto L-DOPA agar, as well as molecular analysis by URA5-RFLP. Antifungal susceptibility was checked for amphotericin B, fluconazole and voriconazole using the E-test method. Multi-Locus Sequence Typing was performed to obtain the molecular profile of the strain that was compaired with other *C. bacillisporus* strains availables in public databases.

**Results:** Histopathology showed severe chronic granulomatous and necrotising dermatitis with serocellular crusting, fibrosis and intralesional yeast-like structures. Phenotypic and molecular analysis of the isolated yeast allowed the identification of *Cryptococcus bacillisporus* (AFLP5/VGIII) as the cause of the infection. Comparison of the MLST profile showed that the strain clustered within the group of *C. bacillisporus* strains from Mexico (Figure 1). The antifungal susceptibility testing showed a low response to fluconazole which was the initial empiric treatment administered, therefore, it was switched to amphotericin B in the parrot’s drinking water (600 mg per litre of water) with a good response after 6 months. The masses size was reduced significantly, although some chronic lesions remained in the rhinoteca, consisting mainly in queratine deposition anomalies and deformed beak growth.

**Conclusions:** *Cryptococcus bacillisporus* is a relatively rare cause of cryptococcosis in humans and animals, and most cases have been described in warm areas of the Americas and Oceania. Its description as a cause of cryptococcosis is exceptional in Europe.

**Figure 1.** Phylogenetic tree with *Cryptococcus bacillisporus* isolates clustered by the MLST information.

## P160 The Rise of Zoophilic Dermatophytes during Corona Pandemic in Germany

**Silke Uhrlass ^1^, Daniela Koch ^1^, Hanna Muetze ^1^, Constanze Krueger ^1^ and Pietro Nenoff ^1^**

Laboratory of Medical Microbiology

**Objectives:** During the months-long lockdown due to the Corona pandemic, significantly more pets were probably bought and kept. Whether more zoophilic dermatophytes are subsequently isolated, and which species are in the foreground, is the focus of this study.

**Methods & Materials:** In the period of one year, from March 2020 to February 2021, all zoophilic dermatophytes from all submissions to the Mölbis laboratory were recorded. Both the cultural and the molecular evidence of fungal detection directly from skin scales, hair roots, in individual cases from nails were taken into account. For dermatophyte DNA detection an in-house-PCR (Polymerase chain reaction)-Elisa (Enzyme linked immunosorbent assay) was used. In distinct cases, identification of dermatophytes was confirmed by sequencing of the “internal transcribed spacer”- (ITS) region of the rDNA, and of the gene of the Translation Elongation Factor (TEF)-1α.

**Results:** In 2.7% of the 21290 materials studied, zoophilic dermatophytes were detectable with the PCR-Elisa. The total of 579 zoophilic dermatophytes split as follows (Figure 1): *Trichophyton* (*T*.) *benhamiae* 186 (32.1%), T. mentagrophytes 173 (29.9%), *T. quinckeanum* 110 (19.0%), *Microsporum* (*M*.) *canis* 78 (13.5%), *T. verrucosum* 22 (3.8%), *Nannizzia* (*N*.) *persicolor* 8 (1.4%), *T. erinacei* 1 (0.2%) and *T. equinum* 1 (0.2%). *T. benhamiae* had the highest prevalence from June to September 2020, then again in December. *T quinckeanum* is associated with a sharp increase in the mice population in Germany in 2020, a significant increase was found in the months September 2020 to January 2021 (Figure 2). *T. mentagrophytes* had a conspicuous peak in September, while *M. canis* did not have a conspicuous peak until November 2020. Up to 50% of the dermatophytoses caused by *T. mentagrophytes. T. quinckeanum* and *M. canis* affected children and adolescents, in the case of *T. benhamiae* it was as much as two thirds. Tinea corporis was the most common, followed by Tinea faciei and Tinea capitis. *M. canis* infections affected more common the scalp than the face.

**Conclusions:** Zoophilic dermatophytes were increasingly isolated during the lockdown due to the Corona pandemic in Germany. In the first place, the dermatophyte *T. benhamiae* from guinea pigs was found as the cause of Tinea corporis, Tinea faciei and Tinea capitis in children and adolescents. A significant proportion of dermatophytoses concerned adults. *T. quinckeanum* is an emerging pathogen in Germany with unprecedented high infection rates in 2020.

**References:** Uhrlaß, S.; Schroedl, W.; Mehlhorn, C.; Krüger, C.; Hubka, V.; Maier, T.; Gräser, Y.; Paasch, U.; Nenoff, P. Molecular epidemiology of *Trichophyton quinckeanum*—A zoophilic dermatophyte on the rise. *J. Dtsch Dermatol. Ges.* **2018**, *16*, 21–33.

## P161 Otomycosis in Northern Iran: Epidemiology, and Diagnosis

**Behrad Roohi ^1^, Tahereh Shokohi ^1,2^, Shadman Nemati ^3^, Abbas Alipour ^4^, Leila Faeli ^1^, Sabah Mayahi ^2^ and Iman Haghani ^1^**

^1^ Invasive Fungi Research Center, Communicable Diseases Institue, Mazandaran University of Medical Sciences
^2^ Department of Medical Mycology, School of Medicine, Mazandaran University of Medical Sciences
^3^ Department of Otolaryngology and Head and Neck Surgery, Otorhinolaryngology Research Center, School of Medicine, Guilan University of Medical Sciences
^4^ Department of Community Medicine, Faculty of Medicine, Mazandaran University of Medical Sciences

**Objectives:** Otomycosis is a common type of external ear infection caused by a wide range of fungal species and affects the external auditory canal in tropical and subtropical regions. Due to the long period of treatment and recurrence, this may be challenging for physicians. It also can be fatal in acute invasive or chronic invasive forms. This study aimed to assess the epidemiologic pattern and etiological agents of otomycosis north of Iran.

**Methods:** In this cross-sectional study, all patients with clinical suspicion of otomycosis enrolled in a sequential random manner at ENT subspecialty referral center from October 2020 in a sequential random sampling method in Guilan province, near the Caspian Sea, northern Iran. Patients’ demographic characteristics, clinical findings, laboratory results, and risk-related disease factors were recorded. Each sample was examined by direct examination and cultured on Sabouraud Dextrose Agar with Chloramphenicol as well as CHROMAgar *Candida*. Definitive identification of fungal isolate was made by molecular methods. Our investigations are still ongoing.

**Results:** Out of 75 clinically suspected patients, 59 (78.7%) cases were confirmed after mycological investigation. 65 fungal strains isolated from these cases counting multiple fungal species infections. The most common symptoms were otalgia (52%) followed by itching (49.33%), the fullness of the ear (43.42%), hearing loss (17.11%), and tinnitus (13.16%). The most prevalent fungal agent was *Aspergillus* section *nigri* (24; 32%) followed by *Candida* spp. (14; 18.66%), *Aspergillus* section *Flavi* (11; 14.66%), *Aspergillus* section *Fumigati* (8; 10.66%), *Aspergillus* section *Nidulantes* (2; 2.66%), and *Mucor* spp. and *Alternaria* spp. (both 1; 1.33%). The Polymorphism of the Internal transcribed spacer region for Candida isolates was analyzed with the restriction enzyme *Msp1*. Accordingly, *Candida parapsilosis* was isolated in 50% (7) of cases, followed by *C. albicans* (5; 35.71%), and *C. krusei* (2, 14.28%).

**Conclusions:** Consistent with previous studies, the most eminent isolated fungi were *Aspergillus* section *nigri* and *C. parapsilosis* complex among filamentous and yeast fungi, respectively, the impact of subtropical climate in the geographical region of Guilan could not be neglected in the increasing trend in otomycosis prevalence. However, the species of etiological agent seems to be independent of the regional climate as *C. parapsilosis* was reported as the most prevalent agent in dried climates of Iran. It might be suggested that the ability of *C. parapsilosis* in adhesion to epithelial cells and surfaces could affect the rate of occurrence as well as recurrence in otomycosis. Species identification by appropriate laboratory diagnosis would definitely be useful for accurate and successful treatment.

## P162 Increasing Incidence of Candidemia and Changing Trend towards non-*albicans Candida* Species: Results from a 10-Year Nationwide Study in Greece

**Georgia Vrioni ^1^, Vassiliki Mamali ^2^, Maria Siopi ^3^, Stefanos Charpantidis ^4^, Vassiliki Baka ^5^, Stavroula Baka ^6^, Theodora Biniari ^7^, Nikoletta Charalampaki ^8^, Athanasios Chatzimoschou ^9^, Athanasia Christidou ^10^, Myrto Christofidou ^11^, Genovefa Chronopoulou ^12^, Ioannis Deliolanis ^13^, Ioannis Dendrinos ^14^, Maria Dimitriou ^15^, Maria Drogari-Apiranthitou ^16^, George Ganteris ^17^, Konstantina Gartzonika ^18^, Panagiota Giannopoulou ^8^, Eirini Glynou ^4^, Helen Kafkoula ^5^, Stefanos Karachalios ^7^, Stergios Karapsias ^19^, Paraskevi Karle ^20^, Anna Katsiaflaka ^21^, Helen Koiliari ^22^, Eirini Lamprou ^15^, Paraskevi Mantzana ^23^, Sofia Maraki ^10^, Fani Markou ^24^, Maria Martsoukou ^25^, Joseph Meletiadis ^3^, Chrysi Michailidou ^26^, Alexandra Mpakosi ^20^, Martha Nepka ^27^, Maria Orfanidou ^17^, Zoi-Dorothea Pana ^28^, Maria Panopoulou ^29^, Angeliki Pantazatou ^13^, Kalliopi Panteli ^30^, Virginia Papaemmanouil ^15^, Vassiliki Papaioannou ^22^, Efstathia Perivolioti ^27^, Evangelia Platsouka ^31^, Aggeliki Poulou ^24^, Spyros Pournaras ^3^, Efthalia Priavali ^18^, Emmanuel Roilides ^28^, Christina Sereti ^8^, Vassiliki Skandami ^32^, Nikoletta Skarmoutsou ^25^, Anna Skiada ^33^, Lemonia Skoura ^23^, Anastasia Spiliopoulou ^11^, Katina Themeli-Digalaki ^2^, Anastasios Tsakalos ^17^, Sofia Tsiplakou ^22^, Athanasia Tsiringa ^32^, Helen Vagdatli ^26^, Helen Vagiakou ^17^, Olga Vasilaki ^23^, Christina Vossou ^12^, Ioanna Voulgaridi ^21^, George Samonis ^34^ and Athanasios Tsakris ^1^**

^1^ Department of Microbiology, Medical School, National and Kapodistrian University of Athens
^2^ Department of Microbiology, Tzaneio General Hospital
^3^ Clinical Microbiology Laboratory, “Attikon” University General Hospital, Medical School, National and Kapodistrian University of Athens
^4^ Department of Microbiology, “Elena Venizelou” Maternity Hospital
^5^ Department of Microbiology, Korgialenio Benakio Hellenic Red Cross Hospital
^6^ Department of Microbiology, “Aretaieion” University Hospital, National and Kapodistrian University of Athens
^7^ Department of Microbiology, “Agioi Anargyroi” General Oncology Hospital
^8^ Department of Microbiology, Thriassio General Hospital
^9^ Laboratory of Infectious Diseases, 3rd Department of Pediatrics, Aristotle University School of Health Sciences, Hippokration General Hospital
^10^ Department of Microbiology, University Hospital of Heraklion, Heraklion
^11^ Department of Microbiology, University Hospital of Patras
^12^ Department of Microbiology, Euroclinic Hospital
^13^ Department of Microbiology, Laikon General Hospital
^14^ Department of Microbiology, Metropolitan Hospital
^15^ Department of Microbiology, “Metaxa” Anticancer Hospital
^16^ 4th Department of Internal Medicine, “Attikon” University General Hospital, National and Kapodistrian University of Athens
^17^ Department of Microbiology, “G. Gennimatas” General Hospital
^18^ Department of Microbiology, University Hospital of Ioannina
^19^ Clinical Department of Microbiology, 251 Air Force General Hospital
^20^ Department of Microbiology, General Hospital of Nikaia “Agios Panteleimon”
^21^ Department of Microbiology, University Hospital of Larissa
^22^ Department of Microbiology, KAT General Hospital
^23^ Department of Microbiology, AHEPA University Hospital, Aristotle University of Thessaloniki
^24^ Department of Microbiology, General Hospital of Serres
^25^ Department of Microbiology, Sismanogleio General Hospital
^26^ Department of Microbiology, Hippokration General Hospital
^27^ Department of Microbiology, Evaggelismos General Hospital
^28^ 3rd Department of Paediatrics, Infectious Diseases Unit, Aristotle University School of Medicine, Hippokration General Hospital
^29^ Laboratory of Microbiology, School of Medicine, Democritus University of Thrace
^30^ Department of Microbiology, Asklepieion Voulas General Hospital
^31^ Department of Microbiology, Konstantopouleio-Patission General Hospital
^32^ Department of Microbiology, Hippokration Athens General Hospital
^33^ 1st Department of Medicine, Laiko Hospital, School of Medicine, National and Kapodistrian University of Athens
^34^ Department of Internal Medicine, Univerity of Crete School of Medicine, Heraklion

**Objectives:** Geographic variations in the epidemiology of candidemia highlight the need for monitoring local trends, in order to guide appropriate empiric antifungal therapy and thus reduce mortality. Reported epidemiological data regarding the general population of Greece are scarce. Therefore, we conducted a nationwide survey to describe the epidemiologic characteristics of *Candida* spp. isolated from hospitalized patients with bloodstream infections during 2009–2018, with a view to evaluate epidemic trends and provide evidence for empirical treatment regimens.

**Materials & Methods:** All microbiologically confirmed candidemia cases in patients hospitalized in 28 centers across Greece during 2009–2018 were analysed. Ward of hospitalization at the time of the diagnosis, species identification and in vitro antifungal susceptibility profile (where available) were recorded. Identification of *Candida* isolates and susceptibility testing were performed as per hospital protocol. Annual incidence rates/100,000 inhabitants were calculated. Chi-square test for trend was used for evaluation of changes in species distribution.

**Results:** A total of 6.057 candidemic episodes/patients were analysed. In 3% of cases a mixed *Candida* spp. infection was diagnosed. Cases were reported in 44% from internal medicine wards, 33% intensive care units and 23% surgical wards. The overall incidence of candidemia was 5,56/100.000 inhabitants with a significant increase over the years (3.75, 5.83, and 7.01/100.000 inhabitants in 2009–2011, 2012–2014, and 2015–2018, respectively; *p* = 0.0002). *C. parapsilosis* species complex (SC) was the predominant species (41%), followed by *C. albicans* (37%), *C. glabrata* SC (10%), *C. tropicalis* (7%), *C. krusei* (1%), and other rare *Candida* spp. (4%). *C. albicans* rates decreased from 2009 to 2018 (48% to 31%) in parallel with a doubling of *C. parapsilosis* SC rates (28% to 49%, *p* < 0.0001) (Figure). The majority of pathogens was susceptible/wild-type to the antifungals tested. No resistance to amphotericin B and flucytosine was found. Fluconazole resistance was detected in 418/2101 (20%) of *C. parapsilosis* SC isolates (15/418 pan-azole-resistant) with no trend in resistance over time. Echinocandin resistance was found in 10/203 (5%) of *C. glabrata* SC isolates (7/10 pan-echinocandin-resistant) (Table).

**Conclusions:** This is the first multicentre nationwide candidemia study in Greece demonstrating an increasing incidence of candidemia with a shift towards non-*albicans Candida* spp., in particular *C. parapsilosis* SC, as the leading causative agent. Although antifungal resistance rates remain relatively low in the total sample, fluconazole-resistant *C. parapsilosis* SC raises concern.

## P163 Prospective Surveillance (2005–2018) of Invasive Fusariosis in France: Species Level Identification, Mics and Association to Clinical Findings

**Dea Garcia-Hermoso, Karine Sitbon, Stephane Bretagne, Francoise Dromer and The French Mycoses Study Group**

Institut Pasteur, CNRS, Unité de Mycologie Moléculaire, Centre National de Référence Mycoses Invasives et Antifongiques (NRCMA), UMR2000

**Objectives:** Physiopathology of fusariosis is often restricted to a specific patient population preventing any correlation between fungal species and clinical presentation. Here we analyzed fusariosis cases prospectively collected over 14 years (2005–2018) with precise polyphasic identification of the isolates and taking into account the most recent nomenclature.

**Methods:** More than 500 cases of probable and proven fusariosis with isolates sent to the NRCMA were collected between 2005 and 2018 through the nationwide network of French university hospitals (RESSIF). Cases were classified as proven or probable ocular fusariosis (OF), or invasive fusariosis (IF) according to the EORTC/MSGERC definitions. The possible cases were excluded and onyxes were not captured. Isolates were sent to the CNRMA where they were characterized phenotypically by morphological methods and molecularly using a multi-locus sequence analysis of translation elongation factor 1-α (TEF-1α) and of RNA-dependent polymerase subunit II (Rpb2). In addition, antifungal susceptibility testing of 8 antifungal drugs was performed using EUCAST microdilution method.

**Results:** Multilocus sequencing was performed on 505 isolates.The most represented complex was *Fusarium solani* (FSSC) with more than 200 strains (40.3%) from which 60% were identified to the species level resulting in seven different species. *Fusarium fujkuroi* species complex (FFSC) (26.4%) was represented by eight different species with *Fusarium proliferatum* as the most denoted species, while the126 strains (25%) belonging to the *Fusarium oxysporum* species complex (FOSC) were mostly represented by *Fusarium veterinarium* (clade FOSC VII). We identified strains which belonged to additional species complexes such as *F. incarnatum*-*equiseti* species complex (n = 5), *F. redolens* species complex (n = 3), *F. sporotrichoides*. and *F. nisikadoi* with one isolate each. Finally, *Fusarium dimerum* species complex was represented by *Fusarium (Bisifusarium) dimerum* and *B. delphinioides*. The MIC profiles to azoles, candins and amphotericin B were similar for the *Fusarium solani, oxysporum* and *fujikuroi* complexes except for the strains of *Bisifusarium dimerum* and *B. delphinoides* which had lower MIC values to amphotericin B.

Preliminary analysis showed that, for the three main complexes, the repartition was different with less FFSC in Ocular Fusariosis than in Invasive Fusariosis and among the latter, more FFSC in immunocompromised patients. Further studies are needed to explore these differences.

## P164 Shift in Mucormycosis Epidemiology in France: A Cohort Study from 2012 to 2018

**Laura Gouzien ^1^, Dea Garcia-Hermoso ^1^, Karine Sitbon ^1^, Françoise Dromer ^1^, Julien Durand ^2^, Didier Che ^2^ and Fanny Lanternier ^1^**

^1^ Molecular Mycology Unit, Institut Pasteur, CNRS, UMR 2000, French National Reference Center for Invasive Mycoses and Antifungals
^2^ Santé Publique France

**Objectives:** Mucormycosis is a rare but severe emerging fungal infection. At risk population includes immunocomprised patients, patients with *diabetes mellitus*, but also immunocompetents with trauma or burns. Its characteristics evolved over the last decade, as at risk population widened, guidelines for the management of immunocompromised patients were updated, and PCR-based diagnosis became available in 2015. The incidence of mucormycosis has been reported to increase in some countries, possibly in line with these changes.

Here, we aim to describe the epidemiology and outcomes of mucormycosis in France between 2012 and 2018, and to evaluate the risk factors for death.

**Materials & Methods:** Data included all proven, probable and putative mucormycosis cases from the RESeau de Surveillance des Infections Fongiques (RESSIF) database, recorded from 2012 to 2018 by the French National Reference Center for invasive Mycoses & Antifungals (NRCMA). RESSIF is a surveillance network including 29 French volunteer centers, which prospectively collect standardized epidemiological and mycological data regarding all unusual invasive fungal infections.

The 2020 European Organisation for Research and Treatment of Cancer/Mycoses Study Group definitions were applied for proven and probable cases. Putative cases were defined by a PCR-positive BAL or serum sample in patients presenting with clinical and radiological abnormalities compatible with the diagnosis. Risk factor for death at day 90 were analysed using logistic regression models.

**Results:** A total of 313 cases (145 proven, 113 probable, 55 putatives) were included. Haematological malignancies represented a majority of the main underlying diseases (194/313, 62%), followed by diabetes (8.6%), trauma and solid organ transplant (24/313, 7.7% each), and burns (21/313, 6.7%). Site of infection were mostly lungs (144/313, 46%), then rhinocerebral (58/313, 18.5%), cutanéo-articular (57/313, 18.2%) and disseminated (35/313, 11.2%). Fungal species were only available in 205 cases, with a majority of *Rhizopus arrhizus* (41/205, 20%), followed by *Lichtheimia corymbifera* (30/205, 14.6%), *Lichtheimia ramosa* (28/205, 13.6%), *Mucor circinelloides* and *Rhizomucor pusillus* (27/205, 10.2% each) and *Rhizopus microsporus* (21/205, 10.2%). PCR was positive in samples from 172 patients (55%).

Mortality at day 90 was 59%, with a median survival time of 39 days. The risk of death was increased by age (OR = 1.02 [1.00–1.03] per year), haematological malignancies (OR 2.74 [1.70–4.47]), diagnosis in intensive care unit (ICU) (OR 2.92 [1.73–5.08]), and diagnosis before 2015 (OR 1.88 [1.13–3.18]), but reduced in cutaneo-articular compared to disseminated (OR 0.17 [0.06–0.43]) infection in univariate analysis. All results remained statistically significant in multivariate analysis. In a sensitivity analysis with a landmark of one day on 259 cases, surgery reduced mortality in univariate (OR 0.23 [0.12–0.41], *p* < 0.0001) and multivariate.

**Conclusions:** We showed an increase in mortality compared to prior studies in France, despite the improvements made in diagnosis and treatment. It can be linked to the increased proportion of haematological malignancies as main underlying diseases, confirming a shift in at risk population over the last decade. Mucormycosis remains highly lethal, with a prognosis worsened by age, haematological malignancies and diagnosis while in ICU, but improved by surgery, and maybe early detection by PCR.

## P165 The Impact of the COVID-19 Pandemic on the Consumption of Antifungals in a Tertiary Greek Hospital

**Antonios Markogiannakis ^1^, Athanasios Kakasis ^2^, Nikolaos Pantazis ^3^, Nikolaos Sipsas ^2^ and Maria Gamaletsou ^2^**

^1^ Pharmacy Department, “Laiko” Athens General Hospital
^2^ Pathophysiology Department, Medical School, National and Kapodistrian University of Athens
^3^ Department of Hygiene, Epidemiology and Medical Statistics, Medical School, National and Kapodistrian University of Athens

**Objectives:** Our purpose was to determine the impact of COVID-19 pandemic on the consumption of in-hospital intravenous antifungal agents (IHIVAFs) in the tertiary hospital of Athens “Laiko”.

**Methods and Materials:** The monthly consumption data of IHIVAFs, expressed in DDDs/100 occupied bed days (OBD) were calculated for all eight IHIVAFs used during the first year of pandemic (March 2020 to February 2021, COVID-19 period-CP), in comparison to the corresponding preceding one-year period (March 2019 to February 2020, pre COVID-19 period-PCP) using an open-source software (AMC Tool, vers.1.9.0). Additional indices such as the total number of OBD, and the total number of patients, for all the clinics and separately for the five clinics with special populations such as hematologic or renal transplant patients (3 internal medicine departments, 1 hematologic department and 1 renal transplant unit) which usually demonstrate a high consumption of IHIVAFs, were also taken into account. Statistical analysis was performed using time-series analysis models. The differences in average IHIVAFs usage between the two periods were assessed through regression with Newey-West standard errors allowing for autocorrelated and heteroscedastic errors. All analyses were performed using Stata version 15.1 (Stata Corp., College Station, TX, USA). P-values less than 0.05 were considered as indicating statistical significance.

**Results:** A statistically significant reduction in IHIVAFs consumption was observed in five of eight agents and in particular for Liposomal Amphotericin B (L-AB) (*p* = 0.032), fluconazole (*p* = 0.048), voriconazole (*p* = 0.01), caspofungin (*p* < 0.001) and mycafungin (*p* = 0.003). These significant decreases were more evident in months with exaggerated COVID-19 outbreaks e.g., during March–April 2020 or January–February 2021 (Figure 1). Considerably, only for isavuconazole, a statistically significant increase in consumption was noticed during the CP (*p* = 0.02), possibly because of its use in COVID-19 patients. Overall there was a 16.3% reduction in the use of IHIVAFs during the CP and it was considered statistically very significant (*p* < 0.001; Figure 2). The total number of patient days and the total number of patients admitted to all clinics was slightly reduced between the two periods (157.627 vs. 142.318, 9.7% decrease and 26.356 vs. 22.129, 16% decrease), while it was more evident for the five clinics with special patient populations: 42.234 vs. 34.562 (18.2% decrease) regarding to the patient days and 8.984 vs. 7.086 (21.1% decrease) regarding to the number of patients.

**Conclusions:** The COVID-19 pandemic seems to have a considerable impact regarding to the consumption of IHIVAFs in our hospital, particularly for the most of those agents that are been used for empirical or pre-emptive treatment of invasive fungal infections. The reduction in the consumption of these agents was more apparent in the periods with the higher incidence of COVID-19 cases. These findings could be due to the reduced number of hospitalized patients and especially due to the low number of admissions in the hematologic and renal transplant units. This fact is highlighted by the decrease-during the pandemic period—in the total number of OBD and in the number of admitted patients, especially to those clinics with sensitive patient populations with a high consumption of IHIVAFs.

## P166 Blastomycosis—Risk Factors for Severe Disease and Mortality

**Timothy O’Dowd, Jack McHugh, Mark Enzler and Paschalis Vergidis**

Mayo Clinic

**Objectives:** *Blastomyces dermatitidis* and the more recently described *Blastomyces gilchristii* are thermally dimorphic fungi. An increase in the incidence of blastomycosis has been reported in recent years possibly related to environmental changes. We sought to examine risk factors for severe disease and mortality.

**Methods:** We performed a retrospective review study of patients diagnosed with blastomycosis from 1 January 2004 through 31 March 2020. Disease was defined in patients with clinical and/or radiographic evidence of infection and one or more of the following: (i) Isolation of *Blastomyces* in culture of respiratory, skin or sterile specimens, (ii) histopathologic or cytopathologic evidence of yeast consistent with *Blastomyces*, (iii) positive serology, (iv) positive urine or serum *Blastomyces* antigen, or (v) *Blastomyces* PCR.

Logistic regression was used to evaluate risk factors for severe disease. A Cox proportional hazards model was constructed to assess mortality. Variables with *p*-values < 0.20 were included in the multivariate model. Statistical analysis was performed using STATA.

**Results:** We identified 210 patients during the study period. Mean age was 51 years (range, 6–84). Most were male (71%). The diagnosis was confirmed by culture of respiratory specimens in 62%. Skin/soft tissue involvement was confirmed by culture in 11%. The most common radiographic finding was presence of pulmonary nodule or lung mass. In terms of antifungal therapy, 38.9% (81/208) of patients received induction treatment with a formulation of amphotericin B. Itraconazole and voriconazole were the most commonly used azoles.

In this cohort, 40.5% of patients were treated as outpatients (mild disease), 37.6% were admitted to the hospital but not in the ICU (moderate disease), and 21.9% required ICU admission (severe disease). 14.8% patients required mechanical ventilation and 1.4% required extracorporeal membrane oxygenation support. Older age, chronic obstructive pulmonary disease, diabetes mellitus, obesity (BMI ≥ 30), extrapulmonary disease and immunosuppressive therapy were not associated with severe disease. Independent risk factors for severe disease were lymphopenia (≤1.0 × 10^9^/L) and neutrophilia (≥7.5 × 10^9^/L) at the time of diagnosis.

All-cause 90-day mortality was 11.9%. Median time from diagnosis to death was 23 days (interquartile range, 8–31 days). The main cause of death was multiorgan failure. Independent risk factors for mortality were older age, neutrophilia, and lymphopenia, as shown in the Table. 90-day survival by age and lymphocyte count are shown in the Figures.

**Conclusions:** In previous studies, immunosuppression was associated with severe disease and increased mortality, a finding that was not confirmed in our cohort. As blastomycosis is a rare condition limited to certain geographic areas, it is difficult to make definitive conclusions on the impact of immunosuppression on disease outcomes. Unlike other endemic mycoses, blastomycosis may cause severe disease with respiratory failure in previously healthy individuals.

## P167 Molecular Reidentification of *Candida guilliermondii* and *Candida famata* Clinical Isolates from a Culture Collection

**Elena Palencia-Mulero, Aitor Jauregi-Urrutia, Estibaliz Mateo, Cristina Marcos-Arias, Esther Tamayo, Katherine Miranda-Cadena, Elena Eraso and Guilliermo Quindós**

Department of Inmunology, Microbiology and Parasitology, Faculty of Medicine and Nursery, University of the Basque Country (UPV/EHU)

**Introduction and Objective:** Candidiasis caused by *Candida guilliermondii* complex are of clinical interest given conventional phenotypic diagnostic methods may misidentify these species. Therefore, their prevalence may have been underestimated. The aim of the study was to reidentify clinical isolates from a culture collection that were previously identified as *C. guilliermondii* and *Candida famata* by conventional phenotypic methods.

**Materials & Methods:** The study included 84 clinical isolates from individual patients collected between 1993 and 2018 and stored in the yeast stock collection of the Medical Mycology Laboratory at the University of the Basque Country (UPV/EHU). These isolates were originally identified by Chromagar *Candida* (Becton-Dickinson, Franklin Lakes, NJ, USA) or *Candida* Chromogenic Agar (Condalab, Madrid, Spain), and by API ID 32C or API 20C AUX (bioMérieux, Craponne, France) identification systems. All isolates were reidentified by sequencing of the ITS ribosomal DNA, and in some of them (n = 41) ACT1 gene sequencing was performed. Sequences were assembled manually and subjected to BLAST analysis to find similarities to sequences deposited at GenBank and Mycobank databases.

**Results:** Isolates were phenotypically identified as *C. guilliermondii* (n = 55) or *C. famata* (n = 2), while for the remaining 27 isolates it was not possible to determine the species.

By sequencing of ITS region, 83 isolates were classified into the *C. guilliermondii* species complex (78 as *C. guilliermondii*, four as *C. fermentati*, and one isolate as *Candida neustonensis*) and the remaining one was included into the *Candida famata* complex. All the sequences showed a homology ranging from 98 to 100% comparing with the sequences from BLAST databases, except five sequences of *C. guilliermondii* that yield lower homologies ranging from 92 to 97%. ACT1 gene sequencing confirmed the analysis of ITS region, the 41 isolates analyzed were classified as *C. guilliermondii* (n = 40), and *C. fermentati* (n = 1) with a homology ranging from 98 to 100%, except three sequences of *C. guilliermondii* that yield lower homologies but not lesser than 97%.

The four *C. fermentati* isolates, phenotipically identified as *C. guilliermondii*, were isolated from superficial candidiasis (n = 3) and from a bronchoaspirate (n = 1). *C. neustonensis*, also phenotipically identified as *C. guilliermondii*, was isolated from an infant’s oral cavity and, to date, this is the first isolation described in humans. Among the two isolates phenotipically identified as *C. famata*, one was molecularly identified as *C. guilliermondii* and the other one was classified within the *C. famata* complex, but the species could not be determined due to the very low interspecies variation between *Debaryomyces fabryi* and *Debaryomyces prosopidis*.

**Conclusions:** The use of molecular methods improved the conventional identification of *C. guilliermondii*, but the low interspecies variation requires the analysis of more loci in addition to the ITS region for the discrimination between species within the complexes. Molecular identification allowed us to report, to our knowledge, the first isolation of *C. neustonensis* from a person.

**Funding:** GIC15/78 IT-990-16 (Gobierno Vasco-Eusko Jaurlaritza).

## P168 COVID-19 Associated Pulmonary Aspergillosis (CAPA): Hospital or Home Environment as a Source of Life-Threatening *Aspergillus fumigatus* Infection?

**Irene Gonzalez-Jimenez ^3^, Alejandra Roldan ^3^, Andrea Garcia-Fernandez ^1^, Lorena Forcelledo ^1,2^, Jose Luis Carretero ^1^, Belen Rivaya ^1^, Blanca Leoz-Gordillo ^1^, Rodrigo Albillos ^1^, Mar Martinez-Suarez ^1,2^, Fernando Vazquez ^1,2^, Santiago Melon ^1,2^, Maria Luisa Sanchez ^1^, Emilia Mellado ^3^ and Teresa Pelaez ^1,2^**

^1^ Hospital Universitario Central de Asturias (Servicio de Microbiología, Servicio de Medicina Intensiva, Servicio de Medicina Preventiva)
^2^ Fundación para la Investigación Biosanitaria del Principado de Asturias (FINBA)
^3^ Instituto de Salud Carlos III

**Objectives:** Most cases of invasive aspergillosis are caused by *Aspergillus fumigatus*, whose conidia are ubiquitous in the environment. Additionally, in indoor environments, such as houses or hospitals, conidia are frequently acknowledged too. Hospital-acquired aspergillosis is usually associated with airborne fungal contamination of the hospital air, especially after building construction events. *A. fumigatus* strain typing can fulfill many needs both in clinical settings and otherwise. The high incidence of aspergillosis in COVID patients from our hospital, made us wonder if they were hospital-acquired aspergillosis. The purpose of this study was to evaluate whether the hospital environment was the source of aspergillosis infection in CAPA patients, admitted to the Hospital Universitario Central de Asturias, during the first and second wave of the COVID-19 pandemic, or whether it was community-acquired aspergillosis prior to admission.

**Materials & Methods:** During 2020, sixty-nine *A. fumigatus* strains were collected for this study: 59 were clinical isolates from 46 COVID-19 patients and 10 strains were environmentally isolated from 8 hospital rooms and intensive care units. A diagnosis of pulmonary aspergillosis was based on: positive culture, galactomannan antigen (index 5–10 in respiratory samples), positive PCR in respiratory samples (CT < 36), positive LFD in respiratory samples, and/or β-D-glucan on blood, and clinical suspicion with presence of bilateral pneumonia compatible with aspergillosis on chest computed tomography image and/or X-ray images. Strains were genotyped by PCR amplification and sequencing of a panel of four hypervariable tandem repeats within exons of surface protein coding genes (TRESPERG) (Simpson index (D) = 0.9972). For each strain a type was obtained according to the composition and number of repeats for each gene and a final genotype for each strain was assigned combining the types obtained with the four genes.

**Results:** A total of seven genotypes among the 10 environmental strains and 32 genotypes among the 59 clinical strains were identified. Genotyping revealed that one *A. fumigatus* strain from UCI 5 (box 54) (1 cfu/m^3^) isolated in October (30 October 2020) and one *A. fumigatus* isolated from a COVID-19 patient admitted in Pneumology (Room 532-B) in November (24 November 2020) had the same genotype. Only two patients, one from March (30 March 2020) in the Cardiac ICU (Box 10) and another from November (13 November 2020) in the general ICU (Box-91) shared the same genotype. There was no coincidence in time or space between the two matched genotypes between patients, nor between hospital air and patients.

**Conclusions:** To our knowledge, this is the first study monitoring and genotyping *A. fumigatus* isolates from hospital air and COVID-19 patients admitted with aspergillosis obtained during one year. *A. fumigatus* strains showed a wide diversity of genotypes. Our work shows that patients did not acquire *A. fumigatus* in the hospital. This proves that COVID-associated aspergillosis in our hospital is not a nosocomial infection, but supports the hypothesis of “community aspergillosis” acquisition outside the hospital, having the home environment (pandemic period at home) as the main suspected focus of infection.

## P169 Invasive Aspergillosis Co-Infection Associated with SARS-CoV-2: Experience in a Tertiary University Hospital during a Pandemic Year

**Lorena Forcelledo ^1,2^, Blanca Leoz-Gordillo ^1^, Rodrigo Albillos ^1^, Irene Gonzalez ^3^, Alejandra Roldan ^3^, Guillermo Albacieta ^1,2^, Santiago Melon ^1,2^, Fernando Vazquez ^1,2^, Maria Luisa Sanchez ^1,2^, Emilia Mellado ^3^ and Teresa Pelaez ^1,2^**

^1^ Hospital Universitario Central de Asturias (Servicio de Microbiología, Servicio de Medicina Intensiva, Servicio de Medicina Preventiva)
^2^ Fundación para la Investigación Biosanitaria del Principado de Asturias (FINBA)
^3^ Instituto de Salud Carlos III

**Objectives:** Coronavirus disease-19 (COVID-19) has emerged as an important disease that predisposes patients to pulmonary aspergillosis (CAPA). Diagnosis of CAPA remains challenging and the incidence of CAPA varies between hospitals and between countries. Our study describes the incidence of CAPA among patients admitted at intensive care units (ICU) in a tertiary university hospital (1000-bed) in Spain, during the 2020 pandemic year.

**Materials & Methods:** A study was conducted among 300 patients tested for COVID-19 infection who were admitted to the hospital at ICU during the first and second waves (March–May 2020 and October–December 2020). COVID-19 diagnosis was performed by in-house quantitative PCR expressed in copies/10^3^ cells. The laboratory was proactive (high mycological suspicion) and all respiratory samples from patients admitted to the ICU were processed. A diagnosis of pulmonary aspergillosis was based on ECCM/ISHAM criteria: positive culture, galactomannan antigen, PCR (CT < 36), and LFD in respiratory samples, β-D-glucan on blood, and clinical suspicion with presence of bilateral pneumonia compatible with aspergillosis on chest computed tomography and/or X-ray image. Clinical and laboratory data were collected from 35 patients, antifungal and viral therapies; underlying conditions; use of steroids and immunosuppressive drugs; hospitalization days and outcome.

**Results:** During the first wave, the incidence of patients with CAPA was 17.7% (11 pts) with a mortality of 10%. The *Aspergillus* species involved were as follows: *A. fumigatus* 60%, *A. terreus* 30%, and 10% of mixed infections (*A. fumigatus* + *A. nidulans*). During the second wave, the incidence of patients with CAPA was 10.1% (24 pts), with a mortality of 37.5%. The Aspergillus species involved were as follows: *A. fumigatus* 50%, *A. niger* 13%, *A. terreus* 8% and 25% of mixed infections with more than one *Aspergillus* species (17% *A. fumigatus* + *A. terreus*, 4% *A. fumigatus* + *A. niger*, 4% *A. fumigatus* + *A. terreus* + *A. niger*) and no growth in 4% of patients. Regarding the diagnostic tools, mycological culture, PCR, LFD and β-D-glucan (100%), and GM in respiratory specimen (87.5%) had high specificity and sensitivity. On the other hand, the sensitivity of GM in serum was only 16.7%. All *A. fumigatus* were azole susceptible. Two *A. terreus* and one *A. niger* showed azole resistance. The fact that all the patients in the first wave had *Aspergillus* positive PCR in the same sample as the COVID-19 diagnosis supports the co-infection of Sars-CoV-2 and *Aspergillus* since the first day of hospital admission.

**Conclusions:** In our hospital, there was a high incidence of patients with COVID-19 co-infection and aspergillosis, not only among immunocompromised patients but also immunocompetent patients. A large percentage of patients had a poor outcome even when antifungal therapies were administrated. We emphasize the importance to perform mycological screening to all patients entering the ICU with COVID-19. Finally, it is important to detect IA co-infection caused by different *Aspergillus* species, with different susceptibility profiles that can complicate antifungal therapy and clinical outcome.

## P170 Epidemiology of Mucormycosis in Greece; Results from a 15-Year Nationwide Survey

**Maria Drogari-Apiranthitou ^1^, Anna Skiada ^2^, Ioannis Pavleas ^3^, Elias Iosifidis ^4^, Myrto Christofidou ^5^, Emmanuel Roilides ^4^, George Petrikkos ^6,7^**

^1^ Infectious Diseases Research Laboratory, 4th Department of Internal Medicine, National and Kapodistrian University of Athens, Attikon General Hospital
^2^ 1st Department of Internal Medicine, National and Kapodistrian University of Athens, Laiko General Hospital
^3^ Intensive Care Unit, Laiko General Hospital
^4^ 3rd Department of Paediatrics, Aristotelion University of Thessaloniki
^5^ Department of Microbiology, University Hospital of Rion, Patras
^6^ National and Kapodistrian University of Athens
^7^ European University Cyprus

**Objectives:** Mucormycosis has emerged as an important infection in the past two decades, affecting mainly immunocompromised patients. The aim of the study was to analyse the epidemiological parameters of this potentially devastating infection, in a multicentric, nationwide survey in Greece.

**Materials & Methods:** The survey started in 2005 and is ongoing. Each case was recorded in a Case Report Form and sent to our departments together with the fungus, or the paraffin (FFPE) embedded tissue block whenever cultures were negative or not available. From 2008 onwards, the cases were submitted electronically, at www.zygomyco.net, which is the site of the ECMM/ISHAM Working Group on Zygomycosis. The collected data included demographic, clinical characteristics of the patients, risk factors, time and type of laboratory diagnosis, treatment, and outcome. Only proven and probable infections according to EORTC/MSG criteria were analysed. Laboratory diagnosis was based on histopathology, microbiology, and molecular methods. The incidence was calculated in the general population, based on the latest census (2011).

**Results:** There were 88 cases registered from January 2006 to December 2020. Male to female ratio was 1.3:1, with a median age of 57 years. The underlying diseases were haematologic malignancies/neutropenia and haematopoietic stem cell transplantation (HSCT) (37.5%), diabetes mellitus (18.2%), other immunodeficiencies (13.6%) (including autoimmune diseases/high dose corticosteroids, solid tumours, chronic renal failure, and solid organ transplantation), whereas 24% were cutaneous/soft tissue infections occurring in immunocompetent individuals after major trauma, burns, or surgery. Of the cutaneous infections 28.6% were healthcare-associated and two occurred after natural disasters. The most frequent form of infection was the rhino-cerebral (49%), followed by the cutaneous (31.8%), pulmonary (12.5%) and disseminated (6%). Almost all immunocompetent patients had the cutaneous form. *Rhizopus* (66%, mostly *R. arrhizus*) was the predominant genus followed by *Lichtheimia* (11.2%) and *Mucor* (4.2%). Other Mucorales were *Saksenaea vasiformis* (2 cases), *Apophysomyces elegans* and *Syncephalastrum racemosum* (1 case each) and 12.7% were unspecified Mucorales. Culture was negative in 8% of the cases. Histopathology was obtained from all cases. Treatment was multimodal, including antifungals (liposomal amphotericin B, posaconazole and isavuconazole); surgery in cases where this was possible, and reversal of underlying risk factors. Crude mortality rate was high (51%) and ranged between 30% (diabetics) to 64% (haematological patients). The incidence rate appears to be stable and the estimate is 6 cases/year or 0.05/100,000 population.

**Conclusions:** Our results show that mucormycosis in Greece follows the epidemiology of other European countries and has a stable rate. Increased knowledge and awareness may help preventing many cases especially in the immunocompetent and increase survival.

**Note:** The study was part of the ECMM/ISHAM Working Group of Zygomycosis registry (www.zygomyco.net)

## P171: Canine Dermatophytoses and Associated Risk Factors among Dogs in Osun and Kwara States, Nigeria

**Yemisi Adesiji ^1^, Daniel Oluwayelu ^2^ and Julius Aiyedun ^3^**

^1^ Department of Medical Microbiology and Parasitology
^2^ Department of Veterinary Microbiology, University of Ibadan
^3^ Department of Veterinary Public Health and Preventive Medicine, University of Ilorin

**Objectives:** This study was designed to investigate the occurrence and associated risk factors of dermatophytoses in dogs with dermatophytic skin lesions presented at Veterinary Clinics in Osun and Kwara States, Nigeria.

**Materials & Methods:** This prospective, cross-sectional study was carried out in Osogbo, Osun State and Ilorin, Kwara State in southwestern and north-central Nigeria, respectively. A total of 325 dogs with lesions suggestive of dermatophytosis were examined between July and November 2019. Plucked hairs and skin scrapings were emulsified in 10% potassium hydroxide and examined microscopically for fungal elements. All the samples were also cultured using standard mycological procedures. A structured questionnaire was administered to obtain information on the dogs’ demographic characteristics and possible risk factors for dermatophytosis from the animal owners and clinicians. The level of association between variables and occurrence of dermatophytoses was determined using Chi-square test and *p*-values ≤ 0.05 were considered significant.

**Results:** Our findings revealed that of the 325 dogs examined, 35 (10.8%) samples were positive for fungal elements by direct microscopy while 14.8% (48/325) yielded cultures positive for dermatophytes. Out of the 48 positive cultures obtained, 37.5% (n = 18) were identified as *M. canis*, 27.1% (n = 13) as *M. gypseum* and 8.3% (n = 4) as *T. mentagrophytes*. Others included *Aspergillus flavus* 12.5% (n = 6), and *Malassezia canis* 12.5% (n = 6). The age distribution of positive cases were <1 year (n = 24, 50.0%), 1–3 years (n = 14, 29.2%) and >3 years (n = 10, 20.8%). Risk factors associated with dermatophytosis included the dog breed (*p* = 0.006), sex (*p* = 0.023), history of dermatophytosis (*p* = 0.001), housing type (*p* = 0.001), sanitation (*p* = 0.003), and grooming (*p* = 0.012).

**Conclusions:** This study established the occurrence of dermatophytosis in dogs kept for companionship (i.e., pets), security and breeding purposes, and presented at veterinary clinics in two States located in two different geographical regions of Nigeria. Our findings underscore the need for routine mycological investigations in these dogs to facilitate early detection of cases and prompt institution of treatment interventions, thereby preventing zoonotic transmission of dermatophytes to their owners, handlers and veterinarians.

## P172 Epidemiological, Diagnosis and Management of Cryptococcosis Cases in Fann Teaching Hospital from 2005 to 2017 in Senegal

**Doudou Sow ^1,3^, Carole Pab Minlekib ^2^, Isaac A Manga ^2^, Mamadou Dia ^3^, Souleye Lelo ^2,3^, Cheikh Binetou Fall ^2^, Khadime Sylla ^2,3^, Magatte Ndiaye ^2^, Roger CK Tine ^2,3^, Babacar Faye ^2^, Thérèse Dieng ^2,3^**

^1^ Université Gaston Berger
^2^ Université Cheikh Anta Diop
^3^ Centre Hospitalier National Universitaire de Fann

**Background:** *Cryptococcal Meningitis* is one of the most important opportunistic infection and a major contributor to early mortality. In sub-Saharan Africa, particularly in Senegal, prevalence of *Cryptococcal Meningitis* remains high. This study aimed to describe the epidemiology, laboratory profile, therapeutic and outcome of cases diagnosed in Dakar.

**Methods:** We have analyzed the cryptococcosis cases diagnosed at the department of parasitology-mycology in Fann Teaching Hospital in Dakar from 2005 to 2017. The diagnosis was confirmed by culture on Sabouraud’s dextrose agar and/or by India ink preparation and/or by cryptococcal antigen detection. The diagnosis methods were assessed by using culture as reference. During a period of 6 months in 2017, the accuracy and the reliability of the Dynamiker Cryptococcal Antigen Lateral Flow Assay (LFA), a new antigen kit was assessed compared to the latex agglutination used in routine activities.

**Results:** Out of the 2425 patients screened, a total of 194 cases of *Cryptococcal Meningitis* were diagnosed. The prevalence of *Cryptococcal Meningitis* was 8%. The most infected patients were aged between 31–45 years old (42.2%). There were slightly more male (53.4%) than female (46.6%) patients; 92.3% of the patients were found to be infected with HIV. India ink staining was positive in 32 patients (16.4%) while the culture of the fungus in the cerebrospinal fluid was positive in 52 patients (26.8%). 120 patients (61.8%) were diagnosed using the cryptococcal antigen detection in cerebrospinal fluid. The performance of the Dynamiker Lateral Flow Assay yielded a sensitivity at 100%, a specificity at 75% and a Youden at 75 when it was compared to the Latex agglutination. The most frequently used antifungal drug was fluconazole (86.7%), and the mortality rate was 64%.

**Conclusions:** Early diagnosis is essential to control cryptococcosis, and countries should prioritize widespread and reliable access to rapid diagnostic cryptococcus antigen assays. But it is important to make available the antifungal drugs including the Amphotericin B and the Flucytosine to reduce the mortality rate.

**Keywords:** cryptococcal meningitis; epidemiology; laboratory profile; therapeutic outcome

## P173 A Cross-Sectional Study of Epidemiological Aspects of Mucormycosis in Educational Hospitals Affiliated to Shiraz University of Medical Sciences, Iran

**Marjan Motamedi**

Department of Medical Mycology & Parasitology, School of Medicine, Shiraz University of Medical Sciences

**Objectives:** Invasive diseases caused by the Mucorales fungi occur amongst patients with immunodeficiency and other predisposing factors. The clinical signs of Mucormycosis are reflections of infarction and necrosis of the host tissue, which is due to hypha attack on the arteries and pose a high mortality rate despite medical advances. Due to the differences in the epidemiological aspects of Mucormycosis, we sought to assess the epidemiological and clinical characteristics of patients suffering from Mucormycosis infection admitted to educational hospitals affiliated to Shiraz University of Medical Sciences, Shiraz, Iran.

**Materials and Methods:** Our goal population in this cross-sectional study were all patients suffering from Mucormycosis infection admitted to main referral centers for infectious diseases in Fars province (including Namazi, Shahid Faghihi, Khalili and Amir), during 1392 to 1399. Demographic, clinical and diagnostic data was obtained by reviewing and entering in a semi-structured data collection form from medical records.

**Results:** After de-duplication, a total of 164 patients (55.5 percent male, 44.5 percent female) with a mean age of 41.66 ± 22.91 years were included in the study. The highest number of hospitalizations was observed in autumn (31.7 percent) and the lowest in spring (15.9 percent). The most common type of invasion was rhino-cerebral with 74.4 percent abundance. The highest frequency of risk factors belonged to hematological malignancies and neutropenia (37.8 percent), followed by diabetes mellitus (37.3 percent). The mortality rate was 14.6 percent. There was a significant relationship between the risk factor and the location of infection (*p*-value < 0.001), and also a significant relationship between the administered therapy and the prognosis (*p*-value < 0.02).

**Conclusions:** Due to the high frequency of patients with hematologic malignancy, control measures and improvement of environmental and staff sanitary conditions in hematology departments should be considered to reduce the risk of mucormycosis infection. Moreover, treating neutropenia is warranted. In addition, control of diabetic ketoacidosis in diabetic patients should be on the agenda.

## P174 Genetic Variation and Population Structure Analysis of Microsporum Canis Using Microsatellite and Multilocus Typing

**Chioma Aneke ^1,2^, Adela Cmokova ^3,4^, Vit Hubka ^3,4^, Wafa Rhimi ^1^, Domenico Otranto ^1,5^ and Claudia Cafarchia ^1^**

^1^ University of Bari
^2^ Department of Veterinary Pathology and Microbiology, University of Nigeria
^3^ Department of Botany, Faculty of Science, Charles University
^4^ Laboratory of Fungal Genetics and Metabolism, Institute of Microbiology of the Czech Academy of Sciences
^5^ Faculty of Veterinary Sciences, Bu-Ali Sina University

**Objectives:** Molecular epidemiological studies on *Microsporum canis* are still rare because of lack of sufficiently variable DNA sequences and polymorphic markers. In this study, we were searching for variable DNA sequence and microsatellite markers.

**Methods:** A total of 65 *M. canis* strains isolated from humans, animals with and without lesions were selected subtyping by multilocus sequence typing (MLST) and multilocus microsatellite typing (MLMT). Firstly, we used a limited set of strains for screening of variability among 12 housekeeping genes and among available and newly detected microsatellite loci.

**Results:** We observed no intraspecific variability among 10 out of 12 housekeeping genes, and only ITS and IGS regions showed two and three sequence genotypes, respectively. However, one from these 12 loci were useful for differentiation between *M. canis, M. audoinii* and *M. ferrugineum*.

Eighteen microsatellite genotypes (A-R) were recognized using multilocus microsatellite typing (MLMT) based on 8 loci, allowing a subdivision of the strains into two populations following of Bayesian statistical approach. Genotype C was isolated from dogs, cats, man, whereas other genotypes were host specific.

**Conclusions:** The present study suggests that MLST is not useful for typing *M. canis* but ITS and IGS regions might be used to detect limited genetic variability. However, microsatellite-analysis is a powerful tool for subtyping and can be used for surveillance studies and for gaining insight into the epidemiology of infections due to *M. canis*.

## P175 Pneumocystis Jirovecii Pneumonia in Fann Teaching Hospital from 2009 to 2017 in Senegal

**Doudou Sow ^1,3^, Carole Pab Minlekib ^2^, Isaac A Manga ^2^, Mamadou Dia ^3^, Souleye Lelo ^2^, Cheikh Binetou Fall ^2^, Khadime Sylla ^2,3^, Magatte Ndiaye ^2^, Roger CK Tine ^2,3^, Babacar Faye ^2^ and Thérèse Dieng ^2,3^**

^1^ Université Gaston Berger
^2^ Université Cheikh Anta Diop
^3^ Centre Hospitalier National Universitaire de Fann

**Background:** Pneumocystis pneumonia (PcP) is caused by *Pneumocystis jirovecii*, an ascomycetous fungus that causes opportunistic pulmonary infections in immunocompromised patients such as human immunodeficiency virus (HIV) infection, haematological/solid malignancies, transplant recipients and receiving chronic immunosuppressive medications. In Sub-saharan Africa, data is lacking due to the low sensitivity of diagnostic tools in many countries. The objective of this study is to describe the frequency of pneumocystis pneumonia cases in Fann Teaching Hospital in Dakar.

**Materials and methods:** A descriptive longitudinal study was carried out from January 2009 to December 2017, in Fann Teaching Hospital in Dakar. The bronchoalveolar lavages received in the laboratory were examined microscopically for *Pneumocystis jirovecii* using indirect fluorescent assay and Giemsa staining.

**Results:** Out of the 477 patients screened for *Pneumocystis jirovecii*, 28 were positive yielding a frequency at 5.8%. Fourteen patients of the 28 infected (50%) were aged between 31 and 60 years old. There was more male (60.7%) than female (39.3%). The high number of diagnosed cases was found in 2011 (42.8%), in 2009 (21.4%) and in 2015 (17.8%). Seven patients (25%) were HIV positive. All the 28 patients were diagnosed using the indirect fluorescent assay.

**Conclusions:** Pneumocystis pneumonia remains a severe invasive fungal infection in immunocompromised patients. So, there is a need to improve diagnostic level including molecular testing for a better management of cases.

**Keywords:** pneumocystis pneumonia; bronchoalveolar lavage; indirect fluorescent assay; Giemsa

## P176 Isolation of *Rasamsonia argillacea* Species Complex in a Cystic Fibrosis Adult Patient—First Case in Portugal

**Dinah Carvalho ^1^, Raquel Sabino ^2,4^, Cristina Veríssimo ^2^, Helena Simões ^2^, Pilar Azevedo ^3^, Luis Marques Lito ^1^ and José Melo Cristino ^1^**

^1^ Centro Hospitalar Universitário Lisboa Norte
^2^ National Health Institute Dr. Ricardo Jorge, Reference Unit for Parasitic and Fungal Infections—Infectious Diseases Department
^3^ Centro Hospitalar Universitário Lisboa Norte—Cystic Fibrosis Reference Center
^4^ Instituto de Saúde Ambiental, Faculdade de Medicina, Universidade de Lisboa

**Objectives:** Cystic fibrosis (CF) is the most common monogenetic autosomal recessive disease in the human population. An important fungal biota has been described in respiratory secretions of patients suffering from CF being *Aspergillus fumigatus* and *Candida albicans* the most common fungi found. We report the isolation, for the first time in Portugal, of the emerging fungal pathogen *Rasamsonia argillacea* species complex, from a respiratory sample of an adult patient with CF.

**Material & Methods:** A 51-year-old male patient with heterozygous CF due to mutations ΔF508/P205S, is being followed in Cystic Fibrosis Reference Center for about 10 years. In the last years, he has been consistently colonized with Methicilin-susceptible *Staphylococcus aureus, Pseudomonas aeruginosa* and *Aspergillus* section *Fumigati* whereby is under chronic suppression therapy with two inhaled antibiotics. Recently, there has been a progressive clinical respiratory functional deterioration. In a periodic evaluation, a microbiology control sputum was requested. Sample was cultured, in parallel, for bacteriology and mycology evaluation.

**Results:** Apart from detection of *S. aureus* and *Raoultella ornithinolytica*, after 3–5 days of incubation at 37 °C, the cultures showed several cream-coloured colonies, powdery to velvety. Microscopic examination showed hyaline septate hyphae, *Penicillium*-like conidiophores with rough wall, ovoid to cylindrical phyalides with a narrow neck and cylindrical unicellular smooth-walled microconidia, arranged in unbranched basipetal chains arising from phialides.

The isolate was identified as *Rasamsonia argillacea* species complex based on its morphology and confirmed by MALDI-TOF mass spectrometry. As no septate hyphae were seen on direct examination, a new sample was requested to exclude extrinsic contamination. The second sample was inoculated as previously, confirming the persistent presence of *Rasamsonia argillacea* species complex in the sputum of this patient. The identification of this isolate was further confirmed by sequencing the internal transcribed spacer (ITS) region of ribosomal DNA, showing 100% homology with sequences deposited on databases. Antifungal susceptibility testing showed high minimal inhibitory concentrations (MIC) to almost all tested antifungals (posaconazole, voriconazole, amphotericin B) and low MIC to anidulafungin. The patient had no great exacerbation of his respiratory problems and the isolated fungus was interpreted as colonization being the patient under more frequent surveillance.

**Conclusions:** Although colonization of the upper respiratory tract in CF patients by *R. argillacea* species complex has been described as an emerging situation, the role of these fungi in clinical or functional deterioration of the disease remains controversial. Indeed, data about its real prevalence in the CF population are lacking. However, taking into account the ability of this species to predominantly affect the lungs, to induce pneumonia and to disseminate to adjacent organs or even to the central nervous system (CNS) in immunocompromised patients, it is essential to promote its accurate identification that is often misidentified as *Penicillium* spp. or *Paecilomyces* species. Antifungal susceptibility testing should be performed for epidemiological purposes and to guide therapy, as *Rasamsonia* spp. usually presents a marked antifungal resistance profile.

## P177 Epidemiology of Invasive Candidiasis in Hematological (Hem) and Non-Hematological (Non-Hem) Patients: Results of Prospective Multicenter Study in Russia

**Galina Klyasova ^1^, Anna Malchikova ^1^, Irina Molchanova ^2^, Olga Kutsevalova ^3^, Mihail Maschan ^4^, Antonina Vetokhina ^5^, Tatiyana Chernenkaya ^6^, Natalia Zvyozdkina ^7^, Oksana Khoreva ^8^, Larisa Krainova ^9^ and Svetlana Shushurina ^10^**

^1^ National Research Center For Hematology
^2^ Chelyabinsk Regional Clinical Hospital
^3^ Rostov Research Institute of Oncology
^4^ Dmitry Rogachev National Medical Research Center Of Pediatric Hematology, Oncology and Immunology
^5^ Irkutsk Regional Clinical Hospital
^6^ Sklifosovsky Moscow City Research Institute of Emergency Medicine
^7^ Regional Clinical Hospital
^8^ Surgut District Clinical Hospital
^9^ Novosibirsk Regional Clinical Hospital
^10^ Samara Regional Clinical Hospital named after V.D. Seredavin

**Background:** The aim of the study was to evaluate the etiology of invasive candidiasis in hem and non-hem patients (pts).

**Materials/methods:** The prospective multicenter study was performed in the period from 2005 to 2019 in 10 centers and 8 cities in Russia. *Candida* spp. isolates were collected from hem pts and non-hem pts (81.3% non-hem pts were in ICU) from blood and other sterile sites and were identified by matrix-assisted laser desorption ionization time-of-flight mass spectrometry (MALDI-TOF MS).

**Results:** During the 15-year study period, a total of 569 *Candida* spp. isolates were evaluated. *Candida* spp. were isolated from blood culture in 488 (85.8%) cases, from other sterile specimens in 81 (14.2%), from hem pts in 233 (40.9%), from non-hem pts in 336 (59.1%). Detection of *Candida* spp. from blood culture was predominated in hem pts compared to non-hem pts (94.4% vs. 79.8%, *p* < 0.001). Among non-hem pts *Candida* spp. were significantly more often isolated from peritoneal fluid (10.4% vs. 3.4%, *p* = 0.002) and cerebrospinal fluid (2.7% vs. 0%, *p* = 0.01) compared to other sities. The most common species were.

*C. albicans* (n = 229, 40.2%), followed by *C. parapsilosis* (n = 131, 23.0%), *C. glabrata* (n = 48, 8.4%), *C. krusei* (n = 42, 7.4%), *C. tropicalis* (n = 42, 7.4%), *C. guilliermondii* (n = 22, 3.9%), *C. pelliculosa* (n = 16, 2.8%), *C. lusitaniae* (n = 12, 2.1%), *C. auris* (n = 11, 1.9%), *C. kefyr* (n = 7, 1.2%), *C. lipolytica* (n = 4, 0.7%), *C. dubliniensis* (n = 2, 0.4%), *C. orthopsilosis * (n = 1, 0.2%), *C. methapsilosis* (n = 1, 0.2%), *C. fabianii* (n = 1, 0.2%). In total 11 *Candida* species were detected among hem pts and 14—in non-hem (Figure 1). *C. krusei* (11.6% vs. 4.5%, *p* = 0.002), *C. guilliermondii* (7.7% vs. 1.2%, *p* < 0.001) and *C. pelliculosa* (5.2% vs. 1.2%, *p* = 0.08) prevailed in hem pts compared to non-hem pts while *C. albicans* and *C. auris* were most frequently detected from non-hem pts (31.3% vs. 46.4%, *p* < 0.001 and 3.3% vs. 0%, *p* = 0.004, respectively). The species distribution varied by age and clinical specimens (Figure 2). Distribution of *Candida* species obtained from pts below 18 years of age were limited by 10 species compared to 15 species in pts older than 18 years; 14 *Candida* species were isolated from blood culture compared to 9—from other sterile sites.

**Conclusions:** The etiology of invasive candidiasis in hem and non-hem pts in Russia is characterized by a wide species diversity. The rate of *C. albicans* was less than 40.2%. The third most prevalent *Candida* species among hem pts was *C. krusei* (11.6%), among non-hem pts in ICU—*C. glabrata* (10.1%). *C. auris* was isolated only from non-hem pts in ICU. Among hem pts *Candida* spp. were isolated more commonly from blood culture (94.4%), but 10.4% of *Candida* species from non-hem pts were from peritoneal fluid ().

## P178 Trends of the Epidemiology of Candidemia in Switzerland: A 15-Year FUNGINOS Survey

**Kai-manuel Adam ^1^, Michael Osthoff ^1^, Frédéric Lamoth ^3,4^, Anna Conen ^5^, Véronique Erard ^6^, Katia Boggian ^7^, Peter W Schreiber ^8^, Stefan Zimmerli ^9,10^, Pierre-Yves Bochud ^3^, Dionysios Neofytos ^11^, Mapi Fleury ^12^, Hans Fankhauser ^13^, Daniel Goldenberger ^14^, Konrad Mühlethaler ^9,10^, Arnaud Riat ^15^, Reinhard Zbinden^16^, Andreas Kronenberg ^10^, Chantal Quiblier ^16^, Oscar Marchetti ^3,17^ and Nina Khanna ^1,2^**

^1^ Division of Infectious Diseases and Hospital Epidemiology, University Hospital of Basel
^2^ Department of Clinical Research
^3^ Infectious Diseases Service, Department of Medicine, Lausanne University Hospital and University of Lausanne
^4^ Institute of Microbiology, Lausanne University Hospital and University of Lausanne
^5^ Division of Infectious Diseases and Hospital Epidemiology, Cantonal Hospital of Aarau
^6^ Infectious Diseases Service, Department of Medicine, Cantonal Hospital
^7^ Division of Infectious Diseases and Hospital Epidemiology, Cantonal Hospital
^8^ Division of Infectious Diseases and Hospital Epidemiology, University Hospital of Zurich and University of Zurich
^9^ Department of Infectious Diseases, Bern University Hospital
^10^ Institute for Infectious Diseases, University of Bern
^11^ Infectious Diseases Service, University Hospital and University of Geneva
^12^ Department of Oncology, Lausanne University Hospital and University of Lausanne
^13^ Institute of Laboratory Medicine, Cantonal Hospital of Aarau
^14^ Clinical Bacteriology & Mycology, University Hospital of Basel and University of Basel
^15^ Division of Laboratory Medicine, Laboratory of Bacteriology, University Hospital of Geneva
^16^ Institute of Medical Microbiology, University of Zürich, Zürich
^17^ Department of Medicine, Ensemble Hospitalier de la Côte

**Objectives:** The increasing incidence of candidemia and emergence of drug resistant *Candida* (*C*.) species are major concerns worldwide. Long-term surveillance studies are needed.

**Materials & Methods:** The Fungal Infection Network of Switzerland (FUNGINOS) conducted a 15-year (2004–2018) nationwide epidemiological study of candidemia. Hospital-based incidence of candidemia, *Candida* species distribution, antifungal susceptibility and consumption were stratified in three periods (2004–2008, 2009–2013, 2014–2018). Population-based incidence over the period 2009–2018 derived from the Swiss Antibiotic Resistance Surveillance System (ANRESIS).

**Results:** A total of 2273 *Candida* blood isolates were studied. Population and hospital-based annual incidence of candidemia increased from 2.96 to 4.20/100,000 inhabitants (*p* = 0.022) and 0.86 to 0.99/10,000 patient-days (*p* = 0.124), respectively. The proportion of *C. albicans* decreased significantly from 60% to 53% (*p* = 0.0023), whereas *C. glabrata* increased from 18% to 27% (*p* < 0.0001). Other non-*albicans Candida* species remained stable. *C. glabrata* bloodstream infections occurred predominantly in the age group 18–40 and above 65 years. A higher proportional increase of *C. glabrata* was recorded in wards (18% to 29%, *p* < 0.0001) vs. intensive care units (19% to 24%, *p* = 0.22). According to CLSI, non-susceptibility to fluconazole in *C. albicans* was observed in 1% of isolates, and anidulafungin and micafungin non-susceptibility in 2% of *C. albicans* and *C. glabrata*. Fluconazole consumption, the most frequently used antifungal, remained stable, whereas use of mold-active triazoles and echinocandins increased significantly in the last decade (*p* < 0.0001).

**Conclusions:** Over the 15-year period, the incidence of candidemia increased. A species shift toward *C. glabrata* was recently observed, concurring with increased consumption of mold-active triazoles.

## P179 *Aspergillus fumigatus* Genotyping from CF Patient Isolates Demonstrates Possible Acquisition from the Home Environment

**Mireille van der Torre ^1^, Sara Gago ^2^, Lisa J. Collier ^1^, Andrew M. Jones ^1^, Malcolm D. Richardson ^1^ and Lily Novak Frazer ^1^**

^1^ MFT Wythenshawe Hospital
^2^ University of Manchester

**Objectives:** *Aspergillus fumigatus* is the most common filamentous fungus isolated from respiratory secretions from Cystic Fibrosis (CF) patients. Despite the high chronic colonisation rate and the contribution of allergic bronchopulmonary aspergillosis (ABPA) to disease progression in CF, little is known about the source of *A. fumigatus* in CF patients.

We compared A. fumigatus isolates from the homes of CF patients in the Greater Manchester municipal area and corresponding isolates produced from patient respiratory specimens using a sequence-based genotyping technique (TRESPERG) to determine whether the patients could acquire *A. fumigatus* from the environment of their own homes.

**Materials & Methods:** The A. fumigatus isolates collected from the homes and respiratory tract of 10 CF patients were characterised with a panel of four genes: cell surface protein A (CSP); MP-2 antigenic galactomannan protein (MP2); hypothetical protein with a CFEM domain (CFEM); and putative C-24 sterol reductase (ERG4B). The environmental samples were also sequenced to determine the presence of cyp51A-mediated azole resistance mechanisms.

**Results:** The time span between sampling the respiratory tract of patients and environmental sample collection was no longer than 8 weeks. Nineteen environmental isolates from 11 air samples collected from the bedrooms of CF patients with a corresponding respiratory tract *A. fumigatus* infection were identified as *A. fumigatus* through phenotypic and genotypic identification. The corresponding 10 clinical isolates were also confirmed as *A. fumigatus*; all were susceptible to the antifungal drugs itraconazole, voriconazole, posaconazole and amphotericin B. However, cyp51A sequencing revealed the environmental azole resistance mechanism ‘TR34/L98H’ in 2 of 19 (10.5%) environmental isolates.

TRESPERG genotyping revealed 6 CSP, 8 MP2, 7 CFEM and 8 ERG4B genotypes among the 29 A. fumigatus isolates including 1 CFEM and 2 MP2 novel genotypes (Table 1). The most common individual genotypes for CSP, CFEM, MP2 and ERG4B were t01 or t02 (31%), C08A (31%), m1.1 (65.5%) and e07 (37.9%), respectively (n = 12).

There was only one case (1 in 10) where the genotypes of the environmental and corresponding patient airway samples were identical for all four genes tested. However, we found two other patients whose clinical and environmental isolates shared 3 out of 4 markers, suggesting close genetic relatedness. Moreover, there were identical genotypes among clinical and environmental isolates but from different patients (Table 1).

**Conclusions:** This study suggests that acquisition of environmental *A. fumigatus* that could potentially become established clinically in CF patients is possible but may occur at low frequency; further sequencing is required to establish causality. This work suggests that preventive measures should focus on the living environment of vulnerable patients to avoid exposure. The use of this genotypic tool can identify quickly whether isolates are genetically distinct. Further analysis of the source of environmental acquisition is ongoing, including environmental testing of the CF centre.

## P180 Azole Resistance in *Aspergillus fumigatus*—Local Epidemiology in Skåne, Southern Sweden, and a Validation of the Four-Well Agar Plate

**Unn Tjörnstrand ^1^ and Karl Oldberg ^1,2^**

^1^ Clinical Microbiology, Region Skåne
^2^ Section for Infection Medicine, Lund University

**Objectives:** Azole resistance in *Aspergillus fumigatus* is a threat to effective antifungal therapy of invasive aspergillosis, and international guidelines recommend that the choice of agent for empirical treatment should be guided by local prevalence of resistance. Antimicrobial susceptibility testing (AST) with the reference method, broth microdilution (BMD), is often too demanding for most microbiological laboratories. The four-well azole agar plate (FWP) has previously been validated as a simpler method of screening for azole resistance: Three of the wells contain voriconazole, itraconazole and posaconazole, respectively, while the fourth is a growth control. Growth in any of the azole-containing wells indicates possible resistance. Our first objective was to validate the FWP as a routine method of AST on clinical isolates of *A. fumigatus* in our laboratory. Secondly, to evaluate the local prevalence of azole resistance in our region—Skåne in southern Sweden, with a population 1.38 million in 2020.

**Materials & Methods:** A commercially available FWP, VIPcheck™ (EWC Diagnostics, Steenwijk, The Netherlands) was used according to the instructions of the producer. The amount of growth was scored from 0 to 3.

Sensitivity, specificity and variation was investigated using a panel of 11 resistant strains and 11 sensitive strains that had been tested with BMD. This panel was tested twice, each time by two blinded individuals. The highest score for any azole well was registered.

The local prevalence of resistant isolates was studied by testing all clinical isolates of *A. fumigatus* in the Skåne region with the FWP during one year. Isolates exhibiting growth score ≥0.5 in an azole well were sent to Karolinska University Laboratory, Stockholm, Sweden, for BMD. Selected isolates (the first resistant or ambiguous isolate per patient) were sent to Statens Serum Institut, Copenhagen, Denmark, for sequencing of the *cyp51A* gene.

**Results:** All resistant isolates in the panel were identified, with a growth score of ≥1. In five cases a sensitive strain was given the score 0.5, giving a per test specificity of 77%.

During November 2018 to February 2020, 235 isolates from 157 patients were screened. 18 isolates were positive in the FWP (Table 1). Of these, ten isolates from three patients were resistant in BMD. Two patients had isolates with the mutation T_34_/L98H. The proportion of resistant isolates was 4.3% and the proportion of patients with resistant isolates was 1.9%.

The specificity in the screening study was 97% if isolates with growth score 0.5 were included as possibly resistant.

**Conclusions:** The sensitivity of the FWP is high, and it can be used to rule out azole resistance in the routine of microbiological laboratories. The distinction between no growth and weakest possible growth (0.5) can sometimes be difficult without experience, leading to reduced specificity. We believe this is why we classified more isolates to 0.5 in the early tests with the panel, compared with the later screening study.

We found a proportion of resistant isolates similar to what has been described in nearby Denmark. We see no need of exchanging azoles as the first line therapy for *Aspergillus* infections in our region.

## P181 Epidemiology of Candidemia in the Netherlands: An Update on Aetiology and Antifungal Susceptibility from a Nationwide Survey

**Renee Van Bentum ^1^, Claudy Oliveira dos Santos ^2,3,4^, Greetje Kampinga ^2^, Douwe Postma ^1^ and Paul Verweij ^4^**

^1^ Department of Internal Medicine, University Medical Centre Groningen
^2^ Department of Medical Microbiology, University Medical Centre Groningen
^3^ Laboratory of Clinical Microbiology and Infectious Diseases, Isala Hospital
^4^ Centre for Expertise in Mycology, Department of Medical Microbiology, Radboud University Medical Center

**Objectives:** Candidemia is the most frequent form of invasive candidiasis and results in high morbidity and mortality. Proper empirical treatment requires knowledge of the local incidence, species distribution, and susceptibility, yet the incidence of *Candida* species can differ per geographical region. Unfortunately, epidemiological data of *Candida* infections in the Netherlands is lacking after 2001. We use a retrospective cohort study of fungemia to gain knowledge of recent *Candida* species distribution and their susceptibility in the Netherlands.

**Methods:** Positive blood cultures with *Candida* species were retrieved from the Laboratory Information Systems of four tertiary care centres in different areas of the Netherlands (UMCG Groningen, AMC and VUMC Amsterdam, Radboud UMC Nijmegen) during the period 2005–2015. Only first isolate from episodes of candidemia were included. Identification and susceptibility testing was performed according to local procedures. Based on the MIC-distributions, the proportion of isolates outside the wildtype distribution was assessed.

**Results:** 984 isolates were retrieved from 744 patients. Incidence could not be calculated as the total amount of collected blood cultures or patient admissions was not known at this time. The most frequently found *Candida* species were *Candida albicans* (53.8%), *C. glabrata* (20.7%), *C. parapsilosis* (7.5%), *C. krusei* (4.8%), and *C. tropicalis* (4.7%). This distribution was relatively stable from 2005 to 2015 with the average percentage of non-*albicans* ranging from 44.8% from 2005–2009 to 45% from 2010–2015. 96.8% of the *C. albicans*, 83.6% of the *C. glabrata* and 92.5% of *C. parapsilosis* fell within the wildtype distribution range for fluconazole according to the EUCAST epidemiological cut-off (ECOFF) values. This was 100%, 98.6%, and 92.9% respectively for amfotericine B. For caspofungin, this was 98.9% for *C. albicans* and 87.1% for *C. glabrata*. For *C. parapsilosis* no formal EUCAST ECOFF value is available, with regards to caspofungin.

**Conclusions:** *C. albicans* remains the most common cause of candidemia in the Netherlands; the major subspecies of non-*albicans* candidemia are *C. glabrata* and *C. parapsilosis*. Throughout the follow-up period this remained relatively stable. Fluconazole resistance was rare. These data can be used in the development of Dutch treatment guidelines and choice of empirical treatment strategies. Future efforts will include updating the database to the present and combining microbiologic with clinical and pharmacologic data to obtain insights into current management and antifungal stewardship of candidemia in the Netherlands.

## P182 Surveillance of Azole-Resistant *Aspergillus fumigatus* Clinical Isolates in Greece: First Detection of TR34/L98H Alteration in cyp51A Associated with Pan-Azole Resistance

**Joseph Meletiadis ^1^, Maria Siopi ^1^, Olga Rivero-Menendez ^2^, Narda Medina ^2^, Athanasios Chatzimoschou ^3^, Angeliki Stathi ^4^, Helen Kirikou ^4^, Paraskevi Mantzana ^5^, Stavroula Antonopoulou ^6^, Eleni Vagiakou ^6^, Lemonia Skoura ^5^, Levantia Zachariadou ^4^, Aristea Velegraki ^7,8^, Georgia Vrioni ^8^, Emmanuel Roilides ^3^, Ana Alastruey-Izquierdo^2^, Spyros Pournaras ^1^ and Athanasios Tsakris ^8^**

^1^ Clinical Microbiology Laboratory, “Attikon” University General Hospital, Medical School, National and Kapodistrian University of Athens
^2^ National Centre for Microbiology, Instituto de Salud Carlos III, Mycology Reference Laboratory
^3^ Infectious Diseases Laboratory, 3rd Department of Pediatrics, “Hippokration” General Hospital, Faculty of Medicine, Aristotle University School of Health Sciences
^4^ Microbiology Department, “Aghia Sophia” Children’s Hospital
^5^ Microbiology Department, “AHEPA” University Hospital, Aristotle University School of Health Sciences
^6^ Microbiology Department, “G. Gennimatas” General Hospital
^7^ University of Athens/Hellenic Collection for Pathogenic Fungi (UOA/HCPF), Medical School, National and Kapodistrian University of Athens
^8^ Microbiology Department, Medical School, National and Kapodistrian University of Athens

**Objectives:** We have recently shown that azole-resistant *Aspergillus fumigatus* (AR-*Af*) with an environmental signature is present in Greece (Siopi JAC 2020). Nevertheless, the prevalence of azole resistance in Greek clinical isolates remains unknown. Based on these grounds, we investigated the occurrence of clinical AR-*Af* in Greece.

**Materials/methods:** A total of 206 *A. fumigatus* species complex (SC) strains recovered from multiple respiratory specimens of 180 patients (64 haematological, 75 paediatric whereof 50 with cystic fibrosis, 30 hospitalized in ICU whereof 13 with COVID-19 infection, 36 other) were collected from 7 centres (5 Athens, 2 Thessaloniki), were stored in 10% glycerol stocks at −70 °C and were retrospectively tested. Isolates were macro-/micro-scopically identified to SC level, while *A. fumigatus sensu stricto* (*SS*) were presumptively identified based on growth at 48 °C. In vitro susceptibility testing against amphotericin B (AMB), itraconazole (ITC), voriconazole (VRC), posaconazole (POS), isavuconazole (ISA), anidulafungin (AFG), caspofungin (CAS) and micafungin (MFG) was performed according to EUCAST E.DEF 9.3.2. Isolates exhibiting reduced susceptibility to azoles were subjected to confirmatory molecular identification and were further studied for the detection of specific mutations in the *cyp51A* gene, including its promoter region, associated with azole resistance (Mellado AAC 2007).

**Results:** All isolates grew at 48 °C indicating that they belonged to *A. fumigatus SS*. Antifungal susceptibility patterns among all strains are summarized in Table. Overall, 3/206 (1.5%) AR-*Af SS* from 2/180 (1.1%) patients were detected. Particularly, one of the three strains, recovered from pleural fluid culture of an ICU patient, did not have mutations in *cyp51A* gene and showed resistance to ITC/POS (MIC > 8/0.5 mg/L) but was susceptible to VRC/ISA (MIC 1/1 mg/L). The other two strains, isolated from subsequent sputum specimens of a 16-year-old cystic fibrosis patient without prior azole exposure, were pan-AR-*Af* (ITC, VRC, ISA and POS MIC >8, 4, 8 and 1 mg/L, respectively) harbouring the TR_34_/L98H resistance mechanism.

**Conclusions:** We report for the first time pan-azole-resistant clinical *A. fumigatus* in Greece. Although the occurrence of AR-*Af* is still limited, this finding must alarm the systematic local surveillance of azole resistance.

**Keywords:** azole-resistant *Aspergillus fumigatus*, clinical isolates, Greece

## P183 Prevalence of Dermatophytosis in Sheep and Goats Sold at Live Animal Markets in Ibadan, Oyo State, Nigeria

**Rotimi Oludare and Dimeji Oluwayelu**

Department of Veterinary Microbiology, Ahmadu Bello University, Zaria 810211, Nigeria

**Objectives:** The study was undertaken to determine the prevalence of dermatophytosis in sheep and goats sold at two major live animal markets in Ibadan, Oyo State, Nigeria. The two markets, Oranyan and Akinyele, are known for the sale of West African Dwarf (WAD) sheep and goats raised in Southwestern Nigeria and Ouda sheep and Red Sokoto goats transported from the northern region, respectively.

**Materials & Methods:** Based on the availability of sheep and goats, purposive sampling method was used. Samples were collected from adult sheep and goats with skin infections suggestive of dermatophytosis as follows: skin scrapings (sheep, n = 70; goats, n = 50) and hair samples (sheep, n = 20; goats, n = 10). The samples were treated with 10% KOH and examined microscopically for fungal elements. Thereafter, they were cultured using Dermatophyte Test Medium, Sabouraud’s dextrose agar, corn meal agar and potato dextrose agar with incubation at room temperature, followed by identification of each isolate through observation of colonial morphology and microscopic appearance of lactophenol cotton blue-stained smears prepared from the cultures. Statistical analysis was done using SPSS software and level of association between variables and occurrence of dermatophytosis was determined using Chi-square test. *p*-values <0.01 were considered significant.

**Results:** Out of the 150 samples, 13 dermatophytes (8.7%) were isolated namely: *Microsporum canis* (n = 3, 23.1%), Microsporum *gypseum* (n = 3, 23.1%), *Trichophyton tonsurans* (n = 2, 15.4%), *Trichophyton verrucosum* (n = 4, 30.8%) and *Epidermophyton floccosum* (n = 1, 7.7%). Dermatophytic lesions were found on four anatomical sites on the bodies of the sampled animals, viz: the head, neck, limbs and flank region with dermatophyte isolation rates per total sample collected being: 12%, 5%, 10% and 7%, respectively. However, there was no statiscally significant association between the number of dermatophytes obtained and the anatomical sites (*p* > 0.01).

Comparison of dermatophytosis prevalence among examined animals revealed highest detection rates in Ouda sheep (14.0%) and Red Sokoto goats (7.5%) which were from northern Nigeria and least detection rates in the WAD sheep and goats. There was however no significant association between the prevalence of dermatophytosis and breed of sheep and goats (*p* > 0.01). Similarly, although the prevalence of dermatophytosis was higher (25.3%) in female sheep and goats than in males (3.3%), the difference was not statistically significant. Two anthropophilic dermatophytes, *Trichophyton tonsurans* and *Epidermophyton floccosum*, were isolated from Ouda sheep in this study.

**Conclusions:** The zoophilic dermatophytes (*Trichophyton verrucosum, Microsporum gypseum* and *Microsporum canis*) isolated in this study pose a substantial health risk to occupationally exposed humans especially animal handlers, livestock farmers, abattoir workers and veterinarians. Additionally, the isolation of anthropophilic *Trichophyton tonsurans* and *Epidermophyton floccosum* from sheep in this study underscores the role of humans in the transmission of dermatophytes to domestic animals. This shows that these animals are not only reservoirs of zoophilic dermatophytes, but they can also serve as reservoirs of anthropophilic dermatophytes, thus making them potentially capable of transmitting the latter to in-contact or occupationally exposed humans. To our knowledge, this is the first report of isolation of *Epidermophyton floccosum* in domestic animals in Nigeria.

## P184 A Modified Point Prevalence Study of Antifungal Drug Use in Neonatal Units across Europe

**Elisavet Chorafa ^1^, Elias Iosifidis ^1^, Andrea Oletto ^2^, Adilia Warris ^3^, Elio Castagnola ^4^, Roger Bruggemann ^5^, Andreas Groll ^6^, Thomas Lehrnbecher ^7^, Laura Ferreras-Antolin ^8^, Alessio Mesini ^4^, Emmanuel Roilides ^1^ and CALYPSO Study Group ^1^**

^1^ Aristotle University Of Thessaloniki
^2^ Fondazione Penta Onlus
^3^ MRC Centre for Medical Mycology, University of Exeter
^4^ Istituto Giannina Gaslini
^5^ Radboud University Medical Centre
^6^ University Children’s Hospital Munster
^7^ Hospital for Children and Adolescents, Johann Wolfgang Goethe-University
^8^ St. George’s University Hospitals NHS Foundation Trust

**Objectives:** Knowledge of antifungal prescribing in neonatal units is extremely important. However, data on antifungal use in neonatal inpatients are limited. There is need to collect standardized multi-center data. Aim of CALYPSO study was to record antifungal consumption in infants hospitalized in neonatal units across Europe in order to obtain a better understanding of current practice and to identify areas to be targeted in future.

**Materials & Methods:** We organized a multicenter European 12-wk modified point-prevalence study (mPPS). All patients hospitalized in neonatal units (NUs) and receiving systemic antifungals in participating centers across Europe were included. Information about ward demographics and policies was collected once at the beginning; weekly ward and patient data (demographics, underlying conditions, risk factors, antifungal agents prescribed, dose, rational) were collected prospectively during the 12-wk study period and entered in REDCap database.

**Results:** Twenty-six NUs (18 Level 3, 4 Level 2, 4 Level 1) from 17 hospitals, located in 8 European countries with a median capacity of 21 beds participated in the study. The median percentage of neonates receiving antifungal agents per mPPS week across all NUs Level 3 was 9.9% (range 7.3–11.9). Great variations were observed among different NUs; median antifungal consumption ranged from 0% to 48.9% during the 12 w study period. A total of 166 patients were included in the study; 156 patients aged ≤90 d (md age = 6, Q1 = 3, Q3 = 18.5) and 10 aged 3–14mo (md age = 4, Q1 = 3, Q3 = 6.3). Prematurity was most common underlying condition among patients ≤ 90 d of age (87%), whereas chronic respiratory disease (60%) and history of surgery (40%) were among patients 3–14 mo. Indication for antifungal prescribing upon inclusion in the study was prophylaxis for 77% and treatment for 23% of prescriptions (69% empirical, 10% preemptive, 21% targeted, n = 39). Most common reasons for prophylaxis were prematurity, birth weight <1000 g, and presence of central venus catheters; whereas late onset sepsis was for empirical treatment. Fluconazole was the most frequently prescribed agent both for prophylaxis (98%, n = 129) and treatment (39%, n = 39). Dose of fluconazole for prophylaxis ranged from 2 to 8 mg/kg/day (md dose = 4.8) and for treatment from 3 to 12 mg/kg/day (md dose = 8.6), respectively. Liposomal amphoterecin b was used in 26% of patients for treatment with a dose range from 3 to 5 mg/kg/day (md dose = 4.2).

**Conclusions:** The majority of antifungal prescriptions across European NUs is for prophylaxis. Fluconazole is the most commonly prescribed antifungal agent both for prophylaxis and treatment with significant variation in dosing regimens across NUs. Results from this multicenter study can be a first step to guide a European antifungal stewardship program.

## P185 Behavioural Factors and Vulval Symptoms Associated with RVVC among Women of Childbearing Age in Southern Nigeria

**Samuel Fayemiwo ^1,2^, Lily Novak-Frazer ^3^, Isaac Adewole ^1^ and Riina Richardson ^3,4^**

^1^ College of Medicine, University of Ibadan
^2^ Division of Infection, Immunity and Respiratory Medicine, Faculty of Biology, Medicine and Health, School of Biological Sciences
^3^ Division of Infection, Immunity and Respiratory Medicine, School of Biological Sciences, NIHR Manchester Biomedical Research Centre (BRC) at the Manchester Academic Health Science Centre, The University of Manchester
^4^ Manchester University NHS Foundation Trust, Wythenshawe Hospital, Manchester

**Objectives:** Recurrent vulvovaginal candidosis (RVVC) is a clinical condition defined by a history of four or more acute inflammatory and culture-positive symptomatic episodes of vulvovaginal candidosis (VVC) in a 12-month period. This condition is established by the presence of compatible clinical signs, symptoms, and detection of *Candida* species in the laboratory by microscopy and culture from vaginal swabs. Predisposing factors for RVVC have not been thoroughly characterised, but are thought to include hormonal changes, as seen during pregnancy, uncontrolled diabetes, magnified host immune response, as well as other idiopathic causes. This study aimed to examine the behavioural and clinical characteristics associated with RVVC among women of childbearing age in southern Nigeria, which may differ from the western world (prevalence of 8–9%).

**Materials & Methods:** A prospective population-based cross-sectional study was conducted in three geopolitical zones of southern Nigeria (Anambra in the southeast (SE), Rivers in the south (SS) and Oyo in the southwest (SW)). Women of childbearing age were recruited in their homes or at Primary Health Centres in two randomly selected local government areas of the selected states. The participants, either symptomatic or asymptomatic, were given standardised symptom, health and lifestyle questionnaires. Data were analysed using SPSS version 25.0.

**Results:** The mean age of the participants was 35 years (range 18–55). Of the 585 women enrolled, 66.5% (389/585) were symptomatic at enrollment, with the highest prevalence in women from southeastern Nigeria (*P* = 0.001). The prevalence of women with a history of RVVC was also highest in SE Nigeria: 34.1% (62/182) in contrast to 25.7% (57/222) in the SW; and 8.8% (16/181) in the SS (*P* = 0.001). Overall, the most common behavioural factor associated with RVVC was frequent sexual activity, with an odds ratio (OR) of 2.7 (1.0–6.9, 95%CI, *P* = 0.035). Other factors, though not statistically significant, included the practice of oral and anal sex, wearing of tight-fitting trousers, use of local herbs. Risky sexual behaviour was more common in the southeastern zone. Clinical characteristics associated with RVVC included being symptomatic at time of enrollment (3.1 (2.1–4.7) *P* = 0.0001), genital soreness (2.6 (1.6–4.2); *P* = 0.001), burning sensation (2.4 (1.5–3.9); *P* = 0.001;), genital irritation (3.3 (2.1–5.1) *P* = 0.0001), abnormal vaginal discharge (3.3 (2.1–5.1), *P* = 0.003), genital pruritus (2.3 (1.6–3.4), *P* = 0.0001), presence of genital rashes (2.4 (1.0–5.7), *P* = 0.043), dysuria (2.2 (1.3–3.6), *P* = 0.002) and vaginal spotting (2.2 (1.07–4.3), *P* = 0.028). Logistic regression showed that those who inserted synthetic objects into their genitals (*P*-value for AOR = 0.044) and those having abnormal vaginal discharge (*P*-value for AOR = 0.001) were at least twice likely to have RVVC.

**Conclusions:** RVVC is more common in Nigeria than in the western world, and there are regional differences in their aetiology and epidemiology. Women between the ages of 25–29 and 35–39 years are mostly affected. Numerous factors, including risky sexual behaviour, vaginal spotting and dysuria, were associated with the occurrence of RVVC.

## P186 Cystic Fibrosis Patients Fungal Epidemiology in a Tertiary Hospital in the North of Portugal

**Dolores Pinheiro**

Serviço de Patologia Clínica—Centro Hospitalar Universitário S. João

**Objectives:** Cystic Fibrosis (CF), a hereditary disease, is caused by mutations on the CF transmembrane conductance regulator (CFRT) gene, on chromosome 7. CFRT protein is present in multiple organs of the human body, but it is particularly important in the respiratory trat, where its impairment leads to increased mucus thickness, unable to be cleared by the mucociliary system. This condition promotes local chronic infection and inflammation. Accordingly, patients with CF develop recurrent infections by bacteria, virus, or fungi together or separately along different periods of time and patient’s ages. The purpose of this work was to prospectively identify yeast and filamentous fungi (FF) in the patient’s sputum samples sent to the microbiology laboratory in 2019, in a tertiary hospital on the north of Portugal.

**Materials & Methods:** All sputum samples belonging to CF patients with mycological exam, were culture on Sabouraud medium, incubated in aerobiosis atmosphere at 25 °C and observed for growth every 2 days, until a total of 7. Those media with presence of yeast and FF proceeded for identification. For yeast, Vitek® MS (MALDI-TOFF methodology) and Vitek®2 (biochemical tests) from biomerieux were used; for FF, classical morphological and microscopical methods of fungal colony assessment were employed.

**Results:** On 32 patients (20 female and 12 males) with a medium age of 21.6 years old (range: 5 to 51) a total of 114 samples exhibited fungal growth and 137 strains were identified. Among them, 79 (57.7%) were yeast: 53 *Candida albicans*, 20 *C. parapsilosis*, 2 *C. dubliniensis*, 2 *C. lusitanea* and 2 *C. glabrata*; and 58 (42.3%) were FF: 15 *A. fumigatus*, 1 *A. niger*, 12 *Exophiala dermatitidis*, 12 *Rasamsonia argillacea complex*, 8 *Scedosporium apiospermum*, 9 *Penicillium* spp. and 1 *Fusarium* spp. In most patients, one single strain was identified but in some, two or even three strains were present.

**Conclusions:** The results show a fair balance between yeast and FFs in these patients. In addition, they provide evidence on fungal diversity, suggesting that some strains are replaced by others over time. While the meaning of these changes is uncertain, also because *Candida* spp. are a usual inhabitant of the oral cavity, they emphasize the view that CF patients require regular fungal assessment along the course of the disease.

## P188 Analysis of Resistance in Antifungals (ARIA)—Global Surveillance of *Candida* spp. Isolates, Including *C. auris*, in 2019

**Ian Morrissey ^1^, Stephen Hawser ^1^, Nimmi Kothari ^1^ and Mahmoud Ghannoum ^2,3^**

^1^ IHMA Europe
^2^ Center for Medical Mycology, University Hospitals Cleveland Medical Center and Case Western Reserve University
^3^ NTS Ventures

**Objectives:** ARIA is an annual surveillance initiative collecting yeast and fungal isolates from hospitals worldwide designed to determine susceptibility to antifungal agents and trends over time. The data presented here are for *Candida* spp. collected in 2019 (study year 1) from Argentina (number of sites = 1), Australia (2), Czech Republic (1), Germany (1), Italy (1), Panama (1), Taiwan (1), Turkey (1) and the USA (3).

**Materials & Methods:** Isolates (n = 730) were collected and re-identified by MALDI-TOF or molecular methods. MIC determinations were performed at a central laboratory by the Clinical and Laboratory Standards Institute (CLSI) broth microdilution method (CLSI standard M27) using amphotericin B (AMB), anidulafungin (AFG), caspofungin (CFG), fluconazole (FLC), isavuconazole (IVC), micafungin (MFG), posaconazole (PSC) and voriconazole (VRC). Percentage susceptibility (%S) or wild-type (%WT) were calculated according to CLSI breakpoints (CLSI supplement M60) or epidemiological cut-off values (CLSI supplement M59), respectively.

**Results:** Summary MIC and susceptibility data for all isolates combined are shown in the Table. Most isolates were at least 90% %S or %WT to the antifungals tested. Those with lower susceptibility are highlighted in grey shading and in these cases susceptibility was variable by country. The %WT for PSC vs. *C. albicans* ranged from 26.7% in Turkey to 90.9% in Germany. Similarly, AFG susceptibility against *C. glabrata* was 15.8% in Italy but 91.7% in Panama. MFG susceptibility in *C. glabrata* ranged from 76.9% in Turkey to 100% in Argentina. Micafungin susceptibility in *C. krusei* was 100% in Argentina, Australia. Italy and Taiwan, whereas susceptibility in the USA was 73.3%. FLC susceptibility in *C. parapsilosis* was 100% in all countries except Italy (52.4%) and Turkey (76.2%) which reduced the overall susceptibility to 88.5%. Isolates of *C. tropicalis* from Australia, Panama, USA and Taiwan were >90% WT to PSC in contrast to isolates from Argentina (57.1%) and Italy (47.4%). No CLSI breakpoint or epidemiological cut-off value is available for *C. auris*. However, the US Centres for Disease Control and Prevention has issued tentative breakpoints (https://www.cdc.gov/fungal/candida-auris/c-auris-antifungal.html). Using these breakpoints, full susceptibility was observed to all the antifungals tested except FLC where 56.3% were susceptible.

**Conclusions:** Most *Candida* spp. were fully-susceptible to the antifungal agents tested, but where non-susceptibility did occur this varied by country and antifungal agent. These differences are important to help clinicians make optimum choice of antifungal agent. As ARIA evolves it will become an essential tool to monitor and assess changes in antifungal resistance by geography and over time.

## P189 Large-Scale Molecular Epidemiological Assessment of Candida Species Isolated from Patients in Nigeria

**Rita Oladele ^1,9^, Folake Peters ^2,9^, Mark Okolo ^3^, Iriagbonse Osaigbovo ^4^, Y AbdulHakeem ^5^, U Udoh ^6^ and Ben Stielow ^7,8^**

^1^ Department of Medical Microbiology and Parasitology, College Of Medicine, University Of Lagos, Lagos State, Nigeria
^2^ Mycology Unit, Medical Microbiology and Parasitology Laboratory, Lagos University Teaching Hospital
^3^ Department of Medical Microbiology and Parasitology, University of Jos Teaching Hospital
^4^ Department of Medical Microbiology and Parasitology, University of Benin Teaching Hospital
^5^ Federal Medical Centre, Yola
^6^ University of Calabar Teaching Hospital
^7^ Radbound UMC, Centre for Infectious Diseases
^8^ Thermo Fisher Scientific
^9^ Medical Mycology Society of Nigeria

**Objectives:** Molecular identification and typing methods have proven to be very useful to unravel the epidemiological and population structure of geography dependent *Candida* species infections, facilitating the understanding of the dynamics of candidiasis in the human population. Information obtained from molecular approaches determines specific features that enable isolate discrimination and measure of isolate relatedness. This study aimed to determine the molecular epidemiology of *Candida* species isolated from patients in a tertiary hospital in Lagos Nigeria.

**Materials and Methods:** Study design was cross-sectional involving isolates from clinical samples of in-patients as well as out-patients. Stored identified isolates of *Candida* spp. were collected on pre-prepared sabouraud dextrose slants. Pre-identification of *Candida* spp. was performed using routine conventional microscopy and biochemical tests. Molecular characterization methods were employed for the identification of the *Candida* species using internal transcribed spacer (ITS) and 28S large ribosomal subunit regions as well matrix-assisted laser desorption/ionization time-of-flight mass spectrometry (MALDI TOF). Gene sequences obtained were accessioned and deposited in the Gene Bank.

**Results:** Majority of the *Candida* spp. were isolated were from; HVS 112(51.4%), urine 44(20.2%), sputum 18(8.3%), blood cultures 14(6.4%), wound swabs 10(4.6%) and stool 10(4.6%) samples. Age range of patients was between 8 weeks up to 97 years, median 33 years with IQR 18.5. Females accounted for majority of the samples (160 [73.4%]). The total number of yeast isolates sequenced were 219; *Candida albicans* 114(52.3%), *Candida tropicalis* 43(19.7%), *Candida glabrata* 22(9.7%), *Candida parapsilosis* 11(5.0%), *Candida orthopsilosis* 7(3.2%), and other atypical yeast spp. (including *Aurobasidium melanogenum, Issatchenkia orientalis (Pichia kudriavzevii), Kluyveromyces cf marxianus, Starmerella sorbosivorans*). Twenty-one (9.6%) of the yeast isolates identified as *Candida* spp. were not *Candida*.

**Conclusions:** The results of the present investigation revealed a high degree of genetic diversity among the yeast isolates being isolated from clinical specimens in Nigeria. Whole-genome sequencing will provide more information regarding evolutionary pathway geographical locations and anatomical sources of the *Candida species*.

## P190 Physiological and Genetic Relatedness between Human and Animal *Candida albicans* Isolates Recovered from Southeastern Nigeria

**Ifeanyi Elibe Mba and Emeka Innocent Nweze**

University Of Nigeria Nsukka

**Objectives:** *Candida albicans* is currently the most implicated pathogenic fungal species recognized as the major cause of various human and animal fungal infections globally. Knowing the local epidemiology of *Candida albicans* and evaluating its diversity is essential and will help understand and control their transmission globally. The present study was conducted to evaluate the physiological and genetic relatedness between human and animal *C. albicans* isolates in Nigeria.

**Methods:** Clinical *Candida albicans* (n = 96) were isolated from urine, high vaginal swab (HVS) and blood. In contrast, *Candida albicans* (n = 41) were isolated from the rectal swab, blood, feces, and milk in animals: goat, sheep, cattle, pig and chicken. The identification of the species was performed using standard methods. Enzymatic activity was screened using plate methods. Susceptibility testing was carried out using disk diffusion and broth microdilution methods. The *C. albicans* isolates that were highly resistant to the antifungal agents and showed a strong ability to produce extracellular hydrolytic enzymes were typed by random amplified polymorphic DNA polymerase chain reaction (RAPD-PCR) using three primers (P4, OPA-03 and OPE-18). RAPD-PCR DNA banding patterns that represent the DNA fingerprint were analyzed. After ethidium bromide staining, polymorphism was detected as the presence or absence of bands of particular sizes and intensity. The dendrogram was constructed using a matrix generated by UPGMA (Unweighted Pair Group Method with Arithmetic Means).

**Results:** A statistically significant difference (*P* = 0.031) was observed in the distribution of *Candida* spp. recovered from humans and animals. The Pz values of human *Candida* albicans for proteinase, hemolysin, lipase and phospholipase were 0.65 ± 0.97, 0.61 ± 0.81, 0.59 ± 0.47 and 0.76 ± 0.74 respectively, while that of *Candida* species recovered from the animal were 0.67 ± 0.13, 0.61 ± 0.95, 0.62 ± 0.67 and 0.69 ± 0.70 respectively (Table 1). Thus, there was no statistically significant difference (*P* > 0.05) in the in vitro enzymatic activity between the two groups. A high azole-resistance rate was observed. Resistance was higher among human *Candida* isolates than animal isolates, although the difference was not statistically significant (*P* = 0.519). The three primers used in this study produced appropriate multi-shape bands encoded within a range from 100 bp–1300 bp (Figures 1–3). Regardless of isolate sources, several DNA bands with different sizes and variable electrophoretic patterns were observed in this study. Therefore, our RAPD analysis showed a high degree of heterogeneity. No evidence of any clonal relationship was observed as a high degree of separation of human and animal *C. albicans* isolates into distinct genotypes was evident. Most of our isolates showed less than 80% similarity suggesting the presence of independent strains.

**Conclusions:** Though this is a preliminary study, we observed different banding patterns suggesting a high degree of discriminatory power for the three primers used. It further confirms the usefulness of RAPD-PCR in molecular typing of *Candida* isolates. Moreover, this study underscores the importance of animals and their products as potential sources/reservoirs and means of transmission of pathogenic and multi-azole resistant *Candida* species in Nigeria.

**Keywords:** *Candida albicans*; antifungal resistance; virulence factors; genotyping; RAPD PCR; human; animal

## P191 Prevalence of Dermatophytoses in School Age Children and Outpatients in Enugu Metropolis, Enugu State, Nigeria

**Ekene Chidebelu, Emeka Nweze and Ukamaka Udeh**

University Of Nigeria Nsukka

**Objectives:** Dermatophytosis is an evolving epidemiologically important fungal infection in Nigeria with increased incidence especially in children of school age. The infection has contributed to overall public health burden due to the treatment cost complicated by the occassional emergence of resistant strains. This study investigated the prevalence of dermatophytosis in children of school age, and among patients in Enugu metropolis.

**Materials & Methods:** The study population whose informed consents and demographic data were obtained comprises of school children and outpatients from selected schools and hospitals within the study area. Preliminary screening of the samples (n = 200) was done by direct observation of the wet mount digested in 20% KOH under the ×10 and ×40 objectives. The samples in normal saline solution, were inoculated on SDA plates using sterile swab sticks, and then incubated at room temperature and at 37 °C for 2 to 4 weeks. The suspected colonies consistent with cultural and microscopic characteristics of dermatophytes were identified using the reference fungal atlas.

**Results:** The recovery rate of dermatophytes was 30.5%. Higher prevalence was recorded in school samples (93.44%) than the hospital samples (6.56%). Age distribution of the positive samples showed a higher occurrence among early school age (5–9 yrs, 19.67%) and the adolescent age groups (10–14 yrs, 65.57%); than the adult population (1.64–3.28%). Incidence in females was more in (52.5%), than in males (47.5). Body lesion with the highest incidence of dermatophytes was scalp (67.2%), while the rate of isolation from nails was the lowest (4.91%). The predominant dermatophyte species were *Trichophyton mentagrophytes* (40.98%), *T. tonsurans* (24.59%), and *T. verrucosum* (18.03%).

**Conclusions:** These findings indicated that school age children are vulnerable to dermatophytoses, and good personal hygiene should be encouraged in children. Sharing of personal effects such as towels and handkerchiefs among children should be discouraged to reduce the risk of the infection spread in the young population.

## P192 Mucormycosis: An Emerging Challenge in Covid19 Pandemic

**Jagdish Chander and Surinder Singhal**

Sector 32, Government Medical College Hospital

**Objectives:** Mucormycosis is a rapidly destructive necrotising fungal infection usually seen among the diabetics and also in patients with other types of immunocompromised background. It occurs due to disruption of normal protective barrier hence local risk factors include trauma, burns, surgery, surgical splints, arterial lines, injection sites, biopsy sites, tattoos and insect or spider bites. The systemic risk factors for mucormycosis are hyperglycemia, ketoacidosis, malignancy, leucopenia and immunosuppressive therapy; however, infections in immunocompetent host are now well-described. Mucormycetes are upcoming as emerging agents leading to fatal consequences, if not timely detected/treated. Covid19 pandemic has led to more than 11,000 cases of rhino-orbito-cerebral mucormycosis in India during a very shot span of time. In the mainstream media mucormycosis is being projected as black fungus throughout this pandemic. This is rather a misnomer and should not be used because Mucorales are hyaline fungi. Recently, mucormycosis has been declared as a notifiable disease by various states of India. Eventually ICMR/GOI on May 20, 2021 covered this disease under Epidemic Disease Act, 1897, wherein all government and private health facilities, medical colleges will follow guidelines for its screening, diagnosis and management. Therefore, it has become an acute emergency being a life-threatening during Covid19 pandemic to the patients thereby a challenge to the medical fraternity at large. This two minutes video is being presented to highlight the importance of this unprecedented disease.

**Materials & Methods:** The debrided necrotic skin tissue was processed as per standard mycological protocol ring the Covid19 pandemic period. The clinical entity was diagnosed by mounting biopsy material on potassium hydroxide (10%), CFW, histopathology study of tissue sections stained by hematoxylin and eosin (H&E), periodic acid-Schiff (PAS) and Gomori methenamine silver (GMS) stainings as well as by fungal culture on conventional media, with morphological identification of isolates with LCB mount. All patients were treated with amphotericin B along with extensive surgical debridement of necrotic tissue tissue.

**Results:** Majority of the martinets belong to the rhino-orbital mucormycosis and Rhizopus arrhizus is the commonest species isolated throughout the country. The underlying factor was either diabetes mallets or indiscriminate use of steroids for treating Covid19. The other risk factors like use of mask, lack of exercise due to frequent lockdowns, contaminated masks and use of contaminated oxygen supply accessories, however, these have not yet proved. The overall mortality among the patients was found to be on an average a fifty percent.

**Conclusions:** During this pandemic of Covid19, thousands of patients have presented with mucormycosis particularly rhino-orbito-cerebral type. This is very significant change in the Incidence and prevalence of the Mucoralean fungi and underlying factors are to be explored in details. Moreover, index of suspicion of fungal etiology should be kept very high to avoid any time wastage in establishing diagnosis and empirical treatment on bacterial lines. Early diagnosis, prompt and extensive surgical debridement and appropriate antifungal therapy are the key to treat such type of patients and their lives can be saved from mucormycosis.

## P193 Antifungal Prescription Practices and Average Length of Stay (ALoS). Evidence from the Public Hospitals in Romania

**László Lorenzovici ^1,2^, Andrea Bârzan-Székely ^1^, Szabolcs Farkas-Ráduly ^1^ and Maria Gheorghe ^3^**

^1^ Syreon Research Romania
^2^ G. E. Palade University of Medicine, Pharmacy, Science andTechnology
^3^ Pfizer

**Objective:** ECDC indicates that Romania is among the Member States with the highest levels of antimicrobial resistance (AMR) in Europe. However, there is no National Antimicrobial Stewardship Program (ASP) implemented to facilitate national guidelines on antimicrobial prescription. Furthermore, studies using local hospital data of cases with aspergillosis and mucormycosis are scarce. The aim of this study is to estimate the average length of stay (ALoS) and observe the prescription practices of treating aspergillosis and mucormycosis infections in public hospitals in Romania.

**Methods:** This analysis used official diseases codes data reported from all public hospitals in Romania (515 hospitals) in years 2018 and 2019. We used 6 diseases codes for aspergillosis and mucormycosis including pulmonary aspergillosis, gastro-intestinal mucormycosis and other forms of unspecified aspergillosis and mucormycosis. A separate analysis using data from 21 large hospitals investigated to what extent prescription protocols for fungal infections included a microbiologic test.

**Results:** We found that on average, in 2019, Romanian hospitals reported approximately 4 cases of fungal infections treated per 100,000 per year with 80% of these cases having a disease code indicative of pulmonary aspergillosis. However, in the absence of rapid testing only in 20% of all cases the fungal prescription practices included a test to confirm the fungal infection. In addition, we found that the average ALoS was 17 days [95% CI: 16.2–17.2] with approximately 6 days [95% CI: 5.7–6.3] spent on average in the intensive care unit (ICU). Importantly, we observed that, compared to patients who did not have renal impermeant, those with renal impermeant spent on average 60% more time in the intensive care unit.

**Conclusions:** Considering that 80% of patients treated with antifungals in Romanian public hospitals did not have a lab test to confirm the fungal etiology, we can conclude that antifungals are often used in an ad-hoc manner. An optimal use of antifungal treatment in Romania and a decrease of ALoS can be achieved through the development of national guidelines on rapid diagnosis and early treatment of fungal infections.

## P194 Fatal Cryptococcal Meningitis in Anon-HIV Infected Male with Documented Exposure Risk in Nigeria

**Iriagbonse Osaigbovo ^1,2^ and Steven Igetei ^2^**

^1^ University Of Benin
^2^ University of Benin Teaching Hospital

**Objectives:** To describe a fatal *Cryptococcal Meningitis* in an apparently immunocompetent patient with documented exposure risk.

**Materials & Methods:** A 28 year old Nigerian male was referred to our facility with recurrent frontal, throbbing headaches and vomiting of 7 weeks, fever of 1 week and irrational talk of a day’s duration. Headaches began insidiously, were of moderate to severe intensity, each episode lasting up to 18 h a day, affecting daily activities and transiently relieved by analgesics. Headaches worsened 2 weeks prior to presentation with associated diplopia and neck stiffness necessitating presentation at the referring centre. There was no history of psychoactive drug use or psychiatric illness nor recent travel to states in the well described meningitis belt. He was not diabetic and retroviral status was unknown. Antibiotics given at the referring centre did not improve clinical status hence the referral.

Lumbar puncture was done and CSF sent for microscopy, culture and sensitivity (M/C/S), GeneXpert and chemistry. Full blood count, electrolytes/urea/creatinine, retroviral screen, and random blood glucose were ordered.

**Results:** CSF M/C/S was blood stained so white cells were not reported. No organisms were seen on Gram stain. Cultures yielded no growth after 48 h. GeneXpert did not detect *Mycobacterium tuberculosis*. However, CSF protein was markedly elevated at 184.8 mg/dL; CSF glucose was low at 29 mg/dL with a concomittant random blood glucose of 127 mg/dL. The retroviral screen was non-reactive. Clinical microbiologist invited to review on day four considered cryptococcal meningitis. Serum cryptococcal antigen testing using non-routine IMMY CrAg strips placed on-site by the Medical Mycology Society of Nigeria for screening HIV patients returned positive. Archived CSF also tested positive. Culture plates were discarded after 48 h so speciation was not done.

Further probing revealed history of recent move to a house opposite a poultry 3 months before presentation. Patient took ill a month after the move. On day four review, patient was unable to perceive light. Bilateral lateral rectus palsy was noted. An assessment of complicated *Cryptococcal Meningitis* in an apparently immunocompetent adult was made.

Induction therapy was commenced with intravenous Amphotericin B 50 mg into 500 mL of 5% dextrose water over 3 h daily and intravenous Fluconazole 1.2 g daily. He deteriorated by day 7 with blood pressure of 170/110 mmHg and persistent tachycardia; ECG showed sinus tachycardia. Glasgow coma scale dropped from 13/15 to 7/15. An assessment of raised ICP was made and therapeutic lumbar puncture planned. However, he continued to deteriorate until his demise on the eight day.

**Conclusions:** *Cryptococcal Meningitis* in the immunocompetent is missed or diagnosis delayed in sub-Saharan Africa because of lack of diagnostic tools and a myopic, though justified focus on cryptococcal disease caused by HIV/AIDS. In this case, treatment was delayed due to low index of suspicion because of non-HIV status and failure to promptly elicit environmental exposure. Interdisciplinary collaboration and use of a simple but non-routine diagnostic test finally clinched diagnosis. Attention should be paid to the epidemiology of non-HIV cryptococcal disease and making diagnostic tests readily available in sub-Saharan Africa.

## P195 The Impact of the COVID-19 Pandemic on the Frequency of Candidemia in a Tertiary Greek Hospital

**Nikolaos Zapaniotis ^1^, Athanasios Kakasis ^2^, Angeliki Padazatou ^1^, Ioannis Delliolanis ^1^, Nikolaos Sipsas ^2^ and Maria Gamaletsou ^2^**

^1^ Microbiology Department, “Laiko” Athens General Hospital
^2^ Pathophysiology Department, School of Medicine, National and Kapodistrian University of Athens

**Objectives:** To evaluate the possible impact of the COVID-19 pandemic on the incidence of candidemia in a tertiary hospital.

**Methods:** We retrospectively reviewed the *Candida* spp. isolates in blood cultures within the first year of the COVID-19 pandemic (March 2020 to February 2021) in comparison to the previous one-year period (March 2019 to February 2020). For that purpose we extracted data from the Microbiology Laboratory of “Laiko” Athens General Hospital. Duplicate blood culture results which were corresponding to the same patient were excluded.

**Results:** Overall, during the first year of the pandemic there were 47 *Candida* spp. isolates in blood cultures of which 25 (53.2%) were *Candida parapsilosis*, 16 (34.1%) *Candida albicans*, 4 (8.5%) *Candida tropicalis*, 1 (2.1%) *Candida krusei* and 1 (2.1%) *Candida glabrata*. During the preceding one-year period there were also recorded 47 *Candida* spp. blood culture isolates. These isolates were distributed as follows: 22 (46.9%) *Candida albicans* isolates, 12 (25.5%) *Candida parapsilosis* isolates, 7 (14.9%) *Candida glabrata* isolates, 4 (8.5%) *Candida krusei* isolates and 2 (4.2%) *Candida tropicalis* isolates. There is a significant increase (*p* = 0.006) in the number of *Candida parapsilosis* isolates during the pandemic period. These results can be overviewed in Figure 1.

**Conclusions:** There was no difference in the incidence of candidemia cases in our hospital during the first year of the COVID-19 pandemic compared to the previous one-year period. However, there is a significant change in *Candida* spp. distribution, notably a shift towards *Candida parapsilosis*. This finding could be due to a decline in the hospital’s infection control program, taking into consideration that *Candida parapsilosis* is associated with poor hand hygiene practices. Such findings underline the overwhelming impact of the pandemic which caused an increased workload on hospital staff.

## P196 Head and Neck Manifestations of Paracoccidioidomycosis: A Retrospective Study of Histopathologically Diagnosed Cases in Southern Brazil

**Maria Lúcia Scroferneker ^1,2^, Alessandra Koehler ^1^, Fábio Muradás Girardi ^3^, Leo Kraether Neto ^4^ and Paulo Cezar de Moraes ^1^**

^1^ Postgraduate Program in Medicine: Medical Sciences, Universidade Federal do Rio Grande do Sul
^2^ Department of Microbiology, Immunology and Parasitology, ICBS, Universidade Federal do Rio Grande do Sul
^3^ Integradet Oncology Center, Hospital Ana Nery
^4^ Professor at Universidade de Santa Cruz do Sul (professor) and coordinator of the Bucal Diagnostic Project from Universidade de Santa Cruz do Sul

**Objectives:** Analyze the clinical and epidemiological characteristics of 28 cases of paracoccidioidomycosis (PCM) with head and neck manifestations from southern Brazil.

**Materials & Methods:** Retrospective analysis of cases of PCM with head and neck manifestations referred to two medical centers in the municipality of Santa Cruz do Sul, state of Rio Grande do Sul, during a 10-year period (2011–2020). The medical centers were the Hospital Ana Nery and the Dentistry Clinic of Universidade de Santa Cruz do Sul. All cases were histopathologically diagnosed. The following data were analyzed: year of diagnosis, age at diagnosis, gender, race, smoking habit, place of origin, schooling, anatomical sites of the lesions, evolution time of the symptoms, presence of cancer and other associated diseases and outcomes. Data analysis was carried out by descriptive statistics. Informed consent forms were obtained from all of the participants.

**Results:** Twenty-eight patients were selected. The number of cases remained stable during the analyzed period, ranging from one to four cases per year. However, in 2019, there was a considerable increase, with 11 diagnosed cases. Age at diagnosis ranged from 29 to 80 years. The predominant age range was between 40 and 59 years old, with 46% of the patients. In total, 21 (75%) were men and 7 (25%) were women, with the male:female ratio 3:1. Most were Caucasian (92%) and 46% were smokers. Patients were from 12 municipalities of the East Center region of the state of Rio Grande do Sul. Most of the patients (59%) had not finished elementary school. Regarding clinical characteristics, the two most common anatomical sites of the lesions were the soft palate and the larynx, both in six cases, followed by the lips (five cases) and the face skin (four cases). The evolution time of the symptoms was recorded only in ten cases. Among these, eight cases had evolution time between one and four months. A longer time (15 and 24 months) was observed only in two cases. The associated diseases recorded were hepatitis C, HIV, HIV plus pulmonary/ganglionic tuberculosis, diabetes mellitus (each one in one case) and hypertension (three cases). Associated squamous cell carcinoma was present in three cases. A total of three deaths occurred, but none of them was directly associated with PCM.

**Conclusions:** This is the first study to analyze PCM cases from the East Center region of the state of Rio Grande do Sul. The predominance of men aged between 40 and 59 years and smokers is according with the epidemiological data found in literature. However, we found a lower male:female ratio (3:1) than that usually reported (22:1). The occurrence of a greater number of cases in Caucasian individuals is according to a recent study that showed that white individuals are more affected by PCM. The large increase in cases diagnosed in 2019 is a data that deserves attention and is possibly associated with the climatic conditions of the period, when there were many droughts in the region. PCM is endemic to southern Brazil and differential diagnosis with granulomatous and neoplastic diseases must always be done.

## P197 Epidemiological Aspects of Sporotrichosis in Brazil: A Study of 62 Cases in the State of Rio Grande do Sul

**Maria Lúcia Scroferneker ^1,2^, Natália Andressa Buss Venier ^3^, Alessandra Koehler ^1^, Fernanda Brandão Pacheco ^3^ and Gerson Vettorato ^3^**

^1^ Postgraduate Program in Medicine: Medical Sciences, Universidade Federal do Rio Grande do Sul
^2^ Department of Microbiology, Immunology and Parasitology, ICBS, Universidade Federal do Rio Grande do Sul
^3^ Department of Dermatology of the Hospital Santa Casa de Misericórdia de Porto Alegre

**Objectives:** Analyze the clinical and epidemiological characteristics of 62 cases of sporotrichosis from southern Brazil.

**Materials & Methods:** Retrospective study of reports from the Mycology Laboratory located in the Dermatology Department of the Irmandade Santa Casa de Misericórdia Hospital Complex, Teaching Hospital of the Federal University of Health Sciences of Porto Alegre (UFCSPA), in the state of Rio Grande do Sul, in the southern region from Brazil, over a period of seventeen years, two months and 11 days. All patients, of all ages, who visited the mycology laboratory from 1 January 2002 until 11 March 2020, the date of the closure of the service, who had a positive result for *Sporothrix* sp. in the cultural mycological examination, obtained from scraping of skin lesions or fragment of lesions suggestive of sporotrichosis, were included.

**Results:** 62 patients were included, 34 male (54.83%) and 28 female (45.16%). The age ranged from 8 to 82 years, with an average of 46.43 years and a child of 8 years (1.6%). The number of cases per year was variable, with an average of 3.44 cases per year in the period from 2003 to 2020. The year of 2019 had the highest number of cases (n = 8) and the years 2018 and 2020 did not have diagnosed cases in the laboratory. The upper extremities were the most affected (67.74%), followed by the lower limbs (25.80%), and also affected the head and face, chest and buttocks (3.22%). Of the 62 patients, 69.35% were from the metropolitan region of Porto Alegre, with 16 patients from the city of Porto Alegre and the rest from cities in the metropolitan region. The second region with the highest number of patients was northwest with 6.45%, followed by southeast with 4.83%, southwest and eastern center, both with 3.22%. The northeast and central eastern regions of the state of Rio Grande do Sul had no reported cases. There is a record of the treatment instituted for only 10 patients, due to the exchange for eletronic medical records. Of these, 60% were treated with itraconazole, 30% with potassium iodide, 10% with amphotericin B and 10% had spontaneous resolution of the condition. Regarding comorbidities, 40% were healthy, 30% hypertensive, 20% with thyroid disease and 10% generalized anxiety disorder.

**Conclusions:** Sporotrichosis is the most prevalent subcutaneous mycosis worldwide, with acute and subacute clinical manifestations. In Brazil, most cases are reported in the South and Southeast regions. It affects men and animals, with an increase in the incidence of cases in recent years, receiving prominence from public health entities such as the World Health Organization and the Pan American Health Organization. Several factors are also known that can lead species of the genus *Sporothrix* to present resistance to antifungals, like mutations in cytochrome P450 monooxygenases. Therefore, epidemiological studies on sporotrichosis are essential.

## P198 Clinical and Microbiological Findings Associated with RVVC among Women in Southern Nigeria

**Samuel Adetona Fayemiwo ^1,2^, Lily Novak-Frazer ^3^, Isaac Folorunso Adewole ^1^ and Riina Rautemaa-Richardson ^3,4^**

^1^ College of Medicine, University of Ibadan
^2^ Division of Infection, Immunity and Respiratory Medicine, Faculty of Biology, Medicine and Health, The University of Manchester, Manchester
^3^ Division of Infection, Immunity and Respiratory Medicine, School of Biological Sciences, NIHR Manchester Biomedical Research Centre (BRC) at the Manchester Academic Health Science Centre, The University of Manchester
^4^ Manchester University NHS Foundation Trust, Wythenshawe Hospital

**Objectives:** Vulvovaginal candidosis (VVC) is a common vaginal inflammatory disease in women of childbearing age and is mainly caused by opportunistic *Candida* species. Recurrent vulvovaginal candidosis (RVVC) is defined by a history of four or more acute inflammatory and culture-positive symptomatic episodes of VVC in 12 months. The signs and symptoms of VVC are non-specific and many other infectious or non-infectious conditions can present similarly and/or concomitantly. This study aimed to determine some epidemiological, clinical and microbiological features of RVVC and association with other co-infections among women of childbearing age in southern Nigeria.

**Materials & Methods:** A population-based prospective cross-sectional study was conducted across three states in the southern geopolitical zones of Nigeria (southeastern Nigeria, southsouthern Nigeria and southwestern Nigeria). A total of 585 participants, aged 18 to 55 years and with or without vulvovaginal symptoms at the time, were recruited from urban and rural areas in two randomly selected local government areas (LGAs) within the geopolitical zones. Patient histories were collected using a structured interview form. High vaginal swabs were collected from the sides of the vaginal wall to screen for the presence of *Candida* spp. by culture and sexually transmitted organisms by PCR. The Quick-DNA mini–Prep ZYMO extraction kits and the CFX-96 Real-time PCR using the AllplexTM Assay system (Seegene, Seoul, Korea) were used for the PCR. Data were analysed using SPSS version 25.0.

**Results:** The mean age of the participants was 35 years (range 18–55). Three-quarter (75.7%; 443/585) were married and their mean age of sexual debut was 19 years (Range 9–37). At the time of enrolment, two-thirds (66.5%) of the participants reported two or more vulval symptoms. The highest incidence was in southeastern Nigeria (*P* = 0.001). The prevalence of women with a history of RVVC was 23.1%, and 30.4% (41/135) of these had one or more *Candida* species isolated at enrolment. The prevalence of VVC in this study was 20.7%, while 6.3%. of them had asymptomatic vaginal Candida colonisation. Five *Candida* species were isolated from participants of any form of VVC: *C. albicans* (75.3%) followed by *C. glabrata* (12.0%), *C. krusei* (8.9%), *C. tropicalis* (3.2%) and *C. ciferrii* (0.6%). Other infections included bacterial vaginosis (BV) (43.6%), *Trichomoniasis* (13.0%), *Chlamydia trachomatis* (0.7%), *Mycoplasma genitalium* (1.4%), Hepatitis B infection (4.1%) and HIV infection (3.8%). In the multivariate analysis, there was no significant association between the Candida species isolated, vaginal colonisation and the likelihood of RVVC (*P* > 0.05). However, the odds ratio of 1.6 for current VVC (1.1–2.6) 95%CI; *P* = 0.028) and BV (OR (95%CI) = 1.6 (1.1–2.6); *P* = 0.027) were significantly associated with the increased risk of RVVC, respectively.

**Conclusions:** RVVC is common in Nigeria, and there are no significant regional differences in their aetiology and epidemiology. *Candida albicans* remains the main causative agent in all geopolitical zones in Nigeria. However, other concomitant infections were common in the study population.

## P210 Invasive Aspergillosis in Adult ICU Patients

**Olga Shadrivova ^1^, Nikolay Klimko ^1^, Evgeniia Zaytseva ^1^, Alisa Volkova ^2^, Marina Popova ^2^, Yulia Chudinovskikh ^3^, Marina Lebedeva ^4^, Andrey Saturnov ^5^, Olga Uspenskaya ^5^, Mariya Penkova ^5^, Svetlana Ignatyeva ^1^, Tatiyana Bogomolova ^1^ and Lyudmila Zubarovskaya ^2^**

^1^ North-Western State Medical University Named After I.I. Mechnikov
^2^ I. Pavlov First Saint Petersburg State Medical University
^3^ The Federal State Budget Institution «N.N. Petrov Research Institute of Oncology» of the Ministry of Public Health of Russian Federation
^4^ State Budgetary Healthcare Institution “Saint-Petersburg Clinical Scientific and Practical Center for Specialised Types of Medical Care (Oncological)”
^5^ Leningrad Regional Clinical Hospital

**Objectives:** Analysis of risk factors, aetiology, clinical features, treatment and survival rates in ICU patients with invasive aspergillosis (IA).

**Materials & Methods:** Retrospective analysis of data from 110 adult ICU patients with IA was conducted. The following criteria were used for diagnosis: EORTC/MSGERC (2019), ECMM/ISHAM (2020), and clinical algorithm *Asp*ICU (S. Blot, 2012). Group I—43 non-hematological patients, median age—63 (19–99), females—44%. Group II—67 hematological patients, 47 (18–75) years, females—34%.

**Results:** In non-hematological patients, IA more often developed against the background of severe COVID-19 or influenza (42% vs. 6%, *p* = 0.005), decompensated diabetes mellitus (35% vs. 7%, *p* = 0.006), acute or chronic renal failure (23% vs. 8%, *p* = 0.04). Other underlying conditions in the non-hematological group were autoimmune diseases—21%, malignant neoplasms—12%, heart and vascular diseases—7%, severe pneumonia—5%, primary immunodeficiency—2%; in hematological patients-lymphomas—43%, acute leukemias—42%, chronic leukemia—6%, aplastic anemia—6%, and multiple myeloma—3%. Common risk factors for the IA development in the ICU patients were the use of systemic steroids (72% vs. 78%), prolonged lymphocytopenia (80% vs. 82%, median—18 days in both groups), and immunosuppressive therapy (28% vs. 25%). Severe neutropenia was detected predominantly in hematological patients with IA (5% vs. 70%, *p* = 0.0001). Other risk factor in the hematological cohort was hematopoietic stem cell transplantation (24%).

The main sites of IA were lungs (100% vs. 97%). Dissemination of infection with ≥2 organs involvement (9% vs. 18%) and central nervous system involvement (4% vs. 12%) were more often diagnosed in hematological patients. In non-hematological patients, most severe clinical symptoms of IA were noted: fever (100% vs. 76%), respiratory failure (95% vs. 77%, *p* = 0.001), hemoptysis (37% vs. 13%, *p* = 0.01), cough (86% vs. 66%, *p* = 0.007) and chest pain (52% vs. 13%, *p* = 0.0003).

In non-hematological patients, cavities of destruction on CT scan more often registered (51% vs. 9%, *p* = 0.0001), the “halo” symptom was determined in hematological patients (9%). *Aspergillus* spp. positive culture was received in 67% and 45%, *p* = 0.03. The main etiology agents of IA were *A. fumigatus* (59% vs. 50%), *A. niger* (23% vs. 22%), and *A. flavus* (18% vs. 16%). In both groups, the most commonly used drug was voriconazole (90% vs. 82%). The overall 12-week survival rate of ICU patients with IA was low (55% vs. 45%).

**Conclusions:** In ICU patients, the main risk factors for IA development were steroid use (72% vs. 78%), lymphocytopenia (80% vs. 82%), neutropenia (5% vs. 70%, *p* = 0.0001) and immunosuppressive therapy (28% vs. 25%). Causative agents are *A. fumigatus* (59% vs. 50%), *A. niger* (23% vs. 22%) and *A. flavus* (18% vs. 16%). The main sites of infection were lungs (100% vs. 97%). Fever (100% vs. 76%), respiratory failure (95% vs. 77%) and haemoptysis (37% vs. 13%) are characteristic. The overall 12-week survival rate of ICU patients with IA was low (55% vs. 45%).

## P211 *Aspergillus* spp. Isolation in COVID-19 Critical Patients during Second, Third and Fourth Pandemic Waves in a Tertiary Hospital in Madrid

**Federico Becerra-aparicio ^1^, María Cruz Soriano-Cuesta ^2^, Jesús Fortún ^3^, Juan de Dios Caballero ^1,4^, Daniel Marcos-Mencía^1^, Rafael Cantón ^1,4^ and Elia Gómez G. de la Pedrosa ^1,4^**

^1^ Servicio de Microbiología, Hospital Ramón Y Cajal-IRYCIS
^2^ Servicio de Medicina intensiva, Hospital Ramón y Cajal
^3^ Servicio de Enfermedades Infecciosas, Hospital Ramón y Cajal
^4^ Red Española de Investigación en Patología Infecciosa (REIPI)

**Objectives:** To assess the prevalence and the microbiological characteristics of *Aspergillus* spp. isolates prospectively recovered from respiratory samples of non-immunocompromised patients admitted to the intensive care units (ICUs) in our institution during the period of the second, third and fourth COVID-19 pandemic waves in Madrid.

**Materials & Methods:** All *Aspergillus* spp. isolates recovered from COVID-19 patients admitted to the different ICUs in our hospital from one or more lower respiratory samples from September 2020 to May 2021 were included. Species identification was performed by microscopic visualization and MALDI-TOF MS (MALDI Biotyper®, Bruker Daltonik, Bremen, Germany) analysis using both Bruker (MBT Filamentous Fungi Library 3.0) and MSI online databases (species identification threshold >20 and >2 respectively). Antifungal susceptibility to amphotericin B and voriconazole of first isolate per species and patient was assessed by gradient strips (Liofilchem, Teramo, Italy). Galactomannan (GM) testing was performed using Platelia Aspergillus (BioRad Laboratories, Marnes-la-Coquette, France; cut-off ≥0.5 in serum and ≥1.0 in BAL) and 1,3-β-D-glucan (BDG) in serum was performed when required with the Wako β-glucan test (Fujifilm Wako Pure Chemical Corporation; cut-off = 11 pg/mL).

**Results:** Overall, 51 patients with 58 isolates were included (5/51 with 2 and 1/51 with 3 different species). Global prevalence during the study period was 8.3% (51/614). A total of 103 respiratory samples from these episodes yielded positive cultures, including 83 BAS, 15 BAL, 4 sputum and 1 tracheal aspirate. *Aspergillus* spp. growth in 2 or more samples was detected in 49% of patients (25/51). Most common species were *A. fumigatus sensu stricto* (60.3%, 35/58), *A. flavus* (10.3%, 6/58) and *A. terreus* (8.6%, 5/58). Other species were *A. hiratsukae, A. nidulans* (n = 2 each), *A. citrinoterreus, A. calidoustus, A. lentulus, A. parasiticus, A. sublatus* and *A. tubingensis* (n = 1 each). Discrepancies between Bruker and MSI identification were detected in 10 cases, 8 of them corresponding to *Aspergillus* cryptic species only identified using MSI database. BAL-GM was positive in 15/51 (29.4%) and 4/51 in serum samples (7.8%). BDG detection was assessed only in 3 cases with 1 positive result. Ten isolates (19.6%) showed in vitro high MIC values to amphotericin B (5 *A. terreus*, 3 *A. flavus*, 1 *A. fumigatus* and 1 *A. nidulans*, MIC > 32 mg/L) and only one isolate (1.9%) in the case of voriconazole (1 *A. fumigatus sensu stricto*, MIC > 32).

**Conclusions:** A high prevalence of isolation of different *Aspergillus* species among COVID-19 non-immunocompromised ICU patients (8.3%) was detected, including cryptic species. COVID-19 associated pulmonary aspergillosis could be assessed in 29.4% according to BAL-GM results. Moreover, in 11.7% cultures (6/51) more than one species were found, which also included cryptic species. Susceptibility studies revealed a high proportion of isolates displaying high MIC values to amphotericin B and voriconazole.

## P212 A Novel Zebrafish Larva Model for Influenza-Associated Aspergillosis

**Simon Feys ^1,2^, Jana Van Dycke ^3^, Cato Jacobs ^2^, Agustin Reséndiz Sharpe ^4^, Annelies Stevaert ^3^, Lieve Naesens ^3^, Katrien Lagrou ^4,5^, Joost Wauters ^1,2^ and Joana Rocha-Pereira^3^**

^1^ Department of Microbiology, Immunology and Transplantation, Laboratory for Clinical Infectious and Inflammatory Disorders, KU Leuven
^2^ Medical Intensive Care Unit, UZ Leuven
^3^ Department of Microbiology, Immunology and Transplantation, Rega Institute, Laboratory of Virology and Chemotherapy, KU Leuven
^4^ Department of Microbiology, Immunology and Transplantation, Laboratory of Clinical Bacteriology and Mycology, KU Leuven
^5^ Laboratory Medicine, UZ Leuven

**Objectives:** Influenza-associated pulmonary aspergillosis (IAPA) is a severe co-infection with high mortality rates, affecting critically ill influenza patients. Knowledge on the pathophysiology of IAPA is key to establish new diagnostic and therapeutic modalities, but is currently lacking. We aim to establish an infection model for influenza-associated aspergillosis using the transparent zebrafish larva. Its swimbladder has been validated as a model for the human alveolus at the anatomical, developmental and transcriptional level. Since zebrafish larvae do not possess an adaptive immune response yet, they are perfectly suited to investigate innate immunity. Development of these models will allow longitudinal in vivo visualization of the interplay between the pathogens and the innate immune response, and may additionally be used for high-troughput antiviral and antifungal drug screening.

**Materials & Methods:** Virus [10 to 100 RNA copies of influenza A (H1N1) virus: strains A/Virginia/ATCC3/2009 (pdm09) or GFP-expressing A/Puerto Rico/8/34 (GFP-PR8)]; or fungus [1 to 10 TagRFP-T-expressing *Aspergillus fumigatus* conidia (TagRFP-T-AF)]; or a combination of virus and fungus was micro-injected in the swimbladder of five days-old zebrafish larvae. Virus- or co-inoculated larvae were harvested in groups of 10 larvae at 0, 8, 24, 48, 72, 96 and 120 h post-inoculation (hpi) for viral copy determination. Larvae were observed using high-resolution fluorescence-microscopy at 0, 24, (32), 48, 80 and 120hpi.

**Results:** Virus replication in the swimbladder yielded a 20- to 1000-fold rise in viral RNA for both influenza virus strains, by RT-qPCR. Fluorescence microscopy of GFP-PR8 infected zebrafish larvae revealed a heterogenous green aspect of the swimbladder epithelium from 24hpi (Figure 1), but no associated mortality. Zebrafish larva inoculation with only TagRFP-T-AF conidia resulted in aspergillosis (defined as the development of hyphae seen by fluorescence-microscopy) in 14 out of 61 larvae (23%). This incidence was significantly higher in zebrafish larvae inoculated with GFP-PR8 or pdm09 prior to TagRFP-T-AF conidia (42/73, 58%, *p* < 0.001 and 43/68, 63%, *p* < 0.001, respectively, Graph 1). Aspergillosis developed in the zebrafish larva swimbladder in four distinct phases both upon mono- or co-infection: swelling of the conidia, germination with formation of hyphae (Figure 1), extensive non-invasive hyphal mass formation and finally invasion through the swimbladder wall into the surrounding tissue.

**Conclusions:** We present a novel influenza virus—*Aspergillus fumigatus* co-infection model in zebrafish larvae, which enables us to visualize the course of these (co-)infections as they develop in the air-filled swimbladder. Here, aspergillosis recapitulates what is observed in the human respiratory tract, starting with swelling of conidia and ending up with invasion of the surrounding tissue. Likewise, a sublethal influenza virus infection leads to a rise in incidence of aspergillosis in the swimbladder. Further validation of these zebrafish larva models is ongoing and will soon provide a novel tool for pathophysiological and therapeutic research regarding aspergillosis and IAPA.

## P213 COVID-19-Associated Pulmonary Aspergillosis and Antifungal Consumption in the Critical Care Unit

**Grace Chan, Nuala Scanlon, Assumpta Killarney, Fiona Hegarty, Colman O’Loughlin, Deirdre Brady, Margaret Hannan and Maureen Lynch**

Mater Misericordiae University Hospital

**Objectives:** COVID-19-Associated pulmonary aspergillosis (CAPA) is increasingly reported in our Critical Care Unit, a National Centre for Extracorporeal Membrane Oxygenation (ECMO). Since 2018, the *Aspergillus* Lateral Flow Device, *Asp*LFD (OLM Diagnostics) has been validated for onsite testing in our institution to aid the diagnosis of invasive pulmonary aspergillosis. We examined the incidence of CAPA and the trend of antifungal consumption in our Critical Care Unit during COVID-19 pandemic.

**Materials & Methods:** The incidence of CAPA in our Critical Care Unit over 12 weeks from January 2021 (the peak of third wave of COVID-19 infection in Ireland) was compared to 12 weeks from March 2020 (the beginning of the first wave in Ireland). In addition to standard investigations, fungal biomarkers namely *Asp*LFD and galactomannans (GM) were performed in bronchoalveolar lavage (BAL) specimens. GM and β-D-glucan (BDG) were also performed in serum specimens. CAPA was defined by the 2020 ECMM/ISHAM criteria (modified *Asp*ICU and EORTC/MSG definitions). Antifungal consumption during these study periods in 2021 and 2020 were calculated in Defined Daily Doses per 1000 Bed Days Used (DDDs/1000 BDU) and compared to the consumption rate in 2019.

**Results:** BAL specimens were tested in 30 of 71 (42%) and 8 of 50 (16%) patients in the third wave and first wave of study period respectively. In the third wave, 6 of 71 (8.5%) patients were classified as having CAPA (5 probable and 1 possible) on the basis of positive BAL (*n* = 1) or sputum (*n* = 3) culture for *Aspergillus fumigatus*, positive BAL GM (*n* = 5) or BAL *Asp*LFD (*n* = 4). One of six patients with CAPA had co-infection with Mucormycosis. Two of six patients with CAPA required ECMO. In the first wave, one of 50 (2%) patients had possible CAPA with *Aspergillus fumigatus* isolated from one sputum culture. In the third wave, BAL *Asp*LFD had a 100% (4/4) positive predictive value and 100% (25/25) negative predictive value for CAPA when compared to BAL GM. Consumption of liposomal Amphotericin B was 3523, 361 and 222 DDDs/1000 BDU, whilst Voriconazole was 770, 141 and 191 DDDs/1000 BDU, and Caspofungin was 954, 750 and 511 DDDs/1000 BDU during the study period in 2021, 2020 and 2019 respectively.

**Conclusions:** Ireland saw its highest incidence of COVID-19 infections in the beginning of January 2021 (third wave) which coincided with the increase of Critical Care admissions and antifungal consumption. The practice of performing BAL in critically ill patients with COVID-19 in the later stage of pandemic has led to early recognition of CAPA of 8.5% with onsite *Asp*LFD testing. We found the diagnostic yield of BAL *Asp*LFD to be comparable to BAL GM and useful in informing antifungal stewardship in these high-risk patients.

## P214 Abdominal and Gastro-Intestinal Mucormycoses, 5 Years’ Experience in a University French Hospital Group

**Adela Angoulvant ^1^, Charlotte Mussini ^2^, Catherine Guettier ^3^, Nadia Anguel ^4^, Oanez Ackermann ^5^, Audrey Coilly ^6^, Charles Damoisel ^7^, Fabrice Chrétien ^8^, Alexandre Alanio ^9^ and Stéphane Jauréguiberry ^10^**

^1^ AP-HP—Université Paris Saclay, hôpital Bicêtre, Service des maladies infectieuses et tropicales, Le Kremlin-Bicêtre, France. INRAE, CNRS, AgroParisTech, GQE-Le Moulon, Gif-sur-Yvette, France
^2^ AP-HP—Université Paris Saclay, Hôpital Bicêtre, Service d’Anatomie Pathologique
^3^ AP-HP—Université Paris Saclay, Hôpital Bicêtre, Service d’Anatomie Pathologique. INSERM UMR-S 1193, France
^4^ AP-HP—Université Paris Saclay, Hôpital Bicêtre, Service de Médecine intensive et réanimation
^5^ AP-HP—Université Paris Saclay, Hôpital Bicêtre, Service d’Hépatologie pédiatrique
^6^ AP-HP—Université Paris Saclay, Hôpital Paul-Brousse, Centre Hépato-Biliaire, Inserm, UMRS 1193 Physiopathogénèse et traitement des maladies du Foie; FHU Hepatinov
^7^ AP-HP—Université Paris Saclay, Hôpital Antoine Béclère, Service de Réanimation Polyvalente
^8^ Paris Psychiatrie et Neurosciences-Université de Paris, Hôpital Sainte Anne, Service de neuropathologie, Unité Histopathologie Humaine et Modèles Animaux, INSERM, Institut Pasteur Paris
^9^ AP-HP, Nord—Université de Paris, Hôpital Saint-Louis, Laboratoire de parasitologie-mycologie. Unité de Mycologie Moléculaire, CNRS UMR2000 et Centre National de Référence Mycoses Invasives et Antifongiques, Institut Pasteur Paris
^10^ AP-HP—Université Paris Saclay, hôpital Bicêtre, Service des Maladies Infectieuses et Tropicales, INSERM 1018 CESP Centre de Recherche en Epidémiologie et Santé des Populations

**Background:** Abdominal and gastro-intestinal (GI) localizations of mucormycoses are less frequent than pulmonary or rhino-orbito-cerebral one’s. Their diagnostic remains a challenge due to the nonspecific clinical presentation especially when they occur in non-neutropenic-patients. We describe here four cases diagnosed in our hospital during the 2016–2020 period.

**Methods & Materials:** We conducted a retrospective study on mucormycosis diagnosed in our tertiary care hospital during the 2016–2020 period. Our hospital includes kidney and liver transplantation, medical and surgical intensive care, diabetology and tropical and infectious diseases departments. The clinical and mycological data were retrieved from medical and biological charts.

Mycological identification was done by morphology and MALDI-TOF and/or ITS sequencing PCR when culture was available and by PCR and/or immunohistochemistry on formalin-fixed paraffin-embedded tissue. Mucorales qPCR was performed on blood samples when available.

**Results**. Among 12 proven cases of mucormycosis identified during the study period, 4 presented with abdominal or gastro-intestinal localization. One case (hepatic) was diagnosed in a 7-month-old child (patient 1) and the three others in adult patients: 1 intra-abdominal in a female 56 years old (patient 2) and 2 gastric, in 2 males, 50 years (patient 3) and 66 years old (patient 4).

Underlying diseases were: liver transplant for biliary cirrhosis (patient 1), liver transplant for NASH and corticosteroid induced diabetes (patient 2), acute respiratory distress syndrome (ARDS) due to *Legionella pneumophila infection* and alcoholism (patient 3) and ARDS due to *SARS Cov2* (patient 4). Patients 2, 3 and 4, had abdominal pain and distension and gastric bleeding (patients 3 and 4) or intestinal necrosis (patient 2).

Mucormycoses was not suspected at the time of diagnosis, that was initially made by histology in 3 cases (patients 1, 3 and 4) and by culture at the bacteriology laboratory (patient 2).

In patient 2, retrospective direct examination, of the remaining samples (ascites fluid and intra-abdominal hematoma) performed by the mycologist showed fungal hyphae consistent with Mucorales. Mucorales qPCR performed on blood samples of patients 1, 3 and 4 were positives for *Mucor/Rhizopus. Rhizopus microsporus* was the causative agent for patient 2 and 4 and *Rhizopus* spp. for patient 1 and 3.

One patient (1), died before diagnosis and 2 (2 and 4) died within hours of diagnosis. Patient 3 received isavuconazole (due to renal compromise) for 84 days and had total gastrectomy. He died 8 months later of digestive hemorrhage.

**Conclusions:** Mucormycosis is a rare but deadly fungal infection, probably due to the low number of patients with hematological malignancy or with uncontrolled diabetes mellitus. However, it is to note that 1/3 of mucormycoses during the five last past years, had digestive localization and half occurred in patients without classical risk factors but both hospitalized in ICU. These data highlight the importance of early suspicion of fungal infection, in patients at risk but also those with ventilation/nasogastric tube, having fever, abdominal distension and pain moreover with GI bleeding. Mycological diagnostic on invasive samples and Mucorales qPCR performed on blood samples in specific contexts, should help for quick diagnosis of, mucormycosis before histology.

## P215 COVID-19-Associated Pulmonary Aspergillosis (CAPA): Incidence, Potential Risk Factors and Outcome in a Multicentre, Observational Cohort Study

**Nico Janssen ^1,2^, Rémy Nyga ^3^, Lore Vanderbeke ^4,5^, Cato Jacobs ^4^, Mehmet Ergün ^1,6^, Jochem Buil ^1,6^, Karin van Dijk ^7^, Josje Altenburg ^8^, Catherine Bouman ^9^, Hans van der Spoel ^10^, Bart Rijnders ^11^, Albert Dunbar ^11^, Jeroen Schouten ^12^, Katrien Lagrou ^5,13^, Marc Bourgeois ^14^, Marijke Reynders ^15^, Niels van Regenmortel ^16^, Lynn Rutsaert ^16^, Piet Lormans ^17^, Simon Feys ^17^, Yves Debavaye ^18^, Fabienne Tamion ^19^, Damien Costa ^20^, Julien Maizel ^3^, Hervé Dupont ^21^, Taieb Chouaki ^22^, Saad Nseir ^23,24^, Boualem Sendid ^24,25^, Roger Brüggemann ^1,26^, Frank van de Veerdonk^1,2^, Joost Wauters ^4,5^ and Paul Verweij ^1,6^**

^1^ Radboudumc—CWZ Center of Expertise for Mycology
^2^ Department of Internal Medicine, Radboud University Medical Center
^3^ Department of Medical Intensive Care, Amiens University Hospital
^4^ Medical Intensive Care Unit, University Hospitals Leuven
^5^ Department of Microbiology, Immunology and Transplantation, KU Leuven
^6^ Department of Medical Microbiology, Radboud University Medical Center
^7^ Department of Medical Microbiology, Amsterdam University Medical Centers, location VUmc
^8^ Department of Pulmonology, Amsterdam University Medical Centers, location AMC
^9^ Department of Intensive Care Medicine, Amsterdam University Medical Centers, location AMC
^10^ Department of Intensive Care Medicine, Amsterdam University Medical Centers, location VUmc
^11^ Department of Internal Medicine, Erasmus Medical Center
^12^ Department of Intensive Care Medicine, Radboud University Medical Center
^13^ Clinical Department of Laboratory Medicine and National Reference Center for Respiratory Disease Pathogens and National Reference Center for Mycosis, University Hospitals Leuven
^14^ Department of Intensive Care, Algemeen Ziekenhuis Sint-Jan Brugge-Oostende
^15^ Department of Laboratory Medicine, Algemeen Ziekenhuis Sint-Jan Brugge-Oostende
^16^ Department of Intensive Care Medicine, Ziekenhuis Netwerk Antwerpen Campus Stuivenberg
^17^ Department of Anesthesiology and Intensive Care Medicine, Algemeen Ziekenhuis Delta
^18^ Surgical Intensive Care Unit, University Hospitals Leuven
^19^ Medical Intensive Care Unit, Rouen University Hospital
^20^ Department of Medical Parasitology and Mycology, Rouen University Hospital
^21^ Department of Anesthesiology and Critical Care Medicine, Amiens University Hospital
^22^ Department of Medical Parasitology and Mycology, Amiens University Hospital
^23^ Department of Intensive Care Medicine, Critical Care Center, Lille University Hospital
^24^ INSERM U1285, CNRS UMR 8576—Unité de Glycobiologie Structurale et Fonctionnelle, University of Lille
^25^ Department of Medical Parasitology and Mycology, Lille University Hospital
^26^ Department of Pharmacy, Radboud University Medical Center

**Objectives:** Soon after the outbreak of the coronavirus disease 2019 (COVID-19) pandemic, COVID-19-associated pulmonary aspergillosis (CAPA) was recognized as a complication in patients admitted to the intensive care unit (ICU). However, reported incidence varies due to differences in the patient populations studied and the definitions used. Therefore, we set out to investigate CAPA incidence, potential risk factors and its impact on patient outcome.

**Materials & Methods:** A partially prospective/partially retrospective observational cohort study was performed during the first wave of the pandemic in eight Dutch and Belgian ICUs (discovery cohort) and three French ICUs (validation cohort). CAPA was defined according to the European Confederation of Medical Mycology and International Society for Human and Animal Mycology (ECMM/ISHAM) classification.

**Results:** Of 519 patients included in the discovery cohort, 279/519 (53.8%) underwent required diagnostic tests/procedures for ECMM/ISHAM classification. Ultimately, 42/279 (15.1%) patients were classified with CAPA (6/279 [2.2%] proven, 32/279 [11.5%] probable, 4/279 [1.4%] possible CAPA); CAPA was excluded in 237 patients (Figure 1A). Presence of any EORTC/MSGERC host factor or systemic corticosteroid use prior to and during ICU admission were not significantly more prevalent in the CAPA *versus* CAPA excluded group. However, use of other T or B cell immunosuppressants (16.7% *versus* 5.2%, *p* = 0.014), COPD (19.0% *versus* 8.0%, *p* = 0.042) and HIV/AIDS (7.1% *versus* 0.4%, *p* = 0.011) were. Logistic regression analysis demonstrated that corticosteroid use (any dose) before or during ICU admission was not independently associated with the development of CAPA, in contrast to COPD, HIV/AIDS and use of other immunosuppressants before ICU admission. ICU mortality was 52.4% in CAPA *versus* 34.2% in patients with CAPA excluded (*p* = 0.036), yet CAPA was not independently associated with ICU mortality (adjusted odds ratio [aOR] 2.03; 95% confidence interval [95% CI] 0.94–4.37).In the validation cohort, 304 patients were included, of which 209/304 (68.8%) were classifiable. Here, 21/209 (10.0%) were classified with CAPA (all of whom with probable CAPA; Figure 1B). No significant differences between CAPA and CAPA excluded groups were found regarding presence of any EORTC/MSGERC host factor, corticosteroid or other immunosuppressant use, COPD or HIV/AIDS. In this smaller cohort, use of corticosteroids before or during ICU admission was not independently associated with CAPA occurrence, as demonstrated by logistic regression analysis, nor was use of other immunosuppressant prior to ICU admission. ICU mortality in the CAPA group was 42.9% vs. 24.9% in the CAPA excluded group (*p* = 0.115). Here, too, CAPA was not independently associated with ICU mortality (aOR 1.53; 95% CI 0.51–4.55).

**Conclusions:** CAPA affects 10–15% of classifiable patients with COVID-19 admitted to ICU. COPD, HIV/AIDS, and use of immunosuppressants other than corticosteroids are potential risk factors for CAPA development. In this study, corticosteroid use was not found to be an independent risk factor for the occurrence of CAPA.

CAPA is associated with a high ICU mortality of 43–52%.

## P216 Invasive Fungal Infections in Hospitalized COVID-19 Patients from Pakistan

**Kauser Jabeen, Nosheen Nasir, Joveria Farooqi, Syed Ahsan Ali, Syed Faisal Mahmood, Moiz Khan, Hassan Wiqar, Najia Ghanchi, Muhammad Irfan, Rumina Hasan and Afia Zafar**

Aga Khan Univeristy

**Objective:** Since the beginning of COVID-19 pandemic, invasive fungal infections (IFI) in COVID-19 patients were reported from many countries. The objective of this study is to report the frequency and clinical characteristic of IFI in COVID-19 patients from Pakistan.

**Methods:** This observational study was conducted at the Aga Khan University Hospital, Karachi, Pakistan from March 2020–April 2021. Hospitalized patients with COVID-19 were identified using admission records and IFI was identified amongst these using laboratory database. These were further verified by application of IFI criteria as defined by EORTC/MSG 2019. Patients with COVID-19 associated aspergillosis (CAPA) were diagnosed using ECMM/ISHAM criteria modified to include tracheal aspirate culture and/or Galactomannan Index (GMI) >4.5 in the possible CAPA category. COVID-19 associated candidemia (CAC) was defined by isolation of *Candida* species from one or more blood cultures. COVID-19 associated mucormycosis (CAM) was defined as updated EORTC/MSG criteria with inclusion of COVID-19 as host factor. *Pneumocystis jirovecii* pneumonia (PJP) was defined by PJP consistent clinical and radiological features and PCR positive bronchoalveloar lavage.

**Results:** During the study period a total of 3506 COVID-19 patients were admitted to the hospital. A total of 123 (3.3%) COVID-19 patients with IFI were identified. This included 78 (2.2%) CAPA patients (42 probable; 36 possible), 29 (0.8%) CAC (5 *C. auris;* 24 non-*C. auris*), 10 (0.3%) CAM (7 pulmonary; 3 rhinocerebral), 3 (0.08%) PJP and three (0.08%) cases of rare invasive fungal infections (2 *C. neoformans*; 1 *Trichosporon asahii*). Mean age of patients with IFI was 62.9 years and 67% patients were male. Outcome data was available on 117/123 patients as 6 patients left against medical advice in a critical condition. Of these 117 patients, 78 expired (66.7%). These include 52/74 (70%) CAPA patients, 17/27 (63%) CAC patients, 7/10 (70%) CAM patients and 2/3 (67%) PJP patients.

**Conclusions:** We report a rate of 3.3% IFI amongst all hospitalized COVID-19 patients at our center. Due to limited number of bronchoscopy procedures conducted during this time period we consider this rate to be an underestimate especially with respect to PJP. Additionally, apart from candidemia, other invasive *Candida* infections were also not included. We also report a higher mortality rate with IFI in our patients than global data probably due to delayed diagnosis, co-infections and limited therapeutic option.

## P217 Attributable Mortality of Candidemia at the University Hospital of Cologne from 1997—2001 before the Introduction of Echinocandins

**Yael Blankenheim^1^, Jon Salmanton-García ^1,2^, Harald Seifert ^3^, Oliver A. Cornely ^1,2,4,5^ and Philipp Koehler ^1,2^**

^1^ University of Cologne, Medical Faculty and University Hospital Cologne, Department I of Internal Medicine, Excellence Center for Medical Mycology (ECMM)
^2^ University of Cologne, Cologne Excellence Cluster on Cellular Stress Responses in Aging-Associated Diseases (CECAD)
^3^ Faculty of Medicine, Institute for Medical Microbiology, Immunology and Hygiene, University of Cologne
^4^ Clinical Trials Centre Cologne, ZKS Köln
^5^ University of Cologne, Medical Faculty and University Hospital Cologne, German Center for Infection Research (DZIF), Partner Site Bonn-Cologne

**Objectives:** The relevance of candidemia has increased over the last decades due to higher incidence rates in an aging society. Studies on amphotericin B and fluconazole have shown high attributable mortality rates of 38% and 49% in the US. Incidence rates and locational factors might have an impact on the mortality rates at the University hospital of Cologne (UHC).

**Materials & Methods:** We performed a matched case-control study including 57 patients with candidemia, hospitalized at the UHC between 1 July 1997 and 30 June 2001. Controls were matched by age, sex, admission date, treatment on intensive care unit (ICU), number of days at risk, underlying diseases, surgical procedures, and the Charlson Comorbidity Index.

**Results:** The incidence of candidemia was 3.5 per 10,000 admissions. We observed a crude mortality of 33.3% and 11.8% for cases and controls, respectively, and a 30-day mortality of 23.5% and 7.8%, resulting in an attributable mortality of 21.5% and an attributable 30-day mortality of 15.7%. Risk factors for candidemia were more frequent in cases than in controls, especially chronic cardiovascular disease (39.2% vs. 25.5%, *p* = 0.138), chronic liver disease (21.6% vs. 0%, *p* < 0.001), treatment on ICU (31.4% vs. 13.7%, *p* = 0.033) and central venous catheter (80% vs. 33% *p* < 0.001).

**Conclusions:** The attributable mortality of candidemia at the UHC between 1997 and 2001 was lower compared to studies performed in the US with a similar design. Contributing factors might be lower incidence rates and less comorbidities in our study.

## P218 Possible COVID-19-Associated Invasive Pulmonary Aspergillosis Is Associated with very High Mortality Rate

**Mihaela Lupse, Mirela Flonta, Lucia Herbel, Bogdan Dombrea and Lucian Flutur**

University Of Medicine And Pharmacy

COVID-19-associated invasive pulmonary aspergillosis (CAPA) raise concerns about this superinfection as an additional contributing factor to mortality. ECMM/ISHAM consensus criteria for proven or probable CAPA are difficult to confirm, but screening of COVID-19 patients in ICU by culture of respiratory specimens obtained via non-bronchoscopic lavage is one step for identifying of possible CAPA.

The aim of our study was to characterize critical COVID-19 patients with positive cultures of respiratory specimens for *Aspergillus* sp.

**Material and method:** a retrospective observational study based on electronic files of all patients admitted into a 20 beds ICU of our COVID-19 dedicated hospital, from 1 April 2020 until 30 April 2021, who developed positive cultures of tracheal aspirates with *Aspergillus* sp. All strains were identified with MALDI-TOF MS Bruker. We evaluated patients regarding: age, co-morbidities, CT pulmonary abnormalities, lab parameters and APACHE 2 and SOFA score upon admission to the ICU, the need for invasive or noninvasive mechanical ventilation, immunomodulatory therapies and disease outcome.

**Results:** 20 out of 401 patients (5%) hospitalized in intensive care presented positive cultures for *Aspergillus* sp (14 *fumigatus*, 3 *niger*, 1 *flavus* and 2 not identified). The average age of our patients was 71.15 years (min 36, max 88), 12 were males, and they had in average 2 co-morbidities, the most common being: hypertension, coronary heart disease, obesity and diabetes. Pulmonary abnormalities at CT scan involved in average 70% of the lung surface, 15 patients have had increased liver function tests and 3 had renal failure. The average APACHE 2 score was 16 and for SOFA 6 and the average value for serum ferritin was 1279, for IL-6 470 and for D-Dimer was 3. The average duration of hospitalization was 20 days and the mortality rate was 90% compared to 37% for patients hospitalized in the ICU, but without aspergillosis.

**Conclusions:** *Aspergillus* sp was associated with criteria for high severity in COVID-19 patients and was followed by a severe outcome, meaning that screening patients for *Aspergillus* superinfection and aggressive antifungal treatment could reduce mortality.

## P219 Molecular Investigation of an Azole Non-Susceptible *Candida parapsilosis* Outbreak in a Neonatal Intensive Care Unit

**Maria Kourti ^1^, Maria Simitsopoulou ^1^, Elias Iosifidis ^1^, Eleni Agakidou ^2^, Aggeliki Kontou ^2^, Eleni Vagdatli ^3^, Kosmas Sarafidis ^2^ and Emmanouel Roelides ^1^**

^1^ 3rd Department of Pediatrics, School of Medicine, Aristotle University of Thessaloniki, Hippokration General Hospital, Thessaloniki, Greece
^2^ 1st Department of Neonatology and Intensive Care Unit, School of Medicine, Aristotle University of Thessaloniki, Hippokration General Hospital
^3^ Microbiology Department, Hippokration General Hospital

**Objectives:** Invasive fungal infections (IFIs) in neonatal intensive care units (NICUs) constitute a substantial health problem, being the second most common cause of infection-related death among critically ill neonates. *Candida parapsilosis* has been associated with several outbreaks in NICUs. We describe the susceptibility and molecular profile of a *C. parapsilosis* outbreak in a NICU.

**Materials & Methods:** A cluster of 3 candedemia cases occurred between 1 January and 28 February 2021 in a 40-bed level III NICU. Blood specimens and perineal swabs were cultured on Sabouraud dextrose agar. Infection control measures were enhanced and active surveillance for *Candida* rectal colonization was implemented. Demographics and medical data were extracted and recorded. Isolates were identified and antifungal susceptibility testing was conducted by Vitek and EUCAST microdilution method. Genomic DNA (n = 3) was isolated using Blood & Cell Culture DNA Kit (Qiagen) and molecular typing was performed by MLST method. The gene set used to provide optimum differentiation between *C. parapsilosis, C. orthopsilosis* and *C. metapsilosis* were: SADH1 (secondary alcohol dehydrogenase), SYA1 (alanyl-tRNA synthetase), VMA (vacuolar ATPase) and ITS1-ITS4 (rRNA inter-transcribed spacers). The amplified products were subjected to bidirectional Sanger dideoxy sequencing. Obtained sequences were subjected to online searching database of NCBI and Genescript Restriction Enzyme Map Analysis.

**Results:** *C. parapsilosis* was initially detected on day of life 17 in the blood of an extremely premature infant born at 22 weeks gestational age. During the following 11 days and within an one-day time interval, two other patients developed clinical symptoms of sepsis and positive blood cultures for the specific fungus. All infants had major risk-factors for fungal infections including extreme prematurity, very low-birth weight, prolonged hospitalization, central venous catheters, total parenteral nutrition and broad-spectrum antibiotic therapy. Infants described received prompt treatment with 5 mg/kg/d liposomal amphotericin B. Although two of the cases were successfully treated, the extremely immature infant with the initial presentation succumbed within 2 days following the onset of the disease.

All isolates were resistant to fluconazole (MIC:8 μg/mL), but susceptible to amphotericin B (MIC:0.5 μg/mL). Negative rectal swab cultures suggested that mucosal colonization by *C. parapsilosis* did not precede invasive infection. The three isolates showed 99.41–99.85% homology to *C. parapsilosis* SADH1, 100% identity to *C. parapsilosis* SYA1 and ITS1-ITS4 ribosomal region. A VMA PCR product, specific to amplify *C. orthopsilosis/C. metapsilosis* DNA, was not detected. Restriction enzyme mapping of the SADH1 amplified 715bp DNA target showed one unique *Ban*I cut site, specific only to SADH1 gene of *C. parapsilosis sensu stricto*. Molecular analysis demonstrated all isolates were *C. parapsilosis sensu stricto*. Although pairwise nucleotide alignment analysis between the *C. parapsilosis* sensu stricto SADH1 and ITS1-ITS4 DNA regions for the 3 isolates showed two nucleotide changes, a clonal relationship between the isolates cannot be concluded. The spread of infection was succesfully interrupted by adopting rigorous infection control based on hand hygiene and disinfection techniques.

**Conclusions:** We molecularly describe an outbreak of *C. parapsilosis sensu stricto* that was not due to previous colonization of the infants. Enhanced surveillance and infection control interventions contained the outbreak.

## P220 No Impact of COVID-19 Associated Pulmonary Aspergillosis on Bacterial Airway Diversity in Patients Hospitalized for Acute Respiratory Distress Syndrome

**Emilie Guemas ^1,2^, Cécile Angebault ^1,3^, Xavier Iriart ^2^, Vanessa Demontant ^1^, Soulaf Suyyagh-Albouz ^1^, Slim Fourati ^1^, Paul-Louis Woerther ^1,3^, Keyvan Razazi ^1^, Béatrice Riu ^2^, Antoine Berry ^2^ and Françoise Botterel ^1,3^**

^1^ CHU Henri Mondor, APHP
^2^ CHU Toulouse
^3^ EA 7380, Dynamyc UPEC

**Objectives:** COVID-19 associated pulmonary aspergillosis (CAPA) is observed in 14 to 28% of critically-ill patients and associated with increased mortality. An hypothesis is that there are specific bacterial associations in CAPA suggesting strong trans-kingdom interactions. Here, we propose a retrospective bicentric study aiming to compare through targeted metagenomics (TM) the bacterial and fungal microbiota (BM and FM) from patients hospitalized in intensive care unit (ICU) for severe acute respiratory distress syndrome (ARDS) due to COVID-19 whether they had CAPA or not.

**Materials & Methods:** COVID-19 patients with ARDS hospitalized in ICU at Creteil or Toulouse Hospital (France) between March and November 2020 were included. Patients were classified as CAPA, controls or *Aspergillus* colonisation/contamination based on clinical, mycological and imaging criteria proposed by ECMM/ISHAM consensus for CAPA classification. Clinical, biological and radiological data of all patients were collected. DNA was extracted from respiratory samples and bacterial V3-V4 16S and fungal ITS2 regions were sequenced (MiSeqTM Illumina, V2-kit) for TM purpose. Operational taxonomic units were defined using QIIME2 and assigned to SILVA and UNITE databases. Statistical analysis was performed using Shaman. Trans-kingdom microbial network analysis was performed using permutation-renormalization bootstrap method (ReBoot, NetCoMi R Cran package).

**Results:** Fifty-three patients were included (Creteil, n = 33; Toulouse, n = 20). Twenty-one were CAPA (18 probable and 3 possible) diagnosed on positive direct examination (n = 9) culture (n = 17) *Aspergillus*-PCR (n = 15) on respiratory samples and/or positive serum galactomannan antigens (n = 7). Seven had mycological arguments that were irrelevant for CAPA classification and were considered as colonisation/contamination and 25 were controls. CAPA and controls were not significantly different regarding median age (65 and 67, respectively), sex ratio (4.2 and 5.2, respectively), length of ICU stay (29d and 23d, respectively), diabetes (57 and 52%, respectively); obesity (33 and 44%, respectively) and mortality (57 and 60%, respectively). Among CAPA, 16 received curative antifungal treatment (amphotericin B, n = 8; voriconazole, n = 9 and isavuconazole, n = 8). FM profiles differed (PCoA, Permanova, *p*-value = 0.02) between controls and CAPA, in whom *Aspergillus* were overrepresented (*p*-value < 0.001). Other than *Aspergillus*, the FM of severe COVID-19 patients was mainly composed of *Sacharomycotina* yeasts (*Candida, Nakaseomyces, Clavispora, Pichia, Meyerozyma, Saccharomyces*) and *Malassezia*. BM profiles assessed through TM were similar between CAPA and controls (*p*-value = 0.28). The main represented taxa were *Pseudomonas. Delftia. Prevotella. Stenotrophomonas. Enterobacter* and *Staphylococcus*. Trans-kingdom network correlation analysis revealed that *Aspergillus* was not involved in significant correlation patterns with bacterial or fungal taxa in CAPA and only with *Malassezia* in controls. Secondarily, we compared FM and BM according to the center: BM profiles differed significantly (*p*-value = 0.009) with a higher bacterial diversity in Creteil when FM profiles were similar (*p*-value = 0.61).

**Conclusions:** In our study comparing FM and BM of patients hospitalized in ICU for severe COVID-19 with or without CAPA, we did not find specific diversity patterns of bacterial or fungal microbiota (except from *Aspergillus* overrepresentation in CAPA) or microbial correlations between patients with CAPA and controls. The hypothesis of strong trans-kingdom interactions between *Aspergillus* and other microorganisms, especially bacteria, is still to be confirmed in further studies.

## P221 Invasive Fungal Infection in the ICU during the First Wave of the COVID-19 Pandemic

**Mathilde Chamula^1,2^, Sharon E Weinberg ^1^, Tim Felton ^1,3,5^, Caroline B Moore ^1,4^ and Riina Rautemaa-Richardson ^1,3^**

^1^ Manchester University NHS Foundation Trust, Wythenshawe Hospital
^2^ Hotel-Dieu de Quebec, CHU de Quebec—Universite Laval
^3^ Division of Infection, Immunity and Respiratory Medicine, School of Biological Sciences, NIHR Manchester Biomedical Research Centre (BRC) at the Manchester Academic Health Science Centre, The University of Manchester
^4^ Mycology Reference Centre Manchester, ECMM Centre of Excellence for Medical Mycology, Manchester University NHS Foundation Trust, Wythenshawe Hospital
^5^ Intensive Care Unit, Wythenshawe Hospital, Manchester University NHS Foundation Trust

**Objectives:** The aim was to describe the prevalence of invasive fungal infections in patients hospitalised for respiratory failure due to COVID-19 in the ICU. We also aimed to evaluate the adherence to our local Trust diagnosis guideline regarding invasive fungal infections.

**Materials & Methods:** A unicentric retrospective study was conducted between the 15 March and 3 June 2020. The records of all patients admitted to the ICU for a COVID-19 infection during the study period were reviewed.

**Results:** 70 adult patients hospitalised in the ICU for COVID-19 infection were included in the study. All 70 patients had one or more sets of blood cultures taken and 39 (56%) had one or more BDG levels measured. A total of 14 (20%) patients had *Candida* spp. reported from one or more blood culture bottles (5 patients, 7%) and/or line tips (12 patients, 17%). Of the 39 patients tested for BDG, 2 (3%) patients and 6 (4%) samples were positive. Of these 39 patients, 4 (10%) had *Candida* spp. also in blood culture. All patients diagnosed as candidaemia were treated according to the Trust guideline. One patient with blood culture positive candidaemia died despite appropriate antifungal therapy.

Of the 70 patients, 24 (34%) had sputum tested for GM. Of these, 19 (79%) had two specimens taken as per our Trust guideline. 4 patients (17% of those tested; 5% of all patients) had one or more positive sputum GM results. 3 of these 4 patients (75%) underwent further examination with CT thorax as per Trust guideline. 2 were reported with no evidence of fungal co-infection. None of these 4 patients had further investigations of their repiratory secretions such as *Aspergillus* PCR. In addition, 3 of the 4 patients with a positive GM sputum had GM measured from their serum. Serum GM was tested in total of 31 patients (44%) with no positive results. 16 patients had more than one serum GM tested. 17 of the 31 patients (55%) also had sputum tested for GM. A total of 7 patients had *Aspergillus* PCR done on their respiratory samples, 3 of these twice or more. Of these 7, 2 were not tested for GM. A total of 6 patients had evidence of possible invasive aspergillosis (4 sputum GM +, 1 sputum *Aspergillus* PCR +, 1 *Aspergillus* spp. in sputum culture). Of these, two (5%) were treated as CAPA (1 sputum GM+ and 1 *Aspergillus* spp. in sputum culture). The other four patients did not receive mould active antifungal treatment. One of the patients with positive sputum *Aspergillus* PCR who did not receive mould active antifungal treatment died.

**Conclusions:** The incidence of COVID-19 associated fungal infections in critically ill patients was significant (29%). In general, adherence to candidaemia guideline was good and diagnostic tests were used and interpreted appropriately. Nearly 40% of all patients were investigated fo CAPA, but the diagnostic pathway was not always fully followed. Interpretation of mycological investigations needs to be supported by infection specialists with expertise in this area.

## P222 A Multi-Site Evaluation of Antifungal Use in Critical Care: Implications for Antifungal Stewardship

**Clare Logan ^1,2^, Carolyn Hemsley ^3^, Amanda Fife ^4^, Jonathan Edgeworth ^3,5^, Mr Paul Wade ^3^, Anna Goodman ^3,5^, Phil Hopkins ^6^, Duncan Wyncoll ^7^, Jonathan Ball ^8^, Tim Planche ^1,2^, Silke Schelenz ^4^ and Tihana Bicanic ^1,2^**

^1^ Institute of Infection & Immunity, St. George’s University London
^2^ Clinical Infection Group, St. George’s University Hospital NHS Foundation Trust
^3^ Department of Infectious Diseases, Guy’s & St. Thomas’s NHS Foundation Trust
^4^ Infection Sciences, King’s College Hospital London
^5^ Centre for Clinical Infection and Diagnostics Research, Department of Infectious Diseases, King’s College London Guy’s & St. Thomas’ NHS Foundation Trust
^6^ Department of Critical Care, King’s College Hospital London
^7^ Department of Critical Care, Guy’s & St. Thomas’ NHS Foundation Trust
^8^ Department of Critical Care, St. George’s University London

**Objectives:** Critical care patients are at increased risk of invasive fungal infections, and subsequently Intensive Care Units (ICUs) are a setting with high antifungal consumption. Yet data on antifungal prescribing patterns and the utilisation of fungal diagnostics in critical care are lacking. The objective of this service evalaution was perform an in-depth assessment of all antifungal therapy prescriptions to inform the development of ICU-specific antifungal stewardship guidelines and services.

**Materials & Methods:** We performed a service evaluation to review all antifungal therapy (AFT) prescriptions from May-November 2019 across ICUs in 3 tertiary London hospitals, evaluating antifungal consumption, prescribing indication and rationale, appropriateness by outcome (possible, probable, proven, no invasive fungal infection), prescription review and de-escalation; diagnostics use and impact on prescribing; invasive fungal infections (IFI) diagnosis and outcome.

**Results:** 6.3% of ICU admissions were prescribed AFT, accounting for 11.44 days of therapy (DOT) per 100-occupied-bed-days (OBDs). Consumption was greatest for empirical prescribing (42%, 4.76 DOT/100-OBDs); followed by targeted (20%); prophylaxis (19%); pre-emptive (14%); and non-invasive infection (6%). Echinocandin (40%, 4.59 DOT/100-OBDs) and fluconazole (37%, 4.21 DOT/100-OBDs) use predominated. In those receiving AFT for suspected/confirmed IFI, 45% ultimately had proven/probable/possible IFI. For those with proven IFI, 50% were already on AFT at time of microbiological diagnosis. β-D-glucan (BDG) utilization and impact on prescribing practice was greatest when turn-around-time was shortest, with testing on-site. Microbiology prescription review occurred for 81% of those prescribed antifungals for suspected/confirmed IFI (median time-to-review 1[IQR 0-3] day). When AFT was started but IFI ultimately unproven, 38% stopped and 5% were de-escalated from echinocandin/amphotericin to azoles within ≤5 days.

**Conclusions:** Balancing the competing goals of limiting AFT overuse, while advocating prompt AFT initiation to improve outcomes in IFI is a challenge. While half of patients given AFT for suspected IFI ultimately had no evidence of fungal infection, an almost equal proportion with proven IFI were not on AFT until microbiological diagnosis. This illustrates the pressing need for accurate, rapid diagnostics to optimize antifungal prescribing decision-making and the importance of timely post-prescription review.

## P223 Investigation of the Value of Precipitins in Severe Acute Respiratory Syndrome Coronavirus 2 Patients with Positive Marker for *Aspergillus* species

**Anne-Pauline Bellanger, Ani Tumasyan Harikian, Jean-Christophe Navellou, Adeline Rouzet, Séverine Lallemand, Emeline Scherer and Laurence Million**

University Hospital

**Objectives:** A high prevalence of Covid-19 associated pulmonary aspergillosis (CAPA) has been reported, though difficulties to distinguish between colonization with *Aspergillus fumigatus* and infection remain. Concomitantly, several studies highlighted similarities between Severe Acute Respiratory syndrome Coronavirus 2 (SARS-CoV-2) and hypersensitivity pneumonitis (HP).^1-3^ The objective of this study was to investigate the value of precipitin assays targeting *A. fumigatus* in the management of SARS-CoV-2 patients hospitalized in Intensive Care Unit (ICU) in 2020.

**Material and Methods:** SARS-CoV-2 ICU patients were screened for fungal co-infection with *A. fumigatus* using usual fungal biomarkers for invasive aspergillosis (IA) (galactomannan antigen and *A. fumigatus* qPCR in serum) and sampling of respiratory samples (tracheal aspirates (TA), broncho-alveolar lavage fluid (BALF)). For all these patients, clinical data, residency details, ICU characteristics (Apache 2 score, qSOFA) and microbial results were collected. Electrosyneresis assays were performed using commercial *A. fumigatus* somatic and metabolic antigens (R-Biopharm). Statistical analysis was performed using R version 4.0.2 (22 June 2020).

**Results:** Our study population consisted of 65 predominantly (80%) male patients, with a median age of 69 years, a median ICU stay of 25 days and a global survival rate of 63%. Thirty-five patients had at least one positive marker for *Aspergillus* species detection (n = 32 positive culture, n = 3 positive biomarker). This group received more frequently voriconazole (*p* 0.001, Pearson’s Chi-squared test). Electrosyneresis using the somatic *A. fumigatus* antigen was significantly more frequently positive for these 35 SARS-CoV-2 ICU patients (*p* 0.04, Wilcoxon rank sum test).

**Conclusions:** Our study showed that of SARS-CoV-2 ICU patients with positive marker for *Aspergillus* species detection presented more frequently precipitins towards somatic *A. fumigatus* antigen. Precipitin assays could thus be an additional tool to assess the clinical relevance of *Aspergillus* species in respiratory samples of SARS-CoV-2 ICU patients.

**References**

1. Bellanger, A.P.; Reboux, G. Studying smoking benefit in farmer’s lung to understand Covid-19. *Occup. Med. (Lond.)* **2020**, *70*, 1–620.
2. Propper, R.E. Does cigarette smoking protect against SARS-CoV-2 infection? Nicotine Tob. Res. 2020, ntaa073.
3. Vuitton, D.A.; Vuitton, L.; Estelle Seillès, E.; Galanaud, P. A plea for the pathogenic role of immune complexes in severe Covid-19. *Clin. Immunol.* **2020**, *217*, 108493.

## P224 Comparison of Clinical Characteristics and Outcome of Invasive Pulmonary Aspergillosis in Critically Ill COVID-19 and Influenza Pneumonia Patients

**Syed Ahsan Ali, Kauser Jabeen, Hammad Niamatullah, Aisha Fareed Siddiqui, Joveria Farooqi, Safia Awan and Muhammad Irfan**

Aga Khan University, Karachi

**Objective:** Although data on influenza associated pulmonary aspergillosis (IAPA) and COVID-19 associated pulmonary aspergillosis (CAPA) have been reported from many countries, no study so far has compared the frequency, risk factors, clinical and radiological features, treatment and outcome of IAPA and CAPA patients admitted within similar time frame. We aim to determine the frequency, risk factors and outcomes of invasive pulmonary aspergillosis in patients with influenza, COVID-19 and community acquired pneumonia admitted in critical care units of a tertiary care hospital in Karachi, Pakistan.

**Methods:** A prospective cross sectional study was conducted at the Aga Khan University Hospital from November 2019–June 2020. Adult patients, of both genders, admitted in the special care or intensive care unit with pneumonia were included. Patients with hematological malignancy, neutropenia, transplant or HIV were excluded. The study was approved by the Ethical Review Committee (ERC# 2019-0847-2567). Patients were divided into three groups; community acquired pneumonia, influenza pneumonia and COVID-19 pneumonia. Invasive pulmonary aspergillosis was diagnosed as per EORTC/MSG criteria. Demographics, level of care (special care unit or ICU), comorbidities, clinical features, laboratory results including microbiological data, imaging, treatment received, complication and outcome were collected on predesigned performa.

**Results:** A total of 140 patients [70 (50%) Influenza and 70 (50%) non-influenza pneumonia] were included. Patients with non-influenza pneumonia were further divided into COVID-19 pneumonia 35 (25%) and community acquired bacterial pneumonia 35 (25%).

Of total, 20 (14.2%) patients were found to have invasive aspergillosis with 10/35 (28.5%), 9/75 (12.8%) and 1/35 (2.8%) patients in COVID-19, influenza and CAP groups, respectively. Duration of symptoms was 12.5±12.13 days in CAPA and 7.56 ± 4.0 days in IAPA patients (*p* = 0.24). Four (44.4%) vs. 3 (30%) patients with influenza and COVID-19 were hypoxic, respectively (*p* = 0.15). Except one patient with unilateral infiltrates on chest X-ray in Influenza group, all patients were found to have bilateral involvement. Mean APCHE II score was 17.4 ± 8.42 and 16.6 ± 6.27 in patients with CAPA and IAPA respectively (*p* = 0.85). Nine (90%) CAPA patients required vasopressor support compared to 3 (33%) patients in IAPA (*p* = 0.020). Seven(70%) CAPA patients required invasive mechanical ventilation compared to 4 (44%) IAPA patients (*p* = 0.37). Length of stay in hospital was highest in CAPA patients (18.3 ± 7.28 days) compared to IAPA patients (11.7 ± 5.33 days) (*p* = 0.036). Similarly, length of stay in SCU/ICU among CAPA patients was 17 ± 8.72 days compared to IAPA patients 9.4 ± 2.3 days (*p* = 0.034). The number of deaths in IAPA patients and CAPA patients was 3 (33.3%) and 5 (50%), respectively (*p* = 0.526).

**Conclusions:** A higher proportion of patients with COVID-19 developed invasive aspergillosis compared to influenza. Although the mortality rate in both CAPA and IAPA patients were comparable, CAPA patients had a significantly longer stay in hospital and in critical care units.

## P225 Impact of COVID-19 on Rates of Candidaemia in a Tertiary Hospital

**Alka Sobti, Claire Mason and Anna Goodman ^1^**

Guys and St. Thomas’ Trust

**Objectives:** Guy’s and St. Thomas’ NHS Foundation Trust (GSTT) expanded from 30 ICU beds to 187 ICU beds in response to COVID-19 in March 2020. We sought to determine if this led to an increase in cases of *Candidaemia* compared to previous years.

**Materials & Methods:** We reviewed all microbiology results from 1 January 2017–30 April 2021 at GSTT using an electronic search engine of a central database. We searched for blood samples containing *Candida* sp. (including novel nomenclature) and then ensured the data was deduplicated and non-clinical samples were removed. We reviewed drug sensitivities, type of *Candida* sp. and hospital location of sample collection.

**Results:** From 2 March and 25 May 2020 critical care services admitted 316 patients with confirmed COVID-19. Over the period January 2017 to end April 2021 202 isolates were found. Seven related to histopathology specimens and so were not analysed further and one was an ascitic tap collected in a blood culture bottle. The remaining isolates belonged to 112 patients across a 4 year 4 month period.

There were an average of 25 cases each year and a peak of 33 cases seen in 2019. During March 2020–March 2021 when 3 COVID-19 peaks occurred in London; there were 26 cases of *Candidaemia* in this 12 month period. Susceptibility to fluconazole overall was 74% across the entire period reviewed and was highest at 100% to date in January–April 2020 (82% was the highest result for a full 12 month period in 2020) and lowest at 66% in 2019. The most common hospital location for *Candidaemia* infection was intensive care, with 38% of patients overall in the 4 year 4 month period, 22% in 2017 and 65% between March 2020 and 2021. The next most common hospital locations for incidence *Candidaemia* were Gastrointestinal Surgery and Urology.

**Conclusions:** Our data does not show an increase in rates of *Candidaemia* during our local COVID-19 surge. It shows that *Candidaemia* cases were more commonly from ICU patients, reflecting the change in profile of our hospital patients as the number of ICU beds increased dramatically during 3 waves of COVID-19. Other areas of high rates of *Candidaemia* were those associated with known risk factors (GI surgery and Urology). Rates of susceptibility to Fluconazole were high in our clinical setting.

## P226 Infection Metallomics for Medical Mycology

**Radim Dobiáš and Vladimír Havlíček^1^**

^1^ Institute of Microbiology
^2^ Public Health Institute

**Objectives:** *Infection metallomics* is a mass spectrometry platform (Figure 1) we established based on the central concept that microbial metallophores are specific, sensitive, and promising biomarkers of invasive infectious diseases. In addition to our own data we will review clinical applications of metallophores in medical mycology from historical and functional perspectives (Figure 2). We will identify under-studied and emerging application areas with high diagnostic potential for the post-covid era.

**Materials & Methods:** Mass spectrometry with isotope data filtering is central to *infection metallomics*; it has been used to study the interplay between “frenemies” in hosts and to monitor the dynamic response of the microbiome to antibiotic and antimycotic therapies. During infection in critically ill patients, the hostile environment of the host’s body activates secondary bacterial, mycobacterial, and fungal metabolism, leading to the production of metallophores that increase the pathogen’s chance of survival in the host.

**Results:** Clinically relevant genera actively secreting metallophores include *Aspergillus* and *Fusarium* spp. (triacetylfusarinine C, Tafc; ferricrocin FC), *Rhizopus,* and other zygomycetes (rhizoferrin), *Trichophyton* and *Ustillago* (ferrichrome), *Scedosporium* (Me-coprogene), *Histoplasma* and *Blastomyces* (coprogens, dimerum acid). As example, for the noninvasive diagnosis of invasive aspergillosis, we used an array of TafC/FC/gliotoxin (GTX) in a cohort of 28 patients (14 positive and 14 negative controls). Limit of detection for TafC in human urine was 0.1 ng/mL. The maximum experienced concentration was 1.7 ug/mL. In the working dataset of critically ill, mostly non-neutropenic humans, the TafC/FC/GTX sensitivity in urine was 93% compared to standard GM in serum (36%). We also will show noninvasive combined monitoring of *Aspergillus fumigatus* and carbapenem-resistant *Pseudomonas aeruginosa*. The invasive coinfection was detected by monitoring levels of the respective siderophore, TafC (23 ± 1 ng/mL) and bacterial pyoverdin E, pyochelin, and 2-heptyl-4-quinolone in concentration profiles (85.3 ± 3.4, 135.1 ± 4.2 and 12.9 ± 1.2 ng/mL) recorded in the urine or condensate of a critically ill patient. Human data will supported by rat infection models with positron emission and computed tomography scanning. Partitioning between animal serum and urine, and biomarker stability studies will be provided.

**Conclusions:** Mass spectrometry can reveal the structures, stability, and threshold concentrations of metal-containing microbial biomarkers of infection in humans and model organisms, and can discriminate invasive disease from benign colonization based on well-defined thresholds distinguishing proliferation from the colonization steady state. *Pneumocystis jirovecii*, yeasts (*candidi*), yeast-like (*cryptococci*), *Paracocccidioides* spp., and other xenosiderophore-uptaking pathogenic fungi remain diagnostically challenging for infection metallomics.

## P227 Triazole Resistance Screening of Aspergillus Species Isolated from Patients with COVID-Associated Pulmonary Aspergillosis from Pakistan

**Sadaf Zaka, Joveria Farooqi, Faheem Naqvi and Kausar Jabeen**

Aga Khan University Hospital

Introduction: COVID-19 associated pulmonary aspergillosis (CAPA) emerged just a few weeks after SARS-CoV was declared pandemic. We have previously reported CAPA rates of 21.7% in the ICU from our center. First line therapy for CAPA includes voriconazole or isavuconazole but azole resistance among *Aspergillus* species in CAPA patients have been identified. Therefore, the aim of this study was to perform triazole resistance in *Aspergillus* species isolated from CAPA patients admitted at our center.

Materials & Method: *Aspergillus* isolates yielded from tracheal aspirates and sputa of CAPA patients sent to the Aga Khan University clinical laboratory from July–December 2020 were saved. All the isolates were identified phenotypically. Triazole resistance screening was performed using protocol by Mortensen et al. A four-well quad-plate containing RPMI 1640-2% glucose agar supplemented with itraconazole (4 µg/mL), voriconazole (1 µg/mL), and posaconazole (0.5 µg/mL), and no antifungal (positive-control well) was prepared. Fungal spores were suspended in normal saline (0.85%) and adjusted to 0.5 McFarland and 50 μL was plated in each well. *C. parapsilosis* ATCC 22019, *C. krusei* ATCC 6258, *A. flavus* ATCC 204304 and a triazole resistant *C. auris*, confirmed from Mycotic Diseases Branch, CDC, were used as control strains. Plates were incubated at 35–37 °C for 72 h in ambient air. The presence or absence of growth was observed visually at 24 h, 48 h, and 72 h.

**Results:** We screened 50 *Aspergillus* isolates from 33 CAPA patients for triazole resistance. Quality control strains showed expected results. Seventeen isolates (12 *A. flavus*, 3 *A. fumigatus*, and 2 *A. niger*) showed growth on only voriconazole well apart from growth control. Sixteen isolates (32%) showed growth at 72 h and only one (2%) at 48 h while 33 (60%) isolates screened negative for resistance (Table 1). MICs of these 17 strains are awaited.

**Table 1.** *Aspergillus* species distribution and their triazole resistance screening results.


**Organism**

**No Growth in All Wells at 72 h**

**Growth Only in Voriconazole Well (1 µg/mL)**

**Total**
48 h72 h
*Asp. flavus*
17 (58.62%)1 (3.44%)11 (37.93%)29
*Asp. fumigatus*
4 (57.14%)03 (42.85)7
*Asp. niger*
12 (85.71%)02 (14.28)14Total33 (60%)1 (2%)16 (32%)50

**Conclusions:** Previous studies from Pakistan evaluating azole resistance in both clinical and environmental using same methodology did not detect any azole resistance. This is first time, where we have found *Aspergillus* isolates from Pakistan that have screened positive for triazole resistance. This resistance will be confirmed by determination of MICs. However, these preliminary results emphasize the importance of periodic surveillance and monitoring for the emergence of azole-resistant clinical isolates.

## P228 A Prospective Multicentre Study of the T2 *Candida* Assay to Diagnose Invasive Candidiasis in High-Risk Patients in the Intensive/Intermediate Care Unit

**Anders Krifors ^1^, Måns Ullberg ^2^, Markus Castegren ^1^, Johan Petersson ^3^, Ernesto Sparrelid ^4^, Helena Hammarström ^5^, Volkan Özenci ^2^ and Ola Blennow ^6^**

^1^ Department of Physiology and Pharmacology, Karolinska Institutet
^2^ Department of Clinical Microbiology, Karolinska University Hospital
^3^ Department of Anesthesiology, Surgical Services and Intensive Care, Karolinska University Hospital
^4^ Department of Clinical Science, Intervention and Technology, Division of Surgery, Karolinska Institutet, Karolinska University Hospital
^5^ Department of Infectious Diseases, Sahlgrenska Academy at University of Gothenburg
^6^ Department of Infectious Diseases, Karolinska University Hospital

**Objectives:** The T2Candida magnetic resonance assay can detect *Candida* spp. directly from whole blood specimens. The result can be obtained within a few hours and, therefore, offers advantages over blood cultures (BC) that typically take a couple of days and are limited by low sensitivity. Over the past decade, efforts have been made to improve the early diagnosis of invasive candidiasis (IC) by screening protocols and novel diagnostic methods. There is scarce clinical data analysing the performance of the T2Candida panel. The present study aimed to prospectively compare T2Candida assay to standard methods including culture and *β-D-Glucan* (BDG) in a selected high-risk population.

**Materials & Methods:** A prospective multicentre study conducted at the intensive/intermediate care units (ICU/IMCU) at the Karolinska University Hospitals, Stockholm, Sahlgrenska University Hospital, Gothenburg and Västerås Hospital, Västerås. Between February 2019 and March 2021, prospective patients >18 years admitted to the ICU/IMCU related to gastrointestinal surgery or necrotizing pancreatitis were enrolled. When the attending physician requested blood cultures, simultaneous testing with T2Candida and BDG was performed. The results of both tests were made clinically available.

**Table 1.** Baseline characteristics, n = 146.

Male, n(%)82 (56)Age (years), median(IQR)64 (55–73)Renal failure, eGFR <60 mL/min10 (7)Immunosuppression, n(%)31 (21)Gastrointestinal surgery, n(%)126 (86)Necrotizing pancreatitis, n(%)20 (14)
**Clinical data at T2Candida/BDG testing**
SOFA score, ±SD3.9 ± 3.5Vasopressor treatment, n(%)54 (37)>1 Gastrointestinal surgery, n(%)27 (18)Days in intensive/intermediate care, median (IQR)3.5 (1–6)Mechanical ventilation, n(%)39 (27)Total parental nutrition, n(%)43 (29)Renal replacement therapy, n(%)15 (10)Broad spectrum antibiotic therapy, n(%)84 (58)Antifungal therapy, n(%)18 (12.3)30-day mortality, n(%)21 (14)

**Results:** In total, 146 patients were enrolled in the study (Table 1). Using the recently updated EORTC/MSG criteria, Proven IC was defined as blood culture positivity or evidence of *Candida* spp. in other normally sterile specimens. Probable IC was defined using a positive BDG at a positivity cut-off threshold of >100 pg/mL. The T2Candida assay identified 3/4 BC+ IC and was in concordance with BDG (Figure 1). The missed BC+ was drawn from a central venous catheter, but both peripheral and arterial catheter BCs were negative, suggestive of colonization. Compared to BDG, the T2Candida assay was less likely to identify proven IC based on evidence other than BC+. The overall sensitivity for proven IC was 47% and 80% for T2Candida and BDG, respectively. Only one case of probable IC was concomitantly positive in the T2Candida assay. 8/19 (42%) of probable IC cases were receiving antifungal therapy at the time of testing. The T2Candida panel presented a specificity of 98%.


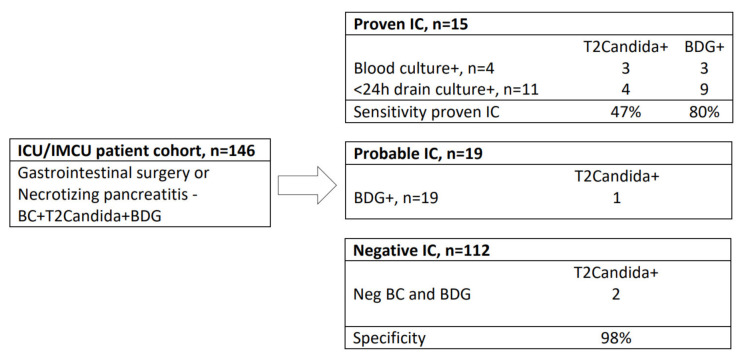


**Figure 1.** The T2Candida assay identified 3/4 BC+ IC and was in concordance with BDG.

**Conclusions:** The T2Candida assay provided excellent diagnostic accuracy in both ruling in and ruling out BC+ IC. However, IC, due to deep-seated abdominal infections, was better diagnosed using BDG. In these cases, fungemia may only be intermittently present and therefore favour the kinetics of BDG. Still, BDG is not *Candida* specific and does not provide any species information that may significantly influence treatment choice. The present data shows that the T2Candida assay is a valuable tool in the diagnosis of IC in the ICU/IMCU setting.

## P229 Broad Spectrum Azoles and Flucloxacillin: A Dangerous Match

**Tine Vangheluwe, Dirk Vogelaers, Frederik Van Hoecke, Alexander Dumoulin, Piet Lormans and Ann-Sofie Vanthournout**

Az Delta

**Objectives:** Voriconazole and high dose flucloxacillin are standard first line treatments for invasive fungal infection with *Aspergillus fumigatus* and bloodstream infection with methicillin-susceptible *Staphylococcus aureus* (MSSA) respectively. When both co-occur, an underestimated risk of a significant interaction needs to be taken into consideration.

**Methods:** Synoptic description of a significant interaction in 2 critically ill patients and analysis of interaction screening in validated data bases.

**Results:** Case 1: in a 72 year old Caucasian female with chronic lymphatic leukemia, admitted to the ICU for severe SARS-CoV-2 pneumonia with respiratory failure, BAL growth of *Aspergillus fumigatus* prompted voriconazole therapy (6 mg/kg bid loading and 4 mg/kg bid maintenance dosing). Three days later, high dose flucloxacillin was initiated because of positive MSSA blood cultures. The voriconazole level on day 10 of the combination therapy, indicated a subtherapeutic level (<0.2 µg/mL), triggering doubling of voriconazole dosing (8 mg/kg bid), whereas flucloxacillin therapy was discontinued because of a positive clinical and biochemical evolution. A second subtherapeutic voriconazole level (<0.2 µg/mL) was reported 2 days after flucloxacillin interruption, subsequently rising to a therapeutic level (3 µg/mL) after 3 days.

Case 2: A 79 year old men, with cardiac history, was admitted to the ICU for SARS-CoV-2 pneumonia with respiratory failure. A classic loading and maintenance voriconazole therapy was started and resulted in a therapeutic level (3.5 µg/mL). After three days, high dose flucloxacillin was initiated because of positive MSSA blood cultures. Two voriconazole levels were undetectable (<0.5 µg/mL). After 7 days, flucloxacillin was stopped. Two days after discontinuation of the flucloxacillin, the voriconazole level was still subtherapeutic because of the persistent effect of flucloxacillin, leading to a dose increase (6 mg/kg bid) and subsequent therapeutic voriconazole level (4.2 µg/mL), further increasing to a toxic level (15.1 µg/mL). The voriconazole therapy was continued intravenously at a maintenance dose of 4 mg/kg bid, which resulted in a therapeutic level (3.2 µg/mL).

**Conclusions:** Subtherapeutic voriconazole levels may be caused by co-administration of high dose flucloxacillin. A major proposed mechanism resides in the activation by flucloxacillin of the pregnane X receptor, which in turn induces expression of CYP3A4 and CYP2C8/9, responsible for the metabolisation of voriconazole ^(1,2)^. Other pathways are being explored ^(3)^.

The need for therapeutic drug monitoring is well established because of non-linear voriconazole pharmacokinetics, narrow therapeutic-toxic margin, inter-individual variability of the expression of cytochrome enzymes responsible for metabolisation and potential for interactions as well as the clinical experience of sub-therapeutic levels at the ICU. We must also take into account the sustained effect of induction after discontinuation of the therapy that induced CYP enzymes.

Clinical pharmacists play a significant role in the multidisciplinary management of serious infections and to this purpose rely on a number of validated databases ^(4,5,6)^. At the time of the clinical cases these databases did not mention this significant interaction. Furthermore, this interaction is not mentioned in the SmPC neither for voriconazole nor for flucloxacillin. However, there are a number of literature reports warning for this adverse event ^(1,2)^. We call for a peer review system guaranteeing updating of different databases.

## P230 *C. auris* Outbreak in COVID-19 Intensive Care Unit

**Buket Erturk Sengel ^1^, Elif Tukenmez Tigen ^1^, Elvan Sayin ^2^, Aytan Sevdaliyeva ^1^, Nilgun Cerikcioglu ^2^, Ismail Cinel ^3^, Volkan Korten ^1^ and Zekaver Odabasi ^1^**

^1^ Infectious Disease and Clinical Microbiology, Marmara University School of Medicine
^2^ Medical Microbiology, Marmara University School of Medicine
^3^ Anesthesiology and Reanimation, Critical Care, Marmara University School of Medicine

**Objectives:** *C.auris* is a highly virulent fungus that causes healthcare-associated infections. Since it causes outbreaks in healthcare settings and is resistant to many antifungals, early identification and infection prevention and control (IPC) measurements are crucial. Prolonged stays in the intensive care unit (ICU) and using immunosuppressive agents and broad-spectrum antibiotics may contribute to infection during the COVID-19 treatment period. This report describes *C. auris* outbreak in patients infected with COVID-19 in ICU.

**Materials & Methods:** Between March and May 2021, *C. auris* was detected in 16 patients infected with COVID-19 in ICU. Real-time polymerase chain reaction results of all patients except Case 2 were positive. *C. auris* was cultured in blood, urine, and body swabs of 5, 2, and 9 patients. Yeast colonies produced from clinical specimens of patients were identified as *C. auris* with an identification score of 99.9% using MALDI-TOF MS (VITEK MS, V3.0, BioMérieux).

**Results:** All patients were over 50 and had a history of using broad-spectrum antibiotics and steroids. The most common comorbidity was hypertension (63%). The demographic and clinical characteristics of the patients were summarized in Table 1. Although seven patients had no blood culture positivity, they were accepted infection due to clinical deterioration. The patients to be though infected with *C. auris* were started anidulafungin. All patients died except Case 10 and 11 in 30 days. The cultures of both were accepted as colonization and not started antifungal treatment.

**Conclusions:** Both *C.auris* and COVID-19 present a serious global health threat worldwide, and the IPC measures are essential. The break the chain of IPC in especially COVID-19 ICUs, cause rare and fatal infections such as *C. auris*. While the healthcare personnel is trying to protect themselves, they may not be paying attention to hand hygiene or contact isolation measurements. Therefore, to prevent these outbreaks in the near future, more stringent infection control measures are required. Our report is remarkable because of being the first *C. auris* series documented both in COVID-19 patients and Turkey.

**Table 1.** Characteristics of patients.



**Case**

**Age**

**Sex**

**Comorbidities**

**Antibiotic**

**Usage**

**Site**

**Infection/**

**Colonization**

**Treatment**

**Outcome**
180fHT, DMP/M/C/LbloodinfectionADFexitus275fHT, ischemic CVEAMP/M/Fswabcolonization-exitus382fHT, HL, CADM/LswabcolonizationADFexitus472fHT, CRDAMP/FbloodinfectionADFexitus560mHT, CAD, IPFAMP/M/VswabcolonizationADFexitus673mCOPDM/CswabcolonizationADFexitus754m-P/M/C/LswabcolonizationADFexitus872mHT, CLLM/C/LswabcolonizationADFexitus961m-M/C/Furinecolonization-exitus1062m-AMP/Purinecolonization-survive1162mHT, DM, obesityM/C/Fswabcolonization-survive1266mHT, obesityP/M/CbloodinfectionADFexitus1365fHT, DM, CADP/M/C/F/LbloodinfectionADFexitus1459fHT, DM, COPDP/M/C/LbloodinfectionADFexitus1563m-P/MswabcolonizationADFexitus1654m-M/C/VswabcolonizationADFexitus
ADF; anidulafungine, AMP; ampicillin sulbactam, CAD; coronary arter disease, CLL; chronic lymphocytic leukemia, C; colistin, COPD; chronic obstructive pulmonary disease, CRD; chronic renal disease, CVE; cerebrovascular event, DM; diabetes mellitus, f; female, F; fluorochinolone, HL; hyperlipidemia, HT; hypertension, IPF; idiopatic pulmonary fibrosis, L; linezolid, m; male, M; meropenem, P; piperacillin tazobactam, V; vancomycin.


## P231 Raised Serum Galactomannan Levels as a Predictor of Mortality in COVID-19-Associated Pulmonary Aspergillosis Patients from Tertiary Care Hospital in Pakistan

**Moiz Khan, Joveria Farooqi, Kauser Jabeen and Afia Zafar**

Aga Khan University

**Objective:** The objective of this study was to evaluate role of raised serum galactomannan levels as a predictor of mortality in COVID-19 associated pulmonary aspergillosis (CAPA) patients.

**Materials and Methods:** We conducted a retrospective observational study in COVID-19 patients admitted at the Aga Khan University Hospital from March 2020 to April 2021. Patients were characterized as CAPA cases using the updated EORTC/MSG (2020) and ECMM/ISHAM (2020) criteria. Information regarding age, gender, co-morbidities, disease severity, galactomannan index (GMI), administration of steroids and tocilizumab, duration of stay in hospital and intensive care unit, administration of steroids and tocilizumab, patient outcome viz. discharge or death, and duration of follow up, was obtained from the patient care inquiry database. Statistical significance of the collected data was assessed using the *t*-test and Kruskal-Wallis H test.

**Results:** A total of 75 patients with CAPA were identified. Of these 93.3% (n = 70) were characterized as severe and 6.7% (n = 5) as moderate COVID. Overall mortality was seen in 68% (n = 51) CAPA patients. Serum GMI >0.5 was observed in 61.3% (n = 46) CAPA patients. Of the 51 expired patients, 35 (68.6%) had positive GM and 16 (31.4%) had negative GM values (*p* = 0.062). Furthermore, mortality was seen in 25 patients with diabetes mellitus (*p* = 0.28), 13 with chronic kidney disease (*p* = 0.21), 4 with lung disease (*p* = 0.52) and 1 with liver disease (*p* = 0.82). Severity of COVID and increased length of ICU stay were significantly associated with mortality (*p* = 0.025 & *p* = 0.036). Furthermore, mortality was seen in 43 patients who received steroid therapy and 16 patients who were given tocilizumab (*p* = 0.13 & *p* = 0.6).

**Conclusions:** In our cohort of CAPA patients, increased mortality was seen in patients with raised GM value although this association did not reach statistical significance. Increased length of ICU stay and severity of disease were significantly associated with mortality in our patients. Further studies are required on a large and multi-institutional scale to fully assess the role of GM in predicting mortality in CAPA patients.

## P232 COVID-19-Associated Invasive Candidiasis (CAIC): The Results of Prospective Multicenter Study in Russia

**Olga Kozlova ^1^, Denis Gusev ^3^, Alexandr Rysev ^2^, Sofya Khostelidi ^1^, Maria Vashukova ^3^ and Nikolay Klimko^1^**

^1^ North-Western State Medical University named after I.I. Mechnikov
^2^ St. Petersburg Research Institute named after I.I. Janelidze
^3^ Clinical Infectious Disease Hospital named after S.P. Botkin

**Background:** Patients hospitalized for COVID-19 are at risk for healthcare-associated infections, including bloodstream infections caused by Candida spp. Publications about CAIC are limited.

**Aim:** To study of CAIC in actual clinical practice in Russia.

**Methods:** The prospective multicenter study in 2020–2021 yy. For diagnosis of invasive candidiasis we used criteria EORTS/MSGERC, 20;19.

**Results:** In the study were included 19 patients with CAIC, men—58%, median age —63 ÷ 13.1 (37–90) y. The median time from detection of Sarc-Cov-2 to to diagnosis of CAIC was 15 ÷ 15.1 (0–52) days. The risk factors were antibacterial drugs—100%, systemic corticosteroids—100%, CVC—100% (median time—8.5 ÷ 5.9 (0–17) days), mechanical ventilation—100% (median time—12.5 ÷ 6 days), lymphopenia—63%, hypoalbuminemia—63%, blood transfusions—37%, anti-IL6—26%, hemodialysis—26%, and surgery in the last 2 weeks—26%. The candidemia was in 100% patients, bacteremia—74%. Signs and symptoms were non-specific: fever—63%, hypothermia—10%, renal failure—58%, and liver failure—26%. The median SOFA score at the start of candidemia was 10. The main etiology agents were *Candida albicans* (53%), *C. auris* (10%), and *C. glabrata* (5%). All patients received antifungal treatment: fluconazole—68.7%, voriconazole—18.7%, amphotericin B—12.5%, and anidulafungin—6%. The median time in the intensive care unit was 25 ÷ 17.2 (9–60) days. The 30 days overall survival rate was 43.7%.

**Conclusions:** The median time from detection of Sarc-Cov-2 to diagnosis of invasive candidiasis was 15 (0–52) days. The main risk factors were antibacterial drugs—100%, systemic corticosteroids—100%, CVC—100% (median time—8.5 days), mechanical ventilation—100% (median time—12.5 days), lymphopenia—63%, and hypoalbuminemia—63%. The candidemia was in 100% patients. The median SOFA score at the start of candidemia was 10. The main etiology agents were *Candida albicans* (53%), *C. auris* (10%), and *C. glabrata* (5%). All patients received antifungal treatment, the 30 days overall survival rate was 43.7%.

## P233 Candidemia in Hematological Malignancies and Intensive Care Patients

**Iftihar Koksal and Hulya Iren Guvenc**

Acibadem University

**Objective:** Invasive fungal infections are common life-threatening disease and major cause of morbidity, particularly in patients with hematologic malignancies and patients of intensive care, and *Candida* spp. is the most common isolated fungi in bloodstream. In this study, Candida species, risk factors and comorbidities were investigated retrospectively among patients with hematological malignancies and intensive care unit.

**Methods:** This study conducted in Acibadem University Medical Faculty Atakent Hospital. Between 2019 and 2020, hospital data were retrospectively analyzed and recorded. The patients’ age, gender, primary diseases, comorbidities, and development time of candidemia were evaluated.

**Results:** Acute leukemia, lymphoma, or myelodysplastic syndrome are the most common hematological malignancies associated with candidemia, while among ICU patients solid tumors and transplant has the majority of candidemia cases. Our results show that non-*albicans* candidemia is dominant among hematological malignancy patients and intensive care patients. While all patients with hematologic malignancies had non-*albicans* candidemia, this rate was 72.5% in intensive care patients. *Candida glabrata* was the most common isolate in both group. (Table). The results are summarized in the table.



**Hematological Malignancies Patients (n:9)**

**ICU Patients (n:29)**
Age52.5 (36–72)58.3 (33–74)Gender (M)66.6%65.5%
Primary diagnosis Primary diagnosis Acute leukemia44.4%Solid organ malignancy41.3%Lymphoma22.2%Transplantation13.7%Myelodysplastic syndrome33.3%Diabetes Mellitus6.8%
Cerebrovascular disorders10.3%Others27.5%Day of hospital stay until candidemia (mean)22.7 days (11–34 days)22.8 days (7–95 days)
*C. albicans*
0%27.5%
*Non-*albicans Candida**
100%72.5%
*C. glabrata*
33.3%27.5%
*C. krusei*
22.2%6.8%
*C. tropicalis*
22.2%6.8%
*C. parapisilosis*

3.4%
*C. kefyr*

17.2%
*C. famata*
11%

*C. dubliniensis*
11%6.8%
*C. lypolytica*

3.4%

**Conclusions:** Candidemia is emerging an important problem in intensive care unit patients and patients with hematological malignancies. Non-*albicans Candida* species are the major cause of candidemia, as determined in our study.

## P240 Local Experience Feedback on Performing Routinely the PCR Mucorales on Serum Samples in At-High Risk Patients

**Anne Pauline Bellanger, Ana Berceanu, Emeline Scherer, Etienne Daguindeau, Yohan Desbrosses and Laurence Millon**

University Hospital

**Objectives:** Our center has been applying a strategy including the early detection of circulating fungal DNA using *Aspergillus* and *Mucorales* qPCR (in-house technique) for diagnosis of invasive mold disease (IMD) in athigh risk patients (induction, GVHD, HSCT, feverish aplasia, solid organ transplant) and in patients with suspicion based on imagery. The aim of this study was to assess this strategy for mucormycosis diagnosis in the past four years (2017–2020).

**Methods & Materials:** A total of 7595 fungal qPCRs were performed in 1603 patient hospitalized in our center between January 2017 and December 2020. Clinical data were collected for the patients with positive *Mucorales* qPCR on serum (at least 2 consecutive positive samples, or one sample with Cq ≤ 40).

**Results:** Thirty-nine patients (0.02%) had positive serum Mucorales qPCR. Six patients were classified according to MSG/EORTC criteria as proven (n = 1) or probable mucormycosis (n = 5). For the 33 other patients, they were considered as “mucormycosis PCR only” cases, as *Mucorales* serum qPCR is not included as microbial MSG/EORTC criteria yet.

Considering the 33 “mucormycosis PCR only” cases, a majority were male (70%), the median age of the cohort was 57 year-old (from 18 to 74) and 54.5% were alive 3 months after the diagnosis n = 18). Most of these patients were diagnosed with acute myeloid leukemia (AML) (60.6%), 80% of them were HSCT recipients. The positive *Mucorales* qPCR was followed by immediate prescription of liposomal amphotericin B except in two cases (palliative situations). In ≤10 days, 76% of the patients had negative *Mucorales* qPCR (n = 25) and 52% were alive 3 months post IMD diagnosis (n = 13). In 8 cases (24%), a mixed IMD was diagnosed (with concomitant positive galactomannan detection and/or positive *Aspergillus fumigatus* qPCR, and even once positive blood culture for *Fusarium solani*) which would have led to voriconazole treatment alone (50% of the patients with mixed IMD were alive 3 months post IMD diagnosis).

**Conclusions:** The MSG/EORTC guidelines were revised in 2019 and include now the serum Aspergillus qPCR for invasive aspergillosis diagnosis. However, serum qPCR is not yet recognized as a microbial criteria for mucormycosis. Currently, commercial kits are available for *Mucorales* qPCR so, more and more centers will be able to use locally this tool to screen patients for mucormycosis. Recognizing molecular biology in serum for mucormycosis as a microbial MSG/EORTC criteria would facilitate classification and epidemiological studies. In clinical setting, positive Mucorales qPCR on serum help prompt initiation of appropriate antifungal treatment.

## P242 Effect of Invasive Aspergillosis on Different Causes of Death in Older Patients with Acute Myeloid Leukaemia or High-Risk Myelodysplastic Syndrome

Rebecca van Grootveld ^1^**, Valentina Masarotto ^2^, Peter A. von dem Borne ^1^, Nicole M.A. Blijlevens ^3^, Dana A. Chitu ^4^, Martha T. van der Beek ^1^, Marta Fiocco ^1,2^ and Mark G.J. de Boer ^1^**

^1^ Leiden University Medical Center
^2^ Mathematical Institute, Leiden University
^3^ Radboud University Medical Center
^4^ Erasmus University Medical Center

**Objectives:** Study objectives were to estimate the cumulative incidence for different causes of death (CODs) and to investigate the effect of invasive aspergillosis (IA) on different CODs in older patients with acute myeloid leukaemia (AML) or high-risk myelodysplastic syndrome (MDS) included in the Haemato-Oncology Foundation for Adults in the Netherlands (HOVON) 43 randomized controlled trial (2000–2006).

**Materials & Methods:** Collected data from the trial was obtained from the HOVON registry and clinical information about diagnosis, treatment, survival, COD and occurrence of IA was extracted. At the time, chemoprophylaxis to prevent filamentous fungal infections was not standard practice. A selection was made of baseline patient characteristics before start of therapy to investigate the effect of these prognostic factors on different CODs (Table 1). In the dataset COD was grouped into the following categories: leukaemia, pneumonitis, infection, haemorrhage, veno-occlusive disease, secondary malignancy, other COD or unknown. Thereafter, for modelling purposes, different CODs were grouped together: infection and other COD, because other COD included 12/70 patients with infection as COD as well (Table 1), as well as pneumonitis, haemorrhage, veno-occlusive disease, secondary malignancy and unknown (‘miscellaneous’). A competing risk model was applied to estimate the cumulative incidence of death with the competing events ‘leukaemia’, ‘infection and other COD’ and ‘miscellaneous’. A cause specific hazard Cox regression model was used to investigate the effect of IA and the baseline patient characteristics on different CODs.

**Results:** In total, 806 patients were included in the final analyses. The mean age at time of diagnosis was 70 years (range 61–88) and 441 (55%) were male. Most patients died of leukaemia 380 (53.4%), followed by infection and other COD 216 (30.4%), or miscellaneous 114 (16%). The cumulative incidences for death due to leukaemia or infection and other COD at 3, 6, 12 and 36 months were 0.06–0.11–0.23–0.42 and 0.17–0.19–0.22–0.25 respectively and are depicted in Figure 1. A total of 171 patients (21%) developed IA during follow-up, of whom 40 recovered. The remaining 131 patients had IA up until in their final chemotherapy cycle and of these patients 121 (92.3%) died. Diagnosis of IA up until the final chemotherapy cycle was associated with an increased risk of dying from leukaemia cause-specific hazard ratio (_CS_HR): 1.75, 95% CI 1.34–2.28 and a trend was observed for infection or other COD (_CS_HR: 1.36, 95% CI 0.96–1.91) (Table 1).

**Conclusions:** The probability of dying from an infection or other COD was high compared to other CODs in the first year of diagnosis and patients with IA had an increased risk of dying from leukaemia and infection or other COD.

## P243 Case Report: Acute Disseminated Candidiasis with Skin and Muscular Lesions

**Anne-Pauline Bellanger, Ana Berceanu, Anne-Sophie Brunel, Tony Labaigt, Etienne Daguindau and Laurence Millon**

University Hospital

**Context:** *Candida tropicalis* is the *Candida* strain that is mostly involved in case of acute disseminated candidiasis.^1^ We report here a case of acute disseminated candidiasis with whole body dissemination (pulmonary, cutaneous, muscular, hepatic, spinal and cerebral) highlighted by impressive imagery obtained by PET scanner (Figure 1A–C).

**Description of the case report:** A 37 year-old man was diagnosed with an Acute Lymphoid Leukemia (ALL). The induction regimen begun at D0. At D10, a blood culture was positive with *Candida tropicalis* and an antifungal treatment with echinocandins was begun at D12. The antifungal treatment was switched for fluconazole per orally on D20. At D35, a 2^nd^ blood culture was positive with *C. tropicalis* so the antifungal treatment was changed again for echinocandins In between the 2 blood cultures, the patient was in intense pain from D14 to D20. Steroids, part of the chemotherapy plan, were administered from D7 to D21.

At D38, both CT-thorax imagery and PET scanner showed pulmonary and muscle dissemination in both legs and arms of the patients (Figure 1A). Cutaneous lesions also appeared and were positive to *C. tropicalis* in culture.

Susceptibility testing towards antifungals was performed for the successive isolated *C. tropicalis* strains and the Minimal Inhibitory Concentrations (MIC) did not change over time; all the strains were susceptible to echinocandins and fluconazole.

At D53, an MRI showed a cerebral lesion and, at D58 a novel MRI showed spinal dissemination (Figure 1B).

At D65 a novel PET scanner showed increased dissemination (pulmonary, cutaneous, hepatic, and spinal) (Figure 1C).

At D70, the antifungal treatment was changed for liposomal amphotericin B.

From this date, the patient improved steadily to reach normality on imagery at D130. The chemotherapy was followed all along despite the concomitant fungal infection. After D130 the patient received fluconazole per orally only.

**Conclusions:** The fact that echinocandin treatment did not stop the dissemination in the 1st phase of the infection may be explained by several facts: the initial high fungal load, the steroids administered for pain and also the typical lack of CNS penetration of echinocandins. Liposomal amphotericin B was finally prescribed but this treatment is associated with drug-drug interactions, especially with the chemotherapy regimen, that is why it was avoided as long as possible.

**References**

1. Guarana, M.; Nucci, M. Acute disseminated candidiasis with skin lesions: a systematic review. *Clinical Mircob. Infect*. **2018**, 246–250.

**Figure 1.** Illustration of the dissemination from D38 (A) to D53-58 (B) and D65 (C).

## P244 Antifungal Treatment Changes Are Frequently Required in Patients Treated for an Invasive Mold Infection

**Romain Samuel Roth ^1^, Stavroula Masouridi-Levrat ^2^, Federica Gianotti ^2^, Anne-Claire Mamez ^2^, Veronique Erard ^3^, Emmanouil Glambedakis ^4^, Frederic Lamoth ^4^, Pierre-Yves Bochud ^4^, Stephane Emonet ^5^, Arnaud Riat ^6^, Adrien Fisher ^6^, Christian Van Delden ^1^, Laurent Kaiser ^1^, Yves Chalandon ^2^, Dionysios Neofytos ^1^**

^1^ Division of Hematology, Bone Marrow Transplant Unit, University Hospital of Geneva and faculty of Medicine, University of Geneva, Geneva, Switzerland
^2^ Division of Hematology, Bone Marrow Transplant Unit, University Hospital of Geneva and faculty of Medicine, University of Geneva, Geneva, Switzerland
^3^ Division of Infectious Diseases, Cantonal Hospital of Fribourg, Fribourg, Switzerland
^4^ Division of Infectious Diseases, University Hospital of Lausanne, Lausanne, Switzerland
^5^ Division of Infectious Diseases, Cantonal Hospital of Sion, Sion, Switzerland
^6^ Laboratory of Bacteriology, University Hospital of Geneva, Geneva, Switzerland

**Objectives:** There are limited data to describe end-of-treatment (EOT) parameters of antifungal therapy for invasive mold infections (IMI).

**Methods:** We performed a 10-year cohort study of all adult (≥18-year-old) allogeneic hematopoietic cell transplant recipients with proven/probable IMI to describe IMI treatment duration and patient profile at treatment discontinuation. Only patients who had received a minimum of 84 days of antifungal treatment and were alive on day 84 post IMI diagnosis were included.

**Results:** Eighteen of 61 (29.5%) and 2/61 (3.3%) patients with IMI had died and discontinued treatment before day 84 post-IMI diagnosis, respectively. The remaining 41 (67.2%) patients were included for further analyses, with 14 and 29 proven and probable IMI, respectively. There were 30 (73.2%) and 11 (26.8%) patients with invasive aspergillosis (IA) and non-IA IMI (6-mucormmycosis, 5-other), respectively. Mean treatment duration was 360 days (range: 87, 2016, median: 246, IQR: 167–355): 340 and 415 days for IA and non-IA IMI, respectively (*p* = 0.55). By EOT, 14 (51.8%) and 13 (18.2) patients had complete/partial response and stable/deterioration of radiographic imaging, respectively. Twenty-eight (68.3%) patients were receiving steroids by EOT (18/28, 64.3% at prednisone dose equivalent >20 mg/day). Thirty-two (78%) patients were receiving immunosuppressive therapy with at least one of the following agents: tacrolimus (17/41, 41.5%), cyclosporine (15/41, 36.6%), mycophenolate mofetil (14/41, 34.2%), methotrexate (1/41, 2.4%). Laboratory values at EOT are shown in Table 1. Considering the mean treatment duration of 360 days, we divided patients in two groups: those who had died and those who were still alive by 365 days post-IMI diagnosis. The latter group would represent a patient group on which treatment discontinuation would be less likely due to death, hence variables leading to the clinical decision for treatment discontinuation could be more accurately described. Patients who were alive by 1-year post-IMI diagnosis were more likely to have higher lymphocyte, platelet and immunoglobulin counts and less hepatotoxicity, compared to patients who had died during the first year of IMI diagnosis (Table 1). There was a trend for patients still alive by 1-year post-IMI diagnosis to have discontinued steroid treatment (10/23, 43.5%), compared to patients who had died during the first year of IMI diagnosis (3/18, 16.7%; *p* = 0.07).

**Conclusions:** In this real-life cohort study, antifungal treatment for IMI was long and discontinued with bone marrow recovery and decrease of immunosuppression.

## P245 Breakthrough Invasive Fungal Infection among Patients with Hematological Malignancies: A National Prospective Multicenter Study

**Pedro Puerta Alcalde ^1^, Cecilia Martín-Gandul ^2^, Juan Carlos Ramos ^3^, Júlia Laporte-Amargós ^4^, Marina Machado ^5^, Pilar Martín ^6^, Mireia Franch-Sarto ^7^, Isabel Sánchez-Romero ^8^, Jon Badiola ^9^, Lucia Gomez ^10^, Isabel Ruiz ^11^, Lucrecia Yáñez ^12^, Lourdes Vazquez ^13^, Celia Cardozo^1^, Francesc Marco ^1^, Alex Soriano ^1^, Ana Alastruey-Izquierdo ^14^, Pedro González ^9^, Ana Fernández-Cruz ^8^, Montserrat Batlle ^7^, Jesús Fortún ^6^, Jesús Guinea ^5^, Carlota Gudiol ^4^, Julio Garcia ^3^, Manuela Aguilar-Guisado ^2^ and Carolina Garcia-Vidal ^1^**

^1^ Hospital Clínic De Barcelona
^2^ Hospital Universitario Virgen del Rocío
^3^ Hospital Universitario La Paz
^4^ Hospital Universitari de Bellvitge
^5^ Hospital General Universitario Gregorio Marañón
^6^ Hospital Universitario Ramón Y Cajal
^7^ Hospital Germans Trias i Pujol
^8^ Hospital Universitario Puerta De Hierro
^9^ Hospital Universitario Virgen de las Nieves
^10^ Hospital Universitari Mútua Terrassa
^11^ Hospital Universitari Vall d’Hebron
^12^ Hospital Universitario Marqués de Valdecilla
^13^ Hospital Universitario de Salamanca
^14^ Instituto de Salud Carlos III. Centro Nacional de Microbiología

**Objectives:** We aimed to describe the current epidemiology and outcomes of breakthrough invasive fungal infections (BtIFI) in hematological patients in Spain.

**Materials & Methods:** BtIFI occurring in patients with ≥7 days of antifungals were prospectively diagnosed (36 months—13 hospitals) according to revised EORTC definitions. Some antifungals were defined as resistant despite the lack of specific breakpoints when: (i) the fungus was considered intrinsically resistant; (ii) the MIC would be considered resistant to a similar fungus (e.g., Candida orthopsilosis with *C. parapsilosis*).

**Results:** 124 episodes of BtIFI were documented. Mean age (IQR) of patients was 58 (47–64) years and 56% were males. Most frequent prior antifungals were: posaconazole (32%), echinocandins (28%) and fluconazole (24%), mainly administered for primary prophylaxis (80%). Most common hematological malignancy was acute myeloid leukemia (52%), followed by non-Hodgkin lymphoma (11%) and myelodysplastic syndrome (10%). Sixty-two (50%) patients had received a hematopoietic stem-cell transplant (HSCT), mostly allogenic (54 of them). Figure 1 details the microbiological characteristics of the 39 (32%) proven cases. Candidemia was the most frequent proven BtIFI (59%) followed by mucormycosis (18%). Remarkably, 87% of Candida species were non-*albicans*. Resistance to prior antifungal was widespread. A probable BtIFI was diagnosed in 56 (45%) cases, mainly aspergillosis. Among those probable BtIFI, neutropenia (77%) and HSCT (54%), were the most common host factors, while a CT scan suggestive of fungal infection was the main clinical criteria (95% of cases). Microbiological criteria were met due to positive galactomannan in 42 (75%) patients, and a positive culture in 22 (39%). Additionally, there were also 29 (23%) possible BtIFI. Upon diagnosis, antifungal therapy was usually changed (113, 91%), most commonly to liposomal amphotericin-B (61, 49%), voriconazole (35, 28%), or echinocandins (30, 24%). Antifungal combination was initially used in 22 (18%) patients. Among the 46 episodes of BtIFI to posaconazole or voriconazole, therapeutic drug monitoring was only performed in 17 (37%) cases. After the first 100 days, 33 (27%) patients experienced progression of the fungal infection, and 100-day mortality was 51%, reaching to 54% when considering only probable and proven BtIFI.

**Conclusions:** BtIFI is commonly caused by rare fungi like Mucorales, Geotrichum or Fusarium and non-*albicans Candida* spp., although probable aspergillosis is still the most frequent IFI in this population. Most proven BtIFI were resistant to the prior antifungal administered. 100-day mortality was high.

## P246 Clinical Features and Surgery Outcome of Chronic Pulmonary Aspergillosis in Chinese Population

**Xianzhen Chen ^1^, Junqi Wu ^2^, Wenjie Fang ^1^, Yiliang Su ^2^, Qiankun Chen ^2^, Xiaodi Lu ^1^, Tianyang Chen ^1^, Wanqing Liao ^1^, Weihua Pan ^1^ and Chang Chen ^2^**

^1^ Shanghai Changzheng Hospital
^2^ Shanghai Pulmonary Hospital

**Objectives:** Chronic pulmonary aspergillosis (CPA), including simple aspergilloma, chronic cavitary pulmonary aspergillosis (CCPA) and chronic fibrosing pulmonary aspergillosis, affects over 3 million patients globally. CPA tends to infect immunocompetent or mildly immunosuppressed patients with or without preexisting lung disorders, such as chronic obstructive pulmonary disease or pulmonary tuberculosis. The 5-year mortality of CPA is approximately 40–50%, and long-term antifungals or surgery is needed for treatments. Few large-scale investigations of CPA based on Chinese population is reported so far. Hence this study aims to explore the epidemiology, clinical profiles and surgical outcomes of CPA in our clinical center.

**Materials & Methods:** This study was approved by the institutional review board of the Shanghai Pulmonary Hospital. The following data were collected for analysis: date of inpatient admission, gender, age, clinical manifestations, laboratory findings, information on misdiagnoses, antifungals, surgical treatment and outcome. SPSS (version 23.0, IBM Corp., Armonk, NY, USA) were used for the statistical analyses. Results were presented as mean±s.d. for normal data or median with interquartile ranges (IQRs) for non-normal data.

**Results:** From 2011 to 2017, there were 368 pathologically diagnosed CPA patients who underwent surgery and completed a follow-up at least two years. The mean age was 51.3 ± 13.0 years (95% CI: 49.9–52.6), and there was no significant difference in sexual distinction (male/female ratio = 0.947). Among patients with pulmonary disorders, the principal underlying lung disease was tuberculosis (31.5%), followed by bronchiectasis (33.9%), lung cancer (4.9%), pulmonary abscess (2.1%), COPD (1.1%), and silicosis (0.2%). CT scan shown that the location of infection involved the right upper lobe (33.2%), right middle lobe (3.0%), right lower lobe (11.4%), left upper lobe (19.8%), left lower lobe (16.0%) and multiple lobe (13.3%) Hemoptysis was the most common symptom (64.4%), followed by expectoration (52.2%), fever (29.1%), chest pain (6.2%), and weight loss (1.1%). Nearly 85.6% of the patients were misdiagnosed prior to surgery. For cases suspected as CPA, itraconazole was most frequently used for anti-*Aspergillus* treatment. The surgical treatment involved lobectomy (82.3%), wedge resection (1.4%), segmentectomy (4.6%) and pneumonectomy (5.4%), and others (6.2%). The overall mortality during follow-up is 0.8%.

**Conclusions:** We provided the largest cohort of CPA in Chinese patients, and further described tis clinical features and surgery outcome. We hold that antifungal treatment combined with timely surgical operation can achieve high therapeutical effective rate and low mortality. We appreciate Pfizer Medical for providing support.

## P247 Identifying Conserved Generic *Aspergillus* spp. Co-Expressed Gene Modules Associated with Germination Using Cross-Platform and Cross-Species Transcriptomics

**Tim Baltussen ^1^, Jordy Coolen ^1^, Paul Verweij ^1^, Jan Dijksterhuis ^2^ and Willem Melchers ^1^**

^1^ Radboudumc
^2^ Westerdijk Fungal Biodiversity Institute

**Objectives:** One of the important means of distribution within the genus *Aspergillus* are single-celled survival structures, called conidia, that are released into the air. Conidia of fungal species in different genera, including *Aspergillus*, are globally distributed, and, due to their small size, can enter the lungs of animals, including humans. Germination of inhaled conidia is crucial to establish an infection in the host. After a conidium breaks dormancy, the germination is characterized by two stages: isotropic growth and polarized growth. Isotropic swelling of the conidia is followed by polarized growth. In this phase, the growth is directed to a local region on the cell surface, which results in the formation of a germ tube. A more profound understanding of the transitions from dormant conidia via germ tube initiation to hyphal tip formation may be vital in the search for possible novel strategies to eradicate early infection. In this study, we performed a cross-platform, cross-species comparative analysis of germinating *A. fumigatus* and *A. niger* conidia using transcriptional data from two published studies.

**Materials & Methods:** The raw data were accessed through the NCBI Gene Expression Omnibus (GEO) accession number GSE36439 and the NCBI Sequence Read Archive (SRA) accession number PRJNA408076. The *A. fumigatus* data were obtained via Illumina RNA-Seq and the *A. niger* data via Affymetrix *A. niger* Genome Genechips. To compare the transcriptomic profiles of *A. fumigatus* and *A. niger*, pairs of genes were identified in two different genomes using the Reciprocal Best Hit (RBH) method. The gene pairs were used to integrate the RNA-Seq dataset with the microarray dataset. The integrated dataset was analyzed using a constructed consensus network. The network was built using the WGCNA R package. The integrated dataset was put into a multi-set format suitable for consensus analysis.

**Results:** The consensus co-expression network analysis identified four gene modules associated with stages of germination. These modules showed numerous shared biological processes between *A. niger* and *A. fumigatus* during conidial germination. The turquoise module contained 992 genes, and was the largest module detected by the consensusWGCNA. The module was mostly enriched with genes involved in metabolism (secondary metabolism, fatty acid and carbohydrate metabolism), but other FunCat categories were also enriched, such as transcription and cellular transport. The black module contained 308 genes that were mostly involved in protein synthesis, but categories associated with energy were also enriched. The darkgreen module contained 65 genes, and was the second smallest module. Enriched categories were conjunction of sulfate associated with metabolism and modification by ubiquitin-related proteins associated with protein fate. The blue module contained 770 genes, and was the second largest module. This module was highly enriched in genes involved in polarized growth, together with genes involved in cell cycle and DNA processing.

**Conclusions:** We demonstrated the possibility for comparative analysis between *Aspergillus* spp. using two different transcriptional profiling platforms, which introduces the opportunity to perform cost-effective insightful comparisons. Through cross-platform, cross-species comparative analysis, we were able to identify biologically meaningful modules shared by *A. fumigatus* and *A. niger*, which underscores the potential of this approach.

## P248 Development of the ECMM EQUAL Score Scedosporiosis/Lomentosporiosis to Measure Guideline Adherence and Clinical Management of Scedopsoriosis and Lomentosporiosis

**Jannik Stemler ^1,2,3^, Michaela Lackner ^4^, Sharon A. Chen ^5^, Martin Hoenigl ^6,7,8^ and Oliver A. Cornely ^1,2,3,9^**

^1^ Department I for Internal Medicine, University Hospital of Cologne
^2^ Chair Translational Research, Cologne Excellence Cluster on Cellular Stress Responses in Aging-Associated Diseases (CECAD), University of Cologne
^3^ German Centre for Infection Research (DZIF), Partner Site Bonn-Cologne
^4^ Institute of Hygiene and Medical Microbiology, Department of Hygiene, Medical Microbiology and Public Health, Medical University Innsbruck
^5^ Centre for Infectious Diseases and Microbiology Laboratory Services, Institute of Clinical Pathology and Medical Research, New South Wales Health Pathology, Westmead Hospital and the University of Sydney
^6^ Division of Infectious Diseases and Global Public Health, University of California San Diego
^7^ Clinical and Translational Fungal-Working Group, University of California San Diego
^8^ Section of Infectious Diseases and Tropical Medicine and Division of Pulmonology, Medical University of Graz
^9^ Clinical Trials Centre Cologne (ZKS Köln), Faculty of Medicine and University Hospital Cologne, University of Cologne

**Objectives:** To develop a scoring tool to facilitate and measure adherence to current guideline recommendations for diagnosis, treatment and follow-up of invasive scedosporiosis and lomentosporiosis.

**Materials & Methods:** Experts from ECMM excellence centers reviewed current guidelines for clinical management of invasive infections with *Scedosporium* spp. and/or *Lomentospora* spp.

Recommendations for diagnosis, treatment and follow-up were summarized, assembled and weighed according to their strength of recommendation and evidence level (strongly recommended = 3 points; moderately recommended = 2 points; marginally recommended = 1 point, recommended against = 0 points).

**Results:** A total of 170 recommendations were identified. A 24-item tool was developed as EQUAL score card as displayed in Table 1. Twelve items for diagnosis with 19 achievable points were assembled.

For treatment, three general recommendation items with a maximal score of 9 were developed while for specific antifungal treatment, the two pathogens were separated. For scedosporiosis three items with a maximum achievable score of 4 and for lomentosporiosis four items with a maximum achievable score of 6 were established, respectively. Follow-up comprises two items (four points max.). Key recommendations for clinical outcome were weighed accordingly.

**Conclusions:** We propose the ECMM EQUAL Score Scedosporiosis and lomentosporiosis to quantitate current guideline recommendations for patient management. It remains to be validated in real-life patient cohorts and correlated with patient outcome.

## P249 147 Invasive Trichosporonosis Cases from Two Registries: The French Society for Medical Mycology and the Trichosporon Registry within FungiScope®

**Stefanie Gräfe ^1,2,3^, Danila Seidel ^1,2,3^, Ulrike Binder ^4^, Luisa Duran Graeff ^1,3^, Jon Salmanton-García ^1,2,3^, Stefanie K. Graefe ^1,2,3^, Michaela Lackner ^4^, Philipp Koehler ^1,2,3^, Alessandro Pasqualotto ^5^, Hilmar Wisplinghoff ^6^, Paschalis Vergidis ^7^, Jean-Pierre Gangneux^8^, Christophe Hennequin ^9^ and Oliver A. Cornely ^1,2,3,10^**

^1^ Excellence Center for Medical Mycology (ECMM), Department I of Internal Medicine, Faculty of Medicine and University Hospital Cologne, University of Cologne
^2^ Faculty of Medicine and University Hospital Cologne, Cologne Excellence Cluster on Cellular Stress Responses in Aging-Associated Diseases (CECAD), University of Cologne
^3^ Partner Site Bonn-Cologne, German Centre for Infection Research (DZIF)
^4^ Institute of Hygiene and Medical Microbiology, Medical University of Innsbruck
^5^ Infection Control Department, Santa Casa Complexo Hospitalar, Universidade Federal do Rio Grande do Sul
^6^ Institute for Medical Microbiology, Immunology and Hygiene, University of Cologne
^7^ Division of Infectious Diseases, Mayo Clinic
^8^ Infection, Immunité, Facteurs Environnementaux et foie [2IFEF], Université Rennes 1
^9^ Service de Parasitologie-Mycologie—Hopital St. Antoine CIMI, Université Pierre et Marie Curie-Inserm
^10^ Clinical Trials Centre Cologne (ZKS Köln), Faculty of Medicine and University Hospital Cologne, University of Cologne

**Objectives:** *Trichosporon* is the second most common cause of opportunistic invasive yeast infection after *Candida* species. Trichosporonosis is associated with poor outcome and high mortality rate. Amphotericin B shows limited efficacy against *Trichosporon* and species are intrinsically resistant to echinocandins. Even though triazoles are the preferred antifungal agents, studies evaluating improved outcome are scarce. Here, clinical data of *Trichosporon* infections and antifungal susceptibility patterns of clinical isolates were collected for comprehensive analysis.

**Materials & Methods:** Clinical data were collected in the FungiScope® registry. Collaborators retrospectively provided anonymized clinical data. Additionally, cases identified through the national lead investigator of the French Society of Medical Mycology (SFMM) were included. All cases were reviewed by FungiScope® infectious diseases experts. Clinical isolates were collected for standardized pathogen identification and susceptibility testing.

**Results:** In total, 150 cases of invasive trichosporonosis were documented from 22 countries with 59 included through SFMM. Most of the isolated species were *T. asahii* (n = 98, 65%), followed by *T. inkin* (n = 14, 9%). Main predisposing factors observed were malignancy (n = 81, 54%), chemotherapy (n = 66, 44%), and neutropenia (n = 59, 40%). Other predisposing factors included treatment with immunosuppressants (n = 23, 15%), stem cell transplantation (n = 20, 13%), solid organ transplantation (n = 16, 11%), and abdominal surgery (n = 15, 10%). A third of patients (n = 49, 32%) were treated in intensive care units prior to first signs of infection. The majority of patients presented with disseminated disease (n = 121, 81%), mostly confirmed by blood culture (n = 115, 77%). Breakthrough invasive fungal infection was reported in 42 cases (28%). Overall, amphotericin B was the most frequently used antifungal agent (n = 73, 48%), followed by voriconazole (n = 63, 42%). Voriconazole and/or posaconazole-based treatment was associated with improved survival compared to amphotericin B alone or no antifungal treatment. All-cause mortality rate was 54%, with 90% of deaths attributable to fungal infection.

**Conclusions:** Invasive trichosporonosis is most commonly caused by *T. asahii* with malignancy presenting as the most frequent predisposing factor. Voriconazole and/or posaconazole-based treatment is associated with improved outcome compared to amphotericin B alone.

## P250 *Olecranon bursitis* caused by *Scedosporium apiospermum* in a Patient Treated with CAR-T Cells

**Willem Falkenburg ^1^, Marit Jalink ^2^, Marie José Kersten ^2^, Jochem Buil ^3^ and Karin van Dijk^1^**

^1^ Department of Medical Microbiology and Infection Control, Amsterdam University Medical Centers
^2^ Department of Haematology, Amsterdam University Medical Centers
^3^ Department of Medical Microbiology, Radboud University Medical Center, and Radboudumc—CWZ Center of Expertise for Mycology

**Background:** *Scedosporium* species can be found everywhere in the environment, including in soil and in polluted water. They cause infections mainly in immunocompromised hosts and are difficult to treat. We present an immunocompromised patient with bursitis of the olecranon caused by *Scedosporium apiospermum*.

**Case presentation:** A 44-year old patient presented with a bursitis of his left elbow. Six months before he was treated with lymphodepleting chemotherapy directly followed by B-cell associated CD19-specific chimeric antigen receptor (CAR) T cell therapy for recurrence of follicular lymphoma. He was considered immunocompromised, with total neutrophils and lymphocytes slightly below normal levels, absent B-cells, and a treatment associated persistent hypogammaglobulinaemia. Four weeks before presentation at our hospital he contacted his GP because of swelling and pain of his left elbow. His GP aspirated fluid from his elbow, injected steroids and started antibiotics, but now there was recurrence of swelling and pain. Bursitis without arthritis was diagnosed by a rheumatologist and the bursa was surgically opened and drained, relieving pus. The patient was started on oral flucloxacillin and sent home. Two days later the culture of the pus from the bursa grew white colonies on a Sabouraud dextrose agar plate, showing long conidophores with large oval conidia on microscopy. *Scedoporium* infection was suspected, and the patient was switched to voriconazole therapy (200 mg, twice daily, orally). A PET-CT scan did not show signs of systemic involvement and blood cultures remained negative. Because of vivid hallucinations and sleeplessness four days after starting voriconazole the patient was switched to posaconazole 300 mg a day and drug serum concentrations were monitored. Over the next few weeks the bursitis symptoms diminished. The isolate was identified as *Scedosporium apiospermum* using beta-tubulin sequence analysis and MICs were determined using the EUCAST reference method (voriconazole 0.5 mg/L, posaconazole 1.0 mg/L). Voriconazole is considered first line treatment for *Scedosporium* infections. However, posaconazole was continued because of the side effects experienced by the patient on voriconazole and the lack of alternative treatment options for *Scedosporium* infections. Two months after his initial presentation, swelling of the patients’ elbow returned and an ultrasound again showed a significant bursitis. Fluid was aspirated and cultures again showed *Scedosporium apiospermum* even though the patient was still using posaconazole with adequate drug concentrations. MICs however were increasing (posaconazole 2.0 mg/L, voriconazole 1.0 mg/L) and it was feared that the infection would spread systemically and therefore the bursa was surgically extirpated. Eight weeks later there were no signs of bursitis and posaconazole was discontinued.

**Discussion and Conclusions:** This case highlights that patients treated with CAR-T cells can be immunocompromised for months or longer after treatment. Physicians should be alert for opportunistic infections, such as infections with *Scedosporium* species. These infections can be difficult to treat, may require surgery and prolonged antifungal therapy, with limited antifungal options.

**Declarations:** Written informed consent was obtained from the patient.

## P251 Invasive Infections due to Saprochaete Species in Hematological Malignancies Patients. A Multicenter Study on Behalf of Seifem/Fungiscope Registry

**Maria Ilaria Del Principe ^1^, Marianna Criscuolo ^2^, Danila Seidel ^3,24^, Michelina Dargenio ^4^, Zdeněk Ráčil ^5^, Monica Piedimonte ^6^, Francesco Marchesi ^7^, Gianpaolo Nadali ^8^, Philipp Koehler ^3,24^, Nicola Fracchiolla ^9^, Chiara Cattaneo ^10^, Nicolay Klimko ^11^, Angelica Spolzino ^12^, Deniz Yilmaz Karapinar ^13^, Hayati Demiraslan ^14^, Rafael Duarte ^15^, Judit Demeter ^16^, Marta Stanziani ^17^, Lorella Melillo ^18^, Claudia Maria Basilico ^19^, Simone Cesaro ^20^, Giovangiacinto Paterno ^1^, Catello Califano ^21^, Mario Delia ^22^, Alessandro Busca ^23^, Oliver A Cornely ^3,24,25^ and Livio Pagano ^2^**

^1^ Department of Biomedicine and Prevention, Tor Vergata University
^2^ Dipartimento di Diagnostica per immagini, radioterapia oncologica ed ematologia Fondazione policlinico universitario A. Gemelli IRCCS Roma
^3^ University of Cologne, Faculty of Medicine and University Hospital Cologne, Department I of Internal Medicine, Excellence Center for Medical Mycology (ECMM)
^4^ Ematologia e Trapianto di Cellule Staminali, Ospedale Vito Fazzi
^5^ Internal Haematology and Oncology Clinic, University Hospital Brno, Czech Republic, Institute of Hematology and Blood Transfusion
^6^ Dipartimento di Medicina Clinica e Molecolare, Azienda Ospedaliera Universitaria Sant’Andrea di Roma Università Sapienza di Roma
^7^ Hematology and Stem Cell Transplant Unit, IRCCS Regina Elena National Cancer Institute
^8^ Unità Operativa Complessa di Ematologia, Azienda Ospedaliera Universitaria Integrata di Verona
^9^ UOC di Ematologia, Fondazione IRCCS Ca’ Granda, Ospedale Maggiore Policlinico
^10^ Divisione di Ematologia, ASST-Spedali Civili di Brescia
^11^ Department of Clinical Mycology, Allergy and Immunology, North Western State Medical University
^12^ Unità di Ematologia e Trapianto di Midollo Osseo, Dipartimento di Medicina e Chirurgia, Università di Parma
^13^ Departments of Pediatrics Medical Genetics, Ege University Faculty of Medicine
^14^ Department of Infectious Diseases, Faculty of Medicine, Erciyes University
^15^ Department of Hematology, Hospital Universitario Puerta de Hierro Majadahonda
^16^ Semmelweis University Department of Internal Medicine and Oncology, Division of Hematology
^17^ Istituto di Ematologia ed Oncologia Medica “L. e A. Seragnoli”, Ospedale Sant’Orsola Malpighi
^18^ Divisione di Ematologia; IRCCS Casa Sollievo della Sofferenza
^19^ Dipartimento delle Medicine Specialistiche, ASST Sette Laghi Struttura Complessa di Ematologia, Ospedale di Circolo
^20^ Pediatric Hematology Oncology, Department of Mother and Child; Azienda Ospedaliera Universitaria Integrata di Verona
^21^ U.O.C EmatologiaP.O. A. Tortora.Pagani
^22^ Sezione di Ematologia, Dipartimento dell’Emergenza e dei Trapianti d’Organo-Università di Bari
^23^ Stem Cell Transplant Center, AOU Citta’ della Salute e della Scienza
^24^ University of Cologne, Faculty of Medicine and University Hospital Cologne, Chair Translational Research, Cologne Excellence Cluster on Cellular Stress Responses in Aging-Associated Diseases (CECAD)
^25^ University of Cologne, Faculty of Medicine and University Hospital Cologne, Clinical Trials Centre Cologne (ZKS Köln)

**Objectives:** The epidemiology of invasive fungal infections in patients affected by hematological malignancies (HM) has varied, due to the antifungal treatment strategies, the increased use of antifungal prophylaxis (AP), the changes of hosts features and the HM treatments. Though *Candida* and *Aspergillus species* (spp.) keep playing the major role, rare yeasts as *Saprochaete (S.)* species have been increasingly reported as agents of fungaemia and severe infections. Owing the difficulty of isolation and the intrinsic resistance to echinocandins, these infections are associated with high mortality rates. **Materials & Methods:** To identify baseline factors and provide a basis for therapeutic decisions, we conducted a retrospective multicenter study. All cases of proven *S. capitata* and *S. clavata* infection, observed from January 2010 to December 2020 in HM patients, were collected from SEIFEM (Sorveglianza Epidemiologica Infezioni nelle Emopatie) group and from FUNGISCOPE (Global Emerging Fungal Infection Registry) database. The characteristics of our patients were compared with those of a group of HM patients with *Candida (C)*species infection, matched for age and treatment. **Results**. We recorded 90 *S.* species cases, median age 54 years (range 2–78), 46 patients (50%) were female and 69 (74%) had a diagnosis of acute leukemia. Of these, 64 (71%) were classified as *S. capitata* infections and 26 (29%) as *S. clavata*. In univariate analysis, the infection of *S. clavata* was associated with age <60 years (21/26 patients, *p* = 0.01). Overall, 82% cases presented fungemia. Focal or disseminated organ involvement was observed in 40 (44%) of cases. Antifungal prophylaxis (AP) and the central venous catheter (CVC) correlated with *S.* species (*p* = 0.000) and *C.* spp. infections (*p* = 0.004), respectively. Thirty-six (40%) *S. * species cases were breakthrough infections as occurring during AP, mainly anti-mold prophylaxis. Two patients didn’t receive antifungal therapy (AT). The AT was liposomal amphotericin B (L-AMB) in 38 (43%), azoles in 26 (30%), echinocandins in 24 (27%) and combination (azoles plus L-AMB) in 7 (8%) patients. The efficacy of first AT was observed in 8/38 (21%), 16/26 (61%) and 1/24(4%) of patients who received L-AMB, azoles and echinocandins, respectively (*p* = ns). CVC was removed after fungal isolation in 42/77 (54%). Mortality rate at 30 days was 46%. Factors that influenced outcome were the age >60 years (*p* = 0.024), the septic shock (*p* = 0.012), the duration of steroid treatment (*p* = 0.035) and the neutrophil recovery (*p* = 0.000). In multivariate analysis, the only parameter influencing the outcome was the neutrophil recovery (OR: 8.54, 95%CI 2.07–35,240, *p* = 0.003). **Conclusions**. The *S.* species infections are often breakthrough infections, the most effective treatment for which has not yet been established, but neutrophil recovery appears to play an important role in the favorable outcome.

## P252 *Subcutaneous phaeohyphomycosis* due to *Phaeoacremonium parasiticum* in a Patient with Chronic Granulomatous Disease

**Dinah Carvalho ^1^, Raquel Sabino ^2,6^, Cristina Veríssimo^2^, Helena Simões ^2^, Susana Lopes Silva ^3^, Tiago Marques ^4^, João Janeiro ^5^, Luis Marques Lito ^1^ and José Melo Cristino^1^**

^1^ Centro Hospitalar Universitário Lisboa Norte—Clinical Pathology Department
^2^ National Health Institute Dr. Ricardo Jorge, Reference Unit for Parasitic and Fungal Infections, Department of Infectious Diseases
^3^ Centro Hospitalar Universitário Lisboa Norte—Immunoallergology Department
^4^ Centro Hospitalar Universitário Lisboa Norte—Infectious Diseases Department
^5^ Centro Hospitalar Universitário Lisboa Norte—Imaging Department
^6^ Instituto de Saúde Ambiental, Faculdade de Medicina, Universidade de Lisboa

**Objectives:** *Phaeoacremonium parasiticum* is a ubiquitous dematiaceous mold that rarely causes infection in humans. Its spectrum of disease ranges from subcutaneous infections to disseminated disease. The majority of those reported few cases involve immunocompromised patients. Chronic granulomatous disease (CGD) is an inherited disorder affecting nicotinamide adenine dinucleotide phosphate (NADPH) oxidase complex. CGD patients are susceptible to a broad spectrum of opportunistic infections, being fungal infections a major determinant of survival. We report a case of *Subcutaneous phaeohyphomycosis* due to *P. parasiticum* in a young adult with CGD.

**Material and methods:** A 28-year-old male patient with autossomic recessive CGD, due to mutations in CYBA, is followed in Primary Immunodeficiency Center since childhood. He has been under prophylaxis with itraconazole, cotrimoxazole and IFNg and tapering oral steroids, started for granulomatous colitits 2 years earlier.

In a regular visit to the clinical center, he complained of persistent pain on his left leg with no history of recent injury in the affected area and without external inflammatory signs. An ultrasound was performed after 2 weeks revealing a heterogeneous liquid of slightly irregular contour in the sinus of the left anterior tibial muscle with heterogeneity of the adjacent muscle. This piomiositis collection was punctured, under ultrasound control, with drainage of about 7cm^3^ of hemato-purulent exsudate that was promptly processed for bacteriology (aerobic and anaerobic) and mycology studies.

**Results:** Cultures for bacteria were sterile. Culture on Sabouraud was positive after 5 days of incubation, showing slow growing and initially white colonies. Microscopic examination showed hyaline, septate mycelium with long, thin conidiophores and curved, aseptate conidia.The fungus was initially identified as an *Acremonium* sp. and was sent to the Mycology Reference Laboratory for molecular identification and antifungal susceptibility testing. Its identification was performed by sequencing the internal transcribed spacer (ITS) region of ribosomal DNA, being the isolate identified as *Phaeoacremonium parasiticum* (100% homology).

After 3 weeks incubation, coloration of the colonies emerged, becoming greyish black upon subculture, with velvety texture and black reverse. Microscopically, pigmented hyphae with tapering, funnel-shaped phialides were observed, and conidia were hyaline and oblong, forming clusters at the tip of the phialides. At this point, macroscopic and microscopic morphology was consistent with *Phaeoacremonium* species.

Susceptibility pattern showed low minimal inhibitory concentrations (MIC) to posaconazole and voriconazole, and higher MIC values to anidulafungin and amphotericin B. The patient has improved under voriconazol therapy (200 mg; bid)

**Conclusions:** *Phaeoacremonium parasiticum* is an uncommon infection and its appropriate identification is often difficult because morphologically, the genus *Phaeoacremonium* show morphological features resembling both *Acremonium* and *Phialophora* genera.

Molecular identification is determinant to confirm morphology, as many species have indistinguishable characteristics that may lead to incorrect antifungal options. Also, susceptibility testing should be done for these so rare fungi as optimal treatment has not yet been clearly defined.

## P253 Ascertaining the True Incidence of Histoplasmosis in Nigeria Advanced HIV Disease Population

**Rita Oladele ^1^, Iriagbonse Osaigbovo ^2^, Bassey Ekeng ^4^, Mark Okolo ^7^, Mohammed Yahaya ^5^, Olukemi Adekanmbi ^3^, Mary Alex-Wele ^6^, Toyose Ayanbeku ^8^, Uche Unigwe ^9^, Alessandro Pasqualotto ^10^ and Tom Chiller ^11^**

^1^ Department of Medical Microbiology and Parasitology, College of Medicine, University of Lagos
^2^ Department of Medical Microbiology and Parasitology, University of Benin Teaching Hospital
^3^ Department of Medicine, University of Ibadan
^4^ Department of Medical Microbiology and Parasitology, University of Calabar Teaching Hospital
^5^ Department of Medical Microbiology and Parasitology, University of Sokoto Teaching Hospital
^6^ Department of Medical Microbiology and Parasitology, University of Port Harcourt Teaching Hospital
^7^ Department of Medical Microbiology and Parasitology, University of Jos Teaching Hospital
^8^ Department of Medical Microbiology, Federal Medical Centre, Bida
^9^ Department of Medicine, University of Nigeria Teaching Hospital
^10^ Universidade Federal de Ciencias da Saude de Porto Alegre
^11^ Mycotic division, Centre for Disease Control (CDC)

**Objectives:** Histoplasmosis is a neglected fungal disease associated with high mortality in untreated people sufferers of advanced HIV disease. Nigeria is the seventh highest burden country globally for tuberculosis, and histoplasmosis, an AIDS-defining infection, is commonly misdiagnosed as tuberculosis. One of the major problems with Histoplasmosis and TB in HIV is the similarity in signs and symptoms. Nigeria also has the highest number (124 out of 470 cases) of documented histoplasmosis cases in Africa; however, almost all were found before the HIV pandemic. The primary objective of this study was to determine the incidence of histoplasmosis amongst advanced HIV disease (AHD) patients in Nigeria.

**Materials and Methods:** We conducted a prospective multi-center cohort study, in ten study sites, across six geopolitical zones in Nigeria. AHD patients (were either antiretroviral treatment (ART) naïve and exposed) with at least 2 of 6 clinical features (Fever; chronic cough, weight loss; cutaneous lesions; oral ulcers; typical radiological findings), were recruited. Two urinary samples collected one week apart were collected for *Histoplasma* urinary antigen testing (Clarus *Histoplasma* GM, product reference HGM201. IMMY). *Histoplasma* antigen skin sensitivity testing was performed on close contacts and relatives of all cases. Data were analyzed with SPSS 24.0.

**Results:** 1100 patients were recruited. 932 patients’ urine samples were tested. Mean patient age was 40 years, SD 11.17. Females accounted for 62.4% with a male: female ratio of 1:1.6. Median CD4 count was 128 cells/mm^3^ (interquartile range 70–182). One-third (34%) of studied participants had up to secondary school education level, and a significant proportion (41%) of occupation recorded was private business. One hundred and seventeen (16%) were tuberculosis GeneXpert positive. Sixty-four (7%) patients were positive for *Histoplasma* urinary antigen. There was wide geographical variation (1.3–20%) of incidence across the study sites. Six (10%) of the *Histoplasma* urinary antigen positive patients were also positive by GeneXpert, with 16 (25%) receiving anti-tuberculosis therapy. CD4 counts of *Histoplasma* antigen positive patients were significantly lower than those negative (*p*-value, 0.033), and majority (36; 56%) of the *Histoplasma* urinary antigen positives were hospitalized patients. ART was also significant (*p*-value: 0.015) with 50 (78%) *Histoplasma* urinary antigen positives, being ART experienced.

**Conclusions:** Rather than a rare disease, histoplasmosis is hiding in plain sight amongst AHD patients in Nigeria. Observed histoplasmosis and TB confections indicate that misdiagnosis could occur, as both infections may cause similar clinical symptoms. Urgent implementation of histoplasmosis diagnostics into the AHD package of care in Nigeria will save lives.

## P254 Pulmonary Aspergillosis after Lung Transplantation: Incidence of Colonisation, Susceptibility, and Mortality Rates during 10 Years of Follow-Up

**Gerhard Fleurke, G. A. Kampinga, N. Hilt, C. C. van Leer-Buter, E. A. Verschuuren and D. F. Postma**

University Medical Center Groningen

**Objectives:** Aspergillus infections are associated with an increase of mortality in lung transplant patients. Adequate therapy and/or prophylaxis is of great importance and should be guided by knowledge of local species distribution and susceptibility. We are adding mycological culture results to our follow-up database of lung transplant recipients. Our goal is to study the epidemiology and risk factors for Aspergillus colonization or invasive pulmonary aspergillosis and describe our anti-fungal stewardship results. For now, we describe the incidence, species determination, and azole resistance rates of pulmonary colonization with Aspergillus in lung transplant recipients over a period of 10 years.

**Materials & Methods:** Observational cohort study which includes all lung transplant patients transplanted and/or in follow up during the study time window (from 01-01-2011 to 01-05-2021). Reported outcomes will be incidence of Aspergillus colonization, Aspergillus species determination, azole susceptibility, and overall patiënt survival. Positive cultures for Aspergillus within 3 months were considered a single episode of Aspergillus colonization.

**Results:** The total number of lung transplant recipients in follow up was 565 patients comprising 3130 patient follow-up years. The mean age at first lung transplantation was 48 years (median: 53, IQR: 59–40, range: 6–69) and 52% was female. 205 patients (36%) had a positive culture for Aspergillus during 355 episodes over follow-up. The median duration of colonization was 3.0 months (IQR 3.1–3.0, range 3.0–9.8). The median number of episodes per patient was 1.0 (IQR 2.0–1.0, range 1–12).

*Aspergillis fumigatus* is the most commonly cultured species (80%) with an azole susceptibility of 79% (Table 1). Crude all-cause mortality rates were not different for patients with positive cultures for Aspergillus (Figure 1).

Table 1. *Aspergillis fumigatus* is the most commonly cultured species (80%) with an azole susceptibility of 79%.


**Aspergillus Species**

**Azole Susceptibility**


**Sensitive**

**Reduced**

**Sensitivity**


**n**
%
**n**
%
**Total**

*fumigatus*
273797121344
*niger*
187562524
*flavus*
18951519
*versicolor*
5633388
*nidulans*
5100005
*terreus*
5100005
*ochraceus*
0021002
*sydowii*
2100002
*ustus*
0021002
*glaucus*
0011001
*sclerotorium*
0011001species not determined17941618


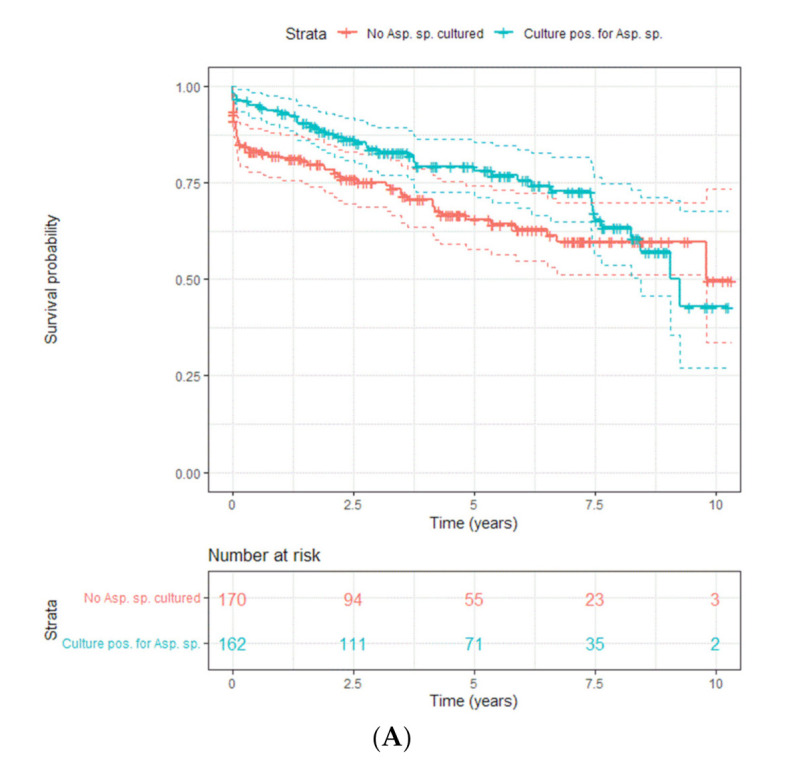
 
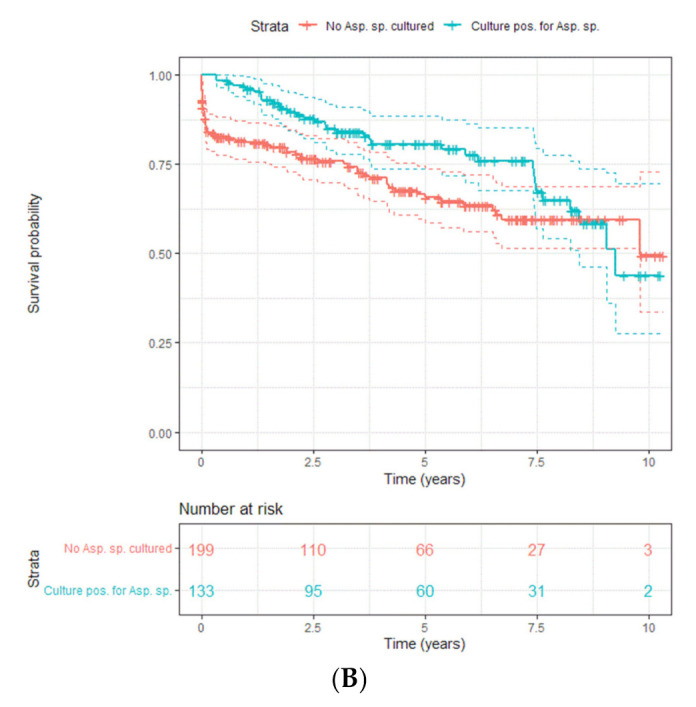


**Figure 1.** (**A**). Kaplan-Meier curve of patients who received a first lung transplant within the study window, stratified for any culture positive for *Aspergillus* vs. no positive cultures (with 95% CI). (**B**). Kaplan-Meier curve of patients who received a first lung transplant within the study window, stratified for culture positive for *Aspergillus* post transplantation vs. no positive cultures post transplantation (with 95% CI).

**Conclusions:** Colonization with *Aspergillus* is quite common in our large cohort of lung transplant recipients. Although resistance rates are increasing, a positive culture for *Aspergillus* is not crudely associated with all-cause mortality.

## P255 Risk Factors and Outcome of *Candida glabrata* Candidemia in 2 Tertiary University Hospitals: An 8-Year Retrospective Study (2012–2020)

**Emma VERSTRAETE ^1^, Alexandre ALANIO ^1^, Matthieu RESCHE-RIGON ^1^, Lou MACAUX ^1^, Anne-Lise MUNIER ^2^, Stéphane BRETAGNE ^1^, Jean-Michel MOLINA ^1,2^ and Blandine DENIS ^1^**

^1^ Saint-louis Hospital
^2^ Lariboisière Hospital

**Objectives:** Even in the last decade, one-month survival rate of candidemia remains below 70%. Furthermore, treatment of candidemia due to *Candida glabrata* is challenging given decreased fluconazole susceptibility and its rapid acquisition of resistance, especially to azoles and echinocandins. In order to better apprehend risks factors associated with *C. glabrata* candidemia, we compared episodes of *C. glabrata* candidemia to *C. albicans* candidemia in a retrospective observational study conducted in two French tertiary hospitals, during the 2012–2020 period. Outcome with global and four-weeks survival rates were also analyzed.

**Materials & Methods:** Data on demographic characteristics, microbiological data, risk factors, clinical manifestations, therapeutic management and outcome associated with *C. glabrata* and *C. albicans* candidemia were retrieved from medical records and from the mycology laboratory.

**Results:** During the study period, 176 episodes of candidemia were analysed, with 51 (29%) *C. glabrata* candidemia and 125 (71%) *C. albicans* candidemia. Globally, 52% of candidemia occurred in medicine wards, 37% in intensive care and 11% in surgery departments, 57% were male patients with a median age of 60 years old [IQR: 48;68]. Hemato-oncology patients were predominant (141/176, 80%), with 89 (51%) patients having hemopathy and 31 (18%) with diabetes. Foreign materials were noted for 168 (95%) episodes, previous candida colonization in 51 (29%) episodes, an antifungal prophylaxis and curative treatment within the past month in respectively 13 (7.5%) and 32 (18%) of episodes. Overall survival was 72% (95%CI: 66–79%) at four-weeks and 35% (95%CI: 28%–43%) at one-year. Compared to *C. albicans*, risk factors significantly associated to *C. glabrata* candidemia were later onset of candidemia during hospitalisation (16 [IQR: 4.5;31] vs. 10 [IQR: 2;18] days; *p* = 0.02), antifungal prophylaxis in the past 30 days (16% vs. 4%, *p* = 0.010), previous allogeneic hematopoietic stem cell transplantation (21.6% vs. 3.2%, *p* = 0.0003), type 2 diabetes mellitus (25.5% vs. 12%, *p* = 0.04), previous history of candidemia (11.8% vs. 3.2%, *p* = 0.04) and type of hemopathy (*p* = 0.04), with more acute leukemia (11/26, 42%) for *C. glabrata* candidemia while lymphoma (38/66, 58%) were predominant for *C. albicans* candidemia. Among allograft patients, graft-versus-host disease was more frequent in *C. glabrata* fungemia, although not significant (63.6% vs. 25%, *p* = 0.28). During antifungal treatment, there were less therapy switches for *C. glabrata* candidemia [15 (35%) vs. 54 (56%), *p* = 0.03]. There was a non-significant trend towards improved survival with *C. glabrata* (HR = 0.75 [0.51;1.11], *p* = 0.15), 4-weeks survival were respectively 82% vs. 68% for *C. glabrata* and *C. albicans* respectively.

**Conclusions:** In comparison to *C. albicans* candidemia, *C. glabrata* candidemia was more likely to occur after a prolonged hospital stay, in patients with recent antifungal therapy, type 2 diabetes mellitus, allogeneic stem cell transplantation, acute leukemia or a history of *C. glabrata* candidemia. Allogeneic stem cell transplantation and acute leukemia are probably more represented given the indication of antifungal prophylaxis in these settings. Previous published factors such as age above 65, previous abdominal surgery or abdominal source of candidemia, broad spectrum antibiotherapy were not significant in our study, probably due to hemato-oncologic specificities of our hospitals.

## P256 Potentiating Effect of Drug Transporters Inhibition on Posaconazole Activity against Mucorales

**Marion Aruanno ^1,2^, Daniel Bachmann ^1^, Eric Durandau ^1^, Dominique Sanglars ^1^ and Frédéric Lamoth ^1,2^**

^1^ Institute of Microbiology, Lausanne University Hospital
^2^ Infectious Diseases Service, Department of Medicine, Lausanne University Hospital

**Objectives:** Molds of the order *Mucorales*, such as *Rhizopus* spp. and *Mucor* spp., are the causal agents of mucormycosis, a life-threatening infection in immunocompromised and diabetic patients. Mucormycosis is notoriously difficult to treat because of the limited number of therapeutic options. The azole drug posaconazole is effective against *Mucorales*, but variable in vitro antifungal activity is observed. Mechanisms of azole resistance in *Mucorales* remain largely unknown, in particular little is known about the role of efflux pumps (ABC and MFS drug transporters). The objective of this work was to assess the ability of the drug transporter inhibitor Phenylalanine-Arginine Beta-Naphthylamide (PaβN) to inhibit these transporters and to potentiate posaconazole activity in *Mucor circinelloides* and *Rhizopus microsporus*.

**Methods:** The inhibitory effect of PaβN on transporters activity was assessed by measuring the efflux of their fluorescent substrate rhodamine 6G following glucose injection. The interaction of PaβN and posaconazole was assessed by the chequerboard dilution method in order to determine the fractional inhibitory concentration index (FICI).

**Results:** The transporter inhibitor PaβN was able to decrease the efflux of rhodamine 6G in both *M. circinelloides* and *R. microsporus* (Figure 1), which suggests its ability to efficiently inhibit drug transporters in *Mucorales*. PaβN could potentiate posaconazole activity against five *M. circinelloides* and *R. microsporus* ATCC or clinical isolates with FICI fulfilling the criteria of synergism (0.5) or close to these criteria (0.5 to 0.6).

**Conclusions:** Activation of efflux pumps (drug transporters) may represent a relevant mechanism of azole resistance in *Mucorales*. Indeed, PaβN could efficiently inhibit these transporters and potentiate the activity of posaconazole against *M. circinelloides* and *R. microsporus*. Drug transporters may represent a novel therapeutic target for the treatment of mucormycosis.

## P257 Complement Evasion of *Candida albicans* Is Glucose-Dependent: The Role of the Factor H Binding Molecule Hgt1

**Verena Harpf ^1^, Samyr Kenno ^1^, Cornelia Speth ^1^, Günter Rambach ^1^, Verena Fleischer ^1^, Nadia Parth ^1^, Christian Weichenberger ^2^, Peter Garred ^3^, Silke Huber ^1^, Cornelia Lass-Flörl ^1^ and Reinhard Würzner ^1^**

^1^ Institute of Hygiene and Medical Microbiology, Medical University of Innsbruck
^2^ Center for Biomedicine, EURAC
^3^ Department of Clinical Medicine, Rigshospitalet—Diagnostisk Center

**Background:** *Candida albicans* is a commensal in humans, but can cause opportunistic infections. It has developed several complement evasion mechanisms like hijacking factor H (FH), the major regulator of the alternative pathway, using FH-binding molecules. The expression of high-affinity glucose transporter 1 (Hgt1), a complement evasion molecule, is glucose-dependent. Diabetes represents a risk factor for developing candidiasis.

**Objectives:** (i) Investigate the impact of diabetes and the complement system on the virulence of a *Candida albicans* strain lacking Hgt1 and the parental strain in a murine model of disseminated candidiasis; (ii) study relevance of glucose for phagocytosis of selected *Candida albicans* strains by granulocytes in vitro; (iii) check in vitro C3b and FH deposition on these selected strains at defined glucose concentrations.

**Materials & Methods:** Immunocompetent, diabetic, and C3-deficient (DC3) mice were intravenously infected with either *Candida albicans* lacking Hgt1 (hgt1−/−) or the parental strain (SN152). Survival and clinical status were monitored over 14 days.

For in vitro experiments *Candida albicans* wild-type (SC5314), SN152, and hgt1−/− strains were grown in 0.1, 0.2, 0.3, and 2% glucose. Subsequently, the yeast cells were opsonized with serum as a source of complement. Phagocytosis was studied by FITC-labelling opsonized yeast cells, incubating these cells with granulocytes, and analyzed using FACS. C3b/iC3b and FH deposition on *Candida albicans* cells were measured by FACS using specific antibodies.

**Results:** The murine model showed hgt1−/− to be more virulent than SN152. In diabetic mice, no significant difference in virulence between the two strains was observed. SN152 displayed significantly higher virulence in diabetic, and particularly in DC3 mice, compared to immunocompetent, which shows the relevance of diabetes and complement in candidiasis.

In vitro, a tendency for lower phagocytosis of SC5314 and SN152 with higher glucose was revealed. No difference was observed at physiological glucose concentrations with hgt1−/−, but significantly less phagocytosis was shown at 2% glucose. Findings indicate lower FH deposition and higher C3b/iC3b deposition on SC5314 and SN152 incubated at higher glucose. While there was no difference in FH and C3b/iC3b deposition on hgt1−/− at physiological glucose concentrations, FH deposition was significantly increased at 2% glucose.

**Conclusions:** Complement can be linked to higher virulence of *Candida albicans*. Although C3b/iC3b and FH deposition are glucose-dependent, this has a minor influence on phagocytosis. The absence of Hgt1 diminishes glucose dependency of C3b/iC3b and FH deposition at physiological glucose concentrations, but this absence cannot be attributed to survival in a murine model. The rise of FH deposition at 2% glucose in hgt1−/− could be due to an upregulation of other FH-binding proteins.

## P258 Clinical Profiles and Culture Results of *Aspergillus* spp. Isolated from Non-Small Cell Lung Carcinoma (NSCLC) Patients

**Jamal Zaini, Abul A’l Al Maududi, Zahra Annisa, Findra Setianingrum, Robiatul Adwiyah, Mulyati Tugiran, Ridhawati Sjam and Anna Rozaliyani**

Faculty Of Medicine, Universitas Indonesia

**Objectives:** Lung cancer patients are at risk of invasive aspergillosis (IA). Most of them develop.

IA related with immunosuppression caused by chemotherapy. Few clinical and laboratory data are available on non-small cell lung carcinoma (NSCLC) patients before chemotherapy. This study aims to determine the correlation between clinical profiles and *Aspergillus* culture in patients with non-small cell lung carcinoma (NSCLC) before chemotherapy.

**Material andMethods:** In a cross-sectional study, 70 NSCLC patients from a national referral hospital in Jakarta, Indonesia were recruited from July 2019–June 2020. Fungal cultures from induced sputum were performed on Sabouraud Dextrose Agar (SDA) medium using the high volume culture method. Species identification was conducted with direct microscopic examination. Clinical characteristics of patients were obtained from medical records.

**Results:** Sixty one percent were men and the mean age was 60 years. Fifty-two (74.3%) patients had adenocarcinoma and 18 (25.7%) patients had squamous cell carcinoma. Most (n = 55, 78.6%) of patients were in advanced stage of cancer. The most common presenting symptoms were shortness of breath (n = 57, 81.4%), chest pain (n = 50, 71.4%), weight-loss (n = 50, 71.4%), cough (n = 32, 45.7%), haemoptysis (n = 20, 28.6%), and fever (n = 6, 8.6%). Underlying conditions were diabetes mellitus (n = 13, 18.6%) and tuberculosis (n = 9, 12.9%). Sixty seven isolates of *Aspergillus* spp. were identified from 43 (61.4%) patients. The distribution of *Aspergillus* species consisted of *Aspergillus niger* 22.1%, *Aspergillus fumigatus* 17.9%, and *Aspergillus flavus* 7.9%. The proportion haemoptysis (65% vs. 35%), shortness of breath (61.4% vs. 38.6%), chest pain (64% vs. 36%) and weight loss (54% vs. 46%) were higher in those with positive culture of *Aspergillus* spp. compare to patients with negative culture of *Aspergillus* spp., although this result were not statistically significant.

**Conclusions:** *Aspergillus* spp. is commonly isolated from NSCLC patients. Haemoptysis, shortness of breath, chest pain and weight loss might indicate early phase of *Aspergillus* spp. infection in this population although definitive diagnosis of IA in this study required radiology and other mycology laboratory tests.

## P259 Clinico-Microbiological and Antifungal Susceptibility Profile of Cryptococcal Infections in Non-HIV Non-Transplant Patients

**Malini Capoor, Annapurna Parida, Parul Varshney, Ranghvendra Samudralaya and BK Tripathi**

VMMC and Safdarjung Hospital, Delhi

**Objectives:** Cryptococcal infections remain undiagnosed or misdiagnosed till it becomes fulminant invasive Cryptococcosis. Studies pertaining to cryptococcossis in non-HIV non-transplant patients are scarce. The objectives of the study was to know the prevalence, clinico-mycological profile, antifungal susceptibility and to characterize through DNA sequencing cryptococcal isolates from non-HIV, non-transplant patients.

**Materials & Methods:** The demographic profile, clinical presentation, predisposing condition, radiological features, pertinent laboratory findings, Other opportunistic infections (OIs), course, treatment, outcome were obtained. Cryptococcosis was confirmed by either positive culture or presence of cryptococcal organism identified on microscopy, Latex agglutination test (LAT).Cryptococcal isolates was identified by conventional and molecular methods. Minimum inhibitory concentration of amphotericin B, fluconazole, itraconazole, voriconazole, posaconazole was performed by broth micro dilution method (BMD) as per CLSI guidelines. For DNA sequencing genomic target amplified were ITS-1, ITS-2 and ITS-4 region.

**Results:** Out of total of 22 HIV negative transplant negative patients, disseminated cryptococcosis seen in three: isolated from CSF (3), BAL (1) and urine (1), soft tissue (1). Most common presentation was meningitis (20), primary pulmonary (1), soft tissue (1). Five pts expired even after initiation of AFT, in one case relapse occurred after completion of treatment. Among HIV negative patients known risk factors were leucopenia (9), neutropenia (2), Hodgkin’s lymphoma (1), lymphocytosis (2), tuberculosis (1) and apparently immunocompetent (7). On DNA Sequencing: 22 isolates were subjected to DNA sequencing: *C. neoformans* var. *grubii* (Serotype A) AFLP I/VNI (19), *C. neoformans* serotype D (var. *neoformans*) AFLP II/VNIV (1), *C. gattii* serotype B VGI (var. *gattii*) (2). The MIC 50 and MIC 90 for Cryptococcal isolates for amphotericin B was 0.25 μg/mL and 0.5 μg/mL, respectively and 1 μg/mL and 4 μg/mL for fluconazole. *C. gattii* isolates had higher MIC’s. Three patients from whom *C. gattii* (2) expired after 2–4 weeks of antifungal therapy and thus the mortality was more as compared to those patients in whom *C. neoformans* var. *neoformans* was isolated.

**Conclusions:** Cryptococcossis in HIV negative and transplant negative is not rare. Mortality rate is high amongst HIV negative patients. Dual pathology of tuberculosis and cryptcoccosis in both HIV negative patients is unique to Asia including India. *Cryptococcosis neoformans var. grubii* is predominant serotype. Identification of Cryptococcus to species level is important. The regimens for both species are similar but *C. gattii* may require prolong therapy owing to severity, extensive CNS involvement, simultaneous pulmonary involvement and high MIC. Significant population of apparently healthy hosts with cryptococcosis with no immunologic defects calls for in depth immunological studies of these patients.

## P260 Improving Diagnosis of Invasive Mycoses in Immunocompromised Patients by ITS Sequencing

**Galina Solopova, Olesya Kozhushnaya, Alexander Voropaev, Zhanna Markova and Galina Novichkova**

Dmitry Rogachev National Research Center For Pediatric Hematology, Oncology and Immunology

**Objectives:** Mould identification in clinical specimens can be difficult due to the low efficiency of cultural methods—up to 50% cases of morphological detection of hyphae without microbiological confirmation. Some species are not included in MALDI-TOF MS spectral databases. The aim of the study was to assess the possibility of introducing and potential benefits of fungal internal transcribed spacers (ITS) sequencing for the diagnosis of invasive mycoses in immunocompromised patients.

**Materials & Methods:** The study was conducted at Dmitry Rogachev National Medical Research Center of Pediatric Hematology, Oncology and Immunology (NMRC PHOI) in 2021. Study was performed on 5 patients with probable/proven invasive mycosis of paranasal sinuses or lungs, when microbiological diagnosis was difficult (data is presented in Table 1). Fluorescent microscopy with calcofluor white staining (CFW), culture on Sabouraud and Czapek-Dox agar were performed for all specimens and galactomannan (GM) levels were tested in broncho alveolar lavage (BAL). In case of positive culture, colonies were examined morphologically and by MALDI-TOF MS. DNA was extracted either directly from clinical specimens or from pure fungal culture with DNeasy Plant Mini Kit (Qiagen). PCR was performed with direct primer ITS1 (5′ TCC GTA GGT GAA CCT GCG G ′3) and reverse primer ITS4 (5′ TCC TCC GCT TAT TGA TAT GC ′3). Sequencing was performed using ABI 3500XL Genetic Analyzer (Applied Biosystems, USA). The data were analyzed with Sequencing Analysis 7 software and aligned with MEGA7 and nucleotide BLAST. Homologous sequences were searched and retrieved from GenBank database (http://www.ncbi.nlm.nih.gov).

**Results:** Three patients (cases 1, 2, 3) with rhino-orbital mucormycosis were confirmed by CFW microscopy and of these only one case was a fungi culture—*Lichtheimia* spp. With ITS sequencing, all three cases were confirmed with identification *Mucor circinelloides. Lichtheimia ramosa* and *Rhizopus oryzae*. Two more patients (cases 4, 5) were with probable invasive aspergillosis. In the case 4 septate hyphae in BAL were detected (GM negative) and ITS sequencing of BAL and subsequent culture identified *Oxyporus corticola*. This pathogen was described mostly in animals and a literature search revealed only a few cases in immunocompromised patients. The protein spectra were added to the MALDI-TOF MS library for future identification of this species. In case 5 we found very high level of GM only, and identification of *Aspergillus flavus* was done with ITS sequencing of BAL. Out of 5 described cases, in 4 sequencing was performed directly in clinical specimens.

**Conclusions:** ITS sequencing shows promising results in mould identification either directly in clinical specimens or in grown culture to the species level. Thus, using sequencing, the certain pathogen was identified in all cases and accurate diagnosis of invasive mycosis became possible.

## P261 Rapid Molecular Identification of *Pneumocystis jirovecii* Using Liaison MDX Platform Compared to Giemsa Staining in an Over-Loaded Short-In-Staff Laboratory

**Shiraz Halevi, Shoshana Cohen, Ana Listman, Rawan Khashab, Or Kriegr and Sharon Amit**

Sheba Medical Center

**Objective:** *Pneumocystis jirovecii* (PJ) is a ubiquitous fungus that causes a life-threatening infection in immunocompromised patients. Rapid detection of PJ is crucial for the outcome of patients. Traditional identification of PJ through Giemsa staining and direct microscopy is time consuming, requires high expertise, long training period for new staff and has low sensitivity. Molecular tests are much more sensitive than microscopy, however, home-made molecular tests also require expertise and are time consuming especially when small amounts of samples are run frequently in an over-loaded short-in-staff clinical laboratory yet, there is no commercial FDA approved rapid test available. Liaison MDX platform (Diasorin) offers primer pair for identification of PJ enabling 2 strategies of loading of specimen on the amplification discs; direct from sample or after nucleic acids extraction. The direct sample option shortens time to result. Once the Liaison MDX is loaded, it takes 48 min for a result. Here, we assessed the feasibility of this sample to result platform to minimize the “hands on” time of the laboratory staff and its sensitivity compared to direct microscopy.

**Methods:** Between 1 May 2019–13 June 2021 bronchoalveolar lavage (BAL) or induced sputum samples from 258 patients with clinical suspicion of PJ were examined microscopically (Giemsa stain) and/or molecularly using PJ primer pair kit on the Liaison MDX platform. BAL samples were centrifuged for 15 min at 5000 rpm and sediment was diluted in saline. For the molecular test BAL and induced sputum were pre-treated with beads and Sputasol (Thermo Fisher).

**Results:** Out of 137 samples examined by direct microscopy during 1 May 2019–13 June 2020, cystic or trophic forms were definitely detected in 4 samples (2.9%) and one more was inconclusive (adding together to 3.6%) while during 14 June 2020–13 June 2021 twelve (9.9%) samples were positive for PJ out of 121 samples tested with the Liaison MDX PJ assay using the direct sample loading option. Ct values varied between 24 to 36. Out of the 12 positive samples, 6 were examined microscopically; Three samples were positive for cystic or trophic forms microscopically having Ct values of 24, 22.4 and 25, one inconclusive had a Ct value of 31 and 2 negative ones had a Ct value 34.4 and 34.

**Conclusions:** Using the direct sample loading option on the Liaison MDX we found about 20% inhibition of the PCR test. Sputasol treatment of all the samples prior to run decreased inhibition significantly though added time and work to the process. Although the molecular test increased by 2.75-fold sensitivity, our overall positivity rate for PJ using direct microscopy and MDX platform are lower than previously published. In addition, we saw that Ct of ~30 is the limit of detection for direct microscopy. Overall, the Liasion MDX platform is an option for rapid detection of PJ with higher sensitivity than direct Giemsa stained microscopy and less labor.

## P262 Eumycetoma due to *Parathyridaria percutanea* in an Immunosuppressed Patient—An Imported Case in Portugal

**Marta Fraga ^1^, Dinah Carvalho ^1^, Raquel Sabino ^2,4^, Cristina Veríssimo ^2^, Helena Simões ^2^, Guilherme Sapinho^3^, Ana Alho^3^, Eduardo Espada ^3^, Raul Moreno ^3^, João Forjaz de Lacerda ^3^, Luis Marques Lito ^1^ and José Melo Cristino ^1^**

^1^ Centro Hospitalar Universitário Lisboa Norte- Serviço de Patologia Clínica
^2^ Instituto Nacional de Saúde Dr. Ricardo Jorge -Departamento de Doenças Infecciosas, Unidade de Referência de Infeções Parasitárias e Fúngicas
^3^ Centro Hospitalar Universitário Lisboa Norte—Serviço de Hematologia e Transplantação de Medula
^4^ Instituto de Saúde Ambiental, Faculdade de Medicina, Universidade de Lisboa

**Objectives:** Eumycetoma is a chronic subcutaneous fungal infection characterized by swelling, fistulization and discharge of fungal granules. These infections occur after traumatic inoculation and progress slowly over months or even years into a chronic form.

*Parathyridaria percutanea* is a rare cause of subcutaneous phaeohyphomycoses and there are no published reports of discharging sinus or granuloma formation due to this fungus.

We present a rare case of eumycetoma due to *Parathyridaria percutanea* in a 10-years-old patient from Angola, presenting after allogeneic hematopoietic stem cell transplant (alloHSCT) for severe aplastic anemia.

**Material and methods:** A 10-year-old boy from Angola with severe aplastic anemia was transferred to Portugal and submitted to alloHSCT from a mismatched unrelated donor at a single HLA-*locus* (9/10). After secondary graft failure due to post-transplant hemophagocytic lymphohistiocytosis, he underwent a second alloHSCT, without engraftment. Twenty-three days after the 3rd alloHSCT from the same donor and seven days under prophylaxis with posaconazole, the patient presented with febrile neutropenia with no clear focus. He complained of painless swelling of the right knee, with slight fluctuation. An ultrasound was performed revealing a heterogeneous hypoechogenic area with imprecise contours, inside of which there was a nodular formation of approximately 10 mm. Two weeks later, a spontaneous drainage of a grossly spherical grain from the right knee occurred. It was characterized as extra-articular, subcutaneous with filamentous aspect. The material was sent to the laboratory where its macroscopic observation allowed the identification of a granule of possible mycotic etiology, consistent with the diagnosis of eumycetoma. This sample was promptly processed for bacteriology (aerobic and anaerobic) and mycology studies.

**Results:** Direct microscopic examination of the grain revealed septate hyphae with irregular hyphal swellings. Cultures for bacteria (aerobic and anaerobic) were sterile. After 1 week, slow growing colonies appeared on Sabouraud dextrose agar (SDA) incubated at 25 °C. Those colonies were flat, spreading with sparse aerial hyphae, become creamy after 5 to 10 days and with orange reverse and then turned dark brown after 4 weeks. Lactophenol cotton blue mount revealed nonsporulating dematiceous hyphae with clamidospores. Phenotypic identification of the organism was uncertain but sequencing of the internal transcribed spacer (ITS) region of ribosomal DNA identified as *Parathyridaria percutanea* (100% homology, 99% coverage). The isolate showed in vitro resistance to itraconazole, fluconazole and anidulofungin and susceptibility to posaconazole, voriconazole and amphotericin B. As this pathogen is extremely rare, no antifungal guidelines have been proposed so far.

The patient received dual coverage with posaconazole and liposomal amphotericin-B but died 2 weeks later of complications of the underlying disease.

**Conclusions:** The presented case maybe the first described case of eumycetoma caused by *Parathyridaria percutanea* associated with fistulae presentation. Molecular identification of rare fungi is essential, especially when they are poorly sporulated, as in this case. Moreover, rare fungi can present with odd clinical signs, as in this case, emphasizing the relevance of a polyphasic approach to identify the etiological agent.

## P263 Exophiala Dermatitidis Can Undergo Patient-Patient Transmission in Cystic Fibrosis Patients

**Jonathan Ayling-Smith ^1,2^, Gemma Ford ^3^, Lorraine Speight ^1^, Jacques F. Meis ^4^, Rishi Dhillon ^5^, Matthijs Backx ^5^, P. Lewis White ^5^, Kerry Hood ^2^ and Jamie Duckers ^1^**

^1^ Cardiff and Vale University Health Board
^2^ Cardiff University
^3^ Cwm Taf Morgannwg University Health Board
^4^ Centre of Expertise in Mycology
^5^ Public Health Wales Microbiology

**Objectives:** *Exophiala dermatitidis* is an opportunistic yeast. It is assumed that infection and colonisation takes place from the environment. However, new positive cases in a CF unit led to an investigation into historical patient data to establish the following objectives: was transmission was taking place in the environment or was transmission taking place between patients in hospital-facilitated settings.

**Materials & Methods:** A sample of *Exophiala dermatitidis* cultures grown from routine sputum tests were genotyped. Of the 40 isolates analysed, 21 were genetic clones and were cultured from 15 CF patients. A complete list of secondary and tertiary care contact dates for each patient, from the year 2012 (the first growth in the cohort) to the present day was made using health board databases. These dates were cross-referenced and filtered by macrolocation (hospital) and microlocation (ward/department) to identify potential positive patient to negative patient events, termed “clashes”. Potential clashes where both patients tested positive for *E. dermatitidis* less than 6 months later were termed “significant clashes”.

**Results:** The number of outpatient dates reviewed was 1506. Clashes occurred 49 times in outpatients, 12 being significant clashes. The number of inpatient patient-days was 2196. Clashes occurred 17 times on the ward, of which 11 were significant clashes. All of the inpatient clashes involved 5 of the negative patients (36%). 11 negative patients (79%) had clash events and 8 of them (57% of total) were significant clashes. 4 patients had no identifiable clash event before turning positive. No pattern was found in positive patients home postcodes. Environmental swabs taken on the ward were negative.

**Conclusions:** We present data that suggests that *E.dermatitidis* can be transmitted from patient-patient and the most likely location for this within the hospital setting is CF outpatients. There is therefore the possibility of inadequacy in current local infection control policies regarding outpatient clinics.

## P264 Molecular Identification of Fungal Species Causing Surface Mycosis in Patients with Leprosy in a Reference Ambulatory in Southern Brazil

**Maria Lúcia Scroferneker ^1,2^, Alessandra Koehler ^1^, Amanda Carvalho Ribeiro ^3^, Danielle Machado Pagani ^4^, Paulo Cezar Moraes ^1,5^, Rodrigo Vetoratto ^1,6^ and Letícia Maria Eidt^5^**

^1^ Postgraduate Program in Medicine: Medical Sciences, Universidade Federal do Rio Grande do Sul
^2^ Department of Microbiology, Immunology and Parasitology, ICBS, Universidade Federal do Rio Grande do Sul
^3^ Graduation in Pharmacy, Universidade Federal do Rio Grande do Sul
^4^ Postgraduate Program in Agricultural and Environmental Microbiology, Universidade do Rio Grande do Sul
^5^ Sanitary Dermatology Outpatient Clinic from Porto Alegre
^6^ Dermatology Service from the Hospitalar Complex Santa Casa de Misericórdia de Porto Alegre

**Objectives:** Evaluate superficial mycoses in leprosy patients, performing the molecular identification of the fungal species present and evaluating their prevalence.

**Materials & Methods:** 239 patients participated in the research, assisted by the Leprosy Service of the Sanitary Dermatology Outpatient Clinic in Porto Alegre. The samples collected from the lesions were subjected to direct mycological examination and cultural mycological examination. The fungi were isolated on Mycosel agar. Molecular identification was done through DNA extraction with the Power Soil DNA Isolation Kit (Qiagen) and amplification of the ITS region. The PCR product was purified and sequenced and the sequences were analyzed using the BLAST tool (GenBank).

**Results:** At the time of collection, 108 patients had at least one site with a fungal lesion. In total, 64 collections had a positive direct mycological examination and cultural mycological examination. Of these, 36 cultures were positive for dermatophyte filamentous fungus, 8 for non-dermatophyte filamentous fungus and 20 for yeast. So far, 23 dermatophyte filamentous fungus isolates have been molecularly identified, in the species *Epidermophyton floccosum* (n = 1), *Trichophyton interdigitale* (n = 18) and *T. rubrum* (n = 4). For non-dermatophyte filamentous fungus, 5 isolates were identified, in the species *Acremonium brachypenium* (n = 1), *Arthrinium arundinis* (n = 1), *Fusarium keratoplasticum* (n = 2) and *F. oxysporum f. sp. vasinfectum* (n = 1). For yeasts, 20 isolates were identified, in the species *Candida albicans* (n = 2), *C. duobushaemulonis* (n = 1), *C. haemulonis* (n = 1), *C. guilliermondii* (n = 2), *C. metapsilosis* (n = 1), *C. orthopsilosis* (n = 1), *C. parapsilosis* (n = 5), *C. tropicalis* (n = 4), *C. zeylanoides* (n = 1), *Trichosporon montevideense* (n = 1) and *T. dermatis* (n = 1).

**Conclusions:** Leprosy patients have skin lesions that can be a gateway for fungi, generating secondary infections. In fact, the preliminary results allow us to visualize the wide variety of fungi associated with lesions in patients with leprosy. Of these, several can cause more serious systemic infections, which emphasizes the importance of knowledge of the opportunistic fungal species present in the lesions of patients with this serious disease.

## P265 *Mucor circinelloides* Isolated from a Leprosy Patient: A Case Report

**Maria Lúcia Scroferneker ^1,2^, Paulo Cezar de Moraes ^1,3^, Letícia Maria Eidt ^3^, Alessandra Koehler ^1^, Amanda Carvalho Ribeiro ^4^, Danielle Machado Pagani ^5^ and Rodrigo Vetoratto ^1,6^**

^1^ Postgraduate Program in Medicine: Medical Sciences, Universidade Federal do Rio Grande do Sul
^2^ Department of Microbiology, Immunology and Parasitology, ICBS, Universidade Federal do Rio Grande do Sul
^3^ Sanitary Dermatology Outpatient Clinic from Porto Alegre
^4^ Graduation in Pharmacy, Universidade Federal do Rio Grande do Sul
^5^ Postgraduate Program in Agricultural and Environmental Microbiology, Universidade do Rio Grande do Sul
^6^ Dermatology Service from the Hospitalar Complex Santa Casa de Misericórdia de Porto Alegre

**Case report:** A 31-year-old male patient, residing in a junkyard in the municipality of Sapucaia do Sul, Rio Grande do Sul, Brazil, with previous history of alcohol and drug abuse without other known comorbidities, was presented to the Sanitary Dermatology Outpatient Clinic from Porto Alegre. The patient reported ulcerated lesions in the lower limbs for 4 years, nodular lesions in the arms and legs for 3 years, weight loss of ±10 kilograms in the last 6 months, complains of paresthesia in the lower limbs and pain in ulcerated lesions. On examination, nasal collapse, septum perforation, madarosis, infiltration of eyebrows and bilateral pinna, hardened erythematous nodules in the forearms, arms and lower limbs, deep ulcers (grade IV) in the lower limbs and onychomycosis in the toenails were observed. The patient presented a biopsy positive for multibacillary virchowian leprosy. For sample collection for mycological examination, the nail area was cleaned with 2% chlorhexidine, the lesion was scarified and scraped with a curette, the material was stored in sterile Petri dishes sealed with tape and taken to the laboratory. For direct mycological examination, the material was clarified with potassium hydroxide and observed under an optical microscope, with hyphae visualization. The fungus was isolated on Mycosel agar, grown at 30 °C for 14 days. For molecular identification, DNA extraction and genetic sequencing of the ITS region was performed. GenBank basic local alignment search tool (BLAST) searches were performed for species identification, resulting in *Mucor circinelloides* with 99% of query cover and 98.85% of identity. The treatment of leprosy was initially performed with a 24-dose multibacillary replacement scheme (single supervised monthly doses of 600 mg rifampicin, 400 mg ofloxacin and 100 mg of minocycline). For treatment of onychomycosis, miconazole cream was prescribed for use for 3 months, in the three times the patient came for consultation. However, due to the long interval between consultations and lack of follow-up, the treatment was not completely effective for healing.

**Discussion and conclusions:** Leprosy is an infectious disease caused by *Mycobacterium leprae* that preferentially attacks skin and peripheral nerves. Late diagnosis can lead to sequelae due to cutaneous, ophthalmic and neural manifestations resulting from inflammation caused by bacilli. During this period, patients have impaired immunity due to the systemic use of corticosteroids and antibiotics, making them susceptible to other opportunistic microorganisms such as fungi that cause onychomycosis. The species *M. circinelloides* belongs to the phylum Zygomycota, a group of fungi rarely isolated from clinical samples. The genus *Mucor* comprises opportunistic fungi that cause infection mainly in individuals who have diseases that cause immunosuppression. To the best of our knowledge, this is the first report of an infection of a fungus of the genus *Mucor* in a leprosy patient. There are reports of *M. circinelloides* causing outbreaks of onychomycosis in workers who dealt with oranges infected by the fungus. However, the species can also cause invasive infections, the most common forms being rhinocerebral and pulmonary. Therefore, it is important to constantly monitor infections caused by *M. circinelloides*, especially in immunocompromised patients.

## P266 Invasive Infections Caused by Rare Molds: Result of Prospective Study in Saint-Petersburg, Russia

**Sofya Khostelidi ^1^, Olga Shadrivova ^1^, Tatiyana Bogomolova ^1^, Marina Popova ^2^, Ludmila Zubarovskaya ^2^, Elmira Boychenko ^3^, Nadezhda Medvedeva ^4^, Iliya Zjuzgin ^5^, Margarita Belogurova ^6^, Olga Uspenskaya ^7^, Vyacheslav Semelev ^8^ and Nikolay Klimko ^1^**

^1^ I.I. Mechnikov North-West State Medical University
^2^ I.P.Pavlov St. Petersburg State Medical University
^3^ Children’s City Hospital No. 1
^4^ City Hospital No. 31
^5^ N.N. Petrov Research Institute of Oncology
^6^ Clinical Scientific and Practical Center of Specialized Types of Medical Care
^7^ Leningrad Regional Clinical Hospital
^8^ S.M. Kirov Military Medical Academy

**Background:** Invasive fungal infections caused by rare molds micromycetes are severe opportunistic infections with high mortality. Publications about invasive fungal infections caused by rare molds micromycetes are limited.

**Objective:** To analyze the demographic parameters, risk factors, etiology, and results of treatment of invasive infections caused by rare molds in St. Petersburg, Russia.

**Methods:** The prospective study during 2000–2021 yy. The diagnosis of mycosis was made according to EORTC/MSGERC criteria (2020).

**Results:** In the study were included 75 patients from 8 hospitals in Saint-Petersburg. The median age of patients was 33,5 years (range 1–78), male/female ratio—2:1 (males—63%, females—37%), children—30%.

The main underlying conditions were hematological diseases—73% (AML—34%, ALL—24%, AL—2%, Non-Hodgkin’s lymphoma—12%, Hodgkin’s lymphoma—8%, CLL —8%, CML—4%, MDS—4%, multiple myeloma—4%), COPD—7%, solid malignancy—5%, AIDS—4%, trauma/burns —7%, autoimmune diseases—1%, organ transplantation—1%, drug induced neutropenia—1%, septic endocarditis—1%, diabetes mellitus—1%. The main risk factors were severe neutropenia (73%), corticosteroids use (62%), allogeneic hematopoietic stem cells transplantations (19%), surgery (15%), and ICU (15%). The main clinical forms were pneumonia (71%), disseminated infection (21%), sinusitis (15%), CNS infection (4%), and subcutaneous infection (4%).

Diagnosis was established by histology in 25% patients. In all cases the diagnosis was confirmed with culture. Etiologic agents were *Paecilomyces* spp. (25.3%), *Fusarium* spp. (17.3%), *Acremonium* spp. (16%), *Trichoderma* spp. (9,3%), *Alternaria* spp. (9.3%)*, Exophiala* spp. (6%), *Aureobasidium pullulans* (5.3%), *Trichosporon* spp. (4%), *Scedosporium* spp. (3%), *Scopulariopsis* spp. (3%), *Cladosporium* sp. (1.3%).

Antifungal therapy (voriconazole—72% patients, amphotericin B deoxycholate—26%, itraconazole—17%, caspofungin—17%, amphotericin B lipid complex—11% and posaconazole—11%) was used in 73% patients, post-mortem were diagnosed 27% cases. Combination therapy was used in 62% patients, surgical treatment—15%. The overall survival of patients in 12 weeks was 62%.

**Conclusions:**

1. Main underlying diseases patients with invasive fungal infections caused by rare molds micromycetes were hematological diseases—73% The main risk factors were severe neutropenia (73%), corticosteroids use—(62%), and allogeneic hematopoietic stem cells transplantations (19%).
2. The main pathogens were *Paecilomyces* spp. (25.3%), *Fusarium* spp. (17.3%), and *Acremonium* spp. (16%).
3. The main clinical forms were pneumonia (71%), disseminated infection (21%), and sinusitis (15%).
4. The overall survival of patients in 12 weeks was 62%.

## P267 Invasive Infections Caused by Rare Yeasts: Result of Prospective Study in Saint-Petersburg, Russia

**Sofya Khostelidi ^1^, Elena Shagdileeva ^1^, Tatiyana Bogomolova ^1^, Marina Popova ^2^, Aleksey Kolbin ^2^, Ludmila Zubarovskaya ^2^, Elmira Boychenko ^3^, Nadezhda Medvedeva ^4^, Margarita Belogurova ^5^, Andrey Saturnov ^6^, Aleksey Kuzmin ^7^ and Nikolay Klimko ^1^**

^1^ I.I. Mechnikov North-West State Medical University
^2^ I.P.Pavlov St. Petersburg State Medical University
^3^ Children’s City Hospital No. 1
^4^ City Hospital No. 31
^5^ Clinical Scientific and Practical Center of Specialized Types of Medical Care
^6^ Leningrad Regional Clinical Hospital
^7^ S.P. Botkin Clinical Hospital for Infectious Diseases

**Background:** Publications about invasive infections caused by rare yeasts are limited.

**Objective:** To analyze the demographic parameters, risk factors, etiology, results of treatment of invasive infections caused by rare yeasts in St. Petersburg, Russia.

**Methods:** The prospective study during 2000–2021 yy. The diagnosis of mycosis was made according to EORTC/MSGERC criteria (2020).

**Results:** In the study were included 37 patients from 7 hospital in Saint-Petersburg. The median age of patients was 18 years (range 1–60), children—42%. Male/female ratio was 2:1 (males—70%, female—30%). The main underlying conditions were hematological diseases (36%), surgery (27%), solid malignancy (16%), AIDS (11%), and trauma/burns (11%). The main risk factors were prolonged stay in the ICU (89%), central venous catheter use (92%), and cytostatic chemotherapy with neutropenia and lymphocytopenia (51%).

Main clinical forms were fungemia (78%), CNS infection (16%), mycotic peritonitis (3%), and isolated skin and deep tissue infection (3%). Diagnosis was established by positive result of culture of blood (78%), cerebrospinal fluid—16%, peritoneal fluid—3%, and tissue biopsy—8%. Direct microscopy and hystology of BAL, CSF, and other samples were positive in 20% of patients.

In all cases the diagnosis was confirmed by culture. Etiologic agents were *Rodotorula* spp. (46%), *Trichosporon* spp. (30%), *Malassesia* spp. (8%), *Geotrichum* spp. (8%), *Saccharomyses* spp. (5%), and *Debaryomyces* sp. (3%).

Antifungal therapy (fluconasole—43%, voriconazole—29%, amphotericin B deoxycholate—16%, echinocandins—16%) was used in 89% patients, post-mortem were diagnosed 11% cases. The median duration of antimycotic therapy was 23 days (3–98). The overall survival of patients in 30 days was 63%, 12 weeks—31%.

**Conclusions:**

1. In patients with invasive infections caused by rare yeasts the main underlying conditions were hematological diseases (36%) surgery (27%), and solid malignancy (16%).
2. The main risk factors were prolonged stay in the ICU (89%), central venous catheter use (92%), and cytostatic chemotherapy with neutropenia and lymphocytopenia (51%).
3. The main pathogens were *Rodotorula* spp. (46%) and *Trichosporon* spp. (30%).
4. The main clinical forms were fungemia (76%) and CNS infection (16%).

The overall survival of patients in 30 days was 63%.

## P268 Pulmonary Cryptococcosis in Patients with Idiopathic CD4+ Lymphocytopenia

**Julia Melekhina, Sofia Khostelidi, Yuliya Borzova, Ekaterina Desyatik, Irina Bekhtereva, Yurii Avdeenko, Ekaterina Frolova, Tatyana Bogomolova, Svetlana Ignatieva, Natalya Vasilyeva and Professor Nikolay Klimko**

North-Western State Medical University Named After I.i. Mechnikov

**Objectives:** Clinical cases of successful treatment of pulmonary cryptococcosis in patients with idiopathic CD4+ lymphocytopenia.

**Materials & Methods:** We present two cases of isolated pulmonary cryptococcosis in patients with idiopathic CD4+lymphocytopenia. The EORTC/MSG 2020 diagnostic criteria were used.

**Results:** Patient, 37 years old, was hospitalized complaining of hemoptysis. He received anti-TB treatment for 3 months without any positive dynamics. Left lung lobectomy was performed. The diagnosis of pulmonary cryptococcosis was established based on histological examination, and positive Crypto Plus test in blood serum. Immunological examination of blood revealed a decrease number of CD4+ cells (0.046 cells/mcl). *Cryptococcal Meningitis* and HIV-infection were excluded. Idiopathic CD4+ lymphocytopenia was diagnosed. Fluconazole 400 mg/day was used with long remission of cryptococcosis.

Patient V., 59 years old, was hospitalized complaining of dry cough. Two years before the patient underwent rectal resection and segmentectomy of the liver due to rectal adenocarcinoma with distant metastases. He received 8 courses of polyochemotherapy according to the XELOX scheme. After 2 years, changes in the right lung were detected on computed tomography. Tuberculosis was excluded. The right lung upper lobe was resected. The diagnosis of pulmonary cryptococcosis was established based on histological examination, a positive Crypto Plus test in bronchoalveolar lavage. Immunological examination of blood revealed a decrease absolute number of CD4+T cells (135 cells/mcl). *Cryptococcal Meningitis* and HIV-infection were excluded. Idiopathic CD4+ lymphocytopenia was diagnosed. The patient received fluconazole 400 mg/day with long remission of cryptococcosis.

**Conclusions:** Idiopathic CD4 + lymphocytopenia should be excluded in patients with pulmonary cryptococcosis. Surgical removal of the lesion and antifungal prevention of relapse can be a successful treatment.

## P269 Disseminated Cryptococcosis in the COVID-19 Pandemic: Case Report of a Late Diagnosis

**Jessica Benelli ^1,2^, Rossana Basso ^1,2^, Marcia Rodrigues ^1,2^, Vanice Poester ^1^, Livia Munhoz ^1^ and Melissa Xavier ^1,2^**

^1^ Universidade Federal Do Rio Grande
^2^ HU-FURG/EBSERH

**Objectives:** Deaths associated with COVID-19 pandemic, started in December 2019, is already up to 3.5 million around the world. Besides that, other diseases are being neglected due to the overwhelming of the healthcare systems, also promoting indirect victims by infectious or chronic disease which could be prevented and avoided. Some authors refer to this impact on public health, as an “invisible epidemic”. Here we report a case of a fatal disseminated cryptococcosis in an HIV infected patient with a late diagnosis of both infections. **Case report:** A male patient, 37 years old, English teacher, homosexual, was referred to the HU-FURG/EBSERH, with a historical of fever, enlarged cervical nodes, asthenia, adynamia and anorexia for around 30 days. He also reported headache with a moderate intensity, that had worsened in the last 10 days, and an ulcerated lesion near to the scalp. He had been in at least 3 outpatients visits in the last month, being submitted exclusively to a COVID-19 investigation (RT-PCR and antigen test—with negative results). Patient was admitted to the hospital to a better diagnosis investigation and in 48 h, he had an episode of loss of consciousness and left deviation of the rima oris. Serological tests resulted in a HIV reagent test and a VDRL titer of 1:8. Large and spherical blastoconidia were detected from a sample (swab) of the ulcerated skin lesion, as well as from the cerebrospinal fluid (LCR) evaluated by India ink test. *Cryptococcus neoformans* grew in Sabouraud dextrose agar, being confirmed by MALDI-TOF. In addition, polyomavirus BK virus (BKV) was detected on LCR by real-time polymerase chain reaction. Besides the antifungal therapy with Amphotericin B deoxycholate 50 mg/d and fluconazole 800 mg/d, and intramuscular benzathine penicillin G 2,400,000 IU initiated, the patient had clinical worsening with deterioration of his consciousness state in 24 h and died after 48 h of hospitalization. **Conclusions:** We present a case of disseminated cryptococcosis as an AIDS-defining disease in a patient, which had evolved for more than 30 days. In the context of the COVID-19 pandemic, his full diagnostic investigation was neglected, resulting in a severe disease progressing to death with a late diagnosis. The co-infection with BKV, increases the evidences of a high degree of immunosuppression on our patient. The case reported shows another indirect victim of the COVID-19 pandemic, warning about the danger in neglecting systemic severe fatal diseases.

## P270 Fatal Rhino-Orbital Mucormycosis Complicating Successful Treatment of COVID-19: The Double-Edged Sword of Immunomodulary Interventions

**Sara Georgiadou ^1^, Aggelos Stefos ^1^, George Giannoulis ^1^, George Vatidis ^1^, Despoina Stergioula ^1^, Stella Gabeta ^1^, Vasiliki Lygoura ^1^, Athanasios Saratziotis ^2^, Jiannis Hajiioannou ^2^, George Ntaios ^1^, Nikolaos Gatselis ^1^ and George Dalekos ^1^**

^1^ Department of Medicine and Research Laboratory of Internal Medicine, National Expertise Center of Greece in Autoimmune Liver Diseases, University Hospital of Larissa, University of Thessaly
^2^ Otorhinolaryngology Department—Head and Neck Surgery, University Hospital of Larissa, University of Thessaly

**Objectives:** COVID-19 has been associated with several complications including secondary bacterial and fungal infections. Treatment protocols for COVID-19 pneumonia and related cytokine storm have been proposed as a cause of several opportunistic infections. We present a patient with acute, fatal rhino-orbital mucormycosis following treatment of COVID-19 pneumonia and hyperinflammatory syndrome.

**Methods/Results:** A 72-year-old man with a history of uncontrolled diabetes, end stage chronic kidney disease and chronic alcohol abuse was admitted to our department due to 2-days pyrexia, exfoliating skin rash, new onset gradually developed exophthalmos and ophthalmoplegia bilaterally, inflammation of the facial soft tissue and hard palate’s necrotic lesions. He had been recently treated with prednisolone, intravenous infusions of γ-globulin and tocilizumab due to COVID-19 pneumonia with accompanied macrophage activating syndrome, after infection by the novel severe acute respiratory syndrome coronavirus 2 (SARS-CoV-2). Computed tomography of the skull revealed ethmoid and sphenoid sinuses occupation, as well as left sphenoid bone erosions without lung involvement. The clinical diagnosis of invasive fungal infection was presumed and he was started on high doses of intravenous liposomal amphotericin B. Moreover, endoscopic maxillary antrostomy, ethmoidectomy and sphenoidotomy were performed bilaterally under general anaesthesia and diagnosis was finally established by histopathological examination that revealed aseptate broad hyphae and sporangia containing sporangiospores compatible with invasive mucor disease. Our patient was admitted to the intensive care unit where second surgical intervention was performed, but unfortunately he died due to septic shock and multiorgan failure.

**Conclusions:** Immune dysregulation caused by SARS-CoV-2, in combination with patients’ poor health status and immunomodulatory medical interventions can cause fatal opportunistic invasive fungal infections, such as mucormycosis. Physicians should be aware of the possible risk factors in the era of COVID-19, as the prognosis is poor and prompt diagnosis is of outmost importance.

## P271 Antifungal Susceptibility of Fungal Isolates Causing Surface Mycosis in Patients with Leprosy in a Reference Ambulatory in Southern Brazil

**Maria Lúcia Scroferneker ^1,2^, Amanda Carvalho Ribeiro ^3^, Alessandra Koehler ^1^, Danielle Machado Pagani ^4^, Paulo Cezar Moraes ^1,5^, Rodrigo Vetoratto ^1,6^ and Letícia Maria Eidt^5^**

^1^ Postgraduate Program in Medicine: Medical Sciences, Universidade Federal do Rio Grande do Sul
^2^ Department of Microbiology, Immunology and Parasitology, ICBS, Universidade Federal do Rio Grande do Sul
^3^ Graduation in Pharmacy, Universidade Federal do Rio Grande do Sul
^4^ Postgraduate Program in Agricultural and Environmental Microbiology, Universidade do Rio Grande do Sul
^5^ Sanitary Dermatology Outpatient Clinic from Porto Alegre
^6^ Dermatology Service from the Hospitalar Complex Santa Casa de Misericórdia de Porto Alegre

**Objectives:** Evaluate the in vitro antifungal susceptibility of fungi isolated from lesions of leprosy patients.

**Materials & Methods:** 239 patients participated in the study, assisted by the Leprosy Service of the Sanitary Dermatology Outpatient Clinic in Porto Alegre. The collected samples were subjected to direct mycological and cultural mycological examination, and identified by genetic sequencing. The profile of sensitivity to clinical antifungals was assessed following protocols M38-A2 and M27-A3 of the Clinical and Laboratory Standards Institute (CLSI). The minimum inhibitory concentrations (MIC) of 8 antifungal agents for dermatophyte filamentous fungi were determined: terbinafine (0.5–0.001 μg/mL), cyclopyroxolamine (32–0.06 μg/mL), fluconazole and griseofulvin (64–0.125 μg/mL), ketoconazole, itraconazole, posaconazole and voriconazole (16–0.03 μg/mL); and MIC of 6 antifungals for non-dematophyte filamentous fungi and yeasts: caspofungin (8–0.015 μg mL), fluconazole (64–0.125 μg/mL), ketoconazole, itraconazole, posaconazole and voriconazole (16–0.03 μg/mL).

**Results:** 48 isolates were identified molecularly: 20 yeasts, 23 dermatophyte filamentous fungi and 5 non-dematophyte filamentous fungi. So far, all non-dermatophyte samples were resistant to the tested antifungals. Of the tested dermatophytes, only four isolates were sensitive to fluconazole (MIC between 2–4 μg/mL), with most of them showing resistance to this antifungal (MIC between 8–64 μg/mL). Of the yeasts tested, only four isolates were sensitive to itraconazole (MIC 0.03 μg/mL), the others showed drug resistance (MIC between 0.06–16 μg/mL).

**Conclusions:** Itraconazole and fluconazole are antifungal agents widely used to treat superficial mycoses, however a large number of the tested isolates showed in vitro resistance to these drugs. This shows the relevance of studies evaluating the antifungal susceptibility of fungi associated with lesions in patients with leprosy, since they are predisposed to infections.

## P272 Cases of Chronic Pulmonary Aspergillosis (CPA) after COVID-19

**Elena Shagdileeva, Evgenya Zaytseva, Olga Shadrivova, Natalya Nikolaeva, Ekaterina Desyatik, Vladimir Mitrofanov, Yuliya Borzova, Ekaterina Frolova, Larisa Filippova, Alexandra Uchevatkina, Olga Shurpickaya, Svetlana Ignatyeva, Tatyana Bogomolova and Nikolay Klimko**

North-West State Medical University named after I. I. Mechnikov

**Objectives:** It is well known that patients with severe COVID-19 may develop invasive aspergillosis. We present two clinical cases of CPA after COVID-19.

**Materials & Methods:** For the CPA’s diagnosis clinical and laboratory criteria ECMM/ESCMID/ERS 2016 were used. COVID-19 diagnosis was confirmed with PCR test.

**Results:** Clinical case No. 1. Woman, 53 years old, with past medical history of hypertension, impaired glucose tolerance and chronic pyelonephritis, in July 2020 suffered bilateral polysegmental pneumonia caused by SARS-Cov-2. She received an antibacterial treatment, glucocorticoids and oxygen therapy, with a positive clinical effect. Two chest CT scans were performed in 2 month. On second CT scan a cavity with a soft-tissue component was visualized (Figure 1). At November 2020 she was admitted to the mycological clinic due to complaints of subfebrile temperature, increased dyspnea with exertion and general weakness. Based on the clinical manifestations and examination results (aspergilloma on the CT scan and positive Aspergillus IgG test (1:400) in blood serum), CPA was diagnosed and treatment with voriconazole was started. Currently, the patient’s condition is satisfactory.

Clinical case No. 2. A 66-year-old woman with medical history of rheumatoid arthritis, type 2 diabetes, diabetic neuropathy, and hypertension, in June 2020 suffered COVID-19 with destructive pneumonia of the left lung lower lobe. She received glucocorticoids and antibacterial treatment, with a positive clinical effect. On the control chest CT scan was visualized a thick-walled cavity with a soft-tissue component (Figure 2). **At September 2020** he was admitted to the mycological clinic due to complaints of cough and severe general weakness. CPA was diagnosed based on CT-signs (aspergilloma), and a positive microscopy for *Aspergillus* mycelium in the bronchoalveolar lavage (BAL). A patient received voriconazole with a positive clinical effect.

**Conclusions:** Chronic pulmonary aspergillosis can develop after COVID-19. If chronic cavities are detected on chest CT scan, mycological examination is required: a test for *A. fumigatus* IgG in blood serum, microscopy, culture of sputum/BAL and galactomannan test in BAL.

## P273 Fungal Coinfections in Critically Ill Patients with Coronavirus Disease 2019 (COVID-19)

**Marija Jandrlić**

Department Of Clinical And Molecular Microbiology

**Objectives:** As a human-to-human transmitted disease, COVID-19 has been pandemic across the whole world. The novel coronavirus (SARS-CoV-2) is equipped by special abilities to spread and dysregulate the immune mechanisms. Severe or critical COVID-19 is associated with intensive care unit admission, increased secondary infection rate, and leads to significant worsened prognosis.

**Materials & Methods:** Retrograde analysis of demographic, clinical and mycological data of patients treated at the COVID unit (30 beds) and ICU (18 beds) of University Hospital Center Zagreb from 30 December 2020 to 30 April 2021. SARS-CoV-2 infection was confirmed by positive polymerase chain reaction (PCR) testing of nasopharyngeal or respiratory secretion samples. All patients had at least one mycology sample.

**Results:** A total of 243 patient samples were analyzed from the COVID unit and ICU (89 36.6%, 154 63.4%). 77 31.7% patients died and 166 68.3% patients were discharged or transferred to another hospital. Symptoms of COVID-19 disease can be classified as mild 52.7%, moderately severe 16.9% and severe 30.5%. During hospitalization, patients were breathing spontaneously 26.7% or had oxygen supplementation 41.6%, HFNC 9.1% (high flow nasal cannula), MV 21.4% (intubation and invasive mechanical ventilation), ECMO 1.2% (extracorporeal membrane oxygenation). Fungal isolates were not detected in 56 patients, while in 187 patients more than one fungus was isolated. Fungal colonization was confirmed in 186 patients and invasive fungal infection in 48 patients. The most frequently isolated fungi were *Candida* 184 patients, *Aspergillus* 26 and saprophytic molds 12. The patients 42 17.3% were treated with antifungal drugs.

**Conclusions:** Patients hospitalized with COVID-19 had a high incidence of secondary infections. These coinfections lead to death of a significant number of patients with COVID-19 due to complications following the primary viral disease.

## P274 *Candida glabrata* Endocarditis—A Therapeutic Dilemma

**Kin Ki Jim ^1^, Joelle J.N. Daems ^2^, S. Matthijs Boekholdt ^2^ and Karin van Dijk ^1^**

^1^ Department of Medical Microbiology and Infection Prevention, University of Amsterdam, Amsterdam UMC
^2^ Department of Cardiology, University of Amsterdam, Amsterdam UMC

**Background:** *Candida glabrata* endocarditis is a rare but serious entity. Here we report a case of *C. glabrata* prosthetic valve endocarditis illustrating a therapeutic dillemma for Candida endocarditis caused by multidrug-resistant isolates.

**Case presentation:** A 47-year old male patient with a surgically corrected Tetralogy of Fallot and pulmonary valve replacement (BioPulmonic Conduit) was admitted to the cardiology ward for treatment of his progessively worsening symptomatic heart failure. His medical history reports multiple other co-morbidities including supravalvular (prothetic pulmonary valve) stenosis, supraventricular tachycardia, atrial fibrillation, diabetes mellitus type I, and intellectual disability. No history of IV drug use or recent immunosuppressive therapy was reported. On admission he received diuretic treatment and a percutaneous intervention was planned to treat the supravalvular pulmonary stenosis. However, ten days after admission he developed fever (40.5 °C) with elevated C-reactive protein (237.9 mg/L) and leukocytosis (14.0 × 10^9^/L). Bloodcultures showed growth of *C. glabrata*, susceptible to only echinocandines (MIC 0.016 mg/L) and Amphotericin B (MIC 0.38 mg/L)as determined by antifungal susceptibility testing. A cardiac CT showed pulmonary valve vegetations and PET-CT scan showed increased FDG uptake in the pulmonary prosthetic valve, and the diagnosis *C. glabrata* prosthetic valve infective endocarditis was made. Anidulafungine IV was started and follow-up bloodcultures were taken routinely. The patient improved clinically and his inflammation markers normalized. However, the bloodcultures were persistently positive, albeit with periods of negative cultures, despite adequate treatment with high-dose anidulafungin 200 mg IV daily. International guidelines recommend surgery to treat Candida prostethic valve infective endocarditis and/or long-term suppressive therapy with antifungal drugs. Because of the of high mortality and morbidity rate associated with cardiac surgery in these patients a multidisciplinary team decided, in consultation with the patient and his family, that the risk of surgical complications outweigh the potential benefits. Therefore, long-term suppressive therapy with an echinocandine and/or Amphothericin B via IV route was the only remaining option, with potential adverse effects such as toxicity, metastatic abscessses and relapse. However, there has been very limited evidence that long-term suppresive therapy with an echinocandine and/or Amphothericin B has a more favorable outcome in these patients.

**Conclusions:** *C. glabrata* endocarditis is a rare but serious and life-threathening disease and often cause a therapeutic dillemma. Therefore, the urge for better (oral) treatment strategies against resistant invasive Candida infections remains high.

## P275 Mysterious-Coccus Alias Cryptococcus Revealed! Under what Circumstances?

**Valentina Ruxandra Moroti Constantinescu ^1^, Georgiana Neagu ^2^, Irina Penescu ^3^ and Serban Nicolae Benea ^1^**

^1^ Carol Davila University Of Medicine And Pharmacy
^2^ National Institute for Infectious Diseases ‘Matei Bals’
^3^ Fundeni Clinical Institute

**Objective:** To observe the features of cryptococcal infection in central nervous system, in HIV patients.

**Material&Methods:** In 11 years of surveillance (2006–2017), in a tertiary infectious diseases’ Romanian hospital, were admitted 67 cases of cryptococcal meningoencephalitis (C-ME) in HIV patients.

**Results:** In the analysed period, there were little changes regarding the temperature&humidity per year, but 2008 was the draughtiest year (16 inches of rain fall, vs. the annual average of 25 inches) and registered the higher number of cases (11 out of 67).

There was a predominance of the cases from urban area (61%), a possible explanation being the crowded birds’ population—pigeons in our cities, that transmit the fungi by their excreta.

Our patients’ sex-ratio was 1.57:1 male:female—a clear masculine predominance. Testosterone is suggested to have an immunosuppressor role and oestrogens, by contrary, could augment the phagocytosis.

The mean age was 30-year-old [16–65], the median = 26-year-old. There was a maximum of C-ME in young adults, with 1.4:1 case in patients <27-year-old. Intriguingly, no C-ME was registered in children, even though the HIV-positive children are largely addressed to our hospital. We had no case under 16-year-old and there were just four cases in patients aged 16 and 17.

Regarding the HIV diagnosis, 20 patients were concomitantly diagnosed with HIV and C-ME and another five in the first year after the HIV diagnosis. The majority (79%) were diagnosed with C-ME in the first 10 years from HIV diagnosis. Except one patient (related below), all were without a HIV proper treatment (because of concomitant diagnosis or non-adherence).

Eighty-six percent had a CD4 < 100/mm^3^. All but two patients had CD4 < 200/mm^3^: a 26-year-old girl, previously diagnosed with HIV two years ago, CD4 = 292/mm^3^, undetectable viral load (VL)—a surprising case, with a good evolution and no further detailed identification of *Cryptococcus* species, and a 40-year-old woman, previously diagnosed with HIV, non-adherent, with CD4 = 282/mm^3^ and VL = 68700 c/mL; good outcome.

Ninety-five percent had detectable VL. There were three cases with VL < 1000 c/mL—one case related above; another 19-year-old girl with VL = 47 c/mL, concomitantly diagnosed with HIV and favourable outcome; a 33-year-old man, concomitantly diagnosed with HIV, VL = 68/mm^3^ and favourable evolution.

The C-ME main symptoms were headache—38,89%, fever—18,06% and vomiting—18.06%. Just 2.78% had nuchal rigidity.

CSF parameters: 130 ± 165 cells/mm^3^, glucose = 44 ± 21mg/dL, proteins = 125 ± 102 mg/dL. Three patients had concomitantly *Mycobacterium tuberculosis* (*MTB*) detection in CSF (PCR-*MTB* or culture).

The most frequent association is with *MTB*.

Evolution of cases: 28 out of 67 died during the hospitalisation for C-ME, 10 of 26 women (38%) and 18 of 41 men (43%), with no statistical difference. There was no correlation between the good outcome and age. The survivors had a higher value of CD4 count (66/mm^3^ vs. 28/mm^3^), a lower VL (239.000 c/mL vs. 539.000 c/mL) with statistical correlation. The CSF analyses showed no correlation between the values of glucose, protein or lactic acid and survival).

**Conclusions:** Cryptococcus attacks young men but spares children, remains gentle with women, takes advantages of crowded areas, perhaps of drought weather, is friend with HIV and *MTB* (=mortal association, especially when there were no CD4-lymphocites around!).

## P276 Hidden in the Crypts: A Difficult Diagnosis of Disseminated Cryptococcal Infection

**Maaike Schalkwijk ^1^, Bela Kubat ^2^ and Astrid Oude Lashof ^3^**

^1^ Department of Internal medicine, Maastricht University Medical Center
^2^ Department of Pathology, Maastricht University Medical Center
^3^ Department of Medical Microbiology, Maastricht University Medical Center

**Background:** With an average of 7.7 cases per year, a *Cryptococcus neoformans* infection is quite rare in the Netherlands. The majority of patients is immunocompromised, HIV/AIDS is the most common condition. We present a case of complicated cryptococcocal disease in a patient that is immunocompromised due to prolonged use of corticosteroids.

Case description: The patient is a 60-year-old male who was diagnosed with *C. neoformans* meningitis 6 weeks prior to admission in our hospital. His relevant medical history included ischemic cardiomyopathy, ICD implantation for ventricular tachycardia (VT) and amiodarone-induced hyperthyroidism that required corticosteroid use. Initial treatment included flucytosine in combination with liposomal amphotericin B for four weeks and phasing out corticosteroids. The patient showed significant clinical improvement, therefore consolidation therapy with isavuconazole was started as fluconazole was contra-indicated with patients comorbidities, in particular ventricular tachycardia and prolonged QT-interval. After two weeks of consolidation therapy, the patient had a relapse in fever and elevated inflammatory markers, but no neurological symptoms. He was referred to our infectious disease department. A lumbar puncture was performed, opening pressure was normal at 12 cm H_2_O and showed mild pleicoytosis (12 × 10^6^/L). Cryptococcal antigen testing was negative in both serum and cerebrospinal fluid (CSF). Blood and CSF cultures remained negative. Isavuconazole was continued and empirical piperacillin-tazobactam was started awaiting further investigation. A PET-CT scan (Figure 1) showed a large pulmonary infiltrate in the upper left lobe, hilar and mediastinal lymphadenopathy. Broncho-alveolar lavage and endoscopic biopsy produced no causative micro-organism. A pulmonary biopsy was scheduled, but unfortunately the patients cardiac condition deteriorated as he suffered multiple episodes of VT. The recurring VT eventually led to cardiac arrest. Autopsy was performed and microscopic investigation showed extensive abscessing pulmonary infection with dense granulomatous infiltration in the upper left lobe. Grocott methamine silver stain (Figure 2) showed an extensive amount of encapsulated yeasts. Hilar and mediastinal lymph nodes showed the same abnormalities. Although cultures remained negative, disseminated cryptococcal disease is the probable cause of death, in relation with the patients known cardiac comorbidities. Results of polymerase chain reaction on the pulmonary tissue for confirmation are pending.

Discussion: We considered a disseminated cryptococcal infection, but were not able to confirm the diagnosis as a pulmonary biopsy was impossible with this cardiac instable patient. Immune Reconstitution Inflammatory Syndrome after corticosteroid tapering was thought to be unlikely since the patient showed no neurological deterioration, had normal opening pressure and only mild pleiocytosis on lumbar puncture. All investigations performed while the patient was alive could not confirm the pulmonary cryptococcosis and showed no sign of treatment failure for meningitis. Autopsy of the brain was not granted, so this remains a question to date.Our patient was treated in accordance with local guidelines for *C. neoformans* meningitis. According to those guidelines the management of pulmonary cryptococcosis is the same, however prospective studies that specifically address the management of pulmonary cryptococcosis are lacking.

**Conclusions:** Difficult to diagnose disseminated cryptococcal infection.

## P277 Discontinuation Considerations of Antifungal Treatment for an Invasive Mold Infection

**Romain Roth ^1^, Stavroula Masouridi-Levrat ^2^, Frederica Gianotti ^2^, Anne-Claire Mamez ^2^, Veronique Erard ^3^, Emmanouil Glambedakis ^4^, Frederic Lamoth ^4^, Pierre-Yves Bochud ^4^, Stephane Emonet ^5^, Arnaud Riat ^6^, Adrien Fisher ^6^, Christian Van Delden ^1^, Laurent Kaiser ^1^, Yves Chalandon ^2^ and Dionysios Neofytos ^1^**

^1^ Division of Infectious Diseases, University Hospital of Geneva, Geneva, Switzerland
^2^ Division of Hematology, Bone Marrow Transplant Unit, University Hospital of Geneva and faculty of Medicine, University of Geneva, Geneva, Switzerland
^3^ Division of Infectious Diseases, Cantonal Hospital of Fribourg, Fribourg, Switzerland
^4^ Division of Infectious Diseases, University Hospital of Lausanne, Lausanne, Switzerland
^5^ Division of Infectious Diseases, Cantonal Hospital of Sion, Sion, Switzerland
^6^ Laboratory of Bacteriology, University Hospital of Geneva, Geneva, Switzerland

**Objectives:** There are limited data to describe end-of-treatment (EOT) parameters of antifungal therapy for invasive mold infections (IMI).

**Methods:** We performed a 10-year cohort study of all adult (≥18-year-old) allogeneic hematopoietic cell transplant recipients with proven/probable IMI to describe IMI treatment duration and patient profile at treatment discontinuation. Only patients who had received a minimum of 84 days of antifungal treatment and were alive on day 84 post IMI diagnosis were included.

**Results:** Eighteen of 61 (29.5%) and 2/61 (3.3%) patients with IMI had died and discontinued treatment before day 84 post-IMI diagnosis, respectively. The remaining 41 (67.2%) patients were included for further analyses, with 14 and 29 proven and probable IMI, respectively. There were 30 (73.2%) and 11 (26.8%) patients with invasive aspergillosis (IA) and non-IA IMI (6-mucormmycosis, 5-other), respectively. Mean treatment duration was 360 days (range: 87, 2016, median: 246, IQR: 167–355): 340 and 415 days for IA and non-IA IMI, respectively (*p* = 0.55). By EOT, 14 (51.8%) and 13 (18.2) patients had complete/partial response and stable/deterioration of radiographic imaging, respectively. Twenty-eight (68.3%) patients were receiving steroids by EOT (18/28, 64.3% at prednisone dose equivalent >20 mg/day). Thirty-two (78%) patients were receiving immunosuppressive therapy with at least one of the following agents: tacrolimus (17/41, 41.5%), cyclosporine (15/41, 36.6%), mycophenolate mofetil (14/41, 34.2%), methotrexate (1/41, 2.4%). Laboratory values at EOT are shown in Table 1. Considering the mean treatment duration of 360 days, we divided patients in two groups: those who had died and those who were still alive by 365 days post-IMI diagnosis. The latter group would represent a patient group on which treatment discontinuation would be less likely due to death, hence variables leading to the clinical decision for treatment discontinuation could be more accurately described. Patients who were alive by 1-year post-IMI diagnosis were more likely to have higher lymphocyte, platelet and immunoglobulin counts and less hepatotoxicity, compared to patients who had died during the first year of IMI diagnosis (Table 1). There was a trend for patients still alive by 1-year post-IMI diagnosis to have discontinued steroid treatment (10/23, 43.5%), compared to patients who had died during the first year of IMI diagnosis (3/18, 16.7%; *p* = 0.07).

**Conclusions:** In this real-life cohort study, antifungal treatment for IMI was long and discontinued.

## P278 Challenges in Invasive Fungal Infections in Patients with Acute Leukemia

**Mariana Miranda, Ana Luísa Pinto, Rui Bergantim and Fernanda Trigo**

Centro Hospitalar Tondela-Viseu

**Introduction:** Invasive fungal infections (IFI) are important complications of hematological malignancies and its treatment. Despite prophylactic measures it remains a major cause of morbidity and mortality in this population.

Case 1. A 58-year-old woman recently diagnosed with acute myeloid leukemia (AML) started induction therapy with cytarabine and daunorubicin, as well as IFI prophylaxis with posaconazole. The patient had a fever since presentation and at day twenty after beginning of induction she presented severe headache localized to the right frontal and temporal lobes and right orbit, associated with photophobia, diplopia, reduced visual acuity, papilledema, ptosis and third and fourth cranial nerve palsies. CT-scan showed pansinusitis and MRI showed infiltration of the right cavernous sinus and sphenoid sinus and a massive pseudoaneurysm suggesting IFI. Empiric treatment with liposomal amphotericin B was initiated and she underwent a maxillectomy, right ethmoidectomy and bilateral sphenoidotomy. Biopsy of these structures confirmed the presence of *Aspergillus hyphae*. Despite reduction of symptoms and antifungal therapy there was almost no improvement of lesions and the patient eventually died.

Case 2. A 42-year-old man with previous history of non-specified pulmonary infection, presented productive cough, respiratory distress, fever, night sweats and pleural effusion. CT-scan showed moderate right pleural effusion and a heterogeneous consolidation process with hypodense areas, foci of cavitation, and air bronchogram. Lung biopsy showed pulmonary parenchyma involved by a necrotizing inflammatory process, in the presence of fungal structures (suspected aspergillosis). Patient started voriconazole but PCR for *Aspergillus* and *Mucorales* was negative in the biopsy sample. Further molecular studies were performed and a *Clasdosporium sphaerospermum* was identified. One month later, the patient was diagnosed with a B-cell acute lymphoid leukemia BCR-ABL+, with central nervous system involvement, and chemotherapy was started. During chemotherapy, voriconazole was changed to liposomal amphotericin B due to hepatic toxicity. Later on, it was possible to restart voriconazole. Despite all this, there was only mild improvement of pulmonary lesions in imagiological evaluations.

Case 3. A 66-year-old woman admitted with acute myeloid leukemia started induction therapy with cytarabine and daunorubicin and IFI prophylaxis with posaconazole. One month later she developed febrile neutropenia. CT-scan showed several nodular opacities of ill-defined limits, predominantly in the lower pulmonary lobes, and bilateral ground glass nodules. She started empiric cefepime and voriconazole. Antibacterial regimen was later changed to meropenem and amikacin due to persistent fever. PCR on bronchoalveolar lavage was positive for *Pneumocystis jirovecii*. Trimethoprim/sulfamethoxazole was initiated, but fever and respiratory symptoms persisted leading to the suspicion of an emergent IFI. Patient started liposomal amphotericin B with improvement of symptoms, defervescence and imagiological regression of the lesions. Nonetheless, no agent was identified.

**Conclusions:** These clinical cases illustrate how invasive fungal infections are very heterogenous and difficult to approach with unpredictable outcomes in hematological patients. Establishing a definite diagnosis of IFI is particularly challenging in immunocompromised patients, but delayed initiation of appropriate antifungal therapy increases mortality. A strategy of empirical or preemptive introduction of antifungal therapy is very important in this population.

## P279 Post COVID-19 Endogenous Endophthalmitis due to *C. albicans*: A Rare Case Report

**Gladys Kusumowidagdo ^1^, Lukman Edwar ^2^, Ari Djatikusumo ^3^, Anggun R. Yudantha ^3^, Umar Mardianto ^4^, Anna Rozaliyani ^5^, Ikhwan Rinaldi ^6^ and Meilania Saraswati ^7^**

^1^ Department of Ophtalmology, Faculty of Medicine Universitas Indonesia, Cipto Mangunkusumo National General Hospital, Jakarta, Indonesia
^2^ Infection and Immunology Division, Department of Ophthalmology, Faculty of Medicine Universitas Indonesia, Cipto Mangunkusumo National General Hospital, Jakarta, Indonesia
^3^ Vitreoretina Division, Department of Ophtalmology, Faculty of Medicine Universitas Indonesia, Cipto Mangunkusumo National General Hospital, Jakarta, Indonesia
^4^ Refractive and Contact Lens Division, Department of Ophtalmology, Faculty of Medicine Universitas Indonesia, Cipto Mangunkusumo National General Hospital, Jakarta, Indonesia
^5^ Department of Parasitology, Faculty of Medicine Universitas Indonesia, Jakarta, Indonesia
^6^ Medical Hematology and Oncology Division, Department of Internal Medicine, Faculty of Medicine Universitas Indonesia, Cipto Mangunkusumo National General Hospital, Jakarta, Indonesia
^7^ Department of Anatomical Pathology, Faculty of Medicine Universitas Indonesia, Cipto Mangunkusumo National General Hospital, Jakarta, Indonesia

**Background and Objective:** Ocular involvement in COVID-19 is rare and often mild. Recent reports showed possible opportunistic fungal infection post COVID-19 infection with risk factors of intensive care treatment, steroid use, immunosuppression, and due to the virus itself. We would like to report a rare case of severe ocular candidosis post COVID-19 infection.

**Case Illustration:** Male, 48 years old suffered from visual loss on both eyes. Patient was with history of COVID-19, ICU stay and ventilator use, hypercoagulable state, and diabetes mellitus. Patient also had history of tocilizumab administration and consumed steroid. Best corrected visual acuity (BCVA) was 6/30 on right eye (RE) and 6/6 on left eye (LE). Examination revealed fibrovascular on the optic nerve of RE with multiple exudates in macula and perimacular region. Anterior segment evaluation of both eyes (BE) was within normal limit. Patient was lost to follow up and returned with rapid deterioration of BE. BCVA was light perception (LP) on RE and three meters finger counting on LE. Posterior examination revealed tractional retinal detachment (TRD) on RE along with white lesion with hemorrhaghes. LE showed string of pearls and snowball. Patient had chest lump with destructive properties shown on CT-scan. Culture of chest lump was positive for *C. albicans* and positive for galactomannan. Patient was diagnosed with ocular candidosis of BE and treated with intravenous voriconazole for 14 days. Vitrectomy and intravitreal voriconazole was performed on BE. Final BCVA was hand movement good projection on RE and two meters finger counting on LE.

**Discussion and Conclusions:** Increasing incidence of COVID-19 infection warrants awareness of ocular involvement in high risk patients; patients with ICU stay, tocilizumab steroid use, systemic diseases.

## P280 Positivity Rate of Aspergillus-Specific Antibody among Non-Small Cell Lung Cancer (NSCLC) Patients in Indonesia

**Findra Setianingrum ^1,2^, Asiyah Taqiyya Fakhrur Razi ^1^, Jamal Zaini ^3,4^, Abul A’la Al Maududi ^3,4^, Mulyati Tugiran ^1,2^, Robiatul Adawiyah ^1,2^, Ridhawati Sjam ^1,2^ and Anna Rozaliyani ^1,2^**

^1^ Department of Parasitology, Faculty of Medicine, Universitas Indonesia, Indonesia
^2^ Pulmonary Mycosis Centre, Indonesia
^3^ Department of Pulmonology and Respiratory Medicine, Faculty of Medicine, Universitas Indonesia
^4^ Persahabatan National Respiratory Referral Hospital, Indonesia

**Objectives:** Lung cancer patients are more susceptible to fungal infections, including aspergillosis. Detection of *Aspergillus*-specific IgG with immunochromatography (ICT) has been used in recent years and showed reliable performances to diagnose chronic pulmonary aspergillosis (CPA). This study aim to determine the positivity rate of *Aspergillus*-specific antibody among non-small cell lung cancer (NSCLC) patients at Persahabatan National Respiratory Referral Hospital, Indonesia.

**Material andMethods:** We tested serum aliquots for 77 NSCLC patients with ICT LDBio to detect *Aspergillus*-specific antibody. The serology tests were carried out at the laboratory of Parasitology Department, Faculty od Medicine Universitas Indonesia.

**Results:** Seventeen (22%) patients had positive results of *Aspergillus*-specific antibody. Fifty eight (75%) patients had adenocarcinoma and 19 (25%) patients had squamous cell carcinoma. The most common radiological findings was a pulmonary mass (*n* = 33, 43%) followed by consolidations (*n* = 24, 31%), nodules (*n* = 11, 14%) and infiltrates (*n* = 10, 13%). There is no different in age and type of lung cancer between positive and negative *Aspergillus*-specific antibody group. Patients with positive *Aspergillus*-specific antibody had higher proportion of advanced stage of lung cancer (88% in positive *Aspergillus*-specific antibody group; 77% in negative *Aspergillus*-specific antibody group; *p* = 0.499). However, this difference was not statistically significant. History of previous tuberculosis was found in 10 (17%) patients from negative *Aspergillus*-specific antibody group.

**Conclusions:** These results indicated that *Aspergillus* infection may exist in NSCLC patients and further studies including clinical profiles and fungal culture to identify the prevalence of aspergillosis in NSCLC patients.

## P281 Secondary Invasive Fungal Infection in Hospitalized Patients with COVID-19 in the United States

**George R. Thompson ^1^, Jeanette Jiang ^2^, Emily Shortridge ^2^, Kalatu Davies ^3^, Giridharan Gurumoorthy ^3^, Tomomi Kimura ^3^**

^1^ UC Davis Health
^2^ Astellas Pharma Global Development, Inc.
^3^ Astellas Pharma US, Inc.

**Objective:** Invasive fungal infections (IFI) can be a cause of morbidity and mortality in COVID-19 patients, especially those in intensive care (ICU) and/or with mechanical ventilation (MV) support. To date, most studies examining IFI in COVID-19 cases are from outside the US and/or in a single hospital. The objective of this study was to describe the impact of IFI in COVID-19 on patient outcomes and resource utilization in the US.

**Materials & Methods:** Using a nationwide hospital database capturing about 25% of annual US inpatients, patients hospitalized with COVID-19 and discharged or deceased from April to September 2020 were identified. IFI was defined by diagnosis codes or microbiology findings from sterile sites, plus systemic antifungal use. Patients without IFI at admission were followed until discharge or death and the incidence of IFI was estimated. Further, IFI patients were matched to non-IFI patients with time-dependent propensity score to estimate excess mortality, excess days to discharge, and excess costs attributable to IFI.

**Results:** Of 172,170 hospitalized COVID-19 patients (median 63 years, 51.4% male), 758 developed IFI (0.44%, 0.51/1000 patient-days). Higher incidence was observed in patients admitted to the ICU (653/51,931 [1.26%], 1.03/1000 patient-days). Major pathogens were candidiasis and aspergillosis. Medications for COVID included remdesivir (13.5%), tocilizumab (6.7%), and corticosteroids (56.1%). Overall mortality was 14.4% (IFI: 59.8%, non-IFI: 14.2%) and median length of stay was 6 days (IFI: 31 days, non-IFI 6 days). Notable baseline conditions in patients developed IFI included: complicated diabetes (47.2%), aplastic anemia (21.6%), and chronic respiratory airway abnormalities (14.2%). MV use was strongly associated with IFI (Hazard ratio [95% CL] = 5.29 [4.10, 6.83]). In the matched group, excess mortality, median excess days to discharge, and median excess costs were estimated as 17.9% [15.2, 20.7], 3 days [−7, 15], 22,000 USD [18,010, 25,630], respectively.

**Conclusions:** Incidence was low, but IFI attributable clinical and economic burden was significant. IFI and COVID-19 manifest similarly so IFI burden may be underestimated. Greater awareness of IFI in COVID-19 patients is critical to minimize delays in treatment.

## P290 Invasive Fungal Infections in a Pediatric Hematology-Oncology Department: A 16-Year Retrospective Study

**Nikoleta Kazakou ^1^, Timoleon-Achilleas Vyzantiadis ^2^, Anastasia Gambeta ^1^, Eleni Vasileiou ^1^, Eleni Tsotridou ^1^, Maria Palabougiouki ^1^, Maria Ioannidou ^1^, Emmanuel Hatzipantelis ^1^ and Athanasios Tragiannidis ^1^**

^1^ Faculty OF Health Sciences, Aristotle University of Thessaloniki
^2^ First Department of Microbiology, School of Medicine, Aristotle University of Thessaloniki, Greece

**Objectives:** Invasive fungal diseases (IFDs) are a major cause of morbidity and mortality in immunocompromised children. The purpose of our study was to evaluate the incidence of IFDs in pediatric patients with underlying hematologic malignancies (acute leukemias, lymphomas) and determine the patient characteristics, predisposing factors, diagnosis, treatment, efficacy, and outcome of IFDs.

**Methods and Materials:** For the purpose of the study, a retrospective analysis was performed on cases with proven and probable fungal infections from January 2001 to December 2016 (16 years). Results: During this period, 297 children with hematologic malignancies were admitted to the 2nd Pediatric Department of Aristotle University of Thessaloniki, Greece, and 24 cases of IFIs were registered. The most common underlying diseases were acute lymphoblastic leukemia (ALL; *n* = 19, 79%), followed by acute myeloid leukemia (AML; *n* = 4, 17%) and non-Hodgkin lymphoma (NHL; *n* = 1, 4%). The crude incidence rates of IFDs in ALL, AML, and NHL were 10.5%, 18.2%, and 2.8% respectively. Based on the results, 25% (*n* = 6) and 75% (*n* = 18) of the patients were diagnosed as proven and probable IFI cases, respectively. The lung was the most common site of involvement in 16 (66.7%) cases. Furthermore, Aspergillus and Candida species represented 58.3% and 29.1% of the identified species, respectively. Regarding antifungal treatment, liposomal amphotericin B was the most commonly prescribed therapeutic agent (*n* = 21), followed by voriconazole (*n* = 9), caspofungin (*n* = 3), posaconazole (*n* = 3), micafungin (*n* = 1), and fluconazole (*n* = 1). In addition, 12 children received combined antifungal treatment. Two patients with mucormycosis were successfully treated with a combination of surgery and prolonged antifungal treatment with L-AmB and posaconazole. The crude mortality rate was obtained as 33.3%. Conclusions: As the findings of the present study indicated, despite the progress in the diagnosis and treatment of IFDs with the use of new antifungal agents, the mortality rate of these infections still remains high.

## P291 Malassezia Furfur as a Causative Agent of Invasive Mycoses in Neonates

**Tatiana Bogomolova ^1,2^, Tatiyana Bogdanova ^2^, Andrei Alekseev ^2^, Natalia Vasilyeva ^1,2^, Igor Riabinin ^1,2^, Elena Shagdileeva ^3^ and Olga Kozlova ^3^**

^1^ Kashkin Research Institute of Medical Mycology, North-Western State Medical University n.a. I. I. Mechnikov
^2^ Department of Medical Microbiology, North-Western State Medical University n.a. I. I. Mechnikov
^3^ Department of Clinical Mycology, Allergy and Immunology, North-Western State Medical University n.a. I. I. Mechnikov

**Objectives:** The role of *Malassezia furfur* as a causative agent of invasive mycoses is largely underestimated. The aim of the study: to evaluate prevalence of colonization and infection caused by *M. furfur* in neonates hospitalized in neonatal intensive care units (NICUs) of two children’s hospitals in St. Petersburg, Russia.

**Materials & Methods:** The half-year study (Sep 2020 to Apr 2021) included neonates with suspected invasive mycoses and at least one of risk factors such as prolonged hospitalization, lipid parenteral nutrition, congenital immunodeficiency disorder, prematurity, low or very low birth weight and implanted intravascular and/or urinary catheters. Biomaterials from patients (blood, cerebrospinal fluid (CSF), sputum, bronchoalveolar lavage (BAL), urine, feces) were cultivated on modified Leeming-Notman medium (agar and broth), meat peptone agar and Sabouraud dextrose agar and broth at 35 °C for 2–14 days. Blood and CSF samples were incubated in BactAlert (BioMerieux) without/with lipids. Identification of yeast and bacterial cultures was based on morphological characteristics and confirmed by MALDI TOF/TOF tandem-mass spectrometry (MS) (Autoflex Speed, Bruker). Susceptibility testing was conducted by Sensititre Yeast One test panels (Thermo Fisher Scientific, UK) supplemented with 0.05% v/v Tween 80 and 1% v/v glycerol.

**Results:** A total of 106 samples from 57 neonates were examined. Positive cultures (yeasts and bacteria) were found in 38 neonates (66.7%), including 17 patients with *M. furfur* isolation. Ten neonates had confirmed bloodstream infection (BSI). The majority of BSIs were caused by bacteria—6 cases: *Staphylococcus* spp.—4 (*St. epidermidis*—2, *St. capitis*—1, *St.* sp.—1); *Klebsiella pneumonia* + *Escherichia coli*—1; * Dermacoccus nishinomiyaensis*—1. Fungal BSIs were found in 4 cases: 2—*Rhodotorula mucilaginosa*, 2—*M. furfur*. Five neonates had possible invasive lung fungal infection caused by *M. furfur*: in 4 cases *M. furfur* was isolated from sputum, in 1 case—from BAL. These patients presented with clinical features of pulmonary infection, fungal load in sputum/BAL reached 10^8^ CFU/mL (Figure 1) and no other etiologic agents were found. *M. furfur* colonization of gastrointestinal and/or urinary tract was detected in 6 (10.5%) of neonates, respiratory tract—in 5 neonates (8.7%). Antifungal susceptibility profiles are shown in Table 1.

**Conclusions:** Prevalence of *M. furfur* in biomaterials of neonates hospitalized in NICUs was 30.9%. Proven (BSIs) and possible (pulmonary) cases of invasive malasseziosis were revealed in 12.3% of patients.

## P292 Invasive Aspergillosis in Children in St. Petersburg, Russia

**Elena Shagdileeva ^1^, Olga Shadrivova ^1^, Sofia Khostelidi ^1^, Yulia Dinikina ^2^ Alice Volkova ^3^, Marina Popova ^3^, Ludmila Zubarovskaya ^3^, Alexey Kolbin ^3,4^, Tatyana Bogomolova ^1^, Svetlana Ignatyeva ^1^, Margaret Belogurova ^2,5^, Elmira Boichenko ^6^ and Nikolay Klimko ^1^**

^1^ North-West State Medical University Named after I. I. Mechnikov
^2^ V. Almazov National Medical Research Center
^3^ I. P. Pavlov First St. Petersburg State Medical University
^4^ St. Petersburg State University
^5^ St. Petersburg Clinical Scientific and Practical Oncological Center
^6^ Children’s City Multidisciplinary Clinical Specialized Center of High Medical Technologies

**Objectives:** To analyze the demographic parameters, risk factors, etiology, and results of treatment of IA in children in St. Petersburg, Russia.

**Materials & Methods:** The retropective analysis of the registry (1998–2021) data. Diagnosis of IA was made according to EORTC/MSGERC criteria (2019).

**Results:** In the study were included 132 children with “proven” (12%) and “probable” (88%) IA according to the EORTC/MSG 2019 criteria. The median age was 11.5 y (1–17), girls—53%.

The underlying conditions were oncohematologycal diseases—83% (ALL—45%, AML—32%, aplastic anemia—6%, myelodysplastic syndrome—5%, Hodgkin lymphoma—3%, non-Hodgkin’s lymphoma—2%, other—4%), solid tumor—8%, other—9%. The main risk factors were glucocorticosteroid therapy—58%, severe neutropenia—53% (median—18.5 days), lymphocytopenia—52% (median—14 days), immunosuppression—40%, allo-HSCT—37%, GVHD—33%, primary immunodeficiency—2%.

Galactomannan test in bronchoalveolar lavage/blood serum/cerebrospinal fluid was positive in 73% patients. Microscopy revealed septate mycelium in 11% patients, culture was positive (*A. fumigatus*—42%, *A. niger*—20%, *A. flavus*—20%, *A. oharaceus*—3%, *A. nidulans*—6%, *A. terreus*—3%, *A. ustus*—3%, *Aspergilus* spp.—3%) in 26% cases. Two pathogens of *Aspergillus* spp. were detected in 2% of patients, mixed-mycoses were detected in 2% of children. Histological confirmation received in 12% childrens.

The sites of IA were lungs (89%), sinuses (8%), CNS (7%), soft tissue (3%), eyes (2%), liver (2%), heart (2%), bones (2%), spleen (1%), ≥2 organ involvement—13%. The clinical manifestations were fever (71%), cough (55%) and respiratory failure (35%), and hemoptysis (5%). CT scan signs of pulmonary IA were infitrates (59%), bilateral lesion (43%), “halo” sign (7%) and “sickle” sign (7%).

Antifungal treatment (voriconazole—76%, amphotericin B deoxycholate—24%, caspofungin—18%, itraconazole—12%, lipid amphotericin B—7%, liposomal amphotericin B—6%, posaconazole—8%) was used in 98% patients, surgery—4%. The median duration of antifungal treatment was 76 days (3–370). The overall survival of patients in 12-weeks was 70%.

**Conclusions:** Invasive aspergillosis developed more often in children with oncohematological diseases (83%). The main risk factors were glucocorticosteroid therapy (58%), prolonged neutropenia (53%) and lymphocytopenia (52%) immunosuppression—40%, and allo-HSCT—37% with GVHD—33%. The main sites of invasive aspergillosis were lungs (89%), ≥2 organ involvement—13%. Antifungal treatment was used in 98% patients, voriconazole—76%. The overall survival of patients in 12-weeks was 70%.

## P293 Double Trouble: Tuberculous and Fungal Osteomyelitis in An Immunompetent Adolescent Girl

**Salima Rattani, Sonia Qureshi, Joveria Farooqi, Sadaf Zaka and Kauser Jabeen**

Agakhan University Hospital

**Introduction:** Skeletal tuberculosis (TB) commonly effects a single site in 90–95% of reported cases. Fungal osteomyelitis is a rare disease occurring in immunocompromised patients affecting a single large joint. Here we report a case of TB osteomyelitis in an immunocompetent young girl with superimposed fungal infection.

**Case Report:** A 9 year old girl admitted with complains of right thigh pain after a fall from bed. Radiographs showed fracture of the right femoral shaft for which she underwent open reduction and internal fixation (ORIF) of right femur and was discharged on oral cephalexin and non-weight bearing exercises. A month later she developed right knee pain and swelling along with fever. Synovial fluid detailed report showed protein of 5500 mg/dL, glucose of 22 mg/dL and total leucocyte count of >10,000 × 10 ^3^/µL with 85% polymorphonuclear cells and 15% lymphocytes representing septic arthritis. Acute inflammatory markers were raised (ESR; 112 mm/1st hour and CRP; 193.4 mg/L) respectively. Bacterial culture and Methicillin Resistant Staphylococcus Aureus (MRSA) Xpert^®^ from the synovial fluid were negative. She underwent a right knee arthrotomy for washout, debridement and placement of antibiotic (vancomycin) beads and tissue was sent for bacterial, mycobacterial and fungal cultures and antimicrobial susceptibility testing. Xpert^®^ MTB/RIF assay detected *Mycobacterium tuberculosis* (MTB) with indeterminate rifampicin resistance and the patient was started on four drugs anti-tuberculous therapy (ATT). Two days later tissue culture grew mold that was later identified as *Aspergillus terreus*. Patient was started on intravenous amphotericin and later switched to oral voriconazole after mold identification. Later TB culture showed growth of MTB susceptible to first line ATT. A second knee debridement was done along with removal of foreign material (antibiotic beads) after 2 weeks. Tissue culture sent revealed growth of *Aspergillus terreus* again. Patient is now in regular follow-up and has shown improvement in clinical, laboratory and radiological parameters. Antifungal and antibiotics have been discontinued after 6 weeks of therapy with continuation of ATT.

**Conclusions:** Despite its rarity, osteomyelitis due to fungal and tuberculous co-infection should be managed by a combination of surgical and medical interventions. Culture and histopathological examination from biopsy or intraoperative samples are indispensable to ensure optimum outcomes when managing these cases. Drug interactions could be a problem with ATT and triazoles for which close monitoring of liver function is a must.

## P294 Differences in Pediatric Candidemia Trends in Two Hospitals: Boston (US) and Valencia (Spain)

**Anabel Piqueras ^1^, Lakshmi Ganapathi ^2^, Javier Peman ^1^ and Julia Köhler ^2^**

^1^ Hospital Universitario La Fe
^2^ Boston Children’s Hospital

**Objectives and Materials & Methods:** Epidemiology and outcome of candidemia differ significantly between children and adult patients and vary widely among different geographic areas. Some apparent differences might be attributable to heterogeneous study designs. Using the same study design, we compared changes in candidemia trends, microbiology and antifungal treatment in 62 children (68 episodes) with candidemia admitted to Hospital La Fe (HLFE) with ~200 pediatric beds in Valencia, Spain (2011–2018) and 182 children (208 episodes) at Boston Children’s Hospital with ~300 pediatric beds (BCH) in Boston, USA (2006–2016).



**Results: Demographic Characteristics and Outcome of Patients wih Candidemia**


**Hospital La Fe**

**BCH**


**NICU**

**Other**

**NICU**

**Other**
N patients (%)30 (48)3228 (15)154N episodes (%)33 (49)35 (51)29 (14)179 (86)Median age [IQR]13 d [10, 18]27 m [14, 59]32 d [19, 60]57 m [23, 155]Breakthrough candidemia (%)7 (21)7 (20)5 (17)31 (17)Deaths (%) ^1^8 (27)2 (6)7 (25)18 (11)
NICU Neonatal intensive care Unit, ^1^ At BCH, there were no deaths in children ≤ 4 years of age after 2011.


Among NICU patients, *Candida albicans* was the most frequent species at HLFE (53%), whereas at BCH *C. parapsilosis* predominated (48%). In both hospitals, liposomal amphotericin was used in neonatal candidemia. A decline in the annual incidence rate of candidemia was observed in both hospitals with highest incidence rates observed in younger patients. At BCH infants and children less than 1–4 years of age had the highest candidemia incidence rates and the downward trend in candidemia overall was driven by these younger patients. At HLFE the incidence rate in the NICU remained unchanged over time.

Overall, liposomal amphotericin was the antifungal agent most frequently prescribed empirically in both hospitals. Antifungal resistance rates were extremely low in our study, with no signs of resistance emergence over time. All isolates were susceptible to prior prophylactic antifungals as well as to the empiric antifungal agents chosen.

At HLFE, candidemia-related death predominated in NICU infants with a median gestational age of 24 weeks [IQR 23, 26], with *C. albicans* predominating (62%). Mortality in NICU infants did not change at HLFE over the study period. All neonatal deaths attributed to candidemia at BCH occurred prior to 2012. Among patients who expired at BCH, 11 were immunocompromised, 7 were immunocompetent and 7 were neonates.

**Conclusions:** Wide differences in patient populations, approaches to management of underlying disease, use of broad-spectrum antibiotics, antifungal prophylaxis and infection control measures may contribute to the heterogeneity of candidemia among institutions. The underlying disease, success in source control and timeliness of antifungal therapy are apparently associated with mortality. The two very different pediatric institutions examined here share an absence of antifungal resistance emergence and prevalent use of amphotericin. New antifungal agents with lower toxicity, that induce fitness defects in resistant *Candida* strains as amphotericin does, hence might improve resistance prevalence in adult patients. Improving candidemia outcomes may require development of algorithms for empiric therapy in susceptibility populations as well as antibiotic stewardship and improved management of underlying conditions.

## P295 Invasive Candidiasis (IC) Caused by *Candida albicans* and *Candida* Non-*albicans* in Neonatal Intensive Care Units (NICUs) in St. Petersburg

**Elena Shagdileeva ^1^, Olga Belova ^2^, Tatyana Kuznetsova ^2^, Sergei Voronovich ^2^, Georgy Rubin ^3^, Svetlana Vorobeva ^2^, Natalya Kotina ^2^, Tatyana Bogomolova ^1^, Irina Vybornova ^1^, Alexey Kolbin ^4,5^ and Nikolay Klimko ^1^**

^1^ North-West State Medical University Named after I. I. Mechnikov
^2^ Children’s City Multidisciplinary Clinical Specialized Center of High Medical Technologies
^3^ City Children’s Hospital № 17
^4^ I. P. Pavlov First St. Petersburg State Medical University
^5^ St. Petersburg State University

**Objectives:** IC is a serious complication in newborns. We investigated etiology, risk factors, clinical features and results of treatment of IC caused by *C. albicans* and *Candida* non-*albicans*.

**Materials & Methods:** The prospective study in January 2015 to June 2020. Diagnosis of IC was made according to EORTC/MSG criteria (2019).

**Results:** In the prospective study were included 32 neonates with “proven” IC. 15 children were with IC caused by *C. albicans* and 17 were with IC caused by *Candida* non-*albicans*, boys—53% vs. 35%. Premature neonates accounted for 100% vs. 76% patients. Median birth weight was—1200 g (470–2920) vs. 1680 g (560–4000), median gestational age at birth—28 weeks (23–35) vs. 30 weeks (23–40).

Antibacterial drugs were used in 100% patients, mechanical ventilation—100% vs. 71%, *p* = 0.026, central venous catheter (CVC)—100% vs. 94%, parenteral nutrition—93% vs. 53%, *p* = 0.017. The clinical variant of IC was candidemia (100% vs. 94%), chorioretinitis (7% vs. 6%), meningitis (0% vs. 6%), hepatitis (7% vs. 0%). *C. albicans* was isolated in 47% cases of all patients. The pathogens of IC caused by *Candida* non-*albicans* were *C. parapsilosis* (47%), *C. famata* (24%), *C. tropicalis* (12%), *C. pelliculosa* (12%), *C.guiliermondii* (6%), and *Candida* spp. (12%). In vitro resistant to fluconazole were 7% vs. 6% pathogens, dose-dependent susceptible to fluconazole—0% vs. 6%. In the first 24 h after diagnosis, CVC was removed/replaced in 80% vs. 88% patients, antifungal treatment was used in 100% patients: fluconazole (100% vs. 100%), micafungin (33% vs. 41%), amphotericin B deoxycholate (20% vs. 23%), amphotericin B lipid complex (7 vs. 0%). The median duration of treatment was 25 vs. 20 days (1–54 vs. 1–39). The overall 30-day survival rate was 73% vs. 88%.

**Conclusions:** IC was detected mainly in premature neonates (100% vs. 76%) patients in NICUs. Main risk factors were antibioltics use 100%, mechanical ventilation—(100% vs. 71%, *p* = 0.026), CVC—100% vs. 94% and parenteral nutrition—93%. The pathogens of IC caused by *Candida* non-*albicans* were *C. parapsilosis* (47%), *C. famata* (24%). The main clinical presentations were candidemia (100% vs. 94%). The overall 30-day survival rate was 73% vs. 88%.

## P296 Phaeoacremonium Species Infection in a Knee of a Healthy 5-Year-Old Girl Identified Using ITS Sequencing

**Zeala Gazit, Hofit Lorenz, Shiraz Halevi ^1^, Shoshana Cohen, Or Krieger and Sharon Amit**

Sheba Medical Center

**Objectives:** To identify a fungus that was undetectable using traditional or MALDI-TOF methods.

**Materials & Methods:** A 5-year-old previously healthy girl was examined in the pediatric rheumatology clinic after 1 month of limping, swelling and pain in her left knee. She was discharged home with a short regimen of NSAIDS, and due to the prolongation of symptoms, received hydrocortisone knee injection with no clinical response. After further deterioration of knee swelling—she was hospitalized 5 months after symptoms onset. Upon arrival to the emergency room, synovial fluid was withdrawn and transferred to 2 sets of blood culture bottles and further arthroscopic synovectomy retrieved tissue specimen that was plated on growth media for microbiological identification. Wet mount with lactophenol fuchsin was used in microscopy to observe the fungus. MALDI-TOF was executed according to manufactures guidelines (Bruker) and ITS1–5.8S-ITS2–28S rDNA sequencing was run using primers that fully cover the ITS1/ITS2 region including part of 28S (603 bp long).

**Results:** Inoculation of the same batch of hydrocortisone injection returned sterile, ruling out steroid contamination. An identical fungus grew from both synovial fluid samples after 5 days of incubation but not from the tissue sample. Septate hyphae with slightly curved small conidia was observed under the microscope. MALDI-TOF failed to identify the fungus. ITS1–5.8S-ITS2–28S rDNA sequencing identified *Phaeoacremonium fuscum*. Synovectomy of the infected tissue and Voriconazole antifungal treatment resulted in complete infection recovery, although Voriconazole treatment was continued and rehabilitation treatment was initiated.

**Conclusions:** The genus *Phaeoacremonium* is associated with opportunistic human infections, as well as stunted growth and die-back of various woody hosts. Here we present a rare infection with *Phaeoacremonium fuscum* in a young girl that was identified solely by ITS1–5.8S- ITS2 rDNA sequencing, demonstrating the importance of this technique in clinical microbiology laboratories.

## P298 Infection Caused by *Malassezia furfur* Is Strong Risk Factor for BPD in Extremely Preterm Newborns with ELBW

**Oleg Ionov ^1,2^, Anna Kirtbaya ^1,2^, Ekaterina Balashova ^1^, Anastasiya Nikonets ^1,2^, Olga Goncharuk ^1^, Diiana Sharafutdinova ^1,2^, Ludmila Lubasovskaya ^1^, Irina Nikitina ^1^, Tatiana Priputnevich ^1^ and Victor Zubkov ^1^**

^1^ National Medical Research Center for Obstetrics, Gynecology and Perinatology Named after Academician V. I. Kulakov
^2^ Sechenov First Moscow State Medical University of the Ministry of Health of the Russian Federation (Sechenov University)

**Objectives:** To evaluate the factors that influence on the development of BPD in extremely preterm newborns with ELBW.

**Materials & Methods:** Retrospective, single-center study of preterm ELBW newborns admitted between 2015 and 2019 in the NICU named after A. G. Antonov of the National medical research center for obstetrics, gynecology and perinatology named after academician V. I. Kulakov. Detailed demographical and clinical data were collected daily up to discharge. A total of 132 infants were enrolled, 45 infants in group with BPD and 87 infants in the control group without BPD. Exclusion criterion: death of the child before 36 weeks of postmenstrual age (PMA).

**Results:** The presence of invasive fungal infections caused by *M. furfur* increased the risk of BPD (OR = 8.6, 95% CI (2.4–13.9), *p* < 0.001) and severe BPD (OR = 9.3, 95% CI (2.2–38.9), *p* = 0.003) in newborns with ELBW. The presence of cases of Malassezia bloodstream infection increased the risk of BPD (OR = 5.0, 95% CI (2.0–12.7), *p* < 0.001) and severe BPD (OR = 6.7, 95% CI (2.4–19.4), *p* = 0.0004) in newborns with ELBW. The frequency of BPD among preterm infants with gestational age (GA) less than 26 weeks was 49.2%, GA 27–31 weeks—22.9%. The frequency of BPD increased to 75% in preterm infants with the birth weight less than 700 g, comparing with children with the birth weight more than 700 g at birth (26.8%), *p* < 0.001. Treatment with antenatal corticosteroids was associated with a reduction in BPD to 28.6% (compared with no treatment 46.3%), *p* = 0.046. In the cases of need for mechanical ventilation the frequency of BPD increased to 49.3%, *p* < 0.001.

**Conclusions:** Infection caused by *Malassezia furfur* increased the risk of BPD in extremely preterm newborns with ELBW. Also BPD was associated with lack of treatment with antenatal corticosteroids, prematurity and GA less than 27 weeks, birth weight less than 700 g and the use of mechanical ventilation.

## P299 Bloodstream Candida Infections in a Neonatal Intensive Care Unit from Southern Brazil

**Leandre Willot ^1,2^, Jessica Benelli ^1,2^, Rossana Basso ^1,2^, Gabriel Klafke ^1^ and Melissa Orzechowski Xavier ^1,2^**

^1^ Universidade Federal Do Rio Grande
^2^ HU-FURG/EBSERH

**Objectives:** Candidemia in hospitalized patients, especially in those admitted to Neonatal Intensive Care Units (NICU), leads to high mortality rates, prolonged hospitalization periods, antifungal therapy, and consequently higher hospitalar costs. An appropriate knowledge of the local prevalence of this disease and the proportion of *Candida* spp. between the microbes causing blood infections in preterm infants are necessary for appropriate therapeutic and surveillance interventions. This study aimed to perform a retrospective study of neonatal sepsis focused in candidemia at a tertiary care hospital from Southern Brazil in a period of ten years.

**Methods & Materials:** The study was conducted at the Universitary Hospital of Rio Grande (HU-FURG/EBSERH), which is composed of 18 NICU beds. We evaluated the prevalence of candidemia, diagnosed trough yeast isolation in blood cultures (by automated blood culture system—Bactec FX^TM^) for detection of bloodstream infections between January 2011 and December 2020. Data regarding the total number of patients submitted to blood cultures, and the etiology of blood infections from NICU during the same period were collected.

**Results:** During the ten-year study a total of 2579 blood cultures from patients at the NICU were evaluated in the hospital lab. Septicemia was confirmed, by some pathogen growth, in 270 patients, corresponding to an average of 27 cases/year (ranging from 5 to 47 cases/year). During this period, fungemia by *Candida* species was responsible for 10% (*n* = 27) of the neonatal sepsis. However, this rate had a great oscillation between years, being candidemia responsible for 2.3% of the neonatal sepsis in the year with the less prevalence (2020), as up to 23.2% in 2017. In addition, *Candida* yeast was the third main microbe related to blood infection at the NICU during the period studied (*n* = 27), just behind *Staphylococcus epidermidis* (*n* = 66) and *Klebsiella pneumoniae* (*n* = 52). Unfortunately, given that our hospital does not routinely perform exams to *Candida* specie identification, our results are shown just regarding genus.

**Conclusions:** *Candida* species are important pathogens associated with neonatal sepsis in our hospital. Surveillance studies of the local prevalence of candidemia in NICU are extremely important to identify its importance and impact on the preterm infants. These data increases the aware regarding the necessity of an early diagnosis and treatment which are associated with a better prognosis.

## P300 Invasive Fusariosis Caused by *Fusarium verticillioides* in a Pediatric Patient with Leukemia Admitted to a Public Children’s Hospital in Brazil

**Regina Ramos ^1^, Luciana Ruiz ^2^, Bruna Lara ^2^, Mário Bonci ^1^, Diniz Júnior ^3^, Debora Moreira ^4^, Rennan Santos ^1^, Marcelo Otsuka ^5^, Rinaldo Gandra ^6^, Paula Trezza ^7^, Maria José Silveira ^7^ and Claudete Paula ^1^**

^1^ School of Dentistry, University of São Paulo (USP)
^2^ Adolfo Lutz Institute (IAL)
^3^ Medical School—Federal University of Mato Grosso (UFMT)
^4^ Medical School, University of São Paulo (USP)
^5^ Darcy Vargas Children Hospital
^6^ University of Western Paraná State—Campus of Cascavel
^7^ CONTROLBIO Microbiological Technical Assistance SS LTDA

**Objectives:** The aim of this study was to report one case of nosocomial infection caused by *Fusarium verticillioides* in children with cancer hospitalized in a public children’s hospital located in Brazil.

**Materials & Methods:** The reported fusariosis case occurred during the year of 2019, and the pediatric patient were admitted to the Oncology wards of the Infantil Darcy Vargas Hospital located in the city of São Paulo, SP, Brazil. Fungal strain was isolated from blood from this patient. After culture isolation, the strain was identified based on their macroscopic and microscopic characteristics and molecularly studied for species confirmation through amplification and sequencing of TEF region with primers EF1 and EF2. Antifungal susceptibility test of fungal isolate was performed using Etest. The following antifungal drugs were tested: amphotericin B, itraconazole, voriconazole, fluconazole and caspofungin.

**Results:** The patient was male, 11-years-old, had febrile neutropenia and, as the underlying disease, had acute lymphocytic leucemia. Blood was collected for cultures, which showed growth of white fungal mycelium. Phenotypic study of strain isolated from blood revealed microscopic and macroscopic characteristics typical of the *Fusarium* genus. Using molecular analysis, the samples were identified as *F. verticillioides*. Susceptibility test in vitro was performed and high values of minimum inhibitory concentration (MIC) were observed for fluconazole, itraconazole and caspofungin, whereas lower values were detected for amphotericin B and voriconazole. MIC values were not interpreted since there are still no clinical breakpoints formally proposed for fungi of the *Fusarium* genus.

**Conclusions:** This case highlights fusariosis as a potential severe health threat, especially in the context of immunosuppression. Considering the emergence of filamentous fungi as etiological agents of hospital infections, it is important to identify the agent causing the infection, to understand the types of clinical manifestations that may occur, the environments in which these organisms survive, and thus to better plan patient care.

## P305 Invasive Aspergillosis in Adult COVID-19 Patients

**Olga Shadrivova ^1^, Kseniya Panchishina ^1^, Olga Kozlova ^1^, Ekaterina Desyatik ^1^, Elena Shagdileeva ^1^, Sofya Khostelidi ^1^, Vitaly Gusarov ^2^, Mikhail Zamyatin ^2^, Nikolay Lovtsevich ^2^, Vasiliy Belash ^3^, Aminat Gasanova ^3^, Vladimir Lee ^4^, Yulia Borzova ^1^ and Nikolay Klimko ^1^**

^1^ North-Western State Medical University Named after I. I. Mechnikov
^2^ National Medical and Surgical Center Named after N. I. Pirogov of the Russian Ministry of Health
^3^ I. Pavlov First Saint Petersburg State Medical University
^4^ Saint Petersburg State Research Institute of Phthisiopulmonology, Ministry of Healthcare of the Russian Federation

**Objectives:** To study demographic parameters, risk factors, etiology, clinical symptoms and the effectiveness of therapy of COVID-19 associated invasive pulmonary aspergillosis (CAPA).

**Materials & Methods:** Retrospective analysis of CAPA cases in adult patients. For the diagnosis of CAPA, the ECMM/ISHAM 2020 criteria were used.

**Results:** The study included 27 CAPA patients, median age—62 (34–82) years, men—78%. The underlying diseases were decompensated diabetes mellitus in 33% patients, non-Hodgkin’s lymphoma—19%, other oncohematological diseases—8%, COPD—11%, malignant neoplasms—7%.

The main risk factors for the IA development were long-term lymphocytopenia (<1.0 × 10^9^/L, median—15 days)—85% patients, glucocorticosteroids use, including high-dose pulse therapy in the treatment of acute respiratory distress syndrome (ARDS)—85%, and immunosuppressive therapy (tocilizumab)—48%. Neutropenia was not typical—15%.

The primary site of infection were lungs—100%, specific tracheobronchitis was detected in 4%.

COVID-IA is characterized by a severe course, 85% patients stay in ICU, ARDS was noted in 44%. The main clinical manifestations were temperature > 38.5—100%, respiratory failure—93%, cough—81%, hemoptysis—37%, and chest pain—26%.

CT-scan signs were nonspecific, bilateral lung involvement was noted in 100% patients, infiltrative changes—88%, cavities of destruction—44%, and hydrothorax—11%. The positive galactomanann test in BAL was obtained in 63% patients. Mycological examination of BAL fluid was carried out in 44% patients, septated mycelium was found in 19%, positive *Aspergillus* spp. cultures—42%. The etiology agents of IA were *A. niger* (50%), *A. fumigatus* (33%), and *A. flavus* (17%).

All patients received antifunfal therapy: voriconazole predominantly (85%), caspofungin (33%), micafungin (7%), and amphotericin B (7%). Combination of antimycotics was used in 26% patients. The median duration of antifungal therapy was 17.5 (2–90) days. Overall 12 weeks survival rate was 54%.

**Conclusions:** CAPA developed in patients with diabetes mellitus (33%), non-Hodgkin’s lymphoma—19%, COPD (11%) and malignant neoplasms (7%). Risk factors: lymphocytopenia (85%), glucocorticosteroid (85%) and immunosuppressive therapy (48%). The main pathogens were *A. niger* (50%), *A. fumigatus* (33%), and *A. flavus* (17%). CAPA characterized by lung involvement (100%), the ARDS development (44%), and hemoptysis (37%). All patients received antifungal therapy, and the 12 weeks overall survival rate was 54%.

## P306 Antifungal Stewardship in COVID-19 Patients

**Emre Kara ^1^, Gokhan Metan ^2^, Aygin Bayraktar-Ekincioglu ^1^, Seda Banu Akinci ^3^, Arzu Topeli ^4^ and Omrum Uzun ^2^**

^1^ Hacettepe University Faculty of Pharmacy, Department of Clinical Pharmacy
^2^ Hacettepe University Faculty of Medicine, Department of Infectious Diseases and Clinical Microbiology
^3^ Hacettepe University Faculty of Medicine, Department of Anesthesiology and Reanimation, Division of Intensive Care
^4^ Hacettepe University Faculty of Medicine, Department of Internal Medicine, Division of Intensive Care

**Objectives:** The risk of invasive fungal infections (IFIs) particularly invasive pulmonary aspergillosis (IPA) is of major concern during Coronavirus Diseases 2019 (COVID-19) pandemic. This study was aimed to examine the appropriateness of antifungal treatment in COVID-19 patients by implementation of antifungal stewardship.

**Materials & Methods:** Patients with COVID-19 are aged over 18 years and given antifungal therapy at the Hacettepe University Hospitals between April and November 2020 were included in the study. The ethics approval was obtained from the University Clinical Research Ethics Committee. Demographics and treatment characteristics of patients were analyzed. The appropriateness of antifungal treatments was evaluated by using previously developed 6 criteria by Valerio et. al. The recommendations were made regarding the treatment in case of inappropriateness by an antifungal stewardship team.

**Results:** In this study, antifungal treatments were evaluated in 39 patients (35.9% female) with a median age (minimum–maximum) of 68 (21–87) years. The median number of comorbidities was 3 (0–7) and comedications was 19 (3–22). The most common indication for antifungal treatment was for IPA and used antifungal drug was voriconazole (*n* = 18, 27.7%). Thirty-nine (83.0%) antifungal drugs were administered parenterally. A total of 33 recommendations were made by the antifungal stewardship team for 18 (52.9%) patients. Antifungal treatment was appropriate in 31 (79.5%) patients. However, inappropriateness was found in dosage [*n* = 2 (5.1%)], administration route [*n* = 4 (10.3%)], and treatment duration [*n* = 2 (5.1%)] of antifungal treatments (Table 1).

**Conclusions:** COVID-19 becomes more complex disease in patients with several co-morbid disease. Some of the critical components of the antifungal treatment can be overlooked by the main care team due to patient overload. A smooth implementation of antifungal stewardship might be useful to improve the success of antifungal treatment.

**Table 1.** The appropriateness of antifungal treatments.


**Criteria for Appropriateness (*n* = Applicable for Assessment)**
*n* (%)1. Indications (39)39 (100)2. Choice of antifungal (39)38 (97.4)3. Dose of antifungal (37)37 (94.8)4. Microbiological appropriateness (18)18 (100)5. Intravenous-oral switch (11)7 (63.6)6. Treatment duration (21)19 (90.5)

## P310 Characterisation of Programmed Cell Death in a Repeat Challenge Model of Allergic Aspergillosis

**Thomas Williams ^1^, Luis Gonzales-Huerta ^1^, Amelia Bercusson ^2^, Anand Shah ^3^ and Darius Armstrong-James ^1^**

^1^ Department of Infectious Diseases, Imperial College London
^2^ Cystic Fibrosis Unit, University Hospital Southampton NHS Foundation Trust
^3^ Department of Respiratory Medicine, Royal Brompton and Harefield NHS Foundation Trust

**Objectives:** Cystic fibrosis (CF) is a disease caused by mutations of the cystic fibrosis transmembrane conductance regulator (CFTR) protein, resulting in poor ion transfer and impaired mucus formation, leading to persistent infections and increased inflammation in the lung. The opportunistic fungi *Aspergillus fumigatus* is a chronic pathogen found in 6–60% of CF patients, colonising the airways and causing invasive or allergic disease. These infections lead to increased inflammation in the lung which contributes to eventual lung decline and there is growing evidence that intrinsic defects in immune cell function contribute to this pathophysiology. One of these intrinsic defects observed in our lab is that CFTR KO macrophages are more prone to undergo inflammatory cell death in response to *Aspergillus fumigatus*. Whether this cell death is pyroptotic, necroptotic, or some other form of programmed cell death is yet unknown.

We wanted to use an in vivo model of allergic aspergillosis that is most similar to the chronic exposure that most patients would face. Therefore, we aimed to assess a repeat challenge model of allergic aspergillosis to investigate whether it is an appropriate for the characterisation of programmed cell death during aspergillosis and whether it will be suitable for the assessment of therapeutic inhibitors.

**Materials & Methods:** To meet these objectives, we employed a repeat challenge model of *Aspergillus fumigatus* infection. In this model, wild-type mice were sedated by isoflurane inhalation and dosed intranasally with 2 × 10 ^5^ resting CEA-10 *Aspergillus fumigatus* conidia or PBS control, repeated daily for a total of 14 infections (Figure 1). 24 h after the final infection mice were culled by intraperitoneal injection of pentobarbital and either bronchoalveolar lavage or lung inflation and fixation was carried out. BAL fluid was processed into single-cell suspension for immunotyping by flow cytometry and supernatant for inflammatory marker assessment. Inflated lungs were processed for histological assessment.

**Results:** We show that mice subjected to daily *Aspergillus fumigatus* infections have significantly increased numbers of immune cells in BALF and that this increase is characterised mainly by infiltrating eosinophils, neutrophils and monocytes, while resident alveolar macrophages are significantly depleted. This shift in immune cell profile is accompanied by an increase in the cell death markers lactate dehydrogenase and IL-1 as well as significant increases in IL-33 and galectin-1. Histology showed that there was increased inflammation of the airways as well as marked cell infiltrates in the tissue. Future work includes immunofluorescence histology for the identification of terminal programmed cell death proteins such as cleaved gasdermin D and phosphorylated MLKL.

**Conclusions:** Repeat challenge infection provides an adequate in vivo model of allergic aspergillosis for the assessment of inflammatory markers and host cell death. Using this model, it will be possible to characterise the phenotypic differences of lung diseases that are pre-disposed to allergic brocnhopulmonary aspergillosis, including cystic fibrosis. Not only will we be able to phenotype responses during disease, but the outcomes detailed above provide measurements that can be used to assess the efficacy of immunomodulatory therapeutics in treating inflammation induced during allergic aspergillosis.

## P311 Distribution of IgM and IgG in Granulomatous Lesions of *P. brasiliensis*-Infected Mice Treated with Antifungal and Anti-Inflammatory Drugs

**Lauana Aparecida Santos ^1^, Julianne Caravita Grisolia ^1^, Nayara Andrade Dias ^1^, Bruno José Nascimento Gomes ^1^, Vitor Roberto Souza ^1^, Fernanda Borges de Araújo Paula ^1^, Luiz Cosme Cotta Malaquias ^1^, Adriano Macedo de Oliveira ^2^ and Eva Burger ^1^**

^1^ Federal University of Alfenas
^2^ Alzira Velano University Hospital

**Introduction:** Paracoccidioidomycosis is a severe systemic mycosis, caused by the fungus *Paracoccidioides brasiliensis*. The initial infection can be either exudative or granulomatous, and the presence of granulomas characterizes this disease.

**Objetive:** To evaluate the effectiveness of the combined treatment of paracoccidioidomycosis with the antifungal Itraconazole and the anti-inflammatory Celecoxib drugs.

**Methods:** Swiss mice were infected intraperitoneally with the virulent Pb18 *P. brasiliensis* strain and three days after infection, treatment with Itraconazole (3 mg/mL), Celecoxib (6 mg/mL) or combination of Itraconazole (3 mg/mL) and Celecoxib (6 mg/mL) was initiated. The drugs were administered daily by gavage for 15 days and on alternate days for 120 days. Survival rate was monitored daily and blood was collected for serum at 7, 15, 30, 60, 90, 120 days post-infection for IgM and IgG measurement by ELISA. At the last day of treatment, the mice were sacrificed and spleen, lungs, liver and epiploon/pancreas were collected. The organs were processed and stained with H/E for general histology and for quantification of viable fungi by counting colony forming units. The epiploon (target organ) was processed and specific reagents for immunohistochemistry were used for IgM and IgG antibodies immunolocalization both in the groups of mice with acute (15 days) and chronic (120 days) models of infection.

**Results:** The survival rate was not altered with either treatment 15 and 120 days after infection. In the acute model of infection there was a peak of IgM production at the 7th day in all groups. In the chronic infection, a peak of IgM production was found at the 15th day, but remained constant, with no significant differences until the 120th day. IgG levels showed no significant alterations at the 15th day in the chronic model, in all the groups (infected and untreated as well as infected and undergone the different treatments). In chronic infection, there was a peak of IgG production at the 60th day of infection. However, IgG levels tended to decrease, especially in the group of mice that received the combined antifungal plus anti-inflammatory treatment. In this later group, less edema, inflammatory exudate and altered organ coloration were found, as well as lower viable fungi counts in all organs. An intense, statistically significant immunostaining for IgM in the epiploon from mice that had undergone combined treatment was found only at the 15th day but not at the 120th post-infection and treatment. On the other hand, there was an intense statistically significant immunostaining in the epiploon from mice subjected to the combined treatment at the 120th days of infection, but not at the 15th day post-infection and treatment.

**Conclusions:** The presence of antibodies in situ in the lesions may suggest a positive response and improvement of the infectious process in mice with the combined treatment. This fact is reinforced since there was a reduction in the number of viable fungi. These results allow the conclusion that the combined treatment with antifungal and anti-inflammatory drugs may be a new therapeutic strategy for paracoccidioidomycosis.

**Scholarships and Grants:** 5. CAPES, 6. FAPEMIG grant # 309917/2020–4.

## P312 Antibiofilm Effects of Human Neutrophils in Combination with Liposomal Amphotericin B or Voriconazole against *Fusarium* spp. and *Scedosporium apiospermum* Biofilms

**Aikaterini Vikelouda ^1^, Maria Simitsopoulou ^1^, Charalampos Antachopoulos ^1^, Lemonia Skoura ^2^ and Emmanuel Roilides ^1^**

^1^ 3rd Department of Pediatrics, Aristotle University, School of Medicine
^2^ Department of Microbiology, Aristotle University, School of Medicine

**Objectives:** *Fusarium* and *Scedosporium* species are emerging opportunistic pathogens, causing invasive fungal diseases in humans. *Fusarium* spp. have evolved to be the second most common nosocomial fungal pathogen among immunocompromised patients, while in some countries *Scedosporium* spp. have emerged as the second most frequent mold pathogen associated with respiratory infections following *Aspergillus* spp. Both organisms can grow in biofilms (BF) on solid and tissue culture surfaces. Biofilms are resistant to innate mechanisms of defense. We assessed the antibiofilm activity of human polymorphonuclear neutrophils (PMN) alone or in combination with liposomal amphotericin B (L-AMB) and voriconazole (VRC) against *Fusarium solani* (FS) and *Scedosporium apiospermum* (SA) mature biofilms.

**Materials & Methods:** 10^5^ cfu/mL of *FS* (*n* = 3) and *SA* (*n* = 3) BF-producing strains were incubated in RPMI at 37 °C for 48 h. PMN were isolated from healthy volunteers by dextran sedimentation/ficoll centrifugation. BF were incubated with PMN at effector-to-target ratio 5:1 alone or in combination with L-AMB (*FS*: 0.25/2/32 mg/L; *SA*: 0.25/1/16 mg/L) or VRC (*FS*: 8/64 mg/L; *SA*: 0.5/32 mg/L) at 37 °C/5% CO_2_ for 24 h. Antifungal activity was assessed by XTT reduction assay of metabolic activity. MIC50 was determined as ≥50% BF damage. The % damage of the PMN+drug treatment was compared to that of PMN or drug alone by ANOVA (*n* = 8, *p* < 0.05). Synergism was defined as significantly greater damage by the combination than by PMN plus drug alone. Additivity was defined as significantly greater damage by the combination than by either PMNs or drug alone.

**Results:** LAMB and VRC BF MIC50’s for *FS* were 2 and >256 mg/L, whereas MIC50’s for *SA* were 2 and 32 mg/L, respectively. PMN-mediated damage against *FS* BF was 4.5 ± 2.1%. While an additive effect was demonstrated between 2 mg/L L-AMB+PMN vs. L-AMB, antagonism against *FS* BF damage was found at drug concentrations of 32 mg/L L-AMB+PMN vs. L-AMB and 64 mg/L VRC+PMN vs. VRC. In contrast, the PMN-mediated damage against *SA* BF was 12 ± 2%, while an additive effect was observed for selective combinational treatments with L-AMB and VRC against *SA* BF: L-AMB 0.25 mg/L+PMN:15 ± 3.5% vs. L-AMB: 5.5 ± 7% and VRC 32 mg/L+PMN: 69.5 ± 3% vs. VRC: 61 ± 4% or PMN:12 ± 2%. None of the combinations exhibited antagonism against *SA* BF damage.

**Conclusions:** Biofilms of *F. solani* and *S. apiospermum* appear to be relatively more resistant to VRC than L-AMB. The combined treatment of PMN with L-AMB shows synergistic or additive activities at low concentrations compared to VRC against *S. apiospermum* BF, having potential implications in clinical practice. In contrast, the combined treatment of PMN with L-AMB or VRC against *F. solani* BF shows antagonistic activities at certain concentrations.

## P313 Primary Human Airway Epithelium: A Model to Study Host-Fungal Interactions That Drive the Innate Immune Response

**Jatin Vyas ^1,2^**

^1^ Massachusetts General Hospital
^2^ Harvard Medical School

**Objectives:** Current methods to evaluate the contribution of airway epithelium in coordinating the innate immune resposne to fungal infections are limited. Immortalized cell lines do not fully recaptulate essential functions and diversity of cell types seen in primary human airways, limiting our conclusions using these models. Moreover, mouse models show a remarkable reistance to many pathogenic fungal organisms for humans. We adapted a novel model using primary human airway basal stem cells to primary human airway epithelium that faithfully replicates human airway.

**Materials & Methods:** We use inverted air-liquid interface cultures to demonstrate that the human airway epithelium responds to apical stimulation by *A. fumigatus* to promote the transepithelial migration of neutrophils from the basolateral membrane surface to the apical airway surface.

**Results:** Promoting epithelial transmigration with *Aspergillus* required prolonged exposure with live resting conidia. Swollen conidia did not expedite epithelial transmigration. Using *A. fumigatus* strains containing deletions of genes for cell wall components, we identified that deletion of the hydrophobic rodlet layer or dihydroxynaphthalene-melanin in the conidial cell wall amplified the epithelial transmigration of neutrophils, using primary human airway epithelium. Ultimately, we show that an as-yet-unidentified nonsecreted cell wall protein is required to promote the early epithelial transmigration of human neutrophils into the airspace in response to *A. fumigatus*

**Conclusions:** In the present study, we demonstrate that direct epithelial cell-conidium contact via a non-heat-labile protein stimulates neutrophil recruitment to the airways following *A. fumigatus* infection. Furthermore, melanin masks this protein, resulting in decreased neutrophil recruitment.

## P314 Neutrophil Responses to *C. albicans* in Disease-Specific States

**Michael Mansour**

Massachusetts General Hospital/Harvard Medical School

**Objectives:** Neutrophils are a first-line defense against invasive fungal infections including *Candida albicans*. Patients with a loss of neutrophils or dysfunctional innate immunity are at high risk for infection from *C. albicans*. Two such populations include solid organ (SOT) and stem cell transplant (SCT) recipients. Both are susceptible patient populations at an increased risk of invasive fungal disease despite normal neutrophil counts. In this study, our objective was to measure neutrophil anti-*Candida* response from SOT and SCT recipients compared to healthy controls to accurately determine functional defects against *C. albicans*.

**Methods and Materials:** Twenty-one SOT, 19 SCT and 23 healthy control patients (HC) were identified and consented at primary care/outpatient transplant clinics. Transplant recipients were enrolled at a time period two to four months post-transplant. Following consent and peripheral blood collection, neutrophils were freshly isolated and co-incubated with *C. albicans* and the percent remaining live yeast was measured using a viability dye compared to a standard control to determine fungal elimination by neutrophils. Growth inhibition of *C. albicans* was also quantified by neutrophil swarming to yeast spotted onto glass slides, assessed by live cell imaging with or without G-CSF. Effect of soluble factors on neutrophils function was determined by incubation of healthy neutrophils with SOT and SCT serum and measuring ability to control *C. albicans*.

**Results:** Neutrophils isolated from SCT and SOT patients had decreased fungicidal capacity compared to HC (SCT: 76% ± 24%, SOT: 73% ± 23%, HC: 86% ± 10%, *p* < 0.0001) and diminished ability to control hyphal growth. In addition, serum from SCT and SOT patients inhibited the ability of healthy control neutrophils to control *C. albicans* when compared to serum from healthy controls (55% ± 16, 42% ± 16, 49% ± 15; respectively. SCT vs. HC *p* < 0.001, SOT vs. HC *p* = 0.555, SOT vs. SCT *p* = 0.1341). Neutrophil’s ability to control hyphal growth was partially restored following treatment with G-CSF or GM-CSF.

**Conclusions:** Despite normal circulating numbers, our study demonstrates that neutrophils from SOT and SCT recipients display a profound PMN dysfunction against *C. albicans*. These neutrophil defects are related to both, intrinsic variables to PMN as well as inhibitory effect from extrinsic soluble serum components. Furthermore, this neutrophil dysfunction may be partially reversed with cytokine augmentation ex vivo.

## P315 Inflammatory Response of Individual or Co-Cultured Macrophages and Epithelial Cells Following Exposure to Cigarette Smoke Extract and/or *Aspergillus fumigatus* Spores

**Alexandra Bouyssi ^1^, Marie Delles ^1^, Tanguy Déméautis ^1^, Fany Agostini ^1^, Florence Persat ^1,2^, Gilles Devouassoux ^1,2^, Azzak Bentaher ^1^, Jean Menotti ^1,2^**

^1^ Université Claude Bernard Lyon 1
^2^ Hospices Civils de Lyon

**Objectives:** Lung exposure to *Aspergillus fumigatus* spores (AFS) results in abnormal airway inflammation, responsible for exacerbation of Chronic Obstructive Pulmonary Disease (COPD), an irreversible inflammatory and tissue-destructive disease whose main etiologic factor is cigarette smoke. Using relevant cell culture models, the aim of the study was to compare the inflammatory processes involved in response to AFS and/or cigarette smoke extract (CSE) exposure.

**Materials & Methods:** To better understand these inflammatory responses, monolayers of murine macrophages RAW 264.7 and/or alveolar epithelial cells MLE-15 were exposed to CSE and/or viable *AFS*. Gene expression of a large panel of cytokines was then analyzed and compared within these different conditions. The presence of the two cell types in coculture was confirmed using immunofluorescent microscopy and anti-CD68 and anti-E-cadherin antibodies to immunostain macrophages and epithelial cells respectively. The expression of genes of interest was quantified by RT-qPCR using TaqMan™ gene expression assays. Data were then analyzed by two-way ANOVA with Bonferroni’s multiple comparisons tests.

**Results:** Treatment of macrophages by AFS led to significant gene expression induction of *IL-1β, IL-1α, MIP-2, CXCL-1* and *GM-CSF*. In CSE-treated macrophages, we observed that gene expression of *IL-1α* and *MIP-2* was significantly induced. Exposure of epithelial cells to AFS led to an induction of *IL-1α, MIP-2, CXCL-1* and *TNF-α* gene expression whereas treatment with CSE alone resulted in significant induction of *IL-1α* gene expression. In co-cultured cells treated by AFS, we observed a significant induction of gene epxression for *IL-1β, CXCL-1, MIP-2, TNF-α, GM-CSF* and *IL-1α*, whereas CSE alone induced a significant increase of *MIP-2* gene expression, and a significant decrease of *IL-1β* and *GM-CSF* gene expression. In cocultured cells, after treatment with both CSE and AFS, gene expressions of *MIP-2, TNF-α* and *IL-1α* were significantly increased when compared with treatment with CSE alone.

**Conclusions:** Individual or cocultured macrophages and alveolar epithelial cells respond differently to AFS and CSE exposure displaying specific inflammatory patterns. Our date also allude to the presence of different inflammatory cascades, involved in exacerbated and stable COPD. In particular, the induction of MIP-2, TNF-α and IL-1α gene expression suggests the importance of these cytokines in the AFS-mediated exacerbation phase of COPD that is usually characterised by massive infiltration of neutrophils. The present experimental cell culture model could be useful to further enhance our understanding of COPD pathogenesis.

## P316 Immunoglobulin Subclass Levels in Cryptococcosis Patients from Colombia

**Paola Becerra, Patricia Escandón, Carolina Firacative**

Universidad Del Rosario

**Objectives:** Cryptococcosis, a mycosis caused by *Cryptococcus neoformans* (*Cn*) and *Cryptococcus gattii* (*Cg*), occurs globally mostly in adults. This study aims to determine total and specific antibodies against *C. neoformans* and *C. gattii* antigens in sera from patients with cryptococcosis and from healthy individuals from Colombia, which will help to elucidate sero-epidemiological variations in the incidence of the disease in the country.

**Materials & Methods:** Sera from child and adult patients with *Cn-* and *Cg*-cryptococcosis (*n* = 109) and sera from healthy children and adults from Colombia (*n* = 100) were studied. Using ELISA, total levels of immunoglobulin (Ig)G, IgA and IgM were determined in sera.

**Results:** Total IgG, IgA and IgM levels were higher in HIV+ compared with HIV- patients with cryptococcosis. In adults, total IgA and IgM levels were higher than in children. Regarding the etiological agent, *Cn*-cryptococcosis patients presented higher total IgG and IgM levels (*p* < 0.05). Antibody levels did not differ between male and female patients (*p* > 0.05). Including all samples, a moderate positive correlation between total IgA and IgM levels was found (r = 0.305).

**Conclusions:** In cryptococcosis patients from Colombia, serum immunoglobulins levels differ depending on HIV status, as reported previously. However, this study shows for the first-time variations in immunoglobulin production among adults and children with cryptococcal disease and between *Cn* and *Cg*-cryptococcosis patients. Analyses including healthy Colombian patients are currently being carried out. Identification of specific cryptococcal antigens that are involved in the pathogenesis of *C. neoformans* and *C. gattii* are still needed.

## P317 Mycobacterial Modulation of Macrophage Responses to *Aspergillus fumigatus*

**Luis Gonzales-Huerta, Thomas Williams, Renad Aljohani, Valentina Capizzuto, Brian Robertson, Carlton Evans and Darius Armstrong-James**

Department of Infectious Diseases, Imperial College London

**Objectives:** *Mycobacterium tuberculosis and atypical mycobacteria* have been associated with an increased susceptibility to fungal infection. It has been estimated that 1.2 million people worldwide have chronic pulmonary aspergillosis (CPA) secondary to pulmonary tuberculosis (TB). However, the underlyning mechanisms that explain an increased susceptibility to *Aspergillus fumigatus* (*Af*) are unknown. Multiple strategies are employed by Mycobateria which enable survival in the intracelllular space, thereby disrupting host immune pathways. Some of these strategies may impair the clearance of other intracellular pathogens, such as *Af* (Herbst et al., 2015). We hypothesized that the persistence of mycobacteria in the myeloid compartment modulates the immune response against *Af*, contributing to increased susceptibility to develop CPA. Thus, we investigated the effect of mycobacterial virulence factor, lipoarabinomannan, on macrophage responses to *Af*.

**Materials & Methods:** The effect of lipoarabinomannan (LAM) from *Mycobacterium smegmatis* or mannose-capped lipoarabinomannan (ManLAM) from *Mycobacterium tuberculosis* on *Af* was assesed by measuring conidia swelling, germination, and hyphae growth rate using wide-field microscopy. Fungal metabolism of carbon energy sources was determined by applying the Omnilog metabolomic platform based on redox reactions.

J774A.1 and murine bone marrow-derived macrophages (BMDMs) were treated with LAM or ManLAM for 2 h prior to infection with swollen *Af*. Cell death was determined by measuring the release of lactate dehydrogenase. The release of inflammatory cytokines were measured by ELISA, and time-lapse fluorescence microscopy was applied to study phagocytosis, conidia killing, and phago-lysosome fusion. Culture in Sabouraud agar of lysed macrophages’ content was performed for counting colony-forming units. Finally, the expression of pathogen recognition receptors (PRRs) was studied by flow cytometry.

**Results:** Neither LAM nor ManLAM showed a direct change on *Af* development. Treatment with LAM increased macrophage susceptibility to *Af* in a dose-dependent manner from 6 h post infection(hpi). Increased cytotoxicity was associated with increased release of inflammatory cytokines TNF-α, IL-1β and CXCL1. LAM also reduced colocalization of lysosome and *Af* conidia at early stage of infection, and increased the yield of CFUs by lysed macrophages at 3hpi. Additional preliminary data suggest that LAM might reduce intracellular killing of *Af* conidia and increase the expression of NOD2 on macrophage.

**Conclusions:** With this work we have demostrated that treatment of macrophages with LAM disrupts the response against *Af* in a dose-dependent manner. LAM is a TLR-2 agonist, which triggers the MyD88-dependent intracellular pathway and NOD2 has been shown to have an important dose-dependent effect on TLR2-mediated cytokine release (Borm et al., 2008). Therefore, our findings coincide with current scientific literature, which shows that mycobacteria increases inflammatory cytokine release via upregulation of NOD2 signalling (Brooks et al., 2011), but reduces antifungal activity in macrophages (Gresnight et al., 2018). To our knowledge, this is the first time evidence is shown on the potential mechanism behind increased susceptibility to *Af* in the context of mycobacterial infection.

## P318 Granulocyte-Macrophage Colony Stimulating Factor (GM-CSF) as Adjuvant Therapy for Invasive Fungal Diseases in Pediatric and Adult Patients

**Tempe Chen, Jagmohan Batra, David Michalik, Jacqueline Casillas, Maritza Ruiz, Ramesh Patel, Catherine Small, Panagiotis Zagaliotis, Carolyn Ragsdale, Louis Leal, Emmanuel Roilides and Thomas Walsh**

New York Presbyterian Hospital and Weill Cornell Medicine of Cornell University

**Objectives:** Granulocyte-macrophage colony stimulating factor (GM-CSF) augments innate host immune responses against pathogenic fungi and accelerates hematopoietic recovery of chemotherapy-induced neutropenia. However, the clinical efficacy of GM-CSF as an adjunctive immunotherapy for invasive fungal infections is not well understood.

**Materials & Methods:** We analyzed the clinical courses of 10 patients with pediatric malignancies and invasive fungal diseases (IFDs) who were treated adjunctively with the sargramostim form of GM-CSF from a single institution. We further conducted a systematic review of published case reports and series on the use of GM-CSF for invasive fungal infections from two electronic databases (PubMed and Embase, 1990–2020).

**Results:** Newly reported pediatric patients.

Among the 10 pediatric patients (1–16 years) who received sargramostim for adjunctive therapy, IFDs consisted of those caused by *Candida* spp. (n = 7), *Aspergillus* spp. and *Rhizopus* spp. coinfection (n = 1), *Scedosporium* spp. (n = 1), and *Trichosporon* spp. (n = 1) (Table 1). The majority (80%) was neutropenic at baseline, and 8 patients were considered to be refractory to prior antifungal therapy. Sargramostim was initiated at a dosage of 250 µg/m^2^/day twice weekly if the ANC was less than 500 cells/µL, then reduced to 100 µg/m^2^/day when counts exceeded 500 cells/µL. Of the 10 patients treated, 7 complete responses (resolution of infection) were achieved, with an additional 3 partial responses for an overall response rate of 100% in all 10 treated patients. Moreover, with the addition of sargramostim, patients were able to successfully complete scheduled chemotherapy and/or undergo planned hematopoietic cell transplantation.

Previously published pediatric and adult patients.

Previously published case reports on the use of GM-CSF for adjunctive treatment of IFDs are summarized in Table 2. Among the 41 patients with IFDs, were those with invasive candidiasis and invasive mould diseases, including those caused by *Aspergillus* spp., *Mucorales*, and *Fusarium* spp. Sixteen (39%) had baseline neutropenia and 27 (66%) were considered to be refractory to standard therapy prior to GM-CSF administration. Among the 41 patients with IFDs, complete responses were reported in 35 (85%) patients and partial response in 1 patient, with 5 patients having no or unclear response.

**Conclusions:** GM-CSF, including sargramostim, may serve as an effective adjunctive immunomodulatory treatment, for patients with IFDs that are resistant to standard therapy, at risk for developing resistance, and/or at risk for toxicities with other therapies. Randomized clinical trials are needed to better define the patients and pathogens for which GM-CSF is most effective and to identify biomarkers that could predict response.

## P319 Impact of Cigarette Smoke Exposure on Macrophages Response to Airborne Moulds

**Florian Leprêtre ^1^, Maxime Paluch ^1^, Muriel Pichavant ^1^, Philippe Gosset ^1^ and Emilie Fréalle ^1,2^**

^1^ Institut Pasteur de Lille, U1019—UMR 9017—CIIL—Center for Infection and Immunity of Lille, University Lille, CNRS, Inserm, CHU Lille
^2^ CHU Lille, Laboratoire de Parasitologie-Mycologie

**Objectives:** Chronic Obstructive Pulmonary Disease (COPD) is a poorly known chronic inflammatory disease, mostly caused by cigarette smoke exposure, which affects 8% of subjects over 45 years-old in France. Although COPD exacerbations are mainly caused by bacteria or virus, COPD is also a risk factor for invasive aspergillosis (1.6–3.9% incidence), which is often lethal. Furthermore, domestic mould exposure is known to be involved in asthma. But the impact on COPD patients is poorly known.

**Materials & Methods:** Since macrophages functionality is altered during COPD, and regarding their essential role in the defense against moulds, we developed an experimental model on human monocyte-derived macrophages by inoculation with different fungi (*Aspergillus fumigatus, Aspergillus niger, Scopulariopsis brevicaulis* and *Paecilomyces variotii*) in standard culture or after exposure to cigarette smoke extract (CSE). Mould viability and immune response were studied via the quantification of fungal biomass by species-specific qPCR, expression of receptors involved in the antifungal response, phenotyping of macrophages using flow cytometry, and analysis of inflammatory response by ELISA determination of cytokines concentrations.

**Results:** Increased quantities of fungal DNA and increased hyphal elongation were observed when macrophages were exposed to CSE. CSE induced macrophages maturation, as shown by a significant increase in CD80, CD86 and/or HLA-DR, whereas expression of CD54 was decreased. Lastly, *A. fumigatus* and *A. niger* yielded an increase in TLR2 expression, which was inhibited in the presence of CSE.

**Conclusions:** These data indicate that cigarette smoke exposure is associated with a defective control of fungal growth by macrophages. Inhibition of *A. fumigatus* and *A. niger*-induced TLR2 expression suggests this receptor could be involved in impaired response against these 2 moulds.

## P320 Modulation of the Vaginal Microbiota Impacts Long-Term Innate Immune Response

**Diletta Rosati ^1^, Mariolina Bruno ^1^, Arnab Pradhan ^2^, Jorge Domínguez Andrés ^1^, Mark Gresnigt ^3^, Alistair Brown ^2^ and Mihai Netea ^1^**

^1^ Department of Internal Medicine and Radboud Center for Infectious Diseases
^2^ MRC Centre for Medical Mycology, University of Exeter
^3^ Microbial Immunology Research Group, Hans Knöll Institute

**Keywords:** vulvovaginal candidiasis; vaginal microbiota; *Candida albicans* infection; host defence; innate immunity; trained immunity

**Objectives:** Anually over 75% of women of childbearing age suffer from at least one episode vulvovaginal candidiasis (VVC), with up to 9% suffering from recurrent episodes (RVVC). Among the mucosal surfaces colonised by *Candida* species, the vaginal mucosa continuosly adapts to changes in the enviroment, i.e., carbon sources avaialbility, pH, and estrogen. Alterations in the vaginal microbiota can force *Candida* to undergo metabolic reprogramming, which can lead to cell wall remodelling. Therefore, we investigated whether growth in a lactic acid-depleted environment, reminescent of a dysbalanced vaginal microbiota, influences *C. albicans* pathogenicity and its recognition by the innate immune system.

**Materials & Methods:** To mimick the vaginal enviroment, *C. albicans* was grown either in the absence or presence of lactic acid in Vaginal Simulative Medium (VSM). Cytokine release by neutrophils and Peripheral Blood Mononuclear Cells (PBMCs) was assessed in response to *C. albicans* grown under the different conditions. Induction of innate immune memory against non-specific reinfection was evaluated by measuring pro-inflammatory cytokines TNF and IL-6. In addition.

**Results:** In response to *C. albicans* grown under lactic acid-depleted conditions, neutrophils released significantly higher IL-8 levels compared to lactic acid grown *C. albicans*. In PBMCs innate and adaptive immune responses to *C. albicans* were differentially shaped by the conditions under which the yeast cells were grown. This was characterized by decreased innate and adaptive responses, except for IL-22 production, under lactic acid-depleted condition. Interestingly, exposure of monocytes to *C. albicans* grown in the absence of lactic acid resulted in enhanced pro-inflammatory state when the cells received secondary stimulation with non-specific stimuli.

**Conclusions:** Collectively, these results demonstrate that lactic acid in the vaginal environment modulates both early and long-term immune responses via altering fungal biology. A better understanding of how lactic acid depletion modulates *C. albicans* cell wall remodelling and pathogenicity mechanisms could aid the understanding of how changes in the microbiota shape inflammatory responses, which underlie the pathogenesis of VVC.

## P321 Toll-like Receptor 2 and 4 Expression in Feline Dermatophytosis Lesions

**Anahita Kasmaie ^1^, Alireza Salimi ^1^, Farzad Katiraee ^1^, Javad Ashrafi Helan ^1^ and Ali Shabestari Asl ^2^**

^1^ University of Tabriz
^2^ Tabriz Branch, Islamic Azad University

**Objectives:** Dermatophytosis is a common skin disease in cats and there are few studies on the role of innate immune response in dermatophytosis. Fungal pathogens are detected by specific receptors on cells from the innate immune system. The recognition of pathogen fungal structures by pattern recognition receptors (PRRs) leads to the secretion of pro-inflammatory cytokines by immune cells. Toll-like receptors (TLRs) 2 and 4 are the main PRRs that recognize fungal components. The aim of this study was to evaluate the expression of TLR-2, TLR-4 in feline dematophtosis lesions.

**Materials & Methods:** A total of 60 cats with skin lesions were examined from Jun 2019 to October 2020. Hair and skin samples were examined by microscopy with 20% KOH and cultured on SCC. Also two skin biopsies were taken by sterile, single use biopsy punch from margins of annular lesions for pathology and Real-time PCR studies.

**Results:** Dermatophytes species were found in 20 (33.33%) samples and *Microsporum canis* were isolated from cultures. Real time PCR showed the increase of TLR-2 and TLR-4 mRNA levels in skin biopsies of cats with dermatophytosis.

**Conclusions:** Increased expression of TLR-2 and TLR-4 mRNAs in feline skin biopsies suggests that these receptors are involved in the host immune response through the recognition of dermatophytosis.

## P322 Granulocyte-Macrophage Colony Stimulating Factor (GM-CSF) as Adjuvant Therapy for Invasive Fungal Diseases

**Tempe Chen, Jagmohan Batra, David Michalik, Jacqueline Casillas, Maritza Ruiz, Ramesh Patel, Catherine Small, Panagiotis Zagaliotis, Carolyn Ragsdale, Luis Leal, Emmanuel Roilides and Thomas Walsh**

New York Presbyterian Hospital and Weill Cornell Medicine of Cornell University

**Objectives:** Granulocyte-macrophage colony stimulating factor (GM-CSF) augments innate host immune responses against pathogenic fungi and accelerates hematopoietic recovery of chemotherapy-induced neutropenia. However, the clinical efficacy of GM-CSF as an adjunctive immunotherapy for invasive fungal infections is not well understood.

**Methods:** We analyzed the clinical courses of 10 patients with pediatric malignancies and invasive fungal diseases (IFDs) who were treated adjunctively with the sargramostim form of GM-CSF from a single institution. We further conducted a systematic review of published case reports and series on the use of GM-CSF for invasive fungal infections from two electronic databases (PubMed and Embase, 1990–2020).

**Results:** Newly reported pediatric patients.

Among the 10 pediatric patients (1–16 years) who received sargramostim for adjunctive therapy, IFDs consisted of those caused by *Candida* spp. (n = 7), *Aspergillus* spp. and *Rhizopus* spp. coinfection (n = 1), *Scedosporium* spp. (n = 1), and *Trichosporon* spp. (n = 1) (Table 1). The majority (80%) was neutropenic at baseline, and 8 patients were considered to be refractory to prior antifungal therapy. Sargramostim was initiated at a dosage of 250 µg/m^2^/day twice weekly if the ANC was less than 500 cells/µL, then reduced to 100 µg/m^2^/day when counts exceeded 500 cells/µL. Of the 10 patients treated, 7 complete responses (resolution of infection) were achieved, with an additional 3 partial responses for an overall response rate of 100% in all 10 treated patients. Moreover, with the addition of sargramostim, patients were able to successfully complete scheduled chemotherapy and/or undergo planned hematopoietic cell transplantation.

Previously published pediatric and adult patients.

Previously published case reports on the use of GM-CSF for adjunctive treatment of IFDs are summarized in Table 2. Among the 41 patients with IFDs, were those with invasive candidiasis and invasive mould diseases, including those caused by *Aspergillus* spp., *Mucorales*, and *Fusarium* spp. Sixteen (39%) had baseline neutropenia and 27 (66%) were considered to be refractory to standard therapy prior to GM-CSF administration. Among the 41 patients with IFDs, complete responses were reported in 35 (85%) patients and partial response in 1 patient, with 5 patients having no or unclear response.

**Conclusions:** GM-CSF, including sargramostim, may serve as an effective adjunctive immunomodulatory treatment, for patients with IFDs that are resistant to standard therapy, at risk for developing resistance, and/or at risk for toxicities with other therapies. Randomized clinical trials are needed to better define the patients and pathogens for which GM-CSF is most effective and to identify biomarkers that could predict response.

## P323 Formation of Inducible Bronchus-Associated Lymphoid Tissue (iBALT) Is Associated with Pulmonary Clearance of Pneumocystis in Immunocompetent *P. murina*-Infected Mice

**Elena Charpentier ^1,2^, Sandie Ménard ^2^, Catherine Marques ^2^, Nicolas Blanchard ^2^, Antoine Berry ^1,2^ and Xavier Iriart ^1,2^**

^1^ Medical Mycology Laboratory—CHU de Toulouse
^2^ EPIIC—Infinity INSERM, Toulouse University III

**Objectives:** *Pneumocystis* pneumonia remains nowadays one of the deadliest fungal infections for immunocompromised patients. Conversely, an immunocompetent host (Human or rodent) is able to eliminate *Pneumocystis* sp. without developing any respiratory symptoms. The immune physiological response occurring in healthy hosts is still poorly known. In order to better understand the implicated cells and their interaction for an effective elimination of *Pneumocystis* sp., histopathological evaluations of lung sections and immunophenotyping were performed in immunocompetent and immunocompromised mice infected with *P. murina*.

**Materials & Methods:** The lungs sections from four groups of balb/c mice were compared: two groups of *P. murina*-infected mice with or without corticosteroid immunosuppression, and two control groups of uninfected mice (with or without corticosteroid). The infection was performed intranasally with 10^6^ *P. murina* cysts while uninfected mice received sterile PBS. The lungs were collected at 1, 3 and 6 weeks post instillation (p.i) in the four groups. Lung sections were stained with hematoxilin-eosin and Gomori-grocott. Immunohistochemistry stainings were also performed targeting T lymphocytes (anti-CD3), B lymphocytes (anti-B220), polymorphonuclear neutrophils (PMN) (anti-L6yg) and macrophages (anti-F480) on separate lung sections. In the four groups, lymphocytes numbers were evaluated in the lung tissue and in the spleen by flow cytometry, among which T follicular helper cells (Tfh), and B cells. *P. murina* specific qPCR on lung tissue and an evaluation of respiratory rate were also performed.

**Results:** Immunocompetent infected mice had large leukocyte infiltrations around bronchi and vessals at 3 weeks p.i. There was almost no infiltration left at 6 weeks p.i, when the fungus was cleared. Immunochemistry revealed that these infiltrates were composed mostly of B lymphocytes, closely located to T lymphocytes and surrounded by macrophages. These peribronchial infiltrates and their composition suggested the structure of Inducible Bronchus-Associated Lymphoid Tissue (iBALT). iBALT are temporary tertiary germinal structures in which there is an important communication between B cells and T lymphocytes, that are for the most part TCD4 Tfh. The increase of Tfh cells in the lungs of infected immunocompetent mice was confirmed by flow cytometry. There was almost no PMN around bronchi or in the parenchyma.

In contrast, immunosuppressed infected mice did not have those iBALT structures at 3 or 6 weeks p.i. However, their lungs parenchyma was infiltrated with leukocytes and their alveolar tissue was filled with an heterogenous exsudate at 6 weeks p.i, concurrently with a high respiratory rate reflecting a reduced pulmonary function. The leukocytes were mostly polymorphonuclear cells, T lymphocytes and macrophages but there were very few B cells.

**Conclusions:** During a *P. murina* infection, immunocompetent mice develop iBALT-like structures bringing together in close proximity B cells and T lymphocytes, probably mostly TCD4 of Tfh phenotype. They then disappear with the fungus clearance. This is consistent with the previous clinical and rodent studies on *Pneumocystis* sp. infection underlining the key role of TCD4 and B cells and their communication. In case of corticosteroid immunosuppression, those structures did not form, possibly because of a B cell important inhibition.

## P325 Fungal and Host Protein Persulfidation Are Functionally Correlated and Modulate Both Virulence and Antifungal Response

**Monica Sueiro-Olivares ^1^, Jennifer Scott ^1^, Sara Gago ^1^, Dunja Petrovic ^2^, Emilia Kouroussis ^2^, Jasmina Zivanovic ^2^, Yidong Yu ^3^, Marlene Strobel ^3^, Cristina Cunha ^4^, Darren Thomson ^1^, Rachael Fortune-Grant ^1^, Sina Thusek ^1^, Paul Bowyer ^1^, Andreas Beilhack ^3^, Agostinho Carvalho ^4^, Elaine Bignell ^5^, Milos R. Filipovic ^6^ and Jorge Amich ^1^**

^1^ MFIG, University of Manchester
^2^ Université de Bordeaux
^3^ University Hospital Würzburg
^4^ University of Minho
^5^ University of Exeter
^6^ Leibniz Institute for Analytical Sciences

**Objectives:** The ability to adapt to the harsh conditions imposed by a host is fundamental for pathogens’ infective capacity. Concomitantly, the host cellular response must be finely tuned to efficiently clear infection. Post-translational modifications (PTMs) are important for fast adaptation and therefore are expectedly crucial for both pathogen and host.

The overall aim of this work was to determine the relevance of the PTM persulfidation for both fungal virulence and antifungal host defence. The particular objectives were:

1. To demonstrate the relevance of persulfidation for *A. fumigatus* virulence.
2. To prove the importance of persulfidation for the host antifungal capacities.
3. To examine if host and fungal persulfidation levels are functionally correlated.
4. To explore if a treatment to increase host persulfidation has beneficial effects on infection.

**Materials & Methods:** We deleted *A. fumigatus* genes to identify the enzymes primarily responsible for persulfidation as confirmed by fluorescence proteomic and microscopic analyses. We then used enzymatic assays, killing experiments and murine models to characterize the relevance of persulfidation for *A. fumigatus* virulence. We performed a genetic association study of the frequencies of a Single Nucleotide Polymorphism (SNP) in the human gene with the probability of suffering Invasive pulmonary aspergillosis (IPA) in hematopoietic stem cell transplant (HSCT) recipients. Next, we investigated the antifungal capacities of host cells using our engineered A549 cell line and alveolar macrophages and neutrophils derived from knock-out mice. Subsequenlty we investigated the interdependency of fungal and human persulfidation levels using Western-blot and fluorescence microscopy. Finally, we performed killing assays in the presence of a sulfide donor to potentiate host persulfidation.

**Results:** An *A. fumigatus* mutant with no persulfidation could not be constructed. A weakly persulfidating *A. fumigatus* Δ*mecB* mutant was more susceptible to host-mediated killing and displayed reduced virulence in two murine models of infection. Besides, we found that a SNP in the human gene encoding the persulfidating enzyme cystathionine-γ-lyase (CTH) reduced enzymatic activity and predisposes to IPA in HSCT recipients. This is likely due to a decreased antifungal activity of lung-resident host cells (alveolar macrophages and epithelial cells) caused by the reduced levels of persulfidation, as suggested by the lower killing capacity and imbalance cytokine production in CTH^−/−^ cells. Interestingly, the level of host persulfidation in host cells, which positively correlate with their antifungal killing capacity, impacted the level of persulfidation that the fungus requires to counteract host attack, reflecting a host-pathogen functional correlation. Finally, a treatment with the sulfide donor GY4137, known to increase persulfidation levels in human cells, enhanced the conidia killing capacity of epithelial cells.

**Conclusions:** We propose that persulfidation is an essential cellular process, show that its correct functioning is required for both fungal virulence and host antifungal defence and demonstrate a functional correlation between persulfidation levels in the host and the pathogen. Moreover, increasing persulfidation levels in host cells enhance their antifungal capacity. Therefore, persulfidation must be considered as a relevant PTM for infection, where its modulation may be a promising and novel strategy to target both pathogens and immune responses.

## P326 Development of a Bioinformatic Tool for the Treatment of WGS Data for Dermatophytes Typing and Characterization: Focus on Terbinafine Resistance

**Rosalie Sacheli ^1^, Maiken Arendrup ^2^, Anuradha Chowdhary ^3^, Wouter Coppieters ^4^, Aymeric Naomé ^5^, Denis Baurain ^5^, Marc Hanikenne ^5^ and Marie-Pierre Hayette ^1^**

^1^ Department of Clinical Microbiology, Belgian National Reference Center for mycoses, University Hospital of Liege
^2^ Department of Microbiology & Infection Control, Statens Serum Institut
^3^ Department of Medical Mycology, Vallabhbhai Patel Chest Institute, University of Delhi
^4^ University Hospital of Liège, GIGA—Genomics Platform
^5^ Hedera 22, Bioinformatics Division

**Objectives:** The present work aims to use the Whole Genome Sequencing (WGS) as a tool to characterize dermatophytes strains. Data generated by WGS are analyzed by using a bioinformatic tool called “WGS typer” and several markers are highlighted, such as genes implicated in resistance to antifungals or genes linked with high virulence in dermatophytes. The tool will also permit to analyze dermatophytes following their genetic diversity and provide similarity dendrograms. The present work focus on squalene epoxidase (SQLE) gene characterization among *T. rubrum* and *T. indotineae* strains by the WGS typer.

**Materials & Methods:** 15 strains of *T. rubrum* (7 resistant to terbinafine and 8 susceptible) and 19 strains of *T. indotineae* (8 resistant to terbinafine and 11 susceptible) from a multicenter study, previously characterized by Eucast E.Def.11.0 method (Arendrup et al., 2020) were used for SQLE characterization by WGS.WGS has been performed by the GIGA genomics platform using the Illumina technology. The WGS Typer is a commercial bioinformatics tool developed by Hedera-22 (http://www.hedera22.com) and licensed to the Department of Clinical Microbiology of the University of Liège. This tool enables high-throughput typing of pathogen isolates based on raw sequencing data and a collection of relevant markers (single genes, gene variants, gene clusters, MLST). The analysis reports the presence/absence of targeted markers or genotypes from a sequence homology search against the assembled sequencing data according to a set of sequence identity/coverage thresholds.

**Results:** We evaluated the ability of the tool to detect mutations in the SQLE gene that are responsible for terbinafine resistance in dermatophytes. Seven *T. rubrum* showed a resistant profile to terbinafine (MIC values >0.25 µg/µL) with the microdilution method. Among these, four shared the F397L mutation on SQLE, one was wearing L393F mutation while two other shared the L393S mutation. All these mutations were efficiently highlighted by the WGS typer. Among the eight strains presenting a MIC value under 0.25 µg/µL, no mutation was found on SQLE gene. Regarding *T. indotineae*, 8 strains were previously characterized to be resistant to terbinafine with the microdilution method (MIC values >0.25 µg/µL). Among them, the WGS typer detected seven strains with the mutation F397L and one strain with the mutation L393F on the SQLE gene. Among the eleven strains presenting a MIC value under 0.25 µg/µL by microdilution, no mutation was found on SQLE. The study was completed with genetic similarity comparisons and dendrogram creation. No clear separation into clusters was observed between resistant/susceptible strains neither in the *T. rubrum* group nor in the *T. indotineae* group. *T. rubrum* and *T. indotineae* species were well separated into two distinct clusters.

**Conclusions:** We present here a valuable and innovative tool for the analysis of dermatophytes. The tool permits to easily and accurately detect mutations on the SQLE gene responsible for terbinafine resistance. A dendrogram of similarity based on WGS data can also be generated.

## P327 Analysis of FKS1 and FKS2 Gene Mutations in Invasive *Candida glabrata* strains from Pakistan

**Saba Memon ^1,2^, Najia Ghanchi ^1^, Urooj Zafar ^2^, Joveria Farooqi ^1^, Sadaf Zaka ^1^ and Kauser Jabeen ^1^**

^1^ Aga Khan University Hospital
^2^ University of Karachi

Keywords: *Candida glabrata*; FKS; echinocandin resistance

**Introduction:** Infections caused by *Candida glabrata* have caused worldwide concern and exhibit greater rates of antifungal resistance than those with other species. Echinocandin resistance in *Candida glabrata* poses a serious clinical challenge. Caspofungin use has been gradually increasing in Pakistan over the past few years, raising the probability of encountering echinocandin resistance in local clinical strains. We sequenced and determined mutation in *FKS1* and *FKS2* genes in invasive *Candida glabrata* strains at the Aga Khan University laboratory, Karachi, Pakistan.

**Material and Method:** Thirty-six clinical isolates of invasive *C. glabrata* strains were analyzed in this study. Minimum inhibitory concentrations (MICs) were determined using colorimetric broth microdilution (YeastOneSensititre, Trek diagnostics). Genomic DNA was isolated using Qiagen DNA extraction Kit. The *FKS1* and *FKS2* gene fragment were amplified and PCR products were purified prior to sequencing. Double-strand sequencing of PCR products was performed using Sanger sequencing methodology. Sequences were analyzed with MEGA-6 software to identify specific SNP combination against wild-type sequences of *C. glabrata*.

**Results:** In our collection, the median (Min–Max) MICs for caspofungin was 0.06 (0.015–0.25) mg/L, micafungin was 0.015 (0.008–0.06) mg/L and anidulafungin was 0.06 (0.015–0.12) mg/L. For caspofungin, five isolates had MIC of 0.25 mg/L, eleven isolates had MIC of 0.125 mg/L, fifteen isolates hadMIC of 0.06 mg/L, four isolates had MIC of 0.03 mg/L and one isolate had MIC0.015 mg/L. In *FKS1* non-synonymous mutation D632H was observed in one isolate with caspofungin MIC0.25 mg/L. Synonymous mutation at position A2226T was observed in 75% of the isolates.35 (97.2%) isolates analyzed for *FKS 2* gene were observed as wild-type. A novel mutation at I661T was observed *FKS2*-gene in one isolate with caspofungin MIC of 0.12 mg/L and anidulafungin and micafungin MIC of 0.06 mg/L and 0.015 mg/L respectively. Novel *FKS2* synonymous mutations at position A1941T, A1956G and T2119C were observed in 44%, 69% and 63% isolates respectively.

**Conclusions:** Low frequencies of both non-synonymous and synonymous polymorphisms were observed in invasive *C. glabrata* strains in this study. S663P in *FKS2*-genehas been reported to be associated with caspofungin resistance and mutation at 661 codonin our strains needs genomic surveillance and correlation with treatment outcome data.

## P330 Challenging the Novel Fungal Pathogen *Candida auris* via STIMULAN^®^ Antifungal-Loaded Calcium Sulfate Beads—An In-Vitro Analysis

**Mark Butcher ^1^, Jason L. Brown ^1^, Rebecca Wilson-van Ohs ^2^, Donald Hansom ^3^, Craig Delury ^2^, Phillip A. Laycock ^2^ and Gordon Ramage ^1^**

^1^ University of Glasgow
^2^ Biocomposites
^3^ Forth Valley Royal Hospital

**Objectives:** *Candida auris* has emerged as a fungal pathogen of considerable nosocomial concern. The pattern of antifungal resistance displayed by *Candida* auris, as well as other fungal species, cannot be ignored. We have recently published evidence on the *in-vitro* efficacy of antibiotic loaded, fully absorbable calcium sulfate * (CS) beads containing the antifungal agents Caspofungin (CSP), Amphotericin B (AMB) and Fluconazole (FLZ) against a panel of medically relevant fungal organisms as a potential nexus for localised infection control.

We developed an in-vitro model where CS beads were mixed with AMB, CSP, and FLZ, to investigate their in-vitro efficacy when introduced to clinically relevant fungi in planktonic and sessile states. Additionally, we employed a novel hydrogel model to investigate CS efficacy in a wound proxy setting. Finally, we aimed to investigate the inflammatory profile of *Candida auris* and CS beads when in co-culture with epithelial tissue.

**Materials & Methods:** A panel of fungi were selected for broth microdilution testing. Antifungal-loaded CS beads were then introduced to *in-vitro* fungal biofilms to assess biofilm formation and cell viability through a combination of Crystal Violet and XTT assays.

Inoculation of a hydrogel substrate, packed with antifungal-loaded CS beads, was used to assess diffusion through a semi-dry material, to mimic active infection in-vitro. This was assessed via Q-PCR and electron microscopy.

Finally, to investigate the inflammatory profile of CS beads and *Candida auris*, EpiSkin^®^ reconstructed human epithelium was co-cultured alongside *Candida auris* and assessed via RT^2^ PCR profiler array.

**Results:** Planktonic inhibition remained consistent over 7-days. AMB and CSP-loaded beads reduced biomass and inhibited cell metabolism, whilst CS beads containing FLZ displayed a fungistatic effect. This was confirmed by SEM imagery (Figure 1).

A similar trend followed in assessment of hydrogels, with a reduction in CFU when comparing CS beads loaded with CSP and AMB.


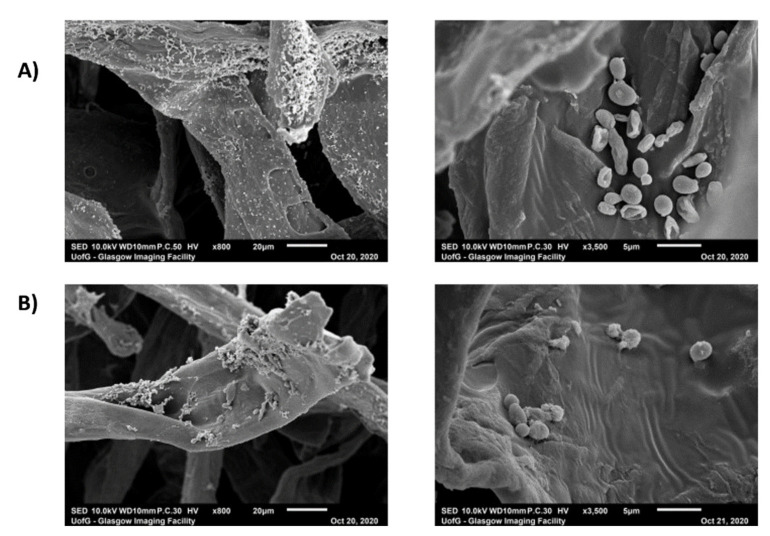


**Figure 1.** Scanning Electrion Micrograph of *Candida auris* grown on hydrogel cellulose matrix. *C. auris* grown for 48 h atop cellulose matrix in hydrogel system and incubated with (**A**) AMB-loaded CS beads and (**B**) CSP-loaded CS beads. Images were taken at 800× and 3500× magnification (left to right). Bars are provided for scale. Notable cell wall deformities are observable in treated cells.

Finally, gene expression analysis of EpiSkin^®^ co-culture revealed that the inflammatory profile differed in cells exposed to both *C. auris* and antifungal-loadedCS beads.

**Conclusions:** Our results have shown that antifungal-loaded CS beads produce a sustained antimicrobial effect over 7 days, which inhibits planktonic and sessile cells of clinically relevant fungal species in-vitro. Moreover, we have provided evidence of an inhibition in the novel fungal pathogen *C. auris*. * STIMULAN^®^ Rapid Cure, Biocomposites LTD.

## P331 Identification of Potential Inhibitors of *Rhizopus* Species in the Pandemic Box Collection

**Mariana Ingrid Dutra da Silva Xisto ^1^, Rodrigo Rollin-Pinheiro ^1^, Luana Pereira Borba-Santos ^2^, Sonia Rozental ^2^ and Eliana Barreto-Bergter ^1^**

^1^ Departamento de Microbiologia Geral, Instituto de Microbiologia Paulo de Góes, Universidade Federal do Rio de Janeiro, 21941-902
^2^ Programa de Biologia Celular e Parasitologia, Instituto de Biofísica Carlos Chagas Filho, Universidade Federal do Rio de Janeiro, 21941-902

**Objectives:** Mucormycosis is a worldwide invasive fungal infection, with high mortality rates due to its aggressive nature, difficulties in diagnosis and high intrinsic resistance to many antifungal drugs such as amphotericin B, posaconazole and isavuconazole. Mucorales species, such as *Rhizopus* and *Mucor*, cause wide spectrum infections in humans, affecting especially the sinuses or lungs of immunocompromised patients or the skin through trauma or burns. After the initial infection, the disease progresses quickly, with angioinvasion that leads to thrombosis and tissue necrosis. The main risk factors are immunosuppression, untreated diabetes and iron overload. Mucormycosis has gained interest due to the increased incidence of immunosuppressed patients, since mucormycosis is the third most common invasive fungal infection in patients with malignant and transplanted hematological tumors. Few antifungal drugs are effective in the treatment of mucormycosis and the identification of new antifungal drugs for the treatment of this infection is extremely important. The aim of this study was to identify compounds with in vitro antifungal activity in the Pandemic Box^®^ compound collection, developed by Medicines for Malaria Venture (MMV, Switzerland, http://www.pathogenbox.org/), against three *Rhizopus* species.

**Materials & Methods:** An initial screening of the 400 compounds (5 µM) in this collection was performed with the *Rhizopus oryzae*, according to the EUCAST protocol. Compounds with activity against *R. oryzae* have also been tested against other mucormycosis etiological agents (*Rhizopus microsporus* e *Rhizopus stolonifer*) at concentrations ranging from 0.08 µM to 20 µM.

**Results:** Seven compounds inhibited more than 50% of the *R. oryzae* growth: MMV396785 (Alexidine), MMV1580844, MMV1581558, MMV642550, MMV098836, MMV1580502 and MMV019724. The MMV1580844 compound showed the lowest MIC values (0.08 µM) but did not show fungicidal activity. Alexidine presented MIC values between 0.63 µM and 1.25 µM and fungicidal activity at 10 µM for the three *Rhizopus* species tested. The compounds with lower MIC values or fungicidal activity were selected to analyze their influence on the biofilm formation by these three *Rhizopus* species. MMV396785 (Alexidine), MMV1580844 and MMV019724 were able to inhibit about 70–95% of biofilm formation in the three species tested.

**Conclusions:** Our results indicate that Alexidine, MMV1580844 and MMV019724 are promising compounds for the development of new antifungal agents.

## P332 Diphenyl Diselenide on the Treatment of Experimental Sporotrichosis by *Sporothrix brasiliensis*

**Livia Munhoz, Vanice Poester, Jéssica Benelli, Aryse Melo and Melissa Orzechowski Xavier**

Universidade Federal Do Rio Grande

**Objectives:** Zoonotic sporotrichosis caused by *Sporothrix brasiliensis* is an epidemic disease in Brazil. Given that difficulties associated with the classical treatment of sporotrichosis are considered a limitation to an effective control of the disease, search for alternative therapies is necessary. Diphenyl diselenide (DD) shows a promising in vitro activity against *S. brasiliensis*. Therefore, we aimed to evaluate the activity of DD in a murine model of sporotrichosis by *S. brasiliensis*.

**Materials & Methods:** 50 Balb/c mice, male, five to seven-week-old, were subcutaneous infected (left footpad) by a clinical isolate of *S. brasiliensis* (5.4 × 10^7^ yeast/mice). Treatments started seven days post-inoculation (p.i) in all five groups (10 mice/each): G1 control group (placebo—receiving just the diluent of the drugs), G2 treatment group (itraconazole—ITZ 50 mg/kg); G3 to G5 DD treatment groups, 1, 5 and 10 mg/kg, respectively. All mice were treated once a day by oral gavage, for 30 consecutive days. Physical conditions and behavior were daily evaluated, and individual weight was weekly determined. At the 32nd day p.i. (48 h after the end of the treatments) animals were euthanasied and lung, liver, brain, kidneys, testicle and footpad were recovered aseptically and homogenized to mycological analysis. Fungal burden in each organ was measured by quantitatively counting colony-forming units (CFU). The determination of colony forming units (CFU)/gram of tissue (g) of *S. brasiliensis* in internal organs of groups were compared by non-parametric tests using Kruskal-Wallis combined with Wilcoxon. The weight loss assay was calculated considering the percentage of loss and/or increase between the value corresponding to the fungal inoculation day and to the end of the treatment.

**Results:** All animals progressed to a disseminated form of sporotrichosis, independently of the treatment, confirmed by macroscopic lesions on internal organs associated with *S. brasiliensis* isolation from them. Despite that, animals treated with ITZ (G2) had a lower fungal burden from internal organs in comparison to the placebo group (G1) (*p* < 0.01). On the other hand, in disagreement to our hypothesis, animals treated with DD (1, 5 or 10 mg/kg) (G3 to G5) had higher fungal burdens on internal organs when compared to the placebo group (*p* < 0.05). In addition, animals treated with DD 5 mg/kg (G4) had a statistically higher rate of weight loss during the treatment in comparison to the control group (G1).

**Conclusions:** These partial results suggest that DD, at the dosages tested and associated with an experimental infection of *S. brasiliensis* instead to play a protective role in the development of the disease, clinically worsened the infection, indicating a possible toxicity. Since DD is a janus-faced compound, further studies with an adjustment on the inoculum size to avoid the severe disseminated sporotrichosis and with lower dosages of this compound to treat this mycosis are necessary to evaluate its potential therapeutic characteristics.

**Financial support:** Research Support Foundation of Rio Grande do Sul (FAPERGS).

## P333 In Vitro Activity of Citral in Combination with Anidulafungin, Amphotericin B, Fluconazole and Isavuconazole against *Candida auris*

**Iñigo de la Fuente, Anna Batllé, Elena Sevillano, Katherine Miranda, Elena Eraso, Guillermo Quindós and Andrea Guridi**

University Of the Basque Country Upv/ehu

**Objective:** Candidiasis are the most common opportunistic mycoses. In recent years, the global emergence of *Candida auris* has become particularly relevant. This species is an emerging pathogen that causes hospital outbreaks of invasive candidiasis. In addition, *C. auris* can persist in the hospital environment for prolonged periods, being a constant source of infection. Treatment of invasive candidiasis caused by *C. auris* is a major clinical challenge due to its multidrug-resistant pattern. This problem implies the search for novel therapies to treat these diseases, so the aim of this work was to evaluate the in vitro interaction between citral and anidulafungin, amphotericin B, fluconazole and isavuconazole against clinical isolates of *C. auris*.

**Methods:** We studied 22 *C. auris* isolates obtained from different clinical samples from the Hospital La Fe, Valencia, Spain (eight from blood culture, seven from oropharynx and seven from urine cultures). *Candida parapsilosis* ATCC 22019 and *Candida krusei* ATCC 6258 were used as quality controls. The minimum inhibitory concentration (MIC) was determined according to the European Committee on Antibiotic Susceptibility Testing (EUCAST) EDef 7.3.1 document. The interaction between citral and the rest of antifungal drugs was assessed by a checkerboard microdilution method. The final concentration of anidulafungin, amphotericin B and isavuconazole tested ranged from 0.008 to 4 μg/mL; fluconazole from 1 to 64 μg/mL and citral from 16 to 1024 μg/mL. The in vitro interaction of the drug combinations was interpreted in terms of the fractional inhibitory concentration index (FICI) as follows: FICI < 1, synergistic; FICI equal to 1, additive; and FICI > 1, indifferent or antagonistic.

**Results:** The combination of citral with anidulafungin significantly reduced the MIC of citral against *C. auris* isolates. The MIC of citral decreased from > 1024 μg/mL to 3–128 μg/mL against 21 of the 22 isolates included and the MIC of anidulafungin decreased from 0.125 to 0.016 μg/mL. The combination of citral with amphotericin B had the same efficacy as the combination with anidulafungin and the MIC from amphotericin B decreased from 0.25 to 0.016 μg/mL. The effect of the combination of citral with fluconazole against three of the isolates studied was interpreted as synergistic and against eight as additive. Furthermore, the effect of the combination of citral with isavuconazole against seven of the isolates was interpreted as synergistic and against seven others as additive.

**Conclusions:** The combination of citral with amphotericin B, anidulafungin, fluconazole or isavuconazole reduced MICs of both antifungal drugs and citral against *C. auris*.

**Funding:** GIC15/78 IT-990-16 (Gobierno Vasco- Eusko Jaurlaritza).

## P334 In Vitro Study about the Antifungal Activity of Peptides Derived from Human Cathelicidin LL-37, against Different Strains of *Candida* spp.

**Julián E. Muñoz ^1^, Wendy D. Mejía ^2^, Karem J. Guzmán ^2^, Gladys Pinilla ^2^ and Claudia Andrea Cruz ^2^**

^1^ School of Medicine and Health Sciences, Universidad del Rosario
^2^ REMA Research Group, Faculty of Health Sciences, Colegio Mayor de Cundinamarca University

**Objectives:** To verify the antifungal effect of analogous peptides derived from LL-37, against yeasts of the genus *Candida*, specifically clinical isolates from patients with recurrent vulvovaginal candidiasis (RVVC) and reference strains. Additionally, to analyze the antifungal effect and possible synergism of the LL-37 analogous peptides associated with fluconazole.

**Materials & Methods:** The antifungal effect of four analogous peptides to LL-37 (LL-37-1, D, AC1, AC2) and their synergistic effect associated with fluconazole was analyzed in 15 clinical isolates from patients with RVVC and in reference strains of *C. parapsilosis* ATCC 22019, *C. krusei* ATCC 6558 and *C. albicans* ATCC 10232, through the determination of the minimum inhibitory concentration (MIC) using the broth microdilution assay according to the CLSI protocol (M27-S4, 2012). As an inhibition control, the antifungal fluconazole commonly used in the treatment against candidiasis was used. Through the use of transmission electron microscopy, the possible structural damage of *Candida albicans* ATCC 10232 yeasts exposed to the four LL-37 analogous peptide treatments compared with the untreated control group was verified.

**Results:** The four peptides derived from human cathelicidin LL-37 showed antifungal profiles against the strains of *Candida* spp. studied. Synergistic effect of peptide + fluconazole (blue line) was outstanding in the significant control of yeast growth as shown in Figure 1, where the MIC of the analogous peptides of LL-37 vs. the reference strain *Candida albicans* ATCC 10232 is observed. Transmission electron microscopy indicated plasma membrane damage and genetic material condensation, which confirms the antifungal effect displayed by the peptides derived from LL-37.

**Figure 1.** Minimal Inhibitory Concentration (MIC) of LL-37 analogous peptides and fluconazole profiles in *C. albicans* ATCC 10231. (**A**) Effect of LL-37-1 peptide (red line), associated with fluconazole (blue line) and fluconazole alone (black line). (**B**) Response to the LL37-D peptide (red line), associated with fluconazole (blue line) and only fluconazole (black line). (**C**) Effect of AC-1 peptide (red line), associated with fluconazole (blue line) and fluconazole alone (black line). (**D**) Response to the AC-2 peptide (red line), associated with fluconazole (blue line) and only fluconazole (black line). Upper dotted line represents the positive control without any treatment.

**Conclusions:** The analogous peptides of LL-37 herein studied, showed a promising anti-*Candida* effect and an outstanding synergistic effect when were associated with fluconazole. Variations at the peptide chemical synthesis level derived from LL-37 have generated shorter peptide sequences and probably more resistance to the effect of proteases secreted by some microorganisms. Rational design of new peptides has improved their antimicrobial activity, that why these molecules reflect an important therapeutic alternative in the treatment of infections caused by *Candida* species.

## P335 Rezafungin Safety and Pharmacokinetics in Subjects with Moderate or Severe Hepatic Impairment

**Shawn Flanagan ^1^, Voon Ong ^1^, Taylor Sandison ^1^, Rebeca Melara ^2^, Thomas Marbury ^3^, Alena Jandourek ^1^ and Jade Huguet ^2^**

^1^ Cidara Therapeutics
^2^ Altasciences
^3^ Orlando Clinical Research Center

**Objectives:** Rezafungin is a novel echinocandin antifungal being developed for treatment of candidemia/invasive candidiasis, and for prevention of invasive fungal disease caused by *Candida* and *Aspergillus* spp. and Pneumocystis jiroveciiamongimmunosuppressed patients. This study was designed to assess the safetyand pharmacokinetics (PK)of rezafungin in subects with normal hepatic function and subiects with moderate or severe hepatic impairment as clinical trials are limited to enrolling patients with moderate HI due to the comparator caspofungin’s product labeling.

**Materials &Methods:** The safety, tolerability, and PK of rezafungin in subjects with moderate (Group 1a) and severe (Group 2a) HI (Child-Pugh class Band C) and healthysubjects (HS, Groups 1b and 2b) was investigated in this open-label, single-dose study. Each Hl group enrolled was matched to a separate HS group (n = 8 each 32 total) by age, sex and body mass index (BMI) and received a single 400-mg intravenous 1 h infusion of RZF.Plasma PK sampling was performed at various time points through 336 h post- dose. Rezafungin PKparameters were derived using non-compartmental analysis. Safety and tolerability were assessed throughout the study.

**Results:** Subjects with moderate or severe HI were well matched to their respective heathy subject groups. The mean age and BMl of moderate HI and matched HS groups were 57.0 and 56.9 years, and 32.0 and 31.1 kg/m respectively with 4 males and 4 females per group. The mean age and BMl of severe Hl and matched HS groups were 58.0 and 56.6 years, and 29.7 and 29.7 kg/m^2^ respectively, with 6 males and 2 females per group.

Mean RZF exposure in subjects with moderate or severe HI was up to ~30% lower than that in HS with considerable overlap between individuals (AUC Figure 1. Cmx Figure 2). These differences were not considered to be clinically relevant. Mean half-life values were similar between groups and ranged from 110 to 124 h.

There were 9 AEs in 7 subjects: 3 occurred in the severe hepatic impairment group (bronchitis, worsening hepatic encephalopathy, hyponatremia) all of moderate severity and unrelated toRZF; 5 occurred in the moderate hepatic impairment group (nausea, headache, infusion site infiltration, body aches, productive cough) all of mild severity and only 1AE was considered related to RZF (headache); 1AE occurred in the healthy group (infusion site infiltration) of mild severity was unrelated. All adverse events resolved or were resolving at the end of the study.

**Conclusions:** Rezafungin exposure was modestly reduced on average in subjects with moderate or severe hepatic impairment relative to matched healthy subjects, with considerable overlap between individuals. There were no significant safety findings associated with rezafungin in any group. More adverse events were experienced by subjects with moderate or severe HI than HS, as expected given their underlying liver disease. These results indicate that rezafungin can be administered to subjects with all levels of hepatic impairment without dose adjustment.

## P336 Oral Ibrexafungerp for Treatment of Patients with Refractory Mycoses: Interim Analysis of Outcomes from a Phase 3 Open-Label Study (FURI)

**Oliver Cornely ^1^, Philipp Koehler ^1^, John Walton Sanders ^2^, Caryn Gee Morse ^2^, Luis Ostrosky Zeichner ^3^, Rachel Miller ^4^, Riina Rautemaa-Richardson ^5^, Rohit Bazaz ^5^, Thomas Walsh ^6^, Jose Vazquez ^7^, Andrej Spec ^8^, Peter Pappas ^9^, Todd McCarty ^9^, George R. Thompson ^10^, George M. Lyon ^11^, Marisa Miceli ^12^, Thomas Patterson ^13^, Martin Hoenigl ^14^, Nkechi Azie ^15^ and David Angulo ^15^**

^1^ University of Cologne
^2^ Wake Forest University
^3^ University of Texas at Houston
^4^ Duke University
^5^ University of Manchester
^6^ Cornell University
^7^ Augusta University
^8^ Washington University at St. Louis
^9^ University of Alabama
^10^ University of California at Davis
^11^ Emory University
^12^ University of Michigan
^13^ University of Texas Health Science Center
^14^ University of California at San Diego
^15^ SCYNEXIS, Inc.

**Objectives:** There are limited oral treatment options available for patients with fungal infections who fail currently available antifungals or who have an infection caused by resistant organisms. Ibrexafungerp is an investigational broad-spectrum glucan synthase inhibitor antifungal with activity against *Candida* and *Aspergillus* species, including azole- and echinocandin-resistant strains. A Phase 3 open-label, single-arm study of ibrexafungerp (FURI; NCT03059992) is ongoing for the treatment of patients intolerant of or with fungal disease refractory to standard antifungal therapy. We present a subset of patients from the FURI study who were refractory to currently available therapy as determined by the investigator.

**Methods:** FURI patients were eligible for enrollment if they have proven or probable, severe mucocutaneous candidiasis, invasive candidiasis or invasive aspergillosis and documented evidence of failure, intolerance, or toxicity related to a currently approved standard-of-care antifungal treatment or could not receive approved oral antifungal options (e.g., susceptibility of the organism) and continued IV antifungal therapy was clinically undesirable or unfeasible.

**Results:** There were 74 patients enrolled in the FURI study from 22 centers in US, UK and EU treated with ibrexafungerp for various fungal infections from 2016–2020. A total of 44 patients were enrolled due to the refractory nature of their disease, including failure of or resistant to previous antifungal therapy. The predominant fungal disease diagnoses at baseline included, intraabdominal candidiasis, bone/joint candidiasis, oropharyngeal candidiasis, esophageal candidiasis, vulvovaginal candidiasis, other *Candida* infections invasive and pulmonary aspergillosis. Table 1 shows outcomes for refractory patients as determined by the DRC. The percent of refractory patients who had a complete response (CR), partial response (PR), clinical improvement (CI) was 59.1%, stable disease (SD) was 22.7%, and patients with progression of disease 6.8%. Four patients were indeterminate, and one patient died of causes unrelated to their fungal disease.

**Conclusions:** Analysis of 44 refractory patients from the FURI study indicates that oral ibrexafungerp provides a favorable therapeutic response in patients who have failed other therapies with difficult to treat fungal infections.

## P337 Outcomes of Oral Ibrexafungerp by Pathogen from Two Open-Label Studies of Patients with Serious Fungal Infections (FURI and CARES)

**Riina Rautemaa-Richardson ^1^, Rohit Bazaz ^1^, Oliver A. Cornely ^2^, Philipp Koehler ^2^, Deven Juneja ^3^, Peter G. Pappas ^4^, Todd McCarty ^4^, Rachel Miller ^5^, Jose Vazquez ^6^, John Walton Sanders ^7^, Caryn Gee Morse ^7^, Luis Ostrosky-Zeichner ^8^, Robert Krause ^9^, Juergen Prattes ^9^, Andrej Spec ^10^, Thomas J. Walsh ^11^, Francisco Marty ^12^, Isabel H. Gonzalez-Bocco ^12^, Marisa H. Miceli ^13^, Thomas F. Patterson^14^, Martin Hoenigl ^15^, Nkechi E. Azie ^16^ and David A. Angulo ^16^**

^1^ University of Manchester
^2^ University of Cologne
^3^ Max Super Specialty Hospital
^4^ University Alabama
^5^ Duke University
^6^ Augusta University
^7^ Wake Forest University
^8^ University of Texas
^9^ Medical University at Graz
^10^ Washington University at St. Louis
^11^ Cornell University
^12^ Brigham and Women’s Hospital
^13^ University of Michigan
^14^ University of Texas Health Science Center
^15^ University of California
^16^ SCYNEXIS, Inc.

**Objectives:** *Candida* and *Aspergillus* infections resistant to currently available antifungals are an emerging global threat. Ibrexafungerp is an investigational broad-spectrum glucan synthase inhibitor antifungal with activity against *Candida* and *Aspergillus* species, including azole- and echinocandin-resistant strains. Two ongoing Phase 3 open-label, single-arm studies of oral ibrexafungerp for the treatment of patients (>18 years), bv FURI (Clinicaltrials.gov NCT03059992) with fungal diseases who are refractory to or intolerant of standard antifungal therapies and CARES (Clinicaltrials.gov NCT03363841) for adult patients with *Candida auris* infections.

**Methods:** FURI subjects were eligible for enrollment if they had proven or probable severe mucocutaneous candidiasis, invasive candidiasis, invasive aspergillosis, or other fungal diseases with evidence of treatment failure, intolerance, or toxicity related to a currently approved standard-of-care antifungal treatment or if they were unable to receive an approved oral antifungal option (e.g., susceptibility of the organism) and a continued IV antifungal therapy was clinically undesirable or unfeasible. CARES patients were eligible for enrollment if they had a documented *Candida auris* infection.

**Results:** There were 74 patients enrolled in the FURI study from 22 centers in US, UK and EU and 10 patients were enrolled in the CARES study from 4 centers in South Africa and India for a total of 84 patients treated with ibrexafungerp for invasive and mucocutaneous fungal infections. In the two studies the predominant fungal disease diagnoses at baseline included, candidemia, intraabdominal candidiasis, bone/joint candidiasis, oropharyngeal candidiasis, esophageal candidiasis, vulvovaginal candidiasis, other *Candida* infections and invasive pulmonary aspergillosis. Table 1 shows outcomes by pathogens as determined by the DRC from both studies. Combining outcomes from the two studies, the percent of patients who were determined to have a complete response (CR), partial response (PR) and clinical improvement (CI) was 65.1%; stable disease (SD) was 27.1%; patients with progression of disease 5.9%; and 4 patients were indeterminate. Additionally, there was 1 death in the CARES study; 1 patient with a pathogen not identified and 1 death in the FURI study. The death was determined to be not related to fungal disease.

**Conclusions:** Preliminary analysis of outcomes by pathogen for these 84 cases from the FURI and CARES studies indicate that oral ibrexafungerp provides a favorable and similar therapeutic response in patients with serious fungal infections caused by *Candida*, regardless of species or resistance profile.

## P338 Evaluation of the Post-Antifungal Effect of Rezafungin and Micafungin against *Candida albicans, Candida parapsilosis* and *Candida glabrata*

**Cecilia G. Carvalhaes ^1^, Jennifer D. Thompson ^1^, Paul R. Rhomberg ^1^, Jeffrey B. Locke ^2^ and Mariana Castanheira ^1^**

^1^ JMI Laboratories
^2^ Cidara Therapeutics

**Objectives:** Post-antifungal effect (PAFE) is defined as the growth suppression of fungal cells after exposure to an antifungal agent. Understanding PAFE is useful to evaluating dosage regimens of an antifungal agent. Rezafungin is a new echinocandin with extended half-life exhibiting activity against *Candida* spp. *Aspergillus* spp. and *Pneumocystis* spp. In this study, we evaluated the PAFE of rezafungin compared to that of micafungin against Candidaalbicans (CA), *C. glabrata* (CGLA), and *C. parapsilosis* (CPRP) isolates.

**Materials &Methods:** Six *Candida* spp. Isolates (CA ATCC90028CPRP ATCC22019, and 4 clinical isolates) were susceptibillity tested (CLS1 M27) in triplicate to establish baseline MIC (bMIC) values. For PAFE determinations, antifungal concentrations of 1×, 4×, and 16× bMiC were used. A starting inoculum of 1–5 × 10 CFU/mLwas added to RPMl with the antifungal agent. Post 1 h exposure, cells were washed 3 times and resuspended in 10 mL of pre-warmed RPMl. Colony counts were performed at to, 1 h before and after final washt t2, t4, t8, t12, t24 and t48 h. PAFE was calculated as the difference in time required for isolates to rrow1-logio following the final cell wash compared to the growth control.

**Results:** ThebMiCresults are shown in the Table. Rezafungin and micafungin PAFEs were >14.9 h against the CA clinical isolate for all concentrations. Rezafungin PAFE results were >40 h for both CGLA strains, regardless of the concentration. These results were equivalent to micafungin PAFE values (>40 h) for CGLA isolates at all concentrations, except the 1× bMic for the CGLA #3 isolate when the micafungin PAFE value was 20.4 h compared to the rezafungin PAFE of >46.7 h. Rezafungin PAFE results were also >40 h against the CPRP ATCC 22019 strain at all concentrations. In contrast, no micafungin PAFE was observed against CPRP ATCC22019 at 1× and 4× bMiC and a short PAFE (1.8 h was noted at 16× bMiC.The CPRP clinical isolate displayed prolonged rezafungin PAFE values (range, 18.4 h > 36.6 h) regardless of the concentration tested; however, a short PAFE was displayed by micafunginat 1× (1.6 h) and 4× (7.4 h) bMiCagainst this CPRP clinical isolate. A PAFE of 31.3 h was noted for micafungin against the CPRP clinical isolate at 16× bMiC.The CA ATCC 90028 control failed to re-grow over1-log10; the rezafunginand micafungin PAFEs could not be determined against this isolate.

**Conclusions:** Differences in PAFE against Candida species can be useful to consider when evaluating antifungal agent dosing. Rezafungin showed sustained activity following drug removal, and displayed equivalent or longer PAFE results than micafungin against all tested *Candida* spp.

## P339 Novel Antifungal Peptide NP339 Inhibits *A. fumigatus* Growth in Whole Human Blood

**Daniel Smith, Emma Lovie, Laura Simpson and Deborah O’Neil**

NovaBiotics Ltd.

**Objectives:** NP339 is a broad-spectrum antifungal peptide with a novel mechanism of action. To assess its potential as a systemically administered therapy for invasive fungal disease under physiologically relevant conditions, we developed a robust ex vivo assay employing whole human blood (WHB). To improve the utility and clinical relevance of broth microdilution antimicrobial susceptibility testing (AST), particularly for non-traditional compounds such as NP339, biological matrices including blood and plasma can and should be employed to better predict clinical activity, stability and other key drug properties. Here we demonstrate that NP339 inhibits *A. fumigatus* growth in WHB and importantly, at doses lower than those suggested by standard AST which utilise RMPI and other cell culture media.

**Methods:** The antifungal activity of NP339 was investigated using an ex vivo model of *A. fumigatus* infection. Citrated WHB was provided by the Scottish National Blood Transfusion Service (SNBTS) following Ethical approval by SNBTS Committee For The Governance Of Blood And Tissue Samples For Non-Therapeutic Use, And Donor Research. WHB was inoculated with conidia from three *A. fumigatus* strains (AM 2002/0066, AF293 & A1163) to a final concentration 1 × 10^5^ conidia/mL. Infected blood samples were treated with NP339, control antifungal, or PBS vehicle for final ratio of 85% WHB, 10% inoculum, 5% treatment. Samples were incubated at 37 °C for 24 h and antifungal efficacy was assessed in three ways (1) Galactomannan antigen concentration in the plasma fraction as judged by Platelia™ GM ELISA. (2) *A. fumigatus* burden was quantified by quantitative real-time PCR (qPCR). (3) Fungal burden was visualised by fluorescence microscopy utilising calcofluor white (CFW) staining.

**Results:** NP339 is more potent in clinically relevant WHB compared to standard AST conditions and RPMI. By GM ELISA, the minimum effective concentration of NP339 against *A. fumigatus* in WHB was observed as being 10-fold less than that predicted by standard AST methodologies. Further interrogation of the WHB ex vivo model by molecular analysis demonstrated a statistically significant reduction in *A. fumigatus* burden by qPCR analysis following NP339 treatment. Finally, the microscopy demonstrates the dramatic inhibition if hyphal growth in WHB following NP339 treatment.

**Conclusions:** In this series of experiments, NP339 is shown to possess anti—*A. fumigatus* activity in a clinically relevant matrix. This activity is underappreciated—and may even be overlooked/dismissed—if standard AST methodologies were followed and highlights the requirement to challenge efficacy of putative therapeutic candidates in clinically predictive models. Our data support the continued development of NP339 as a much-needed novel antifungal candidate, but importantly, the WHB ex vivo assay described employing ELISA and molecular read-outs can be expanded to other fungi (for NP339) and other antifungal agents.

## P340 Anticandidal Activity of Jelleine-II and Derivatives

**Aitzol Perez Rodriguez, Elena Eraso, Eneko Largo, Guillermo Quindós and Estibaliz Mateo**

Department of Immunology, Microbiology and Parasitology, Faculty of Medicine and Nursery, University of the Basque Country (UPV/EHU)

**Objective:** Jelleine-II, present in the royal jelly of the honeybee *Apis mellifera*, is a nine aminoacid peptide with antimicrobial activity. The objective of this study was to evaluate the in vitro activity of the jelleine-II peptide and several derivatives against different species of *Candida*.

**Materials & Methods:** The jelleine-II peptide and 13 derivatives, nine of which were designed following the alanine scanning technique, were synthesized and obtained from Proteogenix (France). The alanine scanning technique consists on changing each aminoacid of the original peptide for an alanine, with the purpose of determining the relative importance of the individual residues for the anticandidal activity in this case.

A total of 14 *Candida* clinical isolates with different fluconazole susceptibility and belonging to five different species were tested: three isolates of each *Candida albicans, Candida auris, Candida glabrata*, and *Candida parapsilosis* and other two of *Candida krusei*.

The minimal inhibitory concentrations (MIC) and minimal fungicidal concentrations (MFC) were determined by broth microdilution method following the guidelines of the EUCAST definitive document 7.3.2. Final concentrations of the peptides ranged from 8 to 128 µM. MIC values were determined by optical density at 530 nm as the lowest concentration at which the growth of the yeast resulted to be 50% or lower in comparison to the growth of the positive control. Moreover, MFCs were obtained by colony counting in Sabouraud-dextrose agar and were defined as the lowest concentration of antimicrobial peptide causing the death of 99.9% of the inoculum. The experiments were performed at least in three separate times.

**Results:** The original peptide and three of its derivatives displayed activity against all tested *Candida* isolates at 24 h with a concentration ≥ 32 µM. The improvement in the efficacy was observed as a reduction of the concentration of peptide needed to exert the candidacidal activity for each isolate. The replacement of the threonine in the first position of the original peptide for an alanine improved the anticandidal activity, but not as much as changing it for an arginine. Exchanging the histidine in the eigth position for an arginine also improved the activity against *Candida*.

Moreover, the derivate obtained with arginine in the first and eigth positions was able of inhibiting the growth of the 92.8% (13/14) of *Candida* isolates when a concentration of ≥ 32 µM was applied.

The effect of the rest of the assayed peptides were also dose and strain dependent but was not close to the effect achieved by the aforementioned five most active peptides.

**Conclusions:** Jelleine-II and some of its derivatives exert candidacidal activities against *C. albicans, C. parapsilosis, C. auris, C. glabrata* and *C. krusei*.

**Funding:** GIC15/78 IT-990-16 (Gobierno Vasco-Eusko Jaurlaritza). Aitzol Perez-Rodriguez was funded by Ph.D. grants from UPV/EHU (PIF17/167).

## P341 Identifying and Assaying New Molecular Targets in Fungi Following a Novel Strategy Based on Binding Site (Dis)Similarities with Human Targets

**Marcela Rubio-Carrasquilla ^1,2^, Johann E. Bedoya-Cardona ^1^, Mario S. Valdés-Tresanco ^1^, Iliana M. Ramírez-Velásquez ^3^ and Ernesto Moreno ^1^**

^1^ University of Medellin
^2^ Corporation for Biological Research (CIB)
^3^ Instituto Tecnológico Metropolitano

**Objectives:** Invasive fungal infections account for a high burden of morbidity and mortality. This is aggravated because of the toxicity and resistance problems associated to current antifungal drugs, which in whole target only a handful of fungal molecules. In this scenario, new target identification and drug design, together with drug repurposing, represent promising strategies. We aim to identify and test in vitro potential new therapeutic targets in fungi.

**Materials & Methods:** Our strategy consists in identifying fungal proteins with active sites (meaning the set of residues lining the binding pocket) that are similar, but not identical!, to sites of proteins from the human pharmacolome. A high structural similarity with a human counterpart allows validation of the fungal target using cross-reactive inhibitors of the human protein. On the other hand, a few amino acid differences in the binding pocket produce local topological and chemical changes that create a “design space” for new specific inhibitors of the fungal target.

**Results:** Applying our own bioinformatics approach and taking advantage of the >200 available crystal structures of proteins of the human pharmacolome in complex with inhibitors, we have identified ca. 30 proteins in several fungal species of the genera Histoplasma, Candida, Criptococcus, Aspergillus and Fusarium, whose binding sites share at least 70% amino acid identity with their similar binding pockets in human pharmaceutical targets. So far we have assayed in vitro, in seven different fungal species, ca. 60 known inhibitors of around twenty of the orthologous human proteins. Some of the tested inhibitors have been previously assayed in different species in drug repurposing screenings, while others, to our knowledge, have not been yet tested. Over a dozen of these compounds, targeting eight different protein targets, showed IC50 values in the micromolar order in one or across several species. In general, yeasts were more significantly affected than molds.

**Conclusions:** Our results point to new potential fungal targets that can be exploited for the design of new antifungal agents. Ongoing work by our group aims to identify, by virtual screening, specific inhibitors for several of these potential targets.

## P342 Miltefosine against Scedosporium and *Lomentospora* Species: Antifungal Activity and Its Effects on Fungal Cells

**Rodrigo Rollin-Pinheiro ^1^, Yuri de Castro Almeida ^1^, Victor Pereira Rochetti ^1^, Mariana Ingrid Dutra da Silva Xisto ^1^, Luana Borba-Santos ^2^, Sonia Rozental ^2^ and Eliana Barreto-Bergter ^1^**

^1^ Departamento de Microbiologia Geral, Instituto de Microbiologia Paulo de Góes, Universidade Federal Do Rio De Janeiro
^2^ Programa de Biologia Celular e Parasitologia, Instituto de Biofísica Carlos Chagas Filho, Universidade Federal do Rio de Janeiro

**Objectives:** *Scedosporium* and *Lomentospora* species are filamentous fungi responsible for a wide range of infections in humans and are frequently associated with cystic fibrosis and immunocompromising conditions. Since they are usually resistant to many antifungal drugs available in clinical settings, studies of alternative targets in fungal cells and therapeutic approaches are necessary. In the present work, we evaluated the in vitro antifungal activity of miltefosine against *Scedosporium* and *Lomentospora* species and how this phospholipid analog affects the fungal cell.

**Materials & Methods:** Antifungal susceptibility tests and biofilm assay were performed to evaluate the activity of miltefosine against *Scedosporium* and *Lomentospora* species. Electron microscopy and fluorescent dyes, such as 2′,7′ –dichlorofluorescein diacetate and JC-1, were used to check the effects on cell integrity, oxidative stress and mitochondrial membrane potential, respectively. Miltefosine effects on fungal membrane were evaluated by fillipin staining, the susceptibility to membrane and cell wall stressors and the analysis of cell membrane permeability. Sinergystic assay was performed to check miltefosine interaction with current antifungal agents used in clinical settings.

**Results:** Miltefosine inhibited different *Scedosporium* and *Lomentospora* species at 2–4 µg/mL and reduced biofilm formation. The loss of membrane integrity in *S. aurantiacum* caused by miltefosine was demonstrated by leakage of intracellular components and lipid rafts disorganization. Exogenous addition of glucosylceramide decreases the inhibitory activity of miltefosine. ROS production and mitochondrial activity are also affected by miltefosine, as well as susceptibility to fluconazole, caspofungin and myoricin.

**Conclusions:** The data obtained in the present study contribute to clarify the dynamic of interaction of miltefosine with *Scedosporium* and *Lomentospora* cells, highlighting its potential use as new antifungal drug in the future.

## P343 The Mechanism of Nanostructured Lipid Carrier Drug Delivery System against Fluconazole-Resistant *Candida glabrata*: Do ATP-Binding Cassette Transporters Play a Role?

**Maryam Moazeni ^1^, Hamidreza Kelidari ^2^, Mojtaba Nabili ^3^, Behrad Roohi ^4^ and Ali Nokhodchi ^5^**

^1^ Invasive Fungi Research Center, Communicable Diseases Institute, Mazandaran University of Medical Sciences
^2^ Department of Pharmaceutics, Faculty of Pharmacy, Mazandaran University of Medical Sciences
^3^ Faculty of Medicine, Sari Branch, Islamic Azad University
^4^ Student Research Committee Center, Mazandaran University of Medical Sciences
^5^ Pharmaceutics Research Laboratory, School of Life Sciences, University of Sussex, Arundel Building, Brighton BN1 9QJ, UK

**Objectives:** The present study was designed to evaluate the possible role of efflux transporters in converting fluconazole resistance in *Candida glabrata* isolates exposed to fluconazole loaded nano structured lipid carriers (FLZ-NLCs).

**Materials & Methods:** Four fluconazole-resistant *C. glabrata* isolates were used. FLZ-NLCs were prepared using the ultrasound technique. Each fluconazole-resistant *C. glabrata* isolate was exposed to fluconazole (alone and in the form of FLZ-NLCs) and placebo (NLCs without fluconazole (FLZ)) for 20 h at 37 °C and the total RNA extracted. Real-time PCRs were performed to assess the probable alternations in ATP-binding cassette transporter genes.

**Results:** Under the FLZ-NLCs treated condition, no significant alteration was observed in the expression of *CgCDR1, CgCDR2* and *CgSNQ2* genes although the genes were over-expressed when exposed to fluconazole.

**Conclusions:** It is highly suggested that nanostructure lipid carrier may prevent drug detection by transporter’s proteins avoiding the drug pumping out. As such, the developed formulation may be fundamentally beneficial for the treatment of candidiasis caused by *C. glabrata*.

## P344 In Vitro Evaluation of Essential Oils, Fractions and Terpenes against Clinical Isolates of *Candida auris* with Different Antifungal Susceptibility Profiles

**Carolina Zapata-Zapata ^1^, Manuela Loaiza-Oliva ^2^, Juan Carlos Gómez-Velásquez ^3^, Liliana Torcoroma García ^4^, Elena Stashenko ^5^ and Ana Cecilia Mesa-Arango ^1^**

^1^ Group of Investigative Dermatology, University of Antioquia
^2^ Laboratory of Oral Microbiology, Faculty of Dentistry, University of Antioquia
^3^ Synlab S.A.S Laboratory
^4^ Infectious Disease Research Program, University of Santander
^5^ CROM-MASS-CENIVAM, Industrial University of Santander

**Objectives:** To evaluate in vitro essential oils, enriched fractions and terpenes against clinical isolates of *C. auris* with different antifungal susceptibility profiles.

**Materials & Methods:** a total of 28 samples (twelve essential oils (EO) from different plants species collected in Colombia, eight enriched fractions prepared in the lab, and eight commercial terpenes) were evaluated against eight clinical isolates of *C. auris* and against the *C. auris* CDCB11903 strain. Essential oils were isolated from plant material by using hydrodistillation and analyzed by GC/MS, the yeast identification was carried out by means of MALDI-TOF, and antifungal activity was evaluated following broth microdilution reference method CLSI M27-A3. Also, antifungal susceptibility profiles of *C. auris* were obteined for fluconazole (FLZ), amphotericine B (AMB) and itraconazole (ITZ). *Candida krusei* ATCC 6258 and *C. parapsilosis* ATCC 22019 were included as control strains. Initially, samples (28) were evaluated at 256 μg/mL, and for those in which >90% growth inhibition was observed, minimal inhibitory concentrations (MICs) were determinated using concentrations within the 256–16 μg/mL range. The MICs corresponded to the lowest concentration of the sample where a ≥90% reduction of visible fungal growth was observed. The assays were performed in duplicate at two different times. The results were expressed as ranges and geometric means (GM).

**Results:** clinical isolates of *C. auris* showed different antifungal susceptibility profiles. The GM MICs and ranges values were: 0.43 (0.125–2 µg/mL), 0.12 (0.125–1 µg/mL), and 5.26 (1–32 µg/mL), for AMB, ITZ and FLZ, respectively. All isolates were sensitive to four EO (GM-MICs range 207.9–157.6 µg/mL), and one EO was active with seven isolates (GM = 128 µg/mL); however, the enriched fractions were not active against clinical isolates. The activity of terpenes was as follows: D-limonene (GM = 20.39 µg/mL) > thymol (GM = 104 µg/mL) > carvacrol and p-cymene (GM = 215.3 µg/mL).

**Conclusions:** *C. auris* is an emergent yeast that causes invasive infections, mainly in intensive care units. This fungus is resistant or has low sensitivity to the main antifungal agents of clinical use. This fungus forms biofilms, and these adherent communities allow permanence and transmission in hospital environments. Althought chlorine-based products (such as chlorhexidine) are the most widely used as disinfectants; however, colonization often persists. With this in mind, and with the results of this study, it can be said that EO or terpenes, particularly limonene, which was the most active (MICs range 16–128 µg/mL), can be a source for the development of new anti-*C. auris* products for the decolonization of patients, and for the cleaning and disinfection of surfaces or reusable equipment.

**Acknowledgments:** The authors thank funding from the Ministry of Science, Technology and Innovation, the Ministry of Education, the Ministry of Industry, Commerce and Tourism, and ICETEX, Programme Ecosistema Científico-Colombia Científica, from the Francisco José de Caldas Fund, Grant RC-FP44842-212-2018.

## P345 Cytotoxic and Anti-*Candida* spp. Activity of Essential Oils and Terpenes

**Carolina Zapata-Zapata ^1^, Manuela Loaiza-Oliva ^2^, Yaneth Miranda-Brand ^1^, María Cecilia Martínez-Pabón ^2^, Elena Stashenko ^3^ and Ana Cecilia Mesa-Arango ^1^**

^1^ Group of Investigative Dermatology, University of Antioquia
^2^ Laboratory of Oral Microbiology, Faculty of Dentistry, University of Antioquia
^3^ CROM-MASS-CENIVAM, Industrial University of Santander

**Objective:** To determine the cytotoxicity and anti-*Candida* spp. activity of essential oils and terpenes.

**Materials & Methods:** A total of twelve essential oils (EOs) from different plant species collected in Colombia and eight commercial terpenes, were evaluated against nine strains of clinically relevant *Candida* spp. (*C.albicans* ATCC 10231, *C. albicans* ATCC 64550, *C. parapsilosis* ATCC 22019, *C. tropicalis* ATCC 750, *C. tropicalis* ATCC 200956, *C. glabrata* LMDM 34, *C.metapsilosis* MUM 1512, *C.orthopsilosis* MUM 1713 and *C.lusitaniae* MUM 1708) with different antifungal susceptibility profiles. EOs were obtained from plant material by using hydro-distillation, and their components were analyzed by GC/MS. Antifungal activity was determined using the broth microdilution reference method CLSI M27-A3. Initially, a screening of all EOs and terpenes was carried out at 256 µg/mL, and for those in which >90% growth inhibition was observed, minimal inhibitory concentrations (MICs) were determined using concentrations from 256 to 16 µg/mL. The MICs corresponded to the lowest concentration of the EOs or terpenes where a >90% reduction of visible fungal growth was observed. In addition, the susceptibility profiles of all yeasts were evaluated with fluconazole(FLZ), amphotericine B(AMB)and itraconazole(ITZ). The results were expressed as ranges and geometric means (GM). The cytotoxicity of the compounds that showed the highest antifungal activity was tested by means of an MTT assay using the human immortalized keratinocyte cell line HaCat.

**Results:** The strains of *Candida* spp. showed different antifungal susceptibility profiles; GM MICs and ranges values were: 0.13 (0.03–2 µg/mL), 0.11 (0.03->16 µg/mL) and 4.66 (0.5–32 µg/mL) for AMB, ITZ and FLZ, respectively. The terpenes thymol and limonene showed the best antifungal activity, inhibiting all yeasts with GM-MIC ranges of 256–128 ug/mL and 128–8 µg/mL respectively. *Candida tropicalis* ATCC 200956, a yeast with known resistance to azoles and amphotericin B, was susceptible to 18 of 20 compounds evaluated. *C. lusitaniae* MUM 1708 and *C. methapsilosis* MUM 1512 presented sensitivity to eight EOs (GM-MIC range 256–128 µg/mL) and five terpenes (GM-MIC range 256–8 µg/mL), while ATCC 10231, *C. albicans* ATCC 64550, *C. glabrata* LMDM 34 and *C. tropicalis* ATCC 750, just were susceptible to terpenes, and none of the EOs showed any activity. The cytotoxic activity obtained from EOs and terpenes on the HaCat cell line showed IC_50_ values ranging from 354.7 to 903.6 µg/mL. Limonene displayed higher selectivity to *C. methapsilosis* MUM 1512, *C. orthopsilosis* MUM 1713, *C. tropicalis* ATCC 200956 and *C. glabrata* LMDM 34 with selectivity index (SI) of 50, 25, 12 and 6 respectively.

**Conclusions:** The (SI) obtained allow one to identify compounds as possible alternatives for the design of products for the eradication of resistant yeasts. The broad sensitivity of the multiresistant *C. tropicalis* ATCC 200956 to terpenes and EOs suggests that their mechanism of action may be different from that of the main antifungals of clinical use.

**Acknowledgments:** The authors thank funding from the Ministry of Science, Technology and Innovation, the Ministry of Education, the Ministry of Industry, Commerce and Tourism, and ICETEX, Programme Ecosistema Científico-Colombia Científica, FJC Fund, Grant RC-FP44842-212-2018.

## P346 The Quest for Antifungal Compounds with Novel Mode of Action against *Candida albicans* by Mining the Soil Microbiota

**Paul Vandecruys ^1,2^, Giel Vanreppelen ^1,2^, Jurgen Wuyts ^1,2^, Haibo Hu ^3^, Walter Luyten ^3^ and Patrick Van Dijck ^1,2^**

^1^ KU Leuven Laboratory of Molecular Cell Biologyleuven/VIB
^2^ VIB-KU Leuven Center for Microbiology

**Objectives:** The repertoire of antifungal drugs currently in use to combat systemic fungal infections is finite and restricted to three major classes, each with their own mode of action (MOA). With high patient mortality rates, emerging resistance to frequently used antifungal drugs and the rise of emerging fungal pathogens like *Candida auris*, the necessity for novel antifungal drugs, with new mode of actions, is considerable. With our research we wish to meet this need.

**Materials & Methods:** Historically, microorganism-produced compounds have provided us with breakthrough drugs such as penicillin, amphotericin B and caspofungin. Gradually, re-discovery of compounds led to reduced interest to turn to nature for novel antifungal drugs. However, the potential of environmental organisms to yield antifungals with novel MOA, is far from exhausted since standard cultivation techniques fail to support the growth of most microorganisms [1]. In order to tap into this underexplored diversity, we applied In Situ cultivation with the use of a isolation chip (ichip) for high-throughput microbial cultivation [2]. To direct our efforts towards the purification of compounds that display a novel MOA, impedance spectroscopy, using the CellSine technology [3], was applied [4]. With this method, *C. albicans* is grown in microtiter plates that are modified with electrodes through which a small alternating current is applied. The response of the culture to this signal can be measured as impedance, the alternating current analogue of electrical resistance. These impedance profiles are distinct for the different antifungal drug classes and may therefore be indicative for the MOA by which the fermentation extract inhibits *C. albicans* growth.

**Results:** Soil from sixteen different locations in Belgium was sampled with a focus on deciduous forest, wet and dry heathland. Over 4000 isolates were cultured in various conditions and tested for antifungal compound production. Here, we focused on anti-*Candida albicans* activity. Fermentation extracts that inhibited the growth of our test-organism were retained and 360 hits, organisms that produce compounds that inhibit *C. albicans*, were obtained. Evaluation of these extracts by impedance spectroscopy yielded several fermentation extracts with potential novel MOA.

**Conclusions:** These extracts are currently further investigated. By bioactivity-guided fractionation, LC-MS based compound-identification and NMR structure determination, we aspire to identify the active compounds within these fermentation extracts and deliver lead compounds with a novel MOA for antifungal drug development.

**References**

1. Hug, L.A. Sizing Up the Uncultured Microbial Majority. *mSystems* **2018**, *3*.
2. Nichols, D.; et al. Use of ichip for high-throughput in situ cultivation of “uncultivable” microbial species. *Appl. Environ. Microbiol.* **2010**, *76*, 2445–2450.
3. CellSine. Widening your drug discovery horizon 2018. May 2021. Available online: https://www.cellsine.com (accessed on 8 October 2021).
4. Bouhenna, M.M.; et al. Anticancer Activity Study of Chromone and Coumarin Hybrids using Electrical Impedance Spectroscopy. *Anticancer Agents Med. Chem.* **2018**, *18*, 854–864.

## P347 Development and Evaluation of Antimicrobial Peptides from the Scarabaeidae *Coleopteran* Family against Pathogenic Yeasts with Public Health Impact

**Julián E. Muñoz ^1^, Beatriz L. Gómez ^1^, Carolina Firacative ^1^, David Andreu Martínez ^2^, Javier Valle ^2^, Bruno Rivas Santiago ^3^, Germán Alberto Tellez ^4^, Lily Johana Toro ^4^, Diana Carolina Henao ^4^ and Jhon Carlos Castaño Osorio ^4^**

^1^ School of Medicine and Health Sciences, Universidad del Rosario
^2^ Universidad Pompeu Fabra
^3^ Instituto Mexicano del Seguro Social
^4^ Universidad del Quindío

**Objectives:** Host defense peptides (HDP) are produced within the diversity of beetles. The aims of this work were to find new promising peptides from the Coleoptera family *Scarabaeidae* with potential biomedical applications, to modify physicochemical and structural characteristics of these peptides in order to improve their antimicrobial properties, and to evaluate the in vitro activity of the HDPs against reference strains of pathogenic *Candida* and *Cryptococcus* species.

**Materials & Methods:** From the *Scarabaeidae* family transcriptome, 14 promising HDPs were identified. Subsequently, we designed new sequences modifying the net charge, hydrophobic angle and the general composition of amino acids, among other properties, in order to improve the HDPs antifungal activity. The in vitro antifungal susceptibility of the 14 modified HDPs against *Candida krusei*, ATCC 6558, *Candida parapsilosis* ATCC 22019, *Candida glabrata* ATCC 2001, *Candida tropicalis* ATCC 750, *Cryptococcus neoformans* H99 and *Cryptococcus gattii* H0058-I-2029 isolates were evaluated by broth microdilution, with a concentration ranging from 0.19 to 50 μg/mL for all peptides.

**Results:** From the 14 peptides, all showed in vitro activity against *C. krusei, C. parapsilosis* and *C. glabrata*. Six peptides showed in vitro activity against *C. tropicalis*, 12 against *C. neoformans* and 13 against *C. gattii*. MIC ranges per species and per peptide are shown in Table 1.

**Table 1.** Minimum Inhibitory Concentrations (MICs) of 14 peptides designed.




**Minimum Inhibitory Concentration (MIC in μg/mL) against Different Fungal Strains Evaluated**

***Candida***

***Cryptococcus***

**Peptide Name**

**Sequence**

***C. krusei* ATCC 6558**

***C. parapsilosis* ATCC 22019**

***C. albicans***

**SC5314**

***C. tropicalis***

**ATCC 750**

***C. albicans***

**ATCC 1453**

***C. glabrata***

**ATCC 2001**

***C. neoformans***

***H99***

***C. gattii***

***H0058-I-2029***
Act1KSKRWRKFEKRVKKIFEKTKEA-amide
**1.56**

**3.12**
>5050>503.12506.25Act2KSKRWRKFEKRVKKIFEKTKEAK-amide
**0.78**

**1.56**
>5050>501.56253.12Act2.1K(pS)KRWRKFEKRVKKIFEKTKEAK-amide (pS: phosphoserine)
**0.78**

**1.56**
>50>50>501.56>501.56Act2.2KSKRWRKFEKRVKKIFEKFKEAK-amide
**0.78**

**1.56**
>5012.5>501.5612.51.56Act3KSKRWRKFEKRVKKIFEHTKEA-amide
**0.78**

**3.12**
>50>50>501.56503.12Act4KSKRWRKFEKRVKKIFEHTKEAK-amide
**0.78**

**1.56**
>5050>503.12501.56Act5GSKRWRKFEKRVKKIFEKTKEA-amide
**1.56**

**3.12**
25–50>50>503.12251.56Act6GSKRWRKFEKRVKKIFEKTKEAK-amide
**0.78**

**1.56**
>50>50>503.12503.12Act7GSKRWRKFEKRVKKIFEHTKEA-amide
**0.78**

**1.56**
>50>50>503.12503.12Act8GSKRWRKFEKRVKKIFEHTKEAK-amide
**0.78**

**1.56**
>50>50>503.12503.12domcecGSKRWRKFEKRVKK-amide
**3.12**

**3.12**
>50>50>506.25>50>50ConCecGSKRWRKFEKRVKKIFEETKEALPVVQGVAGVAGAVGRR-amide
**0.78**

**0.39**
>5025>501.56251.56ox322GSKRWRKFEKRVKKVFEHTKEA-amide
**1.56**

**1.56**
>50>50>503.12503.12sat122RSKKWRKIEKRVKKIFEKTKEA-amide
**1.56**

**0.39**
>5050>503.12503.12 

**Conclusions:** The HDPs herein analyzed showed a significant in vitro antifungal activity against six *Candida* and two *Cryptococcus* pathogenic species. These findings encourage further work with in vivo experimental models in order to better understand the mechanisms of action of these and other antimicrobial peptides. HDPs from different species are increasingly being used as a promising therapeutic alternative in the control of fungal infections.

## P348 Antifungal Activity of Honokiol against Dermatophytes: Insights into Mechanism of Action and Combinatorial Effects with Terbinafine

**Adriana Trifan ^1^, Andra-Cristina Bostanaru ^2^, Simon Vlad Luca ^3^, Alexandra Jitareanu ^1^ and Mihai Mares ^2^**

^1^ Gr. T. Popa University of Medicine and Pharmacy
^2^ Ion Ionescu de la Brad University of Life Sciences
^3^ TUM School of Life and Food Sciences

**Objectives:** Honokiol, a natural compound found in the stem bark of *Magnolia* species, was investigated for its antifungal activity against standard strains and clinical isolates of dermatophytes. Our study aimed to determine its mechanism of activity and to assess the ability of honokiol to improve the antifungal activity of terbinafine against dermatophytes.

**Materials & Methods:** The minimum inhibitory concentration (MIC) of honokiol was assessed by broth microdilution method against standard strains (*Trichophyton rubrum* ATCC 28188, *Trichophyton mentagrophytes* ATCC 9533), and clinical isolates (*T. rubrum, T. ajelloi, Microsporum canis* and *M. gypseum*) [1]. Evaluation of honokiol effect on ergosterol biosynthesis was assessed by high performance liquid chromatography [2]. Checkerboard microtiter method was used to identify synergistic combinations of honokiol with terbinafine against *Trichophyton rubrum* ATCC 28188. The combinatorial effect was evaluated by calculating the fractional inhibitory concentration index (FICI) [3].

**Results:** MIC values of honokiol ranged from 8 to 16 mg/L for all tested strains, whereas the positive control, terbinafine, displayed MIC values of 0.03–0.06 mg/L. The mechanism of antifungal activity is related to the ability of honokiol to damage the cell membrane of *T. rubrum*; honokiol significantly reduced the ergosterol content in dermatophyte (9.52 ± 0.43 mg%, compared to 13.56 ± 0.35 mg% in control, *p* < 0.001). More, honokiol showed additive interactions with terbinafine against *T. rubrum* (FICI values of 0.53–0.75).

**Conclusions:** Our study showed that honokiol can be regarded as a putative drug for controlling dermatophytes growth; also, combinatorial strategies that include honokiol and terbinafine can exert a multi-target antifungal activity, being effective in the treatment of dermatophytosis, and more, in reducing fungal resistance.

**Acknowledgments:** This work was supported by a grant of the Romanian Ministry of Education and Research, CNCS-UEFISCDI, project number PN-III-P1-1.1-TE-2019-1894, within PNCDI III.

**References**

1. Arendrup, M.C.; Meletiadis, J.; Mouton, J. *Eur. Comm. Antimicrob. Susceptibility Test.* **2017**, 1–23.
2. Lopes G.; Pinto E.; Andrade P.B. *PLoS ONE* **2013**, *8*, e72203.
3. Schwalbe R.; Steele-Moore L.; Goodwin A.C., (Eds.). Antimicrobial Susceptibility Testing Protocols; CRC Press: Boca Raton, FL, USA, 2007; pp. 275–340.

## P349 In Vitro Antifungal and Cytotoxic Effects of Morencidine-Like Peptide against Standard Strains of *Candida albicans, Candida glabrata* and *Candida tropicalis*

**Nasrin Amirrajab, Noor Ali Ahmadi Sarsahra, Seyed Amin Ayatollahi Mousavi, Behrooz Taheri and Samira Salari**

Ahvaz Jundishapur University of Medical Sciences

**Keywords:** morencidine-like peptide; *Candida albicans*; *Candida glabrata*; *Candida tropicalis*; nystatin

**Objectives:** Today, candidiasis is one of the most important infectious diseases in the world due to *Candida* species. *Candida albicans* is one of the most important opportunistic pathogenic fungi, which is the most common etiological cause of candidiasis. Due to the fact that the resistance of fungi to the number of antifungal drugs has increased and many of these drugs are toxic and expensive, it is necessary to study the natural products effective on these fungi. Therefore, the aim of this study was to investigate the in vitro antifungal and cytotoxic effects of morencidine-like peptide against standard strains of *Candida albicans, Candida glabrata*, and *Candida tropicalis*.

**Methods & Materials:** To evaluate the antifungal effect of each combination of nystatin and morencidine-like peptide, the protocol presented in CLSI M27-A3 and CLSI M27-S4 was used and the minimum inhibitory concentration was determined.

**Results:** The maximum inhibitory effect of morencidine-like peptide composition was 8 µg/mL for *Candida tropicalis* and *Candida albicans* and 32 µg/mL for *Candida glabrata*. The MIC of nystatin was determined to be 1.25 µg/mL for *Candida glabrata* and *Candida albicans* strains and 0.625 µg/mL for *Candida tropicalis* strains. The MFC composition of morencidine-like peptide was determined for *Candida tropicalis* and *Candida albicans* strains 8 µg/mL and for *Candida glabrata* strain 64 µg/mL. The results of cytotoxicity and hemolysis of morencidine peptide test on macrophage showed that morencidine peptide has no cytotoxicity and toxicity properties.

**Conclusions:** According to the results of the present study, the combination of morencidine-like peptide could be a new strategy in the treatment of infections caused by *Candida* strains. The discovery of the exact mechanism of which requires extensive clinical studies in this field.

## P350 Developing Efficient Probiotic Treatment for Vulvovaginal Candidiasis by Using and Improved Platform

**Liesbeth Demuyser ^1,2^, Mart Sillen ^2^, Silke Baldewijns ^2^, Ilse Palmans ^1,2^, Paul Vandecruys ^1,2^ and Patrick Van Dijck ^1,2^**

^1^ VIB-KU Leuven Center for Microbiology
^2^ KU Leuven, Department of Biology

**Objectives:** Although a lot of progress has been made over the past decades to equalize the rights of men and women, a gender imbalance is still present in health-related aspects of scientific research, termed the gender health gap. Female-specific health issues, such as vulvovaginal candidiasis (VVC), are studied to a far less extent compared to male-specific diseases. The main reason here is the lack of funding. This is striking, as one can appreciate that a disease, such as VVC, affecting 75% of all women worldwide at least once in their life and causing substantial economic losses, would indeed draw the attention of governmental funding agencies. In case of recurrent (R)VVC, women experience at least four episodes of infection every year, further increasing the emotional and economic burden.

Treatment of VVC is currently limited to azole intake or polyene application. Especially in case of non-*albicans* VVC and RVVC, treatment is insufficient, as misdiagnosis, fast recolonization and resistance impair efficient clearance. With this project, we aim to develop a novel probiotic therapy to target *Candida* infections in the vaginal niche, thereby striving towards improvement of the patients’ wellbeing.

**Materials & Methods:** By far most of the research on VVC has been performed in non-optimal in vitro conditions, using systemic *Candida* isolates and non-representative medium. We used an optimized in vitro platform to select *Candida* isolates that represent significant pathogenicity in the mimicked vaginal niche. Using the same platform, we identified a number of probiotic micro-organisms that show great promise in inhibiting virulence of *Candida* in this niche.

**Results:** (1) In this study, we screened a set of 150 vaginal *Candida* isolates for virulence properties specific to the vaginal niche. The isolate representing the highest pathogenicity was used to identify promising probiotic organisms and active metabolites. (2) The use of lactobacilli as probiotic therapy against VVC is debated. We, indeed, find that certain bacterial strains increase rather than inhibit pathogenicity of *Candida* and point out the role of lactic acid in this process. By using our optimized in vitro platform, we show the potential of specific *Saccharomyces cerevisiae* strains in inhibiting growth and virulence by *C. albicans* and *C. glabrata*. We identified a role for specific fatty acid metabolites in this process.

**Conclusions:** By using an appropriate platform, we investigated the potential of certain lactobacilli and *S. cerevisiae* strains, and combinations thereof, in inhibition of *Candida* virulence.

## P355 Characterization of the Activity of Amphotericin B Used in AmBisome against the Most Relevant Clinical Fungal Species

^1^ Health Institute Carlos III
^2^ Institute of Hygiene and Medical Microbiology, Medical University of Innsbruck
^3^ Department of Medical Mycology, Radboud University Medical Center, and Radboudumc/CWZ Center of Expertise in Mycology
^4^ Department of Microbiology. Faculty of Medicine, University of Hong Kong
^5^ PHW Mycology Reference Laboratory, University Hospital of Wales
^6^ McGovern Medical School, The University of Texas
^7^ Federal University of Health Sciences (UFCSPA)

**Objectives:** Amphotericin B (AmB) is one of the most widely used antifungals in clinics but it presents associated toxicity. To reduce deleterious effects, nowadays it is mainly administered in liposomal formulation, mainly AmBisome (Gilead). Paradoxically, no detailed studies have been performed with this specific Amb. The objective of this study is to describe the in vitro antifungal activity of AmB used in the AmBisome formulation.

**Methods:** We performed a multicenter study to determine the antifugal susceptibility to AmB from AmBisome (provided by Gilead) using both CLSI and EUCAST protocols. We tested this activity against the most relevant clinical fungal species.Representative isolates from different geographical regions were included in the study. Other antifungals were evaluated (fluconazole, itraconazole, voriconazole, posaconazole, caspofungin, micafungin and anidulafungin).

**Results:** AmB from AmBisome showed a wide spectrum of activity against yeast species using both EUCAST and CLSI protocols in seven different laboratories. The strongest activity was shown against *Candida albicans* (GM-EUCAST 0.23 mg/L, CLSI 0.21 mg/L), *C. parapsilosis* (GM-EUCAST 0.36 mg/L, CLSI 0.38 mg/L), *C. glabrata* (GM-EUCAST 0.34 mg/L, CLSI 0.33 mg/L), *C. auris* (GM-EUCAST 0.46 mg/L, CLSI 0.31 mg/L), and *C. tropicalis* (GM-EUCAST 0.36 mg/L, CLSI 0.31 mg/L). In filamentous fungi, the activity was variable depending on the species. Lowest MICs were found for *Aspergillus fumigatus* (GM-EUCAST 0.65 mg/L, CLSI 0.64 mg/L), and *A. niger* (GM-EUCAST 0.22 mg/L, CLSI 0.36 mg/L), while for other *Aspergillus* species, there was a higher variability in the MICs (*A. flavus*, GM-EUCAST 1.7 mg/L, CLSI 1.1 mg/L; *A. terreus* GM-EUCAST 2 mg/L, CLSI 1.4 mg/L; *A. nidulans* GM-EUCAST 1.7 mg/L, CLSI 0.9 mg/L). For other filamentous fungi, AmB from AmBisome showed a strong activity (GM around 0.2–0.5 mg/L for both methods, including *Talaromyces marneffei*), while the lowest activity was detected against *Lomentospora prolificans* and *Cunninghamella bertholletiae*.

**Conclusions:** AmB from AmBisome showed a wide spectrum of action against most relevant fungal pathogens, and only showed low activity against some species, such as *L. prolificans* or *C. bertholletiae*. No significant differences were detected between the geographical origin of the species. This ongoing study will provide a full description of the antifungal activity of the AmB drug that is most widely used in clinics.

**Funding:** This work is funded by Gilead (Reference: CO-ES-131-4510).

## P356 A Retrospective Review of the Antifungal Stewardship Programme, University Hospitals of Leicester; Effectiveness, Benefits and Challenges

**Lauren Ramm, Nelun Perera, Sarah Hackney, Matthew Kennedy, Rosalind Saunders, James Veater and Corrine Ashton**

University Hospitals of Leicester NHS Trust

**Objectives:** In January 2020 the University Hospitals of Leicester NHS Trust (UHL), implemented a trust wide antifungal stewardship (AFS) programme to optimise antifungal prescribing. The mycology laboratory offered access to conventional and indirect diagnostics in mycology with rapid turnaround times. The aim was to review the effectiveness, benefits, and challenges of implementing an AFS programme.

**Methods:** All adult and paediatric in-patients commenced on antifungal drugs (fluconazole, itraconazole, voriconazole, isavuconazole, caspofungin, Ambisome and flucytosine) for ‘likely’ or proven invasive fungal infections (IFI) were identified using daily reports generated from the electronic prescribing system and the trust pharmacy dispensing system. Patients prescribed antifungals for superficial fungal infections e.g., oral and genital thrush, those on prophylactic antifungals and those admitted to hospital on established treatment were excluded.

A combination of front-end and back-end stewardship approaches were adopted, facilitated by fungal culture and two fungal biomarkers (β-D-glucan and galactomannan) with rapid turnaround times. Rapid diagnostics alongside a dedicated team comprising of an antifungal pharmacist, a team of microbiologists and ward teams allowed thorough reviews of patients prescribed antifungal agents from admission until discharge/death. The patients were reviewed at least weekly, initially ward based (pre Covid-19) and later virtually (post Covid-19).

Patient demographics, criteria for diagnosis, prescribed drugs, advice provided by the AFS team and side effects were collected at the time of review. Absolute antifungal consumption and antifungal consumption weighted per 1000 admissions, along with trust spend on antifungal drugs were later collated from hospital databases.

**Results:** Between January and December 2020 a total of 244 patients with ‘likely’ IFI were reviewed. This included 231 adult and 13 paediatric patients. 24% had ‘likely’ invasive mould infections while 76% were ‘likely’ *Candida* infections of which 26 had a confirmed candidaemia.

Absolute antifungal consumption measured as daily defined doses (DDD) following the introduction of the AFS programme showed a reduction by 14% compared to 2019. Absolute antifungal expenditure showed a significant decrease from a monthly average of £59,799 in 2019 to £33,081 in 2020 (excludes posaconazole which is used for prophylaxis in the trust) Figure 1. This data should be interpreted considering changes in trust activity relating to Covid-19; however the weighted data per 1000 admissions also suggests a positive impact on consumption and spend on antifungals. Continued monitoring will provide confirmation of impact.

Interventions by the AFS team included advice on mycological sampling, source identification and control, drug, dose, route and duration of treatment and therapeutic drug monitoring.

**Conclusions:** The introduction of a dedicated AFS programme together with timely diagnostics provides opportunity for targeted interventions optimising antifungal prescribing and containment of antifungal expenditure.

## P357 Early Acquisition of Azole Resistance in Experimental *Candida albicans* Populations Exposed to Fluconazole

**Pilar Menéndez-Manjón ^1,2^, Inés Arrieta ^1,2^ and M. Dolores Moragues ^1,2^**

^1^ Department of Nursing I, University of the Basque Country UPV/EHU
^2^ Department of Immunology, Microbiology and Parasitology, University of the Basque Country UPV/EHU

**Objectives:** The development of azole resistance in *Candida albicans* represents a serious problem in the clinic due to treatment choice limitations among the small range of available antifungal drugs. Four resistance mechanisms have been broadly described, but there are still *C. albicans* clinical isolates with unknown resistance mechanisms. Consequently, evolution experiments in the presence of antifungal drugs are extremely relevant. Accordingly, the aim of this study was to analyze the evolution of azole resistance in *C. albicans* after exposition to increasing concentrations of fluconazole (FLC).

**Materials & Methods:** Two experimental populations, founded from a single colony of *C. albicans* SC5314 reference strain and BE-47 clinical isolate, respectively, were serially propagated for 23 days in RPMI supplemented with increasing concentrations of FLC, from 0.25 to 64 µg/mL. Control populations were grown in parallel without the drug. Following the fluconazole exposure period, the control and experimental populations were propagated daily in RPMI without fluconazole for 22 additional days. Susceptibility to azoles was evaluated at several time-points of the experiment. *ERG11, TAC1, UPC2, MRR1* and *MRR2* genes were sequenced, and expression of *ERG11, CDR1* and *MDR1* genes was measured. Moreover, fitness of experimental and control populations in the absence of fluconazole was analyzed, as well as their zygosity for the mating-type-like locus (*MTL*).

**Results:** After 7 days of exposure to fluconazole, the SC5314 experimental population increased the MIC from 0.5 to 8 μg/mL, and remained FLC resistant throughout the experiment. This change was accompanied by a mutation N977D in Tac1, already described and associated to resistance and *CDR1* overexpression. However, we did not observe associated fitness-costs at any time-point of the experiment. The *MTL* zygosity study revealed that the experimental population was heterogeneous, since we found clones homozygotes and heterozygotes for this locus. On the other hand, even though the BE-47 experimental population increased slightly the MIC for all the azoles tested, it did not harbour any mutation in any of the genes studied.

**Conclusions:** The exposition of *C. albicans* SC5314 to low concentrations of fluconazole induced an early rise of the MIC value accompanied by the acquisition of a resistance-associated mutation (N977D in Tac1) with apparently no fitness cost, while BE-47 does not appear to have acquired resistance to fluconazole. Additionally, exposure to antifungal drugs can induce the appearance of subpopulations with different resistance or adaptive mechanisms.

Furher studies are warranted in order to identify new resistance mechanisms and to help in the search for new antifungal targets.

This work was supported by the Basque Government (IT 913-16).

## P358 E-Test Performance in Determination of *Aspergillus fumigatus* Susceptibility to Azoles

**Ravil Huseynov ^1^, Samir Javadov ^1^, Ali Osmanov ^2^ and David Denning ^2,3,4^**

^1^ Azerbaijan Medical University
^2^ Global Action Fund for Fungal Infections
^3^ Faculty of Biology, Medicine and Health, University of Manchester, Manchester Academic Health Science Centre
^4^ National Aspergillosis Centre, Wythenshawe Hospital, Manchester University NHS Foundation Trust, Manchester Academic Health Science Centre, Southmoor Road

**Keywords:** *Aspergillus fumigatus*; e-test; broth microdilution test

**Objectives:** Increase of mould-associated invasive infections (especially, *Aspergillus* spp.) demands usage of easy-to-perform and fast susceptibility testing methods. The aim of the study was to compare efficacy of E-test and European Committee on Antimicrobial susceptibility testing (EUCAST) broth microdilution methods in evaluation of susceptibilities of *Aspergillus fumigatus* isolates to azoles.

**Methods:** The research was conducted on 29 *A. fumigatus* strains. Minimum inhibitory concentrations (MIC) were detected by broth microdilution method (BM) following EUCAST definitive document E.DEF 9.3.2 and E-test in accordance with manufacturer instructions.

**Results:** The percent agreement after 48 h (24 h) between 2 tests was 96.5% (100%) for itraconazole, 86.2% (96.5%) for voriconazole and 100% (96.5%)—for posaconazole (Table 1).

**Table 1.** Comparison of EUCAST broth microdilution and E-test methods.



**Agents**

**Time**

**Broth Microdilution**

**E-Test**

**Pearson’s Correlation**

**Percent Agreement**

**(BM/E)**

**MIC Range**

**MIC_50_**

**MIC_90_**

**MIC Range**

**MIC_50_**

**MIC_90_**
Itraconazole28 h0.5–80.50.50.25–80.7510.97710048 h0.5–160.510.5–640.7510.89696.5Voriconazole28 h0.25–40.510.125–40.250.380.67796.548 h0.25–8120.125–80.250.380.66686.2Posaconazole28 h0.125–20.250.50.125–20.190.250.88596.548 h0.125–40.250.50.09–20.190.250.965100
MIC—minimum inhibitory concentration, BM—broth microdilution, E—E-test.


**Conclusions:** E-test method has shown good correlation with broth microdilution method. E-test method can be conducted routinely as alternative to time-consuming broth microdilution method.

## P359 High Burden of Azole-Resistant *Aspergillus fumigatus* in Veterinary Samples in Belgium—Rethinking Clinical Breakpoints in Veterinary Medicine

Hanne Debergh ^1,2^, Claude Saegerman ^2^, Roel Haesendonck ^3^, Nadine Botteldoorn ^4^ and Ann Packeu ^1^

^1^ Mycology and Aerobiology, Sciensano
^2^ Fundamental and Applied Research for Animal and Health (FARAH) Center, Uliège
^3^ Zoolyx
^4^ Animal Health Care Flanders (DGZ)

**Objectives:** Although the full burden of azole resistant *Aspergillus* spp. is not fully elucidated, it is clear that it is becoming a public health threat worldwide. A trans-disciplinary approach, using the OneHealth concept, involving medicine, veterinary medicine and environmental science, is necessary to address globally emerging diseases. We aimed at estimating the prevalence of resistant *A. fumigatus* in veterinary isolates from Belgium.

**Materials & Methods:** Veterinary *A. fumigatus* isolates were collected by two veterinary laboratories in 2020 and 2021 in Flanders, Belgium. Isolates originate from companion animals, production animals and zoo animals.

All have been confirmed as *A. fumigatus* by MALDI-TOF MS using the BCCM/IHEM database. The antifungal susceptibility was evaluated using the microdilution EUCAST method. *Cyp51A* gene sequencing has been performed on all resistant isolates.

**Results:** A total of 40% of the isolates were resistant to at least one medical azole (voriconazole, itraconazole, isavuconazole and posaconazole). Only 6.7% of the animals were treated with antifungals. No resistance was observed for itraconazole (ITC) and isavuconazole (ISA), however 33% had values equal to the clinical breakpoint for ITC and 27% were in the area of technical uncertainty (ATU) for ISA. Regarding voriconazole and posaconazole, 47% and 60% had values in ATU, respectively. The high prevalence of MIC values in ATU in veterinary isolates raises the question if the same clinical breakpoints should be used for veterinary samples.

**Conclusions:** Considering the OneHealth concept, high burden of azole-resistant *A. fumigatus* veterinary isolates is highly alarming. More data needs to be collected regarding azole resistance in veterinary isolates and their subsequent breakpoints.

## P360 Influenza-Induced Epithelial Barrier Disruption Enhances Invasive Pulmonary *Aspergillosis*

**Laura Seldeslachts ^1^, Cato Jacobs ^2^, Birger Tielemans ^1^, Lore Vanderbeke ^3^, Stephanie Humblet-Baron ^4^, Katrien Lagrou ^3^, Erik Verbeken ^5^, Joost Wauters ^2^ and Greetje Vande Velde ^1^**

^1^ Department of Imaging and Pathology, Biomedical MRI Unit/MoSAIC, KU Leuven
^2^ Department of Microbiology, Immunology and Transplantation, Laboratory for Clinical Infectious and Inflammatory Disorders, KU Leuven
^3^ Department of Microbiology, Immunology and Transplantation, Laboratory of Clinical Bacteriology and Mycology, KU Leuven
^4^ Department of Microbiology, Immunology and Transplantation, Laboratory of Adaptive Immunity, KU Leuven
^5^ Department of Imaging and Pathology, KU Leuven

**Objectives:** In the last decade, co-infection with *Aspergillus* species has been increasingly recognised worldwide in critically ill influenza patients without classical EORTC-host factors (Donnelly et al., 2019). Despite the increasing clinical awareness and the development of preclinical murine models (Tobin et al., 2020) (Seldeslachts et al., ECCMID 2021), the exact causative pathways for influenza-associated pulmonary aspergillosis (IAPA) remains to be elucidated. Here, we investigated whether influenza-induced disruption of the respiratory epithelial barrier provides a passageway for invasive growth of *Aspergillus* in murine IAPA.

**Materials & Methods:** Immunocompetent mice were challenged with an intranasal instillation of influenza A on day 0 followed by orotracheal inoculation with *Aspergillus fumigatus* 4 days later. Single infection controls (either influenza-only or *A. fumigatus*-only) were included. After sacrifice (day 7) lungs were isolated, filled with 10% formalin in phosphate buffered saline (PBS) and transferred to 0.1% Na^+^ Azide in PBS solution after 24 h. 5 µm paraffine sections were cut and stained with haematoxylin-eosin (H&E) and Grocott’s methenamine silver (GMS) staining. Stainings were scored blinded by an experienced pathologist (EV).

**Results:** In influenza-infected mice, we observed a diffuse lymphocytic bronchitis and pneumonia pattern, with necrosis and erosion of alveolar/bronchial epithelium (Figures 1A and 2A). *Aspergillus*-only instillation resulted in a focal deposit of *Aspergillus* conidia (mainly found intracellular in macrophages) at the beginning of the alveolar parenchyma, corresponding to the region where air flow drops. This focal presence of conidia was surrounded by a limited number of inflammatory cells, with no signs of bronchitis, pneumonia nor epithelial damage (Figures 1B and 2B). In contrast, co-infection with *Aspergillus* in influenza-infected mice resulted in a diffuse purulent hyperinflammatory response, characterized by a severe neutrophil-predominant bronchitis and pneumonia pattern, specifically observed in the region where influenza-induced epithelial damage forms a passageway for *Aspergillus* and results in invasive growth of *Aspergillus* hyphae (Figures 1C and 2C). In addition, bronchiectasis was observed in mice with IAPA disease (Figure 2C).

**Conclusions:** We observed a clearly distinct histological phenotype between IAPA and single infection control mice (*Aspergillus*-only and influenza-only). Together, these pathological results support a possible mechanism for IAPA development, consisting of a co-localization of influenza-induced destruction of respiratory epithelium with invasive hyphal growth of *Aspergillus*.

## P361 Incidence of *Candida* spp. in Oral Cavity of Healthy Individuals and Comparative Antifungal Potential of Selected Medicinal Plants

**Adijat Olabisi Atayese ^1^, Olufunke Shittu ^1^ and Parvaze Ahmad Wani ^2^**

^1^ Federal University of Agriculture Abeokuta
^2^ Crescent University Abeokuta

**Objectives:** This study investigated *Candida* spp. from the oral cavity of healthy individuals and possible alternative antifungal remedy using extracts of *Vernonia amygdalina, Zanthoxylum xathoxyloides* and *Prosopis africana* plants.

**Materials & Methods:** Two hundred and fifty-eight (258) samples were collected from the oral cavity of healthy newly admitted 100 Level students on routine medical check-up at the Health Centre of the Federal University of Agriculture, Abeokuta, Nigeria. Samples were grown of Chromagar *Candida* medium for isolation and rapid identification of *Candida* spp. based on colony characteristics while semi-nested Polymerase Chain Reaction (snPCR) was used to confirm species. *Vernonia amygdalina* leaves and twigs of *Zanthoxylum xathoxyloides* and *Prosopis africana* were extracted using a soxhlet extractor and concentrated with a rotary evaporator. Antifungal activity of extracts was determined using the agar well-diffusion method on Muller Hinton medium, while Minimum Inhibitory Concentrations (MIC) and Minimum Fungicidal Concentrations (MFC) were also determined. Thirty-seven samples (14.45%) collected were positive for *Candida* spp. Isolated were identified molecularly as *C. albicans* and *C. krusei* and *Candida* sp.

**Results:** The majority of students had only *C. albicans* inhabiting their oral cavity with a 75.68% incidence rate, while *C. krusei* and *Candida* sp. isolated in mixed culture had incidence rates of 21.62% and 2.7% respectively. Antifungal potential of the medicinal plants showed that both aqueous and ethanolic extracts of *V. amygdalina* had antifungal activity against only *Candida* sp. (38 mm) while aqueous extract of *P. africana* and ethanolic extract of *Z. xathoxyloides* showed low bioactivity (8–12 mm) against *C. albicans, C. krusei* and *Candida* sp. Inhibitions were observed at ≥50 mg/mL of *V. amygdalina*, and ≥150 mg/mL for both *Z. xathoxyloides* and *P. africana*, but MIC and MFC were non-determinable. It was also observed that both positive controls, Listerine (14–26 mm) and Oral-B (18–30 mm) had higher activity against all *Candida* spp. compared with both *Zanthoxylum xathoxyloides* and *Prosopis africana*, but *Vernonia amygdalina* showed the highest activity against *Candida* sp. compared with both conventional mouthwash products.

**Conclusions:** This study has shown an incidence rate of *Candida albicans* greater than 70% in healthy individuals in a tertiary institution in Nigeria. *V. amygdalina, P. africana* and *Z. xathoxyloides* exhibited varied antifungal activity on *Candida* spp. Further study on synergism and additivity of the tested plants is recommended.

## P362 Role of Pleiotropic Drug Resistance Transporters in the Azole Resistance of Fungi Causing Mucormycosis

**Sándor Kiss-Vetráb, Csilla Szebenyi, Bernadett Vágó, Rakesh Varghese, Kitti Bauer, Csaba Vágvölgyi, Tamás Papp and Gábor Nagy**

Department of Microbiology, University of Szeged

Mucormycosis is a life-threatening systemic infection caused by certain members of the filamentous fungal order Mucorales (*Rhizopus oryzae. Lichtheimia corymbifera*, and *Mucor circinelloides*). Mucormycosis is associated with a high mortality rate, which can be nearly 100% depending on the underlying condition of the patient. Treatment of mucormycosis is a great challenge because Mucormycotina species are intrinsically resistant to most of the routinely used azoles. Members of the ATP binding cassette transporter superfamily, especially the pleiotropic drug resistance (PDR) transporter subfamily, can play role in the drug efflux and drug resistance.

In the *Mucor* genome database, eight putative *pdr* genes were found. The relative transcript level of the *pdr* genes was measured after azole treatment using quantitative real-time PCR. We have started to create and characterize single (growth ability, susceptibility to different antifungal agents) and double knock-out mutant from *pdr* genes using a CRISPR-Cas9 system.

*Pdr* genes responded differently to the azoles but, only *pdr1* showed significantly increased relative transcript levels in response to all azoles. From the six tested azoles, only itraconazole had the widest effect on *pdr* expression upregulating five of the eight genes. Deletion of *pdr1* caused significantly increased transcript level of *pdr2* and *pdr6*, while the lack of *pdr6* resulted significantly increased transcript level of *pdr1. pdr7*, and *pdr8*. Disruption of *pdr1* and *pdr2* resulted increased sensitivity to posaconazole, ravuconazole, and isavuconazole.

Our result suggested that the regulation of *pdr* genes is highly interconnected and certain *pdr* genes may compensate the lack of the deleted genes. The azole resistance of *Mucor* cannot be completely explained by the activity of PDR proteins, other proteins, regulatory processes might be involved.

This study was supported by the grant GINOP-2.3.2-15-2016-00035 and the NKFI project K131796. G.N. is grateful for the support of the Premium Postdoctoral Fellowship Program of the Hungarian Academy of Sciences (460050).

## P363 Highly Conserved gsc1 Gene of *Pneumocystis jirovecii* in Patients with or without Prior Exposure to Echinocandins

**Pierre Bonnet ^1^, Solène Le Gal ^1^, Florent Morio ^2^, Claire Hoffmann ^1^, Fouleymata Diabira ^1^, Athéna de Quélen ^1^, Guillaume Curral ^1^, Virginie Cogulet ^1^, Jean-François Huon ^2^, Patrice Le Pape ^2^ and Gilles Nevez ^1^**

^1^ Brest University Hospital
^2^ Nantes University Hospital

**Objectives:** *Pneumocystis jirovecii* (*P. jirovecii*) is an opportunistic ascomycete responsible for severe pneumonia in immunosuppressed patients. Echinocandins are noncompetitive inhibitors of GSC1 subunit of the enzymatic complex involved in 1,3-β-D-glucan synthesis, 1,3-β-D-glucan being a major cell wall component of most fungi, including *Pneumocystis* spp. Among these drugs, Caspofungin has been widely used for treatment of systemic candidiasis or invasive aspergillosis whereas it has rarely been used for PCP treatment, its efficiency being a subject of debate. For these reasons, the diversity of the g *sc1* gene of *P. jirovecii* has rarely been studied whereas polymorphisms of the homologous genes *fks* of *Candida* spp., specifically in two hot spot (HS1 and HS2) regions have been reported. The objectives of the study were to provide original data on diversity of *P. jirovecii gsc1* gene and putative selection pressure of echinocandins on *P. jirovecii* microorganisms.

**Methods:** The sequences of the *gsc1* gene of *P. jirovecii* isolates obtained from two groups of patients who developed *P. jirovecii* infection were compared. One group of 27 patients had prior exposure to echinocandins whereas the second group of 24 patients did not have prior exposure to echinocandins, at the time of *P. jirovecii* infection occurrence. Two primer pairs were designed in order to amplify two portions of *gsc1* gene, HS1 and HS2, corresponding to hot spot regions previously described in *Candida* spp. PCR products were sequenced from the two strands. Consensus sequences were aligned and compared.

**Results:** No SNPs were detected within the two hot spots. Nonetheless, three SNPs at positions 2204, 2243, and 2303 close to HS1 region were identified. These three SNPs represent synonymous mutations that were previously reported. Another SNP at position 4540 close to HS2 region was identified. This SNP represents an unreported synonymous mutation. Considering the aforementioned SNPs, three *P. jirovecii gsc1* HS1 alleles (named A, B, and C) and two *gsc1* HS2 alleles (named a and b), and four haplotypes combining HS1 and HS2 alleles (named Ca, Cb, Aa, and Ba) were identified. Ca was the most frequent haplotype without significant difference in the two groups of patients.

**Conclusions:** The diversity of *P. jirovecii gsc1* gene in the two patient groups does not differ. The SNPs that were identified correspond to synonymous mutations that are not involved in codon changes. No specific pressure of echinocandins among *P. jirovecii* microorganisms can be pointed out so far.

## P364 Functions of the Transcription Factor pig1 and DHN-Melanin in *Scedosporium apiospermum*

**Hélène Guegan ^1^, Kévin Ravenel ^2^, Wilfried Poirier ^2^, Aymeric Delabarre ^3^, Odile Sergent ^3^, Jean-Pierre Gangneux ^1^, Amandine Gastebois ^2^ and Sandrine Giraud ^2^**

^1^ University Rennes, CHU, INSERM, EHESP, IRSET (Institut de Recherche en Santé, Environnement et Travail), UMR_S 1085, F-35000 Rennes, France
^2^ Groupe d’Étude des Interactions Hôte-Pathogène (EA 3142), SFR ICAT 4208, UNIV Angers, University Brest, Angers, France
^3^ University Rennes, INSERM, EHESP, IRSET (Institut de Recherche en Santé Environnement et Travail), UMR_S 1085, F-35000 Rennes, France

**Objectives:** *Scedosporium apiospermum* is an opportunistic filamentous fungus causing various human infections especially immunosuppressed patients, and colonizing the respiratory tracts in patients with cystic fibrosis. Like in many pathogenic fungi, the conidia cell wall is a key-element involved in the early recognition by the host immune system. Apart from different immunogenic polysaccharides constituting the cell wall, the 1,8-dihydroxynaphtalene (DHN)-melanin is highly preserved in the fungal kingdom, providing survival advantage in extreme environmental conditions. In *S. apiospermum*, the DHN-melanin biosynthesis is insured by successive enzymatic reactions, depending on the expression of a four-gene cluster, including a transcription factor, orthologue to Pig1. The present work was aimed to characterize the conidial phenotype of Δ*pig1* melanin-deficient strains compared to parental strains, as a starting point toward the elucidation of *pig1* functions and DHN-melanin involvement in *S. apiospermum* virulence processes.

**Methods:** The *pig1* deletion was performed using CRISPR Cas9 genome editing from two parental melanized *S. apiospermum* strains, the wild-type 14462 strain (WT) and the Δ*ku70* deleted 14462 strain (Δ*ku70*), in which the homologous recombination needed for transformation is facilitated. Hygromycin-resistant transformants were first submitted to molecular assays including Sanger sequencing and Southern-Blot to confirm the deletion of *pig1*. (i) Different phenotypical tests were performed from 9-day conidia, including melanin detection using electronic paramagnetic resonance (EPR), growth and conidiation tests. (ii) The cell wall structure was also explored using transmission electronic microscopy (TEM). The comparative exposure of polysaccharides residues at conidial surface in Δ*pig1* mutants was quantified using fluorescent lectins (concanavalin A and wheat germ-agglutin). (iii) Finally, the thermotolerance was explored, by culture after thermal shock at 40 °C and 50 °C.

**Results:** Three Δ*pig1* mutants were obtained, one from the WT strain and 2 from the Δku70 14462 strain. The loss of *pig1* was phenotypically associated with the absence of DHN-melanin in the cell wall, as revealed by the unpigmented Δ*pig1* mutant colonies, and confirmed by EPR. No change in the ability to grow and sporulate could be attributed to *pig1* deletion, showing that *pig1* is not essential for fungal growth. The cell wall thickness was drastically reduced in melanin- deficient Δ*pig1* mutants (−30%). Unexpectedly, *pig1* deletion triggered changes in conidia size and shape, as more than 40% of large septated conidia were observed. The absence of melanin layer drastically increased the exposure of mannose/glucose and N-acetylglucosamine (chitin) residues compared to the melanized spores. Lastly, the mean survival of Δ*pig1* mutants to 50 °C heat shock was drastically hampered compared to the parental strains (6% vs. 55% respectively).

**Conclusions:** These preliminary results confirm the regulatory role of *pig1* on melanin biosynthesis in *S. apiospermum* conidia. Structural changes and poor thermal survival of Δ*pig1* melanin-deficient conidia foreshadow the wider involvement of *pig1* in fungal biological processes, including virulence. Molecular mechanisms under *pig1* control in *S. apiospermum* conidia will be further investigated, based on a comparative transcriptome analysis (RNA-seq) between parental and Δ*pig1* mutants (ongoing).

## P365 In Vitro Efficacy of the Probiotic *Bacillus subtilis* NCIB 3610 Cell Free Supernatant on *Candida* spp. and *Aspergillus* spp.

**Aikaterina Poulopoulou ^1,2^, Maria Touraki ^2^ and Timoleon-Achilleas Vyzantiadis ^1^**

^1^ Department of Microbiology, Medical School, Aristotle University of Thessaloniki
^2^ Laboratory of General Biology, School of Biology, Aristotle University of Thessaloniki

**Objectives:** Probiotic bacteria are defined as live microorganisms, which when ingested or locally applied have specific health benefits for the host. In this category, there are included bacteria like *Bacillus* spp. and especially the bacteriocinogenic *Bacillus subtilis*, which is characterized by the secretion of metabolites with antimicrobial properties. Invasive fungal infections could be fatal to humans, especially in the immunocompromised patients. Bacteriocins target the cellular membrane of other microbes, create pores and interrupt the membrane potential. Thus, they induce cell apoptosis. According to this activity, bacteriocins could serve as potential antifungal agents.

**Methods:** The antifungal effect of the bacterial strain *Bacillus subtilis NCIB 3610* cell free culture supernatant (CFS) was evaluated against strains of *Candida albicans* (ATCC 90028), *Candida parapsilosis* (ATCC 90018), *Aspergillus fumigatus* (ATCC 204305), *Aspergillus flavus* (ATCC 204304) and clinical isolates of *Candida glabrata, Candida tropicalis, Aspergillus niger* and *Aspergillus terreus*. Supernatants of the *Bacillus’* cultures were collected after incubation, extracted and tested for their protein concentration and antimicrobial activity, lyophilized and administrated to the different fungi strains. Further experimental procedures included susceptibility testing of all isolates against the *Bacillus* supernatant by the use of the EUCAST protocol, modified with the addition of resazurin. As inhibitory concentration 50 (IC_50_) was determined the CFS concentration required in order to achieve a 50% decrease of fungal growth. Also, agar based experiments (Figures 1 and 2) of fungal growth in the presence of CFS or co-cultivation with *Bacillus subtilis* were performed.

**Results:** The results of the susceptibility testing against the *B. subtilis* CFS, showed that *A. niger* was mostly affected, approaching a growth decrease of 50% at a CSF concentration (IC_50_) of 16.97 mg/mL. For *A. flavus* and *A. fumigatus* the CFS IC_50_ was 24.36 mg/mL and 39.91 mg/mL respectively. On the contrary, *A. terreus* had an IC_50_ of 60.96 mg/mL. Interestingly enough, the yeasts tested on this experiment did not present lower IC_50_ than the moulds. Specifically, *C. albicans* had an IC_50_ of 45.55 mg/mL, while *C. glabrata, C. tropicalis* and *C. parapsilosis* an IC_50_ of 78.42 mg/mL, 74.75 mg/mL and 105.1 mg/mL respectively. The EUCAST method combined to the resazurin contributed to overcome the issues of color interference due to the lyophilized CFS and the broth media used.

**Conclusions:** The cell free supernatant of the probiotic *Bacillus subtilis* exhibited some specific and interesting antifungal properties, leading to the need for further testing and experimentation on the bacteriocins secreted by the strain of *B. subtilis* NCIB 3610 in the context of a potentially beneficial antifungal factor.

## P366 A Multicentre Registry of Patients Receiving Systemic Mould-Active Triazoles (MATs) for Invasive Fungal Infections (IFIs): Sub-Analysis of Prophylaxis Administration

**Minh Hong Nguyen ^1^, Luis Ostrosky-Zeichner ^2^, Joseph Bubalo ^3^, Barbara D. Alexander ^4^, Marisa H. Miceli ^5^, Pappas Peter ^6^, Jeanette Jiang ^7^, Yi Song ^7^ and George R. Thompson ^8^**

^1^ University of Pittsburgh Medical Center
^2^ McGovern Medical School
^3^ Oregon Health and Science University Hospitals and Clinics
^4^ Duke University
^5^ University of Michigan
^6^ University of Alabama at Birmingham
^7^ Astellas Pharma Global Development, Inc.
^8^ UC Davis Health

**Objectives:** To describe ‘real-world’ experience of MATs as prophylaxis for IFIs.

**Materials & Methods:** This sub-analysis of a multicentre, observational, prospective study of patients receiving MATs (isavuconazole, posaconazole, and voriconazole) as antifungal prophylaxis included data from 55 US centres, March 2017–April 2020. Patients receiving MATs were enrolled prospectively or within 60 days of MAT initiation, and grouped by monotherapy (remained on initial MAT) or switched therapies (≥1 MAT received at/after index/enrolment). The primary objective was to characterise the at-risk population of patients receiving MATs. Exploratory objectives included assessing MAT-related adverse drug reactions (ADRs) and breakthrough IFIs.

**Results:** 2009 patients receiving MATs were enrolled, of whom 1177 received MAT prophylaxis at index/enrolment and were included in the analysis; 925 remained on initial MAT (256 isavuconazole, 397 posaconazole, 272 voriconazole), and 252 switched therapies (data for patients who switched therapies were reported, but are only presented as part of total/overall results here). Most baseline demographics/characteristics were similar across groups (Table 1). Haematologic malignancy (76.5%), neutropenia (60.0%) and use of corticosteroids (60.2%) were the most frequent underlying risk/host factors at baseline. Almost half of patients (48.3%) received a non-MAT antifungal within 90 days before study index/enrolment, and 45.2% received concomitant non-MAT antifungal therapy on/after index/enrolment; most received fluconazole (241/1177 [20.5%]) or micafungin (223/1177 [18.9%]). Most patients completed their MAT therapies (892/1132 [78.8%]); data were missing in 45 patients. Mean duration of therapy (days) after enrolment was numerically longer for patients receiving isavuconazole (162.6) than for posaconazole (126.5) and voriconazole (90.7) groups. Discontinuation of MAT therapy due to lack of efficacy or ADRs was reported in 4.6% (52/1132) and 11.1% (126/1132) of patients, respectively. Numerically higher rates of ADRs were reported for voriconazole (27/267 [10.1%]) and posaconazole (30/368 [8.2%]) groups than for isavuconazole (5/245 [2.0%]). Breakthrough IFIs occurred in 7.1% (73/1030) of patients with an investigator’s assessment; incidence was numerically similar among isavuconazole (11/221 [5.0%]), posaconazole (20/374 [5.3%]), and voriconazole (9/226 [4.0%]) groups (Table 2). The risk of breakthrough IFIs was significantly increased in patients with concomitant bacterial (odds ratio [OR] 2.67, 95% confidence interval [CI] 1.59, 4.50, *p* = 0.0002) and viral (OR 1.99, 95% CI 1.20, 3.30, *p* = 0.0075) infections; other assessed demographic/baseline characteristics were not statistically significant. ORs of breakthrough infection for *Candida* species and *Aspergillus fumigatus* were 330.09 (95% CI 113.11, 963.34; *p* < 0.0001) and 44.91 (95% CI 13.05, 154.51; *p* < 0.0001), respectively, vs. no infection/pathogen. The incidence of IFI-specific mortality was low among all patients receiving prophylaxis (<2%).

**Conclusions:** This ‘real-world’ study showed variation in underlying risk factors across the prophylaxis groups, which was representative of at-risk patients. Breakthrough IFIs were uncommon in patients receiving MAT prophylaxis, regardless of MAT received. MAT discontinuation due to ADRs was uncommon, and numerically lower rates of ADRs were reported for isavuconazole compared with other MATs. Mortality among patients receiving prophylaxis was low for all MAT therapies.

## P367 *Candida albicans* vs. non-*albicans* Invasive Infections in a Tertiary Hospital—A 10-Year Microbiological Study

**Petros Ioannou ^1^, Stamatis Karakonstantis ^1^, Dimitra Stafylaki ^1^, Sofia Maraki ^1^, George Hamilos ^1^ and Diamantis P Kofteridis ^1^**

University Hospital Of Heraklion

**Objectives:** *Candida* species are common fungal opportunistic pathogens associated with significant mortality. Non-*albicans Candida* (NAC) are increasingly prevalent causes of candidiasis and some of them have reduced antifungal susceptibility; thus, treatment of these pathoges may be problematic. The aim of this study was to evaluate the species distribution of *Candida* species causing invasive disease in a tertiary hospital during a ten-year period and also describe the antifungal susceptibility of these species.

**Materials & Methods:** A retrospective study containing microbiological data from 2011 to 2020 from the University Hospital of Herakion, Heraklion, Crete, Greece, was conducted. Data included gender of patients, type of specimen identified, species distribution and antifungal susceptibility.

**Results:** In total, 536 cultures of *Candida* species from 480 patients were isolated. Among all patients, 287 (59.8%) were male. Distribution of specimens were as follows: 411 (77.6%) were from the blood, 91 (17%) were peritoneal fluid, 10 (1.9%) were cerebrospinal fluid, 8 (1.5%) were pleural fluid, 5 (0.9%) were pancreatic fluid, 4 (0.7%) were from transplant tissue, 1 (0.2%) was from synovial fluid and 1 (0.2%) was from lymph node. Distribution of *Candida* species was as follows: *C. albicans* 202 (37.7%) and NAC (62.3%) (and more specifically as shown in Table 1). Antifungal susceptibilty is shown in Table 2.

**Table 1.** Distribution of *Candida* species in regards to the year.




**2011**

**2012**

**2013**

**2014**

**2015**

**2016**

**2017**

**2018**

**2019**

**2020**

**2011–2020**

*C. albicans*
19 (44.2)19 (40.4)26 (46.4)14 (35.9)18 (41.9)23 (47.9)20 (30.3)20 (38.5)22 (30.6)21 (30)202 (37.7)*C.* non-*albicans*24 (55.8)28 (59.6)30 (53.6)25 (64.1)25 (58.1)25 (52.1)46 (69.7)32 (61.5)50 (69.4)49 (70)334 (62.3)
*C. parapsilosis*
12 (27.9)10 (21.3)19 (33.9)16 (41)12 (27.9)12 (25)18 (27.3)15 (28.8)26 (36.1)33 (47.1)173 (32.3)
*C. glabrata*
4 (9.3)8 (17)4 (7.1)3 (7.7)5 (11.6)3 (6.3)11 (16.7)6 (11.5)14 (19.4)13 (18.6)71 (13.2)
*C. tropicalis*
4 (9.3)4 (8.5)5 (8.9)5 (12.8)4 (9.3)8 (16.7)12 (18.2)6 (11.5)8 (11.1)1 (1.4)57 (10.6)
*C. krusei*
2 (4.7)2 (4.3)0 (0)0 (0)0 (0)1 (2.1)0 (0)1 (1.9)0 (0)1 (1.4)7 (1.3)Other species of *Candida* *2 (4.7)4 (8.5)2 (3.6)1 (2.6)4 (9.3)1 (2.1)5 (7.6)4 (7.7)2 (2.8)1 (1.4)26 (4.9)
* *C. lusitaniae, C. pelliculosa, C. famata, C. guilliermondii, C. kefyr, C. lipolytica, C. dubliniensis, C. ciferrii*.


**Table 2.** Resistance of *Candida* strains to antifungals.



**Strain**

**AMB**

**Caspo**

**Fluco**

**5-FC**

**Mica**

**Vori**

*C. albicans*
S 196 (97%) I 4 (2%) R 2 (1%)S 138 (100) I 0 (0%) R 0 (0%)S 199 (98.5%) I 0 (0%) R 3 (1.5%)S 199 (98.5%) I 1 (0.5%) R 2 (1%)S 136 (98.6%) I 1 (0.7%) R 1 (0.7%)S 200 (99%) I 0 (0%) R 2 (1%)
*C. parapsilosis*
S 169 (98.3%) I 1 (0.6) R 2 (1.2%)S 132 (100%) I 0 (0%) R 0 (0%)S 171 (98.8%) I 0 (0%) R 2 (1.2%)S 170 (98.3%) I 1 (0.6%) R 2 (01.2%)S 132 (100%) I 0 (0%) R 0 (0%)S 170 (98.8%) I 1 (0.6%) R 1 (0.6%)
*C. glabrata*
S 69 (97.2%) I 2 (2.8%) R 0 (0%)S 44 (80%) I 8 (14.5%) R 3 (5.5%)S 56 (78.9%) I 3 (4.2%) R 12 (16.9%)S 71 (100%) I 0 (0%) R 0 (0%)S 55 (100%) I 0 (0%) R 0 (0%)S 62 (87.3%) I 2 (2.8%) R 7 (9.9%)
*C. tropicalis*
S 57 (100%) I 0 (0%) R 0 (0%)S 44 (100%) I 0 (0%) R 0 (0%)S 57 (100%) I 0 (0%) R 0 (0%)S 57 (100%) I 0 (0%) R 0 (0%)S 44 (100%) I 0 (0%) R 0 (0%)S 57 (100%) I 0 (0%) R 0 (0%)
*C. krusei*
S 7 (100%) I 0 (0%) R 0 (0%)S 2 (66.7%) I 1 (33.3%) R 0 (0%)S 1 (20%) I 0 (0%) R 5 (80%)S 1 (14.3%) I 3 (42.9%) R 3 (42.9%)S 3 (100%) I 0 (0%) R 0 (0%)S 7 (100%) I 0 (0%) R 0 (0%)Other species of *Candida* *S 26 (100%) I 0 (0%) R 0 (0%)S 18 (100%) I 0 (0%) R 0 (0%)S 25 (100%) I 0 (0%) R 0 (0%)S 26 (100%) I 0 (0%) R 0 (0%)S 18 (100%) I 0 (0%) R 0 (0%)S 26 (100%) I 0 (0%) R 0 (0%)
5-FC: 5-flucytosine; AMB: amphotericin B; Caspo: caspofungin; Fluco: fluconazole; I: intermediate; Mica: micafungin; R: resistant; S: sensitive; Vori: voriconazole * *C. lusitaniae. C. pelliculosa. C. famata. C. guilliermondii. C. kefyr. C. lipolytica. C. dubliniensis. C. ciferrii*.


**Conclusions:** In this study we show that in a ten-year period of study in a Greek tertiary hospital, the most common causes of invasive candidiasis were NAC, followed by *C. albicans*, with NAC being increasingly prevalent the recent years. Antifungal susceptibility showed significant resistance of *C. glabrata* and *C. krusei*, while most of the other species had minimal antifungal resistance.

## P368 In Vitro Susceptibilities of Mucormycosis Etiologic Agents to Amphotericin B and Posaconazole

**Natalia Vasilyeva, Irina Vybornova, Sergey Kovyrshin, Tatiana Bogomolova, Galina Chilina and Ilya Bosak**

North-Western State Medical University n.a. I.I. Mechnikov

**Background:** Mucormycosis is an emerging disease in immunocompromised patients. Clinical breakpoints of antifungals used for treatment of patients with mucormycosis are not yet established. The information about minimum inhibitory concentrations (MICs) of antifungal drugs for clinical isolates of mucormycetes may contribute to the development of clinical breakpoints. The aim of the study was to investigate in vitro susceptibilities to amphotericin B and posaconazole of mucormycetes isolates from patients in Saint Petersburg, Russia.

**Materials & Methods:** A total of 29 molecularly identified strains of the Mucorales were studied: *Lichtheimia* spp.—10 (*L. corymbifera*—5, *L. ramosa*—4, L. ornata—1), Rhizopus spp.—10 (*R. arrhizus*—8, *R. microsporus*—2), *Rhizomucor pusillus*—7, *Mucor* spp.—2 (*M. circinelloides*—1, *M. racemosus*—1). All isolates are deposited into Russian Collection of Pathogenic Fungi (RCPF). Antifungal susceptibility tests were performed by the broth microdilution technique according to EUCAST Definitive document E.DEF 9.3.2. The two-tailed Mann-Whitney test was used for statistical analysis.

**Results:** Posaconazole MICs (mg/L) ranged between 0.03 and 0.5 for *Lichtheimia* spp. isolates, 0.125 and 0.5 for *Rhizopus* spp., 0.06 and 0.125 for *R. pusillus*, 0.125 and 0.25 for *Mucor* spp.

Amphotericin B MICs (mg/L) ranged between 0.03 and 0.5 for *Lichtheimia* spp. isolates, 1 and 4 for *Rhizopus* spp. and *R. pusillus*, 0.25 and 0.5 for *Mucor* spp. (Table 1).

MICs of amphotericin B were significantly higher for *Rhizopus* spp. and *R. pusillus* compared to *Lichtheimia* spp. isolates (*p* = 0.00001 and *p* = 0.0001, respectively).

**Conclusions:** In vitro susceptibility to posaconazole did not depend on the genus of the etiologic agent of mucormycosis. *Lichtheimia* spp. isolates were more susceptible to amphotericin B in vitro in comparison to *Rhizopus* spp. and *R. pusillus*.

## P369 Antifungal Susceptibility and Virulence Traits of Clinical Isolates of *Candida guilliermondii* and the Closely Related Species, *Candida fermentati*

**Elena Palencia-Mulero, Katherine Miranda-Cadena, Cristina Marcos-Arias, Esther Tamayo, Andrea Guridi, Elena Sevillano, Elena Eraso and Guillermo Quindós**

Department of Inmunology, Microbiology and Parasitology, Faculty of Medicine and Nursery, University of the Basque Country (UPV/EHU)

**Introduction and objectives:** Candidiasis caused by *Candida guilliermondii* complex (*Candida guilliermondii, Candida fermentati* and *Candida carpophila*) are of clinical interest given the reduced antifungal susceptibility of these species. Furthermore, information about its virulence traits is scarce.The aim of the study was to determine the in vitro antifungal susceptibility and to characterize phopholipase and proteinase activities of *C. guilliermondii* and *C. fermentati* clinical isolates.

**Materials & Methods:** A total of 64 clinical isolates recovered from patients suffering from superficial or invasive candidiasis were analyzed. Isolates were previously identified by conventional mycologycal methods and re-identified by sequencing of the ITS region and ACT1 gene. In vitro antifungal susceptibility testing was performed by the EUCAST E. Def 7.3.2 microdillution method for amphotericin B, fluconazole, anidulafungin and micafungin. No clinical breakpoints have been established for *C. guilliermondii* species complex so susceptible/intermediate/resistant categories were not used, and only the epidemiological cutoff value (ECV) for *C. guilliermondii* to fluconazole (>16 μg/mL) was considered. Geometric mean, mode, MIC50, MIC90, and range were calculated. Protease and phospholipase production was assessed by agar plate methods.

**Results:** The antifungal susceptibility of 62 *Candida guilliermondii* isolates and two *Candida fermentati* isolates to amphotericin B, fluconazole, anidulafungin and micafungin is presented in the table.


**Antifungals**

***C. guilliermondii* (n = 62)**

***C. fermentati* (n = 2)**

**MIC50 (μg/mL)**

**MIC90 (μg/mL)**

**MIC Range (μg/mL)**

**GM**

**(μg/mL)**

**MIC50 (μg/mL)**

**MIC90 (μg/mL)**

**MIC Range (μg/mL)**

**GM (μg/mL)**
Amphotericin B0.250.250.06–0.50.177--0.125–0.250.125Fluconazole8160.125–647.655--22Micafungin0.250.50.008–0.50.228--0.1250.125Anidulafungin0.250.50.008–10.304--0.250.25

Four isolates of *C. guilliermondii* were classified as non wild-type isolates according to ECVs. The MIC of fluconazole against two of them was 32 µg/mL, one isolate was associated with oral candidiasis and the other one with diaper dermatitis. The remaining two isolates that showed a MIC of 64 µg/mL were also associated with an oral candidiasis and with a diaper dermatitis.

Regarding to virulence characteristics, five *C. guilliermondii* isolates (8.1%) were classified as moderate phospholipase producers, all of them being isolated from non-sterile sites, while none of the *C. fermentati* isolates were producers. Interestingly, one non wild-type *C. guilliermondii* isolate (MIC 32 μg/mL) was phospholipase producer and was associated to diaper dermatitis. Neither *C. guilliermondii* nor *C. fermentati* isolates were proteinase producers.

**Conclusions:** Amphotericin B and echinocandins were the most active antifungal drugs against *C. guilliermondii* and *C. fermentati*. Less than 7% of *C. guilliermondii* isolates showed resistance to fluconazole. Phospholipase production was observed in one non wild-type isolate associated to diaper dermatitis and could play a role in enhanced virulence.

**Funding:** GIC15/78 IT-990-16 (Gobierno Vasco-Eusko Jaurlaritza).

## P370 Disk Diffusion Susceptibility Testing of *Malassezia pachydermatis* and *Candida* spp. Isolated from Animals against Three Most Commonly Used Antifungal Drugs

**Suzana Hadina ^1^, Vesna Mojcec Perko ^1^, Ana Cicmak ^2^, Zrinka Stritof ^1^, Josipa Habus ^1^, Vilim Staresina ^1^, Iva Benvin ^1^, Vladimir Stevanovic ^1^, Matko Perharic ^1^, Kresimir Martinkovic ^1^, Iva Zecevic ^1^ and Ljiljana Pinter ^1^**

^1^ Department of Microbiology and Infectious Diseases with Clinic, Faculty of Veterinary Medicine, University of Zagreb
^2^ Department for Parasitology and Mycology, Croatian Institute for Public Health

**Objectives:** Malassezia yeasts are considered to be the inhabitants of skin microbiota of healthy animals and humans accompanied by Candida. Under certain conditions, they could become opportunistic pathogens where the most frequently isolated species is *M. pachydermatis*, causing otitis and dermatitis, mainly in dogs. *Candida* species might occasionally cause not only ear or skin infections, but also systemic infections in cats, dogs, horses, cattle, pigs, parrots and other animal species. In the past decade there is an increase in the number of reports of *Candida* spp. resistance and reduced susceptibility of *M. pachydermatis* in veterinary medicine. In addition, several case reports documented zoonotic transmission of *M. pachydermatis* to immunocompromised individuals. Taken together, these facts point to growing concerns about fungal resistance in human and veterinary medicine and suggest the need of regular antifungal susceptibility testing of human and animal isolates. The aim of this study was to examine the antifungal susceptibility of *M. pachydermatis* and *Candida* isolates originating from different animal samples submitted to Mycology Laboratory of the Faculty of Veterinary Medicine in Croatia. Disk diffusion test was used for screening of susceptibility of isolated species against most commonly used antifungal drugs in clinical settings: miconazole (MCZ) and clotrimazole (CTZ) for topical treatment and itraconazole (ITZ) for systemic therapy.

**Methods:** Swab samples were plated on Sabouraud dextrose agar (SDA) with the addition of chloramphenicol. *M. pachydermatis* strains were identified by conventional laboratory methods and their ability to grow on SDA without lipid supplementation. The identification of *Candida* isolates was based on their phenotypic characterization and molecular methods using the universal ITS-1 and ITS-4 primers for amplification of the internal transcribed spacer 1 (ITS1), ITS2 regions and the 5.8S ribosomal DNA region followed by sequence-based identification. Susceptibility of *Candida* isolates was determined as described by the CLSI M44-A2 method using MCZ, CTZ and ITZ discs (10 µg, Neo-Sensitabs, Rosco, Denmark). Because the standard procedure for *Candida* susceptibility testing cannot be applied to *M. pachydermatis*, recommended modified protocol was used. The results were interpreted according to manufacturer’s guidelines.

**Results:** A total of 175 *M. pacyhdermatis* and 10 Candida species (four *C. albicans*, two *C. tropicalis*, two *C. palmioleophila*, one *C krusei* and one *C. lusitaniae)* were recovered from different animal species. Using modified disk diffusion test one isolate of *M. pachydermatis* showed reduced susceptibility to all three antifungal drugs tested. In addition, one isolate showed reduced susceptibility to CTZ, and three isolates to MCZ. All *Candida* isolates were susceptible to CTZ, five showed simultaneously resistance to MCZ and ITZ, and two isolates to ITZ alone. In both yeast species, the highest susceptibility was detected to CTZ. Despite the small sample size, the fungal susceptibility pattern showed that Candida isolates were more frequently resistant to tested antifungal drugs.

**Conclusions:** Results of this study showed the presence of potentially resistant yeast isolates from animals. In order to obtain a detailed insight into their resistance pattern and the appropriate choice of antifungal treatment, further tests using broth microdilution method need to be performed.

## P371 Methionine Synthase Downregulation Reduces *Aspergillus fumigatus* Virulence in Models of Established Infection

**Jennifer Scott ^1^, Monica Sueiro-Olivares ^1^, Riba Thomas ^1^, Rachael Fortune-Grant ^1^, Elaine Bignell ^2^ and Jorge Amich ^1^**

^1^ University of Manchester
^2^ University of Exeter

**Objectives:** Invasive fungal infections, such as those caused by *Aspergillus fumigatus*, have mortality rates of ~50% even under antifungal treatment. Current therapeutic options are severely limited and rising antifungal resistance further exacerbates the desperate need for novel drugs. Methionine synthase (MetH) is essential for *A. fumigatus* viability and virulence, however its potential as a target in established infections has not previously been explored.

This work aimed to further validate MetH as an antifungal drug target by developing in vivo models of infection that mimic treatment of an established *Aspergillus* infection. The objectives were:

1. To optimise a genetic model of target downregulation in established *A. fumigatus* infections in vivo.
2. To discover whether *metH* downregulation reduces virulence in established infections.
3. To examine whether MetH present a viable antifungal target, comparable to the target of the azoles.

**Materials & Methods:** We constructed an *A. fumigatus* strain that expresses *metH* under the regulatable control of the tetOFF system (*metH_tetOFF*), whereby the addition of doxycycline (Dox) inhibits target expression. We also produced a control strain in which the target of the azoles is regulated by the tetOFF system (*cyp51A_tetOFF cyp51B*Δ). We characterised the effect of downregulating these targets in vitro by phenotypic and microscopic analyses. To validate the targets in established infections in vivo two models were used: *Galleria mellonella* and leukopenic mice.

**Results:** Shutting down methionine synthase expression in vitro prevented mycelial development and microscopic observations confirmed that growth was halted in a rapid and sustained manner. In *Galleria*, Dox administration resulted in a significant reduction in mortality, which was similar for both tetOFF strains. Finally, in established infections of leukopenic mice, fungal burden was significantly reduced for both strains following Dox administration. In both models the Dox regimen could not completely abrogate *A. fumigatus*’ infective capacity, however the degree of reduction was similar between our target and that of the gold standard antifungals.

**Conclusions:** The rigorous Dox regimen necessary to achieve concentrations capable of target downregulation proved toxic in both infection models, preventing the examination of survival in mice and therefore limiting the usefulness of the tetOFF system in vivo. Yet, the inclusion of the target of the azoles permitted the constraints of the model to be overcome and confirmed that the small reduction in virulence was significant. Therefore, we propose that methionine synthase is a promising antifungal target as its downregulation results in reduced virulence, comparable to targeting *cyp51A*, in two in vivo models of established infection.

## P372 The Gut and Upper Respiratory Tract Mycobiota in Cystic Fibrosis Infants

**Luiza Rodrigues ^3^, Jannaina Vasco ^1^, Carlos Riedi ^1,2^, Eduarda Lazzarotto ^1^ Lilian Pereira-Ferrari ^1^, Nelson Rosário-Filho ^1,2^, Rafael Oliveira ^4^ and Wellington Omori ^4^**

^1^ Post-Graduate Program in Child and Adolescent Health, Federal University of Paraná (UFPR)
^2^ Department of Pediatrics, Federal University of Paraná (UFPR)
^3^ Instituto de Pesquisa Pelé Pequeno Príncipe
^4^ Neoprospecta Microbiome Technologies

**Objectives:** Gut and respiratory microbial colonization are known to be different in infants, children and adults. Mutations in the CFTR gene modify the intestinal and airway microenvironment, changing the microbiota of patients with cystic fibrosis (CF). The mycobiota is an important component of the human microbiota, however, research for mycobiota in patients with CF is still incipient. Our aim was to characterize the upper respiratory tract and gut mycobiota in infants diagnosed with CF.

**Materials & Methods:** This is a prospective observational cohort study, with cross-sectional and longitudinal components to sample acquisition and data analysis. We analyzed stool samples and oropharyngeal swabs every three months, during 9 months, from five clinically stable patients with CF of 7 months of age (diagnosed by newborn screen, sweat chloride test, and confirmatory genetic testing). The participating patients included 3 homozygotes and 2 heterozygotes with the delF508 mutation. The study protocol was approved by the Human Research Ethics Committee of Complex Hospital of Clinics—Federal University Paraná (CHC–UFPR), Curitiba-PR, Brazil (CEP-HC/UFPR ref no: 1.948.265). DNA was obtained using a magnetic beads methodology (Neoprospecta Microbiome Technologies, Brazil). Metagenomic libraries were prepared using ITS1 5′- GAACCWGCGGARGGATCA -3′ and ITS2 5′- GCTGCGTTCTTCATCGATGC-3′ primers to amplify ITS1 region, alongside negative PCR controls and a synthetic mock microbial community in which the composition and relative abundances of community members are known. The sequences were analyzed using a proprietary pipeline (Sentinel, Neoprospecta Microbiome Technologies, Brazil).

**Results:** Overall, 30 samples were collected and analyzed: 15 stool samples and 15 oropharyngeal swabs. Amplicon sequence data from stool samples generated 479,421 raw reads. Of these, 4669 reads were clustered into OTUs and 119 OTUs were taxonomically classified. While, respiratory samples generated 903,751 raw reads. Of these, 12,593 reads were clustered into OTUs and 358 OTUs were taxonomically classified. Only two phyla (*Ascomycota* and *Basidiomycota*) were detected in the gut and upper respiratory tract mycobiota. *Ascomycota* was the most abundant fungi phylum present in all samples, while *Basidiomycota* was detected in only two gut samples and six respiratory samples. In the gut mycobiota, *Aspergillus, Cladosporium, Clavispora, Debaryomyces, Saccharomyces, Tricholoma* and *Wallemia* generas were detected and, in the respiratory mycobiota, besides these, *Candida, Fusarium* and *Trichosporon*. Most stool samples (12/15) were dominated by one or two fungal genera (Figure 1). In general, respiratory samples showed greater fungal diversity compared to stool samples (Figure 2).

**Conclusions:** Here, we describe some differences between gut mycobiota of infants with CF and previously published data on healthy infants, which generally contained *Candida* spp, *Cryptococcus* and *Malassezia* spp. In addition, we detected greater fungal diversity in the upper respiratory tract mycobiome compared to the gut mycobiome. In all patients, in at least one respiratory sample, we found *Aspergillus* spp. Evaluating the formation and dynamics of the microbial community, including fungi, in the stable CF patient is crucial to understanding their relevance for the clinical course of pulmonary disease. Future studies are needed to better correlate the inter-kingdom interactions and inter-organ communication to fill this gap in CF pathophysiology.

## P373 Predicting Mortality Using the EQUAL Candida Score in Patients with Candidemia at a Tertiary Care Center in Lebanon

**Aline El Zakhem and Rachid Istambouli**

American University of Beirut Medical Center

**Objectives:** The European Confederation of Medical Mycology (ECMM) developed the ECMM Quality of Clinical Candidemia Management (EQUAL) Candida to assess the adherence to the latest guidelines in both the diagnosis and the management of candidemia, and to highlight the most important aspects of the current recommendations. Very few studies have evaluated the utility of this score in predicting 30-day mortality due to candidemia. We aim to evaluate the usefulness of the EQUAL Candida score for the prediction of 30-day mortality in our patient population, and to assess the ability of its individual items to predict mortality in these patients.

**Materials & Methods:** This is a single-center retrospective chart review on adult patients with blood culture-proven candidemia admitted to a teriary care center in Lebanon from 2004 till 2019. Patients who died within 24 h of blood culture withdrawal were excluded. Data on patient characteristics, including age, sex, underlying co-morbid conditions and known risk factors for candidemia were collected. The management of candidemia was assessed using the EQUAL Candida score, and points were allocated accordingly (Table 1). Univariate analyses for comparisons between survivors and non-survivors were done using the *t*-test for continuous variables and the Chi-square test for categorical variables, and logistic regression was used to adjust for confounding variables.

**Results:** A total of 142 patients were included in the study, of which 52% were male, 66% were immunocompromised, 44% patients were critically ill and 71% had a central venous catheter (CVC). The most common identifiable etiology of candidemia was gastrointestinal translocation, whereas around one third (30%) of the episodes were of uknown etiology. Thirty-day mortality was 43.7% and both admission to a critical care unit and immunocompromise were associated with higher mortality (*p* = 0.002 and 0.003). In the total population, there was no difference (*p* = 0.256) between the mean EQUAL score in survivors (8.84) and in non-survivors (8.03). When patients with a CVC were considered alone, survivors had a significantly higher (*p* = 0.022) mean EQUAL score (10.11) as compared to non-survivors (8.25). This association remains significant after adjusting for critical illness and immunocompromise (*p* = 0.016). Susceptibility testing and echocardiography in this subpopulation were independently associated with reduced mortality (*p* = 0.013 and 0.009, respectively). In non-CVC carriers however, the EQUAL score is not associated with mortality (*p* = 0.456).

**Conclusions:** The EQUAL Candida score can predict 30-day mortlaity for CVC carriers in our population and can be used by clinicians as a tool to guide proper management.

## P374 The Pulmonary Mycobiome and Its Relationship with The Environment

**Consuelo Ferrer ^1^, Esther Rubio-Portillo ^2^, Beatriz Galvez ^3^, Violeta Esteban ^4^, Marc Mestre ^1^, Elena Castro ^1^, Laura Perez-Martin ^1^, Elisabet Perea ^1^, Carlos Fernandez-Mauricio ^2^, Eusebi Chiner ^4^, Cleofe Fernandez ^5^, Jose Norberto Sancho ^4^, David Orts ^6^, Eleuterio Llorca ^6^, Josefa Anton ^2^ and Francisca Colom ^1^**

^1^ Institute for Healthcare and Biomedical Research of Alicante (ISABIAL), University Miguel Hernández
^2^ Department of Physiology, Genetics and Microbiology, University of Alicante
^3^ Pneumology Department, Vinalopó University Hospital
^4^ Respiratory Department, University Hospital of San Juan
^5^ Respiratory Department, Institute for Healthcare and Biomedical Research of Alicante (ISABIAL), General University Hospital
^6^ Pneumology Service, Elda General University Hospital

**Objectives:** The aim of the study is to analyze the relationship between the mycobiome of the Lower Respiratory Tract (LRT) and the fungi in the domestic environment of the patients.

**Materials & Methods:** Samples consisted in Broncho- Alveolar Lavage (BAL) from 79 patients who underwent bronchoscopy for different diagnostic purposes, and dust and air from the houses (ENV) of 25 of them (31.7%). Inclusion criteria were: adult patients not suffering from any infectious disease and not receiving antimicrobial therapy in the last 4 weeks; immunological status was also taken in account. All samples were processed for DNA extraction and cultures, which were performed in Sabouraud and Potato Dextrose agar for general fungi, and Dixon modified agar for lipophylic yeasts. The fungal Internal Transcribed Spacer (ITS2) was sequenced by the Solexa/Illumina system and sequences were analyzed by QIIME 1.8.0 and compared with the UNITE Database for identification. Additionally, specific nested-PCR and RT-PCR were perfomed for the detection of *Pneumocystis jirovecii*.

**Results:** The mycobiome of ENV samples was richer but less diverse than the one from the LRT. Fungal species, such as Rhodotorula mucilaginosa, Candida parapsilosis, Naganishia albida, Malassezia restricta, Aureobasidium pullulans, Cryptococcus neoformans sl. and Cladosporium sp., were the most prevalent in both environments studied.

The percentage of coincidence of specific OTUs in the two communities (BAL and ENV) was about 75% of co-ocurrence. Some fungi are more prevalent in lungs (*Rh. mucilaginosa, Cr. neformans* and *Aspergillus* spp.) while others are more abundant in the air or dust (e.g., *M. restricta* and *Cladosporium* sp.). *Pneumocystis jirovecii* was detected in 4.8% of BAL samples but in none of the environmental ones.

Most of species detected by DNA-sequencing were cultured from ENV samples, where abundant growth was obtained. However, from lung samples, species were only recovered from 46.7% of the samples cultured on SDA and 39.7% of the cultures on DIxon. In all cases with a very low number of isolates.

In this study, as well as in many others preformed with the same molecular approach, a significant number of OTUs could not be related to any specific fungal taxon through the UNITE and other databases at 97% similarity.

**Conclusions:** Our results confirm that the environment could be the main determinant of the lung mycobiome, and that the health condition of individuals, specifically their inflammatory status, may influence the diversity and richness of the mycobiota more than its microbial composition.

The mycobiome of the lower respiratory tract and the domestic environment share an important percentage of components. It seems that there is a reciprocal influence of the two environments and fungi inhaled from indoor air contribute to the composition of the human mycobiome and, in the same way, the human mycobiome may influence the indoor environmental mycobiota, which also receives most of its members from outdoors. Cultures confirmed the presence of the core mycobiome species in most dust samples but, the low rate of isolation from BAL suggests that most of its mycobiome corresponds to non-culturable cells.

## P375 Phylogenomics Reveal *Aspergillus fumigatus* Lineage Is Associated with Respiratory Disease

**Renad Aljohani ^1^, Andrew Scourfield ^2^, Johanna Rhodes ^2^ and Darius Armstrong-James ^1^**

^1^ Department of Infection Diseases, Imperial College London
^2^ MRC Centre for Global Infectious Disease Analysis, Department of Infectious Disease Epidemiology, St. Mary’s Campus, Imperial College London
^3^ Hospitals NHS Foundation Trust, University College London

**Objectives:** *Aspergillus fumigatus* is a ubiquitous saprophytic airborne fungus and the major cause of life-threatening invasive aspergillosis. *A. fumigatus* respiratory infection is a common complication of cystic fibrosis and is associated with allergic disease and loss of pulmonary function. *A. fumigatus* is found in several environmental niches that contribute to its genotypic and phenotypic heterogeneity among isolates. Given the increasing worldwide emergence of azole-resistant *A. fumigatus*, understanding how antifungal resistance mechanisms evolve in the host environment during infection is of great clinical importance and biological interest. Hence, determining the relationships between the diverse *A. fumigatus* genomes and their numerous biological and phenotypic characteristics, such as their range of pathogenicity and antifungal resistance, is a significant research challenge.

Some *A. fumigatus* phylogenetic groups are responsible for different respiratory infections, and many lineages are resistant to azole antifungal drugs. Therefore, our aim in this research was to investigate the phylogenetic relationship of clonal azole-resistant *A. fumigatus* strains isolated at Royal Brompton Hospital (London, UK), and to determine whether different *A. fumigatus* lineages are associated with different types of infection (e.g., bronchiectasis, asthma/allergic bronchopulmonary aspergillosis, cystic fibrosis).

**Materials & Methods:** All 98 isolates in this study were obtained from the Royal Brompton Hospital and stored at −80 °C at the Flowers Building, South Kensington. All genomes were sequenced with Illumina HiSeq and stored at the Research Computing Service, South Kensington (Imperial College London). We performed genome-scale comparisons of single nucleotide polymorphisms (SNPs) of each isolate vs. a selected reference isolate. The maximum likelihood phylogenetic tree was constructed using the Randomised Axelerated Maximum Likelihood v8.2.9 algorithm, with 1000 iterations for bootstrap support, and visualised using FigTree v1.4.4.

**Results:** Our preliminary results for the phylogenetic relationship tree analysis showed that *cyp51A* resistance mechanisms occurred in 11 azole-resistant *A. fumigatus* isolates from 56 patients with cystic fibrosis. Seven isolates harboured TR_34_/L98H, and three were point mutations in the cyp51A gene, leading to L98H/M220K substitution. These mutations are known to be correlated with azole resistance. Mixed infections (wildtype/non-wildtype) and a case of potential additional tandem-repeat expansion in vivo were detected. The *A. fumigatus* isolates were grouped into two independent Clades, A and B, distinguished by different numbers of SNPs. Isolates that harboured the drug resistance polymorphism TR_34_/L98H were primarily clustered in clade A, while wildtype/non-wildtype isolates were clustered in Clade B.

**Conclusions:** Overall, Phylogenetic analysis showed that the majority of azole resistance polymorphisms clustered into one group, suggesting evolution of azole resistance on a specific genetic background.

Even distributions of the resistance-related genotype and evolutionary genetic variability changes were apparent in both clades in some isolates from a single patient, suggesting infection with a broad range of genotypes.

## P376 Role of the ABC Transporter CDR1 in the Azole Resistance of *Candida lusitaniae*

**Valentin Borgeat ^1^, Danielle Brandalise ^1^, Frédéric Grenouillet ^2^ and Dominique Sanglard ^1^**

^1^ University of Lausanne and University Hospital Center
^2^ Université de Franche-Comté

**Objectives:** *Candida lusitaniae* is an opportunistic pathogen in human that causes infrequent but difficult-to-treat diseases. Antifungal drugs are used in the clinic to treat *C. lusitaniae* infections, however this fungus can rapidly acquire antifungal resistance to all known antifungal drugs (multidrug resistance)^1^. Notably, *C. lusitaniae* acquires azole resistance by gain-of-function (GOF) mutations in the transcriptional regulator *MRR1*^2^. *MRR1* controls the expression of a major facilitator transporter (*MFS7*) that is important for fluconazole resistance. The deletion of *MFS7* in a clinical isolate exhibiting a *MRR1* GOF mutation decrease fluconazole MIC but not to the levels of a wild type isolate, suggesting that *MRR1* controls other mediators of azole resistance^2^. Here we addressed the role of the ABC transporter *CDR1* as additional mediator of azole resistance in *C. lusitaniae*.

**Materials & Methods:** *C. lusitaniae* isolates were described previously^2^. DSY4941 is a novel clinical fluconazole-resistant isolate with a A654V GOF mutation in *MRR1*. MIC assays were performed with a commercial YeastOne colorimetric microdilution method. Rhodamine 6G (R6G) efflux experiments were performed as described^3^. *CDR1* deletions were carried out by Cas9/CRISPR-based approaches.

**Results:** *CDR1* expression in isolates with GOF *MRR1* mutations V688G^2^ and A654V was evaluated by qPCR and was 16- and 8-fold higher, respectively, as compared to wild type. This suggests that *CDR1* is an additional (direct or indirect) target of *MRR1. CDR1* was next deleted in the azole-resistant isolate P3 (V688G GOF) as well as P1 (wild type *MRR1*) and several mutant derivatives lacking *MFS7* and/or *MRR1*^2^. *CDR1* deletion in P3 revealed that MICs of long-tailed azoles, itraconazole and posaconazole, were decreased by 16-fold as compared to P3, which is consistent with the role of this ABC-transporter in efflux of these azoles. Fluconazole MIC was only decreased when *CDR1* was deleted in the background of a *mfs7*∆ mutant from P3, which underpins the dominant role of *MFS7* in the resistance of the short-tailed fluconazole. With R6G efflux readout as *CDR1* efflux capacity, our data showed that *CDR1* efflux was increased in P3, and diminished to background levels in all mutant strains lacking *CDR1*. Milbemycin oxim A3 (Aox3), one known inhibitor of *CDR1*^4^, mimicked efflux phenotypes of *cdr1*∆ mutants.

**Conclusions:** In this work, we provided evidence that *CDR1* is an additional mediator of azole resistance in *C. lusitaniae*, especially of long-tailed azoles. *CDR1* regulation is dependent on *MRR1* and associated GOF mutations, which is reminiscent of the regulatory node described in the related species *Candida auris*^5^.

**References**

1. Asner, S.A.; Giulieri, S.; Diezi, M.; Marchetti, O.; Sanglard, D. *Antimicrob. Agents Chemother.* **2015**, *59*, 7715–7722.
2. Kannan, A.; et al. *mBio* **2019**, *10*, 1227–1221.
3. Coste, A.; et al. *Genetics* **2006**, *172*, 2139–2156.
4. Silva, L.V.; et al. *Antimicrob. Agents Chemother*. **2013**, *57*, 873–886.
5. Li, J.; et al. *Antimicrob. Agents Chemother*. **2021**, *65*, e02663-20.

## P377 Cross-Talk between Calcineurin and the Cell Wall Integrity Pathways Prevents Chitin Overexpression and Loss of Fungal Viability

**Alessandra Da Silva Dantas ^1^, Filomena Nogueira ^2^, Keunsook K. Lee ^2^, Louise A. Walker ^2^, Matt Edmondson^1^, Alexandra C. Brand ^1^, Megan D. Lenardon ^3^ and Neil A. R. Gow ^1^**

^1^ MRC Centre for Medical Mycology, School of Biosciences, University of Exeter
^2^ School of Medical Sciences, Institute of Medical Sciences, University of Aberdeen
^3^ School of Biotechnology and Biomolecular Sciences, University of New South Wales

Echinocandins such as caspofungin are front line antifungal drugs that compromise β-1,3 glucan synthesis in the cell wall. Recent reports have shown that fungal cells can resist killing by caspofungin by up-regulation of chitin synthesis, thereby sustaining cell wall integrity. When echinocandins are removed, the chitin content of cells quickly returns to basal levels, suggesting that there is a fitness cost associated with having elevated chitin in the cell wall. We show here that simultaneous activation the calcineurin and CWI pathways generates a sub-population of cells that have supra-normal chitin levels interspersed throughout wall, and that these cells are non-viable, perhaps due to loss of wall elasticity required for growth. Mutations in the Ca^2+^-calcineurin pathway prevented the formation of these non-viable super high chitin cells by negatively regulating chitin synthesis driven by the CWI pathway. The Ca^2+^-calcineurin pathway therefore acts as an attenuator that prevents the overproduction of chitin cells by coordinating both chitin upregulation and negative regulation of the CWI signaling pathway.

## P378 In Vitro Antifungal Combination of Terbinafine with Itraconazole against Clinical Isolates of *Trichophyton* spp.

**Anne-Laure Bidaud ^1,2^, Patrick Schwarz ^3,4^, Anuradha Chowdhary ^5^ and Eric Dannaoui ^1,2^**

^1^ Hôpital Européen G. Pompidou
^2^ Université de Paris
^3^ Department of Internal Medicine, Respiratory and Critical Care Medicine, University Hospital Marburg
^4^ Center for Invasive Mycoses and Antifungals, Philipps University Marburg
^5^ Department of Medical Mycology, Vallabhbhai Patel Chest Institute, University of Delhi

**Objectives:** Terbinafine is used as first-line therapy against dermatophyte infections, but the incidence of terbinafine-resistance is increasing. Although, there exist no clinical breakpoints for terbinafine, many isolates exhibited high in vitro MICs. For resistant isolates, mutations in the squalene epoxidase gene of *Trichophyton* are the most common mechanism of antifungal resistance.

The aim of this study was to evaluate the combination of terbinafine with itraconazole against clinical *Trichophyton* spp. isolates in vitro.

**Materials & Methods:** The combination has been tested against 9 terbinafine-susceptible and 7 terbinafine-resistant clinical *Trichophyton* spp. isolates from India. In vitro interactions were evaluated by a microdilution checkerboard technique based on the EUCAST reference method for antifungal susceptibility testing. The mode of interaction between terbinafine and itraconazole was determined by the fractional inhibitory concentration index (FICI). The FICI was interpreted as FICI ≤ 0.5 = synergy, FICI > 0.5–4 = no interaction, and FICI > 4.0 = antagonism.

**Results:** For the susceptible isolates, the MIC of the drugs alone ranged from 0.125 to 0.5 µg/mL and 0.03125 to 0.5 µg/mL, with geometric mean MICs of 0.30 µg/mL and 0.12 µg/mL for itraconazole and terbinafine, respectively. When tested in combination, synergistic interactions were observed for 4/9 isolates with FICI values from 0.3125 to 0.5 for the isolates exhibiting synergy. For the resistant isolates, the MIC of the drugs alone ranged from 0.0625 to 0.5 µg/mL and 2 to 64 µg/mL, with geometric mean MICs of 0.19 µg/mL and 11.9 µg/mL for itraconazole and terbinafine, respectively. When tested in combination, synergistic interactions were observed for 4/7 isolates with FICI values from 0.032 to 0.3125 for the isolates exhibiting synergy. Overall, synergy was seen for 50% of the tested isolates, while antagonism was never observed.

**Conclusions:** These in vitro results warrant further investigation of the combination in animal models of dermatophytosis.

## P379 Electron Microscopic Examination of the Morphology of *Aspergillus* Isolated from the Semen of Sire Bulls

**Anna Arsenuk, Marina Manoyan, Vladimir Sokolov and Fatima Borunova**

The Russian State Center for Animal Feed and Drug Standardization and Quality, Fse Vgnki, Federal State Institution

**Objectives:** The main requirement for animal reproduction is strict observance of veterinary and sanitary measures aimed at preventing the emergence and spread of infectious diseases, including through genetic material during artificial insemination of animals. Among the many reasons that can cause infectious processes in animals is the microbial contamination of sperm by mycological agents.

The article presents the results of a study of the morphology of aspergillus isolated from the semen of breeding bulls. The morphology is shown. Differences in the structure of the spore surface were revealed.

**Materials & Methods:** In experimental studies, cryopreserved sperm samples from bovine bulls in straws were used. Straws with frozen semen were removed from liquid nitrogen using forceps, which ensured its storage in a frozen state for the entire period of transportation and storage prior to the start of the study, immersed in a water bath at 38 °C for 10 s, and left at room temperature until the samples were completely thawed. For the study, two Petri dishes with PDAC medium and two dishes with DRBC medium were used for each sample. The prepared samples were sterilely applied in 0.5 cm^3^ suspensions from two adjacent dilutions and the genetic material was evenly distributed over all agar surfaces. The crops were cultivated under aerobic conditions with lids up at a temperature of 28 °C for 5–14 days. The result was considered positive if the growth of micromycetes was observed on at least one Petri dish, provided that the dishes with the control media were clean.

To identify filamentous fungi, the structure of the fungus was studied on a micropreparation and the morphological features of the colony (including color, shape, intensity of growth into the substrate, the presence of folds, the color and structure of the reverse), as the presence of pigment secreted into the substrate.

Samples intended for electron microscopic examination were grown on membrane filters, fixed in vapors of 25% glutaraldehyde, mounted on sample holders, dehydrated twice with propylene oxide vapors, sprayed with gold ions, and viewed on Tescan VEGA || LMN and MAIA3 scanning electron microscopes. Due to the use of low accelerating voltages, it is possible to study the fine structures of the samples.

**Results and Conclusions:** Research results and discussion. 16 samples of sperm from breeding bulls were examined, the growth of fungi was observed in 7 samples out of 16, which amounted to 43.75%. The species composition of the isolated fungi was as follows: *Aspergilus niger* (57.14%) was found in 4 samples, *Aspergillus flavus* (14.29%) was found in 1 sample, and *Mucoraceae fungi* were found in 3 samples (42.8%). In addition, two fungi species were found in two samples, *Aspergilus niger* and *Mucor* spp. (28.57%).

In the central zone of the mycelium of *Aspergilus niger*, an accumulation of mature conidia, an intensively developing vegetative mycelium with a porous surface structure, adhesion of spores on the porous structure of hyphae of the vegetative mycelium were revealed (Figure 1C).


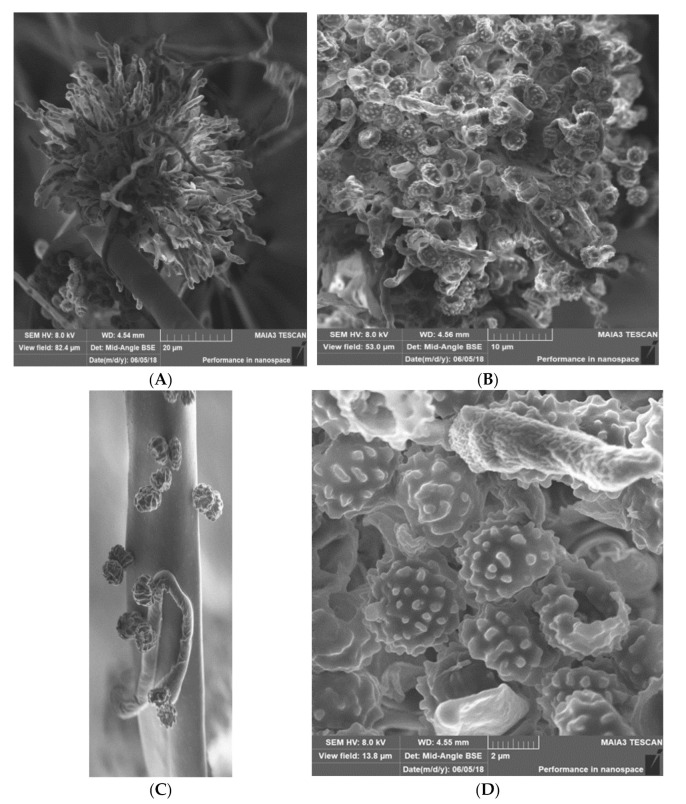


**Figure 1.** Morphology of *Aspergilus niger*. (**A**)—general view, (**B**)—the stage of development of the conidiophoid, (**C**)—the adhesion of spores to the porous structure of the vegetative mycelium hyphae, (**D**)—disputes.

The central element of the structures of asexual reproduction in *Aspergillus flavus* is the swelling—apical expansion of the conidiophoid hypha (conidiophoid) (Figure 2A)

As development progresses at the ends of the phialids, unicellular conidia are sequentially formed (Figure 2B,C), which are located in unbranched chains until maturation (Figure 2D).


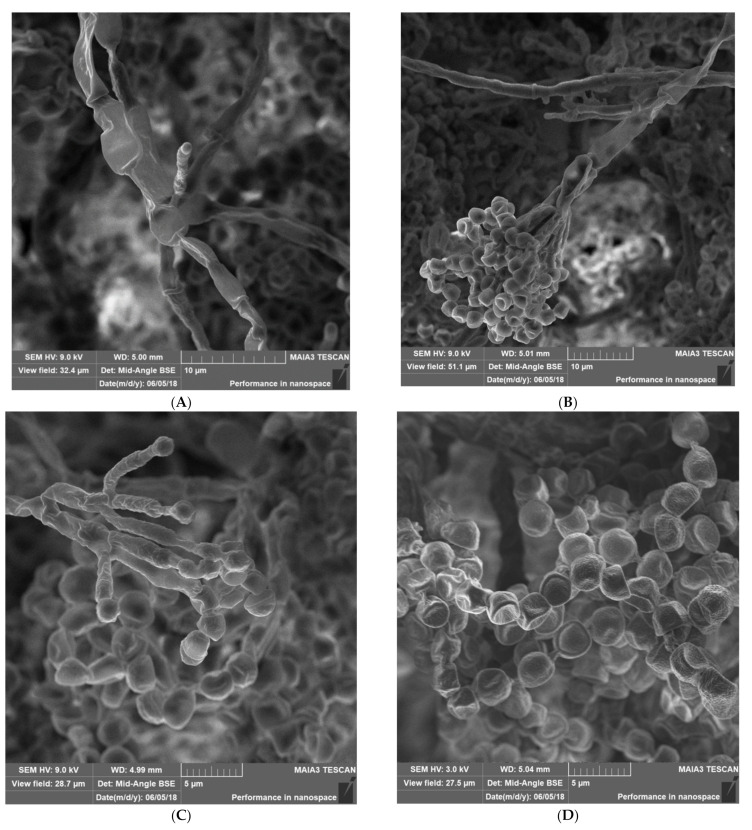


**Figure 2.** Morphology of *Flavus*. (**A**–**C**)—stages of formation of spore-bearing structure, (**D**)—chain of spores.


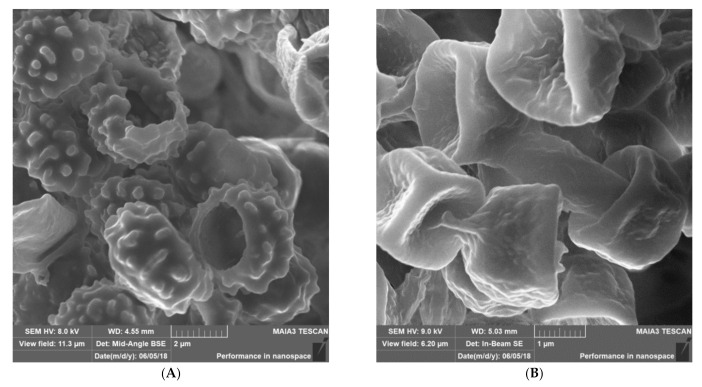


**Figure 3.** Differences in spore morphology. (**A**)—*Aspergilus niger* (**B**)—*Aspergilus flavus*.

The use of an electron microscope as an additional diagnostic test made it possible to reveal qualitative differences in the morphology of the isolated strains of fungi of the *Genus aspergillus*.

## P380 A Case of *Aspergillus niger* Endocarditis after Transcatheter Aortic Valve Implantation (TAVI) in a Patient with Well-Controlled HIV-Infection

Jacobus Herderschee ^1^, Michèle van Vugt ^2^, Matthijs Boekholdt ^3^, Jochem Buil ^4^, Caspar Hodiamont ^1^ and Karin van Dijk ^1^

^1^ Department of Medical Microbiology and Infection Control, Amsterdam University Medical Centers
^2^ Department of Internal Medicine & Infectious Diseases, Amsterdam UMC
^3^ Department of Cardiology, Amsterdam UMC
^4^ CWZ Center of Expertise for Mycology, Nijmegen Medical Center, Department of Medical Microbiology, Radboudumc, Radboud University

**Keywords:** *Aspergillus tubingensis*; endocarditis; bioprosthesis; TAVI

**Background:** Aspergillus is a rare cause of endocarditis, and carries a poor prognosis. Cinically, the disease is characterized by an insidious onset, with peripheral embolisms often the presenting feature. The rarity of the condition combined with the sparsity of literature make diagnosis and management challenging and often delayed.

**Case presentation:** An 84-year old man with well-controlled HIV (viral load below the detection limit, CD4 count 440 cells/mm^3^) and a bioprosthetic aortic valve since 8 years was admitted for heart failure due to premature failure of the prosthesis. He underwent valve-in-valve Transcatheter Aortic Valve Implantation (TAVI). Several weeks post-procedure, he was re-admitted with acute heart failure; echocardiography showed severe TAVI stenosis which was presumed to be caused by valve thrombosis. After two failed thrombolysis attempts, he underwent open heart surgery as a last resort. Both valves were completely removed, and a new bioprosthesis implanted. The valve leaflets were largely disintegrated and sent to the clinical microbiology department for culture. No material was sent to the pathology department. After 1 day of incubation all cultures showed fungal growth and the patient was started on combination antifungal therapy with liposomal amphotericine B (3 mg/kg/d) and voriconazol (started at 270 mg/d; target ≥2 mg/L). Serum galactomannan index was 0.9. Blood cultures, including cultures using BacTec Mycosis IC/F bottles (BD Diagnostics, Sparks, MD, USA), remained negative.

The fungus was identified as *Aspergillus niger* and liposomal amphotericine B was switched to anidulafungin pending susceptibility testing as the patient had pre-existing renal function (eGFR CKD-EPI 40 mL/min/1.73 m²). Sequence analysis identified the fungus as *Aspergillus tubingensis* (*Aspergillus niger* complex); susceptibility performed according to EUCAST: voriconazol MIC 1 mg/L, anidulafungin 0.03 mg/L, caspofungin: 0.06 mg/mL. Despite increasing the anidulafungin dosage to 200 mg daily and adequate voriconazol levels, serum galactomannan levels remained elevated (index 2.1 at discharge). A PET-CT did not provide evidence for extracardiac sources of ongoing infection. Since no extracardiac sources were found and the patient did well clinically, he was discharched. Anidulafungin was switched to caspofungin to facilitate ongoing IV treatment in the outpatient setting. During the following 6 months the patient continued to receive caspofungin and voriconazol while serum galactomannan levels remained elevated but stable. After 6 months serum galactomannan trippled while his NT-proBNP increased 7-fold accompanied by a mild leukocytosis and slightly increased CRP. The patient died shortly after. Obduction was not performed.

**Discussion and Conclusions:** We described a case of *Aspergillus tubingensis* endocarditis after TAVI in a man with a well-controlled HIV infection which could not be controlled despite surgical removal of the affected valve and combination antifungal treatment.

## P381 A Case of Aspergillus Prosthetic Valve Endocarditis

**Salima Rattani, Joveria Farooqi, Faisal Mahmood, Sadaf Zaka and Kauser Jabeen**

Agakhan University Hospital

**Objectives:** Prosthetic valve endocarditis due to fungi is an extremely severe form of infective endocarditis, with poor prognosis and high mortality despite treatment.

**Case Report:** Here we present a case of fungal prosthetic valve endocarditis in a 25-year-old man following aortic and mitral valve replacement in September 2020. He had a complicated course after his valve replacement with a frontal intracranial hemorrhage in January 2021 due to anticoagulation. A month later he complained of lower limb pain associated with numbness, tingling, and skin discoloration. A peripheral CT angiography with contrast was done which showed multifocal cortical infarctions in the right kidney and multifocal occlusions in major arteries of bilateral lower legs including the tibioperoneal trunk, posterior and fibular artery, followed by distal reconstitution. A diagnosis of right foot critical ischemia was made for which a right popliteal embolectomy was done. A follow-up CT scan head with contrast showed a well-defined rounded ring-enhancing lesion in the right frontal lobe likely representing abscess formation. Transthoracic echocardiography (TTE) to rule out endocarditis was performed which was normal. Two weeks later the patient returned with bilateral lower limb pain associated with cold feet, numbness, tingling sensation, and skin mottling. A repeat peripheral CT angiography with contrast showed progression of the arterial occlusion now involving the common and external iliac and superficial femoral artery bilaterally. A bilateral femoral embolectomy was done and the thrombus was sent for culture and sensitivity testing. A 3% potassium hydroxide preparation from the sample showed septate hyphae and culture revealed growth of *Aspregillus fumigatus*. Triazole resistance screen for the isolate was negative with a quadrant each of 1 µg/mL Voriconazole, 4 µg/mL itraconazole and 0.5 µg/mL posaconazole in RPMI agar. Transesophageal echocardiography (TEE) showed a large aortic aneurysm with multiple, large, mobile vegetations of the prosthetic aortic valve. A diagnosis of fungal endocarditis due to *Aspergillus fumigatus* was made and antifungal therapy with 400 mg intravenous voriconazole as loading dose followed by 200 mg twice a day as maintenance dose was initiated and a valve replacement was planned. However, before the surgery could be performed, the patient died of a large intracranial parenchymal hemorrhage involving the frontal lobe.

**Conclusions:** The reported case illustrates an uncommon presentation of fungal endocarditis and demonstrates that a delayed diagnosis and treatment as in this case where patient presented with metastatic vessel occlusion (cerebral, renal, peripheral arterial) leads to higher mortality. A strong suspicion, early microbiological diagnosis, radiological diagnosis with TEE and adequate appropriate therapy, both medical and surgical are crucial to achieve good outcomes in patients with fungal endocarditis.

## P382 Virulence and Antimycotic Resistant Fungi of the Genus *Candida*

**Marina Manoyan, Vladimir Cokolov, Ahasnasya Gurcheva and Narine Gabuzyan**

The Russian State Center for Animal Feed and Drug Standardization and Quality, Fse Vgnki, Federal State Institution

**Objectives:** The aim of this work was to study some virulence factors of *Candida catenulata* and *Candida lipolytica* yeast fungi isolated from different sources associated with farm animals: growth rate at +37 °C, activity of proteolytic enzymes and phospholipases, intensity of mycelial structure formation and antifungal drugs sensitivity—fluconazole and voriconazole.

**Materials and methods:** The yeast fungi studied were isolated from raw milk samples and from cattle udder skin using standard methods. Identification was performed using the CandidaTest21 test system (Erba LaChema). A total of 16 *Candida catenulata* isolates, 12 *Candida lipolytica* isolates and 10 *Candida albicans* strains were used as controls.

The growth rate at a temperature of +37 °C.

Each isolate was cultured in Sabouraud liquid medium for 24 h.

**Activity of proteolytic enzymes and phospholipases.**

Each isolate was cultivated in YCB nutrient medium (Yeast carbon base, HiMedia laboratories) with the addition of bovine serum albumin at 5% (weight/volume) to determine proteolytic activity; egg yolk emulsion at 5% (volume/volume) was added to the nutrient medium to determine phospholipase activity. Five sterile discs of filter paper were placed on the surface of the nutrient medium, on which 10 mcL of culture suspension with a density of 0.5 units McF was then applied, incubated for 24 h at +37 °C. After cultivation, the diameter of the lysis zone around the disk was measured, and then the ratio of the colony (disk)diameter to the diameter of the lysis zone was determined.

**Formation intensity of mycelial structures.**

Each isolate was cultivated in liquid nutrient medium RPMI-1640 with the addition of 20% (volume/volume) of cattle fetal serum (experimental group) and without it (control group) for 60 min at +37 °C with constant stirring. After cultivation, the total number of cells, the number of mycelial structures, and their ratio in each group were counted. Then it was calculated how many times the relative number of mycelial structures in the experimental group exceeds this value in the control group.

**Sensitivity to antifungal drugs.**

The sensitivity of isolated isolates to antifungal drugs was determined by agar diffusion according to the procedure described in CLSI M44. The results obtained were interpreted according to CLSI M44s-3.

In the experiment, we used HiMedia discs containing 25 μg of fluconazole and 1 μg of voriconazole and Muller-Hinton agar modified for fungi (HiMedia).

**Results:** The optical density of cultures of *Candida lipolytica* (4.31 ± 0.16) isolates grown at 37 °C is significantly lower than the optical density of cultures of *Candida albicans* (4.66 ± 0.26) No significant differences in OD between *Candida catenulate* (4.63 ± 0.18) and *Candida albicans* isolates were found.

All the differences obtained by comparing the studied isolates with the control strains in terms of enzyme activity are statistically reliable. Based on these data, it was concluded that proteolytic enzymes and phospholipases of *Candida catenulata* (0.92 ± 0.04 (*proteinases*), 0.94 ± 0.04 (*phospholipases*) and *Candida lipolytica* (0.96 ± 0.02 (proteinases), 0.95 ± 0.04 (*phospholipases*) isolates have less activity in comparison with the control *Candida albicans* (0.8 ± 0.04 (*proteinases*), 0.84 ± 0.04 (*phospholipases*) strains under the experimental conditions.

The differences obtained by comparing the studied isolates with the control strains in terms of the intensity of mycelial structures’ formation are statistically reliable. Based on these data, it was concluded that *Candida catenulata* (3.37 ± 0.28) and *Candida lipolytica* (2.66 ± 0.2) isolates less intensively form mycelial structures—true and pseudomycelium in comparison with the control *Candida albicans* (4.08 ± 0.18) strains under the experimental conditions Figure 1.

Sensitivity to antifungal drugs, 3 *Candida catenulata* isolates and 8 *Candida lipolytica* isolates were determined to be resistant to fluconazole (19 and 67%, respectively), with a growth suppression zone diameter ≥14 mm. 3 *Candida catenulata* isolates and 7 *Candida lipolytica* isolates were found to be resistant to voriconazole (19 and 58%, respectively), with a growth suppression zone diameter ≥13 mm.

**Conclusions:** The virulence potential of the studied *Candida catenulata* and *Candida lipolytica* isolates was characterized as lower in comparison with *Candida albicans*. Indicators of growth rate at +37 °C, activity of proteolytic enzymes and phospholipases, intensity of formation of mycelial structures in the studied isolates were significantly lower than in the control virulent strains of *Candida albicans*.

More than half of the studied *Candida lipolytica* isolates were resistant to fluconazole or voriconazole, and cross-resistance to both drugs was observed in 7 of 8 isolates; among *Candida catenulata* isolates the proportion resistant to fluconazole or voriconazole was 19%.

Our results raise some concerns because the isolates studied were isolated from animals in which azole antimycotics are not used in therapy, or from environmental objects, and these isolates already have a high level of resistance, including cross-resistance. The isolates studied are also virulent.

## P383 Guinea Pigs—An Experimental Model for Dermatophytosis

**Vladimir Sokolov, Marina Manoyan, Anastasya Gursheva and Narine Gabuzyan**

The Russian State Center for Animal Feed and Drug Standardization and Quality, Fse Vgnki, Federal State Institution

**Objectives:** The presented model of dermatophytosis of guinea pigs is used to assess the therapeutic efficacy of new antifungal drugs being developed in the Russian Federation. In view of the fact that pathogens of dermatophytosis acquire resistance to antifungal drugs, the development of new antimycotics is an urgent task both in medicine and veterinary medicine.

The model makes it possible to evaluate the therapeutic efficacy of antifungal drugs, both for topical and systemic use. The model uses at least two main indicators: the severity of the infectious process (clinical signs) and the presence of a viable pathogen in the focus of infection.

**Materials & Methods:** To obtain experimental dermatophytosis in guinea pigs, we used standardized virulent strains of the fungi *Microsporum canis and Trichophyton mentagrophytes* deposited in the collection of FSE VGNKI.

Preparation of virulent cultures for infection: A suspension of microconidia for infecting experimental animals was prepared from 14 day old virulent cultures of the second generation. A preliminary assessment was made of the microbiological purity of the culture and its morphological characteristics. To prepare a suspension of *Microsporum canis/Trichophyton mentagrophytes* microconidia, the culture was washed from the nutrient medium with 0.9% sodium chloride solution with the addition of 0.1% polysorbate 80. Then the suspension was filtered through a track membrane with a pore diameter of 3.0 μm to remove mycelium fragments. After filtration, the concentration of spores was calculated using a Goryaev chamber, then the concentration was brought to 3.0 × 10^7^ spores/mL using a physiological solution of sodium chloride with the addition of 0.1% polysorbate 80. The resulting suspension was used on the day of preparation.

Infection of animals: The animals were shaved off a 3 × 3 cm skin area in the lower back, the shaved area was treated with 70% ethanol solution, and scarified with fine-grained emery paper until hyperemia appeared. A dermatophyte culture suspension containing 1.0 × 10^8^ million spores/mL in a volume of 0.1 mL was applied to the scarified area, thus, the infecting dose was approximately 3.3 million spores per 1 cm^2^ of the skin area.

During the reproduction of the model, the severity of clinical signs was recorded daily. The severity of clinical signs was assessed in points on a scale from 0 to 4, where 0—complete absence of clinical signs, 4—bright severity. The following manifestations were assessed: hyperemia of the skin, the presence of peeling, crusts or scabs, their area, the presence of damaged and/or deformed hair at the borders of the lesion. linical material (hair and/or skin scales) was collected every day to isolate the pathogen. The observation period for the animals was 30 days.

Research of clinical material: The clinical material was plated on Sabouraud’s selective medium with chloramphenicol and cyclohexemide (M664, HiMedia). The growth of the pathogen culture was recorded by examining the crops daily for 14 days.

Data processing: Primary data, scores for assessing the severity of the infectious process, were collected for each animal on a daily basis. Then the lesion index was calculated for each group, in fact, the average score in the group. On the basis of this indicator, the groups of animals involved in the experiment were compared. Comparison was performed using the Mann-Whitney test.

**Result:** In general, the described model is suitable for assessing the effectiveness of antifungal drugs, both local and systemic. The use of virulent breeding strains of dermatophytes makes it possible to guarantee a pronounced infectious process in experimental animals, the duration of which is up to 30 days.

**Conclusions:** The use of guinea pigs (*Cavia porcellus*) as experimental animals seems to be an acceptable solution, since they are susceptible to pathogens of dermatophytosis, are not aggressive when kept in a group, are calm towards humans and are practically not subject to stress, which is inevitable during manipulations.

## P384 Phenotypic Identification of *Aspergillus* Isolates and Their Susceptibility Pattern to Conventional Antifungal Agents by Broth Microdilution Method

**U. Almas Fathima ^1^, Kindo Anupma J. ^1^, S. Prasanna Kumar ^1^, M. Thirunarayan ^2^ and V. Lakshmi Sree ^3^**

^1^ Sri Ramachandra Institute of Higher Education and Research
^2^ Apollo Hospitals
^3^ Apollo Speciality Hospital

**Objective:** To Identify the species of *Aspergillus* isolates and to determine the Antifungal susceptibility patterns of *Aspergillus* isolates by Broth Microdilution method.

**Methods:**

- *Aspergillus* grown from various clinical sample (Ear swab, Bronchial wash, Endotracheal Aspiration, Paranasal sinus, BAL, Sputum) was subcultured on Sabouraud’s Dextrose Agar/Oat meal Agar.
- Tease mount/Slide culture was done to study the morphological features of the hyphae, size, shape and arrangement of the conidia.
- Antifungal susceptibility testing (AFST) was done according to CLSI guidelines M38-A2 2008 documented reference method for Broth Microdilution Antifungal susceptibility of Filamentous fungi.
- Antifungal stock solutions of AmphotericinB, Voriconazole, Posaconazole, Itraconazole and Caspofungin was prepared in di-methyl sulfoxide and stored at −70 °C.
- RPMI medium was prepared, adjusted to pH 7 and filter sterilized. Sterility was checked and stored at 4 °C.
- Dilution of the stock solution was done as per the guidelines.
- Inoculum was prepared by adjusting conidial suspension in sterile distilled water to 0.5 McFarland Turbidity standard; to 100 µL of drug solution, 100 µL of inoculum in RPMI 1640 medium was added.
- Test micro-titre plate was incubated for 48 h at 35 °C.
- Growth and sterility control wells were included in each test.

AFST-READING RESULT:

- For AmphotericinB, Voriconazole, Posaconazole, Itraconazole—MIC is the first well without visible growth.
- For Echinocandins, e.g., Caspofungin- MEC is the point (lowest value) where a visible change in growth as compared to the positive growth is noted.

**Result:** A total of 30 *Aspergillus* isolates were collected—*Aspergillus niger* (14), *Aspergillus flavus* (9), *Aspergillus fumigatus* (6) & *Aspergillus terreus* (1). The results were as follows.

**Conclusions:** The susceptibility profile showed high mean MIC for Amphotericin B for most of the species of *Aspergillus*; high MIC (mean) for Itraconazole in case of *Aspergillus niger* and high MIC (mean) for Itraconazole, Voriconazole and Posaconazole in case of *Aspergillus flavus*. In case of *Aspergillus terreus*, being resistant in nature the low MIC is recorded only with Caspofungin. *Aspergillus fumigatus* was found to be highly susceptible to all the 5 antifungal agents tested. Due to less susceptibility of *Aspergillus* to the common antifungal agents, it is important to perform antifungal susceptibility testing to reduce the morbidity and mortality in patients especially with invasive aspergillosis.

## P386 Quality Control in Mycology from Qualification to Quantification “Indian Ink” Test

**John Guzmán ^1^, José Rodas ^1^, Clara Duque ^1^, Claudia Cuervo ^1^, Iván Mojica ^2^ and Juan Gómez ^2^**

^1^ University Institution Colegio Mayor of Antioquia
^2^ Synlab Laboratory S.A.S

**Objective:** To implement a quantitative methodology which allows a more precise and detailed evaluation in time of the “India ink” test.

**Materials & methods:** Two levels of control were performed; (a) Suspension of capsulated cells of *Cryptococcus neoformans* var. *grubii,* in formalin and adjusted to a volume of 2 mL in McFarland concentration 1.0. (b) *Cryptococcus neoformans* var. *grubii* capsulated and *Rhodotorula mucilaginous* with formalin, adjusted to a volume of 2 mL at 2.0 McFarland concentration. Both strains were identified by MALDI-TOF MS. Subsequently, a validation of the statistical procedure was carried out with the Minitab tool 17 version. The x-R charts were used with two operators, one expert and one in training, including both levels of control. The recounts were conducted in Neubauer’s chamber in duplicate over a period of thirty days. The visualization of the capsule was achieved with a 1:16 dilution of India ink. After graphing the data using the Minitab version 17 tool. The expert’s counts were used to generate a graph of Levey jennings, and those belonging to the operator in formation were graphed to determine the consistency or variability of the process.

**Results:** The analysis of the x-R graphs in both technicians separately showed consistency without excessive variability. Some important fluctuations were observed which did not prevent the approval of the validation by statistical method. Added to the values of *p* > α (i.e., > 0.05), indicating that there are no statistically significant differences, and thus demonstrating that the analysis of the intra-observer results were appropriate. However, the inter-observer analysis yielded *p* < α values that expressed statistically significant differences. The Levey jennings graphs showed excellent performance for level (b), unlike level (a), where -3DS, 10× and 3:1S alarms were observed.

**Conclusions:** Quality controls of a quantitative nature are important, allowing exhaustive monitoring in the time. These controls carried out in mycology allow monitoring processes, such as colorations. Finally, the cell count method in Neubauer chamber with the statistical graphs showed that the assay meets adequate performance characteristics for analytical measurements required.

## P391 Production of Enzymes and Hemolysins by *Nannizzia gypsea* Isolated from the Soil of Parks in São Paulo, Brazil

**Marilia Alarcon Nogueira ^1^, Ulisses Carrer ^1^, Guilherme da Silva Tamaki ^1^, Juan Justino Neves^2^ and Selene Dall’ Acqua Coutinho ^1^**

^1^ Paulista University
^2^ Cruzeiro do Sul University

**Objectives:** Dermatophytosis is of significant importance for public health because of its zoonotic characteristic. In particular, geophilic dermatophytes have been insufficiently studied because they are considered strictly opportunistic. However, cases of infections due to *Nannizzia gypsea*, a geophilic dermatophyte, in both humans and animals have been found globally, confirming the pathogenic potential of geophilic fungi. Dermatophytoses caused by these fungi is attributed to several proteins and enzymes produced by them and also the use of the host’s stratum corneum by the fungi. Therefore, the study of virulence factors of these fungi will be helpful in understanding the epidemiology of dermatophytoses. The aim of this study was to investigate the secretion of hemolysins, proteinase, phospholipase, lipase, and gelatinase enzymes by *N. gypsea* strains.

**Methods & Materials:** Twenty-one strains of *N. gypsea* isolated using the Vanbreuseghem technique from the soil samples of 21 parks in São Paulo, Brazil, were tested. The production of phospholipase, proteinase, and lipase was verified by culturing these strains in Petri dishes containing a solid culture medium. This medium contained the respective substrate for degradation such as egg yolk, bovine serum albumin, and Tween 20. The test was considered positive when there was a clear area of degradation around the colony. To check the production of gelatinase, a medium containing gelatin was used that was distributed in tubes and punctiform inoculation was performed. Before the final reading, the tubes were refrigerated at 4 °C for 30 min. The test was considered positive when there was a liquefaction of the medium. In the hemolysin production test, blood agar base supplemented with 5% defibrinated sheep blood was used. In this test, after reading for 14 days, the plates were incubated at 32 °C. In each of these tests, after inoculation, the plates were incubated for 14 days at 25 °C, with readings taken at 5, 7, 10, and 14 days.

**Results:** All the 21 strains of *N. gypsea* produced phospholipase at 14 days, gelatinase from 10 days, and proteinase and lipase from 7 days of incubation. Most of the reactions were strongly positive for the production of phospholipase. At 14 days at 32 °C, hemolysis was observed in 81.0% (17/21) of the strains, with α-hemolysis (partial hemolysis) in 82.4% (14/17) of the strains and β-hemolysis (complete hemolysis) in 17.6% (3/17) of the strains (Figures 1 and 2). One of the strains produced both α- and β-hemolysis.

**Conclusions:**

- All the strains of *N. gypsea* produced enzymes and/or hemolysins, which are considered virulence factors, thus revealing their pathogenic potential.
- The ideal reading intervals for detecting the production of phospholipase, proteinase, lipase, and gelatinase were 14, 7, 7, and 10 days respectively. For detecting hemolytic activity, 14 days at a temperature of 32 °C is recommended.
- The presence of virulent strains of *N. gypsea* in the soils of parks in São Paulo may represent a potential risk of infection to humans and animals that enjoy these spaces for leisure.

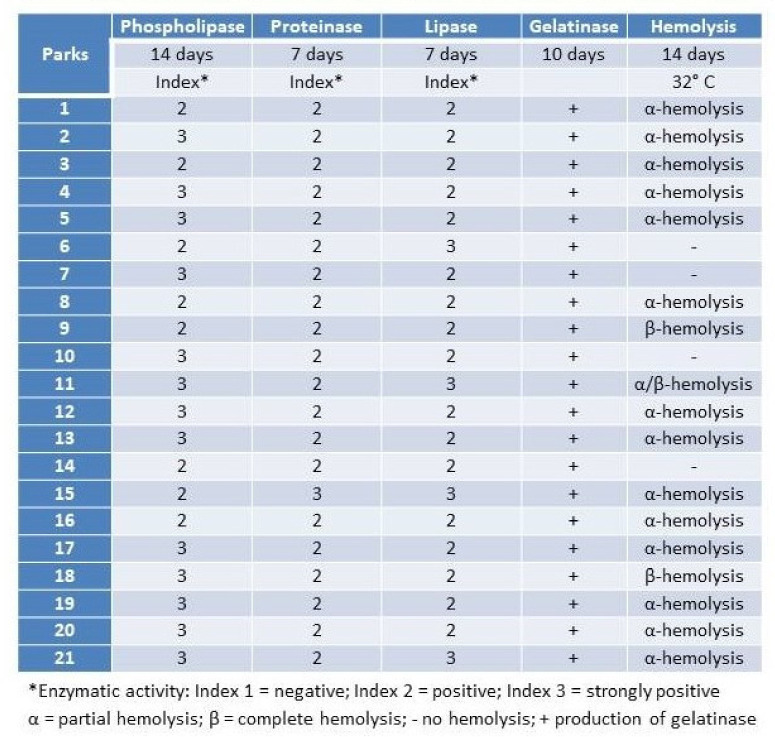


**Figure 1.** Production of phospholipase, proteinase, lipase, gelatinase, and hemolysins by *Nannizzia gypsea* strains isolated from the soil of parks in Sao Paulo.


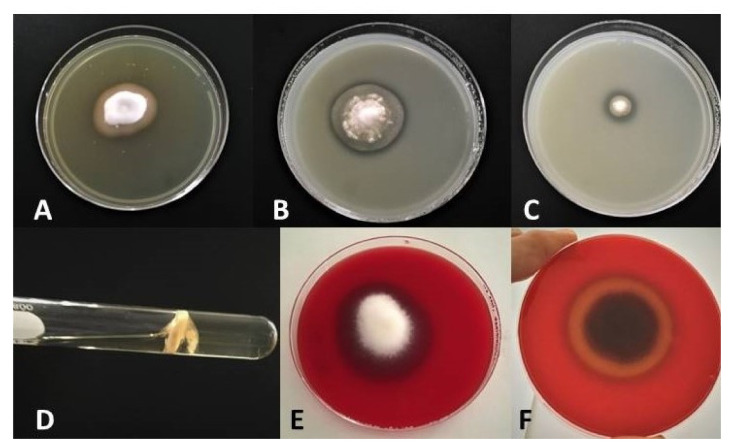


**Figure 2.** Production of enzymes and hemolysins by Nannizzia gypsea. (**A**) phospholipase; (**B**) Lipase; (**C**) proteinase; (**D**) gelatinase; (**E**) α-hemolysis; (**F**) β-hemolysis.

## P395 Effects of Cytochrome p450 (CYP) 2C19 Genetic Polymorphisms on Voriconazole Serum Levels: A Report of 2 Cases

**Zahit Tas ^1^, Emre Kara ^2^, Ozan Deger ^3^, Mukaddes Ispirli ^3^, Gokhan Metan ^1^, Melih O. Babaoglu^3^ and Murat Akova ^1^**

^1^ Department of Infectious Diseases and Clinical Microbiology, Faculty of Medicine, Hacettepe University
^2^ Department of Clinical Pharmacy, Faculty of Pharmacy, Hacettepe University
^3^ Department of Medical Pharmacology, Faculty of Medicine, Hacettepe University

**Introduction:** Voriconazole (VCZ) is a triazole antifungal drug that is the first choice in the treatment of invasive aspergillosis (IA). VCZ is primarily metabolized by the enzyme cytochrome p450 (CYP) 2C19. VCZ has a narrow therapeutic range. The successful treatment outcomes were correlated with plasma trough concentrations between 1.5–5.5 mg/L. Genetic polymorphisms of CYP2C19 have been reported to be associated with variability in VCZ pharmacokinetics that may lead to a decrease in the efficacy of this antifungal drug. In this report, we describe two patients, treated by VCZ for presumed invasive pulmonary aspergillosis, with CYP2C19*1/*17 genotypes whose target serum levels could not be reached despite the appropriate dose of VCZ.

**Case Reports:** Trough serum levels of VCZ were measured by liquid chromatography-tandem mass spectrometry (LC-MS/MS) assays with the EUREKA^®^ kit on the 5th day of VCZ treatment in two patients. VCZ dose was driven by the actual body weight and the patients did not receive any drugs that can interfere with VCZ levels. The first patient received intravenous VCZ at a dosage of 360 mg twice as loading followed by 240 mg twice a day as a maintenance dose. VCZ levels were detected at about 0.2 mg/L, which is too low to be expected. We suspected that he might be an ultrarapid or rapid metabolizer of VCZ and genotyping for CYP2C19 was performed. This patient had CYP2C19*1/*17 genotype and had a predicted phenotype of rapid metabolizer for CYP2C9 activity. Then, the VCZ dose was increased to 6 mg/kg which requires administration of 360 mg i.v. every 12 h. Serum levels that were measured at the 10th and 11th days of the treatment were within the therapeutic concentration range as 4.7 mg/L and 4 mg/L, respectively. In the second patient, VCZ levels were measured as 0 mg/L. Repeated serum sampling yielded a level of 0.1 mg/L. Antifungal treatment was changed to liposomal amphotericin B (5 mg/kg/day) which was stopped after recovery from neutropenia. This second patient had CYPC219*1/*17 genotypes and her predicted phenotype was also a rapid metabolizer for CYP2C9 activity. Primer sequences and enzymes were used to determine cytochrome P450 2C19 (CYP2C19) polymorphisms for each patient were given in Table 1.

**Conclusions:** CYP2C19 polymorphisms should be carefully evaluated in patients with very low plasma levels of VCZ. CYP2C19 genotyping could potentially improve the safety and efficacy of VCZ treatment. To the best of our knowledge, these patients are the first cases reported to have very low plasma levels VCZ associated with CYP2C19 genetic variant alleles in Turkey.

**Table 1.** Primer sequences and enzymes were used to determine cytochrome P450 2C19 (CYP2C19) polymorphisms and restriction fragments in the PCR-RFLP method.



**Genetic Polymorphism**

**Primer Sequence**

**Endonuclease**

**Cleavage Pattern (bp)**

CYP2C19*17

*−3402C > T*

*rs12248560*
F ^a^: 5′-AATAAAGATGACCTTGATCTGG-3′
*MnlI*
280 + 224 (WT) ^c^R ^b^: 5′-GTCTCCTGAAGTGTCTGTAC-3′504
CYP2C19*2 rs4244285
F ^a^: 5′-CAGAGCTTGGCATATTGTATC-3′
*SmaI*
212 + 109 (WT) ^c^R ^b^: 5′-GTAAACACACAACTAGTCAATG-3′321
^a^ Forward primer sequence, ^b^ Reverse primer sequence, ^c^ Wild type.


## P396 Pharmacogenomics and Therapeutic Drug Monitoring of Voriconazole

**Jodi Taraba, Sila Shalhoub, Juliana Merten, Xiaojia Tang, Krishna Kalari, Christina G. Rivera and Paschalis Vergidis**

Mayo Clinic

**Objectives:** Voriconazole undergoes hepatic metabolism by the cytochrome P450 enzyme system. Metabolism is primarily mediated by *CYP2C19*, and to a lesser extent, *CYP2C9* and *CYP3A4*. Voriconazole demonstrates wide interpatient variability in serum concentrations due in part to *CYP2C19* variant alleles. We sought to evaluate the role of pharmacogenomics in predicting voriconazole concentrations in a real-world clinical setting.

**Methods:** We conducted a retrospective review of patients (aged ≥16 years) who received treatment with voriconazole and had *CYP2C19*/*CYP2C9* genotyping and at least one voriconazole trough concentration at steady state between June 2009 and April 2021. We excluded critically ill patients, allogeneic stem cell transplant recipients, and patients receiving treatment with a rifamycin due to the drug-drug interaction with voriconazole.

*CYP2C19* genotypes were classified into the following phenotypes: poor (*2/*2), intermediate (*1/*2, *1/*3), normal (*1/*1), intermediate to normal (*2/*17), rapid (*1/*17) and ultrarapid metabolizers (*17/*17). *CYP2C9* genotypes were classified into poor to intermediate (*2/*3), intermediate (*1/*3, *1/*18, *2/*2), normal (*1/*1) and intermediate to normal (*1/*2) metabolizers.

Drug concentrations <1.0 mcg/mL were considered subtherapeutic. Values were adjusted using the following formula: drug concentration/(daily dose/weight). Two sample *t*-test and one-way ANOVA were used for comparisons between the groups, as appropriate.

**Results:** Sixty-nine patients were included in the analysis. Mean age was 54 years (range, 17–80), 52.2% were female (95.7% white, 2.9% Asian, 1.4% Black). Median weight was 71.0 kg (interquartile range, 60.0–82.0). Most patients received voriconazole via the oral route (92.8%). In 63.8%, treatment was given in the inpatient and 36.2% in the outpatient setting. A proton pump inhibitor was co-administered in 20/47 (42.5%) patients.

For *CYP2C19* (n = 69): (i) 11.6% were poor/poor to intermediate, (ii) 55.1% were normal/intermediate to normal, (iii) 33.3% were rapid/ultrarapid metabolizers. There was no significant difference in mean drug concentration between the 3 groups (1.78, 1.71, 1.46 mcg/mL; *p* = 0.79). No difference was found after adjusting for dose and weight (0.32, 0.29, 0.27; *p* = 0.90). Subtherapeutic concentration was noted in 42.1% (16/38) of normal metabolizers and 43.5% (10/23) of rapid metabolizers (*p* = 0.86).

For *CYP2C9* (n = 59): (i) 18.6% were intermediate/poor to intermediate, (ii) 81.4% were normal/intermediate to normal metabolizers. We did not find a difference in mean concentration (1.78 vs. 1.65 mcg/mL; *p* = 0.79) even after adjustment (0.32 vs. 0.29; *p* = 0.75).

**Conclusions:** In this cohort, *CYP2C19* and *CYP2C9* metabolizer phenotypes were not predictive of supra- or subtherapeutic concentrations of voriconazole. Epigenetic alterations or extrinsic factors such as administration with food, drug interactions, and comorbidities may influence voriconazole metabolism. Functional characterization of variants of unknown significance may improve the accuracy of pharmacogenomics.

## P397 Does Therapeutic Drug Monitoring Affect the Treatment Switch and Route of Administration of Voriconazole?

**Emre Kara ^1^, Pinar Bakir-Ekinci ^2^, Gokhan Metan ^2^, Aygin Bayraktar-Ekincioglu ^1^, Asli Pinar ^3^, Murat Akova ^2^ and Omrum Uzun ^2^**

^1^ Hacettepe University Faculty of Pharmacy, Department of Clinical Pharmacy
^2^ Hacettepe University Faculty of Medicine, Department of Infectious Diseases and Clinical Microbiology
^3^ Hacettepe University Faculty of Medicine, Department of Biochemistry

**Objectives:** Pharmacokinetics of voriconazole (VRC) is not linear and interpatient variability creates a challenge for the treatment, therefore, therapeutic drug monitoring (TDM) is recommended as part of the routine care. In a case of where TDM for VRC is not available, potential toxicity or ineffectiveness can easily trigger the treatment switch. Moreover, physicians can avoid use oral VRC due to the risk of subtherapeutic drug levels. This study aimed to investigate the impact of TDM in voriconazole treatment on the treatment switch and preferences of route of administration.

**Materials & Methods:** Patients are aged over 18 years and given antifungal therapy between January 2019–November 2020 in the Hacettepe University Hospitals were included. The study was approved by the University Clinical Research Ethics Committee. Demographics and antifungal treatment periods of the patients were analyzed retrospectively. As of January 2020, VRC plasma levels had started to be analyzed on a weekly basis in the study setting. Plasma VRC levels (target range: 1–5.5 mg/L) were measured by using liquid chromatography—triple quadrupole mass spectrometer (Shimadzu LCMS-8040). The impact of TDM on appropriateness of VRC treatment were evaluated by the numbers of treatment switch and the use of oral route.

**Results:** During the study period, 70 patients (30% female), with a median age of 55.5 (20–87) years, were treated with VRC. The administration routes of VRC were parenteral in 61 (87.1%) and oral in 9 (12.9%) patients (Table 1). Thirty-one (44.3%) patients received VRC while TDM was not available, and 39 (55.7%) received when TDM became available at the setting. A total of 88 plasma levels of VRC were monitored in 34 (87.2%) patients where a median number of measurements per patient was 2 (1–8). The median plasma level of VRC was 4.2 (0.2–18.1) mg/L. Categorization of plasma levels indicated that 45 (51.1%) were at therapeutic, 14 (15.9%) at subtherapeutic, and 29 (33.0%) at supratherapeutic range. By the availability of TDM, 30 interventions were carried out for VRC therapy; the most commons being reducing the dose (n = 11, 42.3%) and switching to oral therapy (n = 8, 26.7%). Four VRC-related adverse effects were detected, of those observed at supratherapeutic levels (Table 2).

VRC was switched to another antifungal in 7 and 4 patients in no TDM and with TDM periods, respectively. A significant increase was observed in the use oral VRC by the TDM period (Table 1).

**Conclusions:** This study was shown that therapeutic drug monitoring for VRC can increase the usage of oral VRC which is emphasized by the clinical guidelines. Therefore, measurements of plasma levels can be integrated into the routine practice and antifungal treatment can be optimized by implementation of TDM.

**Table 1.** Comparison of the periods before and after the TDM (n = 70), n (%).




**Before TDM **

**After TDM **

*p*-Value *
Antifungal therapy change


Switch to VRC Switch from VRC 5 (41.7) 7 (58.3)8 (66.7) 4 (33.7)0.219Administration route


Parenteral Oral30 (96.8) 1 (3.2)31 (79.5) 8 (20.5)0.038
* Chi-square test.


**Table 2.** VRC treatment and the plasma levels in the TDM period (n = 88), n (%).




**Therapeutic**

**(1–5.5 mg/L)**

**Subtherapeutic (<1 mg/L)**

**Supratherapeutic (>5.5 mg/L)**

***p*-Value**
Administration route, n (%)



Parenteral Oral29 (35.6) 16 (64.4)7 (50.0) 7 (50.0)22 (75.9) 7 (24.1)0.235Adverse effects, n (%)0 (0.0)0 (0.0)4 (13.8)-median dose/day (mg), (minimum–maximum)400 (360–800)480 (300–900)560 (400–800)0.113


## P398 Lower Isavuconazole Blood Levels in ICU Patients Compared to Non-ICU Patients

**Malgorzata Mikulska ^1,2^, Elisa Furfaro ^1^, Daniele Giacobbe ^1,2^, Laura Magnasco ^2^, Silvia Dettori ^1,2^ and Matteo Bassetti ^1,2^**

^1^ Department of Health Sciences, University of Genova
^2^ Division of Infectious Diseases, San Martino Policlinico Hospital—IRCCS

**Objectives:** Isavuconazole (ISV) is the newest azole approved for treatment of invasive aspergillosis and mucormycosis. While therapeutic range has been defined for voriconazole (2–6 mg/L) and posaconazole (1–3.75 mg/L), therapeutic drug monitoring (TDM) of ISV is controversial since large studies did not show association between blood levels and efficacy or toxicity.

The aim of this study was to report ISV blood levels in real life, and to compare them between intensive care unit (ICU) and non-ICU patients.

**Materials & Methods:** ISV levels are routinely tested in our centre HPLC method. The lowest limit of quantification was set at 0.15 mg/L. ISV blood levels obtained between January 2017 and May 2021 were analysed and compared between those obtained from patients in ICU vs. others. Chi-square/Fisher’s exact test and Mann-Whitney test were used for categorical and continuous variables, respectively. Levels of ISV <1 mg/L were defined as low.

**Results:** A total of 431 samples from 63 patients were identified. Patients were mostly male (45, 71%), with the median age of 63 years (range, 24–83). Overall, 354 samples were from 43 patients not admitted to ICU (37 had haematological malignancies, HM), while 77 samples were from 20 patients admitted to the ICU (including 14 patients with COVID-19).

Since ICU patients had significantly shorter duration of ISV therapy compared to patients with HM (*p* < 0.001), only samples from the first two months of treatment were selected for this analysis.

Thus, 265 samples from 63 patients were included in this study (Table 1). Median ISV blood levels were 3.3 mg/L (range 0–14.52), and they were lower in samples from ICU patients compared to samples from non-ICU patients (1.95 mg/L, range 0–8.48 vs. 4.1 mg/L, range 0–14.52, *p* < 0.001), Figure 1.

Significantly more samples with ISV level <1 mg/L were from ICU patients compared to non-ICU cohort (*p* = 0.0001). The management of patients with ISV levels <1 mg/L consisted most frequently (63.6%) of switching to other antifungal after repeatedly low ISV levels (Table 1). Two patients with HM experienced relapse/breakthrough invasive fungal disease (IFD) while having ISV <1 mg/L during the first and second month of ISV administration.

Among ICU patients, samples drawn while patients were receiving renal replacement therapy (RRT) showed more frequently ISV levels <1 mg/L (7/24, 29.2%) compared to samples drawn while patients were not receiving RRT (7/46, 15.2%, *p* = 0.212). However, 3 of 6 patients who did not achieve ISV >1 mg/L were not receiving RRT and their BMI was between 25 and 27.

**Conclusions:** ISV levels were frequently <1 mg/L during the first two months of treatment in the ICU patients, while there were adequate in most of patients outside the ICU, with few cases of relapse/breakthrough IFD during ISV levels <1mg/L. Reasons for low ISV levels in the ICU remain to be determined.

## P399 A Large Retrospective Assessment of Voriconazole Exposure in Patients Treated with Extracorporeal Membrane Oxygenation

**Ruth Van Daele ^1,2^, Britt Bekkers ^2^, Mattias Lindfors ^3,4^, Lars Mikael Broman ^3,4^, Alexander Schauwvlieghe ^5,6^, Bart Rijnders ^6^, Nicole GM Hunfeld ^7^, Nicole P. Juffermans ^8^, Fabio Silvio Taccone ^9^, Carlos Antônio Coimbra Sousa ^9^, Luc-Marie Jacquet ^10^, Pierre-François Laterre ^11^, Eric Nulens ^12^, Veerle Grootaert ^13^, Haifa Lyster ^14,15^, Anna Reed ^15,16^, Brijesh Patel ^16,17^, Philippe Meersseman ^18^, Yves Debaveye ^19,20^, Joost Wauters ^18,21^, Christophe Vandenbriele ^17,22,23^ and Isabel Spriet ^1,2^**

^1^ Department of Pharmaceutical and Pharmacological Sciences, KU Leuven
^2^ Pharmacy Department, University Hospitals Leuven
^3^ ECMO Centre Karolinska, Department of Pediatric Perioperative Medicine and Intensive Care, Karolinska University Hospital
^4^ Department of Physiology and Pharmacology, Karolinska Institutet
^5^ Department of hematology, Ghent University Hospital
^6^ Department of Internal Medicine, Erasmus MC, University Medical Center
^7^ Department of Intensive Care and Department of Hospital Pharmacy, Erasmus MC
^8^ Department of Intensive Care, Amsterdam University Medical Center
^9^ Department of Intensive Care, Hôpital Erasme, Université Libre de Bruxelles (ULB)
^10^ Cardiovascular Intensive Care, Cliniques Universitaires Saint-Luc
^11^ Department of Intensive Care, Cliniques Universitaires St-Luc, Université Catholique de Louvain
^12^ Laboratory Medicine, Medical Microbiology, Algemeen Ziekenhuis Sint-Jan, Brugge-Oostende
^13^ Pharmacy Department, AZ Sint-Jan Brugge-Oostende AV
^14^ Pharmacy Department, Royal Brompton & Harefield hospitals
^15^ Cardiothoracic Transplant Unit, Royal Brompton & Harefield Hospitals
^16^ Imperial College London
^17^ Department of Adult Intensive Care, The Royal Brompton and Harefield Hospitals
^18^ Medical Intensive Care Unit, University Hospitals Leuven
^19^ Intensive Care Unit, University Hospitals Leuven
^20^ Department of Cellular and Molecular Medicine, KU Leuven
^21^ Department of Microbiology and Immunology, KU Leuven
^22^ Department of Cardiovascular Sciences, KU Leuven
^23^ Department of Cardiovascular Diseases, University Hospitals Leuven

**Objectives:** Voriconazole is one of the first-line therapies for invasive pulmonary aspergillosis. Drug concentrations might be significantly influenced by the use of extracorporeal membrane oxygenation (ECMO). We aimed to assess the effect of ECMO on voriconazole exposure in a large patient population.

**Materials & Methods:** Critically ill patients from eight centers in four countries treated with voriconazole during ECMO support were included in this retrospective study. Voriconazole concentrations were collected in both the period on ECMO as before and after ECMO treatment. Multivariate analyses were performed to evaluate the effect of ECMO on voriconazole exposure and to assess the impact of possible saturation of the circuit’s binding sites over time.

**Results:** Sixty-nine patients and 337 samples (190 during and 147 before/after ECMO) were analyzed. The patients’ median [IQR] age and weight were 54 [42–60] years and 77 [65–95] kg and 67% were male. On admission, the median APACHE II score was 18 [14–23] and the median duration of ECMO therapy 19 [11–33] days. Subtherapeutic concentrations (<2 mg/L) were observed in 56% of the samples during ECMO and 39% without ECMO (*p* = 0.80). The median trough concentration, for a similar daily dose, was 2.4 [1.2–4.7] mg/L under ECMO and 2.5 [1.4–3.9] mg/L without ECMO (*p* = 0.58). Inter- and intrasubject variability (%CV) in the voriconazole trough concentration (corrected for the dose) were 47% and 78% on sampling days under ECMO and 46% and 60% for non-ECMO sampling. Neither ECMO nor squared day of ECMO (saturation) were retained as significant covariates on voriconazole exposure.

**Conclusions:** In contrast to previously published ex vivo studies and case reports, this study could not demonstrate an influence of ECMO on voriconazole exposure. There was a wide variability in voriconazole trough concentrations and a high proportion of subtherapeutic concentrations in critically ill patients, but ECMO did not significantly contribute to this. Therapeutic drug monitoring of voriconazole remains important, certainly in this severely ill patient population often presenting with a subtherapeutic exposure.

**Figure 1.** Boxplot; Voriconazole trough concentrations, corrected for dose 24 h, on ECMO vs. non-ECMO sampling days.

**Figure 2.** Voriconazole trough concentration in function of the day of ECMO with spearman-coefficient and *p*-value (n = 145). The dashed line represent the lower limit concentration of 2 mg/L.

## P400 In Vitro Postantifungal Effect of Amphotericin B against *Candida auris*

**Unai Caballero ^1^, Elena Eraso ^2^, Guillermo Quindós ^2^ and Nerea Jauregizar ^1^**

^1^ Department of Pharmacology, Faculty of Medicine and Nursing, University of the Basque Country (UPV/EHU)
^2^ Department of Immunology, Microbiology and Parasitology, Faculty of Medicine and Nursing, University of the Basque Country (UPV/EHU)

**Objectives:** *Candida auris* is a multi-drug resistant fungal pathogen. Amphotericin B is the first alternative to echinocandins for the treatment of invasive candidiasis caused by this species. Previous studies in our research group evidenced that this polyene exerts concentration-dependent fungicidal activity against *C. auris*. The objective of the present work was to evaluate the postantifungal effect (PAFE) of amphotericin B, as it may be helpful to further understand the dynamics of amphoterin B-*C. auris* interaction.

**Materials & Methods:** PAFE experiments were performed in microtitre plates, with six *C. auris* clinical blood isolates (Hospital La Fe, Valencia, Spain). Inoculum size ranged from 1 to 5 × 10^5^ CFU/mL and amphotericin B concentrations assayed were 0.25, 0.5, 1, 2 and 4 mg/L. After an incubation of 1 h at 37 °C, drug was removed from the medium by three centrifugation cycles (2000 rpm, 10 min each). Cell pellets were re-suspended in RPMI medium and added back to the microtitre plates. Sample for viable counts were taken at 0, 2, 4, 6, 8, 24 and 48 h after re-suspension, plated in triplicate onto Sabouraud dextrose agar and incubated for 24–48 h at 37 °C. Experiments were performed in duplicate for each isolate on different days. PAFE was calculated as follows: PAFE = T − C. Where “T” is the time needed by the treated fungal cultures to growth 1 log after drug removal and “C” is the time needed by the control group to growth 1 log after last washout. Additionally, maximum log reductions of time-kill and PAFE experiments were compared and used to calculate a PAFE/time-kill index to determine the percentage of cell killing that could be attributed to the PAFE.

**Results:** Short PAFEs were achieved with the concentrations of 2 and 4 mg/L of amphotericin B, with range values from 3.5 to 6.5 h. Fungal killing was not observed during PAFE experiments, therefore, it could be concluded that PAFE had no role in the fungicidal activity of amphotericin B.

**Conclusions:** Amphotericin B did not exert a signifficant in vitro PAFE against *C. auris*. Nevertheless, further studies are needed to fully understand the antifungal susceptibility of this emerging pathogen.

**Funding:** This study was financed by GIC15/78 IT-990-16 (Gobierno Vasco-Eusko Jaurlaritza). Unai Caballero has received a grant from UPV/EHU (PIF 17/266).

## P405 *Tinea faciei* Possibly Related to Mask Usage during the COVID-19 Pandemic: A Case Report

**Maria Lúcia Scroferneker ^1,2^, Rodrigo Vetoratto ^1,3^, Alessandra Koehler ^1^, Bruna de Matos Bauer ^3^, Joana Roberta Fitz ^3^, Paulo Cezar de Moraes ^1,4^, Amanda Carvalho Ribeiro ^5^ and Danielle Machado Pagani ^6^**

^1^ Postgraduate Program in Medicine: Medical Sciences, Universidade Federal do Rio Grande do Sul
^2^ Department of Microbiology, Immunology and Parasitology, ICBS, Universidade Federal do Rio Grande do Sul
^3^ Dermatology Service from the Hospitalar Complex Santa Casa de Misericórdia de Porto Alegre
^4^ Sanitary Dermatology Outpatient Clinic from Porto Alegre
^5^ Graduation in Pharmacy, Universidade Federal do Rio Grande do Sul
^6^ Postgraduate Program in Agricultural and Environmental Microbiology, Universidade do Rio Grande do Sul

**Case report:** A previously healthy female patient, 31 years old, consulted for the main complaint of an erythematous and pruritic skin lesion in the right hemiface of onset and progressive growth for approximately 1 month. She reports that, in this period, she consulted with a dermatologist, who prescribed mometasone cream for 7 days, with no improvement in the lesion. On physical examination, she had a faded erythematous lesion, with poorly delimited edges and with fine flaking occupying practically the entire right hemiface, exactly at the region of the face covered by the mask. Due to the clinical suspicion of tinea, a direct mycological examination was performed during the consultation, identifying hyphae typical of dermatophyte fungi. In the cultural examination, there was growth of the fungus *Trichophyton mentagrophytes* after 2 weeks. Treatment with fluconazole 150mg orally weekly for 4 weeks was started, with improvement of the lesions.

**Discussion and conclusions:** Tinea faciei is a relatively rare dermatophytosis with classical presentation as erythematous-scaling lesions with central clearing. This dermatophytosis is often misdiagnosed, with the wrong indication for treatment with corticosteroids. The use of these drugs can mask the typical characteristics of tinea, making it “icongnito”, which can lead to greater difficulty in diagnosis and progression of the original fungal infection. The main etiologic agents are fungi of the genus *Trichophyton*. There are already published case series of tinea faciei that were attributed to the use of masks during the COVID-19 pandemic. The masks, especially those made with occlusive materials, create a humid and warm environment ideal for the growth of fungi. In addition, incorrect hygiene, such as washing together with other clothes that may be contaminated with dermatophyte fungi, is also a factor that can predispose to infections. Despite this, it is not possible to confirm with certainty, through case reports, the causal relationship between the use of a mask and the appearance of dermatophytosis. Even so, some authors have already suggested calling this new variation of tinea faciei “mask tinea” and this clinical presentation should be considered by dermatologists during the COVID-19 pandemic.

## P406 Emergence of Difficult-To-Treat *Tinea corporis* due to Isolates of *Trichophyton mentagrophytes* Complex in Paris, France

**Sarah Dellière ^1^, Brune Joannard ^1^, Mazouz Benderdouche ^1^, Anselme Mingui ^1^, Maud Gits-Muselli ^1^, Samia Hamane ^1^, Alexandre Alanio ^1^, Antoine Petit ^2^, Germaine Gabison ^2^, Martine Bagot ^2^ and Stéphane Bretagne ^1^**

^1^ Service de Parasitologie-Mycologie, Hôpital Saint-louis
^2^ Service de Dermatologie, Hôpital Saint-Louis

**Objectives:** Dermatophytes are common keratinophilic fungi responsible for superfical disease involving skin, hair and nails called tinea corporis, tinea capitis and tinea unguium. Dermatophytosis is generally regarded as a relatively benign health problem, easily amenable to the available antifungals. However, in the past decade, outbreaks of extensive tinea corporis with reduced antifungal susceptibility have been described from the Indian subcontinent due to strains from the *Trichophyton mentagrophytes* species complex (TMSC). We describe a serie of difficult to treat tinea corporis in France and investigate the microbiological origin of this resistance.

**Materials & Methods:** For a two year-period (2018–2019), 2282 patients consulted for dermatophytosis in our center in Paris, France. TMSC was identified in 350 patients and 7 (2.0%) had clinically resistant tinea corporis. Squalene epoxidase (SQLE) gene and ITS were sequenced for all 7 corresponding isolates as well as 8 consecutively isolated TMSC considered as controls. If polymorphism was identified in SQLE gene, minimal inhibitory concentrations (MICs) to terbinafine, itraconazole, voriconazole, and posaconazole were determined using the EUCAST broth microdilution method.

**Results:** All seven patients originated from the Indian subcontinent. Cutaneous lesions were multiple and extensive associated with untractable pruritus. All patients showed poor or no response to terbinafine. Three out of four patients who received itraconazole improved or healed temporarily but relapsed within one year after the end of treatment. SQLE sequenging showed mutation F397L (n = 4), L393S (n = 1) associated with high MIC to terbinafine and A448T (n = 2) associated with high MICs to azoles for one of the isolates. Polymorphism (K276N) was also observed in two of the eight control isolates. Internal transcribed spacer sequencing confirmed that the dermatophyte of the 7 clinically resistant tinea corporis was *Trichophyton mentagrophytes* ITS Type VIII already spreading in the Indian subcontinent.

**Conclusions:** *Trichophyton mentagrophytes* ITS type VIII has recently been individualized in a specific species called *Trichophyton indotineae*. It has been reported in several European countries including Germany, Belgium, Switzerland, Denmark, Poland and Switzerland. Although most cases were reported in patients returning or originating from the Indian subcontinent, two cases have been identified in German-born residents who had not travelled generating fear of extension outside the initial epidemic focus. Surveillance is necessary and sould be focused on the identification of *T. mentagrophytes* ITS type VIII/*T. indotineae* rather than SQLE sequences or MICs determinations since all mutations were not associated to high MICs in vitro. Optimal treatment when terbinafine resistance is demonstrated remains to be established in regard to the high rate of itraconazole failure.

## P408 Prevalence of Supeficial Mycoses among Medical Students Population in Belgrade (Serbia)

**Eleonora Dubljanin ^1^, Teodora Crvenkov ^2^, Isidora Vujcic ^3^, Sandra Sipetic Grujicic ^3^, Valentina Arsic Arsenijevic ^1^, Sanja Mitrovic ^1^ and Aleksandar Dzamic ^1^**

^1^ Institute of Microbiology and Immunology, Faculty of Medicine, University of Belgrade
^2^ Faculty of Medicine University of Belgrade
^3^ Institute of Epidemiology, Faculty of Medicine University of Belgrade

**Objectives:** Superficial mycoses are one of the most common skin conditions nowadays representing the fourth most common cause of illness worldwide. Despite numerous advances in health and medical sciences, superficial fungal infecmycoses retained their position as one of the most important skin diseases and continue to be a significant health problem. The aim of this study was to evaluate the prevalence od superficial fungal infectimycoses in the medical students population and to identify the causative fungi and significant potential risk factors for the emergence of these diseases.

**Materials & Methods:** In November and December 2017 cross-sectional study was condacted among fourth-year medical students at the University of Belgrade. Data were collected by anonymous questionnaire: demographic and morphometric data, avarege grade, lifestyle and behavioral practice, possession of pets and/or domestic animals, excessive perspiration, family history of superficial mycoses, as well as personal hystory of superficial mycoses including exsitance of superficial mycosis in time of testing with indicating exact localization. Every student with superficial mycosis in time of testing were refered to mycology laboratory where samples were examinated by microscopy and fungal culture. Statistical analyses were performed using descriptive statistics, univariate and multivariate logistic regression analysis.

**Results:** A total of 493 4th grade medical students from Belgrade University were surveyed, and 469 returned correctly filled questionnaires (response rate 95.13%). Out of them 136 (29%) reported having ever superficial mycoses in lifetime. Participants most frequently reported mycoses in genital area (55/136), feet (29/136), and skin (17/136). Students who reported having ever superficial mycoses in lifetime had significantly lower average grade (*p* = 0.004), higher BMI (*p* = 0.023), excessive perspiration (*p* < 0.001), they were more frequently involved in smoking (*p* = 0.007), and alcohol consumption (*p* = 0.013). They were significantly more likely to have pets and/or domestic animals (*p* = 0.024), wear synthetic clothing and/or footwear (*p* = 0.045), and have positive family history of superficial mycoses (*p* < 0.001).

Eight variables that showed statistical significance were included in the multivariate logistic regression analysis and independent risk factors for superficial mycoses were: family history of superficial mycoses (OR = 2.671; *p* = 0.001), and excessive perspiration (OR = 1.768; *p* = 0.027).

A total of 41/136 (30.15%) students reported having signs and symptoms of superficial mycoses in the moment of testing, while in 33 students who showed for testing were laboratory confirmed. In 9/33 (27.27%) students each superficial mycoses were localized on skin and feet, followed by genital area (n = 7, 21.21%), and nails (n = 5, 15.15%). The most frequent causative agents of superficial mycoses were dermatophytes in 22/33 (66.67%), followed by *Candida* spp. in 8/33 (24.24%), and *Malassezia* spp. in 3/33 (9.09%) patients. Dermatophytes were the only fungi isolated from feet and nail samples. The largest number of different fungal species were isolated from skin and genital area.

**Conclusions:** The results of this study indicate that a quarter of medical students had ever in lifetime superficial mycoses. Since fungal diseases are widespread in the population, it is necessary to increase the awareness of the population about the potential risk factors in order to be able to apply adequate preventive measures.

## P409 Infrared Multispectral Imaging Differentiates between Vital and Non-Vital *T. rubrum*

**Rasmus L. Pedersen^1^, Christian Pedersen ^1^, Peter Tidemand-Lichtenberg ^1^, Gregor BE Jemec ^2,3^, Maiken C Arendrup ^2,4,5^ and Ditte Saunte ^2,3,4^**

^1^ DTU Fotonik, Dept. of Photonics Engineering, Technical University of Denmark
^2^ Department of Clinical Medicine, Faculty of Health Science, University of Copenhagen
^3^ Department of Dermatology, Zealand University Hospital
^4^ Unit of Mycology, Department of Microbiological Surveillance and Research, Statens Serum Institut
^5^ Department of Clinical Microbiology, University Hospital Rigshospitalet

**Objectives:** Clinical evaluation of treatment efficacy in onychomycosis is difficult due to slow regrowth of the nail. It is currently performed by culturing which allows fungal vitality testing, but takes up to 4 weeks before the result is available (4). We explore if an infrared multispectral imaging system (Figure 1), enabling transmission spectroscopy of *Trichophyton (T.) rubrum* cultures on a microscopy slide, is able to differentiate between vital and non-vital *T. rubrum*. The method is under development and this is the first explorative study testing its application in mycology.

**Materials & Methods:** The reference strain *T. rubrum* (SSI 14438) was cultured on Sabouraud-glucose-agar supplemented with cycloheximide and chloramphenicol (SSI, Diagnostika, Hillerød, Denmark) and incubated at 25 °C for 4 weeks. The non-viable culture was produced by adding formaldehyde 4% (Sarstedt AG & Co, Nürnbrecht, Germany) to the culture until the mycelium was covered where after the plate was sealed for 7 days.

The culture samples was sandwiched in a thin layer (10’s of µm) between two infrared transparent microscope CaF_2_ slides. The system scans cross-sectional areas of 1x1 mm^2^, with a spatial resolution of 10 µm, where 41 monochromatic images in the range from 1000 to 1800 cm^−1^ are acquired. Based on image analysis, transmission data of the samples were extracted. Multispectral infrared absorption images of the samples were acquired in the spectral range from 5.5 to 10 µm (1000 to 1800 cm^−1^), followed by simple multispectral post-processing aiming to differential between vital and non-vital cultures (Figure 1). The analysis was performed twice in the viable and non-viable culture, respectively, in six selected areas.

**Results:** Figure 2 shows spectral data from a 1 mm^2^ sample region (100 × 100 positioner (pixels)). The curves V1 (viable) and V2 (non-viable) shows measured data taken from the same culture separated by 15 days. A clear separation between vital and non-vital samples was observed, particularly at the higher wavenumbers (1650 to 1800 cm^−1^ range). In contrast, a small variation between the vital/vital samples and non-vital/non-vital samples was found.

**Conclusions:** Our pilot study suggests, that multispectral infrared technology has a promising potential for differentiation of biological samples such as vital and non-vital cultures, particularly if the culture type is known. The advantage is the fast confirmation or exclusion of vitality. With a uniform sample thickness (on the order of 10 µm) applied across a small area (1 mm^2^) on the slide, the scan time for assessing the vitality of a culture can be as low as a few minutes. The very small sample volume needed for the analysis potentially eliminates the need to culturing of the sample prior to analysis, but is dependent on representative sample material.

Further studies are warranted to explore the performance in different stages of fungal growth, under influence of antifungals and applied directly on nail material. Moreover, fungal species identification using the full scan range, eventually expanded beyond the spectral coverage of the existing system might be a future application of the technique that deserves investigation.

## P410 Description of Two Nov. Species of *Syncephalastrum* Isolated from Clinical Human Samples

**Jihane Kabtani**

Stéphane Ranque

**Introduction:** Mucormycosis is known to be a rare opportunistic infection caused by *Syncephalastrum* species, which are mucorales fungi that belong to the class of *Zygomycetes*. These molds are generally seen as clinical contaminants and are rarely known to cause human diseases. However, these last years, case reports of human infections due to *Syncephalastrum* genus are increasing, especially in immunocompromised hosts.

**Objectives:** In this study, two Nov. species of *Syncephalastrum sp.,* previously isolated from human nails of two different patients at the Mycological laboratory IHU- Mediterranée Infection, la Timone Marseille, are described.

**Methods and materials:** We used here several methods of genetic and phenotypic characterization. For the molecular methods, a multilocus sequencing was achieved, targeting the Internal Transcribed Spacers (ITS1/ITS2), a fragment of the Translation Elongation Factor 1-alpha gene (TEF-1-α), a fragment of β-tubulin gene (TUB2) and D1/D2 domains of the ribosomal DNA large-subunit (LSU).

For the phenotypic analysis, we used the MALDI-TOF MS (Matrix Assisted Laser Desorption Ionization—Time of Flight) to highlight the protein expression, EDX (Energy-Dispersive X-ray Spectroscopy) for performing a chemical mapping, and the Biology’s advanced phenotypic technology for carbon sources assimilation. For the morphological features analysis, we visualized fungal structures by Optical Microscopy (MO) and SEM (Scanning Electron Microscopy).

**Results:** Each one of the new species seems to be close to a different type species of the *Syncephalastum* genus, which, for reminder, is only composed of two species; *Syncephalastrum racemosum* and *Syncephalastrum monosporum*.

**Conclusions:** The combination of all these methods helped us to describe and individualize these two Nov. species of *Syncephalatrum*.

**Keywords:** *Syncephalastrum*; mucormycosis; genotype; phenotype

## P411 Evaluation of Terbinafine Resistance of *Trichophyton rubrum* and *T. interdigitale* in Portugal and Characterization of the Associated Resistance Mechanisms

**Camila Henriques ^1^, Helena Simões ^1^, Cristina Verissímo ^1^ and Raquel Sabino ^1,2^**

^1^ National Institute of Health Dr. Ricardo Jorge
^2^ Instituto de Saúde Ambiental, Faculdade de Medicina, Universidade de Lisboa

**Objectives:** Superficial mycoses caused by *Trichophyton rubrum* are among the most common infections worldwide. In Portugal, it is estimated that 1,510,391 of Portuguese suffer from dermatophytosis, corresponding to an incidence of 14,300 per 1,000,000 inhabitants.

Superficial mycoses caused by *Trichophyton rubrum* are among the most common infections worldwide, being difficult to treat and often associated with recurrences after interruption of the antifungal therapy. Terbinafine is one of the allylamine antifungal agents whose target is squalene epoxidase (SQLE). This agent has been extensively used in the therapy of dermatophytes’ infections. The emergence of resistance to terbinafine in *Trichophyton* species has been recently described and is associated with point mutations in the SQLE gene. The increasing number of patients with *Tinea pedis* or *Tinea unguium* resistant to terbinafine treatment prompted us to screen the terbinafine resistance in *Trichophyton* isolates obtained by culture at the Mycology Reference Laboratory of the National Institute of Health Doctor Ricardo Jorge. We aimed to determine the frequency of terbinafine resistance and associated mechanisms of resistance.

**Materials & Methods:** Dermatophytes collected during 2017–2020 were identified by culture and mass spectrometry (MALDI-TOF-MS) and/or by sequencing the ITS (Internal Transcribed Spacers) region of the rDNA. All isolates were grown onto agar supplemented with terbinafine (0.06 and 0.125 mg/L) in order to detect potential resistant isolates. Antifungal susceptibility testing was performed following CLSI M38A2 broth microdilution method. The squalene epoxidase gene (SQLE) was sequenced and mutations described as conferring resistance to terbinafine were screened.

**Results:** Among 102 *T. rubrum* and 17 of *T. interdigitale* isolates that identified and further tested in what concerns to their antifungal susceptibility, 1 *T. rubrum* isolate (≈1%) and 3 *T. interdigitale* isolates (≈18%) showed to be resistant to terbinafine by screening agar. According to the microdilution method, 2.44% of the isolates were less susceptible to terbinafine. Overall, three isolates (1 *T. rubrum* and 2 *T. interdigitale*) showed high terbinafine resistance (MICs, 4 to ≥ 8 mg/L) and one isolate (*T. interdigitale*) displayed moderate terbinafine resistance (1 to 2 mg/L).

The sequencing of the SQLE gene allowed the detection of the following single point mutations: in one *T. rubrum* and in one *T. interdigitale*, at the position 1189 in the ORF of the SQLE gene (corresponding to a substitution of a phenylalanine at the position 397 by an isoleucine or by a leucine, respectively). In the remaining resistant *T. interdigitale* isolates, no mutations were found in the gene encoding SQLE.

Although the most common substitutions that lead to higher resistance to terbinafine are L393F and F397L, the F397I substitution detected in our study suggests to be associated with high terbinafine resistance.

**Conclusions:** This study allowed us to detect, for the first time in Portugal, *Trichophyton* isolates resistant to terbinafine. It was also possible to estimate, within the studied isolates, a resistance frequency of 2.4%.

Taken together, our results prompt the current knowledge about the necessity of antifungal susceptibility testing to select effective strategies for management of clinical cases of dermatophytosis not only in Portugal but worldwide.

## P412 Geophylic Dermatophytes in Beach Sand: A Public Health Problem

**Mário Bonci ^1^, Daniel Abreu ^2^, Lucas Oliveira ^3^, Clara Mendes ^4^, Regina Ramos ^1^, Rennan Santos ^1^, Maria José Silveira ^5^, Luciana Ruiz ^6^, Águida Oliveira ^4^, Francisco de Assis Baroni ^4^ and Claudete Paula ^1^**

^1^ School of Dentistry, University of São Paulo
^2^ COPPE, Federal University of Rio de Janeiro
^3^ School of Chemistry, Federal University of Rio de Janeiro
^4^ Veterinary Institute, Federal Rural University of Rio de Janeiro
^5^ CONTROLBIO Microbiological Technical Assistance SS LTDA
^6^ Biomedical Sciences Nucleus, Adolfo Lutz Institute

**Objectives:** Despite the inherent rules of the beaches, people have the habit of taking companion animals to share leisure time without worrying about public health. There are practically no reports of dermatophytes isolation from beach sand. Thus, we aimed to demonstrate the presence of geophilic dermatophytes in the sands of Pajuçara beach, Maceió, AL, Brazil.

**Materials & Methods:** The collections were carried out on a single day, in October 2019. Pajuçara, known and tourist resort of Maceió, was divided into 13 points approximately 400 m apart, from the Sampaio Marques Avenue and ending on the edge of Ponta Verde beach. Each point was subdivided into three subpoints, with one collection for each. The first subpoint was established 5 m from the sand limit and the so-called “Calçadão”. The second subpoint was calculated at a distance of 10 m from the first and the third at 20 m from the first, always in a straight line towards the sea. Distances were also modified at the end of the beach, due to the narrower area. For the collections, the immediately superficial sand was removed, removing it at a depth of 5 cm. We use “universal collector” type bottles, noting a number corresponding to the collection point. The samples were transported to the Laboratory of Pathogenic and Environmental Yeasts of the Department of Veterinary Microbiology and Immunology, Veterinary Institute, Federal Rural University of Rio de Janeiro. For each sample, the modified Vanbreuseghem technique was used. In this specific case, the soil was replaced by sand samples mixed with autoclaved hair, obtained from horse hair, with periodic humidification using sterile distilled water to prevent the loss of fungal viability. The incubation took place at room temperature and the observation of the structures by making slides with lactophenol cotton blue stain.

**Results:** Of the 13 points worked out, the presence of geophilic dermatophytes was found in 9 (69.23%). Considering the 39 subpoints, the results were positive in 13 (33.3%). *Nannizzia gypsea* was isolated in seven of the points and terrestrial *Trichophyton* in two. The greatest isolation occurred from wet samples. The presence of dermatophyte fungi in the sands of Pajuçara indicates the presence of keratin sources of desquamative origin from humans and animals, as well as from fish and crustaceans. However, it is a source of environmental infection for visitors, especially those who have the habit of direct contact with the body on the sand. The moisture and abrasiveness of the sand associated with wet or macerated skin provides a favorable environment for the installation of fungi.

**Conclusions:** Dermatophyte fungi are also present in beach sands, with a strong potential for spreading and infection for those visiting such places, mainly due to the abrasiveness of the sand associated with wet and macerated skin. There should be guidance regarding the risk of users’ contact with the sand and bathers should be advised not to take dogs to the showers.

## P413 Genotyping *Microsporum canis* from Domestic Felines across the United States

**Alex Moskaluk ^1^, Erick Gagne ^2^ and Sue VandeWoude ^1^**

^1^ Colorado State University
^2^ University of Pennsylvania

**Objectives:** Our objectives were to categorize *Microsporum canis* subtypes from domestic cats collected from across the United States and compare strains to geography, disease severity and other phenotypic characteristics.

**Materials & Methods:** In collaboration with 5 locations across the US, we collected over 200 hair samples from cats suspected of dermatophytosis. We developed a clinical questionnaire that was completed for each patient to capture clinical presentation information including age, sex, breed, presence of lesions, lesion types, number of lesions, size of lesions, severity of disease, prior housing situation, prior anti-fungal treatments, and concurrent diseases. Hair samples from suspected dermatophytosis patients were cultured and DNA was extracted from dermatophyte colonies. Using multiplex PCR, 8 microsatellite regions were analyzed for allele size using Geneious. Mating type for each dermatophyte sample was determined using conventional PCR for the alpha domain gene. After determining the alleles for each microsatellite region, samples genotypes were determined and principle component analysis (PCA) plots were constructed using adegenet package in R.

**Results:** Based on the microsatellite regions and mating types, we have identified >30 different genotypes of *M. canis* from among 150 unique isolates. Over 90% of *M. canis* samples were positive for the alpha domain gene, indicative of the negative mating type. Comparisons of the genotypes of *M. canis* to clinical presentation data did not reveal substantive differences. However, geographic analysis indicated strain clustering at certain locations.

**Conclusions:** In the US domestic cat populations, *M. canis* positive mating type is at a very low abundance as has been reported for other regions, indicating widespread loss of sexual reproduction characteristics. We identified well over 2 dozen *M. canis* genotypes circulating in the US. Analysis conducted to date does not correlate genotype to clinical presentation; however, geographic associations indicate local transmission of ringworm is predominant, and may indicate *M. canis* adaptation to different climates or ecosystems.

## P414 Assessment of the Importance of Determining the Value of the SCIO Index in Patients with Laboratory Confirmed Onychomycosis

**Eleonora Dubljanin ^1^, Tamara Sura ^2^, Aleksandar Dzamic ^1^, Isidora Vujcic ^3^, Sandra Sipetic Grujicic ^3^, Stefan Mijatovic ^1^ and Ivana Colovic Calovski ^1^**

^1^ Institute of Microbiology and Immunology, Faculty of Medicine, University of Belgrade
^2^ Faculty of Medicine University of Belgrade
^3^ Institute of Epidemiology, Faculty of Medicine University of Belgrade

**Introduction:** Onychomycosis is the most common disorder that affects nail apparatus. This chronic fungal infection is characterized by increasing incidence, and the global prevalence is estimated to be 5.5%. The aim of our study was to perceive objectively severity of onychomycosis by calculating Scoring Clinical Index of Onychomycosis (SCIO) and to correlate this index with accurate laboratory diagnosis in patients with onychomycosis.

**Materials & Methods:** A total of 417 patients with laboratory confirmed onychomycosis were included in the study. For each patient in the study basic demographic data, site of infection, the most affected nail with onychomycosis, clinical presentation and type of onychomycosis were collected, as well as degree of nail involvement, degree of hyperkeratosis, number of abnormal-appearing nails, and data relating to presence of nail deformations, pain and esthetic problems. In order to objectively perceive the severity of disease SCIO index was calculated for every patient separately. Nail samples were obtained by scrapings/clippings depending on clinical type of onychomycosis. Identification of causative fungi was done by microscopy examination of nail sample and fungal culture. A descriptive analysis was used to detail the main characteristics of the study population. Statistical analyses were performed using chi-square and Fisher’s test.

**Results:** The majority of patients had distal and lateral subungual onychomycos (95.44%). Female patients were more commonly affected, with female to male ratio in patients with laboratory confirmed onychomycosis of 1.24:1. Majority of patients had onychomycosis on toenails found in 375/417 (89.93%), only fingernails were affected in 29/417 (6.95%), while 7/417 (1.68%) patients had onychomycosis both on fingernails and toenails. In 100/417 (23.98%) patients onychomycosis was localized on two nails, followed with 77/417 (18.46%) patient who had diseased just one nail. The most affected nail was big toenail found in 261 (62.59%) patients with laboratory confirmed onychomycosis.

Male patients had significantly more nails affected with onychomycosis compared with female patients (*p* = 0.011), while female had significantly more often onychomycosis on fingernails 2–5 (*p* < 0.05), and they reported significantly more often pain (*p* < 0.05) and esthetic problems (*p* < 0.05). Mean SCIO index value in patients with onychomycosis was 16.76. Dermatophytes were isolated in 383/417 (91.85%) patients, while *Trichophyton rubrum* was the most frequent one (337/383). In 22/417 (5.27%) patients causative agents of onychomycosis were yeasts, while non-dermatophyte moulds were identified in 12/417 (2.88%). *Candida albicans* was the most frequent among yeast isolated in 16/22 (72.73%), while *Aspergillus fumigatus* was the most common among non-dermatophyte moulds isolated in 4/12 (33.33%). Mean SCIO index value was highest in onychomycosis caused by dermatophytes, followed by non-dermatophyte moulds, and yeast with values 17.37, 10.75, and 8.66, respectively. In patients with onychomycosis caused by dermatophytes SCIO index had significantly higher values (*p* = 0.032) compared with patients who had onychomycosis caused by yeasts and non-dermatophyte moulds.

**Conclusions:** Comprehensive understanding of disease characteristics will allow introduction of individualized treatment plan for each patient based on proper fungal identification and a standardized method of evaluating disease severity that could help the patient achieve a complete cure.

## P415 Infectious Synergism between *C. albicans* and *S. aureus*: Exploring Underlying Mechanisms and Treatment Options

**Katrien Van Dyck ^1,2^, Felipe Viela ^3^, Marion Mathelié-Guinlet ^3^, Liesbeth Demuyser ^1,2^, Esther Hauben ^4^, Mary Ann Jabra-Rizk ^5,6^, Greetje Vande Velde ^7^, Yves F. Dufrêne ^3^, Bastiaan P. Krom ^8^ and Patrick Van Dijck ^1,2^**

^1^ Laboratory of Molecular Cell Biology, Institute of Botany and Microbiology, Department of Biology, KU Leuven
^2^ VIB Center for Microbiology
^3^ Louvain Institute of Biomolecular Science and Technology (LIBST), UC Louvain
^4^ Laboratory for Pathology, UZ Leuven and Department of Imaging and Pathology, Translational Cell and Tissue Research
^5^ Department of Oncology and Diagnostic Sciences, Dental School, University of Maryland
^6^ Department of Microbiology and Immunology, School of Medicine, University of Maryland
^7^ Biomedical MRI/MoSAIC, Department of Imaging and Pathology, KU Leuven
^8^ Department of Preventive Dentistry, Academic Centre for Dentistry Amsterdam (ACTA), Vrije Universiteit Amsterdam and the University of Amsterdam

**Objectives:** As the oral cavity harbors a tremendous number of microorganisms, *Candida albicans* predominantly co-exists with other commensal bacteria in complex communities. Synergistic interactions between these species can be associated with enhanced pathogenicity, altered infection outcomes and high mortality rates. Moreover, co-infections are often accompanied with increased drug tolerance which makes their treatment very challenging. Therefore, investigating these interspecies interactions is crucial to understand the impact on human health and to develop innovative treatment strategies.

An interesting infectious synergism is observed between the fungus *Candida albicans* and the bacterium *Staphylococcus aureus*, both commensals of the oral cavity. In previous work, a mouse model was developed to study oral co-infections with both species. It was shown that the superficial infection oropharyngeal candidiasis predisposes for a higher colonization and secondary systemic infection of *S. aureus*. Recently, we elucidated the mechanism of this *C. albicans*-mediated dissemination of *S. aureus* from the oral cavity to the kindeys. In the present study, we are exploring an innovative treatment strategy based on a novel endolysin to overcome the consequences of this infectious synergism.

**Materials & Methods:** We used static and dynamic adhesion assays to identify *C. albicans* adhesins involved in the interaction with *S. aureus*. In addition, we used in vitro phagocytosis experiments with macrophages, we altered the level of immunosuppression and we studied the secretion of candidalysin to elucidate a possible role of the host immune response in this mechanism. The experiments were validated in vivo using the established mouse model of oral *C. albicans—S. aureus* co-infection. Finally, we used the same mouse model to validate a treatment option based on the novel anti-*S. aureus* endolysin, XZ.700.

**Results:** We identified *C. albicans* adhesins involved in the interaction with *S. aureus* and we found that this physical interaction is crucial for subsequent bacterial dissemination. Remarkably, we discovered that the host immune response plays a critical yet paradoxical role in this process. In addition, secretion of candidalysin, the *C. albicans* peptide responsible for immune activation and cell damage, is required for *C. albicans* colonization and subsequent bacterial dissemination. The physical interaction of *S. aureus* with *C. albicans* enhances bacterial uptake by phagocytic immune cells, thereby enabling dissemination. Finally, using the mouse model of oral co-infection we have established that treatment with XZ.700 efficiently reduces oral colonization of *S. aureus* and completely prevents *S. aureus* dissemination and symptoms of sepsis when co-infected with *C. albicans*.

**Conclusions:** Polymicrobial diseases are increasingly recognized in clinical settings, however, effective treatment strategies are lacking. Therefore, it is of great importance to understanding the complex mechanisms of interaction between fungal and bacterial species in different niches of the host. Our findings highlight the complexity of the interaction between *C. albicans* and *S. aureus* during an oral co-infection, and identify a potential paradoxical role for the host immune response. Moreover, we discovered an innovative and easy to implement treatment strategy which prevents bacterial dissemination and disease.

## P416 The Levels of Interferon-Gamma and Interferon-Alpha of Patients with Recurrent Vulvovaginal Candidiasis

**Elena Shagdileeva, Alena Buzmakova, Oksana Zhorzh, Yulia Dolgo-Saburova, Ekaterina Frolova, Larisa Filippova, Aleksandra Uchevatkina, Olga Shurpickaya, Ilya Bosak, Irina Vybornova, Natalya Vasilyeva and Nikolay Klimko**

North-West State Medical University named after I. I. Mechnikov

**Objectives:** Recurrent vulvovaginal candidiasis (RVVC) remains a challenge to manage in clinical practice. Data on immunologic responses in patients with RVC are limited.

**Materials & Methods:** The prospective study from December 2018 to February 2021. IFN-γ and IFN-α levels were measured in vaginal flushes and blood cell supernatants by an enzyme-linked immunosorbent assay (ELISA) according to the manufacturer’s instructions (Vector Best, USA).

**Results:** In the prospective study were included 20 woman with RVVC, the median age was 32.5 y (23–44). The frequency of relapses RVVC was from 6 to 10 times in 15% patients, every month relapses were in 85% patients. The main clinical symptoms were white vaginal discharge (100%), vulvovaginal pruritus (85%), burning (80%), and pain during urination (10%). The pathogens of RVVC were *Candida albicans* (95%) and *C. glabrata* (5%). In vitro resistant to fluconazole were 45% pathogens. In the immunology surveys, interferon-gamma (IFN-γ) production was low: local in 95% woman, median IFN-γ level—9.8 ± 3.1 pg/mL (normal range: 20–50), and systemic in 75% woman, median IFN-γ level—782.0 ± 91.2 pg/mL (normal range: 1000–5000). Increased levels of interferon-alpha (IFN-α) in vaginal samples were observed in 75% patients: median IFN-α level—14.4 ± 7.5 pg/mL (normal range: <5). IFN-α systemic production was within the normal range in 100% patients: median IFN-α level—228.9 ± 25.6 pg/mL (normal range: 100—500 pg/mL). Antifungal treatment was used in 100% patients: fluconazole (oral)—60%, ketoconazole (intravaginal)—55%.

**Conclusions:** The ability to local and systemic production of interferon-γ has been reduced in patients with recurrent vulvovaginal candidiasis.

## P417 Evaluation of a Liquid Media MALDI-TOF MS Protocol for the Identification of Dermatophytes Isolated from *Tinea capitis* Infections

**Pauline Lecerf ^1^,** Roelke De Paepe ^2^**, Yasaman Jazaeri ^1^, Anne-Cécile Normand ^3^ and Ann Packeu ^2,4^**

^1^ Université Libre de Bruxelles, Faculty of Medicine
^2^ Sciensano, Service of Mycology and Aerobiology
^3^ AP-HP, Hôpitaux de Paris, Service of Parasitology/Mycology
^4^ BCCM/IHEM Fungal Collection, Sciensano, Mycology and Aerobiology Section

**Background:** *Tinea capitis* is a common childhood superficial infection of the scalp caused by dermatophytes. Conventional methods based on morphological characteristics to identify theses fungi are time-consuming and complex, requiring expert mycological knowledge. Recently, MALDI-TOF MS has become a powerful tool in clinical microbiology for rapid identification of these microorganisms.

**Objectives:** The main purpose is a shorter turnaround time for the identification of dermatophyte species responsible for *Tinea capitis* infection by using a liquid media MALDI-TOF protocol and eliminating sub-culturing steps. Identification techniques (conventional identification and MALDI-TOF MS based on liquid and solid cultures) were evaluated and compared for their rate of correct identification and turnaround time.

**Materials & Methods:** First, the in-house BCCM/IHEM database made from references strains on solid medium was used to validate the liquid media MALDI-TOF MS protocol. Secondly, the same protocol was applied on both sub-cultures and primary isolates of clinical isolates collected from the Saint-Pierre University Hospital. The liquid protocol was evaluated in parallel with the solid protocol. For the primary isolates, identifications with MALDI-TOF MS were initially performed when the cultures had sufficiently grown. Additionally, cultures were identified at fixed points in time, i.e., 3, 7, 14 and 21 days after inoculation.

**Results:** The use of the liquid media MALDI-TOF MS protocol resulted in a rate of 100% of correct identification at species level for reference strains and 76.9% for clinical isolates with sub-culturing step. The turnaround time for this method was 6 days, which was statistically significantly faster than the conventional method. When applied on primary isolates, the rate of correct identification was only 63.4%, with a turnaround time of 12 days. The complete extraction from the solid media MALDI-TOF MS protocol still gave the highest correct species identification with the highest mean of log-scores.

**Conclusions:** The liquid media MALDI-TOF MS technique is an accurate method for the correct identification of dermatophytes at the species level and is much faster than the conventional technique, but a sub-culturing step for clinical samples is necessary to obtain reliable identifications and to limit the turnaround time. For the identification of closely related species, the complete extraction from solid media MALDI-TOF MS protocol remains the most reliable technique.

## P418 Changing Trends of Dermatophytosis in Greece: A 10-Year Survey in a Tertiary Care Academic Hospital. EMERGENCE of Terbinafine-Resistant Trichophyton Mentagrophytes

**Maria Siopi ^1^, Ioanna Efstathiou ^1^, Konstantinos Theodoropoulos ^2^, Spyros Pournaras ^1^ and Joseph Meletiadis ^1^**

^1^ Clinical Microbiology Laboratory, “Attikon” University General Hospital, Medical School, National and Kapodistrian University of Athens
^2^ 2nd Department of Dermatology & Venereology, “Attikon” University General Hospital, Medical School, National and Kapodistrian University of Athens

**Objectives:** Population mobility, climate/socio-economic changes and rampant use of over-the-counter topical antifungals affect dermatophytosis epidemiology globally and locally. Data on the contemporary epidemiology of dermatophytes in Greece are scarce. We therefore conducted a retrospective study to assess changes in incidence rates, species distribution and antifungal susceptibility patterns of dermatophytosis in a tertiary care academic hospital over the past decade.

**Materials/methods:** Samples from patients with clinically suspected dermatophytosis attending the outpatient Dermatology-Venereology Department of “Attikon” hospital during 2010–2019 were collected. Specimens were processed for direct microscopic examination (Blankophor in 10% KOH) and were inoculated on two plates each of Sabouraud’s dextrose agar supplemented with gentamicin + chloramphenicol and the other containing phenol red + cyclohexamide (incubation at 30 °C up to 4 weeks). Recovered isolates were identified to the genus and species level by standard phenotypic methods based on their colonial/microscopic morphology and biochemical properties (hydrolysis of urea) and were stored in 10% glycerol stocks at −70 °C. *Trichophyton* spp. isolates were retrospectively molecularly identified (restriction fragment length polymorphism analysis of the ITS region by MvaI restriction enzyme, Sanger sequencing of the ITS region) and were subjected to in vitro susceptibility testing to terbinafine, itraconazole, voriconazole and amorolfine following the recently proposed EUCAST E.DEF11.0. The target gene squalene epoxidase (SQLE) was sequenced for isolates with reduced susceptibility to terbinafine.

**Results:** A total of 1360 samples from 1238 patients were examined. Dermatophytes were recovered from 200 (16%) patients, whereof 104 (52%) were male of median (range) age 43 (0.8–83) years. The overall (range) annual incidence of dermatophytosis was 16% (9–21%) showing a significant increase between the years 2010–2013 and 2014–2019 (12% vs. 18%, *p* = 0.009). Among the 218 (16%) positive samples, 101 (46%) were skin scrapings, 99 (45%) nail clippings and 18 (8%) hair. The predominant dermatophyte was *T. rubrum* (100; 46%), followed by *M. canis* (50; 23%), *T. mentagrophytes* (28; 13%), *T. interdigitale* (22; 10%), *T. tonsurans* (6; 2%) and other rare species (12; 6%). A significant increase of *M. canis* (6%, 23%, 30%, *p* = 0.001) accompanied by a paralell decrease of *T. rubrum* (55%, 57%, 35%, *p* = 0.0009) was noted during the years 2010–2013, 2014–2016 and 2017–2019, respectively. The agreement between direct microscopy and culture was 82%. 25% of *Trichophyton* spp. isolates failed to grow despite the repeated efforts to revive them. All *T. rubrum* (*n* = 70)*, T. interdigitale* (*n* = 12) and *T. tonsurans* (*n* = 6) isolates were classified as wild-type (WT) to all antifungals. In contrast, 9/24 (37.5%) *T. mentagrophytes* strains displayed elevated terbinafine MICs (0.25–8 mg/L) but not to azoles and amorolfine, all belonged to ITS Type VIII, harboured Leu393Ser (*n* = 5) and Phe397Leu (*n* = 4) SQLE mutations and were isolated from 7 patients during the last 2 years.

**Conclusions:** *T. rubrum* was the predominant dermatophyte, while a significant upward trend for *M. canis* was noted indicating that the periodic review of species distribution is critical to proper dermatophytosis management. Worryingly, high incidence (37.5%) of terbinafine non-WT *T. mentagrophytes* isolates without cross-resistance to other antifungals was found for the first time in Greece. This finding must alarm for susceptibility testing at a local scale particularly in non-responding dermatophytoses.

**Keywords:** dermatophytosis epidemiology; resistance; Greece

## P419 Development and Single-Centre Validation of an Agar-Based Screening Method for Terbinafine, Itraconazole and Amorolfine Susceptibility Testing of *Trichophyton* spp.

**Maria Siopi ^1^, Ioanna Efstathiou ^1^, Maiken C. Arendrup ^2,3,4^, Spyros Pournaras ^1^ and Joseph Meletiadis ^1^**

^1^ Clinical Microbiology Laboratory, “Attikon” University General Hospital, Medical School, National and Kapodistrian University of Athens
^2^ Unit of Mycology, Statens Serum Institut
^3^ Department of Clinical Microbiology, Rigshospitalet
^4^ Department of Clinical Medicine, University of Copenhagen

**Objectives:** Resistance in dermatophytes and particularly in *Trichophyton* spp. has emerged as a global public health issue. The EUCAST has recently released a new microdilution method for susceptibility testing of microconidia-forming dermatophytes. Nevertheless, in vitro susceptibility testing of dermatophytes with a microdilution method is not feasible in all routine laboratories, thereby delaying detection of drug resistance in clinical practice and hindering the determination of the actual burden of antifungal resistance. Based on these grounds, we developed an agar-based screening method for susceptibility testing of *Trichophyton* spp. and subsequently validated its performance using molecularly characterised clinical isolates.

**Materials/Methods:** *Method development:* A total of 9 terbinafine wild-type (WT) *T. rubrum* isolates and 8 terbinafine non-WT *T. rubrum* isolates (MIC 0.125- > 4 mg/L) with different mutations in the squalene epoxidase (SQLE) gene (F397L, L393S, L393F, I121M/V237I) (Arendrup *JAC* 2020) were used for optimization of test conditions. The following test condition ranges were investigated: inoculum concentration (0.5–2 McF), incubation time (4–6 days) and incubation temperature (25–37 °C). The optimal concentrations of terbinafine, itraconazole and amorolfine were evaluated using increasing 2-fold concentrations (0.008–1 mg/L) and an absence of growth endpoint. Stability of agar plates was assessed with the isolates *T. rubrum* SSI-7583 and SSI-7885 (terbinafine MIC 0.016 and >4, respectively) up to 6 months. Inter-experimental variation was evaluated by testing 10 isolates on three different days. *Single-centre validation*: After determining the optimal conditions for the agar screening method, 112 molecularly identified *Trichophyton* spp. clinical isolates were tested. All *T. rubrum* (*n* = 70), *T. interdigitale* (*n* = 12) and *T. tonsurans* (*n* = 6) isolates were classified as wild-type (WT) to all antifungals, while 9/24 *T. mentagrophytes* strains displayed elevated terbinafine MICs (0.25–8 mg/L) based on the EUCAST reference methodology (E.DEF 11.0) and harboured L393S and F397L SQLE mutations.

**Results:** *Method development:* Optimal growth of drug-free controls was observed using an inoculum of 0.5 McF after 5 days of incubation at 30 °C. The optimal concentrations that prevented the growth of WT isolates were 0.01 and 0.125 mg/L of terbinafine for low-level (terbinafine MIC 0.125 mg/L) and high-level (terbinafine MIC 2-> 4 mg/L) resistant isolates, respectively, 1 mg/L of itraconazole and 0.5 mg/L of amorolfine. Variation between experiments was not observed (100% agreement) and the agar plates were stable for up to 6 months. *Single-centre validation:* The method successfully discriminated WT from non-WT *Trichophyton* spp. clinical isolates showing 100% agreement with the reference broth microdilution methodology (Figure 1).

**Conclusions:** The developed agar-based method is suitable for in vitro susceptibility screening of *Trichophyton* spp. and can be used to detect terbinafine non-WT isolates. Such a screening test will be easy to implement and facilitate detection of resistant isolates in non-expert routine diagnostic laboratories.

**Keywords:** agar screening; resistance; *Trichophyton* spp.


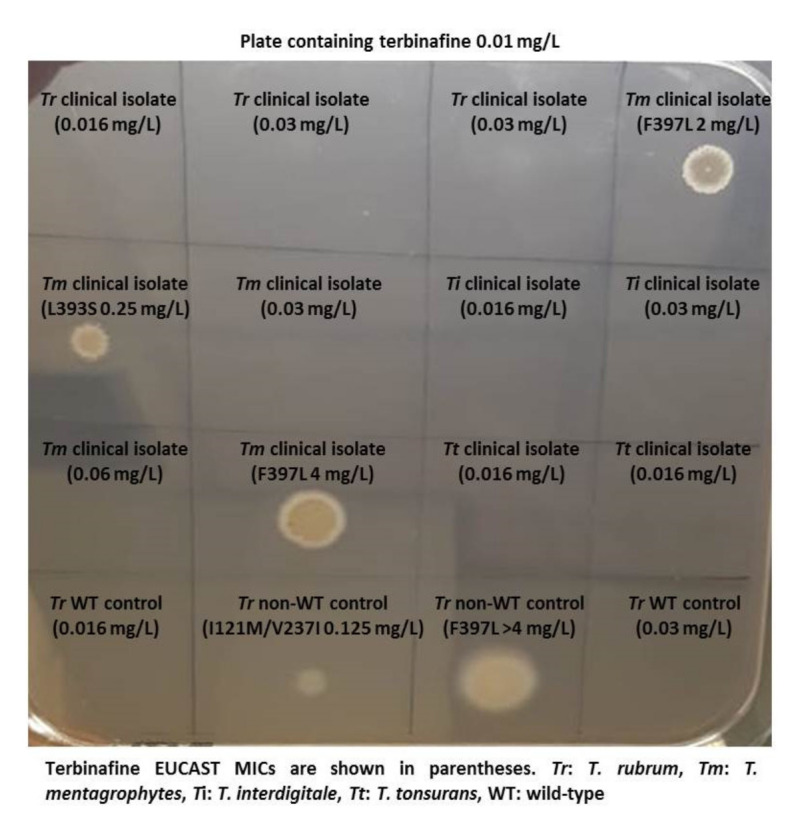


**Figure 1.** The method successfully discriminated WT from non-WT *Trichophyton* spp. clinical isolates showing 100% agreement with the reference broth microdilution methodology.

## P420 Psoriasiform *Tinea capitis* due to *Microsporum canis*: Atypical Presentation

**Maria Lúcia Scroferneker ^1,2^, Rodrigo Vetoratto ^1,3^, Alessandra Koehler ^1^, Joana Roberta Fitz ^3^, Andréa Abê Pereira ^3^, Paulo Cezar de Moraes ^1,4^, Amanda Carvalho Ribeiro ^5^ and Danielle Machado Pagani ^6^**

^1^ Postgraduate Program in Medicine: Medical Sciences, Universidade Federal do Rio Grande do Sul
^2^ Department of Microbiology, Immunology and Parasitology, ICBS, Universidade Federal do Rio Grande do Sul
^3^ Dermatology Service from the Hospitalar Complex Santa Casa de Misericórdia de Porto Alegre
^4^ Sanitary Dermatology Outpatient Clinic from Porto Alegre
^5^ Graduation in Pharmacy, Universidade Federal do Rio Grande do Sul
^6^ Postgraduate Program in Agricultural and Environmental Microbiology, Universidade do Rio Grande do Sul

**Case Report:** A 66-year-old female patient sought care at the dermatology outpatient clinic in December 2019 with complaints of itchy wounds on the scalp, which appeared about 10 months ago. She reported being on long-term prednisone for the treatment of rheumatoid arthritis, simvastatin for dyslipidemia and nesin for type 2 diabetes mellitus. She had already been treated numerous times for psoriasis or seborrheic dermatitis, as shown in previous prescriptions. Upon examination, she presented areas of diffuse alopecia, not tonsurant, in erythematous and desquamative plaques, with a psoriasiform aspect, mainly in the right temporal region. Dermoscopy identified the presence of numerous pustules and rare corkscrew hairs. The examination with Wood’s lamp in the alopecia area showed blue-green fluorescence. A direct mycological examination of the lesions was performed, with the identification of dermatophyte fungi colonizing the patient’s hair (ecto-endotrix colonization). The cultural mycological examination revealed a white filamentous colony, with yellowish reverse, suggesting that it was *Microsporum canis*. In microculture, in addition to septate hyaline hyphae, the presence of numerous thick-walled spindle-shaped macroconidia, compatible with *M. canis*, was identified. For molecular identification, DNA extraction and genetic sequencing of the ITS region was performed, with confirmation of *M. canis* through the GenBank basic local alignment search tool (BLAST) searches. The treatment instituted was terbinafine, orally, 250 mg/day. The patient showed complete improvement of the lesions after the use of oral terbinafine for 3 months.

**Discussion and Conclusions:** tinea capitis is more common in children than adults and the main etiologic agent is the fungus *Microsporum canis*. Immunosuppression is a risk factor for dermatophytosis. There are three main forms of tinea capitis. The tonsurant form may be microsporic or trichophytic. Both have tonsured hair and flaking. However, the first usually has a single lesion, blue-green fluorescence and ectotrix-like colonization, while the second has several minor lesions, without fluorescence and endotrix-like colonization. The kerion type inflammatory form is characterized by painful inflammatory plastron, accompanied by regional adenopathy. The lesion is unique and well-defined, covered with numerous pustules that drain purulent secretion. The honeycomb form is rare and presents an inflammatory pattern with the formation of yellowish crusts in the shape of a shield around the hair follicles and keratotic crusts that contain hyphae and can be highly infectious. In this form, fluorescence is yellowish. In adults, tinea capitis is more common in elderly females. In this population, the clinical presentation is usually atypical and variable, being able to simulate other dermatological diseases such as seborrheic dermatitis, psoriasis, contact dermatitis or bacterial folliculitis. In our case, the patient did not present findings typical of any of the three main forms of tinea capitis. This atypical presentation highlights the importance of differential diagnosis, especially in populations that are not normally affected by this dermatophytosis.

## P421 Investigation of Yeast Agents Colonising the Scalp and Associations of Non-Microbial Causative Factors in Dandruff Sufferers

**Mümtaz Güran**

Eastern Mediterranean University

**Objectives:** Dandruff is a common scalp disorder that nearly half of the healthy population is affected. The disorder causes physical discomfort and has a negative impact on self-esteem. Multiple agents were shown to involve the etiopathology of Dandruff, but it is not fully understood. In this study we aim to investigate the associations of microbial (yeasts) and non-microbial causative agents of Dandruff among male university students, in Northern Cyprus.

**Materials & Methods:** The sample pool was generated randomly by including 241 voluntary male students by convenience sampling method. Self-administered questionnaires consisting 2 demographical and 34 causative agent-related questions were used to collect data for non-microbial causative agents. Samples for yeast observation were collected from scalp of voluntary participants by using sterilized dry cotton swabs. Meantime, presence of Dandruff was identified by analysing inflammation of the scalp in the form of red, scaly, itchy and flaking rash and the severity was graded as mild, moderate and severe dandruff. Yeast agents including were detected by using CHROMagarTM Malassezia Chromogenic Media. Biostatistical methods were used to analyse associations.

**Results:** 210 participants (88%) were detected to suffer from Dandruff while 6% suffered from severe Dandruff. Among people who had Dandruff, 45.6% had colonisation with at least one of the yeast species tested (Table 1). Among the tested non-microbial factors, hair type, having long hair and shower frequency showed a significant positive correlation with having Dandruff (*p* = 0.047, *p* = 0.036 and *p* = 0.007 respectively).

**Conclusions:** In the current study, hair type, having long hair and shower frequency have been shown to be significantly associated with dandruff. Also, yeasts predominantly being *Malassezia* spp. were observed to be colonising scalp flora significantly higher in dandruff sufferers. Taken together, identifying the significant causative agents for Dandruff may provide evidence to prevent and develop novel treatment strategies for scalp disorders.

**Table 1.** The frequencies of tested microbial causative-agents among people who have Dandruff.


***Malassezia* spp.**

***Malassezia furfur***

***Candida albicans***

***Candida krusei***

***Candida tropicalis***

**Other Yeast Species**
21.2%13.3%3.3%1.2%4.1%2.9%

## P425 A Polyphasic Approach to Classification and Identification of Species within the *Trichophyton benhamiae* Complex

**Frederik Baert ^1,2^, Paulien Lefevere ^1^, Elizabet D’hooge ^1,2^, Dirk Stubbe ^1,2^ and Ann Packeu ^1,2^**

^1^ Sciensano, Service of Mycology and Aerobiology
^2^ BCCM/IHEM fungi collection, Mycology & Aerobiology, Sciensano

**Objectives:** In recent years many advances have been made in clearing up the phylogenetic relationships within the family *Arthrodermataceae*. However, certain closely related taxa still contain poorly resolved species boundaries. In this study we try to elucidate the species composition of the *Trichophyton benhamiae* species complex.

**Materials & Methods:** A polyphasic approach is used consisting of multi-gene phylogenetic analysis based on internal transcribed spacer (ITS) and beta-tubulin (BT) gene regions, morphological analysis and spectral comparison using MALDI-ToF.

**Results:** We confirm the existence of 11 different monophyletic clades within the complex representing either species or genetically distinct groups within species. MALDI-ToF spectrometry analysis revealed that most of these clades were readily distinguishable from one another, however some closely related sisterclades such as *T. europaeum* and *T. japonicum* were often misidentified as their counterpart. The distinct “yellow” and “white” phenotypes of *T. benhamiae* do not have a clear genetical basis and should thus be considered as different morphotypes of the same species.

**Conclusions:** Strains traditionally considered *T. benhamiae* can be divided into three main clades: (i) *T. benhamiae*, (ii) *T. europaeum/T. japonicum* and (iii) the phylogenetically distant *T. africanum*. While *T. europaeum* and *T. japonicum* are distinguishable based on their genotype, spectral and morphological analysis did not provide clear delimiting characteristics.

## P426 The Taxonomy of the *Trichophyton rubrum* Complex: A Phylogenomic Approach

**Pierre Becker ^1^, Luc Cornet ^1^, Elizabet D’hooge ^1^, Nicolas Magain ^2^, Dirk Stubbe ^1^, Ann Packeu ^1^ and Denis Baurain ^2^**

^1^ Sciensano
^2^ University of Liège

**Objectives:** The medically relevant *Trichophyton rubrum* species complex has a variety of phenotypic presentations, but shows relatively little genetic differences. Conventional barcodes, such as the internal transcribed spacer (ITS) region or the beta-tubulin (BT) gene, are not able to completely resolve the relationships between these closely related taxa. *Trichophyton rubrum, T. soudanense* and *T. violaceum* are currently accepted as separate species. However, the status of certain variants, including the *T. rubrum* morphotypes *megninii* and *kuryangei* and the *T. violaceum* morphotype *yaoundei* remains to be deciphered.

**Materials & Methods:** We conducted the first phylogenomics analysis of the *Trichophyton rubrum* species complex by studying 3105 core genes of 18 strains from the BCCM/IHEM culture collection and nine publicly available genomes. Robinson-Foulds distances were also calculated to determine potential new markers suitable to accurately distinguish species belonging to the complex.

**Results:** The analyses resulted in a fully resolved phylogenetic tree with five separate clades. *Trichophyton rubrum, T. violaceum* and *T. soudanense* were confirmed in their status as species. The morphotypes *megninii* and *kuryangei* both grouped in their own respective clade with high support, next to *T. soudanense*, suggesting that these morphotypes should be considered as separate species. Strains of the *yaoundei* morphotype were intermingled with *T. violaceum* strains in one supported clade, indicating that *T. yaoundei* is a phenotypic variant of *T. violaceum*. Robinson-Foulds analyses revealed that a combination of two markers (a ubiquitin-protein transferase and a MYB DNA-binding domain-containing protein) could mirror the phylogeny obtained using genomic data.

**Conclusions:** In total, five species are proposed to be accepted in the *T. rubrum* complex, i.e., *T. rubrum, T. soudanense, T. violaceum, T. megninii* and *T. kuryangei*. The two new suggested marker genes could help distinguishing strains belonging to this complex in future molecular identifications.

